# Supplementary material for: Genome-wide investigation of the AP2/ERF superfamily and their expression under salt stress in Chinese willow (Salix matsudana)
Source: PeerJ. 2021 Apr 13;9:e11076. doi: 10.7717/peerj.11076 (PMC8051338; doi:10.7717/peerj.11076)
Supplement: Supplemental Information 1 [file peerj-09-11076-s001.docx]

Genome-wide investigation of the AP2/ERF superfamily and their expression under salt stress in Chinese willow (*Salix matsudana*)

Running title: AP2/ERF superfamily in Chinese willow

Jian Zhang^1a^, Yuna Jiang^1a^, Shi zheng Shi^2a^, Fei Zhong^1^, Guoyuan Liu^1^, Chunmei Yu^1^, Bolin Lian^1^, Yanhong Chen ^1^*

^1^Lab of Landscape Plant Genetics and Breeding, School of Life Science, Nantong University, Nantong, Jiangsu Province, China

^2^ Jiangsu Academy of Forestry，Nanjing 211153，China

^a^These authors contribute equally to this work

*Corresponding author. E-mail: [chenyh@ntu.edu.cn](mailto:chenyh@ntu.edu.cn) ; FAX: 86-513-85012818; Tel: 86-513-85012818

E-mail address for other authors

Jian Zhang: 56071007@qq.com

Yuna Jiang :[18252098108@163.com](mailto:18252098108@163.com);

Shizheng Shi: shshzn@163.com

Fei Zhong: [fzhong@ntu.edu.cn](mailto:fzhong@ntu.edu.cn);

Guoyuan Liu: [cjqm1989@126.com](mailto:cjqm1989@126.com);

Chunmei Yu: [ychmei@ntu.edu.cn](mailto:ychmei@ntu.edu.cn);

Bolin Lian: [lianziadd9@163.com](mailto:lianziadd9@163.com);

**Table S1 qRT-PCR Primers list**

| Gene name | Primer name | Primer Sequence |
| --- | --- | --- |
| SmAP2-33 | EVM0043933-1F | AATGAAAGCAGCCACAGTGG |
| SmAP2-38 | EVM0043933-1R  EVM0028655-2F  EVM0028655-2R | TTACCATGCAAGGCAATGGG  TGGCAAGCTAGAATAGGAAGGG  ATTGCAGCAACGTCATAGGC |
| SmAP2-15 | EVM0036052-3F | TTCTTCGCCGTCAAAGCAAC |
|  | EVM0036052-3R | AATTGCCCCATTCGAGCTTC |
| SmDREB A4-24 | EVM0040155-4F | TCAACTTCCCCAAACTTGCC |
|  | EVM0040155-4R | TGTTGAAGTCCATGGAAGCG |
| SmDREB A1-4 | EVM0040623-5F | TGCCTGCGCCAATATCAAAC |
|  | EVM0040623-5R | ATACCCCGCATTTTCAGACG |
| SmDREB A1-7 | EVM0032370-6F | TGTGTGGGAGGTGAGTTGATG |
|  | EVM0032370-6R | AAGCCTGGCATCCCAAAAAC |
| SmDREB A5-23 | EVM0003861-7F | TTTTTGCTTGCGTGGTCGAG |
|  | EVM0003861-7R | TCCGAATTGGCAAACTTCGC |
| SmDREB A6-6 | EVM0000460-8F | TCGAGCGATAATTGGGGTTG |
|  | EVM0000460-8R | AGAGGACAATCTGCAGACACAG |
| SmERF B3-29 | EVM0024412-9F | AGTCCGTTCGCTTGCATTTG |
|  | EVM0024412-9R | GGTTGTTGTTGAGACTGCACTG |
| SmERF B3-45 | EVM0052610-10F | AGCGGTCTTTGAATCGGATC |
|  | EVM0052610-10R | TAATCTCCGCCGCATACTTCC |
| SmERF B4-19 | EVM0035905-11F | ATGCGAAAGAAGAGGAAGCG |
|  | EVM0035905-11R | TGCACTATCAAAGGTCCCTAGC |
| SmERF B3-42 | EVM0054964-13F | AAAATGAGGGGCAGGAAAGC |
|  | EVM0054964-13R | TTCTCTTCCTTCCTGTGGTTGG |
| SmERF B4-1 | EVM0002769-14F | GGTTGCTGCTCTCAAAAACG |
|  | EVM0002769-14R | ATCGTTCCCGTTACTGCTTG |
| SmERF B4-19 | EVM0000045-15F | AAAGAAACCGGTGCAGCAAC |
|  | EVM0000045-15R | AGTTGCACCAGATGACAACG |
| SapurV1A.0655s0050.1 | Sp Actin1-Q-F | GTCAAGTTCTTTGCTTTCCTCC |
|  | Sp Actin1-Q-F | CATCACAATCACTCTCCGACTA |

T**able S2 Proteins length, MW and PI of 364 SmAP2/ERF family members**

| #ID | Name | length | MW(Da) | pI |
| --- | --- | --- | --- | --- |
| EVM0031439 | SmERF B1-32 | 281 | 31037.4 | 6.07 |
| EVM0013188 | SmDREB A4-36 | 257 | 28380 | 6.6 |
| EVM0020531 | SmERF B3-52 | 327 | 36693.5 | 5.76 |
| EVM0037606 | SmERF B3-53 | 283 | 30573.1 | 8.05 |
| EVM0029808 | SmDREB A1-8 | 329 | 36846.8 | 4.5 |
| EVM0018598 | SmDREB A4-31 | 178 | 18997.9 | 4.77 |
| EVM0054100 | SmERF B1-2 | 205 | 22449.9 | 9.53 |
| EVM0052580 | SmDREB A1-1 | 272 | 30169.7 | 8.15 |
| EVM0007543 | SmERF B1-18 | 143 | 16471.2 | 6.8 |
| EVM0040155 | SmDREB A4-24 | 292 | 31384.9 | 4.56 |
| EVM0054412 | SmERF B1-33 | 293 | 32221.6 | 4.68 |
| EVM0026277 | SmERF B4-17 | 234 | 26126.5 | 6.27 |
| EVM0027008 | SmDREB A5-3 | 222 | 24605.1 | 9.23 |
| EVM0034096 | SmDREB A4-4 | 250 | 27614.3 | 5.36 |
| EVM0002257 | SmDREB A5-4 | 165 | 18654 | 9.45 |
| EVM0043935 | SmERF B6-5 | 288 | 31569.9 | 7.75 |
| EVM0016170 | SmERF B6-17 | 268 | 30414.5 | 5.48 |
| EVM0027493 | SmERF B6-8 | 291 | 31724.1 | 8.76 |
| EVM0051566 | SmERF B4-8 | 213 | 23037.9 | 6.77 |
| EVM0057361 | SmERF B4-9 | 212 | 22656.5 | 5.66 |
| EVM0018988 | SmERF B6-18 | 297 | 32161 | 10.09 |
| EVM0006672 | SmDREB A4-11 | 250 | 26572.1 | 6.98 |
| EVM0009316 | SmDREB A1-3 | 248 | 27046.1 | 4.8 |
| EVM0009672 | SmAP2-9 | 350 | 39368.9 | 6.79 |
| EVM0011072 | SmDREB A2-7 | 180 | 20318.6 | 7.62 |
| EVM0017689 | SmDREB A2-8 | 237 | 26390.3 | 6.27 |
| EVM0023967 | SmAP2-16 | 411 | 46334.8 | 6.33 |
| EVM0046201 | SmDREB A5-7 | 176 | 19944.9 | 10.34 |
| EVM0008767 | SmAP2-6 | 515 | 58268.1 | 8.52 |
| EVM0044116 | SmDREB A1-6 | 273 | 30258.5 | 7.66 |
| EVM0042515 | SmAP2-51 | 480 | 52740.9 | 8.3 |
| EVM0015962 | SmDREB A4-1 | 199 | 21382.6 | 4.73 |
| EVM0042416 | SmDREB A4-12 | 161 | 17962.1 | 4.79 |
| EVM0016569 | SmDREB A4-13 | 179 | 19854.9 | 5.61 |
| EVM0043720 | SmERF B1-1 | 143 | 16436.2 | 6.8 |
| EVM0034620 | SmERF B5-5 | 332 | 36647.3 | 6.17 |
| EVM0038704 | SmDREB A6-7 | 294 | 32572.5 | 9.65 |
| EVM0006649 | SmERF B3-15 | 249 | 27778.1 | 6.96 |
| EVM0041820 | SmERF B3-16 | 266 | 28987.3 | 9.81 |
| EVM0055475 | SmERF B3-17 | 228 | 25463.9 | 4.84 |
| EVM0002852 | SmDREB A4-7 | 164 | 18080.2 | 9.01 |
| EVM0001533 | SmDREB A2-11 | 457 | 51394.8 | 6.44 |
| EVM0056869 | SmDREB A2-12 | 207 | 23050.8 | 8.5 |
| EVM0057140 | SmDREB A2-13 | 275 | 31504 | 6.11 |
| EVM0015665 | SmAP2-25 | 433 | 47879.9 | 8.97 |
| EVM0012873 | SmERF B3-47 | 148 | 16878.6 | 6.04 |
| EVM0033385 | SmDREB A2-1 | 456 | 50372.3 | 5.03 |
| EVM0018037 | SmERF B1-3 | 216 | 23525 | 9.06 |
| EVM0002769 | SmERF B4-1 | 263 | 29350.6 | 8.31 |
| EVM0043678 | SmERF B4-3 | 268 | 29714.2 | 8.49 |
| EVM0030823 | SmERF B6-2 | 271 | 30868 | 5.48 |
| EVM0003762 | SmERF B1-20 | 218 | 23830.3 | 9.69 |
| EVM0008628 | SmDREB A4-2 | 198 | 21873.1 | 5.12 |
| EVM0054355 | SmERF B5-2 | 307 | 35142.6 | 5.18 |
| EVM0046342 | SmERF B6-14 | 266 | 29480.9 | 8.8 |
| EVM0017324 | SmERF B2-8 | 315 | 35645.5 | 5.06 |
| EVM0052698 | SmDREB A2-24 | 452 | 50934.3 | 5.85 |
| EVM0008451 | SmAP2-52 | 528 | 58413.9 | 6.63 |
| EVM0021344 | SmDREB A4-8 | 230 | 25039.8 | 6.78 |
| EVM0049776 | SmDREB A6-3 | 337 | 37467.3 | 6.51 |
| EVM0037879 | SmDREB A5-2 | 217 | 23855.5 | 7.24 |
| EVM0003769 | SmDREB A6-4 | 351 | 39006.1 | 6.29 |
| EVM0030239 | SmERF B3-41 | 282 | 30071.7 | 7.85 |
| EVM0040670 | SmERF B3-40 | 353 | 39480.7 | 5.74 |
| EVM0030192 | SmERF B3-39 | 353 | 39698.9 | 5.74 |
| EVM0051058 | SmDREB A4-30 | 220 | 24517.9 | 6.96 |
| EVM0018852 | SmERF B1-22 | 271 | 30351.1 | 5.83 |
| EVM0023232 | SmERF B2-9 | 375 | 41232.4 | 4.73 |
| EVM0028655 | SmAP2-38 | 567 | 63839.5 | 8.58 |
| EVM0024365 | SmDREB A4-15 | 159 | 17045 | 5.78 |
| EVM0040623 | SmDREB A1-4 | 216 | 24044.5 | 5.17 |
| EVM0026062 | SmDREB A1-5 | 231 | 26314.1 | 6.25 |
| EVM0017041 | SmDREB A2-27 | 194 | 22020.8 | 9.15 |
| EVM0008598 | SmDREB A2-26 | 191 | 21521.1 | 8.86 |
| EVM0054855 | SmERF B1-9 | 416 | 45323.4 | 7.39 |
| EVM0028978 | SmERF B2-6 | 312 | 35336.9 | 6.01 |
| EVM0007646 | SmERF B6-9 | 287 | 31957.4 | 7.95 |
| EVM0035153 | SmAP2-27 | 544 | 60153.8 | 6.97 |
| EVM0056670 | SmAP2-53 | 411 | 46368.7 | 6.14 |
| EVM0049616 | SmDREB A3-2 | 397 | 43363.6 | 9.65 |
| EVM0019343 | SmERF B1-26 | 419 | 46025.1 | 6.83 |
| EVM0050425 | SmAP2-17 | 601 | 67126.2 | 7.27 |
| EVM0012824 | SmAP2-18 | 507 | 55726.7 | 6.89 |
| EVM0040310 | SmAP2-8 | 361 | 40996.8 | 7.76 |
| EVM0054250 | SmDREB A2-6 | 181 | 20340.7 | 8.38 |
| EVM0013578 | SmERF B4-5 | 230 | 25819.1 | 5.68 |
| EVM0039609 | SmRAV-5 | 363 | 40915.6 | 9.35 |
| EVM0001548 | SmDREB A5-15 | 170 | 18435.3 | 6.96 |
| EVM0043548 | SmAP2-20 | 367 | 40853.1 | 6.71 |
| EVM0002934 | SmSol-1 | 232 | 26081.1 | 10.42 |
| EVM0054778 | SmDREB A4-37 | 208 | 22117.4 | 4.81 |
| EVM0013126 | SmDREB A6-8 | 374 | 41409.9 | 6.6 |
| EVM0026052 | SmERF B5-11 | 290 | 33229.5 | 4.83 |
| EVM0034397 | SmDREB A4-38 | 197 | 21696 | 5.09 |
| EVM0013573 | SmDREB A1-10 | 248 | 27125.1 | 4.79 |
| EVM0004316 | SmDREB A4-39 | 251 | 26694.3 | 6.69 |
| EVM0036425 | SmERF B6-19 | 312 | 34581.9 | 4.37 |
| EVM0033748 | SmRAV-6 | 369 | 41357 | 9.47 |
| EVM0006637 | SmAP2-43 | 545 | 59276.5 | 8.03 |
| EVM0019656 | SmAP2-45 | 347 | 39414.6 | 5.87 |
| EVM0040690 | SmDREB A4-17 | 181 | 20018.2 | 5.63 |
| EVM0055308 | SmDREB A4-33 | 297 | 32857 | 5.44 |
| EVM0052610 | SmERF B3-45 | 190 | 21000.2 | 6.78 |
| EVM0053966 | SmERF B3-46 | 202 | 22709.3 | 8.66 |
| EVM0006944 | SmERF B3-54 | 226 | 25678.2 | 5.16 |
| EVM0044050 | SmDREB A4-21 | 190 | 20672.1 | 8.49 |
| EVM0030182 | SmDREB A6-18 | 457 | 50276.7 | 7.17 |
| EVM0000006 | SmERF B6-20 | 297 | 32064 | 10.06 |
| EVM0025220 | SmERF B3-18 | 141 | 15999.4 | 6.52 |
| EVM0035905 | SmERF B3-19 | 228 | 25612 | 4.72 |
| EVM0045646 | SmERF B3-20 | 238 | 26353.8 | 4.81 |
| EVM0055887 | SmDREB A2-15 | 277 | 30253.1 | 5.21 |
| EVM0022735 | SmAP2-31 | 724 | 79018.4 | 6.44 |
| EVM0040588 | SmAP2-28 | 350 | 40049 | 7.61 |
| EVM0049667 | SmERF B5-10 | 351 | 39698.9 | 6.53 |
| EVM0022064 | SmDREB A5-17 | 167 | 18078.9 | 8.49 |
| EVM0005180 | SmDREB A4-20 | 179 | 19845.9 | 5.6 |
| EVM0050604 | SmDREB A4-19 | 186 | 20572.1 | 5.1 |
| EVM0029243 | SmDREB A5-24 | 205 | 22654.3 | 4.4 |
| EVM0042683 | SmERF B1-8 | 291 | 31972.3 | 4.55 |
| EVM0017998 | SmERF B4-6 | 231 | 25705.1 | 5.84 |
| EVM0000535 | SmERF B1-34 | 229 | 25136.7 | 8.63 |
| EVM0044386 | SmERF B2-12 | 252 | 28393.7 | 7.03 |
| EVM0055399 | SmERF B1-28 | 416 | 45379.4 | 6.75 |
| EVM0049532 | SmERF B3-48 | 138 | 15465 | 7.51 |
| EVM0002921 | SmERF B3-49 | 148 | 16683.2 | 6.04 |
| EVM0025231 | SmERF B3-50 | 194 | 21864.5 | 9.58 |
| EVM0038570 | SmDREB A6-19 | 466 | 51574.6 | 7.75 |
| EVM0042411 | SmDREB A4-40 | 251 | 27796.6 | 5.14 |
| EVM0001696 | SmDREB A4-41 | 211 | 23069.5 | 4.61 |
| EVM0031168 | SmERF B5-12 | 358 | 39751.2 | 7.04 |
| EVM0034491 | SmERF B2-11 | 257 | 29346.8 | 8.92 |
| EVM0046023 | SmERF B1-27 | 339 | 37283.8 | 5.35 |
| EVM0023162 | SmERF B1-15 | 208 | 22657 | 9.34 |
| EVM0008654 | SmDREB A6-9 | 361 | 40273.7 | 6.74 |
| EVM0023217 | SmERF B4-2 | 458 | 48856.9 | 7.03 |
| EVM0022031 | SmERF B3-34 | 225 | 25552 | 4.68 |
| EVM0013555 | SmDREB A5-19 | 141 | 15685.4 | 8.04 |
| EVM0045980 | SmDREB A2-16 | 178 | 20173.8 | 9.23 |
| EVM0019845 | SmDREB A2-17 | 194 | 22076.9 | 8.8 |
| EVM0039499 | SmERF B2-1 | 314 | 35542.3 | 6 |
| EVM0004610 | SmERF B4-4 | 355 | 39288.4 | 6.64 |
| EVM0025179 | SmDREB A4-22 | 178 | 19844 | 6.69 |
| EVM0006866 | SmDREB A4-23 | 189 | 20822.3 | 4.53 |
| EVM0041252 | SmERF B5-13 | 301 | 34650.2 | 4.98 |
| EVM0042752 | SmDREB A2-29 | 210 | 23276.4 | 4.5 |
| EVM0020075 | SmERF B2-5 | 262 | 29282.7 | 8.19 |
| EVM0044977 | SmERF B6-3 | 301 | 32570.1 | 10.11 |
| EVM0051919 | SmDREB A6-2 | 458 | 50686.4 | 7.46 |
| EVM0045726 | SmERF B5-7 | 357 | 39427.2 | 6.09 |
| EVM0015466 | SmDREB A6-12 | 309 | 34314.2 | 7.98 |
| EVM0014688 | SmDREB A1-9 | 255 | 28021.3 | 5.07 |
| EVM0044087 | SmDREB A4-35 | 268 | 28750.3 | 5.95 |
| EVM0018678 | SmDREB A5-18 | 198 | 21835.4 | 4.63 |
| EVM0019323 | SmDREB A2-25 | 191 | 21347 | 9.58 |
| EVM0029410 | SmDREB A5-20 | 164 | 18480.3 | 8.67 |
| EVM0018397 | SmAP2-37 | 633 | 70233.4 | 7.16 |
| EVM0028452 | SmDREB A4-5 | 286 | 30839.4 | 4.57 |
| EVM0025980 | SmAP2-3 | 540 | 58827.8 | 7.32 |
| EVM0057600 | SmAP2-54 | 700 | 78130.1 | 6.72 |
| EVM0031973 | SmDREB A4-14 | 197 | 21668 | 6.26 |
| EVM0025365 | SmERF B6-4 | 262 | 28965.9 | 5.72 |
| EVM0000460 | SmDREB A6-6 | 309 | 34523.5 | 8.26 |
| EVM0024472 | SmERF B5-4 | 357 | 39346 | 4.88 |
| EVM0020564 | SmAP2-42 | 511 | 56398.9 | 7.25 |
| EVM0003861 | SmDREB A5-23 | 220 | 24726 | 4.68 |
| EVM0009928 | SmDREB A2-10 | 194 | 22193.9 | 9.06 |
| EVM0053305 | SmERF B1-10 | 221 | 24263.8 | 10.51 |
| EVM0014537 | SmDREB A6-17 | 308 | 34363.9 | 10.06 |
| EVM0054964 | SmERF B3-42 | 228 | 25447.9 | 4.75 |
| EVM0036702 | SmERF B3-43 | 265 | 29316.8 | 5.83 |
| EVM0054070 | SmERF B3-44 | 249 | 27618.9 | 7.69 |
| EVM0054822 | SmERF B3-55 | 283 | 30573.1 | 8.05 |
| EVM0043703 | SmERF B5-6 | 331 | 36933.1 | 6.54 |
| EVM0055401 | SmDREB A6-11 | 470 | 51732.3 | 6.18 |
| EVM0006682 | SmERF B1-19 | 225 | 24650.2 | 9.49 |
| EVM0047172 | SmERF B4-7 | 480 | 51987.9 | 9.6 |
| EVM0053799 | SmDREB A4-25 | 184 | 20440.7 | 4.53 |
| EVM0056893 | SmDREB A2-5 | 207 | 23126.9 | 8.09 |
| EVM0054439 | SmERF B3-11 | 143 | 16105.4 | 6.36 |
| EVM0001248 | SmERF B3-10 | 228 | 25482.8 | 4.53 |
| EVM0008755 | SmERF B3-9 | 240 | 26759.4 | 4.82 |
| EVM0011531 | SmAP2-4 | 686 | 76283.3 | 6.98 |
| EVM0049893 | SmDREB A5-5 | 155 | 17494.3 | 9.64 |
| EVM0035393 | SmERF B6-1 | 384 | 42403.6 | 4.59 |
| EVM0012647 | SmERF B5-1 | 320 | 36095.4 | 6.52 |
| EVM0029842 | SmDREB A6-10 | 368 | 41006.3 | 6.75 |
| EVM0018160 | SmDREB A4-18 | 230 | 25078.9 | 6.78 |
| EVM0051611 | SmERF B1-21 | 167 | 18712 | 8.48 |
| EVM0052364 | SmERF B3-35 | 168 | 18558.4 | 9.57 |
| EVM0017235 | SmERF B3-36 | 142 | 16218.6 | 7.26 |
| EVM0008128 | SmERF B3-37 | 226 | 25299.8 | 5.21 |
| EVM0018983 | SmERF B3-38 | 241 | 26883.7 | 6.26 |
| EVM0027529 | SmAP2-34 | 372 | 41249.8 | 7.81 |
| EVM0039676 | SmERF B1-4 | 154 | 17521.7 | 9.73 |
| EVM0027771 | SmERF B3-1 | 157 | 17269 | 5.83 |
| EVM0014312 | SmERF B3-2 | 142 | 16209.6 | 7.93 |
| EVM0040250 | SmERF B3-3 | 226 | 25330.9 | 5.05 |
| EVM0023703 | SmERF B3-4 | 242 | 26794.6 | 6.53 |
| EVM0000172 | SmDREB A2-2 | 179 | 19450.5 | 8.1 |
| EVM0044966 | SmERF B2-7 | 363 | 40310.3 | 4.78 |
| EVM0026368 | SmERF B1-14 | 273 | 30404.6 | 5.71 |
| EVM0036581 | SmAP2-44 | 366 | 41345.9 | 7.6 |
| EVM0022896 | SmERF B5-9 | 409 | 45784.8 | 5.39 |
| EVM0013798 | SmERF B1-35 | 350 | 38180.9 | 7.12 |
| EVM0003379 | SmDREB A2-20 | 185 | 20891.3 | 6.61 |
| EVM0045763 | SmERF B3-33 | 215 | 24039.4 | 5.2 |
| EVM0036069 | SmERF B3-32 | 148 | 16858.5 | 7 |
| EVM0012743 | SmERF B3-31 | 166 | 18852.8 | 8.97 |
| EVM0001106 | SmERF B4-18 | 349 | 38403.2 | 9.19 |
| EVM0002653 | SmERF B6-21 | 350 | 39051.2 | 4.74 |
| EVM0046641 | SmERF B3-28 | 264 | 28902.4 | 8.24 |
| EVM0006972 | SmERF B3-27 | 285 | 31314 | 7.34 |
| EVM0019924 | SmERF B3-26 | 233 | 26191.9 | 4.76 |
| EVM0032101 | SmERF B1-13 | 287 | 31633.1 | 4.73 |
| EVM0032029 | SmERF B4-12 | 160 | 17711.4 | 10.89 |
| EVM0051729 | SmDREB A6-20 | 452 | 50455.2 | 5.86 |
| EVM0044712 | SmERF B5-14 | 303 | 34733.9 | 5.32 |
| EVM0052641 | SmDREB A4-42 | 206 | 23036.6 | 7.3 |
| EVM0013502 | SmERF B4-15 | 242 | 26761.2 | 7.82 |
| EVM0032021 | SmDREB A4-34 | 252 | 27788.5 | 4.84 |
| EVM0005644 | SmDREB A4-27 | 264 | 28831.2 | 5.21 |
| EVM0045686 | SmERF B1-11 | 208 | 22804.4 | 9.75 |
| EVM0004890 | SmDREB A2-30 | 421 | 46469.2 | 5.89 |
| EVM0039575 | SmAP2-46 | 501 | 55441 | 8.21 |
| EVM0038265 | SmAP2-26 | 686 | 76247.3 | 6.69 |
| EVM0036168 | SmAP2-50 | 486 | 52838 | 7.54 |
| EVM0032156 | SmDREB A3-3 | 331 | 35352.4 | 7.46 |
| EVM0035890 | SmDREB A2-28 | 302 | 32889.8 | 4.87 |
| EVM0007616 | SmAP2-49 | 714 | 78035.8 | 6.54 |
| EVM0017733 | SmERF B5-3 | 318 | 35724.4 | 5.05 |
| EVM0000005 | SmDREB A4-3 | 257 | 28177.6 | 6.52 |
| EVM0055854 | SmERF B1-17 | 337 | 36837.2 | 6.09 |
| EVM0006536 | SmAP2-30 | 501 | 55578.3 | 8.53 |
| EVM0039635 | SmDREB A2-31 | 194 | 22084.9 | 8.8 |
| EVM0044041 | SmAP2-13 | 381 | 43645.3 | 9.65 |
| EVM0042467 | SmERF B1-16 | 383 | 41982.8 | 6.66 |
| EVM0032803 | SmAP2-12 | 668 | 73628.3 | 7.23 |
| EVM0016157 | SmDREB A5-12 | 210 | 23048.7 | 4.44 |
| EVM0051790 | SmDREB A5-25 | 256 | 28514.2 | 4.57 |
| EVM0043629 | SmDREB A6-5 | 371 | 41118.6 | 6.85 |
| EVM0044805 | SmDREB A6-16 | 335 | 37423.2 | 6.31 |
| EVM0033660 | SmDREB A5-21 | 214 | 23431.9 | 5.25 |
| EVM0057138 | SmDREB A2-4 | 207 | 23056.8 | 8.51 |
| EVM0045295 | SmDREB A2-18 | 286 | 32537.3 | 6.35 |
| EVM0000045 | SmERF B4-19 | 451 | 48363.6 | 5.99 |
| EVM0005349 | SmERF B3-21 | 183 | 20067.2 | 10.19 |
| EVM0031305 | SmERF B3-8 | 218 | 24208.7 | 5.15 |
| EVM0017641 | SmERF B3-7 | 227 | 25407 | 6.52 |
| EVM0057308 | SmERF B3-6 | 177 | 19430.4 | 5.73 |
| EVM0007023 | SmDREB A4-6 | 258 | 28580.2 | 5.23 |
| EVM0000144 | SmAP2-10 | 547 | 60291.8 | 6.57 |
| EVM0056353 | SmDREB A4-43 | 190 | 20701.2 | 4.6 |
| EVM0032428 | SmDREB A4-44 | 178 | 19723.8 | 6.53 |
| EVM0001082 | SmDREB A5-26 | 176 | 19202.3 | 9.11 |
| EVM0006666 | SmDREB A4-45 | 190 | 20723 | 8.83 |
| EVM0023183 | SmDREB A6-15 | 466 | 51531.4 | 7.91 |
| EVM0009817 | SmERF B6-13 | 301 | 32572.3 | 10.24 |
| EVM0024412 | SmERF B3-29 | 321 | 35141.5 | 8.85 |
| EVM0015672 | SmDREB A4-16 | 255 | 28295.9 | 6.24 |
| EVM0013811 | SmERF B3-30 | 334 | 36978.2 | 7.09 |
| EVM0004062 | SmAP2-47 | 722 | 79043.6 | 6.33 |
| EVM0051961 | SmERF B6-10 | 400 | 44665.8 | 4.58 |
| EVM0055116 | SmDREB A5-22 | 241 | 26009 | 5.28 |
| EVM0037206 | SmDREB A2-23 | 205 | 22829.5 | 8.49 |
| EVM0047653 | SmDREB A2-22 | 241 | 26469.4 | 6.27 |
| EVM0052780 | SmAP2-1 | 488 | 53223.3 | 8.27 |
| EVM0010374 | SmDREB A4-28 | 274 | 29164.6 | 4.69 |
| EVM0032370 | SmDREB A1-7 | 255 | 27700.8 | 5.76 |
| EVM0016530 | SmERF B1-25 | 188 | 20942.5 | 11.5 |
| EVM0003611 | SmRAV-4 | 367 | 41264 | 9.48 |
| EVM0042153 | SmERF B6-16 | 316 | 35182.8 | 4.49 |
| EVM0056730 | SmAP2-7 | 526 | 58215.7 | 6.46 |
| EVM0001221 | SmAP2-48 | 274 | 31442.8 | 10.03 |
| EVM0031724 | SmERF B4-16 | 244 | 27043.5 | 6.8 |
| EVM0021255 | SmDREB A4-26 | 206 | 21856 | 4.61 |
| EVM0040570 | SmAP2-21 | 505 | 55291.2 | 6.84 |
| EVM0000771 | SmDREB A4-32 | 206 | 21841.2 | 4.81 |
| EVM0020349 | SmDREB A5-10 | 177 | 19805.6 | 8.49 |
| EVM0010409 | SmAP2-19 | 528 | 58714.1 | 6.96 |
| EVM0023642 | SmDREB A5-1 | 164 | 18410.2 | 8.67 |
| EVM0043533 | SmAP2-2 | 637 | 69997.9 | 7.21 |
| EVM0003138 | SmAP2-55 | 497 | 54846.3 | 9.45 |
| EVM0041453 | SmERF B3-56 | 226 | 25690.2 | 5.16 |
| EVM0043216 | SmRAV-2 | 363 | 40996.8 | 9.38 |
| EVM0033580 | SmAP2-23 | 551 | 59798 | 7.72 |
| EVM0011412 | SmAP2-36 | 495 | 55014.4 | 9.25 |
| EVM0047762 | SmAP2-35 | 508 | 56355.1 | 8.69 |
| EVM0004638 | SmDREB A1-2 | 223 | 25032.7 | 7.81 |
| EVM0057013 | SmDREB A4-10 | 177 | 18888.7 | 4.65 |
| EVM0052812 | SmERF B6-12 | 332 | 35558.3 | 6.31 |
| EVM0054684 | SmERF B2-3 | 311 | 35277.9 | 5.51 |
| EVM0030954 | SmERF B4-14 | 479 | 51940.9 | 9.46 |
| EVM0004204 | SmERF B1-5 | 178 | 19221.6 | 10.17 |
| EVM0049898 | SmERF B1-6 | 231 | 25075.8 | 9.71 |
| EVM0012041 | SmERF B3-5 | 180 | 19864.8 | 8.49 |
| EVM0028403 | SmERF B2-2 | 274 | 30681.9 | 5.29 |
| EVM0029476 | SmERF B2-13 | 230 | 25414 | 5.04 |
| EVM0035055 | SmERF B3-51 | 182 | 20037.2 | 10.37 |
| EVM0048064 | SmERF B1-30 | 238 | 25952.6 | 7.56 |
| EVM0001371 | SmERF B1-31 | 176 | 18999.2 | 9.95 |
| EVM0034803 | SmDREB A2-14 | 182 | 20505.9 | 8.15 |
| EVM0040725 | SmERF B4-11 | 235 | 26227.6 | 5.05 |
| EVM0032830 | SmERF B1-36 | 180 | 20216.8 | 9.73 |
| EVM0035539 | SmAP2-40 | 432 | 47763.7 | 8.97 |
| EVM0055941 | SmERF B2-4 | 394 | 43582.9 | 4.66 |
| EVM0012457 | SmERF B1-7 | 270 | 29982.5 | 4.82 |
| EVM0018299 | SmDREB A4-9 | 207 | 23066.4 | 5.37 |
| EVM0009409 | SmERF B3-57 | 130 | 14672.1 | 6.53 |
| EVM0038648 | SmERF B3-58 | 221 | 24817.2 | 4.84 |
| EVM0021312 | SmERF B6-7 | 268 | 30226.2 | 5.41 |
| EVM0049397 | SmERF B1-23 | 175 | 18851.2 | 10.17 |
| EVM0052258 | SmERF B1-24 | 230 | 24997.8 | 9.76 |
| EVM0053591 | SmDREB A5-16 | 155 | 17414.3 | 9.96 |
| EVM0057697 | SmAP2-11 | 517 | 57915.7 | 8.22 |
| EVM0026318 | SmDREB A5-9 | 175 | 19637.5 | 9.99 |
| EVM0046939 | SmERF B1-37 | 208 | 22687 | 8.67 |
| EVM0030767 | SmRAV-1 | 369 | 43136.3 | 9.96 |
| EVM0049295 | SmDREB A5-13 | 151 | 16747.6 | 9.48 |
| EVM0035105 | SmDREB A5-14 | 256 | 28514.2 | 4.57 |
| EVM0000418 | SmAP2-24 | 458 | 50324.2 | 6.52 |
| EVM0045883 | SmERF B2-10 | 276 | 30927.2 | 4.76 |
| EVM0037464 | SmAP2-32 | 298 | 33501.9 | 6.18 |
| EVM0043933 | SmAP2-33 | 541 | 58890 | 7.32 |
| EVM0018696 | SmDREB A5-8 | 175 | 20156.9 | 10.4 |
| EVM0027097 | SmAP2-29 | 231 | 26561.2 | 4.39 |
| EVM0015330 | SmERF B5-15 | 319 | 35664.6 | 5.66 |
| EVM0009348 | SmDREB A2-21 | 275 | 31746.3 | 5.23 |
| EVM0036226 | SmSol-2 | 232 | 26107.2 | 10.42 |
| EVM0052605 | SmERF B2-14 | 276 | 31014.3 | 4.92 |
| EVM0028845 | SmDREB A2-19 | 180 | 20127.5 | 9.65 |
| EVM0005032 | SmDREB A4-29 | 223 | 24532.1 | 4.72 |
| EVM0055695 | SmERF B3-12 | 233 | 25973.7 | 4.76 |
| EVM0049755 | SmERF B3-13 | 285 | 31347.8 | 5.83 |
| EVM0006603 | SmERF B3-14 | 266 | 29260.8 | 8.23 |
| EVM0024690 | SmDREB A5-6 | 207 | 22802.4 | 5.79 |
| EVM0050816 | SmERF B6-15 | 378 | 42777.8 | 4.53 |
| EVM0025018 | SmDREB A6-14 | 449 | 50034.8 | 5.95 |
| EVM0001609 | SmERF B5-8 | 278 | 31239.7 | 9.91 |
| EVM0008900 | SmERF B1-38 | 195 | 21445.7 | 9.76 |
| EVM0045225 | SmERF B3-25 | 225 | 25627.1 | 4.88 |
| EVM0029866 | SmERF B1-12 | 209 | 23182.8 | 9.91 |
| EVM0041629 | SmERF B1-29 | 219 | 23972.4 | 10.17 |
| EVM0050911 | SmAP2-39 | 547 | 60318.8 | 6.52 |
| EVM0043812 | SmRAV-3 | 378 | 43903.8 | 9.52 |
| EVM0015843 | SmERF B4-10 | 207 | 22358.3 | 6.92 |
| EVM0052615 | SmAP2-22 | 529 | 58767.2 | 7.04 |
| EVM0050804 | SmDREB A5-11 | 176 | 19725.5 | 7.77 |
| EVM0017086 | SmERF B3-22 | 212 | 23933.4 | 6.09 |
| EVM0040715 | SmERF B3-23 | 148 | 16895.4 | 5.89 |
| EVM0029622 | SmERF B3-24 | 138 | 15455 | 8.44 |
| EVM0021270 | SmDREB A6-1 | 429 | 47283.6 | 9.08 |
| EVM0004107 | SmDREB A2-3 | 178 | 20185.7 | 9.22 |
| EVM0008274 | SmAP2-41 | 294 | 32938 | 9.96 |
| EVM0049140 | SmERF B1-39 | 116 | 12888.5 | 11.81 |
| EVM0002622 | SmERF B6-6 | 351 | 39077.3 | 4.8 |
| EVM0024408 | SmERF B6-11 | 270 | 30048.1 | 6.17 |
| EVM0009993 | SmAP2-5 | 669 | 73493 | 7.28 |
| EVM0006689 | SmAP2-14 | 710 | 77678.3 | 6.44 |
| EVM0018103 | SmDREB A2-9 | 329 | 35936.2 | 4.98 |
| EVM0025370 | SmDREB A3-1 | 333 | 35695.6 | 6.87 |
| EVM0036052 | SmAP2-15 | 485 | 53139.2 | 7.93 |
| EVM0011459 | SmERF B4-13 | 266 | 29603.7 | 4.81 |
| EVM0028773 | SmDREB A6-13 | 467 | 51331.1 | 6.64 |

**Table S3 AP2/ERF family members and classification of five plant species, which included the model plant Arabidopsis, Populus, and two Salix plants**

| Plant | *A. thaliana* | | *P. trichocarpa* | | *S. matsudana* | | *S.purpurea* | | *S. arbutifolia* | |
| --- | --- | --- | --- | --- | --- | --- | --- | --- | --- | --- |
| Classification | **No.** | **%** | **No.** | **%** | **No.** | **%** | **No.** | **%** | **No.** | **%** |
| DREB | **56** | **38.6** | **77** | **38.5** | **135** | **37.1** | **77** | **40.7** | **57** | **33.0** |
| DREB-A1 | 6 | 4.1 | 6 | 3.0 | 10 | 2.7 | 10 | 5.3 | 4 | 2.3 |
| DREB-A2 | 8 | 5.5 | 18 | 9.0 | 31 | 8.5 | 16 | 8.4 | 8 | 4.6 |
| DREB-A3 | 1 | 0.7 | 2 | 0.1 | 3 | 0.8 | 2 | 1.05 | 2 | 1.2 |
| DREB-A4 | 16 | 11.0 | 26 | 13.0 | 45 | 12.4 | 26 | 13.7 | 19 | 11.0 |
| DREB-A5 | 16 | 11.0 | 14 | 7.0 | 26 | 7.1 | 13 | 6.9 | 15 | 8.6 |
| DREB-A6 | 9 | 6.2 | 11 | 5.5 | 20 | 5.5 | 10 | 5.3 | 9 | 5.2 |
| ERF | **65** | **44.8** | **91** | **45.5** | **166** | **45.6** | **85** | **45** | **88** | **50.8** |
| ERF-B1 | 15 | 10.3 | 19 | 9.5 | 39 | 10.7 | 17 | 9.0 | 19 | 11.0 |
| ERF-B2 | 5 | 3.4 | 6 | 3.0 | 13 | 3.8 | 15 | 7.9 | 7 | 4.05 |
| ERF-B3 | 18 | 12.4 | 35 | 17.5 | 59 | 15.9 | 21 | 11.1 | 32 | 18.5 |
| ERF-B4 | 7 | 4.8 | 7 | 3.5 | **19** | 5.2 | 10 | 5.3 | 6 | 3.5 |
| ERF-B5 | 8 | 5.5 | 8 | 4.0 | 15 | 4.1 | 9 | 4.76 | 8 | 4.6 |
| ERF-B6 | 12 | 8.3 | 16 | 8.0 | **21** | 5.7 | 13 | 6.8 | 16 | 9.2 |
| RAV | **6** | **4.1** | **5** | **2.5** | **6** | **1.6** | **4** | **2.1** | **4** | **2.3** |
| AP2 | 17 | 11.7 | 26 | 13.0 | 55 | 15.1 | 22 | 11.6 | 22 | 12.7 |
| Solosist | **1** | **0.7** | **1** | **0.5** | **2** | **0.55** | **1** | **0.53** | **1** | **0.57** |
| 总量 | 145 |  | 200 |  | 364 |  | 189 |  | 173 |  |

**Table S4 *P. trichocarpa* and *S. purpurea* AP2/ERF family gene list**

| PtAP2/ERF family gene list | SpAP2/ERF family gene list |
| --- | --- |
| Potri.014G008100 | Sapur.001G031600 |
| Potri.010G092800 | Sapur.010G199800 |
| Potri.001G041500 | Sapur.014G004000 |
| Potri.002G114800 | Sapur.007G005500 |
| Potri.014G012200 | Sapur.006G137200 |
| Potri.006G167700 | Sapur.005G119800 |
| Potri.018G091600 | Sapur.018G067800 |
| Potri.007G011600 | Sapur.003G157900 |
| Potri.010G247200 | Sapur.001G015500 |
| Potri.003G185300 | Sapur.010G140700 |
| Potri.003G064700 | Sapur.008G061100 |
| Potri.003G205700 | Sapur.008G006800 |
| Potri.010G181000 | Sapur.007G043500 |
| Potri.005G148400 | Sapur.008G034200 |
| Potri.007G007400 | Sapur.002G092900 |
| Potri.001G018400 | Sapur.005G108500 |
| Potri.008G076400 | Sapur.006G109100 |
| Potri.018G102200 | Sapur.010G040000 |
| Potri.017G078600 | Sapur.002G147200 |
| Potri.001G169500 | Sapur.016G080700 |
| Potri.005G140700 | Sapur.008G134600 |
| Potri.007G046200 | Sapur.002G030300 |
| Potri.016G084500 | Sapur.004G035000 |
| Potri.008G045300 | Sapur.014G058500 |
| Potri.006G179900 | Sapur.010G040100 |
| Potri.006G132400 | Sapur.010G040200 |
| Potri.008G011900 | Sapur.010G005200 |
| Potri.010G216200 | Sapur.001G127900 |
| Potri.010G072400 | Sapur.007G082500 |
| Potri.014G076701 | Sapur.002G075900 |
| Potri.008G166000 | Sapur.001G064200 |
| Potri.004G051700 | Sapur.005G060200 |
| Potri.007G076800 | Sapur.003G111600 |
| Potri.004G051800 | Sapur.006G114500 |
| Potri.005G077300 | Sapur.005G068900 |
| Potri.008G210900 | Sapur.014G073200 |
| Potri.007G090600 | Sapur.017G007100 |
| Potri.001G079800 | Sapur.003G111500 |
| Potri.003G150800 | Sapur.005G130300 |
| Potri.002G094200 | Sapur.007G073000 |
| Potri.003G080600 | Sapur.002G051800 |
| Potri.009G101900 | Sapur.011G039300 |
| Potri.018G038100 | Sapur.010G143500 |
| Potri.002G029400 | Sapur.014G035700 |
| Potri.005G087200 | Sapur.016G190500 |
| Potri.012G134100 | Sapur.017G072300 |
| Potri.005G168700 | Sapur.011G042400 |
| Potri.010G072600 | Sapur.013G041800 |
| Potri.011G061700 | Sapur.002G030100 |
| Potri.011G057000 | Sapur.008G160400 |
| Potri.014G094500 | Sapur.009G079700 |
| Potri.010G006800 | Sapur.008G134800 |
| Potri.014G046600 | Sapur.005G186300 |
| Potri.017G013700 | Sapur.003G140600 |
| Potri.011G115600 | Sapur.012G082100 |
| Potri.013G045200 | Sapur.006G130000 |
| Potri.019G015500 | Sapur.017G042000 |
| Potri.014G047000 | Sapur.001G064300 |
| Potri.001G313500 | Sapur.003G049100 |
| Potri.017G087800 | Sapur.15WG018100 |
| Potri.001G397200 | Sapur.15ZG017800 |
| Potri.011G061800 | Sapur.019G025000 |
| Potri.004G047601 | Sapur.001G127700 |
| Potri.008G166100 | Sapur.011G079300 |
| Potri.002G039100 | Sapur.15WG143900 |
| Potri.002G153500 | Sapur.15ZG126100 |
| Potri.006G138900 | Sapur.004G031700 |
| Potri.017G053700 | Sapur.003G049200 |
| Potri.012G108500 | Sapur.006G197300 |
| Potri.003G050700 | Sapur.018G031200 |
| Potri.008G166200 | Sapur.016G017900 |
| Potri.003G081200 | Sapur.013G096100 |
| Potri.002G039300 | Sapur.006G042000 |
| Potri.001G079900 | Sapur.005G177700 |
| Potri.004G141200 | Sapur.001G075700 |
| Potri.005G233300 | Sapur.001G003600 |
| Potri.001G154100 | Sapur.013G141800 |
| Potri.002G167400 | Sapur.016G255700 |
| Potri.014G046900 | Sapur.004G031600 |
| Potri.001G154200 | Sapur.001G052600 |
| Potri.015G023200 | Sapur.007G123400 |
| Potri.015G136400 | Sapur.012G020100 |
| Potri.013G101200 | Sapur.014G042400 |
| Potri.003G150700 | Sapur.016G047500 |
| Potri.013G158500 | Sapur.017G043500 |
| Potri.006G054500 | Sapur.018G034800 |
| Potri.010G072300 | Sapur.004G035100 |
| Potri.005G195000 | Sapur.005G177600 |
| Potri.006G238600 | Sapur.005G153300 |
| Potri.008G091300 | Sapur.019G035300 |
| Potri.002G065600 | Sapur.005G177800 |
| Potri.001G356100 | Sapur.003G026800 |
| Potri.001G092400 | Sapur.013G052600 |
| Potri.011G056900 | Sapur.003G102600 |
| Potri.010G183700 | Sapur.001G064100 |
| Potri.004G047500 | Sapur.002G030200 |
| Potri.001G004700 | Sapur.012G103100 |
| Potri.014G055700 | Sapur.006G037700 |
| Potri.016G053200 | Sapur.003G120300 |
| Potri.012G032900 | Sapur.019G073100 |
| Potri.005G223200 | Sapur.003G017600 |
| Potri.009G147700 | Sapur.002G115400 |
| Potri.005G219600 | Sapur.016G227700 |
| Potri.018G043900 | Sapur.006G179000 |
| Potri.019G075600 | Sapur.006G056000 |
| Potri.007G138100 | Sapur.002G143500 |
| Potri.019G036100 | Sapur.016G050300 |
| Potri.014G046700 | Sapur.019G098000 |
| Potri.001G187500 | Sapur.009G116700 |
| Potri.005G223100 | Sapur.013G126800 |
| Potri.002G039200 | Sapur.003G099900 |
| Potri.010G163900 | Sapur.013G096000 |
| Potri.001G079600 | Sapur.016G017800 |
| Potri.003G033000 | Sapur.016G191900 |
| Potri.003G220200 | Sapur.018G036500 |
| Potri.001G067600 | Sapur.016G108900 |
| Potri.005G223300 | Sapur.001G090500 |
| Potri.003G151000 | Sapur.001G130000 |
| Potri.016G056400 | Sapur.019G073000 |
| Potri.006G049700 | Sapur.010G096300 |
| Potri.003G162500 | Sapur.003G166800 |
| Potri.008G073600 | Sapur.018G102800 |
| Potri.003G139300 | Sapur.014G101000 |
| Potri.001G110800 | Sapur.001G140100 |
| Potri.001G155700 | Sapur.001G054400 |
| Potri.002G141200 | Sapur.014G035600 |
| Potri.001G094800 | Sapur.003G111700 |
| Potri.002G043300 | Sapur.004G089000 |
| Potri.018G131400 | Sapur.008G058500 |
| Potri.006G069400 | Sapur.001G077800 |
| Potri.019G075500 | Sapur.001G163400 |
| Potri.003G136300 | Sapur.010G146100 |
| Potri.013G135600 | Sapur.003G119100 |
| Potri.017G055400 | Sapur.002G159300 |
| Potri.013G056700 | Sapur.014G078300 |
| Potri.001G453100 | Sapur.008G150000 |
| Potri.006G218200 | Sapur.001G134100 |
| Potri.014G099900 | Sapur.003G041000 |
| Potri.003G079300 | Sapur.018G062500 |
| Potri.001G069300 | Sapur.003G136500 |
| Potri.001G157100 | Sapur.019G112800 |
| Potri.019G131300 | Sapur.018G018300 |
| Potri.003G077700 | Sapur.001G090400 |
| Potri.014G126100 | Sapur.007G040500 |
| Potri.001G315300 | Sapur.006G215800 |
| Potri.001G163700 | Sapur.005G137400 |
| Potri.010G125600 | Sapur.018G023800 |
| Potri.013G101100 | Sapur.002G068300 |
| Potri.016G126100 | Sapur.008G096700 |
| Potri.008G071100 | Sapur.001G038700 |
| Potri.002G201600 | Sapur.014G014900 |
| Potri.003G161000 | Sapur.008G056300 |
| Potri.006G156600 | Sapur.006G114400 |
| Potri.018G085700 | Sapur.003G046200 |
| Potri.007G043400 | Sapur.002G030000 |
| Potri.006G163400 | Sapur.007G009300 |
| Potri.010G186400 | Sapur.008G165200 |
| Potri.002G172200 | Sapur.003G047300 |
| Potri.002G172300 | Sapur.002G100900 |
| Potri.002G172600 | Sapur.007G043700 |
| Potri.014G046800 | Sapur.006G209800 |
| Potri.008G186300 | Sapur.019G067700 |
| Potri.003G179900 | Sapur.010G025900 |
| Potri.004G187001 | Sapur.010G025800 |
| Potri.001G048200 | Sapur.010G026100 |
| Potri.002G085600 | Sapur.012G103000 |
| Potri.006G261200 | Sapur.003G085700 |
| Potri.018G021900 | Sapur.005G108300 |
| Potri.006G138700 | Sapur.010G023900 |
| Potri.001G181500 | Sapur.006G085100 |
| Potri.019G102200 | Sapur.013G109000 |
| Potri.018G028000 | Sapur.010G171900 |
| Potri.018G047300 | Sapur.15WG143800 |
| Potri.005G176000 | Sapur.15ZG126000 |
| Potri.008G120100 | Sapur.016G015300 |
| Potri.006G253800 | Sapur.001G152400 |
| Potri.014G025200 | Sapur.013G094600 |
| Potri.002G124000 | Sapur.019G084600 |
| Potri.001G110700 | Sapur.010G099700 |
| Potri.012G134000 | Sapur.003G085800 |
| Potri.006G104200 | Sapur.008G094300 |
| Potri.006G080300 | Sapur.006G015800 |
| Potri.006G138800 | Sapur.001G090300 |
| Potri.008G215600 | Sapur.019G071100 |
| Potri.007G046500 | Sapur.008G134700 |
| Potri.005G140900 | Sapur.003G161900 |
| Potri.002G039000 | Sapur.006G152200 |
| Potri.019G067400 | Sapur.15ZG054600 |
| Potri.010G046600 | Sapur.15WG058700 |
| Potri.003G054100 |  |
| Potri.003G054400 |  |
| Potri.015G136300 |  |
| Potri.013G100300 |  |
| Potri.008G117100 |  |
| Potri.019G088000 |  |
| Potri.010G129200 |  |
| Potri.006G021000 |  |
| Potri.011G148900 |  |
| Potri.016G018600 |  |
| Potri.003G121200 |  |
| Potri.001G110500 |  |
| Potri.019G073300 |  |
| Potri.015G054500 |  |
| Potri.003G212800 |  |
| Potri.002G246100 |  |
| Potri.018G109201 |  |
| Potri.006G186301 |  |

**Table S5 198 duplicated segments containing *SmAP2/ERF* genes located on 38 chromosomes in *S. matsudana***

| Chromosome  Name | Gene start point | Gene end point | Chromosome  Name | | Gene start point | Gene end point |
| --- | --- | --- | --- | --- | --- | --- |
| Chr2 | 2362316 | 2363385 | | Chr24 | 10084075 | 10085239 |
| Chr5 | 8204025 | 8204798 | | Chr8 | 5721970 | 5723025 |
| Chr5 | 9605140 | 9605634 | | Chr8 | 7687061 | 7688921 |
| Chr5 | 10270878 | 10274101 | | Chr8 | 8581283 | 8584685 |
| Chr5 | 12739677 | 12740523 | | Chr9 | 6429531 | 6430183 |
| Chr10 | 142637 | 143493 | | Chr21 | 310659 | 311522 |
| Chr10 | 2230958 | 2234699 | | Chr11 | 10583659 | 10588186 |
| Chr10 | 2570973 | 2571891 | | Chr11 | 10315688 | 10316632 |
| Chr10 | 3773942 | 3777168 | | Chr11 | 9037440 | 9040917 |
| Chr10 | 4495919 | 4497000 | | Chr11 | 8377781 | 8378971 |
| Chr10 | 4499378 | 4500735 | | Chr11 | 8374018 | 8374878 |
| Chr10 | 4509343 | 4510493 | | Chr11 | 8367863 | 8368841 |
| Chr10 | 5305235 | 5306874 | | Chr11 | 7709988 | 7710922 |
| Chr10 | 5458147 | 5460186 | | Chr11 | 7547197 | 7548260 |
| Chr10 | 8938845 | 8939604 | | Chr21 | 15194295 | 15194840 |
| Chr10 | 9753979 | 9754788 | | Chr21 | 16056458 | 16057619 |
| Chr11 | 3328427 | 3329239 | | Chr21 | 13945144 | 13945965 |
| Chr11 | 3398800 | 3399563 | | Chr31 | 4888379 | 4889041 |
| Chr11 | 6553430 | 6554515 | | Chr21 | 8505611 | 8506620 |
| Chr11 | 6559660 | 6560193 | | Chr21 | 8489401 | 8489933 |
| Chr11 | 7547197 | 7548260 | | Chr5 | 5701082 | 5702192 |
| Chr11 | 8367863 | 8368841 | | Chr21 | 6721016 | 6722211 |
| Chr11 | 8374018 | 8374878 | | Chr21 | 6710324 | 6711766 |
| Chr11 | 8377781 | 8378971 | | Chr21 | 6707199 | 6708550 |
| Chr11 | 8955415 | 8956290 | | Chr21 | 6042701 | 6043564 |
| Chr11 | 9037440 | 9040917 | | Chr21 | 5918111 | 5920667 |
| Chr11 | 10583659 | 10588186 | | Chr21 | 3808051 | 3811251 |
| Chr11 | 10315688 | 10316632 | | Chr21 | 4398100 | 4398870 |
| Chr11 | 11403695 | 11406235 | | Chr21 | 1073084 | 1075613 |
| Chr11 | 12059486 | 12060352 | | Chr21 | 310659 | 311522 |
| Chr12 | 457997 | 458968 | | Chr35 | 605480 | 606315 |
| Chr12 | 443665 | 444411 | | Chr35 | 589680 | 591054 |
| Chr13 | 739809 | 741193 | | Chr33 | 4264719 | 4265444 |
| Chr13 | 2007451 | 2007876 | | Chr29 | 14505240 | 14507365 |
| Chr13 | 2024597 | 2025462 | | Chr29 | 14516627 | 14517978 |
| Chr13 | 2032457 | 2033173 | | Chr29 | 14522233 | 14523230 |
| Chr13 | 4033375 | 4035706 | | Chr5 | 14089844 | 14092768 |
| Chr13 | 5743541 | 5746895 | | Chr9 | 9477130 | 9480646 |
| Chr13 | 10429013 | 10431191 | | Chr16 | 10631142 | 10633208 |
| Chr14 | 3057651 | 3058448 | | Chr7 | 2891931 | 2892743 |
| Chr15 | 411193 | 413308 | | Chr37 | 341047 | 342700 |
| Chr15 | 2138633 | 2139883 | | Chr23 | 9892329 | 9893575 |
| Chr15 | 9573131 | 9574871 | | Chr37 | 5861830 | 5863276 |
| Chr15 | 12743655 | 12748081 | | Chr23 | 4087398 | 4091540 |
| Chr15 | 12903051 | 12905365 | | Chr23 | 3938968 | 3941095 |
| Chr15 | 13110655 | 13111656 | | Chr23 | 3754012 | 3755234 |
| Chr15 | 15085188 | 15088522 | | Chr23 | 2245015 | 2249414 |
| Chr15 | 16472842 | 16476860 | | Chr23 | 539694 | 543463 |
| Chr16 | 658886 | 662784 | | Chr17 | 182540 | 184987 |
| Chr16 | 1022970 | 1027766 | | Chr17 | 626949 | 631152 |
| Chr16 | 4121167 | 4121808 | | Chr17 | 3236607 | 3237230 |
| Chr16 | 9758085 | 9760244 | | Chr16 | 10631142 | 10633208 |
| Chr16 | 10631142 | 10633208 | | Chr9 | 15379142 | 15381161 |
| Chr16 | 13773660 | 13774784 | | Chr22 | 600284 | 601330 |
| Chr18 | 433778 | 434563 | | Chr33 | 20486591 | 20487788 |
| Chr18 | 2397889 | 2398965 | | Chr33 | 17224656 | 17225832 |
| Chr18 | 6392121 | 6396157 | | Chr33 | 13218508 | 13222214 |
| Chr18 | 8875486 | 8876182 | | Chr33 | 10835596 | 10836049 |
| Chr18 | 8882935 | 8883896 | | Chr33 | 10828110 | 10829035 |
| Chr18 | 9509193 | 9512777 | | Chr28 | 22197852 | 22201400 |
| Chr18 | 14012913 | 14014712 | | Chr33 | 9149844 | 9151385 |
| Chr18 | 17554679 | 17555538 | | Chr28 | 25661280 | 25662140 |
| Chr18 | 17929466 | 17932570 | | Chr28 | 25325661 | 25331688 |
| Chr19 | 174699 | 176819 | | Chr2 | 8888949 | 8891400 |
| Chr19 | 1191580 | 1194287 | | Chr25 | 9079918 | 9082495 |
| Chr19 | 3856086 | 3856653 | | Chr25 | 7322672 | 7323241 |
| Chr19 | 3870692 | 3871769 | | Chr25 | 7303215 | 7304284 |
| Chr19 | 4298214 | 4298726 | | Chr24 | 4052839 | 4053342 |
| Chr19 | 9519434 | 9521054 | | Chr24 | 10084075 | 10085239 |
| Chr19 | 9892779 | 9893664 | | Chr24 | 11791058 | 11791735 |
| Chr20 | 5203559 | 5204755 | | Chr29 | 3521521 | 3523072 |
| Chr20 | 6613376 | 6613969 | | Chr5 | 5237572 | 5238301 |
| Chr20 | 8123104 | 8125164 | | Chr29 | 8345150 | 8345970 |
| Chr20 | 8991547 | 8994882 | | Chr8 | 8581283 | 8584685 |
| Chr21 | 310659 | 311522 | | Chr31 | 13216501 | 13217380 |
| Chr21 | 1073084 | 1075613 | | Chr31 | 12413286 | 12415841 |
| Chr21 | 4398100 | 4398870 | | Chr31 | 10954365 | 10955211 |
| Chr21 | 5534853 | 5537937 | | Chr21 | 5918111 | 5920667 |
| Chr21 | 8489401 | 8489933 | | Chr31 | 8381061 | 8381597 |
| Chr21 | 8505611 | 8506620 | | Chr31 | 8364519 | 8365508 |
| Chr21 | 12257100 | 12258303 | | Chr31 | 5063448 | 5064745 |
| Chr21 | 13945144 | 13945965 | | Chr31 | 4796986 | 4797801 |
| Chr23 | 539694 | 543463 | | Chr34 | 520782 | 524925 |
| Chr23 | 3754012 | 3755234 | | Chr37 | 8825502 | 8826497 |
| Chr23 | 3938968 | 3941095 | | Chr37 | 8638202 | 8640601 |
| Chr23 | 4087398 | 4091540 | | Chr34 | 2944484 | 2948720 |
| Chr23 | 8656089 | 8656957 | | Chr37 | 2770586 | 2771217 |
| Chr23 | 8644898 | 8645344 | | Chr34 | 9044031 | 9044477 |
| Chr23 | 8641542 | 8642442 | | Chr37 | 2751487 | 2751903 |
| Chr23 | 9892329 | 9893575 | | Chr34 | 9625056 | 9626315 |
| Chr23 | 11423164 | 11424177 | | Chr34 | 11207870 | 11208889 |
| Chr26 | 2473647 | 2477130 | | Chr33 | 13218508 | 13222214 |
| Chr26 | 3204521 | 3205504 | | Chr6 | 7147911 | 7148771 |
| Chr26 | 8566999 | 8568151 | | Chr6 | 2975044 | 2976279 |
| Chr26 | 9511257 | 9511720 | | Chr6 | 2279899 | 2280706 |
| Chr26 | 10350906 | 10352140 | | Chr33 | 20015505 | 20016966 |
| Chr26 | 10890056 | 10891336 | | Chr33 | 20486591 | 20487788 |
| Chr27 | 7554016 | 7554816 | | Chr3 | 3607753 | 3608777 |
| Chr27 | 9965829 | 9966985 | | Chr3 | 1517729 | 1518547 |
| Chr28 | 5355084 | 5356485 | | Chr4 | 4338260 | 4338879 |
| Chr28 | 8944712 | 8946622 | | Chr4 | 7496115 | 7497558 |
| Chr28 | 12365717 | 12367117 | | Chr4 | 10611904 | 10613280 |
| Chr28 | 12483666 | 12484571 | | Chr4 | 10727231 | 10728136 |
| Chr28 | 22197852 | 22201400 | | Chr4 | 21659902 | 21663629 |
| Chr28 | 25325661 | 25331688 | | Chr33 | 2419545 | 2422985 |
| Chr28 | 25661280 | 25662140 | | Chr33 | 2787827 | 2788686 |
| Chr28 | 27069261 | 27069881 | | Chr4 | 26705254 | 26705853 |
| Chr29 | 905257 | 913866 | | Chr5 | 1023767 | 1031562 |
| Chr29 | 3521521 | 3523072 | | Chr8 | 3209265 | 3210516 |
| Chr29 | 6850002 | 6851072 | | Chr5 | 8204025 | 8204798 |
| Chr29 | 8345150 | 8345970 | | Chr8 | 7687061 | 7688921 |
| Chr29 | 8917550 | 8920909 | | Chr8 | 8581283 | 8584685 |
| Chr29 | 11316289 | 11317057 | | Chr9 | 6429531 | 6430183 |
| Chr29 | 14299365 | 14299903 | | Chr5 | 14923850 | 14924391 |
| Chr29 | 14505240 | 14507365 | | Chr5 | 15195940 | 15197285 |
| Chr30 | 1493177 | 1494887 | | Chr36 | 1540873 | 1542882 |
| Chr30 | 8908621 | 8910693 | | Chr35 | 1968417 | 1970633 |
| Chr30 | 10503978 | 10504950 | | Chr35 | 605480 | 606315 |
| Chr30 | 10512127 | 10513428 | | Chr35 | 589680 | 591054 |
| Chr32 | 2330082 | 2330811 | | Chr7 | 2578613 | 2579537 |
| Chr32 | 2348282 | 2349475 | | Chr7 | 2596986 | 2598195 |
| Chr32 | 2628337 | 2629167 | | Chr7 | 2891931 | 2892743 |
| Chr33 | 4398690 | 4399445 | | Chr6 | 10018269 | 10019048 |
| Chr33 | 7864609 | 7865746 | | Chr5 | 8204025 | 8204798 |
| Chr33 | 8455067 | 8455860 | | Chr8 | 6207721 | 6208607 |
| Chr33 | 13218508 | 13222214 | | Chr6 | 7697739 | 7701199 |
| Chr33 | 18921092 | 18922496 | | Chr6 | 2618410 | 2619739 |
| Chr33 | 20015505 | 20016966 | | Chr6 | 1426854 | 1427871 |
| Chr34 | 2636513 | 2639751 | | Chr37 | 10713379 | 10716237 |
| Chr34 | 2944484 | 2948720 | | Chr37 | 8506928 | 8511166 |
| Chr34 | 4171318 | 4171977 | | Chr38 | 4079202 | 4080697 |
| Chr34 | 6012089 | 6014133 | | Chr37 | 5861830 | 5863276 |
| Chr34 | 9044031 | 9044477 | | Chr37 | 2756447 | 2756893 |
| Chr34 | 9625056 | 9626315 | | Chr37 | 1561185 | 1562435 |
| Chr38 | 7943983 | 7944784 | | Chr7 | 2894558 | 2895494 |
| Chr38 | 7952202 | 7952925 | | Chr7 | 2891931 | 2892743 |
| Chr38 | 8278287 | 8279809 | | Chr7 | 2596986 | 2598195 |
| Chr38 | 8311673 | 8312242 | | Chr7 | 2578613 | 2579537 |
| Chr5 | 3541395 | 3542436 | | Chr20 | 5203559 | 5204755 |
| Chr5 | 9605140 | 9605634 | | Chr20 | 8123104 | 8125164 |
| Chr5 | 10270878 | 10274101 | | Chr29 | 8917550 | 8920909 |
| Chr5 | 12090916 | 12093367 | | Chr29 | 10738774 | 10741189 |
| Chr5 | 12739677 | 12740523 | | Chr29 | 11316289 | 11317057 |
| Chr5 | 15195940 | 15197285 | | Chr13 | 2007451 | 2007876 |
| Chr5 | 15199198 | 15199626 | | Chr29 | 14508793 | 14509221 |
| Chr5 | 15208533 | 15209884 | | Chr13 | 2024597 | 2025462 |
| Chr5 | 15213977 | 15214872 | | Chr13 | 2032457 | 2033173 |
| Chr5 | 15810524 | 15811083 | | Chr13 | 739809 | 741193 |
| Chr6 | 1426854 | 1427871 | | Chr26 | 10350906 | 10352140 |
| Chr6 | 7697739 | 7701199 | | Chr18 | 6392121 | 6396157 |
| Chr6 | 10018269 | 10019048 | | Chr18 | 16371131 | 16371906 |
| Chr8 | 3209265 | 3210516 | | Chr20 | 5203559 | 5204755 |
| Chr8 | 7687061 | 7688921 | | Chr20 | 8123104 | 8125164 |
| Chr9 | 15379142 | 15381161 | | Chr13 | 10429013 | 10431191 |
| Chr11 | 8955415 | 8956290 | | Chr10 | 3900981 | 3901559 |
| Chr16 | 9758085 | 9760244 | | Chr13 | 10429013 | 10431191 |
| Chr17 | 11077210 | 11078241 | | Chr16 | 13773660 | 13774784 |
| Chr21 | 3808051 | 3811251 | | Chr10 | 2230958 | 2234699 |
| Chr21 | 4398100 | 4398870 | | Chr10 | 2570973 | 2571891 |
| Chr21 | 5534853 | 5537937 | | Chr10 | 3773942 | 3777168 |
| Chr21 | 6721016 | 6722211 | | Chr10 | 4509343 | 4510493 |
| Chr21 | 6707199 | 6708550 | | Chr10 | 4495919 | 4497000 |
| Chr21 | 6042701 | 6043564 | | Chr10 | 3900981 | 3901559 |
| Chr21 | 5918111 | 5920667 | | Chr10 | 3773942 | 3777168 |
| Chr21 | 8106404 | 8108067 | | Chr10 | 5305235 | 5306874 |
| Chr24 | 757528 | 760261 | | Chr19 | 1191580 | 1194287 |
| Chr24 | 3781989 | 3782549 | | Chr19 | 3856086 | 3856653 |
| Chr26 | 2473647 | 2477130 | | Chr18 | 6392121 | 6396157 |
| Chr30 | 10503978 | 10504950 | | Chr12 | 457997 | 458968 |
| Chr30 | 10512127 | 10513428 | | Chr12 | 443665 | 444411 |
| Chr31 | 4796986 | 4797801 | | Chr11 | 3328427 | 3329239 |
| Chr31 | 8364519 | 8365508 | | Chr11 | 6553430 | 6554515 |
| Chr31 | 8381061 | 8381597 | | Chr11 | 6559660 | 6560193 |
| Chr31 | 10288327 | 10289356 | | Chr11 | 8377781 | 8378971 |
| Chr31 | 10291978 | 10292839 | | Chr11 | 8374018 | 8374878 |
| Chr31 | 10299294 | 10300395 | | Chr11 | 8367863 | 8368841 |
| Chr31 | 12413286 | 12415841 | | Chr11 | 11403695 | 11406235 |
| Chr31 | 13216501 | 13217380 | | Chr10 | 142637 | 143493 |
| Chr33 | 2419545 | 2422985 | | Chr18 | 17929466 | 17932570 |
| Chr33 | 2787827 | 2788686 | | Chr18 | 17554679 | 17555538 |
| Chr33 | 2998543 | 3002750 | | Chr18 | 17398849 | 17401106 |
| Chr33 | 4398690 | 4399445 | | Chr18 | 16371131 | 16371906 |
| Chr33 | 10255687 | 10259086 | | Chr18 | 9509193 | 9512777 |
| Chr34 | 520782 | 524925 | | Chr15 | 16472842 | 16476860 |
| Chr34 | 2636513 | 2639751 | | Chr15 | 15085188 | 15088522 |
| Chr34 | 2944484 | 2948720 | | Chr15 | 12743655 | 12748081 |
| Chr34 | 6012089 | 6014133 | | Chr15 | 9573131 | 9574871 |
| Chr34 | 6216765 | 6219169 | | Chr15 | 9179658 | 9181959 |
| Chr34 | 9625056 | 9626315 | | Chr15 | 2138633 | 2139883 |
| Chr37 | 1561185 | 1562435 | | Chr15 | 2138633 | 2139883 |
| Chr37 | 2756447 | 2756893 | | Chr23 | 8644898 | 8645344 |
| Chr37 | 8825502 | 8826497 | | Chr15 | 13110655 | 13111656 |
| Chr37 | 8638202 | 8640601 | | Chr15 | 12903051 | 12905365 |
| Chr37 | 8506928 | 8511166 | | Chr15 | 12743655 | 12748081 |
| Chr38 | 7943983 | 7944784 | | Chr32 | 2628337 | 2629167 |
| Chr38 | 7952202 | 7952925 | | Chr14 | 3057651 | 3058448 |
| Chr38 | 8278287 | 8279809 | | Chr32 | 2348282 | 2349475 |
| Chr38 | 8311673 | 8312242 | | Chr32 | 2330082 | 2330811 |

**Table S6 AP2_duplication gene Ka Ks and divergence time calculation**

| gene1 | gene2 | Ka | Ks | ω（Ka/Ks） | Effective  Length | T=Divergence  Time(Year) |
| --- | --- | --- | --- | --- | --- | --- |
| EVM0000005 | EVM0007023 | 0.0947 | 0.418817 | 0.226112 | 759 | 23011921 |
| EVM0000005 | EVM0055308 | 0.108804 | 0.425597 | 0.255651 | 771 | 23384449 |
| EVM0005644 | EVM0000005 | 0.034484 | 0.083295 | 0.413997 | 771 | 4576649 |
| EVM0005644 | EVM0055308 | 0.102386 | 0.436169 | 0.234738 | 777 | 23965342 |
| EVM0055308 | EVM0007023 | 0.011917 | 0.1343 | 0.088733 | 771 | 7379114 |
| EVM0005644 | EVM0007023 | 0.098352 | 0.403741 | 0.243603 | 762 | 22183556 |
| EVM0000006 | EVM0044977 | 0.166456 | 0.468377 | 0.355388 | 882 | 25735001 |
| EVM0044977 | EVM0009817 | 0.041286 | 0.137829 | 0.299546 | 903 | 7573040 |
| EVM0018988 | EVM0000006 | 0.038567 | 0.159174 | 0.242295 | 891 | 8745804 |
| EVM0000006 | EVM0009817 | 0.17235 | 0.511402 | 0.337014 | 882 | 28099022 |
| EVM0000144 | EVM0050911 | 0.007134 | 0.067193 | 0.10617 | 1641 | 3691947 |
| EVM0008451 | EVM0050911 | 0.000819 | 0 | #NAME? | 1584 | 0 |
| EVM0008451 | EVM0000144 | 0.007404 | 0.06933 | 0.106792 | 1584 | 3809344 |
| EVM0008451 | EVM0035153 | 0.045176 | 0.302626 | 0.149279 | 1575 | 16627818 |
| EVM0056730 | EVM0050911 | 0.055843 | 0.319826 | 0.174605 | 1575 | 17572857 |
| EVM0035153 | EVM0056730 | 0.028797 | 0.115738 | 0.248809 | 1578 | 6359258 |
| EVM0035153 | EVM0050911 | 0.043621 | 0.305708 | 0.142689 | 1626 | 16797133 |
| EVM0035153 | EVM0000144 | 0.044468 | 0.338892 | 0.131216 | 1626 | 18620450 |
| EVM0008451 | EVM0056730 | 0.058842 | 0.321329 | 0.183121 | 1524 | 17655414 |
| EVM0000144 | EVM0056730 | 0.056738 | 0.368015 | 0.154173 | 1575 | 20220630 |
| EVM0017689 | EVM0047653 | 0.042074 | 0.095893 | 0.438758 | 711 | 5268828 |
| EVM0000172 | EVM0047653 | 0.129615 | 0.480833 | 0.269564 | 537 | 26419385 |
| EVM0015466 | EVM0038704 | 0.108129 | 0.356461 | 0.303342 | 873 | 19585743 |
| EVM0038704 | EVM0014537 | 0.0468 | 0.121395 | 0.385521 | 873 | 6670049 |
| EVM0000460 | EVM0014537 | 0.090419 | 0.304538 | 0.296906 | 924 | 16732833 |
| EVM0015466 | EVM0000460 | 0.022867 | 0.047654 | 0.479857 | 927 | 2618350 |
| EVM0015466 | EVM0014537 | 0.085542 | 0.27854 | 0.307106 | 924 | 15304422 |
| EVM0000460 | EVM0038704 | 0.113499 | 0.3864 | 0.293736 | 873 | 21230753 |
| EVM0000535 | EVM0048064 | 0.044804 | 0.083323 | 0.537717 | 681 | 4578164 |
| EVM0008128 | EVM0040250 | 0.011528 | 0.061051 | 0.18882 | 678 | 3354431 |
| EVM0035905 | EVM0040250 | 0.083461 | 0.578188 | 0.14435 | 678 | 31768552 |
| EVM0035905 | EVM0008128 | 0.091988 | 0.593478 | 0.154998 | 678 | 32608702 |
| EVM0001248 | EVM0040250 | 0.095253 | 0.599746 | 0.158823 | 678 | 32953077 |
| EVM0035905 | EVM0001248 | 0.043659 | 0.131386 | 0.332298 | 684 | 7218997 |
| EVM0001248 | EVM0008128 | 0.103912 | 0.645934 | 0.160871 | 678 | 35490890 |
| EVM0001371 | EVM0049397 | 0.048981 | 0.23803 | 0.205776 | 525 | 13078547 |
| EVM0004204 | EVM0049397 | 0.015545 | 0.111611 | 0.139274 | 525 | 6132492 |
| EVM0004204 | EVM0001371 | 0.045926 | 0.257924 | 0.178061 | 528 | 14171621 |
| EVM0001533 | EVM0052698 | 0.049341 | 0.07756 | 0.636165 | 1353 | 4261529 |
| EVM0001548 | EVM0022064 | 0.018438 | 0.155899 | 0.11827 | 498 | 8565864 |
| EVM0001548 | EVM0001082 | 0.12062 | 0.561392 | 0.21486 | 507 | 30845693 |
| EVM0001696 | EVM0005032 | 0.014635 | 0.077171 | 0.189639 | 633 | 4240155 |
| EVM0021344 | EVM0018160 | 0.031956 | 0.17596 | 0.181608 | 690 | 9668138 |
| EVM0021344 | EVM0005032 | 0.109251 | 0.593728 | 0.184009 | 654 | 32622404 |
| EVM0018160 | EVM0005032 | 0.121367 | 0.521889 | 0.232553 | 654 | 28675223 |
| EVM0001696 | EVM0018160 | 0.125767 | 0.598047 | 0.210296 | 627 | 32859724 |
| EVM0002257 | EVM0013555 | 0.058392 | 0.138816 | 0.420645 | 423 | 7627226 |
| EVM0002257 | EVM0049295 | 0.187968 | 0.371145 | 0.506454 | 453 | 20392608 |
| EVM0002653 | EVM0002622 | 0.028835 | 0.099132 | 0.290872 | 1050 | 5446797 |
| EVM0043678 | EVM0011459 | 0.13306 | 0.614413 | 0.216565 | 795 | 33758937 |
| EVM0002769 | EVM0011459 | 0.03299 | 0.121542 | 0.271433 | 789 | 6678126 |
| EVM0036069 | EVM0040715 | 0.132016 | 0.337578 | 0.391069 | 444 | 18548246 |
| EVM0002921 | EVM0040715 | 0.045059 | 0.106184 | 0.424344 | 444 | 5834290 |
| EVM0012873 | EVM0036069 | 0.026696 | 0.153539 | 0.17387 | 444 | 8436234 |
| EVM0012873 | EVM0002921 | 0.133544 | 0.27249 | 0.490089 | 444 | 14971957 |
| EVM0012873 | EVM0040715 | 0.12841 | 0.293635 | 0.437311 | 444 | 16133790 |
| EVM0002921 | EVM0036069 | 0.140687 | 0.315321 | 0.446172 | 444 | 17325329 |
| EVM0002934 | EVM0036226 | 0.00748 | 0.038823 | 0.192676 | 696 | 2133114 |
| EVM0011072 | EVM0003379 | 0.131123 | 0.188637 | 0.695107 | 528 | 10364685 |
| EVM0011072 | EVM0054250 | 0.079856 | 0.518854 | 0.153908 | 540 | 28508481 |
| EVM0054250 | EVM0034803 | 0.016919 | 0.093939 | 0.180107 | 543 | 5161497 |
| EVM0054250 | EVM0003379 | 0.207979 | 0.631042 | 0.329581 | 528 | 34672641 |
| EVM0011072 | EVM0034803 | 0.084991 | 0.493419 | 0.172248 | 540 | 27110953 |
| EVM0003379 | EVM0034803 | 0.196367 | 0.518449 | 0.378758 | 528 | 28486224 |
| EVM0003611 | EVM0043216 | 0.068721 | 0.40826 | 0.168328 | 1089 | 22431845 |
| EVM0039609 | EVM0043216 | 0.016654 | 0.093454 | 0.178207 | 1089 | 5134839 |
| EVM0039609 | EVM0003611 | 0.067676 | 0.481887 | 0.140439 | 1089 | 26477322 |
| EVM0039609 | EVM0033748 | 0.074066 | 0.51698 | 0.143266 | 1089 | 28405511 |
| EVM0033748 | EVM0003611 | 0.030286 | 0.104034 | 0.291118 | 1101 | 5716148 |
| EVM0033748 | EVM0043216 | 0.077719 | 0.43979 | 0.176718 | 1089 | 24164283 |
| EVM0003762 | EVM0018037 | 0.021688 | 0.049414 | 0.438908 | 648 | 2715062 |
| EVM0004062 | EVM0006689 | 0.075399 | 0.377578 | 0.199692 | 2121 | 20746065 |
| EVM0022735 | EVM0004062 | 0.016878 | 0.091368 | 0.184721 | 2166 | 5020197 |
| EVM0022735 | EVM0007616 | 0.074022 | 0.409861 | 0.180603 | 2133 | 22519824 |
| EVM0022735 | EVM0006689 | 0.076398 | 0.407978 | 0.187261 | 2124 | 22416375 |
| EVM0007616 | EVM0006689 | 0.018428 | 0.118905 | 0.154984 | 2130 | 6533250 |
| EVM0007616 | EVM0004062 | 0.072696 | 0.391657 | 0.185613 | 2130 | 21519609 |
| EVM0004610 | EVM0001106 | 0.175938 | 0.39792 | 0.442145 | 1032 | 21863740 |
| EVM0025179 | EVM0032428 | 0.024437 | 0.149445 | 0.163519 | 534 | 8211274 |
| EVM0005180 | EVM0025179 | 0.106818 | 0.641185 | 0.166595 | 534 | 35229944 |
| EVM0005180 | EVM0032428 | 0.104258 | 0.763886 | 0.136484 | 534 | 41971781 |
| EVM0016569 | EVM0005180 | 0.016861 | 0.15074 | 0.111857 | 537 | 8282425 |
| EVM0016569 | EVM0025179 | 0.097157 | 0.522 | 0.186124 | 534 | 28681329 |
| EVM0016569 | EVM0040690 | 0.113739 | 0.499991 | 0.227482 | 537 | 27472006 |
| EVM0016569 | EVM0032428 | 0.097344 | 0.607004 | 0.160368 | 534 | 33351887 |
| EVM0040690 | EVM0005180 | 0.127665 | 0.608599 | 0.209768 | 537 | 33439520 |
| EVM0040690 | EVM0025179 | 0.142125 | 0.613697 | 0.231588 | 534 | 33719621 |
| EVM0040690 | EVM0032428 | 0.142407 | 0.732089 | 0.194521 | 534 | 40224681 |
| EVM0005349 | EVM0035055 | 0.014644 | 0.178427 | 0.08207 | 546 | 9803663 |
| EVM0012041 | EVM0035055 | 0.121772 | 0.418856 | 0.290726 | 537 | 23014075 |
| EVM0006637 | EVM0033580 | 0.035952 | 0.109875 | 0.327209 | 1635 | 6037089 |
| EVM0006637 | EVM0043933 | 0.082781 | 0.3808 | 0.217386 | 1608 | 20923094 |
| EVM0006637 | EVM0025980 | 0.0855 | 0.4033 | 0.212002 | 1608 | 22159330 |
| EVM0025980 | EVM0043933 | 0.017951 | 0.154427 | 0.116246 | 1620 | 8484981 |
| EVM0025980 | EVM0033580 | 0.080657 | 0.37836 | 0.213177 | 1611 | 20788998 |
| EVM0033580 | EVM0043933 | 0.077045 | 0.348381 | 0.221153 | 1611 | 19141799 |
| EVM0006649 | EVM0054070 | 0.042203 | 0.166687 | 0.253188 | 747 | 9158602 |
| EVM0006672 | EVM0004316 | 0.025612 | 0.173179 | 0.147894 | 750 | 9515339 |
| EVM0006682 | EVM0041629 | 0.140025 | 0.575248 | 0.243417 | 657 | 31607020 |
| EVM0053305 | EVM0041629 | 0.026529 | 0.13084 | 0.202758 | 657 | 7189026 |
| EVM0053305 | EVM0006682 | 0.121082 | 0.578959 | 0.209138 | 657 | 31810943 |
| EVM0054100 | EVM0006682 | 0.030555 | 0.149656 | 0.20417 | 615 | 8222833 |
| EVM0006866 | EVM0056353 | 0.049791 | 0.151669 | 0.328287 | 567 | 8333485 |
| EVM0050604 | EVM0006866 | 0.071439 | 0.424797 | 0.168171 | 552 | 23340473 |
| EVM0050604 | EVM0056353 | 0.094108 | 0.397581 | 0.236702 | 552 | 21845091 |
| EVM0006944 | EVM0041453 | 0.0144 | 0.09442 | 0.152514 | 678 | 5187923 |
| EVM0006944 | EVM0045225 | 0.113619 | 0.60983 | 0.186312 | 675 | 33507136 |
| EVM0022031 | EVM0041453 | 0.112195 | 0.740021 | 0.151611 | 675 | 40660482 |
| EVM0045225 | EVM0041453 | 0.114616 | 0.576764 | 0.198723 | 675 | 31690315 |
| EVM0022031 | EVM0045225 | 0.015499 | 0.151046 | 0.102614 | 675 | 8299237 |
| EVM0006972 | EVM0049755 | 0.020053 | 0.138258 | 0.145038 | 855 | 7596585 |
| EVM0041820 | EVM0036702 | 0.050165 | 0.176719 | 0.28387 | 762 | 9709851 |
| EVM0041820 | EVM0049755 | 0.082964 | 0.486956 | 0.170372 | 786 | 26755850 |
| EVM0041820 | EVM0006972 | 0.08585 | 0.469083 | 0.183016 | 786 | 25773766 |
| EVM0007543 | EVM0043720 | 0.021707 | 0.072097 | 0.301081 | 429 | 3961354 |
| EVM0008598 | EVM0019323 | 0.009067 | 0.007782 | 1.165096 | 573 | 427591.8 |
| EVM0008598 | EVM0045980 | 0.078898 | 0.642103 | 0.122874 | 534 | 35280400 |
| EVM0008598 | EVM0004107 | 0.075679 | 0.611001 | 0.12386 | 534 | 33571506 |
| EVM0019323 | EVM0004107 | 0.073121 | 0.626329 | 0.116745 | 534 | 34413686 |
| EVM0045980 | EVM0004107 | 0.014502 | 0.162668 | 0.089151 | 534 | 8937805 |
| EVM0045980 | EVM0019323 | 0.073229 | 0.661264 | 0.110741 | 534 | 36333181 |
| EVM0008628 | EVM0052641 | 0.049691 | 0.188739 | 0.263278 | 588 | 10370300 |
| EVM0008628 | EVM0034397 | 0.107002 | 0.484234 | 0.220973 | 585 | 26606244 |
| EVM0008628 | EVM0031973 | 0.115916 | 0.552706 | 0.209725 | 585 | 30368439 |
| EVM0031973 | EVM0052641 | 0.147213 | 0.584995 | 0.251649 | 588 | 32142568 |
| EVM0034397 | EVM0052641 | 0.131347 | 0.492682 | 0.266596 | 588 | 27070433 |
| EVM0034397 | EVM0031973 | 0.024647 | 0.118044 | 0.208799 | 591 | 6485941 |
| EVM0008654 | EVM0029842 | 0.03138 | 0.109121 | 0.287566 | 1071 | 5995677 |
| EVM0008654 | EVM0043629 | 0.101176 | 0.385842 | 0.262222 | 1080 | 21200085 |
| EVM0013126 | EVM0043629 | 0.02376 | 0.108296 | 0.219396 | 1113 | 5950323 |
| EVM0013126 | EVM0029842 | 0.083956 | 0.327831 | 0.256095 | 1104 | 18012694 |
| EVM0013126 | EVM0008654 | 0.098974 | 0.391701 | 0.252679 | 1080 | 21522008 |
| EVM0029842 | EVM0043629 | 0.085234 | 0.36241 | 0.235185 | 1098 | 19912659 |
| EVM0008767 | EVM0057697 | 0.029865 | 0.075157 | 0.397374 | 1545 | 4129489 |
| EVM0008767 | EVM0052615 | 0.072923 | 0.286265 | 0.254739 | 1539 | 15728867 |
| EVM0008767 | EVM0010409 | 0.079428 | 0.298861 | 0.265768 | 1539 | 16420934 |
| EVM0010409 | EVM0052615 | 0.01726 | 0.061936 | 0.27867 | 1584 | 3403100 |
| EVM0010409 | EVM0057697 | 0.0882 | 0.339298 | 0.259948 | 1542 | 18642766 |
| EVM0057697 | EVM0052615 | 0.078835 | 0.317219 | 0.248518 | 1542 | 17429599 |
| EVM0009316 | EVM0013573 | 0.026411 | 0.110111 | 0.239859 | 744 | 6050063 |
| EVM0009316 | EVM0014688 | 0.107813 | 0.402253 | 0.268022 | 738 | 22101808 |
| EVM0009316 | EVM0032370 | 0.108151 | 0.428081 | 0.252642 | 738 | 23520951 |
| EVM0013573 | EVM0014688 | 0.111594 | 0.395918 | 0.281861 | 738 | 21753745 |
| EVM0013573 | EVM0032370 | 0.105816 | 0.433624 | 0.244026 | 738 | 23825498 |
| EVM0014688 | EVM0032370 | 0.031846 | 0.137032 | 0.232401 | 765 | 7529257 |
| EVM0009409 | EVM0029622 | 0.061696 | 0.426458 | 0.144671 | 390 | 23431756 |
| EVM0012743 | EVM0009409 | 0.037359 | 0.087054 | 0.429149 | 387 | 4783194 |
| EVM0012743 | EVM0029622 | 0.071337 | 0.35968 | 0.198336 | 387 | 19762627 |
| EVM0049532 | EVM0029622 | 0.022265 | 0.138455 | 0.160811 | 414 | 7607426 |
| EVM0009928 | EVM0039635 | 0.031763 | 0.363775 | 0.087314 | 582 | 19987658 |
| EVM0017041 | EVM0009928 | 0.013456 | 0.088278 | 0.152425 | 582 | 4850447 |
| EVM0017041 | EVM0019845 | 0.034142 | 0.398679 | 0.085638 | 582 | 21905419 |
| EVM0017041 | EVM0039635 | 0.036433 | 0.413703 | 0.088065 | 582 | 22730926 |
| EVM0019845 | EVM0039635 | 0.011203 | 0.054855 | 0.204223 | 582 | 3013996 |
| EVM0019845 | EVM0009928 | 0.02487 | 0.349863 | 0.071086 | 582 | 19223216 |
| EVM0011412 | EVM0047762 | 0.041172 | 0.049915 | 0.824829 | 1476 | 2742610 |
| EVM0011412 | EVM0003138 | 0.047027 | 0.095099 | 0.494504 | 1431 | 5225208 |
| EVM0011412 | EVM0035539 | 0.063965 | 0.378541 | 0.168977 | 1278 | 20798954 |
| EVM0015665 | EVM0035539 | 0.007108 | 0.040211 | 0.176766 | 1296 | 2209376 |
| EVM0015665 | EVM0011412 | 0.06384 | 0.387708 | 0.16466 | 1281 | 21302619 |
| EVM0015665 | EVM0003138 | 0.063549 | 0.403402 | 0.157534 | 1299 | 22164940 |
| EVM0015665 | EVM0047762 | 0.118834 | 0.454233 | 0.261614 | 1278 | 24957869 |
| EVM0047762 | EVM0003138 | 0.090345 | 0.147158 | 0.613934 | 1470 | 8085604 |
| EVM0047762 | EVM0035539 | 0.117675 | 0.445519 | 0.264129 | 1275 | 24479046 |
| EVM0035539 | EVM0003138 | 0.062575 | 0.39427 | 0.15871 | 1296 | 21663204 |
| EVM0011531 | EVM0038265 | 0.013185 | 0.104455 | 0.126228 | 2058 | 5739259 |
| EVM0050425 | EVM0011531 | 0.085079 | 0.363216 | 0.234238 | 1785 | 19956913 |
| EVM0050425 | EVM0038265 | 0.08136 | 0.392886 | 0.207083 | 1785 | 21587148 |
| EVM0012824 | EVM0040570 | 0.020267 | 0.142673 | 0.142052 | 1512 | 7839195 |
| EVM0042515 | EVM0040570 | 0.036481 | 0.06038 | 0.6042 | 1440 | 3317560 |
| EVM0042515 | EVM0012824 | 0.052584 | 0.17012 | 0.309101 | 1440 | 9347234 |
| EVM0013188 | EVM0015672 | 0.043719 | 0.08392 | 0.520958 | 765 | 4610977 |
| EVM0013188 | EVM0051058 | 0.137305 | 0.453283 | 0.302912 | 660 | 24905650 |
| EVM0013188 | EVM0018299 | 0.146738 | 0.412301 | 0.3559 | 621 | 22653909 |
| EVM0051058 | EVM0018299 | 0.078817 | 0.106789 | 0.738066 | 621 | 5867502 |
| EVM0051058 | EVM0015672 | 0.140859 | 0.358129 | 0.39332 | 660 | 19677426 |
| EVM0015672 | EVM0018299 | 0.145077 | 0.351973 | 0.412183 | 621 | 19339169 |
| EVM0013502 | EVM0031724 | 0.025885 | 0.175268 | 0.147685 | 723 | 9630125 |
| EVM0013578 | EVM0040725 | 0.048485 | 0.095438 | 0.50803 | 687 | 5243839 |
| EVM0013578 | EVM0026277 | 0.076531 | 0.282632 | 0.270781 | 681 | 15529243 |
| EVM0013578 | EVM0017998 | 0.078579 | 0.384373 | 0.204434 | 669 | 21119418 |
| EVM0015962 | EVM0021255 | 0.051863 | 0.084597 | 0.613063 | 594 | 4648166 |
| EVM0015962 | EVM0054778 | 0.102593 | 0.330386 | 0.310525 | 594 | 18153050 |
| EVM0015962 | EVM0000771 | 0.135584 | 0.414614 | 0.327014 | 594 | 22780964 |
| EVM0021255 | EVM0000771 | 0.112345 | 0.373046 | 0.301156 | 618 | 20497010 |
| EVM0054778 | EVM0000771 | 0.023616 | 0.119507 | 0.197608 | 618 | 6566312 |
| EVM0054778 | EVM0021255 | 0.105257 | 0.297639 | 0.353641 | 618 | 16353783 |
| EVM0017235 | EVM0014312 | 0.018558 | 0.085808 | 0.21627 | 426 | 4714712 |
| EVM0025220 | EVM0054439 | 0.012513 | 0.12982 | 0.09639 | 423 | 7132943 |
| EVM0025220 | EVM0014312 | 0.076928 | 0.348835 | 0.220527 | 420 | 19166737 |
| EVM0025220 | EVM0017235 | 0.078739 | 0.45978 | 0.171254 | 420 | 25262624 |
| EVM0054439 | EVM0014312 | 0.082363 | 0.413587 | 0.199143 | 426 | 22724549 |
| EVM0054439 | EVM0017235 | 0.080788 | 0.512988 | 0.157486 | 426 | 28186165 |
| EVM0017324 | EVM0039499 | 0.032457 | 0.08359 | 0.388281 | 939 | 4592874 |
| EVM0017324 | EVM0028978 | 0.146487 | 0.495668 | 0.295535 | 927 | 27234498 |
| EVM0017324 | EVM0054684 | 0.142457 | 0.534453 | 0.266547 | 921 | 29365542 |
| EVM0028978 | EVM0054684 | 0.02688 | 0.100107 | 0.268515 | 933 | 5500369 |
| EVM0028978 | EVM0039499 | 0.144764 | 0.455901 | 0.317533 | 924 | 25049531 |
| EVM0039499 | EVM0054684 | 0.149944 | 0.499838 | 0.299986 | 924 | 27463652 |
| EVM0017733 | EVM0001609 | 0.03137 | 0.060447 | 0.518965 | 834 | 3321276 |
| EVM0043703 | EVM0017733 | 0.059462 | 0.091417 | 0.650442 | 954 | 5022932 |
| EVM0043703 | EVM0001609 | 0.089186 | 0.153467 | 0.581143 | 834 | 8432262 |
| EVM0018397 | EVM0043533 | 0.042816 | 0.125317 | 0.341663 | 1899 | 6885538 |
| EVM0018598 | EVM0057013 | 0.020396 | 0.078968 | 0.258286 | 531 | 4338920 |
| EVM0018678 | EVM0016157 | 0.105645 | 0.567517 | 0.186154 | 591 | 31182280 |
| EVM0027008 | EVM0018678 | 0.117238 | 0.254428 | 0.46079 | 594 | 13979582 |
| EVM0027008 | EVM0029243 | 0.179338 | 1.014972 | 0.176692 | 582 | 55767680 |
| EVM0029243 | EVM0016157 | 0.025888 | 0.284953 | 0.09085 | 615 | 15656732 |
| EVM0029243 | EVM0018678 | 0.10071 | 0.640273 | 0.157292 | 585 | 35179858 |
| EVM0018852 | EVM0012457 | 0.03353 | 0.093461 | 0.358759 | 792 | 5135196 |
| EVM0018852 | EVM0026368 | 0.126345 | 0.411993 | 0.306667 | 804 | 22636971 |
| EVM0026368 | EVM0031439 | 0.038854 | 0.104113 | 0.373192 | 819 | 5720493 |
| EVM0018983 | EVM0023703 | 0.058355 | 0.108817 | 0.536268 | 720 | 5978939 |
| EVM0019656 | EVM0036581 | 0.029874 | 0.073453 | 0.406709 | 1014 | 4035887 |
| EVM0023967 | EVM0056670 | 0.018141 | 0.136466 | 0.132932 | 1221 | 7498131 |
| EVM0023967 | EVM0036581 | 0.139281 | 0.501477 | 0.277741 | 1086 | 27553676 |
| EVM0056670 | EVM0036581 | 0.122608 | 0.515589 | 0.237802 | 1086 | 28329056 |
| EVM0019924 | EVM0055695 | 0.024554 | 0.071716 | 0.342374 | 699 | 3940430 |
| EVM0054964 | EVM0019924 | 0.090174 | 0.375999 | 0.239826 | 681 | 20659281 |
| EVM0054964 | EVM0055695 | 0.091406 | 0.378652 | 0.241399 | 681 | 20805043 |
| EVM0055475 | EVM0054964 | 0.014405 | 0.055702 | 0.258607 | 684 | 3060574 |
| EVM0055475 | EVM0019924 | 0.083573 | 0.422818 | 0.197658 | 681 | 23231773 |
| EVM0055475 | EVM0055695 | 0.085851 | 0.442433 | 0.194043 | 681 | 24309517 |
| EVM0020349 | EVM0050804 | 0.00729 | 0.073341 | 0.099405 | 528 | 4029716 |
| EVM0020349 | EVM0026318 | 0.067533 | 0.276278 | 0.244438 | 525 | 15180123 |
| EVM0026318 | EVM0050804 | 0.072885 | 0.276278 | 0.263811 | 525 | 15180123 |
| EVM0020564 | EVM0000418 | 0.04112 | 0.081874 | 0.502227 | 1374 | 4498596 |
| EVM0021312 | EVM0024408 | 0.034536 | 0.097007 | 0.356017 | 798 | 5330047 |
| EVM0023162 | EVM0046939 | 0.041203 | 0.101789 | 0.404785 | 621 | 5592821 |
| EVM0045686 | EVM0029866 | 0.02339 | 0.11818 | 0.197915 | 624 | 6493409 |
| EVM0045686 | EVM0046939 | 0.15973 | 0.408771 | 0.390758 | 618 | 22459944 |
| EVM0046939 | EVM0029866 | 0.155438 | 0.418496 | 0.37142 | 618 | 22994273 |
| EVM0023217 | EVM0030954 | 0.024397 | 0.135821 | 0.179623 | 1305 | 7462719 |
| EVM0023217 | EVM0000045 | 0.129777 | 0.358893 | 0.361603 | 1353 | 19719374 |
| EVM0023217 | EVM0047172 | 0.136891 | 0.355294 | 0.385289 | 1344 | 19521639 |
| EVM0047172 | EVM0000045 | 0.052671 | 0.137914 | 0.381914 | 1344 | 7577690 |
| EVM0023232 | EVM0055941 | 0.037614 | 0.101528 | 0.370485 | 1125 | 5578435 |
| EVM0023642 | EVM0053591 | 0.070286 | 0.434083 | 0.161919 | 462 | 23850725 |
| EVM0029410 | EVM0023642 | 0.01339 | 0.063338 | 0.211408 | 492 | 3480126 |
| EVM0029410 | EVM0053591 | 0.072318 | 0.424689 | 0.170285 | 462 | 23334540 |
| EVM0049893 | EVM0053591 | 0.02309 | 0.145854 | 0.158308 | 465 | 8013942 |
| EVM0049893 | EVM0023642 | 0.05698 | 0.45217 | 0.126015 | 462 | 24844479 |
| EVM0027771 | EVM0052364 | 0.059146 | 0.165386 | 0.357627 | 468 | 9087121 |
| EVM0028403 | EVM0045883 | 0.039563 | 0.088211 | 0.448496 | 822 | 4846783 |
| EVM0028403 | EVM0052605 | 0.035419 | 0.109758 | 0.322701 | 822 | 6030669 |
| EVM0045883 | EVM0052605 | 0.019738 | 0.030114 | 0.655452 | 828 | 1654590 |
| EVM0030182 | EVM0021270 | 0.034569 | 0.109555 | 0.31554 | 1281 | 6019500 |
| EVM0030767 | EVM0043812 | 0.03012 | 0.128391 | 0.234594 | 1101 | 7054475 |
| EVM0032156 | EVM0025370 | 0.029939 | 0.046609 | 0.642355 | 993 | 2560918 |
| EVM0033660 | EVM0037879 | 0.035684 | 0.134636 | 0.265037 | 642 | 7397583 |
| EVM0034096 | EVM0032021 | 0.091371 | 0.634988 | 0.143894 | 747 | 34889444 |
| EVM0034096 | EVM0042411 | 0.093872 | 0.607834 | 0.154438 | 744 | 33397455 |
| EVM0042411 | EVM0032021 | 0.022879 | 0.109897 | 0.208187 | 753 | 6038273 |
| EVM0042411 | EVM0053799 | 0.073396 | 0.517405 | 0.141854 | 549 | 28428868 |
| EVM0053799 | EVM0032021 | 0.075872 | 0.521842 | 0.145394 | 549 | 28672631 |
| EVM0034620 | EVM0031168 | 0.023661 | 0.087675 | 0.269876 | 987 | 4817283 |
| EVM0035890 | EVM0018103 | 0.038066 | 0.066088 | 0.575989 | 906 | 3631234 |
| EVM0036168 | EVM0036052 | 0.039311 | 0.082645 | 0.475664 | 1455 | 4540937 |
| EVM0036425 | EVM0042153 | 0.022371 | 0.140978 | 0.158688 | 936 | 7746023 |
| EVM0037606 | EVM0054822 | 0 | 0 | NaN | 849 | 0 |
| EVM0037606 | EVM0024412 | 0.023887 | 0.074689 | 0.319826 | 849 | 4103765 |
| EVM0054822 | EVM0024412 | 0.023887 | 0.074689 | 0.319826 | 849 | 4103765 |
| EVM0038570 | EVM0023183 | 0.022705 | 0.028358 | 0.800638 | 1398 | 1558146 |
| EVM0038570 | EVM0051919 | 0.039965 | 0.114081 | 0.350322 | 1368 | 6268204 |
| EVM0051919 | EVM0023183 | 0.038837 | 0.099518 | 0.390256 | 1368 | 5467997 |
| EVM0038648 | EVM0017086 | 0.091037 | 0.444614 | 0.204755 | 636 | 24429366 |
| EVM0045763 | EVM0038648 | 0.010091 | 0.079306 | 0.127242 | 645 | 4357445 |
| EVM0045763 | EVM0017086 | 0.098484 | 0.518562 | 0.189917 | 621 | 28492400 |
| EVM0039575 | EVM0006536 | 0.029068 | 0.121186 | 0.239862 | 1497 | 6658561 |
| EVM0040155 | EVM0028452 | 0.026614 | 0.136861 | 0.194458 | 858 | 7519861 |
| EVM0040310 | EVM0040588 | 0.039968 | 0.149781 | 0.266841 | 1038 | 8229746 |
| EVM0042683 | EVM0032101 | 0.129147 | 0.551555 | 0.234152 | 855 | 30305233 |
| EVM0054412 | EVM0042683 | 0.037199 | 0.117352 | 0.316989 | 873 | 6447905 |
| EVM0054412 | EVM0032101 | 0.116796 | 0.487377 | 0.239641 | 855 | 26778948 |
| EVM0043935 | EVM0007646 | 0.130015 | 0.372611 | 0.348929 | 855 | 20473141 |
| EVM0044050 | EVM0006666 | 0.032921 | 0.128657 | 0.25588 | 570 | 7069056 |
| EVM0044087 | EVM0010374 | 0.036953 | 0.115819 | 0.319062 | 792 | 6363661 |
| EVM0044386 | EVM0020075 | 0.039686 | 0.088382 | 0.449027 | 756 | 4856165 |
| EVM0045646 | EVM0008755 | 0.026001 | 0.138398 | 0.187869 | 714 | 7604284 |
| EVM0045726 | EVM0024472 | 0.029976 | 0.152089 | 0.197095 | 1071 | 8356532 |
| EVM0046023 | EVM0055854 | 0.04494 | 0.191238 | 0.234993 | 1002 | 10507589 |
| EVM0049776 | EVM0044805 | 0.045626 | 0.202254 | 0.225588 | 1005 | 11112855 |
| EVM0049898 | EVM0052258 | 0.027069 | 0.124064 | 0.218186 | 684 | 6816723 |
| EVM0051611 | EVM0032830 | 0.054115 | 0.080472 | 0.67247 | 498 | 4421540 |
| EVM0051729 | EVM0025018 | 0.037744 | 0.075041 | 0.502978 | 1347 | 4123118 |
| EVM0051790 | EVM0035105 | 0 | 0 | NaN | 768 | 0 |
| EVM0052580 | EVM0044116 | 0.040009 | 0.089271 | 0.44818 | 813 | 4905006 |
| EVM0054355 | EVM0044712 | 0.035682 | 0.123802 | 0.288218 | 909 | 6802309 |
| EVM0054855 | EVM0055399 | 0.045295 | 0.183585 | 0.246723 | 1242 | 10087110 |
| EVM0055401 | EVM0028773 | 0.044754 | 0.096855 | 0.462072 | 1401 | 5321691 |
| EVM0055887 | EVM0042752 | 0.039128 | 0.061148 | 0.639884 | 630 | 3359796 |
| EVM0056869 | EVM0037206 | 0.03242 | 0.105503 | 0.30729 | 615 | 5796856 |
| EVM0056869 | EVM0056893 | 0.068569 | 0.376397 | 0.182171 | 615 | 20681134 |
| EVM0056869 | EVM0057138 | 0.079079 | 0.442956 | 0.178526 | 615 | 24338223 |
| EVM0056893 | EVM0057138 | 0.012647 | 0.146714 | 0.086205 | 621 | 8061190 |
| EVM0056893 | EVM0037206 | 0.078753 | 0.416391 | 0.189133 | 609 | 22878616 |
| EVM0057138 | EVM0037206 | 0.084724 | 0.501027 | 0.169101 | 609 | 27528971 |
| EVM0057140 | EVM0045295 | 0.093885 | 0.39508 | 0.237636 | 825 | 21707696 |

**Table S7 Syteny orthologue gene pairs between S. matsudana and two related Salicaceae species, P. trichocarpa and S. purpurea**

| Synteny AP2/ERF gene pairs between P. trichocarpa and S. matsudana | | Synteny AP2/ERF gene pairs between S. matsudana and S. purpurea | |
| --- | --- | --- | --- |
| P. trichocarpa | S. matsudana | S. matsudana | S. purpurea |
| Potri.001G067600 | EVM0013578 | EVM0040310 | Sapur.001G031600 |
| Potri.001G079600 | EVM0055695 | EVM0054250 | Sapur.001G038700 |
| Potri.001G079800 | EVM0049755 | EVM0013578 | Sapur.001G052600 |
| Potri.001G079900 | EVM0006603 | EVM0055695 | Sapur.001G064100 |
| Potri.001G092400 | EVM0000460 | EVM0049755 | Sapur.001G064200 |
| Potri.001G094800 | EVM0024472 | EVM0006603 | Sapur.001G064300 |
| Potri.001G018400 | EVM0056730 | EVM0000460 | Sapur.001G075700 |
| Potri.001G041500 | EVM0040310 | EVM0024472 | Sapur.001G077800 |
| Potri.001G048200 | EVM0054250 | EVM0021344 | Sapur.001G163400 |
| Potri.001G187500 | EVM0021344 | EVM0056730 | Sapur.001G015500 |
| Potri.001G067600 | EVM0017998 | EVM0054250 | Sapur.003G136500 |
| Potri.001G069300 | EVM0042683 | EVM0055695 | Sapur.003G111700 |
| Potri.001G079600 | EVM0055475 | EVM0000460 | Sapur.003G102600 |
| Potri.001G079800 | EVM0041820 | EVM0057013 | Sapur.001G090300 |
| Potri.001G094800 | EVM0034620 | EVM0055475 | Sapur.001G064100 |
| Potri.001G110500 | EVM0057013 | EVM0017998 | Sapur.001G052600 |
| Potri.001G110800 | EVM0004638 | EVM0000144 | Sapur.001G015500 |
| Potri.001G155700 | EVM0018299 | EVM0012457 | Sapur.003G046200 |
| Potri.001G157100 | EVM0012457 | EVM0018299 | Sapur.003G047300 |
| Potri.001G041500 | EVM0009672 | EVM0004638 | Sapur.003G085700 |
| Potri.001G048200 | EVM0011072 | EVM0057013 | Sapur.003G085800 |
| Potri.001G004700 | EVM0043935 | EVM0034620 | Sapur.003G099900 |
| Potri.001G018400 | EVM0000144 | EVM0038704 | Sapur.003G102600 |
| Potri.001G397200 | EVM0053305 | EVM0006649 | Sapur.003G111500 |
| Potri.001G181500 | EVM0040690 | EVM0041820 | Sapur.003G111600 |
| Potri.001G110700 | EVM0040623 | EVM0055475 | Sapur.003G111700 |
| Potri.001G110800 | EVM0026062 | EVM0042683 | Sapur.003G119100 |
| Potri.001G154100 | EVM0024412 | EVM0017998 | Sapur.003G120300 |
| Potri.001G154200 | EVM0013811 | EVM0011072 | Sapur.003G136500 |
| Potri.001G155700 | EVM0015672 | EVM0009672 | Sapur.003G140600 |
| Potri.001G048200 | EVM0034803 | EVM0000144 | Sapur.003G157900 |
| Potri.001G004700 | EVM0007646 | EVM0043935 | Sapur.003G166800 |
| Potri.001G018400 | EVM0035153 | EVM0025220 | Sapur.002G030200 |
| Potri.001G157100 | EVM0026368 | EVM0025220 | Sapur.005G177600 |
| Potri.001G187500 | EVM0018160 | EVM0035905 | Sapur.005G177700 |
| Potri.001G067600 | EVM0040725 | EVM0045646 | Sapur.005G177800 |
| Potri.001G069300 | EVM0032101 | EVM0013126 | Sapur.005G060200 |
| Potri.001G079600 | EVM0019924 | EVM0017689 | Sapur.005G186300 |
| Potri.001G079800 | EVM0006972 | EVM0047172 | Sapur.005G153300 |
| Potri.001G079900 | EVM0046641 | EVM0032803 | Sapur.005G119800 |
| Potri.001G092400 | EVM0015466 | EVM0057697 | Sapur.005G108500 |
| Potri.001G313500 | EVM0009817 | EVM0026318 | Sapur.005G108300 |
| Potri.001G315300 | EVM0023183 | EVM0026318 | Sapur.007G043700 |
| Potri.001G356100 | EVM0003762 | EVM0013126 | Sapur.007G082500 |
| Potri.001G397200 | EVM0006682 | EVM0005349 | Sapur.011G042400 |
| Potri.001G453100 | EVM0052812 | EVM0043216 | Sapur.008G094300 |
| Potri.001G092400 | EVM0014537 | EVM0023967 | Sapur.010G199800 |
| Potri.001G110500 | EVM0018598 | EVM0054855 | Sapur.010G023900 |
| Potri.001G110800 | EVM0029808 | EVM0043216 | Sapur.010G099700 |
| Potri.001G154100 | EVM0030239 | EVM0020075 | Sapur.010G005200 |
| Potri.001G155700 | EVM0051058 | EVM0006689 | Sapur.010G140700 |
| Potri.001G157100 | EVM0018852 | EVM0018103 | Sapur.010G143500 |
| Potri.001G079600 | EVM0054964 | EVM0025370 | Sapur.010G146100 |
| Potri.001G018400 | EVM0050911 | EVM0036052 | Sapur.010G171900 |
| Potri.001G397200 | EVM0041629 | EVM0029842 | Sapur.005G060200 |
| Potri.001G313500 | EVM0044977 | EVM0008654 | Sapur.007G082500 |
| Potri.001G315300 | EVM0051919 | EVM0051566 | Sapur.007G040500 |
| Potri.001G356100 | EVM0018037 | EVM0020349 | Sapur.007G043700 |
| Potri.001G397200 | EVM0054100 | EVM0010409 | Sapur.007G043500 |
| Potri.002G039100 | EVM0035905 | EVM0045686 | Sapur.007G123400 |
| Potri.002G039200 | EVM0025220 | EVM0029842 | Sapur.007G082500 |
| Potri.002G065600 | EVM0047172 | EVM0050425 | Sapur.007G005500 |
| Potri.002G029400 | EVM0017689 | EVM0053305 | Sapur.011G079300 |
| Potri.002G124000 | EVM0053591 | EVM0050804 | Sapur.005G108300 |
| Potri.002G201600 | EVM0028978 | EVM0029866 | Sapur.007G123400 |
| Potri.002G172300 | EVM0031973 | EVM0015843 | Sapur.007G040500 |
| Potri.002G153500 | EVM0027493 | EVM0052615 | Sapur.007G043500 |
| Potri.002G201600 | EVM0017324 | EVM0050804 | Sapur.007G043700 |
| Potri.002G085600 | EVM0033660 | EVM0049295 | Sapur.006G114500 |
| Potri.002G094200 | EVM0044805 | EVM0035105 | Sapur.006G114400 |
| Potri.002G114800 | EVM0018397 | EVM0000418 | Sapur.006G109100 |
| Potri.002G124000 | EVM0029410 | EVM0016157 | Sapur.006G179000 |
| Potri.002G039000 | EVM0018983 | EVM0001533 | Sapur.006G085100 |
| Potri.002G039100 | EVM0008128 | EVM0057140 | Sapur.006G042000 |
| Potri.002G039200 | EVM0017235 | EVM0015665 | Sapur.006G037700 |
| Potri.002G039300 | EVM0052364 | EVM0033580 | Sapur.006G137200 |
| Potri.002G043300 | EVM0051611 | EVM0009928 | Sapur.006G215800 |
| Potri.002G246100 | EVM0036226 | EVM0015665 | Sapur.016G050300 |
| Potri.002G141200 | EVM0005644 | EVM0033580 | Sapur.018G067800 |
| Potri.002G141200 | EVM0055308 | EVM0043703 | Sapur.013G141800 |
| Potri.002G114800 | EVM0043533 | EVM0055401 | Sapur.013G126800 |
| Potri.002G124000 | EVM0023642 | EVM0016569 | Sapur.013G096000 |
| Potri.002G141200 | EVM0000005 | EVM0001548 | Sapur.013G094600 |
| Potri.002G167400 | EVM0054355 | EVM0045225 | Sapur.013G041800 |
| Potri.002G172300 | EVM0008628 | EVM0021312 | Sapur.013G052600 |
| Potri.002G029400 | EVM0000172 | EVM0016569 | Sapur.019G073000 |
| Potri.002G039000 | EVM0023703 | EVM0001548 | Sapur.019G071100 |
| Potri.002G039100 | EVM0040250 | EVM0030823 | Sapur.019G035300 |
| Potri.002G039200 | EVM0014312 | EVM0043720 | Sapur.019G084600 |
| Potri.002G039300 | EVM0027771 | EVM0053591 | Sapur.014G014900 |
| Potri.002G043300 | EVM0039676 | EVM0038265 | Sapur.014G004000 |
| Potri.002G201600 | EVM0039499 | EVM0028978 | Sapur.014G101000 |
| Potri.002G065600 | EVM0023217 | EVM0031973 | Sapur.014G078300 |
| Potri.002G085600 | EVM0037879 | EVM0027493 | Sapur.014G058500 |
| Potri.002G094200 | EVM0049776 | EVM0024365 | Sapur.001G090300 |
| Potri.002G124000 | EVM0049893 | EVM0040623 | Sapur.001G090400 |
| Potri.002G141200 | EVM0007023 | EVM0026062 | Sapur.001G090500 |
| Potri.002G201600 | EVM0054684 | EVM0024412 | Sapur.001G127700 |
| Potri.002G039100 | EVM0001248 | EVM0015672 | Sapur.001G130000 |
| Potri.002G039200 | EVM0054439 | EVM0013811 | Sapur.001G127900 |
| Potri.002G094200 | EVM0003769 | EVM0026368 | Sapur.001G140100 |
| Potri.003G136300 | EVM0024472 | EVM0040690 | Sapur.001G152400 |
| Potri.003G139300 | EVM0000460 | EVM0018160 | Sapur.001G163400 |
| Potri.003G150800 | EVM0049755 | EVM0015466 | Sapur.001G075700 |
| Potri.003G151000 | EVM0055695 | EVM0034803 | Sapur.001G038700 |
| Potri.003G162500 | EVM0013578 | EVM0040725 | Sapur.001G052600 |
| Potri.003G179900 | EVM0054250 | EVM0032101 | Sapur.001G054400 |
| Potri.003G185300 | EVM0040310 | EVM0019924 | Sapur.001G064100 |
| Potri.003G050700 | EVM0021344 | EVM0006972 | Sapur.001G064200 |
| Potri.003G077700 | EVM0012457 | EVM0046641 | Sapur.001G064300 |
| Potri.003G079300 | EVM0018299 | EVM0007646 | Sapur.001G003600 |
| Potri.003G121200 | EVM0057013 | EVM0035153 | Sapur.001G015500 |
| Potri.003G136300 | EVM0034620 | EVM0019924 | Sapur.003G111700 |
| Potri.003G139300 | EVM0038704 | EVM0006972 | Sapur.003G111600 |
| Potri.003G150700 | EVM0006649 | EVM0040725 | Sapur.003G120300 |
| Potri.003G150800 | EVM0041820 | EVM0035153 | Sapur.003G157900 |
| Potri.003G151000 | EVM0055475 | EVM0051961 | Sapur.017G072300 |
| Potri.003G161000 | EVM0042683 | EVM0023162 | Sapur.017G007100 |
| Potri.003G162500 | EVM0017998 | EVM0049616 | Sapur.008G056300 |
| Potri.003G179900 | EVM0011072 | EVM0055887 | Sapur.008G058500 |
| Potri.003G185300 | EVM0009672 | EVM0022735 | Sapur.008G061100 |
| Potri.003G205700 | EVM0000144 | EVM0012743 | Sapur.008G134600 |
| Potri.003G150800 | EVM0006972 | EVM0036069 | Sapur.008G134700 |
| Potri.003G151000 | EVM0019924 | EVM0045763 | Sapur.008G134800 |
| Potri.003G161000 | EVM0032101 | EVM0042467 | Sapur.008G150000 |
| Potri.003G162500 | EVM0040725 | EVM0055854 | Sapur.008G165200 |
| Potri.003G179900 | EVM0034803 | EVM0036069 | Sapur.010G040100 |
| Potri.003G185300 | EVM0040588 | EVM0024408 | Sapur.013G052600 |
| Potri.003G079300 | EVM0015672 | EVM0028773 | Sapur.013G126800 |
| Potri.003G081200 | EVM0024412 | EVM0022031 | Sapur.013G041800 |
| Potri.003G077700 | EVM0026368 | EVM0050604 | Sapur.013G096100 |
| Potri.003G050700 | EVM0018160 | EVM0005180 | Sapur.013G096000 |
| Potri.003G205700 | EVM0035153 | EVM0022064 | Sapur.013G094600 |
| Potri.003G064700 | EVM0028655 | EVM0025018 | Sapur.019G098000 |
| Potri.003G077700 | EVM0018852 | EVM0044050 | Sapur.019G067700 |
| Potri.003G079300 | EVM0051058 | EVM0025179 | Sapur.019G073000 |
| Potri.003G080600 | EVM0030192 | EVM0006866 | Sapur.019G073100 |
| Potri.003G081200 | EVM0030239 | EVM0019845 | Sapur.006G215800 |
| Potri.003G121200 | EVM0018598 | EVM0043933 | Sapur.018G067800 |
| Potri.003G139300 | EVM0014537 | EVM0040155 | Sapur.018G062500 |
| Potri.003G205700 | EVM0050911 | EVM0018678 | Sapur.018G036500 |
| Potri.003G212800 | EVM0043812 | EVM0045980 | Sapur.018G023800 |
| Potri.003G220200 | EVM0046342 | EVM0019845 | Sapur.018G018300 |
| Potri.003G150700 | EVM0054070 | EVM0011459 | Sapur.009G079700 |
| Potri.003G150800 | EVM0036702 | EVM0044116 | Sapur.009G116700 |
| Potri.003G151000 | EVM0054964 | EVM0045295 | Sapur.006G042000 |
| Potri.003G033000 | EVM0028845 | EVM0006682 | Sapur.016G255700 |
| Potri.004G051700 | EVM0005349 | EVM0003762 | Sapur.016G227700 |
| Potri.004G187001 | EVM0044116 | EVM0023183 | Sapur.016G191900 |
| Potri.004G141200 | EVM0011459 | EVM0009817 | Sapur.016G190500 |
| Potri.004G141200 | EVM0002769 | EVM0027529 | Sapur.016G080700 |
| Potri.004G187001 | EVM0052580 | EVM0011412 | Sapur.016G050300 |
| Potri.004G047500 | EVM0049397 | EVM0045295 | Sapur.016G047500 |
| Potri.004G051800 | EVM0045883 | EVM0021255 | Sapur.016G015300 |
| Potri.004G047500 | EVM0001371 | EVM0052364 | Sapur.002G030300 |
| Potri.004G051700 | EVM0035055 | EVM0017235 | Sapur.002G030200 |
| Potri.004G047500 | EVM0004204 | EVM0008128 | Sapur.002G030100 |
| Potri.004G051700 | EVM0012041 | EVM0018983 | Sapur.002G030000 |
| Potri.004G051800 | EVM0028403 | EVM0029410 | Sapur.002G100900 |
| Potri.004G141200 | EVM0043678 | EVM0018397 | Sapur.002G092900 |
| Potri.005G077300 | EVM0013126 | EVM0044805 | Sapur.002G075900 |
| Potri.005G233300 | EVM0017689 | EVM0033660 | Sapur.002G068300 |
| Potri.005G140700 | EVM0057697 | EVM0017324 | Sapur.002G159300 |
| Potri.005G140900 | EVM0026318 | EVM0030954 | Sapur.002G051800 |
| Potri.005G223100 | EVM0025220 | EVM0005644 | Sapur.002G115400 |
| Potri.005G223200 | EVM0035905 | EVM0017235 | Sapur.005G177600 |
| Potri.005G223300 | EVM0045646 | EVM0033660 | Sapur.005G137400 |
| Potri.005G195000 | EVM0047172 | EVM0002769 | Sapur.009G079700 |
| Potri.005G077300 | EVM0029842 | EVM0052580 | Sapur.009G116700 |
| Potri.005G077300 | EVM0008654 | EVM0013502 | Sapur.012G082100 |
| Potri.005G140700 | EVM0010409 | EVM0010374 | Sapur.012G103000 |
| Potri.005G140900 | EVM0020349 | EVM0032370 | Sapur.012G103100 |
| Potri.005G140700 | EVM0052615 | EVM0022896 | Sapur.012G020100 |
| Potri.005G140900 | EVM0050804 | EVM0018852 | Sapur.001G140100 |
| Potri.005G223100 | EVM0017235 | EVM0029808 | Sapur.001G090500 |
| Potri.005G223200 | EVM0008128 | EVM0018598 | Sapur.001G090300 |
| Potri.005G176000 | EVM0033660 | EVM0014537 | Sapur.001G075700 |
| Potri.005G176000 | EVM0055116 | EVM0050911 | Sapur.001G015500 |
| Potri.005G233300 | EVM0047653 | EVM0054964 | Sapur.001G064100 |
| Potri.005G223100 | EVM0014312 | EVM0018852 | Sapur.003G046200 |
| Potri.005G223200 | EVM0040250 | EVM0051058 | Sapur.003G047300 |
| Potri.005G176000 | EVM0037879 | EVM0030192 | Sapur.003G049100 |
| Potri.005G195000 | EVM0023217 | EVM0030239 | Sapur.003G049200 |
| Potri.005G077300 | EVM0043629 | EVM0029808 | Sapur.003G085700 |
| Potri.005G087200 | EVM0004610 | EVM0018598 | Sapur.003G085800 |
| Potri.005G223100 | EVM0054439 | EVM0014537 | Sapur.003G102600 |
| Potri.005G223200 | EVM0001248 | EVM0028845 | Sapur.003G017600 |
| Potri.005G223300 | EVM0008755 | EVM0005032 | Sapur.003G026800 |
| Potri.005G148400 | EVM0009993 | EVM0050911 | Sapur.003G157900 |
| Potri.005G168700 | EVM0003769 | EVM0043812 | Sapur.003G161900 |
| Potri.005G176000 | EVM0024690 | EVM0054964 | Sapur.003G111700 |
| Potri.005G140900 | EVM0046201 | EVM0036702 | Sapur.003G111600 |
| Potri.006G132400 | EVM0000418 | EVM0054070 | Sapur.003G111500 |
| Potri.006G138800 | EVM0035105 | EVM0049397 | Sapur.004G031600 |
| Potri.006G138900 | EVM0049295 | EVM0052258 | Sapur.004G031700 |
| Potri.006G054500 | EVM0057140 | EVM0045883 | Sapur.004G035100 |
| Potri.006G069400 | EVM0056869 | EVM0047653 | Sapur.005G186300 |
| Potri.006G104200 | EVM0001533 | EVM0055116 | Sapur.005G137400 |
| Potri.006G261200 | EVM0009928 | EVM0029243 | Sapur.006G179000 |
| Potri.006G167700 | EVM0033580 | EVM0032021 | Sapur.006G197300 |
| Potri.006G218200 | EVM0016157 | EVM0019323 | Sapur.006G209800 |
| Potri.006G253800 | EVM0045980 | EVM0008598 | Sapur.006G209800 |
| Potri.006G261200 | EVM0019845 | EVM0017041 | Sapur.006G215800 |
| Potri.006G163400 | EVM0040155 | EVM0035539 | Sapur.006G037700 |
| Potri.006G167700 | EVM0043933 | EVM0000771 | Sapur.006G015800 |
| Potri.006G054500 | EVM0045295 | EVM0020564 | Sapur.006G109100 |
| Potri.006G132400 | EVM0020564 | EVM0003861 | Sapur.006G114400 |
| Potri.006G138800 | EVM0003861 | EVM0006637 | Sapur.006G137200 |
| Potri.006G238600 | EVM0032021 | EVM0052698 | Sapur.006G085100 |
| Potri.006G253800 | EVM0019323 | EVM0055308 | Sapur.014G042400 |
| Potri.006G253800 | EVM0008598 | EVM0052610 | Sapur.014G035700 |
| Potri.006G261200 | EVM0017041 | EVM0035539 | Sapur.016G050300 |
| Potri.006G021000 | EVM0000771 | EVM0017041 | Sapur.018G018300 |
| Potri.006G104200 | EVM0052698 | EVM0046023 | Sapur.008G165200 |
| Potri.006G167700 | EVM0006637 | EVM0003611 | Sapur.008G094300 |
| Potri.006G218200 | EVM0029243 | EVM0042153 | Sapur.008G096700 |
| Potri.006G069400 | EVM0037206 | EVM0019656 | Sapur.008G006800 |
| Potri.006G021000 | EVM0015962 | EVM0039575 | Sapur.008G034200 |
| Potri.006G253800 | EVM0004107 | EVM0019343 | Sapur.008G150000 |
| Potri.006G069400 | EVM0057138 | EVM0004062 | Sapur.008G061100 |
| Potri.006G163400 | EVM0028452 | EVM0012873 | Sapur.008G134700 |
| Potri.006G167700 | EVM0025980 | EVM0034491 | Sapur.008G160400 |
| Potri.006G069400 | EVM0056893 | EVM0004062 | Sapur.010G140700 |
| Potri.007G090600 | EVM0013126 | EVM0003611 | Sapur.010G099700 |
| Potri.007G046500 | EVM0026318 | EVM0012873 | Sapur.010G040100 |
| Potri.007G043400 | EVM0051566 | EVM0014688 | Sapur.012G103100 |
| Potri.007G046200 | EVM0010409 | EVM0044087 | Sapur.012G103000 |
| Potri.007G046500 | EVM0020349 | EVM0031724 | Sapur.012G082100 |
| Potri.007G138100 | EVM0045686 | EVM0039609 | Sapur.008G094300 |
| Potri.007G007400 | EVM0050425 | EVM0044386 | Sapur.010G005200 |
| Potri.007G011600 | EVM0012824 | EVM0055399 | Sapur.010G023900 |
| Potri.007G090600 | EVM0029842 | EVM0049532 | Sapur.010G040000 |
| Potri.007G090600 | EVM0008654 | EVM0002921 | Sapur.010G040100 |
| Potri.007G011600 | EVM0040570 | EVM0025231 | Sapur.010G040200 |
| Potri.007G043400 | EVM0015843 | EVM0007616 | Sapur.010G140700 |
| Potri.007G046200 | EVM0052615 | EVM0035890 | Sapur.010G143500 |
| Potri.007G046500 | EVM0050804 | EVM0032156 | Sapur.010G146100 |
| Potri.007G138100 | EVM0029866 | EVM0039609 | Sapur.010G099700 |
| Potri.007G046500 | EVM0046201 | EVM0001371 | Sapur.004G031600 |
| Potri.007G090600 | EVM0043629 | EVM0035055 | Sapur.011G042400 |
| Potri.007G076800 | EVM0004610 | EVM0048064 | Sapur.011G039300 |
| Potri.008G071100 | EVM0025370 | EVM0015962 | Sapur.006G015800 |
| Potri.008G073600 | EVM0018103 | EVM0018037 | Sapur.016G227700 |
| Potri.008G076400 | EVM0006689 | EVM0051919 | Sapur.016G191900 |
| Potri.008G117100 | EVM0043216 | EVM0044977 | Sapur.016G190500 |
| Potri.008G071100 | EVM0049616 | EVM0054100 | Sapur.016G255700 |
| Potri.008G073600 | EVM0055887 | EVM0015962 | Sapur.016G015300 |
| Potri.008G076400 | EVM0022735 | EVM0033385 | Sapur.016G108900 |
| Potri.008G166000 | EVM0012743 | EVM0039499 | Sapur.002G159300 |
| Potri.008G166100 | EVM0036069 | EVM0000005 | Sapur.002G115400 |
| Potri.008G166200 | EVM0045763 | EVM0023642 | Sapur.002G100900 |
| Potri.008G011900 | EVM0027097 | EVM0043533 | Sapur.002G092900 |
| Potri.008G186300 | EVM0019343 | EVM0049776 | Sapur.002G075900 |
| Potri.008G117100 | EVM0003611 | EVM0037879 | Sapur.002G068300 |
| Potri.008G120100 | EVM0042153 | EVM0023217 | Sapur.002G051800 |
| Potri.008G210900 | EVM0034491 | EVM0027771 | Sapur.002G030300 |
| Potri.008G045300 | EVM0039575 | EVM0014312 | Sapur.002G030200 |
| Potri.008G166100 | EVM0012873 | EVM0040250 | Sapur.002G030100 |
| Potri.008G215600 | EVM0046023 | EVM0023703 | Sapur.002G030000 |
| Potri.008G076400 | EVM0004062 | EVM0008628 | Sapur.002G143500 |
| Potri.008G117100 | EVM0039609 | EVM0054355 | Sapur.002G147200 |
| Potri.008G166000 | EVM0049532 | EVM0014312 | Sapur.005G177600 |
| Potri.008G166100 | EVM0002921 | EVM0037879 | Sapur.005G137400 |
| Potri.008G071100 | EVM0032156 | EVM0054355 | Sapur.014G073200 |
| Potri.008G073600 | EVM0035890 | EVM0004107 | Sapur.018G023800 |
| Potri.008G076400 | EVM0007616 | EVM0002257 | Sapur.018G031200 |
| Potri.009G101900 | EVM0011459 | EVM0034096 | Sapur.018G034800 |
| Potri.009G147700 | EVM0044116 | EVM0027008 | Sapur.018G036500 |
| Potri.009G147700 | EVM0052580 | EVM0028452 | Sapur.018G062500 |
| Potri.009G101900 | EVM0002769 | EVM0025980 | Sapur.018G067800 |
| Potri.009G101900 | EVM0043678 | EVM0057138 | Sapur.018G102800 |
| Potri.010G181000 | EVM0006689 | EVM0004204 | Sapur.004G031600 |
| Potri.010G183700 | EVM0018103 | EVM0049898 | Sapur.004G031700 |
| Potri.010G186400 | EVM0025370 | EVM0012041 | Sapur.004G035000 |
| Potri.010G092800 | EVM0044041 | EVM0028403 | Sapur.004G035100 |
| Potri.010G247200 | EVM0023967 | EVM0043678 | Sapur.004G089000 |
| Potri.010G129200 | EVM0043216 | EVM0054684 | Sapur.014G101000 |
| Potri.010G006800 | EVM0020075 | EVM0007023 | Sapur.014G042400 |
| Potri.010G046600 | EVM0054855 | EVM0031305 | Sapur.014G035600 |
| Potri.010G181000 | EVM0022735 | EVM0049893 | Sapur.014G014900 |
| Potri.010G183700 | EVM0055887 | EVM0011531 | Sapur.014G004000 |
| Potri.010G072300 | EVM0045763 | EVM0054439 | Sapur.002G030200 |
| Potri.010G072400 | EVM0036069 | EVM0008755 | Sapur.005G177800 |
| Potri.010G072600 | EVM0012743 | EVM0001248 | Sapur.005G177700 |
| Potri.010G125600 | EVM0042153 | EVM0054439 | Sapur.005G177600 |
| Potri.010G129200 | EVM0003611 | EVM0004610 | Sapur.005G068900 |
| Potri.010G072400 | EVM0012873 | EVM0043629 | Sapur.005G060200 |
| Potri.010G216200 | EVM0039575 | EVM0024690 | Sapur.005G137400 |
| Potri.010G247200 | EVM0036581 | EVM0003769 | Sapur.005G130300 |
| Potri.010G181000 | EVM0004062 | EVM0043629 | Sapur.007G082500 |
| Potri.010G046600 | EVM0055399 | EVM0046201 | Sapur.007G043700 |
| Potri.010G181000 | EVM0007616 | EVM0056893 | Sapur.018G102800 |
| Potri.010G183700 | EVM0035890 |  |  |
| Potri.010G186400 | EVM0032156 |  |  |
| Potri.010G072400 | EVM0002921 |  |  |
| Potri.010G072600 | EVM0049532 |  |  |
| Potri.010G006800 | EVM0044386 |  |  |
| Potri.010G129200 | EVM0039609 |  |  |
| Potri.011G061700 | EVM0005349 |  |  |
| Potri.011G115600 | EVM0053305 |  |  |
| Potri.011G115600 | EVM0006682 |  |  |
| Potri.011G056900 | EVM0049397 |  |  |
| Potri.011G057000 | EVM0052258 |  |  |
| Potri.011G061800 | EVM0045883 |  |  |
| Potri.011G056900 | EVM0001371 |  |  |
| Potri.011G057000 | EVM0048064 |  |  |
| Potri.011G061700 | EVM0035055 |  |  |
| Potri.011G115600 | EVM0041629 |  |  |
| Potri.011G115600 | EVM0054100 |  |  |
| Potri.011G056900 | EVM0004204 |  |  |
| Potri.011G057000 | EVM0049898 |  |  |
| Potri.011G061700 | EVM0012041 |  |  |
| Potri.011G061800 | EVM0028403 |  |  |
| Potri.012G134100 | EVM0009316 |  |  |
| Potri.012G032900 | EVM0022896 |  |  |
| Potri.012G108500 | EVM0013502 |  |  |
| Potri.012G134000 | EVM0010374 |  |  |
| Potri.012G134100 | EVM0032370 |  |  |
| Potri.012G108500 | EVM0031724 |  |  |
| Potri.012G134000 | EVM0044087 |  |  |
| Potri.012G134100 | EVM0014688 |  |  |
| Potri.012G032900 | EVM0049667 |  |  |
| Potri.013G056700 | EVM0021312 |  |  |
| Potri.013G158500 | EVM0043703 |  |  |
| Potri.013G045200 | EVM0045225 |  |  |
| Potri.013G135600 | EVM0055401 |  |  |
| Potri.013G100300 | EVM0001548 |  |  |
| Potri.013G101100 | EVM0016569 |  |  |
| Potri.013G056700 | EVM0030823 |  |  |
| Potri.013G045200 | EVM0022031 |  |  |
| Potri.013G056700 | EVM0024408 |  |  |
| Potri.013G100300 | EVM0022064 |  |  |
| Potri.013G101100 | EVM0005180 |  |  |
| Potri.013G101200 | EVM0050604 |  |  |
| Potri.013G135600 | EVM0025018 |  |  |
| Potri.013G101100 | EVM0025179 |  |  |
| Potri.013G101200 | EVM0006866 |  |  |
| Potri.014G008100 | EVM0050425 |  |  |
| Potri.014G008100 | EVM0038265 |  |  |
| Potri.014G012200 | EVM0038265 |  |  |
| Potri.014G025200 | EVM0053591 |  |  |
| Potri.014G099900 | EVM0031973 |  |  |
| Potri.014G126100 | EVM0028978 |  |  |
| Potri.014G076701 | EVM0027493 |  |  |
| Potri.014G055700 | EVM0005644 |  |  |
| Potri.014G025200 | EVM0029410 |  |  |
| Potri.014G126100 | EVM0017324 |  |  |
| Potri.014G047000 | EVM0052610 |  |  |
| Potri.014G055700 | EVM0055308 |  |  |
| Potri.014G094500 | EVM0054355 |  |  |
| Potri.014G099900 | EVM0008628 |  |  |
| Potri.014G025200 | EVM0023642 |  |  |
| Potri.014G055700 | EVM0000005 |  |  |
| Potri.014G126100 | EVM0039499 |  |  |
| Potri.014G008100 | EVM0011531 |  |  |
| Potri.014G012200 | EVM0011531 |  |  |
| Potri.014G025200 | EVM0049893 |  |  |
| Potri.014G046600 | EVM0031305 |  |  |
| Potri.014G046700 | EVM0017641 |  |  |
| Potri.014G055700 | EVM0007023 |  |  |
| Potri.014G126100 | EVM0054684 |  |  |
| Potri.015G136300 | EVM0006672 |  |  |
| Potri.015G136400 | EVM0009316 |  |  |
| Potri.015G136400 | EVM0032370 |  |  |
| Potri.015G023200 | EVM0022896 |  |  |
| Potri.015G136400 | EVM0014688 |  |  |
| Potri.015G023200 | EVM0049667 |  |  |
| Potri.016G056400 | EVM0015665 |  |  |
| Potri.016G084500 | EVM0000418 |  |  |
| Potri.016G053200 | EVM0045295 |  |  |
| Potri.016G056400 | EVM0011412 |  |  |
| Potri.016G018600 | EVM0021255 |  |  |
| Potri.016G056400 | EVM0035539 |  |  |
| Potri.016G084500 | EVM0020564 |  |  |
| Potri.016G018600 | EVM0015962 |  |  |
| Potri.016G126100 | EVM0033385 |  |  |
| Potri.017G055400 | EVM0021270 |  |  |
| Potri.017G013700 | EVM0045686 |  |  |
| Potri.017G013700 | EVM0029866 |  |  |
| Potri.017G087800 | EVM0051961 |  |  |
| Potri.017G013700 | EVM0023162 |  |  |
| Potri.017G053700 | EVM0009817 |  |  |
| Potri.017G053700 | EVM0044977 |  |  |
| Potri.018G091600 | EVM0033580 |  |  |
| Potri.018G131400 | EVM0056869 |  |  |
| Potri.018G021900 | EVM0009928 |  |  |
| Potri.018G047300 | EVM0016157 |  |  |
| Potri.018G021900 | EVM0019845 |  |  |
| Potri.018G028000 | EVM0045980 |  |  |
| Potri.018G038100 | EVM0013555 |  |  |
| Potri.018G043900 | EVM0053799 |  |  |
| Potri.018G047300 | EVM0018678 |  |  |
| Potri.018G085700 | EVM0040155 |  |  |
| Potri.018G091600 | EVM0043933 |  |  |
| Potri.018G102200 | EVM0037464 |  |  |
| Potri.018G021900 | EVM0017041 |  |  |
| Potri.018G028000 | EVM0019323 |  |  |
| Potri.018G028000 | EVM0008598 |  |  |
| Potri.018G091600 | EVM0006637 |  |  |
| Potri.018G131400 | EVM0037206 |  |  |
| Potri.018G028000 | EVM0004107 |  |  |
| Potri.018G038100 | EVM0002257 |  |  |
| Potri.018G043900 | EVM0034096 |  |  |
| Potri.018G047300 | EVM0027008 |  |  |
| Potri.018G131400 | EVM0057138 |  |  |
| Potri.018G085700 | EVM0028452 |  |  |
| Potri.018G091600 | EVM0025980 |  |  |
| Potri.018G131400 | EVM0056893 |  |  |
| Potri.019G036100 | EVM0021312 |  |  |
| Potri.019G073300 | EVM0001548 |  |  |
| Potri.019G075500 | EVM0016569 |  |  |
| Potri.019G102200 | EVM0055401 |  |  |
| Potri.019G088000 | EVM0043720 |  |  |
| Potri.019G131300 | EVM0012647 |  |  |
| Potri.019G073300 | EVM0022064 |  |  |
| Potri.019G075500 | EVM0005180 |  |  |
| Potri.019G075600 | EVM0050604 |  |  |
| Potri.019G036100 | EVM0024408 |  |  |
| Potri.019G102200 | EVM0028773 |  |  |
| Potri.019G067400 | EVM0044050 |  |  |
| Potri.019G075500 | EVM0025179 |  |  |
| Potri.019G075600 | EVM0006866 |  |  |
| Potri.019G102200 | EVM0025018 |  |  |

**Table S8 The DEGs names of SmAP2/ERF**

| EVM0025980 | SmAP2-3 | EVM0000535 | SmERF B1-34 |
| --- | --- | --- | --- |
| EVM0011531 | SmAP2-4 | EVM0046939 | SmERF B1-37 |
| EVM0036052 | SmAP2-15 | EVM0008900 | SmERF B1-38 |
| EVM0043933 | SmAP2-33 | EVM0054684 | SmERF B2-3 |
| EVM0028655 | SmAP2-38 | EVM0028978 | SmERF B2-6 |
| EVM0052580 | SmDREB A1-1 | EVM0017324 | SmERF B2-8 |
| EVM0013573 | SmDREB A1-10 | EVM0001248 | SmERF B3-10 |
| EVM0040623 | SmDREB A1-4 | EVM0006603 | SmERF B3-14 |
| EVM0044116 | SmDREB A1-6 | EVM0006649 | SmERF B3-15 |
| EVM0032370 | SmDREB A1-7 | EVM0055475 | SmERF B3-17 |
| EVM0014688 | SmDREB A1-9 | EVM0035905 | SmERF B3-19 |
| EVM0042752 | SmDREB A2-29 | EVM0024412 | SmERF B3-29 |
| EVM0018103 | SmDREB A2-9 | EVM0040250 | SmERF B3-3 |
| EVM0057013 | SmDREB A4-10 | EVM0022031 | SmERF B3-34 |
| EVM0006866 | SmDREB A4-23 | EVM0008128 | SmERF B3-37 |
| EVM0040155 | SmDREB A4-24 | EVM0054964 | SmERF B3-42 |
| EVM0053799 | SmDREB A4-25 | EVM0052610 | SmERF B3-45 |
| EVM0005644 | SmDREB A4-27 | EVM0053966 | SmERF B3-46 |
| EVM0010374 | SmDREB A4-28 | EVM0012041 | SmERF B3-5 |
| EVM0018598 | SmDREB A4-31 | EVM0020531 | SmERF B3-52 |
| EVM0032021 | SmDREB A4-34 | EVM0037606 | SmERF B3-53 |
| EVM0044087 | SmDREB A4-35 | EVM0009409 | SmERF B3-57 |
| EVM0004316 | SmDREB A4-39 | EVM0038648 | SmERF B3-58 |
| EVM0049295 | SmDREB A5-13 | EVM0002769 | SmERF B4-1 |
| EVM0037879 | SmDREB A5-2 | EVM0011459 | SmERF B4-13 |
| EVM0003861 | SmDREB A5-23 | EVM0000045 | SmERF B4-19 |
| EVM0001082 | SmDREB A5-26 | EVM0004610 | SmERF B4-4 |
| EVM0046201 | SmDREB A5-7 | EVM0047172 | SmERF B4-7 |
| EVM0015466 | SmDREB A6-12 | EVM0044712 | SmERF B5-14 |
| EVM0014537 | SmDREB A6-17 | EVM0016170 | SmERF B6-17 |
| EVM0051729 | SmDREB A6-20 | EVM0030823 | SmERF B6-2 |
| EVM0000460 | SmDREB A6-6 | EVM0003611 | SmRAV-4 |
| EVM0029866 | SmERF B1-12 | EVM0039609 | SmRAV-5 |
| EVM0048064 | SmERF B1-30 | EVM0033748 | SmRAV-6 |

**Figure S1 Alignment of 412 AP2 domains**

To better classify these SmAP2 genes, 48 AP2 domains from known categories of Arabidopsis and Populus trichocarpa AP2 genes were selected to carry out multiple sequence alignment with AP2 domains of SmAP2/ERF proteins using ClustalW in Website https://npsa-prabi.ibcp.fr/cgi-bin/npsa_automat.pl?page=/NPSA/npsa_clustalw.html

**Figure S2 Conserved motifs in proteins of SmAP2/ERF superfamily**

The online tool MEME (http://meme-suite.org/tools/meme) was used to search for conserved motifs of SmAP2/ERF superfamily proteins. The optimized parameters were employed as follows: any number of repetitions, maximum number of motifs = 10, and the optimum width of each motif was 6–50 residues.

**Figure S3 The divergence time (T Value) of gene pairs** **from** **two groups.**

The divergence time (T Value) of gene pairs can be classified mainly into two groups, a group and b group with two time period, 2–8 Mya (average value, 5Mya ) and 20–36 Mya (average value, 26 Mya) respectively.
