# Supplementary material for: Genome-wide investigation of the AP2/ERF superfamily and their expression under salt stress in Chinese willow (Salix matsudana)
Source: PeerJ. 2021 Apr 13;9:e11076. doi: 10.7717/peerj.11076 (PMC8051338; doi:10.7717/peerj.11076)
Supplement: Supplemental Information 2 — To better classify these SmAP2 genes, 48 AP2 domains from known categories of Arabidopsis and Populus trichocarpa AP2 genes were selected to carry out multiple sequence alignment with AP2 domains of SmAP2/ERF proteins using ClustalW in Website https://npsa-prabi.ibcp.fr/cgi-bin/npsa_automat.pl?page=/NPSA/npsa_clustalw.html. [file peerj-09-11076-s002.pdf]

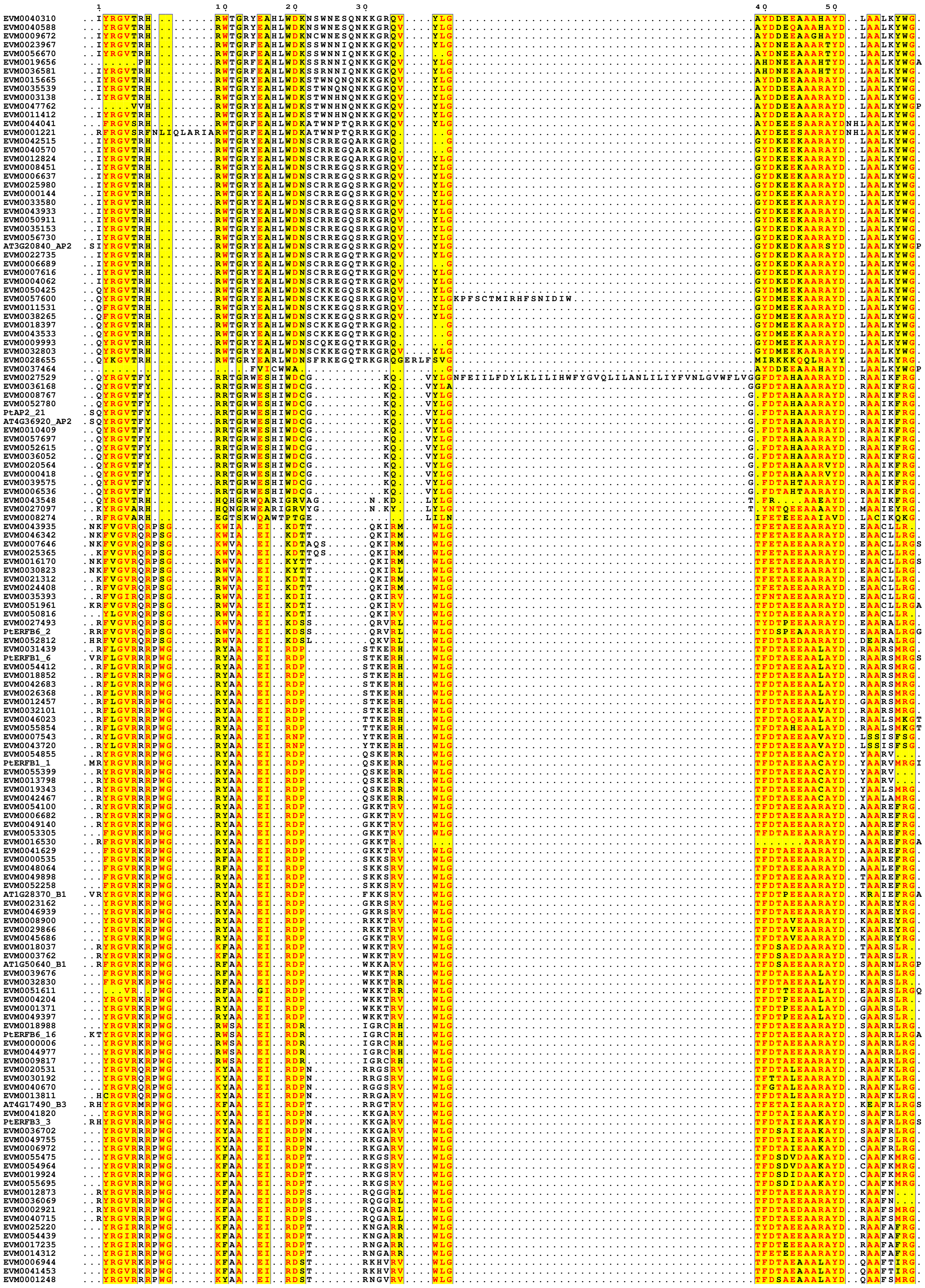

1
10
20
30
40
50

```
EVM0008128   ...YRGVRRRPWG......KYAA..EI..RDST.......RNGVRV....WLG...........................................TFDSAEAAALAYD..QAAFSMRG.
EVM0040250   ...YRGVRRRPWG......KYAA..EI..RDST.......RNGVRV....WLG...........................................TFDSAEAAALAYD..QAAFSMRG.
EVM0035905   ...YRGVRRRPWG......KYAA..EI..RDST.......RNGIRV....WLG...........................................TFDSAEAAALAYD..QAAFSMRG.
EVM0025231   ...YRGVRRRPWG......KFAA..EI..RDST.......RNGRRV....WLG...........................................TFDSAEAAALAYD..QAAFSMRG.
EVM0017086   ...YRGVRRRPWG......KFAA..EI..RDST.......RNGIRV....WLG...........................................TFDSAEAAALAYD..QAAFSMRG.
EVM0045763   ...YRGVRRRPWG......KFAA..EI..RDST.......RHGVRV....WLG...........................................TFDSAEAAALAYD..QAAFSMRG.
EVM0038648   ...YRGVRRRPWG......KFAA..EI..RDST.......RHGVRV....WLG...........................................TFDSAEAAALAYD..QAAFSMRG.
EVM0022031   ...YRGVRRRPWG......KYAA..EI..RDST.......RNGVRV....WLG...........................................TFDTAEAAALAYD..QAAFAMRG.
EVM0045225   ...YRGVRRRPWG......KYAA..EI..RDST.......RNGVRV....WLG...........................................TFDTAEAAALAYD..QAAFAMRG.
EVM0045646   ...YRGVRKRPWG......KYAA..EI..RDST.......RNGVRV....WLG...........................................TFDTGEAAALAYD..QAALSLHG.
EVM0008755   ...YRGVRKRPWG......KYAA..EI..RDST.......RNGVRV....WLG...........................................TFDTGEAAALAYD..QAALSLHG.
EVM0018983   ...YRGVRNRPWG......TYAA..EI..RDST.......RNGVRV....WLG...........................................TFDTGEAAALAYD..QAALSLQG.
EVM0023703   ...YRGVRNRPWG......TYAA..EI..RDST.......RNGARV....WLG...........................................TFDTGEAAALAYD..QAALSLQG.
AT2G31230_B3 .RSYRGVRKRPWG......KFAA..EI..RDST.......RNGIRV....WLG...........................................TFDKAEEAALAYD..QAAFATKGS
EVM0049532   ..KYRGVRRRPWG......KFAA..EI..RDST.......RHGARV....WLG...........................................TFNTAEEAARAYD..RAAYAMRG.
EVM0029622   ..KYRGVRRRPWG......KFAA..EI..RDSA.......RHGARV....WLG...........................................TFNTAEEAARAYD..RAAYAMRG.
EVM0012743   ..KYRGVRKRPWG......KFAS..EI..RDST.......RHGARV....WLG...........................................TFNTAEEAARAYD..RAAYAMRG.
EVM0009409   ..KYRGVRKRPWG......KFAA..EI..RDST.......RHGARM....WLG...........................................TFNTAEEAARAYD..RAAYAMRG.
EVM0052364   ..RYRGVRRRPWG......KFAA..EI..RDST.......RHGARL....WLG...........................................TFNTAEEAARAYD..RAAYSMRG.
EVM0027771   ..RYRGVRRRPWG......KFAA..EI..RDSA.......RHGARL....WLG...........................................TFNTAEEAARAYD..RAAYSMRG.
EVM0005349   ...YRGVRRRPWG......KYAA..EI..RDST.......RHGARI....WLG...........................................TFETAEAAALAYD..RAAFNMRG.
EVM0035055   ...YRGVRRRPWG......KYAA..EI..RDST.......RHGARI....WLG...........................................TFETAEAAALAYD..RAAFNMRG.
EVM0012041   ...YRGVRRRPWG......KYAA..EI..RDSR.......RRGARI....WLG...........................................TFETAEEAALAYD..RAAFNMRG.
EVM0028403   ...YRGVRRRPWG......KYAA..EI..RDSS.......RKGARA....WLG...........................................TFETAEEAALAYD..KAALRIRG.
EVM0052605   ...YRGVRRRPWG......KYAA..EI..RDSS.......RKGARA....WLG...........................................TFETAEEAAMAYD..KAALRIRG.
EVM0045883   ...YRGVRRRPWG......KYAA..EI..RDSS.......KKGARA....WLG...........................................TFETAEEAALAYD..KAALRIRG.
EVM0037606   ...YRGVRQRPWG......KFAA..EI..RDPA.......KNGARV....WLG...........................................TFETAEDAALAYD..RAAYRMRG.
PtERFB3_12   .KHYRGVRQRPWG......KFAA..EI..RDPA.......KNGARV....WLG...........................................TFETAEDAALAYD..RAAYRMRGS
EVM0054822   ...YRGVRQRPWG......KFAA..EI..RDPA.......KNGARV....WLG...........................................TFETAEDAALAYD..RAAYRMRG.
EVM0024412   ...YRGVRQRPWG......KFAA..EI..RDPA.......KNGARV....WLG...........................................TFETAEDAALAYD..RAAYRMRG.
EVM0030239   ...YRGVRQRPWG......KFAA..EI..RDPA.......KNGARV....WLG...........................................TFATAEDAALAYD..RAAYRMRG.
EVM0046641   ...YRGVRQRPWG......KFAA..ET..RDPA.......KNGARV....WLG...........................................TYETAKEAALAYD..RAAYRMRG.
EVM0006603   ...YRGVRQRPWG......KFAA..ET..RDPA.......KNGARV....WLG...........................................TYETAKEAALAYD..RAAYRMRG.
EVM0006649   ...YRGVRQRPWG......KFAA..EI..RDPA.......KNGARV....WLG...........................................TYETAEEAALAYD..RAAYRMRG.
EVM0054070   ...YRGVRQRPWG......KFAA..EI..RDPA.......KNGARL....WLG...........................................TYETAEEAALAYD..RAAYRMRG.
EVM0052610   ..QYRGVRRRPWG......KYAA..EI..RDPK.......KNGARM....WLG...........................................TYETPEDAALAYD..QAAFKIRG.
EVM0057308   ..QYRGVRRRHWG......KYAA..EI..RDPK.......KNGARM....WLG...........................................TYETPEDAALAYD..QAAFKIRG.
EVM0053966   ..QYKGVRRRPWG......KYAA..EI..RDPK.......KIGARM....WLG...........................................TYEKPEDAALAYD..RAAFKMRG.
EVM0031305   ..QYKGVRRRPWG......KYAA..EI..SDPN.......KIGARM....WLG...........................................TYEKPEDAALAYD..RAAFKMRG.
EVM0017641   ..SYKGVRRRPGG......KYAA..EI..RYPM.......KNGARQ....WLG...........................................TYETPEDAAVAYD..LAAFKMRG.
EVM0028978   ...YRGIRQRPWG......KWAA..EI..RDP........RKGVRV....WLG...........................................TFNTAEEAARAYD..REARKIRG.
EVM0054684   ...YRGIRQRPWG......KWAA..EI..RDP........RKGVRV....WLG...........................................TFNTAEEAARAYD..REARKIRG.
EVM0017324   ...YRGIRQRPWG......KWAA..EI..RDP........RKGVRV....WLG...........................................TFNTAEEAARAYD..REA......
EVM0023232   ..QFRGIRQRPWG......KWAA..EI..RDP........RKGVRV....WLG...........................................TFNTAEEAARAYD..AEARRIRG.
EVM0055941   ..QFRGIRQRPWG......KWAA..EI..RDP........RKGVRV....WLG...........................................TFNTAEEAARAYD..AEARRIRG.
EVM0044966   ..QYRGIRQRPWG......KWAA..EI..RDP........RKGVRV....WLG...........................................TFNTAEEAARAYD..VEARRIRG.
EVM0039499   ...YRGIRQRPWG......KWAA..EI..RDP........RKGVRV....WLG...........................................TFNTAEEAARAYD..REA......
AT3G16770_B2 .NVYRGIRKRPWG......KWAA..EI..RDP........RKGVRV....WLG...........................................TFNTAEEAAMAYD..VAAKQIRGD
AT2G47520_B2 .NLYRGIRQRPWG......KWAA..EI..RDP........SKGVRV....WLG...........................................TFKTADEAARAYD..VAAIKIRGR
EVM0044386   ...YRGIRQRPWG......KWAA..EI..RDP........HKGVRV....WLG...........................................TYNTAEEAAKAYD..EAAKRIRG.
EVM0020075   ...YRGIRQRPWG......KWAA..EI..RDP........HKGVRV....WLG...........................................TYNTAEEAAKAYD..EAAKRIRG.
EVM0034491   ...YRGIRQRSWG......KWAS..EI..RDP........HKGARV....WLG...........................................TYNTAEEAAKAYD..EAAKRIRG.
EVM0026277   ...YRGVRQRPWG......KWAA..EI..RDP........KKAARV....WLG...........................................TFDTAEDAAVAYD..KAALKFKG.
PtERFB4_1    .RHYRGVRQRPWG......KWAA..EI..RDP........KKAARV....WLG...........................................TFDTAEDAAVAYD..KAALKFKGT
EVM0017998   ...YRGVRQRPWG......KWAA..EI..RDP........KKAARV....WLG...........................................TFDTAEDAAVAYD..KAALKFKG.
EVM0040725   ...YRGVRQRPWG......KWAA..EI..RDP........KKAARV....WLG...........................................TFDTAEDAAVAYD..KAALKFKG.
EVM0032029   RKALQRSETKTLG......KWAA..EI..RDP........KKAARV....WLG...........................................TFDTAEDAAVAYD..KAALKFKG.
EVM0013578   ...YRGVRQRPWG......KWAA..EI..RDP........KKAARV....WLG...........................................TFDTAEDAAAAYD..KAALKFKG.
EVM0013502   ...YRGVRQRPWG......KWAA..EI..RDP........KKAARV....WLG...........................................TFETAEAAAFAYD..EAALRFKG.
EVM0031724   ...YRGVRQRPWG......KWAA..EI..RDP........KKASRV....WLG...........................................TFETAEAAALAYD..EAALRFKG.
AT5G61890_B4 .RHYRGVRQRPWG......KWAA..EI..RDP........KKAARV....WLG...........................................TFETAESAALAYD..EAALKFKGS
EVM0023217   ..RYRGVRQRPWG......KWAA..EI..RDP........HKAARV....WLG...........................................TFDTAEAAARAYD..EAALRFRG.
EVM0030954   ..RYRGVRQRPWG......KWAA..EI..RDP........HKAARV....WLG...........................................TFDTAEAAARAYD..EAALRFRG.
EVM0047172   ..RYRGVRQRPWG......KWAA..EI..RDP........HKAARV....WLG...........................................TFETAEAAARAYD..EAALRFRG.
EVM0000045   ..RYRGVRQRPWG......KWAA..EI..RDP........HKAARV....WLG...........................................TFETAEAAARAYD..EAALRFRG.
EVM0004610   ..KYRGVRQRPWG......KWAA..EI..RDP........FKAARV....WLG...........................................TFDTAEAAARAYD..EAALRFRG.
EVM0001106   ..KYRGVRQRPWG......KWAA..EI..RDP........VKAARV....WLG...........................................TFDTAEAAARAYD..EAALRFRG.
EVM0002769   ...YRGVRQRPWG......KWAA..EI..RDP........RKAARV....WLG...........................................TFNTAEEAARAYD..KAAIDFRG.
EVM0011459   ...YRGVRQRPWG......KWAA..EI..RDP........RKAARV....WLG...........................................TFNTAEEAARAYD..KAAIDFRG.
EVM0043678   ...YRGVRQRPWG......KWAA..EI..RDP........LRAARV....WLG...........................................TFTTAEEAARAYD..KAAIGFRG.
AT2G33710_B4 .RNYRGVRQRPWG......KWAA..EI..RDP........NKAARV....WLG...........................................TFDTAEEAALAYD..KAAFEFRGH
EVM0051566   ...YRGVRQRPWG......KWAA..EI..RNP........TQRNRV....WLG...........................................TFENAEDAARAYD..MKNIEFRG.
EVM0057361   ..KYRGVRQRPWG......KWAA..EI..SDP........AQGTRV....WLG...........................................TFENAEDAARAYD..MKNIEFRG.
EVM0015843   ..KYRGVRLRPWG......KWAA..EI..RDP........ARASRV....WLG...........................................TFENAEDAARAYD..TKNIEFRG.
EVM0034620   ...FRGVRQRPWG......KWAA..EI..RDP........LRRVRL....WLG...........................................TYDTAEEAAMVYD..NAAIQLRG.
EVM0031168   ..KFRGVRQRPWG......KWAA..EI..RDP........LRRVRL....WLG...........................................TYDTAEEAAMVYD..NAAIQLRG.
EVM0024472   ..KFRGVRQRPWG......KWAA..EI..RDP........LRRVRL....WLG...........................................TYETAEEAAMVYD..NAAIQLRG.
EVM0045726   ...FRGVRQRPWG......KWAA..EI..RDP........SRRVRL....WLG...........................................TYDTAEEAAMVYD..NAAIQLRG.
EVM0022896   ...FRGVRQRPWG......KWAA..EI..RDP........ARRVRL....WLG...........................................TYDTAEEAARVYD..NAAIKLRG.
PtERFB5_5    .RKFRGVRQRPWG......KWAA..EI..RDP........ARRVRL....WLG...........................................TYDTAEEAARVYD..NAAIKLRGP
EVM0049667   ...FRGVRQRPWG......KWAA..EI..RDP........TKKQRL....WLG...........................................TYDTAEEAARVYD..NAAIKLRG.
AT4G27950_B5 .KKYRGVRQRPWG......KWAA..EI..RDP........EQRRRI....WLG...........................................TFATAEEAAIVYD..NAAIKLRGP
EVM0012647   ...FRGVRQRPWG......KWAA..EI..RDP........TRQKRV....WLG...........................................TFNTAEEAATVYD..RAAVKLKG.
EVM0015330   ...FRGVRQRPWG......KWAA..EI..CDP........TRRKRV....WLG...........................................TFSTAEEAATVYD..RAAVELKG.
PtERFB5_7    .KKFRGVRQRPWG......KWSA..EI..RDP........TRRKRV....WLG...........................................TFDTAEEAATVYD..RAALKLKGP
EVM0043703   ...FRGVRQRPWG......KWAA..EI..RDP........TQRKRV....WLG...........................................TFDTAEEAATVYD..RAAVRLKG.
EVM0017733   ...FRGVRQRPWG......KWAA..EI..RDP........NRQKRV....WLG...........................................TFDTAEEAATVYD..RAAVRLKG.
EVM0001609   ...FRGVRQRPWG......KWAA..EI..RDP........NRQKRV....WLG...........................................TFDTAEEAATVYD..RAAVRLKG.
EVM0054355   ..RYRGVRQRPWG......RWAA..EI..RDP........YRKTRL....WLG...........................................TFDTAEEAAMVYD..QAAIRIKG.
EVM0044712   ..RYRGVRQRPWG......RWAA..EI..RDP........YRRARL....WLG...........................................TYDTAEEAAMAYD..QAAIRIKG.
EVM0026052   ...YY....YPEGK.....KWAA..EI..RDP........YRRTRV....WLG...........................................TYDTAEEAAMVYD..EAAIRIKGP
EVM0041252   ..KYRGVRQRPWG......RWAA..EI..RDP........YKRTRV....WLG...........................................TYDTAEEAAMVYD..EAAIRIKG.
AT2G46310_B5 .VKYRGVRQRPWG......KFAA..EI..RDP........SSRTRL....WLG...........................................TFATAEEAAIGYD..RAAIRIKGH
EVM0013188   ..SYRGVRMRHWG......KWVS..EI..RQP........KKKSRI....WLG...........................................TFPTPEMAARAHD..VAALTIKG.
EVM0015672   ..SYRGVRMRQWG......KWVS..EI..RQP........KKKSRI....WLG...........................................TFPTAEMAARAHD..VAALTIKG.
EVM0051058   ...YRGVRMRQWG......KWVS..EI..RQP........KKKSRI....WLG...........................................TFSTAEMAARAHD..VAALTIKG.
EVM0018299   ...YRGVRMRQWG......KWVS..EI..RQP........KKKSRI....WLG...........................................SFSTEEMAARAHD..VAALTIKG.
AT3G16280_A4 .PVYRGVRMRSWG......KWVS..EI..RQP........RKKTRI....WLG...........................................TFVTADMAARAHD..VAALTIKGS
EVM0040155   .PVFRGVRMRTWG......KWVS..EI..REP........RKKNRI....WLG...........................................TFSTPEMAARAHD..VAALSIKG.
EVM0028452   .PVFRGVRMRTWG......KWVS..EI..REP........RKKNRI....WLG...........................................TFSTPEMAARAHD..VAALSIKGK
EVM0034096   ..VYRGVRMRNWG......KWVS..EI..REP........RKKSRI....WLG...........................................TFPTPEMAARAHD..VAALSIKG.
EVM0053799   ........MRNWG......KWVS..EI..REP........RKKSRI....WLG...........................................TFPTPEMAARAHD..VAALSIKGK
EVM0032021   ..VYRGVRMRNWG......KWVS..EI..REP........RKKSRI....WLG...........................................TFPTPEKAARAHD..AAALSIKG.
PtDREBA4_17  .PVYRGVRMRNWG......KWVS..EI..REP........RKKSRI....WLG...........................................TFPTPEKAARAHD..AAALSIKGN
EVM0042411   ..IYRGVRMRNWG......KWVS..EI..REP........RKKSRI....WLG...........................................TFPTPEKAARAHD..AAALSIKG.
EVM0021344   .AVYRGVRKRAWG......KWVS..EI..RQP........RKKSRI....WLG...........................................TFPTPEMAARAHD..VAALSIKGS
EVM0018160   .AVYRGVRKRAWG......KWVS..EI..RQP........RKKSRI....WLG...........................................TFPTPEMAARAHD..VAALSIKGN
EVM0001696   .PVYTGVRRRAWG......KWVS..EI..RQP........RKKSRI....WLG...........................................TFPTPEMAARAHD..VAALSIKGD
EVM0005032   .PVYTGVRRRAWG......KWVS..EI..RQP........RKKSRI....WLG...........................................TFPAPEMAARAHD..VAALSIKGD
EVM0055308   ..TYRGVRMRSWG......KWVC..EI..REP........RKKSRI....WLG...........................................TYPTAEMAARAHD..VAALAIKG.
EVM0007023   ..TYRGVRMRSWG......KWVC..EI..REP........RKKSRI....WLG...........................................TYPTAEMAARAHD..VAALAIKG.
EVM0005644   ..TYRGVRMRSWG......KWVS..EI..REP........RKKSRI....WLG...........................................TYPTAEMAARAHD..VAALAIKG.
EVM0000005   ..TYRGVRMRSWG......KWVS..EI..REP........RKKSRI....WLG...........................................TYPAAEMAARAHD..VAALAIKG.
EVM0029808   .PVFRGTRKRNGD......KWVC..EM..REP........NKKSRI....WLG...........................................TYPTPEMAARAHD..VAALALRGK
EVM0004638   .PVFRGTRKRNGN......KWVC..EM..REP........NKKSRI....WLG...........................................TYPTPEMAARAHD..VAALALRGK
EVM0026062   .PVFRGVRKRNGN......KW.....................SRI....WLG...........................................TYPTPEMAARAHD..VAALALRGK
EVM0040623   .PIYRGVRKRNGD......KWVC..EL..REP........NKKSRI....WLG...........................................TYPTPEMAARAHD..VAALAFRG.
PtDREBA1_3   .PIFRGVRKRNGD......KWVC..EL..REP........NKKSRI....WLG...........................................TYPTPEMAARAHD..VAALAFRGK
EVM0052580   .PVYRGIRRRNGN......KWVC..EM..REP........NKKSRI....WVG...........................................TFKSPEMAARAHD..VAALALKGE
EVM0044116   .PVYRGVRRRNGN......KWVC..EV..REP........NKKSRI....WVG...........................................TFKSPEMAARAYD..VAALALKGE
EVM0009316   ..VYRGVRRRNSG......KWVC..EV..REP........NKKSRI....WLG...........................................TFPTADMAARAHD..VAALALRG.
EVM0013573   ..VYRGVRRRNSG......KWVC..EV..REP........NKKSRI....WLG...........................................TFPTADMAARAHD..VAALALRG.
EVM0014688   .PVFRGVRRRNSG......KWVC..EV..REP........NKKSRI....WLG...........................................TFPIAEMAARAHD..VAALALRGR
EVM0032370   .PVFRGVRRRNSG......KWVC..EV..REP........NKKSRI....WLG...........................................TFPIAEMAARAHD..VAALALRGR
AT4G25480_A1 .PIYRGVRRRNSG......KWVC..EV..REP........NKKTRI....WLG...........................................TFQTAEMAARAHD..VAALALRGR
AT4G25490_A1 .PIYRGVRQRNSG......KWVS..EV..REP........NKKTRI....WLG...........................................TFQTAEMAARAHD..VAALALRGR
EVM0018598   .PVYRGIRSRS.G......KWVS..EI..REP........RKTTRI....WLG...........................................TFPNPEMAAAAYD..VAVLALKGA
EVM0057013   .PVYRGIRSRS.G......KWVS..EI..REP........RKTTRI....WLG...........................................TFPEPEMAAAAYD..VAVLALRGA
EVM0024365   .PMYHGIRCRS.G......KWVS..EI..REP........RKATRI....WLG...........................................TFPKPEMAAAAYD..VAALALKGD
EVM0006672   .PSFRGIRLRS.G......KWVS..EI..REP........RKTTRV....WLG...........................................TYPTPEMAATAYD..VAALALKGP
EVM0004316   .PSFKGIRLRS.G......KWVS..EI..REP........RKTTRV....WLG...........................................TYPTPEMAATAYD..VAALALKGP
EVM0044087   .PSYKGIRLRS.G......KWVS..EI..REP........RKSTRV....WLG...........................................TYPTPEMAAAAYD..VAALALKGP
EVM0010374   .PSYKGIRLRS.G......KWVS..EI..REP........RKTTRV....WLG...........................................TYPTPEMAAAAYD..VAALALKGP
EVM0015962   .RAFRGVRRRSSG......KWVS..EI..REP........KKPNRI....WLG...........................................TFPSPEMAAVAYD..VAALALKGQ
EVM0021255   .RAFRGVRRRSSG......KWVS..EI..REP........KKPNRI....WLG...........................................TFPNPEMAAVAYD..VAALALKGQ
EVM0054778   ..AFRGVRRRSSG......KWVS..EI..REP........RKPNRI....WLG...........................................TFPTPEMAAVAYD..VAALALKG.
EVM0000771   .HGFRGVRRRSSG......KWVS..EI..REP........RKPNRI....WLG...........................................TFPTPEMAAVAYD..VAALALKGQ
EVM0050604   ..RYRGVRQRKWG......KWVS..EI..REP........GKKTRI....WLG...........................................SYEMPEMAAAAYD..VAALHLRG.
PtDREBA4_1   .PRYRGVRQRKWG......KWVS..EI..REP........GKKTRI....WLG...........................................SYEMPEMAAAAYD..VAALHLRGR
EVM0042416   ...YRGVRQRKWG......KWVS..EI..REP........GKKTRI....WLG...........................................SYEMPEMAAAAYD..VAALHLRG.
EVM0006866   ..RYRGIRQRKWG......KWVS..EI..REP........GKKTRI....WLG...........................................SYEMPEMAAAAYD..AAALHLRG.
EVM0056353   ..RYRGIRRRKWG......KWVS..EI..REP........GKKTRI....WLG...........................................SYEMPEMAAAAYD..VAALHLRG.
EVM0016569   ...YRGVRKRKWG......KWVS..EI..REP........GKKSRI....WLG...........................................SFETPEMAATAYD..VAALHFRG.
EVM0005180   ...YRGVRKRKWG......KWVS..EI..REP........GKKSRI....WLG...........................................SFETPEMAATAYD..VAALHFRG.
EVM0002852   .SSYRGIRKRKWG......KWVS..EI..REP........GKKTRI....WLG.........................................................ILHFRGR
```

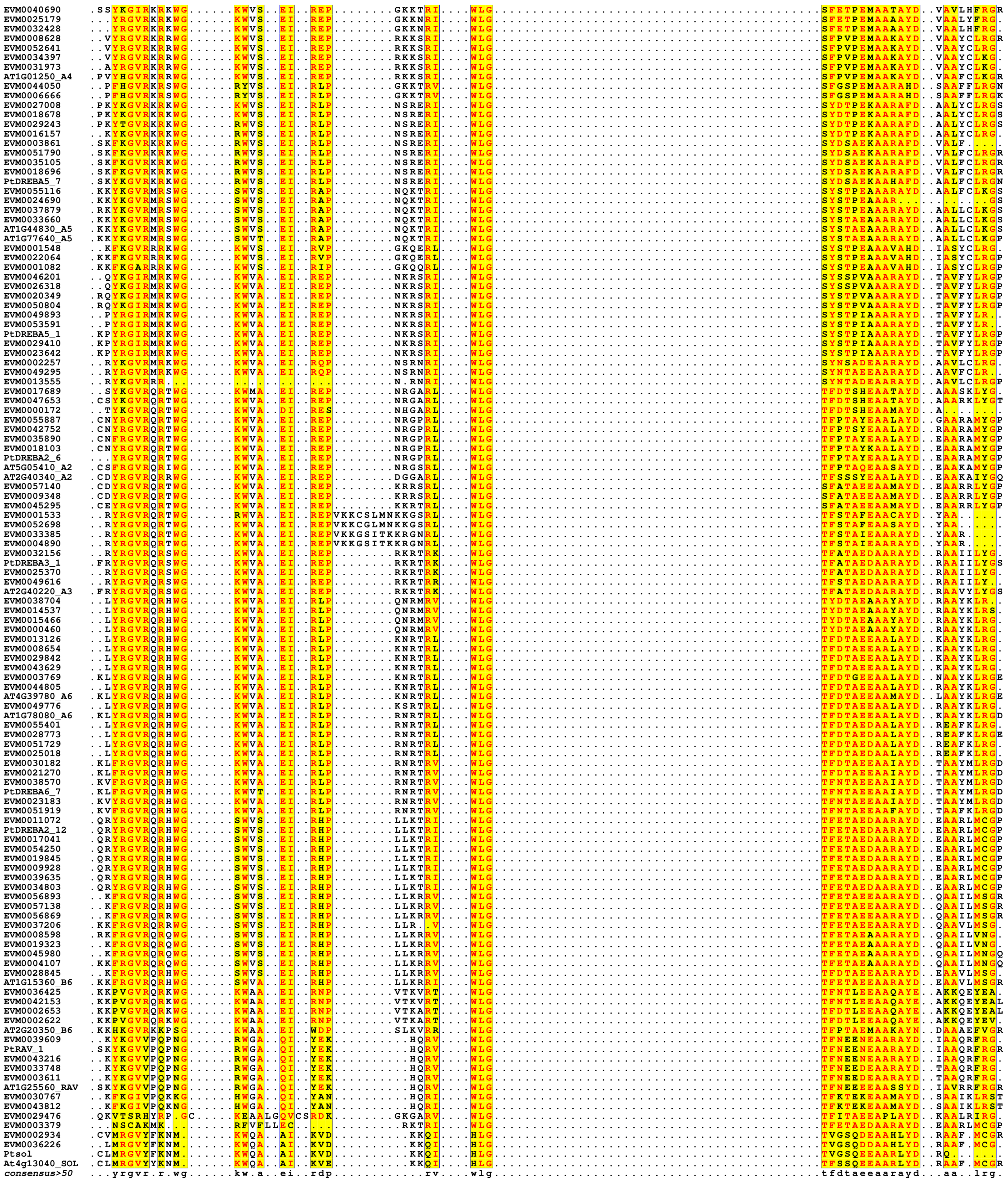

EVM0040310	....
EVM0040588	....
EVM0009672	....
EVM0023967	....
EVM0056670	....
EVM0019656	ETT..........
EVM0036581	....
EVM0015665	....
EVM0035539	....
EVM0003138	....
EVM0047762	....
EVM0011412	....
EVM0044041	....
EVM0001221	....
EVM0042515	....
EVM0040570	....
EVM0012824	....
EVM0008451	....
EVM0006637	....
EVM0025980	....
EVM0000144	....
EVM0033580	....
EVM0043933	....
EVM0050911	....
EVM0035153	....
EVM0056730	....
AT3G20840_AP2	....
EVM0022735	....
EVM0006689	....
EVM0007616	....
EVM0004062	....
EVM0050425	....
EVM0057600	....
EVM0011531	....
EVM0038265	....
EVM0018397	....
EVM0043533	....
EVM0009993	....
EVM0032803	....
EVM0028655	....
EVM0037464	DTILNFPLSTYQN
EVM0027529	....
EVM0036168	....
EVM0008767	....
EVM0052780	....
PtAP2_21	....
AT4G36920_AP2	....
EVM0010409	....
EVM0057697	....
EVM0052615	....
EVM0036052	....
EVM0020564	....
EVM0000418	....
EVM0039575	....
EVM0006536	....
EVM0043548	....
EVM0027097	....
EVM0008274	....
EVM0043935	....
EVM0046342	....
EVM0007646	....
EVM0025365	....
EVM0016170	....
EVM0030823	....
EVM0021312	....
EVM0024408	....
EVM0035393	....
EVM0051961	....
EVM0050816	....
EVM0027493	....
PtERFB6_2	....
EVM0052812	....
EVM0031439	....
PtERFB1_6	....
EVM0054412	....
EVM0018852	....
EVM0042683	....
EVM0026368	....
EVM0012457	....
EVM0032101	....
EVM0046023	....
EVM0055854	....
EVM0007543	....
EVM0043720	....
EVM0054855	....
PtERFB1_1	....
EVM0055399	....
EVM0013798	....
EVM0019343	....
EVM0042467	....
EVM0054100	....
EVM0006682	....
EVM0049140	....
EVM0053305	....
EVM0016530	K............
EVM0041629	....
EVM0000535	....
EVM0048064	....
EVM0049898	....
EVM0052258	....
AT1G28370_B1	....
EVM0023162	....
EVM0046939	....
EVM0008900	....
EVM0029866	....
EVM0045686	....
EVM0018037	....
EVM0003762	....
AT1G50640_B1	....
EVM0039676	....
EVM0032830	....
EVM0051611	....
EVM0004204	....
EVM0001371	....
EVM0049397	....
EVM0018988	....
PtERFB6_16	....
EVM0000006	....
EVM0044977	....
EVM0009817	....
EVM0020531	....
EVM0030192	....
EVM0040670	....
EVM0013811	....
AT4G17490_B3	....
EVM0041820	....
PtERFB3_3	....
EVM0036702	....
EVM0049755	....
EVM0006972	....
EVM0055475	....
EVM0054964	....
EVM0019924	....
EVM0055695	....
EVM0012873	....
EVM0036069	....
EVM0002921	....
EVM0040715	....
EVM0025220	....
EVM0054439	....
EVM0017235	....
EVM0014312	....
EVM0006944	....
EVM0041453	....
EVM0001248	....

```
EVM0008128      .............
EVM0040250      .............
EVM0035905      .............
EVM0025231      .............
EVM0017086      .............
EVM0045763      .............
EVM0038648      .............
EVM0022031      .............
EVM0045225      .............
EVM0045646      .............
EVM0008755      .............
EVM0018983      .............
EVM0023703      .............
AT2G31230_B3    .............
EVM0049532      .............
EVM0029622      .............
EVM0012743      .............
EVM0009409      .............
EVM0052364      .............
EVM0027771      .............
EVM0005349      .............
EVM0035055      .............
EVM0012041      .............
EVM0028403      .............
EVM0052605      .............
EVM0045883      .............
EVM0037606      .............
PtERFB3_12      .............
EVM0054822      .............
EVM0024412      .............
EVM0030239      .............
EVM0046641      .............
EVM0006603      .............
EVM0006649      .............
EVM0054070      .............
EVM0052610      .............
EVM0057308      .............
EVM0053966      .............
EVM0031305      .............
EVM0017641      .............
EVM0028978      .............
EVM0054684      .............
EVM0017324      .............
EVM0023232      .............
EVM0055941      .............
EVM0044966      .............
EVM0039499      .............
AT3G16770_B2    .............
AT2G47520_B2    .............
EVM0044386      .............
EVM0020075      .............
EVM0034491      .............
EVM0026277      .............
PtERFB4_1       .............
EVM0017998      .............
EVM0040725      .............
EVM0032029      .............
EVM0013578      .............
EVM0013502      .............
EVM0031724      .............
AT5G61890_B4    .............
EVM0023217      .............
EVM0030954      .............
EVM0047172      .............
EVM0000045      .............
EVM0004610      .............
EVM0001106      .............
EVM0002769      .............
EVM0011459      .............
EVM0043678      .............
AT2G33710_B4    .............
EVM0051566      .............
EVM0057361      .............
EVM0015843      .............
EVM0034620      .............
EVM0031168      .............
EVM0024472      .............
EVM0045726      .............
EVM0022896      .............
PtERFB5_5       .............
EVM0049667      .............
AT4G27950_B5    .............
EVM0012647      .............
EVM0015330      .............
PtERFB5_7       .............
EVM0043703      .............
EVM0017733      .............
EVM0001609      .............
EVM0054355      .............
EVM0044712      .............
EVM0026052      .............
EVM0041252      .............
AT2G46310_B5    .............
EVM0013188      .............
EVM0015672      .............
EVM0051058      .............
EVM0018299      .............
AT3G16280_A4    .............
EVM0040155      .............
EVM0028452      .............
EVM0034096      .............
EVM0053799      .............
EVM0032021      .............
PtDREBA4_17     SA...........
EVM0042411      .............
EVM0021344      .............
EVM0018160      .............
EVM0001696      .............
EVM0005032      .............
EVM0055308      .............
EVM0007023      .............
EVM0005644      .............
EVM0000005      .............
EVM0029808      .............
EVM0004638      .............
EVM0026062      .............
EVM0040623      .............
PtDREBA1_3      S............
EVM0052580      .............
EVM0044116      .............
EVM0009316      .............
EVM0013573      .............
EVM0014688      .............
EVM0032370      .............
AT4G25480_A1    .............
AT4G25490_A1    .............
EVM0018598      .............
EVM0057013      .............
EVM0024365      .............
EVM0006672      .............
EVM0004316      .............
EVM0044087      .............
EVM0010374      .............
EVM0015962      .............
EVM0021255      .............
EVM0054778      .............
EVM0000771      .............
EVM0050604      .............
PtDREBA4_1      GA...........
EVM0042416      .............
EVM0006866      .............
EVM0056353      .............
EVM0016569      .............
EVM0005180      .............
EVM0002852      GAKL.........
```

```
EVM0040690      .............
EVM0025179      .............
EVM0032428      .............
EVM0008628      .............
EVM0052641      .............
EVM0034397      .............
EVM0031973      .............
AT1G01250_A4    .............
EVM0044050      .............
EVM0006666      .............
EVM0027008      .............
EVM0018678      .............
EVM0029243      .............
EVM0016157      .............
EVM0003861      .............
EVM0051790      .............
EVM0035105      .............
EVM0018696      .............
PtDREBA5_7      .............
EVM0055116      .............
EVM0024690      .............
EVM0037879      .............
EVM0033660      .............
AT1G44830_A5    .............
AT1G77640_A5    .............
EVM0001548      .............
EVM0022064      .............
EVM0001082      .............
EVM0046201      .............
EVM0026318      .............
EVM0020349      .............
EVM0050804      .............
EVM0049893      .............
EVM0053591      .............
PtDREBA5_1      S............
EVM0029410      .............
EVM0023642      .............
EVM0002257      .............
EVM0049295      .............
EVM0013555      SATFNFP......
EVM0017689      .............
EVM0047653      .............
EVM0000172      .............
EVM0055887      .............
EVM0042752      .............
EVM0035890      .............
EVM0018103      .............
PtDREBA2_6      YAR..........
AT5G05410_A2    .............
AT2G40340_A2    .............
EVM0057140      .............
EVM0009348      .............
EVM0045295      .............
EVM0001533      .............
EVM0052698      .............
EVM0033385      .............
EVM0004890      .............
EVM0032156      .............
PtDREBA3_1      RA...........
EVM0025370      .............
EVM0049616      .............
AT2G40220_A3    .............
EVM0038704      .............
EVM0014537      .............
EVM0015466      .............
EVM0000460      .............
EVM0013126      .............
EVM0008654      .............
EVM0029842      .............
EVM0043629      .............
EVM0003769      .............
EVM0044805      .............
AT4G39780_A6    .............
EVM0049776      .............
AT1G78080_A6    .............
EVM0055401      .............
EVM0028773      .............
EVM0051729      .............
EVM0025018      .............
EVM0030182      .............
EVM0021270      .............
EVM0038570      .............
PtDREBA6_7      .............
EVM0023183      .............
EVM0051919      .............
EVM0011072      .............
PtDREBA2_12     RAR..........
EVM0017041      .............
EVM0054250      .............
EVM0019845      .............
EVM0009928      .............
EVM0039635      .............
EVM0034803      .............
EVM0056893      .............
EVM0057138      .............
EVM0056869      .............
EVM0037206      .............
EVM0008598      .............
EVM0019323      .............
EVM0045980      .............
EVM0004107      .............
EVM0028845      .............
AT1G15360_B6    .............
EVM0036425      .............
EVM0042153      .............
EVM0002653      .............
EVM0002622      .............
AT2G20350_B6    .............
EVM0039609      .............
PtRAV_1         .............
EVM0043216      .............
EVM0033748      .............
EVM0003611      .............
AT1G25560_RAV   .............
EVM0030767      DLH..........
EVM0043812      DLHRN........
EVM0029476      .............
EVM0003379      .............
EVM0002934      E............
EVM0036226      .............
Ptsol           .............
At4g13040_SOL   E............
consensus>50    .............
```
