# Supplementary figures and images for: Genome-wide investigation of the AP2/ERF superfamily and their expression under salt stress in Chinese willow (Salix matsudana)
Source: PeerJ. 2021 Apr 13;9:e11076. doi: 10.7717/peerj.11076 (PMC8051338; doi:10.7717/peerj.11076)

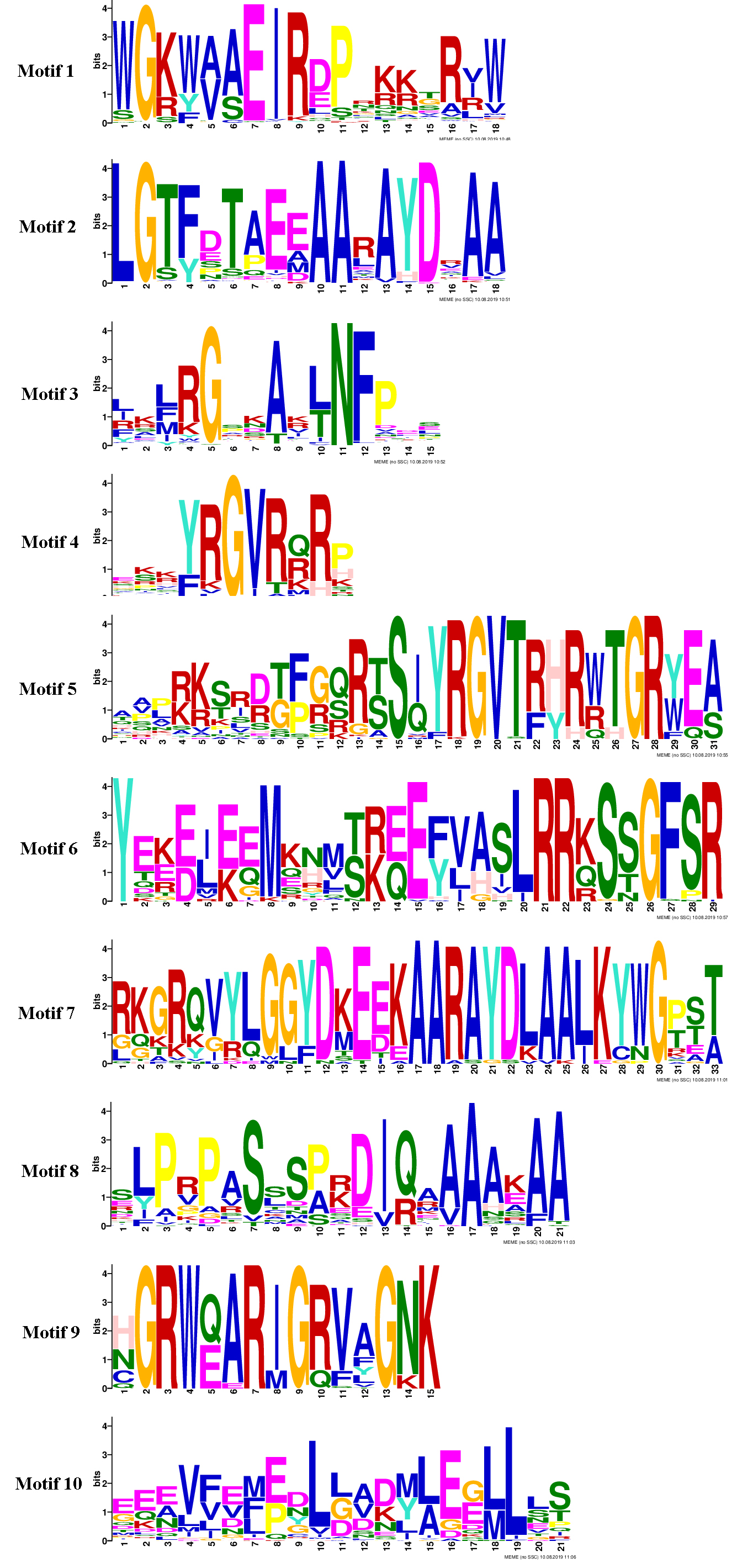

Supplement: Supplemental Information 3 — The online tool MEME (http://meme-suite.org/tools/meme) was used to search for conserved motifs of SmAP2/ERF superfamily proteins. The optimized parameters were employed as follows: any number of repetitions, maximum number of motifs = 10, and the optimum width of each motif was 6–50 residues. [file peerj-09-11076-s003.jpg]

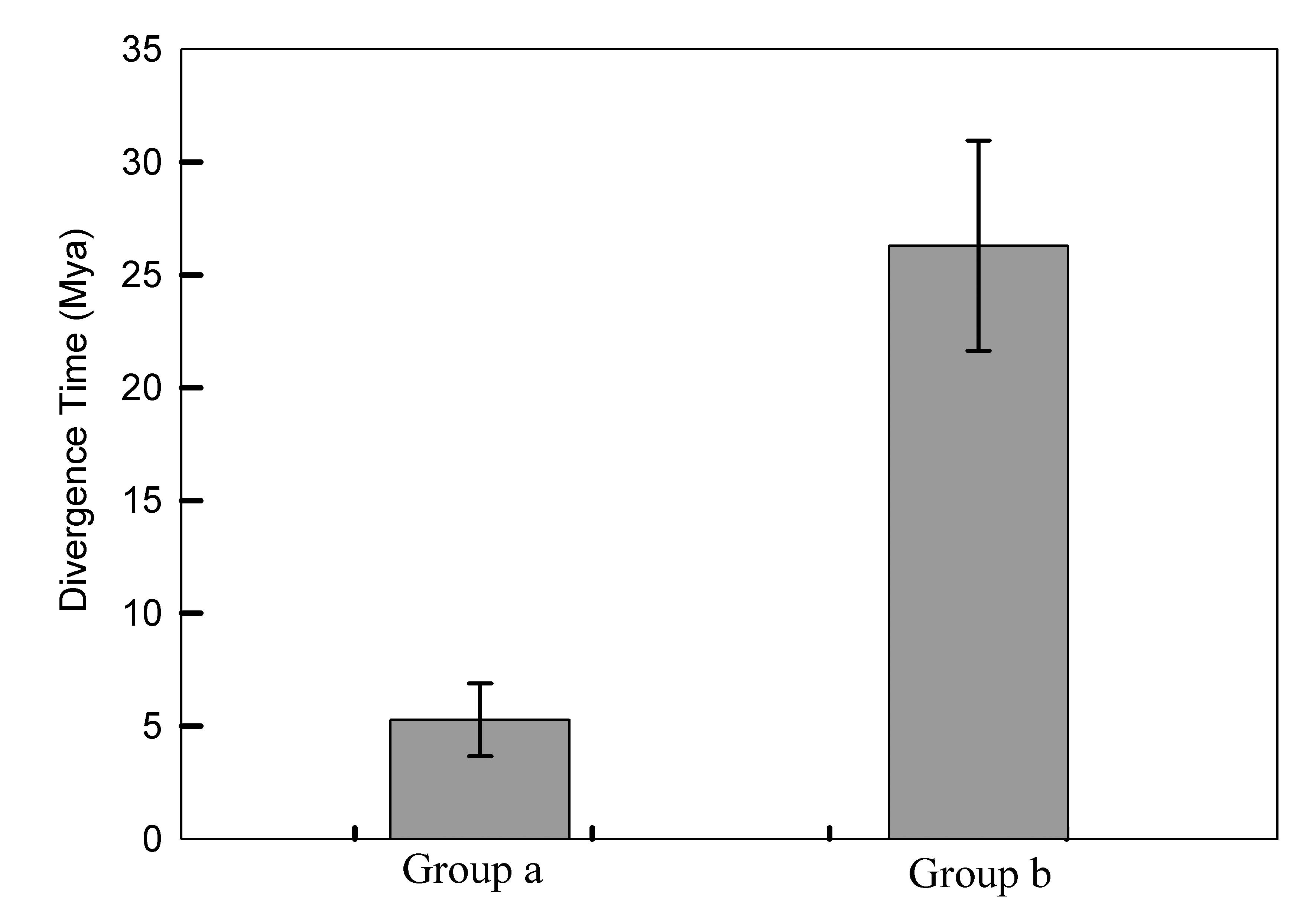

Supplement: Supplemental Information 4 — The divergence time (T Value) of gene pairs can be classified mainly into two groups, a group and b group with two time period, 2–8 Mya (average value, 5Mya ) and 20–36 Mya (average value, 26Mya) respectively. [file peerj-09-11076-s004.jpg]
