# Supplementary material for: Genome-wide investigation of the AP2/ERF superfamily and their expression under salt stress in Chinese willow (Salix matsudana)
Source: PeerJ. 2021 Apr 13;9:e11076. doi: 10.7717/peerj.11076 (PMC8051338; doi:10.7717/peerj.11076)
Supplement: Supplemental Information 5 [file peerj-09-11076-s005.docx]

Genome-wide investigation of the AP2/ERF superfamily and their expression under salt stress in Chinese willow (*Salix matsudana*)

Running title: AP2/ERF superfamily in Chinese willow

Jian Zhang^1a^, Yuna Jiang^1a^, Shi zheng Shi^2a^, Fei Zhong^1^, Guoyuan Liu^1^, Chunmei Yu^1^, Bolin Lian^1^, Yanhong Chen ^1^*

^1^Lab of Landscape Plant Genetics and Breeding, School of Life Science, Nantong University, Nantong, Jiangsu Province, China

^2^ Jiangsu Academy of Forestry，Nanjing 211153，China

^a^These authors contribute equally to this work

*Corresponding author. E-mail: [chenyh@ntu.edu.cn](mailto:chenyh@ntu.edu.cn) ; FAX: 86-513-85012818; Tel: 86-513-85012818

E-mail address for other authors

Jian Zhang: 56071007@qq.com

Yuna Jiang :[18252098108@163.com](mailto:18252098108@163.com);

Shizheng Shi: shshzn@163.com

Fei Zhong: [fzhong@ntu.edu.cn](mailto:fzhong@ntu.edu.cn);

Guoyuan Liu: [cjqm1989@126.com](mailto:cjqm1989@126.com);

Chunmei Yu: [ychmei@ntu.edu.cn](mailto:ychmei@ntu.edu.cn);

Bolin Lian: [lianziadd9@163.com](mailto:lianziadd9@163.com);

File S1 AP2.hmm

HMMER3/f [3.1b2 | February 2015]

NAME AP2

ACC PF00847.20

DESC AP2 domain

LENG 54

ALPH amino

RF no

MM no

CONS yes

CS no

MAP yes

DATE Fri Aug 3 10:13:30 2018

NSEQ 73

EFFN 18.209900

CKSUM 3632432801

GA 20.60 20.60;

TC 20.60 20.60;

NC 20.50 20.50;

BM hmmbuild HMM.ann SEED.ann

SM hmmsearch -Z 45638612 -E 1000 --cpu 4 HMM pfamseq

STATS LOCAL MSV -8.1511 0.71908

STATS LOCAL VITERBI -8.6976 0.71908

STATS LOCAL FORWARD -3.9453 0.71908

HMM A C D E F G H I K L M N P Q R S T V W Y

m->m m->i m->d i->m i->i d->m d->d

COMPO 2.32363 4.23749 3.31158 2.75484 3.16732 2.65190 3.77385 3.19116 2.31296 2.98860 4.06360 3.16832 3.71101 3.23270 2.43625 2.73880 3.08870 2.84999 3.49959 2.94510

2.68618 4.42225 2.77519 2.73123 3.46354 2.40513 3.72494 3.29354 2.67741 2.69355 4.24690 2.90347 2.73739 3.18146 2.89801 2.37887 2.77519 2.98518 4.58477 3.61503

0.02134 6.52953 3.92963 0.61958 0.77255 0.00000 *

1 2.78119 2.86059 3.23429 2.88131 4.61512 3.61164 4.76061 4.35083 2.85876 3.21006 5.26177 3.64315 1.60727 3.86851 3.35046 1.70840 2.19775 3.14791 6.66508 5.27463 1 p - - -

2.68618 4.42225 2.77519 2.73123 3.46354 2.40513 3.72494 3.29354 2.67741 2.69355 4.24690 2.90347 2.73739 3.18146 2.89801 2.37887 2.77519 2.98518 4.58477 3.61503

0.00221 6.51041 7.23275 0.61958 0.77255 0.36914 1.17548

2 3.33402 3.21506 2.59490 3.52982 5.52360 2.01572 3.20231 3.14137 1.66579 2.83823 5.26631 3.46482 4.29484 3.49668 2.44367 2.86337 4.02976 2.46141 3.95824 5.28395 2 k - - -

2.68618 4.42225 2.77519 2.73123 3.46354 2.40513 3.72494 3.29354 2.67741 2.69355 4.24690 2.90347 2.73739 3.18146 2.89801 2.37887 2.77519 2.98518 4.58477 3.61503

0.00217 6.52953 7.25188 0.61958 0.77255 0.48576 0.95510

3 3.93649 5.31540 5.87322 4.27474 2.61115 5.10287 3.75527 1.53096 5.05055 3.00609 3.95878 4.14679 2.80774 5.18686 4.18679 3.28556 4.16866 1.56284 5.93673 1.59654 3 i - - -

2.68618 4.42225 2.77519 2.73123 3.46354 2.40513 3.72494 3.29354 2.67741 2.69355 4.24690 2.90347 2.73739 3.18146 2.89801 2.37887 2.77519 2.98518 4.58477 3.61503

0.00217 6.52953 7.25188 0.61958 0.77255 0.48576 0.95510

4 3.29548 6.29420 3.68597 3.25415 5.63573 4.04123 4.08817 3.13081 1.41091 4.59797 3.93738 4.03589 2.92566 2.89993 1.62239 2.22258 3.39907 2.64856 6.72900 5.32021 4 k - - -

2.68618 4.42225 2.77519 2.73123 3.46354 2.40513 3.72494 3.29354 2.67741 2.69355 4.24690 2.90347 2.73739 3.18146 2.89801 2.37887 2.77519 2.98518 4.58477 3.61503

0.00217 6.52953 7.25188 0.61958 0.77255 0.48576 0.95510

5 4.29259 4.12784 6.60189 6.66811 7.20017 0.17564 7.26930 6.74723 6.72036 6.43303 7.14015 2.93069 5.89226 6.72543 6.65476 2.95055 4.89764 5.70372 8.53077 7.55445 5 G - - -

2.68618 4.42225 2.77519 2.73123 3.46354 2.40513 3.72494 3.29354 2.67741 2.69355 4.24690 2.90347 2.73739 3.18146 2.89801 2.37887 2.77519 2.98518 4.58477 3.61503

0.00217 6.52953 7.25188 0.61958 0.77255 0.48576 0.95510

6 5.16925 6.31829 7.35528 6.93618 5.70090 6.94643 7.80141 2.16258 6.93896 3.17185 5.43278 7.11093 7.09669 7.21048 7.13370 6.43237 5.43096 0.25115 7.95209 3.31156 6 V - - -

2.68632 4.42239 2.77534 2.73138 3.46368 2.40527 3.72509 3.29368 2.67755 2.69369 4.24704 2.90361 2.73754 3.18161 2.89815 2.37901 2.77300 2.98533 4.58491 3.61518

0.04141 3.22256 7.25188 0.68066 0.70579 0.48576 0.95510

7 3.85574 3.92584 3.80342 3.95425 3.34720 4.77395 3.88216 4.28065 3.18860 3.43835 4.83347 4.41646 5.15772 4.25330 1.22083 2.31591 2.53431 4.02968 3.52635 1.57962 9 r - - -

2.68618 4.42225 2.77519 2.73123 3.46354 2.40513 3.72494 3.29354 2.67741 2.69355 4.24690 2.90347 2.73739 3.18146 2.89801 2.37887 2.77519 2.98518 4.58477 3.61503

0.00217 6.52953 7.25188 0.61958 0.77255 0.48576 0.95510

8 3.57023 5.50371 5.21331 4.62615 2.04380 5.01866 5.27377 3.96659 2.77754 3.00764 4.61574 4.90680 5.38913 2.34643 1.89096 4.29455 4.19195 3.13635 2.00376 1.58372 10 y - - -

2.68618 4.42225 2.77519 2.73123 3.46354 2.40513 3.72494 3.29354 2.67741 2.69355 4.24690 2.90347 2.73739 3.18146 2.89801 2.37887 2.77519 2.98518 4.58477 3.61503

0.00217 6.52953 7.25188 0.61958 0.77255 0.48576 0.95510

9 3.79124 4.54383 1.47378 2.90600 5.64957 3.22361 2.28790 3.15578 2.88805 4.60933 5.34417 2.33969 4.95822 3.50870 1.72750 3.09508 3.33604 4.69478 6.73593 3.77284 11 d - - -

2.68618 4.42225 2.77519 2.73123 3.46354 2.40513 3.72494 3.29354 2.67741 2.69355 4.24690 2.90347 2.73739 3.18146 2.89801 2.37887 2.77519 2.98518 4.58477 3.61503

0.00217 6.52953 7.25188 0.61958 0.77255 0.48576 0.95510

10 2.91673 6.29724 3.36173 3.13900 4.13545 3.42034 3.60530 3.74565 1.20433 3.31204 5.33846 3.50547 1.84022 3.85055 2.26922 2.88713 3.44688 4.00201 6.73121 5.32156 12 k - - -

2.68618 4.42225 2.77519 2.73123 3.46354 2.40513 3.72494 3.29354 2.67741 2.69355 4.24690 2.90347 2.73739 3.18146 2.89801 2.37887 2.77519 2.98518 4.58477 3.61503

0.18707 6.52953 1.77695 0.61958 0.77255 0.48576 0.95510

11 2.87187 4.23758 3.92942 3.37335 5.43173 3.83441 3.62771 4.09174 1.54733 4.40538 5.15709 2.47767 4.30134 3.73439 2.11286 1.69391 2.64316 3.82874 3.17097 4.26378 13 k - - -

2.68618 4.42225 2.77519 2.73123 3.46354 2.40513 3.72494 3.29354 2.67741 2.69355 4.24690 2.90347 2.73739 3.18146 2.89801 2.37887 2.77519 2.98518 4.58477 3.61503

0.07642 6.34507 2.63365 0.61958 0.77255 0.12843 2.11590

12 3.06838 6.25092 2.66496 3.23901 4.46428 3.45321 3.06621 3.41917 2.56037 3.20990 3.45631 3.25977 3.49943 2.36968 2.14137 2.67613 2.31359 4.64039 2.21666 5.27527 14 r - - -

2.68618 4.42225 2.77519 2.73123 3.46354 2.40513 3.72494 3.29354 2.67741 2.69355 4.24690 2.90347 2.73739 3.18146 2.89801 2.37887 2.77519 2.98518 4.58477 3.61503

0.00231 6.46871 7.19105 0.61958 0.77255 0.24542 1.52499

13 3.45910 4.19172 3.65938 2.97869 5.64970 1.21903 3.69961 4.22293 2.02077 4.60943 3.34153 2.49697 3.31254 2.41901 3.05938 2.72423 4.02231 4.69488 6.73597 4.42512 15 g - - -

2.68618 4.42225 2.77519 2.73123 3.46354 2.40513 3.72494 3.29354 2.67741 2.69355 4.24690 2.90347 2.73739 3.18146 2.89801 2.37887 2.77519 2.98518 4.58477 3.61503

0.00217 6.52953 7.25188 0.61958 0.77255 0.48576 0.95510

14 2.91467 4.44032 4.05498 3.03496 5.60538 3.43611 4.76089 4.28314 1.74064 2.96436 5.31731 3.33259 4.96565 2.75903 1.24787 2.22391 4.02498 3.06562 6.71371 4.47327 16 r - - -

2.68588 4.42264 2.77559 2.73095 3.46393 2.40492 3.72534 3.29393 2.67701 2.69394 4.24729 2.90386 2.73779 3.18072 2.89840 2.37926 2.77559 2.98270 4.58516 3.61542

0.11505 2.55354 3.47790 0.96197 0.48149 0.48576 0.95510

15 6.78232 7.51891 7.38848 7.46328 2.19285 7.05172 5.52068 6.28317 7.21692 5.30983 6.75529 6.49989 7.27946 6.71652 6.91393 6.47910 6.95063 6.26275 0.28242 2.21454 20 W - - -

2.68618 4.42225 2.77519 2.73123 3.46354 2.40513 3.72494 3.29354 2.67741 2.69355 4.24690 2.90347 2.73739 3.18146 2.89801 2.37887 2.77519 2.98518 4.58477 3.61503

0.00224 6.49890 7.22125 0.61958 0.77255 0.81694 0.58300

16 2.26317 5.52420 4.83960 2.53023 2.77069 4.86622 5.12907 3.11152 3.43772 2.03792 3.41299 3.41006 5.24364 3.90605 2.14127 3.10808 3.14262 1.53830 6.11363 4.88992 21 v - - -

2.68637 4.42245 2.77539 2.73143 3.46060 2.40532 3.72514 3.29374 2.67760 2.69374 4.24709 2.90366 2.73759 3.18166 2.89642 2.37906 2.77539 2.98538 4.58496 3.61523

0.05710 2.90457 7.22125 0.68344 0.70295 0.81694 0.58300

17 0.57713 2.68716 6.81312 6.70728 6.53330 3.91852 7.00894 5.94141 6.50958 5.68901 6.50681 5.88863 5.82485 6.55215 6.43222 2.73155 2.68511 1.64287 7.96065 6.90065 24 A - - -

2.68618 4.42225 2.77519 2.73123 3.46354 2.40513 3.72494 3.29354 2.67741 2.69355 4.24690 2.90347 2.73739 3.18146 2.89801 2.37887 2.77519 2.98518 4.58477 3.61503

0.00224 6.49890 7.22125 0.61958 0.77255 0.32337 1.28628

18 3.56201 4.41919 4.11315 1.55458 3.51602 3.72772 2.66438 3.91718 3.23782 3.25185 5.22554 3.43214 4.99255 2.00083 2.89311 2.60609 3.01336 3.30580 4.58859 2.24632 25 e - - -

2.68852 4.42086 2.77119 2.73115 3.46634 2.40661 3.72775 3.28484 2.67752 2.69336 4.24970 2.90556 2.73972 3.18427 2.89333 2.38075 2.77692 2.98280 4.57553 3.61220

0.59336 0.82435 4.70891 1.20819 0.35487 0.48576 0.95510

19 3.87193 5.58350 4.76433 2.63291 2.24030 3.41961 4.10978 2.97531 4.14964 3.79406 4.68716 3.05972 2.01031 4.44949 3.92469 2.88170 4.10367 3.17415 1.34818 2.90827 30 w - - -

2.68618 4.42225 2.77519 2.73123 3.46354 2.40513 3.72494 3.29354 2.67741 2.69355 4.24690 2.90347 2.73739 3.18146 2.89801 2.37887 2.77519 2.98518 4.58477 3.61503

0.18169 6.52119 1.80382 0.61958 0.77255 0.42654 1.05776

20 3.26960 2.87379 3.39079 3.37255 4.08106 2.87970 4.63163 4.92598 2.07653 4.42391 4.16941 2.35808 4.83556 3.01846 2.32464 1.95750 2.64359 2.73046 6.57068 2.71030 31 s - - -

2.68618 4.42225 2.77519 2.73123 3.46354 2.40513 3.72494 3.29354 2.67741 2.69355 4.24690 2.90347 2.73739 3.18146 2.89801 2.37887 2.77519 2.98518 4.58477 3.61503

0.03318 6.35209 3.47719 0.61958 0.77255 1.43310 0.27255

21 3.35505 6.15796 2.28615 2.03222 5.50413 2.54300 4.60505 2.71206 1.97577 4.46371 5.19833 2.53949 4.81160 3.22972 3.04259 2.29644 2.27899 3.46936 6.58999 5.17837 32 k - - -

2.68614 4.42332 2.77627 2.73111 3.46461 2.40620 3.72176 3.29286 2.67760 2.69290 4.24655 2.90454 2.73611 3.18173 2.89644 2.37799 2.77558 2.98625 4.58584 3.61610

0.77771 0.81924 2.30482 0.44949 1.01598 1.07949 0.41517

22 3.07809 6.12955 2.37623 2.86481 5.47569 2.04341 4.57436 4.96282 3.05459 4.43526 5.17016 1.38236 4.78025 2.80017 2.01815 2.85774 2.85516 4.52081 6.56135 5.14907 36 n - - -

2.68618 4.42225 2.77519 2.73123 3.46354 2.40513 3.72494 3.29354 2.67741 2.69355 4.24690 2.90347 2.73739 3.18146 2.89801 2.37887 2.77519 2.98518 4.58477 3.61503

0.07352 6.28115 2.67350 0.61958 0.77255 0.58060 0.81999

23 2.87657 4.53621 3.92385 3.20694 5.50599 1.09382 4.63242 4.12569 2.31674 4.46898 5.20853 1.64709 4.83851 3.34964 2.89725 2.96718 3.42235 4.55402 6.60193 5.19501 37 g - - -

2.68618 4.42225 2.77519 2.73123 3.46354 2.40513 3.72494 3.29354 2.67741 2.69355 4.24690 2.90347 2.73739 3.18146 2.89801 2.37887 2.77519 2.98518 4.58477 3.61503

0.34697 6.36198 1.23289 0.61958 0.77255 1.58703 0.22883

24 3.14873 3.40647 4.10428 3.54216 4.55276 4.38118 4.60980 2.77759 1.36864 3.60972 3.68783 3.27350 4.76562 3.84667 2.13978 2.24232 3.15247 2.77643 5.93688 2.55783 38 k - - -

2.68663 4.42270 2.77564 2.72996 3.46040 2.40558 3.72539 3.29399 2.67786 2.69400 4.24735 2.89982 2.73784 3.18191 2.89846 2.37932 2.77564 2.98563 4.58522 3.61548

0.10755 2.29471 6.74097 1.31915 0.31110 1.12791 0.39113

25 3.54846 3.88757 3.85282 3.10391 2.38817 4.34365 3.91481 2.88643 1.77582 4.21233 4.98320 3.39933 4.73563 2.82806 1.28049 2.66044 3.42076 4.29242 6.39248 4.13666 43 r - - -

2.68618 4.42225 2.77519 2.73123 3.46354 2.40513 3.72494 3.29354 2.67741 2.69355 4.24690 2.90347 2.73739 3.18146 2.89801 2.37887 2.77519 2.98518 4.58477 3.61503

0.00307 6.18288 6.90523 0.61958 0.77255 1.23374 0.34418

26 3.05402 3.65486 4.42569 3.86088 3.50143 2.77308 3.31276 2.90555 1.73002 2.93576 3.56451 4.29737 4.99167 2.62408 1.83173 3.41369 3.02227 2.24360 6.03095 4.78181 44 k - - -

2.68618 4.42225 2.77519 2.73123 3.46354 2.40513 3.72494 3.29354 2.67741 2.69355 4.24690 2.90347 2.73739 3.18146 2.89801 2.37887 2.77519 2.98518 4.58477 3.61503

0.00283 6.26487 6.98722 0.61958 0.77255 0.59872 0.79743

27 2.97479 4.46988 3.16427 3.40607 3.28260 3.33910 3.04948 4.23867 1.96519 3.23671 3.70043 2.59046 4.87527 2.88990 2.17725 2.04815 2.36949 4.58072 6.63270 3.28311 45 k - - -

2.68546 4.41384 2.77611 2.73215 3.46445 2.40382 3.72536 3.29445 2.67624 2.69446 4.24781 2.90377 2.73805 3.18154 2.89570 2.37939 2.77577 2.98610 4.58568 3.61531

0.34992 1.22262 7.13557 0.61320 0.78004 0.17245 1.84265

28 2.10379 4.14117 3.32224 3.30666 5.64666 4.56529 4.14667 4.56262 1.24277 4.60693 5.34243 3.71418 4.95875 3.07664 1.66984 2.76340 2.51944 3.49263 6.73447 5.32364 49 k - - -

2.68618 4.42225 2.77519 2.73123 3.46354 2.40513 3.72494 3.29354 2.67741 2.69355 4.24690 2.90347 2.73739 3.18146 2.89801 2.37887 2.77519 2.98518 4.58477 3.61503

0.00217 6.52953 7.25188 0.61958 0.77255 0.48576 0.95510

29 3.46331 6.27193 3.65883 3.49950 5.60353 2.96429 3.78373 2.93996 2.57520 3.93880 5.31614 3.12255 4.96581 2.87721 1.65768 2.12929 2.41540 3.94160 4.40279 1.95477 50 r - - -

2.68618 4.42225 2.77519 2.73123 3.46354 2.40513 3.72494 3.29354 2.67741 2.69355 4.24690 2.90347 2.73739 3.18146 2.89801 2.37887 2.77519 2.98518 4.58477 3.61503

0.00217 6.52953 7.25188 0.61958 0.77255 0.48576 0.95510

30 4.05514 5.42158 6.01163 5.40754 0.72783 5.21920 4.32952 2.44421 3.53029 2.64620 4.52982 5.36941 5.57866 5.30808 5.18984 4.53307 4.28686 1.75123 5.91957 2.95120 51 f - - -

2.68618 4.42225 2.77519 2.73123 3.46354 2.40513 3.72494 3.29354 2.67741 2.69355 4.24690 2.90347 2.73739 3.18146 2.89801 2.37887 2.77519 2.98518 4.58477 3.61503

0.00217 6.52953 7.25188 0.61958 0.77255 0.48576 0.95510

31 3.42962 6.28152 4.05102 3.49511 5.61728 3.17515 3.38391 5.09666 3.24144 4.09878 5.32465 3.17995 2.87667 2.80166 2.50216 1.38804 3.11115 4.66715 1.83627 2.68083 52 s - - -

2.68618 4.42225 2.77519 2.73123 3.46354 2.40513 3.72494 3.29354 2.67741 2.69355 4.24690 2.90347 2.73739 3.18146 2.89801 2.37887 2.77519 2.98518 4.58477 3.61503

0.00217 6.52953 7.25188 0.61958 0.77255 0.48576 0.95510

32 2.71003 3.71522 5.90329 5.28800 4.40582 3.95445 5.44233 1.69866 3.02245 1.44669 3.54428 5.27117 2.98803 5.20392 5.08077 3.85344 4.17109 1.36769 5.93589 4.75992 53 v - - -

2.68618 4.42225 2.77519 2.73123 3.46354 2.40513 3.72494 3.29354 2.67741 2.69355 4.24690 2.90347 2.73739 3.18146 2.89801 2.37887 2.77519 2.98518 4.58477 3.61503

0.00217 6.52953 7.25188 0.61958 0.77255 0.48576 0.95510

33 2.44737 4.53901 4.05709 2.58475 4.36094 1.45453 4.76213 4.25571 1.51754 3.96611 5.31334 3.06022 4.96659 3.53151 3.99303 2.52485 2.35654 4.65139 6.71043 4.52644 54 g - - -

2.68618 4.42225 2.77519 2.73123 3.46354 2.40513 3.72494 3.29354 2.67741 2.69355 4.24690 2.90347 2.73739 3.18146 2.89801 2.37887 2.77519 2.98518 4.58477 3.61503

0.02617 6.52953 3.71449 0.61958 0.77255 0.48576 0.95510

34 3.02058 6.27644 3.61533 3.22931 5.61818 3.88467 3.29239 3.41264 1.10556 3.62700 5.31798 4.01753 4.94216 2.98326 2.94114 3.22378 1.54350 4.66529 6.71110 3.39964 55 k - - -

2.68618 4.42225 2.77519 2.73123 3.46354 2.40513 3.72494 3.29354 2.67741 2.69355 4.24690 2.90347 2.73739 3.18146 2.89801 2.37887 2.77519 2.98518 4.58477 3.61503

0.00222 6.50559 7.22793 0.61958 0.77255 0.34840 1.22356

35 4.99912 6.37733 6.25333 3.80431 1.28942 5.94617 2.12061 4.88990 5.52549 2.75442 5.51605 5.75599 6.28272 5.71068 2.96920 4.01779 5.22114 4.71984 5.73578 0.92899 56 y - - -

2.68618 4.42225 2.77519 2.73123 3.46354 2.40513 3.72494 3.29354 2.67741 2.69355 4.24690 2.90347 2.73739 3.18146 2.89801 2.37887 2.77519 2.98518 4.58477 3.61503

0.00217 6.52953 7.25188 0.61958 0.77255 0.48576 0.95510

36 2.85252 7.37607 1.88344 2.73792 6.68018 0.61091 5.53423 6.22429 3.87553 5.67178 6.48518 2.92320 3.12191 4.68202 5.12637 3.73507 4.99084 5.75531 7.80772 6.27867 57 G - - -

2.68618 4.42225 2.77519 2.73123 3.46354 2.40513 3.72494 3.29354 2.67741 2.69355 4.24690 2.90347 2.73739 3.18146 2.89801 2.37887 2.77519 2.98518 4.58477 3.61503

0.00217 6.52953 7.25188 0.61958 0.77255 0.48576 0.95510

37 3.90557 4.21784 5.15284 3.23545 1.60064 3.66023 5.25539 3.30250 3.33307 3.12685 3.29142 4.16103 5.34190 4.16719 4.73481 2.26251 1.65095 3.70863 6.05374 1.79399 58 f - - -

2.68334 4.42309 2.77531 2.73023 3.46438 2.40596 3.72578 3.29438 2.67776 2.69277 4.24773 2.90370 2.73588 3.18230 2.89884 2.37970 2.77603 2.98415 4.58561 3.61587

0.52595 0.89576 7.25188 0.09212 2.43040 0.48576 0.95510

38 2.18780 6.29667 2.73042 1.19867 3.02099 4.56640 4.08197 3.35504 2.50479 3.14991 5.33796 2.74047 4.95981 2.72103 2.91707 3.75512 4.02288 3.97018 6.73080 3.64549 61 e - - -

2.68618 4.42225 2.77519 2.73123 3.46354 2.40513 3.72494 3.29354 2.67741 2.69355 4.24690 2.90347 2.73739 3.18146 2.89801 2.37887 2.77519 2.98518 4.58477 3.61503

0.00217 6.52953 7.25188 0.61958 0.77255 0.48576 0.95510

39 2.69218 6.30440 3.32113 1.09765 5.65059 3.48319 4.75143 5.13735 2.24876 2.89343 2.92604 2.92790 4.95798 2.55102 3.04936 2.76454 3.64781 4.69564 6.73641 4.41398 62 e - - -

2.68618 4.42225 2.77519 2.73123 3.46354 2.40513 3.72494 3.29354 2.67741 2.69355 4.24690 2.90347 2.73739 3.18146 2.89801 2.37887 2.77519 2.98518 4.58477 3.61503

0.00217 6.52953 7.25188 0.61958 0.77255 0.48576 0.95510

40 0.20074 6.18122 6.51290 6.39134 6.91659 4.99168 7.03361 6.38432 6.30081 6.08946 6.84945 5.84441 5.85742 2.95737 6.35552 2.89602 3.84756 3.72452 8.26805 7.22418 63 A - - -

2.68618 4.42225 2.77519 2.73123 3.46354 2.40513 3.72494 3.29354 2.67741 2.69355 4.24690 2.90347 2.73739 3.18146 2.89801 2.37887 2.77519 2.98518 4.58477 3.61503

0.00217 6.52953 7.25188 0.61958 0.77255 0.48576 0.95510

41 1.49194 6.20023 4.40599 3.82226 3.55253 4.24185 3.91328 4.87624 1.12588 3.49755 5.26975 4.32663 5.18459 4.09771 1.58439 4.02219 4.21766 4.55025 6.65586 3.74905 64 k - - -

2.68618 4.42225 2.77519 2.73123 3.46354 2.40513 3.72494 3.29354 2.67741 2.69355 4.24690 2.90347 2.73739 3.18146 2.89801 2.37887 2.77519 2.98518 4.58477 3.61503

0.00217 6.52953 7.25188 0.61958 0.77255 0.48576 0.95510

42 2.98367 6.29950 2.86234 2.44328 5.64339 4.56594 3.58536 3.79168 1.61878 2.17627 3.56880 3.49238 4.95937 2.77593 1.55343 3.75458 4.02288 4.68951 6.73286 3.30843 65 r - - -

2.68618 4.42225 2.77519 2.73123 3.46354 2.40513 3.72494 3.29354 2.67741 2.69355 4.24690 2.90347 2.73739 3.18146 2.89801 2.37887 2.77519 2.98518 4.58477 3.61503

0.00217 6.52953 7.25188 0.61958 0.77255 0.48576 0.95510

43 1.04057 6.20176 4.10606 3.25446 5.50247 4.60002 3.77667 4.95708 2.90395 1.44623 3.88417 3.66019 4.99199 3.12129 2.61381 3.22311 3.56455 4.56840 6.66117 5.28067 66 a - - -

2.68618 4.42225 2.77519 2.73123 3.46354 2.40513 3.72494 3.29354 2.67741 2.69355 4.24690 2.90347 2.73739 3.18146 2.89801 2.37887 2.77519 2.98518 4.58477 3.61503

0.00217 6.52953 7.25188 0.61958 0.77255 0.48576 0.95510

44 0.67275 3.29582 5.03303 4.48909 5.10927 4.95858 2.93598 4.56626 2.85087 4.25833 5.14312 4.84281 5.44153 4.71252 4.63641 3.25636 4.36904 4.29866 6.53486 1.69571 67 A - - -

2.68618 4.42225 2.77519 2.73123 3.46354 2.40513 3.72494 3.29354 2.67741 2.69355 4.24690 2.90347 2.73739 3.18146 2.89801 2.37887 2.77519 2.98518 4.58477 3.61503

0.00217 6.52953 7.25188 0.61958 0.77255 0.48576 0.95510

45 3.90634 2.82241 1.51710 2.66608 4.62163 4.94407 5.21971 1.00389 3.66962 3.13537 3.93436 4.78289 5.31870 4.64929 3.57302 4.20741 4.13828 2.76941 6.10229 4.89027 68 i - - -

2.68618 4.42225 2.77519 2.73123 3.46354 2.40513 3.72494 3.29354 2.67741 2.69355 4.24690 2.90347 2.73739 3.18146 2.89801 2.37887 2.77519 2.98518 4.58477 3.61503

0.00217 6.52953 7.25188 0.61958 0.77255 0.48576 0.95510

46 1.76701 4.82738 3.75223 1.77046 5.55009 4.58249 4.77288 4.16383 2.81504 1.96825 4.04243 3.43261 4.97522 2.36351 2.52100 2.85006 4.02811 2.98363 6.68521 3.47487 69 a - - -

2.68618 4.42225 2.77519 2.73123 3.46354 2.40513 3.72494 3.29354 2.67741 2.69355 4.24690 2.90347 2.73739 3.18146 2.89801 2.37887 2.77519 2.98518 4.58477 3.61503

0.00217 6.52953 7.25188 0.61958 0.77255 0.48576 0.95510

47 1.28313 2.70821 5.88891 5.27450 1.99257 5.10562 3.61612 3.02899 3.51552 3.50410 3.18515 5.26388 5.46943 5.19553 5.07455 4.41638 3.27981 3.56136 3.22528 1.65859 70 a - - -

2.68618 4.42225 2.77519 2.73123 3.46354 2.40513 3.72494 3.29354 2.67741 2.69355 4.24690 2.90347 2.73739 3.18146 2.89801 2.37887 2.77519 2.98518 4.58477 3.61503

0.00217 6.52953 7.25188 0.61958 0.77255 0.48576 0.95510

48 1.64358 6.21314 2.87506 3.53963 5.51879 4.59478 4.78514 4.18680 2.41967 3.17822 3.18681 4.07976 4.98697 2.99804 1.01114 3.48830 4.03579 4.03361 6.66959 5.28528 71 r - - -

2.68618 4.42225 2.77519 2.73123 3.46354 2.40513 3.72494 3.29354 2.67741 2.69355 4.24690 2.90347 2.73739 3.18146 2.89801 2.37887 2.77519 2.98518 4.58477 3.61503

0.00217 6.52953 7.25188 0.61958 0.77255 0.48576 0.95510

49 3.18534 4.08553 4.12632 2.39050 2.96522 4.60686 4.08483 3.06496 1.53057 2.13773 3.59114 2.65467 4.99853 2.58761 3.22001 2.65922 3.22760 4.50513 6.62122 3.41379 72 k - - -

2.68618 4.42225 2.77519 2.73123 3.46354 2.40513 3.72494 3.29354 2.67741 2.69355 4.24690 2.90347 2.73739 3.18146 2.89801 2.37887 2.77519 2.98518 4.58477 3.61503

0.00217 6.52953 7.25188 0.61958 0.77255 0.48576 0.95510

50 2.30197 3.54428 3.67067 2.01407 2.90055 4.56669 4.75405 5.12145 1.96868 4.14342 3.79080 2.75905 4.96009 2.76138 2.26753 2.54062 3.13187 4.00085 6.72991 2.59021 73 k - - -

2.68618 4.42225 2.77519 2.73123 3.46354 2.40513 3.72494 3.29354 2.67741 2.69355 4.24690 2.90347 2.73739 3.18146 2.89801 2.37887 2.77519 2.98518 4.58477 3.61503

0.00217 6.52953 7.25188 0.61958 0.77255 0.48576 0.95510

51 2.72293 4.17317 5.88830 5.27380 3.14217 4.18025 5.43779 2.30011 2.41067 1.41458 1.83227 3.77953 5.46870 3.81567 4.08422 4.41563 4.16908 2.90683 3.64810 2.51387 74 l - - -

2.68618 4.42225 2.77519 2.73123 3.46354 2.40513 3.72494 3.29354 2.67741 2.69355 4.24690 2.90347 2.73739 3.18146 2.89801 2.37887 2.77519 2.98518 4.58477 3.61503

0.00217 6.52953 7.25188 0.61958 0.77255 0.48576 0.95510

52 3.18385 4.36708 2.78550 1.28377 4.26856 3.46500 3.62696 3.10813 2.88519 2.99246 5.25477 3.12452 4.98357 3.89530 1.99068 3.52034 3.49813 3.05691 3.30623 3.57086 75 e - - -

2.68618 4.42225 2.77519 2.73123 3.46354 2.40513 3.72494 3.29354 2.67741 2.69355 4.24690 2.90347 2.73739 3.18146 2.89801 2.37887 2.77519 2.98518 4.58477 3.61503

0.00217 6.52953 7.25188 0.61958 0.77255 0.48576 0.95510

53 2.20213 6.28390 4.04921 2.55813 3.01912 1.43240 4.75751 3.83539 2.20575 4.05678 4.14256 2.39239 4.96288 3.02090 3.07655 3.00465 2.75925 4.67015 6.72150 4.21207 76 g - - -

2.68618 4.42225 2.77519 2.73123 3.46354 2.40513 3.72494 3.29354 2.67741 2.69355 4.24690 2.90347 2.73739 3.18146 2.89801 2.37887 2.77519 2.98518 4.58477 3.61503

0.10142 6.52953 2.35406 0.61958 0.77255 0.48576 0.95510

54 2.25523 4.70531 4.00750 1.56266 5.45679 3.18680 2.25046 3.67307 3.45169 2.55029 3.44337 2.76848 2.89417 3.61567 3.01795 2.46814 3.57322 4.51830 6.60052 3.84702 77 e - - -

2.68618 4.42225 2.77519 2.73123 3.46354 2.40513 3.72494 3.29354 2.67741 2.69355 4.24690 2.90347 2.73739 3.18146 2.89801 2.37887 2.77519 2.98518 4.58477 3.61503

0.00161 6.42973 * 0.61958 0.77255 0.00000 *

//

**File S2 SmAP2_domain_new.hmm**

HMMER3/f [3.1b1 | May 2013]

NAME SmAP2_domain

LENG 59

ALPH amino

RF no

MM no

CONS yes

CS no

MAP yes

DATE Tue Jul 23 05:06:49 2019

NSEQ 39

EFFN 0.861694

CKSUM 3201926944

STATS LOCAL MSV -8.2358 0.71905

STATS LOCAL VITERBI -9.0054 0.71905

STATS LOCAL FORWARD -4.0315 0.71905

HMM A C D E F G H I K L M N P Q R S T V W Y

m->m m->i m->d i->m i->i d->m d->d

COMPO 2.39223 4.39192 2.84651 2.79689 3.32158 2.43438 3.43693 3.29124 2.63211 2.77656 4.09471 3.27635 3.96786 3.06053 2.51401 2.85460 2.91441 2.95118 3.66937 2.82815

2.68622 4.42229 2.77523 2.73127 3.46358 2.40517 3.72498 3.29358 2.67745 2.69359 4.24694 2.90351 2.73744 3.18150 2.89805 2.37846 2.77523 2.98522 4.58481 3.61507

0.33441 1.34705 3.71995 0.21036 1.66226 0.00000 *

1 2.68649 4.53952 3.35515 2.79417 3.67977 3.57444 3.78154 2.09783 2.55915 2.65013 3.60058 3.23241 3.96292 2.17321 2.67417 2.83979 2.91668 2.66745 5.08683 3.83324 2 i - - -

2.68618 4.42225 2.77519 2.73123 3.46354 2.40513 3.72494 3.29354 2.67741 2.69355 4.24690 2.90347 2.73739 3.18146 2.89801 2.37887 2.77519 2.98518 4.58477 3.61503

0.02505 4.09511 4.81745 0.61958 0.77255 0.50447 0.92590

2 3.70875 4.96097 4.61259 4.30823 1.71223 4.42409 3.51875 3.41945 4.15677 2.74731 4.02352 4.09917 4.74501 4.15492 4.21181 3.80394 3.93617 3.34276 3.66391 0.83454 3 y - - -

2.68618 4.42225 2.77519 2.73123 3.46354 2.40513 3.72494 3.29354 2.67741 2.69355 4.24690 2.90347 2.73739 3.18146 2.89801 2.37887 2.77519 2.98518 4.58477 3.61503

0.02505 4.09511 4.81745 0.61958 0.77255 0.50447 0.92590

3 3.27348 5.08571 3.78847 3.25998 4.49767 3.71119 4.00994 4.05981 2.36004 3.53541 4.53466 3.60008 4.20737 3.22348 0.68595 3.34996 3.52708 3.77532 5.49299 4.38199 4 R - - -

2.68618 4.42225 2.77519 2.73123 3.46354 2.40513 3.72494 3.29354 2.67741 2.69355 4.24690 2.90347 2.73739 3.18146 2.89801 2.37887 2.77519 2.98518 4.58477 3.61503

0.02505 4.09511 4.81745 0.61958 0.77255 0.50447 0.92590

4 2.96591 4.71339 3.68481 3.61534 4.74245 0.45559 4.71653 4.40163 3.82658 4.02592 5.00750 3.84991 4.11126 4.13350 4.03291 3.14510 3.46444 3.88691 5.77318 4.85379 5 G - - -

2.68618 4.42225 2.77519 2.73123 3.46354 2.40513 3.72494 3.29354 2.67741 2.69355 4.24690 2.90347 2.73739 3.18146 2.89801 2.37887 2.77519 2.98518 4.58477 3.61503

0.02505 4.09511 4.81745 0.61958 0.77255 0.50447 0.92590

5 2.98759 4.47507 4.42918 4.10471 3.72309 3.94927 4.87129 2.06670 3.97607 2.37480 3.65954 4.25736 4.50142 4.32234 4.16639 3.47122 3.34625 0.78403 5.54335 4.28651 6 v - - -

2.68618 4.42225 2.77519 2.73123 3.46354 2.40513 3.72494 3.29354 2.67741 2.69355 4.24690 2.90347 2.73739 3.18146 2.89801 2.37887 2.77519 2.98518 4.58477 3.61503

0.02505 4.09511 4.81745 0.61958 0.77255 0.50447 0.92590

6 2.34181 4.25385 3.49169 3.21997 4.21567 3.06510 4.26117 3.49970 3.20626 3.30068 4.19573 3.38882 3.79860 3.55329 3.48919 2.24941 1.08684 3.06337 5.60606 4.37197 7 t - - -

2.68618 4.42225 2.77519 2.73123 3.46354 2.40513 3.72494 3.29354 2.67741 2.69355 4.24690 2.90347 2.73739 3.18146 2.89801 2.37887 2.77519 2.98518 4.58477 3.61503

0.02505 4.09511 4.81745 0.61958 0.77255 0.50447 0.92590

7 2.87719 4.68257 3.57344 3.02218 2.31273 3.68233 3.79633 3.16124 2.61270 2.69592 3.74543 3.40059 4.09478 3.16963 1.49472 3.01878 3.11917 2.97823 4.79708 3.33146 8 r - - -

2.68618 4.42225 2.77519 2.73123 3.46354 2.40513 3.72494 3.29354 2.67741 2.69355 4.24690 2.90347 2.73739 3.18146 2.89801 2.37887 2.77519 2.98518 4.58477 3.61503

0.02505 4.09511 4.81745 0.61958 0.77255 0.47424 0.97380

8 3.22421 4.92176 3.63911 3.24740 2.42109 3.92486 1.43127 3.55475 3.07321 2.99788 4.07941 3.54274 4.32652 3.45540 3.34871 3.28204 3.45853 3.35688 4.05058 1.78144 9 h - - -

2.68618 4.42225 2.77519 2.73123 3.46354 2.40513 3.72494 3.29354 2.67741 2.69355 4.24690 2.90347 2.73739 3.18146 2.89801 2.37887 2.77519 2.98518 4.58477 3.61503

0.02464 4.11152 4.83387 0.61958 0.77255 0.48576 0.95510

9 3.29509 5.10423 3.81203 3.28121 4.52056 3.72834 4.02606 4.08472 2.37222 3.55733 4.55694 3.61962 4.22444 3.23948 0.66769 3.37128 3.54802 3.79970 5.50960 4.40221 10 R - - -

2.68618 4.42225 2.77519 2.73123 3.46354 2.40513 3.72494 3.29354 2.67741 2.69355 4.24690 2.90347 2.73739 3.18146 2.89801 2.37887 2.77519 2.98518 4.58477 3.61503

0.02464 4.11152 4.83387 0.61958 0.77255 0.48576 0.95510

10 3.02970 4.73821 3.80203 3.22603 3.19682 3.77082 3.81292 3.34172 2.60496 2.88842 3.89623 3.54451 4.18382 3.26419 1.99651 3.18146 3.26048 3.13657 1.48113 3.20065 11 w - - -

2.68618 4.42225 2.77519 2.73123 3.46354 2.40513 3.72494 3.29354 2.67741 2.69355 4.24690 2.90347 2.73739 3.18146 2.89801 2.37887 2.77519 2.98518 4.58477 3.61503

0.02464 4.11152 4.83387 0.61958 0.77255 0.48576 0.95510

11 2.56018 4.40343 3.66103 3.43280 4.20050 3.23269 4.42922 3.35781 3.38055 3.21259 4.25300 3.59753 3.95780 3.76469 3.62296 2.76483 0.82269 3.02607 5.61653 4.41925 12 t - - -

2.68618 4.42225 2.77519 2.73123 3.46354 2.40513 3.72494 3.29354 2.67741 2.69355 4.24690 2.90347 2.73739 3.18146 2.89801 2.37887 2.77519 2.98518 4.58477 3.61503

0.02464 4.11152 4.83387 0.61958 0.77255 0.48576 0.95510

12 2.99020 4.73507 3.71238 3.64545 4.77038 0.44008 4.74466 4.43453 3.85886 4.05637 5.03875 3.87760 4.13337 4.16453 4.06247 3.17000 3.48999 3.91660 5.79581 4.88261 13 G - - -

2.68618 4.42225 2.77519 2.73123 3.46354 2.40513 3.72494 3.29354 2.67741 2.69355 4.24690 2.90347 2.73739 3.18146 2.89801 2.37887 2.77519 2.98518 4.58477 3.61503

0.02464 4.11152 4.83387 0.61958 0.77255 0.48576 0.95510

13 3.29509 5.10423 3.81203 3.28121 4.52056 3.72834 4.02606 4.08472 2.37222 3.55733 4.55694 3.61962 4.22444 3.23948 0.66769 3.37128 3.54802 3.79970 5.50960 4.40221 14 R - - -

2.68618 4.42225 2.77519 2.73123 3.46354 2.40513 3.72494 3.29354 2.67741 2.69355 4.24690 2.90347 2.73739 3.18146 2.89801 2.37887 2.77519 2.98518 4.58477 3.61503

0.02464 4.11152 4.83387 0.61958 0.77255 0.48576 0.95510

14 3.85371 4.99053 4.88322 4.62435 1.52148 4.55538 3.46290 3.56974 4.43949 2.88129 4.13335 4.19011 4.83883 4.29028 4.38457 3.92179 4.06157 3.47222 2.14126 1.05697 15 y - - -

2.68618 4.42225 2.77519 2.73123 3.46354 2.40513 3.72494 3.29354 2.67741 2.69355 4.24690 2.90347 2.73739 3.18146 2.89801 2.37887 2.77519 2.98518 4.58477 3.61503

0.02464 4.11152 4.83387 0.61958 0.77255 0.48576 0.95510

15 3.17999 5.31982 2.61677 0.72743 4.69942 3.44429 4.06096 4.20233 2.97351 3.78381 4.75460 3.09700 4.06244 3.27624 3.40201 3.14210 3.48841 3.86410 5.82547 4.55683 16 e - - -

2.68618 4.42225 2.77519 2.73123 3.46354 2.40513 3.72494 3.29354 2.67741 2.69355 4.24690 2.90347 2.73739 3.18146 2.89801 2.37887 2.77519 2.98518 4.58477 3.61503

0.02464 4.11152 4.83387 0.61958 0.77255 0.48576 0.95510

16 1.07309 4.19787 3.57681 3.30647 4.34588 2.99430 4.34210 3.69597 3.34807 3.44849 4.29681 3.41000 3.75727 3.63439 3.63531 1.77929 2.76923 3.16969 5.71397 4.50620 17 a - - -

2.68618 4.42225 2.77519 2.73123 3.46354 2.40513 3.72494 3.29354 2.67741 2.69355 4.24690 2.90347 2.73739 3.18146 2.89801 2.37887 2.77519 2.98518 4.58477 3.61503

0.02464 4.11152 4.83387 0.61958 0.77255 0.48576 0.95510

17 3.29281 5.05583 3.39339 3.18377 3.47993 3.67338 0.72462 4.03322 3.02158 3.49388 4.56079 3.58077 4.24222 3.55500 3.26954 3.36329 3.60706 3.76404 4.94013 3.42540 18 h - - -

2.68618 4.42225 2.77519 2.73123 3.46354 2.40513 3.72494 3.29354 2.67741 2.69355 4.24690 2.90347 2.73739 3.18146 2.89801 2.37887 2.77519 2.98518 4.58477 3.61503

0.02464 4.11152 4.83387 0.61958 0.77255 0.48576 0.95510

18 3.36435 4.67559 4.94920 4.42052 3.18957 4.63779 4.98147 1.66090 4.24607 0.92226 2.99933 4.67217 4.83654 4.40441 4.37247 4.01706 3.59829 2.12497 5.28392 4.16689 19 l - - -

2.68618 4.42225 2.77519 2.73123 3.46354 2.40513 3.72494 3.29354 2.67741 2.69355 4.24690 2.90347 2.73739 3.18146 2.89801 2.37887 2.77519 2.98518 4.58477 3.61503

0.02464 4.11152 4.83387 0.61958 0.77255 0.48576 0.95510

19 3.76396 5.04771 4.47657 4.24650 2.95307 4.01505 4.22702 3.80532 3.96767 3.15589 4.42680 4.34924 4.55396 4.34263 4.05160 3.96515 4.07797 3.70197 0.51572 2.94375 20 W - - -

2.68618 4.42225 2.77519 2.73123 3.46354 2.40513 3.72494 3.29354 2.67741 2.69355 4.24690 2.90347 2.73739 3.18146 2.89801 2.37887 2.77519 2.98518 4.58477 3.61503

0.02464 4.11152 4.83387 0.61958 0.77255 0.48576 0.95510

20 3.26524 5.40931 0.62762 2.51720 4.82286 3.40882 4.16665 4.46948 3.29977 4.03746 5.02733 3.08317 4.07898 3.41071 3.84358 3.20502 3.61112 4.08522 5.94284 4.66465 21 D - - -

2.68618 4.42225 2.77519 2.73123 3.46354 2.40513 3.72494 3.29354 2.67741 2.69355 4.24690 2.90347 2.73739 3.18146 2.89801 2.37887 2.77519 2.98518 4.58477 3.61503

0.16921 4.11152 1.97122 0.61958 0.77255 0.48576 0.95510

21 2.81540 5.05770 2.71563 2.42325 4.42316 3.37362 3.67838 3.88535 1.76100 3.42046 4.26777 1.82438 3.87657 2.83474 2.63063 2.80198 3.07029 3.52167 5.53362 4.20600 22 k - - -

2.68618 4.42225 2.77519 2.73123 3.46354 2.40513 3.72494 3.29354 2.67741 2.69355 4.24690 2.90347 2.73739 3.18146 2.89801 2.37887 2.77519 2.98518 4.58477 3.61503

0.02842 3.97073 4.69307 0.61958 0.77255 0.62753 0.76337

22 2.12206 4.35630 3.02689 2.76649 4.25884 3.03574 3.99116 3.69123 2.89820 3.35418 4.18641 2.61604 3.71947 3.21620 3.27314 1.38664 2.78032 3.20912 5.57081 4.28907 23 s - - -

2.68618 4.42225 2.77519 2.73123 3.46354 2.40513 3.72494 3.29354 2.67741 2.69355 4.24690 2.90347 2.73739 3.18146 2.89801 2.37887 2.77519 2.98518 4.58477 3.61503

0.02842 3.97073 4.69307 0.61958 0.77255 0.62753 0.76337

23 2.20240 1.76505 3.86331 3.46807 3.89480 2.99495 4.26096 3.15764 3.35607 2.97881 3.87037 3.46815 3.72329 3.64121 3.58246 1.93378 2.34944 2.77121 5.33920 4.15118 24 c - - -

2.68618 4.42225 2.77519 2.73123 3.46354 2.40513 3.72494 3.29354 2.67741 2.69355 4.24690 2.90347 2.73739 3.18146 2.89801 2.37887 2.77519 2.98518 4.58477 3.61503

0.02842 3.97073 4.69307 0.61958 0.77255 0.62753 0.76337

24 2.80381 4.79397 3.37093 2.72595 3.97528 3.58388 3.62385 3.42652 1.93082 3.01008 3.88216 3.15379 3.94876 2.73560 1.80811 2.87370 3.00106 3.15329 2.97601 3.91517 25 r - - -

2.68618 4.42225 2.77519 2.73123 3.46354 2.40513 3.72494 3.29354 2.67741 2.69355 4.24690 2.90347 2.73739 3.18146 2.89801 2.37887 2.77519 2.98518 4.58477 3.61503

0.02842 3.97073 4.69307 0.61958 0.77255 0.62753 0.76337

25 2.83586 5.07081 3.04358 2.53564 4.44936 3.49713 3.59885 3.85203 1.84873 3.34510 4.17562 2.20352 3.90585 2.73288 1.84870 2.82585 3.04352 3.50346 5.45030 4.18417 26 r - - -

2.68618 4.42225 2.77519 2.73123 3.46354 2.40513 3.72494 3.29354 2.67741 2.69355 4.24690 2.90347 2.73739 3.18146 2.89801 2.37887 2.77519 2.98518 4.58477 3.61503

0.02842 3.97073 4.69307 0.61958 0.77255 0.62753 0.76337

26 2.78208 5.16565 2.39163 1.44535 4.50864 3.26482 3.70022 3.97912 2.56117 3.51849 4.34628 2.41295 3.18908 2.85613 3.04705 2.73323 3.05611 3.58637 5.69065 4.27731 27 e - - -

2.68618 4.42225 2.77519 2.73123 3.46354 2.40513 3.72494 3.29354 2.67741 2.69355 4.24690 2.90347 2.73739 3.18146 2.89801 2.37887 2.77519 2.98518 4.58477 3.61503

0.02842 3.97073 4.69307 0.61958 0.77255 0.62753 0.76337

27 2.37401 4.27437 3.32493 2.89068 3.86652 1.90505 3.93829 2.66407 2.87432 2.92594 3.80040 3.21561 3.77483 3.20296 3.23008 2.28912 2.48045 2.83900 5.25742 4.01097 28 g - - -

2.68618 4.42225 2.77519 2.73123 3.46354 2.40513 3.72494 3.29354 2.67741 2.69355 4.24690 2.90347 2.73739 3.18146 2.89801 2.37887 2.77519 2.98518 4.58477 3.61503

0.02842 3.97073 4.69307 0.61958 0.77255 0.62753 0.76337

28 2.96113 4.94838 2.97026 2.72716 4.12979 3.43234 3.88611 3.77620 2.54131 3.26875 4.29447 3.18752 3.98761 1.06905 2.82330 3.00502 3.25832 3.49996 5.38471 4.08519 29 q - - -

2.68618 4.42225 2.77519 2.73123 3.46354 2.40513 3.72494 3.29354 2.67741 2.69355 4.24690 2.90347 2.73739 3.18146 2.89801 2.37887 2.77519 2.98518 4.58477 3.61503

0.02842 3.97073 4.69307 0.61958 0.77255 0.62753 0.76337

29 2.39224 4.63741 2.96550 2.50902 4.10916 3.27354 3.70549 3.52337 2.49513 3.13412 3.95435 2.40378 3.77331 2.87288 2.66504 2.13111 2.41118 3.15688 5.36807 4.05623 30 s - - -

2.68618 4.42225 2.77519 2.73123 3.46354 2.40513 3.72494 3.29354 2.67741 2.69355 4.24690 2.90347 2.73739 3.18146 2.89801 2.37887 2.77519 2.98518 4.58477 3.61503

0.02842 3.97073 4.69307 0.61958 0.77255 0.62753 0.76337

30 3.04974 5.13008 3.47263 2.81225 4.57566 3.65787 3.61185 3.92603 1.57145 3.37917 4.25930 3.20562 4.03234 2.75181 1.33877 3.05312 3.21862 3.61570 5.42584 4.26309 31 r - - -

2.68618 4.42225 2.77519 2.73123 3.46354 2.40513 3.72494 3.29354 2.67741 2.69355 4.24690 2.90347 2.73739 3.18146 2.89801 2.37887 2.77519 2.98518 4.58477 3.61503

0.02842 3.97073 4.69307 0.61958 0.77255 0.40634 1.09687

31 2.72485 2.63985 3.44095 2.89643 4.06193 3.48238 3.85237 3.36023 1.33520 3.06771 3.97232 3.28600 3.96934 3.06036 2.73098 2.84659 3.01277 3.07147 5.32698 4.09081 32 k - - -

2.68618 4.42225 2.77519 2.73123 3.46354 2.40513 3.72494 3.29354 2.67741 2.69355 4.24690 2.90347 2.73739 3.18146 2.89801 2.37887 2.77519 2.98518 4.58477 3.61503

0.02464 4.11152 4.83387 0.61958 0.77255 0.48576 0.95510

32 2.99020 4.73507 3.71238 3.64545 4.77038 0.44008 4.74466 4.43453 3.85886 4.05637 5.03875 3.87760 4.13337 4.16453 4.06247 3.17000 3.48999 3.91660 5.79581 4.88261 33 G - - -

2.68618 4.42225 2.77519 2.73123 3.46354 2.40513 3.72494 3.29354 2.67741 2.69355 4.24690 2.90347 2.73739 3.18146 2.89801 2.37887 2.77519 2.98518 4.58477 3.61503

0.02464 4.11152 4.83387 0.61958 0.77255 0.48576 0.95510

33 3.16411 5.26601 3.61985 2.90151 4.75789 3.75969 3.65828 4.08738 1.33638 3.50019 4.37513 3.28503 4.11513 2.78988 1.35905 3.15281 3.31532 3.77003 5.51483 4.37258 34 k - - -

2.68618 4.42225 2.77519 2.73123 3.46354 2.40513 3.72494 3.29354 2.67741 2.69355 4.24690 2.90347 2.73739 3.18146 2.89801 2.37887 2.77519 2.98518 4.58477 3.61503

0.02464 4.11152 4.83387 0.61958 0.77255 0.48576 0.95510

34 3.09517 5.07360 3.08509 2.85082 4.27294 3.53984 4.00870 3.94775 2.65643 3.42535 4.45340 3.31006 4.10113 0.86169 2.93285 3.13602 3.39521 3.66450 5.50510 4.21795 35 q - - -

2.68618 4.42225 2.77519 2.73123 3.46354 2.40513 3.72494 3.29354 2.67741 2.69355 4.24690 2.90347 2.73739 3.18146 2.89801 2.37887 2.77519 2.98518 4.58477 3.61503

0.10365 4.11152 2.50011 0.61958 0.77255 0.48576 0.95510

35 2.93754 4.43842 4.34728 4.01811 3.67332 3.88881 4.79336 2.05588 3.88839 2.33392 3.61644 4.18118 4.44226 4.23774 4.08488 3.40467 3.29639 0.84273 5.48425 4.22477 36 v - - -

2.68618 4.42225 2.77519 2.73123 3.46354 2.40513 3.72494 3.29354 2.67741 2.69355 4.24690 2.90347 2.73739 3.18146 2.89801 2.37887 2.77519 2.98518 4.58477 3.61503

0.02664 4.03451 4.75685 0.61958 0.77255 0.56823 0.83593

36 3.48876 4.91181 4.08102 3.84325 2.31680 4.02168 3.70574 3.47622 3.69626 2.88764 4.13489 3.92826 4.49291 3.96293 3.84563 3.60829 3.77688 3.35477 3.96591 0.73369 37 y - - -

2.68618 4.42225 2.77519 2.73123 3.46354 2.40513 3.72494 3.29354 2.67741 2.69355 4.24690 2.90347 2.73739 3.18146 2.89801 2.37887 2.77519 2.98518 4.58477 3.61503

0.02664 4.03451 4.75685 0.61958 0.77255 0.56823 0.83593

37 3.34118 4.75700 4.44902 4.09236 3.20452 4.18639 4.69747 2.42029 3.85853 0.69865 3.18181 4.35206 4.59566 4.18789 4.00696 3.78031 3.62906 2.49917 5.13973 3.89954 38 l - - -

2.68618 4.42225 2.77519 2.73123 3.46354 2.40513 3.72494 3.29354 2.67741 2.69355 4.24690 2.90347 2.73739 3.18146 2.89801 2.37887 2.77519 2.98518 4.58477 3.61503

0.02664 4.03451 4.75685 0.61958 0.77255 0.43745 1.03756

38 2.99020 4.73507 3.71238 3.64545 4.77038 0.44008 4.74466 4.43453 3.85886 4.05637 5.03875 3.87760 4.13337 4.16453 4.06247 3.17000 3.48999 3.91660 5.79581 4.88261 39 G - - -

2.68618 4.42225 2.77519 2.73123 3.46354 2.40513 3.72494 3.29354 2.67741 2.69355 4.24690 2.90347 2.73739 3.18146 2.89801 2.37887 2.77519 2.98518 4.58477 3.61503

0.02464 4.11152 4.83387 0.61958 0.77255 0.48576 0.95510

39 1.59914 4.24990 3.46480 3.26673 4.46892 1.21575 4.37942 3.84590 3.39940 3.57820 4.42350 3.40343 3.77606 3.66911 3.69228 2.49550 2.82137 3.28196 5.79966 4.61061 40 g - - -

2.68618 4.42225 2.77519 2.73123 3.46354 2.40513 3.72494 3.29354 2.67741 2.69355 4.24690 2.90347 2.73739 3.18146 2.89801 2.37887 2.77519 2.98518 4.58477 3.61503

0.02464 4.11152 4.83387 0.61958 0.77255 0.48576 0.95510

40 3.70430 4.95626 4.60305 4.27253 1.48070 4.42997 2.96239 3.54264 4.12339 2.89202 4.10742 4.06575 4.73722 4.11869 4.18745 3.78056 3.91914 3.42297 3.62655 0.97553 41 y - - -

2.68618 4.42225 2.77519 2.73123 3.46354 2.40513 3.72494 3.29354 2.67741 2.69355 4.24690 2.90347 2.73739 3.18146 2.89801 2.37887 2.77519 2.98518 4.58477 3.61503

0.02464 4.11152 4.83387 0.61958 0.77255 0.48576 0.95510

41 3.26524 5.40931 0.62762 2.51720 4.82286 3.40882 4.16665 4.46948 3.29977 4.03746 5.02733 3.08317 4.07898 3.41071 3.84358 3.20502 3.61112 4.08522 5.94284 4.66465 42 D - - -

2.68618 4.42225 2.77519 2.73123 3.46354 2.40513 3.72494 3.29354 2.67741 2.69355 4.24690 2.90347 2.73739 3.18146 2.89801 2.37887 2.77519 2.98518 4.58477 3.61503

0.02464 4.11152 4.83387 0.61958 0.77255 0.48576 0.95510

42 2.61836 4.94871 2.68471 2.25797 4.22985 3.39780 3.60536 3.66882 2.02770 3.22495 3.30019 2.65992 3.80048 2.73195 2.83713 2.61586 2.40036 3.30461 5.42189 4.05690 43 k - - -

2.68618 4.42225 2.77519 2.73123 3.46354 2.40513 3.72494 3.29354 2.67741 2.69355 4.24690 2.90347 2.73739 3.18146 2.89801 2.37887 2.77519 2.98518 4.58477 3.61503

0.02464 4.11152 4.83387 0.61958 0.77255 0.48576 0.95510

43 2.09176 5.00970 2.58437 1.32161 4.45813 3.31553 3.82899 3.80446 2.69080 3.45683 4.32485 2.92480 3.88646 2.99990 3.14933 2.78109 3.08267 3.44327 5.69652 4.32574 44 e - - -

2.68618 4.42225 2.77519 2.73123 3.46354 2.40513 3.72494 3.29354 2.67741 2.69355 4.24690 2.90347 2.73739 3.18146 2.89801 2.37887 2.77519 2.98518 4.58477 3.61503

0.02464 4.11152 4.83387 0.61958 0.77255 0.48576 0.95510

44 2.82432 5.32543 2.20408 1.48785 4.60830 3.32845 2.57926 4.10844 2.56208 3.60694 4.40634 2.79079 3.85105 2.72912 3.07944 2.75266 3.07873 3.69612 5.75785 4.31463 45 e - - -

2.68618 4.42225 2.77519 2.73123 3.46354 2.40513 3.72494 3.29354 2.67741 2.69355 4.24690 2.90347 2.73739 3.18146 2.89801 2.37887 2.77519 2.98518 4.58477 3.61503

0.02464 4.11152 4.83387 0.61958 0.77255 0.48576 0.95510

45 1.89213 4.69953 3.07059 2.60082 4.19837 3.34139 3.75985 3.58200 1.79104 3.20386 4.03126 3.05972 3.84180 2.92570 2.86420 2.46023 2.68723 3.21850 5.43192 4.13525 46 k - - -

2.68618 4.42225 2.77519 2.73123 3.46354 2.40513 3.72494 3.29354 2.67741 2.69355 4.24690 2.90347 2.73739 3.18146 2.89801 2.37887 2.77519 2.98518 4.58477 3.61503

0.02464 4.11152 4.83387 0.61958 0.77255 0.48576 0.95510

46 0.71740 4.32900 3.72210 3.53251 4.30684 3.14567 4.52389 3.46939 3.55540 3.33931 4.35019 3.61630 3.90819 3.87557 3.78454 2.68000 2.97434 3.08156 5.70957 4.53902 47 a - - -

2.68618 4.42225 2.77519 2.73123 3.46354 2.40513 3.72494 3.29354 2.67741 2.69355 4.24690 2.90347 2.73739 3.18146 2.89801 2.37887 2.77519 2.98518 4.58477 3.61503

0.02464 4.11152 4.83387 0.61958 0.77255 0.48576 0.95510

47 0.79752 4.25119 3.61223 3.40991 4.38269 2.81224 4.44221 3.65783 3.46810 3.47770 4.37416 3.49759 3.81815 3.76456 3.72168 2.54984 2.86017 3.17121 5.74344 4.56816 48 a - - -

2.68618 4.42225 2.77519 2.73123 3.46354 2.40513 3.72494 3.29354 2.67741 2.69355 4.24690 2.90347 2.73739 3.18146 2.89801 2.37887 2.77519 2.98518 4.58477 3.61503

0.02464 4.11152 4.83387 0.61958 0.77255 0.48576 0.95510

48 3.06048 5.12154 3.45347 2.85103 4.27378 3.68613 2.74889 3.91416 2.04608 3.38325 4.27149 3.25716 4.07950 2.86170 1.16768 3.07562 3.24961 3.60760 5.34738 4.06555 49 r - - -

2.68618 4.42225 2.77519 2.73123 3.46354 2.40513 3.72494 3.29354 2.67741 2.69355 4.24690 2.90347 2.73739 3.18146 2.89801 2.37887 2.77519 2.98518 4.58477 3.61503

0.02464 4.11152 4.83387 0.61958 0.77255 0.48576 0.95510

49 1.11669 4.20693 3.76358 3.37601 4.06473 3.14009 4.31383 3.06642 3.31861 3.03238 3.98400 3.50004 3.84711 3.62504 3.60223 2.57216 2.36926 2.47275 5.52823 4.32477 50 a - - -

2.68618 4.42225 2.77519 2.73123 3.46354 2.40513 3.72494 3.29354 2.67741 2.69355 4.24690 2.90347 2.73739 3.18146 2.89801 2.37887 2.77519 2.98518 4.58477 3.61503

0.02464 4.11152 4.83387 0.61958 0.77255 0.48576 0.95510

50 3.61056 5.00188 4.20015 3.98022 2.34417 4.11567 3.75735 3.58870 3.83139 2.97919 4.23977 4.03049 4.58663 4.07857 3.96329 3.72084 3.89993 3.47131 3.99503 0.64513 51 Y - - -

2.68618 4.42225 2.77519 2.73123 3.46354 2.40513 3.72494 3.29354 2.67741 2.69355 4.24690 2.90347 2.73739 3.18146 2.89801 2.37887 2.77519 2.98518 4.58477 3.61503

0.02464 4.11152 4.83387 0.61958 0.77255 0.48576 0.95510

51 3.26524 5.40931 0.62762 2.51720 4.82286 3.40882 4.16665 4.46948 3.29977 4.03746 5.02733 3.08317 4.07898 3.41071 3.84358 3.20502 3.61112 4.08522 5.94284 4.66465 52 D - - -

2.68618 4.42225 2.77519 2.73123 3.46354 2.40513 3.72494 3.29354 2.67741 2.69355 4.24690 2.90347 2.73739 3.18146 2.89801 2.37887 2.77519 2.98518 4.58477 3.61503

0.02464 4.11152 4.83387 0.61958 0.77255 0.48576 0.95510

52 2.75611 4.60064 3.41521 2.86367 3.71986 3.59366 3.78217 3.11536 2.45566 1.68437 3.70149 2.93575 3.99919 3.03137 2.17948 2.90219 2.98863 2.88962 5.09470 3.82560 53 l - - -

2.68618 4.42225 2.77519 2.73123 3.46354 2.40513 3.72494 3.29354 2.67741 2.69355 4.24690 2.90347 2.73739 3.18146 2.89801 2.37887 2.77519 2.98518 4.58477 3.61503

0.02464 4.11152 4.83387 0.61958 0.77255 0.48576 0.95510

53 1.13046 4.48249 3.20992 2.90006 3.94457 3.26173 3.21296 3.46748 2.88541 3.14782 4.05198 3.25464 3.88414 3.27701 3.21556 2.69453 2.93274 3.11536 5.33995 4.01006 54 a - - -

2.68618 4.42225 2.77519 2.73123 3.46354 2.40513 3.72494 3.29354 2.67741 2.69355 4.24690 2.90347 2.73739 3.18146 2.89801 2.37887 2.77519 2.98518 4.58477 3.61503

0.06839 4.11152 3.00140 0.61958 0.77255 0.48576 0.95510

54 0.76393 4.30137 3.67249 3.47806 4.25815 3.11967 4.47541 3.41383 3.49966 3.28594 4.29975 3.57466 3.87793 3.82351 3.73353 2.65180 2.94303 3.03382 5.66544 4.48911 55 a - - -

2.68618 4.42225 2.77519 2.73123 3.46354 2.40513 3.72494 3.29354 2.67741 2.69355 4.24690 2.90347 2.73739 3.18146 2.89801 2.37887 2.77519 2.98518 4.58477 3.61503

0.02573 4.06886 4.79121 0.61958 0.77255 0.53306 0.88384

55 3.32113 4.64127 4.88309 4.35536 3.18061 4.58582 4.93096 1.63940 4.18121 0.97140 2.99623 4.61094 4.79618 4.35523 4.31814 3.96218 3.55665 2.08917 5.26003 4.13234 56 l - - -

2.68618 4.42225 2.77519 2.73123 3.46354 2.40513 3.72494 3.29354 2.67741 2.69355 4.24690 2.90347 2.73739 3.18146 2.89801 2.37887 2.77519 2.98518 4.58477 3.61503

0.02573 4.06886 4.79121 0.61958 0.77255 0.53306 0.88384

56 3.16197 5.12376 3.35037 2.94063 4.55540 3.62517 3.88329 3.99820 0.79769 3.52257 4.47764 3.35481 4.11510 3.06666 2.48904 3.19358 3.40270 3.69644 5.53240 4.37555 57 k - - -

2.68618 4.42225 2.77519 2.73123 3.46354 2.40513 3.72494 3.29354 2.67741 2.69355 4.24690 2.90347 2.73739 3.18146 2.89801 2.37887 2.77519 2.98518 4.58477 3.61503

0.02573 4.06886 4.79121 0.61958 0.77255 0.53306 0.88384

57 3.70765 4.93498 4.66023 4.35874 1.46085 4.43488 3.49339 3.39840 4.20407 2.72017 3.99369 4.10516 4.74419 4.16745 4.23536 3.80834 3.92983 3.32366 3.62996 0.96879 58 y - - -

2.68618 4.42225 2.77519 2.73123 3.46354 2.40513 3.72494 3.29354 2.67741 2.69355 4.24690 2.90347 2.73739 3.18146 2.89801 2.37887 2.77519 2.98518 4.58477 3.61503

0.02573 4.06886 4.79121 0.61958 0.77255 0.53306 0.88384

58 2.98705 4.70502 3.75408 3.17691 3.18894 3.73722 3.78405 3.30312 2.57169 2.85487 3.85769 3.50230 4.14816 3.22397 1.98447 3.13815 3.21730 3.09645 1.59454 3.19548 59 w - - -

2.68618 4.42225 2.77519 2.73123 3.46354 2.40513 3.72494 3.29354 2.67741 2.69355 4.24690 2.90347 2.73739 3.18146 2.89801 2.37887 2.77519 2.98518 4.58477 3.61503

0.02573 4.06886 4.79121 0.61958 0.77255 0.53306 0.88384

59 2.92761 4.67913 3.64125 3.56778 4.69809 0.48154 4.67196 4.34950 3.77551 3.97763 4.95801 3.80616 4.07630 4.08444 3.98605 3.10583 3.42413 3.83988 5.73718 4.80807 60 G - - -

2.68618 4.42225 2.77519 2.73123 3.46354 2.40513 3.72494 3.29354 2.67741 2.69355 4.24690 2.90347 2.73739 3.18146 2.89801 2.37887 2.77519 2.98518 4.58477 3.61503

0.01739 4.06052 * 0.61958 0.77255 0.00000 *

//

File S3 SmAP2/ERF_CDS_confirmed.fa

>EVM0031439

ATGAATCCATCTTCATCAAAAGGTAAAAAGAAGCAACATCAGCAAGTACAGCAGGAACCT

GCAGGCACTGGATTAAGGTTTCTAGGTGTCAGGAGAAGGCCATGGGGAAGATATGCAGCA

GAGATAAGAGACCCTTCAACAAAAGAACGGCATTGGCTAGGCACCTTTGACACTGCCGAG

GAAGCTGCCTTGGCCTATGACAGAGCTGCTCGCTCCATGCGCGGTTCTCGTGCTCGCACA

AACTTTGTTTACTCAGACATGCCTGCTGGTTCTTCTGTCACATCCATTATTTCCCCTGAT

GAACAGCAATCTCTACAGCAGCAGCAGCAGCACCAGCACCAGCAACATGGTGGTAATGAT

AACAGCTTGTCTTCATTACTCTTCAATGTCGTCCCTTCACTCCATGATCATCAGCAGCAG

GACTCCACTCCCATTTTCAACCAGGATTATAGCTCCCAACGCCATTTGGTAGAGGGGTTT

TCTTCAACGACATCTGGTGGAGAGTTTTGGAGCTGCTCCAGTAATAATAACTCATGTAAG

CAACAGCTACAACATGCTATTCAAAACAATGTGCTTTCTCATGATTTTCCTTCTGATACT

TTTCACGGCTCGGGTTATAATACGGGTCAATGTGATTGGATAGATTCGTCAGCTTCGGGG

TTGCTGGGTTTTGAGGACCAGACAACAATGACTAATGGGCTCGAGTCTGTGGGGTGTAGC

AGTGGTTCGTATTTCGGGTTTGATTCTGGAGAGTACGTCCACAGTCCACTTTTTAGCAGG

ATGCCCCCAGTCTCAGATACAGCACCAGATGGTTTTGATTTGGGCTCCTCCTCTTATTTC

TTCTAG

>EVM0013188

ATGGAAGCATCACTCAATATACTATGTGATGCTCAAACTACCACCTTTTCCTTTTCCTCC

CCTGCCTCTTCTTCTTCTTCAATCACCTCTTCCCTCCCTTCTCCATTAATGAAGAGTCCC

AGAAAACAAGATCAGTCTGATCAAGACCCGAAAAACAATAATGAAAGTAACGAAAAGGCC

AAGAGTGGTGGAAATGCTGGAAGGCACCCATCCTACCGTGGAGTTAGAATGCGTCATTGG

GGCAAATGGGTGTCCGAAATCCGGCAACCAAAGAAGAAGTCGAGAATCTGGCTGGGGACT

TTCCCAACACCAGAAATGGCGGCTCGAGCTCACGATGTAGCTGCCCTTACAATTAAAGGT

CAGTCAGCTCACCTAAATTTCCCTGAACTCGCTCATGAGTTTCCACGTCCAGCCAGTTCA

TCTCCCAAAGATATTCAGGCCGCCGCTGCCCTAGCAGCTTCCTTGAGCTGCAAGACAAGT

CAAAAGGGCCGTGAGACCGAAGCTGGAGGGGAGCTGGTGCTTCCTCGCTCCCCGGGTAGT

GCTCTGGCATCTAATGAGACACAGGAGTACTCATTAAGCTCACCATTAAGAGATGATGAT

GGCGACGCGTTTATCGATTTGCCGGATATTTTGCAGGACACGAGCCATCAATTTTATGAA

TTTTGTTACAGGCCATCATGGCAGCTGGTTGGAGCCGAGACTTCTGATATCGGGTTCTGG

CATCACGAGGAGCCTTCTTTATGGGAATACCACTCAAGTAGAAACATCTATTGA

>EVM0020531

ATGTCAGCAGAAGTATCATCAGCCCTGGAGTTCATCAAGAACCACTTGTTTGGCGATCTT

CTTTCACCTCTTGCTACCTCCTCCTCTTCAGTTTCTTTTTATCAGTTTTCTACTAATACT

GAGATTTATACTAATGAGACTACCAGCTGCCGCTCACAGACTTCGACTTCTGCCTCTTCC

ACTGCATTTCCTGGTTTTTTCGATTCTCCTGACCCTAGTTTCTTCGAATTCACTTCCGTT

TTCAGTCCTGCTCAAGATAACAAGACCAATATCTTCGAGTTTGAAGCTAAACCTGAAATT

ATTGATCTCTCAACGCCAAACAGCAGCACTAACTCGTTCGAACTTGTGAAACCACAAATT

ATTTCTCAACCAAGCAACAATTTCTTCTATGAAGAATCAAGGCCCGGAGTTCAACCAACT

CGGAAACCGTCATTGAAAATATCGCTGCCCGGCAAAAAATCCGAATGGATTCAGTTTTCT

AACCAGGACTCGCAACACTTGGATGATAATTCTGGTGTTGCTGTAGAAGCGAAAAGGCAT

TACAGAGGAGTCCGACAAAGGCCATGGGGCAAGTATGCAGCTGAGATACGGGACCCGAAC

CGGAGAGGTTCACGTGTGTGGCTAGGAACCTTCGATACAGCTCTCGAAGCTGCCAGAGCC

TATGATCGAGCTGCCTTCAAGCTGCGTGGGTCGAAAGCGATTCTCAATTTTCCATTAGAA

GCTGGGAAGTGTGATGTACGCGCCAACGAAGAGGGTGAAAGGAAAAGACTTAGAGAGTGT

GACGCAGAAGAGGTGGAGGATGTTAAGAGGGTTATGCGAGTGGTTAAGAGAGAGGAACCC

GAGAGGGATGTTCCGCTGACGCCGTCATGTTGGACAGCTGTGTGGGATCATTGCAGCGAT

TCAAATGGTGTCTTTAACGTGCCTCCGTTGTCTCCGTTATCTCCTCACCCGCCGTTGGGA

TTTTCACAGCTAATGGTTATTTGA

>EVM0037606

ATGTACGGACAGAACATGCTTGATCAGTCCGATTATGCCTTTCTTGAGTCCGTTCGCTTG

CATTTGCTTGGCGAAACTATCTCTTCTGCAGCTGCCAGCAGCTGCTTTAATCCAGATACT

ACTAACAGTGCGATCTCAACAGCAACCACCCCTGCAACCTTTACTCCAGCCCCTGTTTAC

TGCAGGAGCACAAGCTTCAGCAGCTTGTACCCTTGCTTAACAGAAAACTGGGGAGACTTG

CCACTCAAAGTCGATGATTCTGAGGATATGTTCCTTTACGGTGTCCTCCGCGACGCCGTC

ACCGTTGGTTGGGTCCCATCACTCATGACCGATCAATTTATTGAGCACAGTCTTCCAGCG

GTGAAGCTAGAACCCTCTGAGAATTTGACGATATCCCCTTCGCGCGCGCAGGCTCCGATA

GCTGAGCCGGCGGTGGTTCCGTCCAAGGGGAAGCATTACCGGGGTGTGAGGCAGCGGCCG

TGGGGGAAATTCGCTGCGGAGATTAGGGACCCGGCGAAGAATGGGGCCCGGGTTTGGCTG

GGGACATTCGAGACGGCAGAGGATGCAGCGTTGGCTTACGACCGCGCTGCCTATAGAATG

CGTGGGTCGAGGGCGTTGTTGAATTTTCCGTTGAGGGTGAATTCCGGTGAACCGGATCCG

GTTAGAGTGACTTCGAAGAGGTCGTCGCCGGAGCCATCATCGTCTGTGGAGAGTGCGTCC

CCAAAGAGGAGGAAGAAGGTTGGTGGCGCGGCGGGTAATGCTACAGTGATTGCTCAAGCT

GGGATGCAAATGACGAATGGGGTTGGGTATCAAGTGGGCACACATGGCGAACAATTATTG

GTTGCTAGATAA

>EVM0029808

ATGTCTACTGACAGGCAAGAAACATACCCATCTTCTGATAGCAGCAACTCTCCAGTGACA

GTCCACTCTGATGAAGAAGTGCTGCTAGCAGAAAGCTGTCCGAAAAAGCGCGCTGGCCGC

AGGATATTCAAGGAGACTCGGCACCCGGTTTTTAGAGGTACCAGGAAGAGGAACGGAGAC

AAATGGGTGTGTGAAATGCGGGAACCAAACAAGAAGTCACGAATATGGTTAGGAACATAT

CCTACACCAGAAATGGCAGCACGGGCCCATGATGTCGCTGCTTTGGCACTTAGAGGCAAA

TCTGCCTGCCTTAACTTCGCTGATTCTGCTTGGAGGTTGCCTGTGCCGGTTTCAAACGAC

GCGAAGGATATTAGAAGGGCAGCAAATGAGGCAGCAGAATTATTCAGGCCTCAGGAGTTC

GGTGGCTGCCAGGCAAGCCAACAAAACAGCAATGCAGTACCAGAGGATTGCTCAAGTGAA

GCTTGCAATGATGGTCGCGAAACTTTTCAACAAAACAGCAATGCAGTACCAGAGGATTGC

TCAAGAGAAGCTTGCAATGATGGTCGAGAAACTTTTCAACAAAACAGCAATGCAGTACCA

GAGGATTGCTCAAGAGAAGCTTGCAATAATGGTCGCGAAACTTTTCAACAATCAAGAGAA

GCTTGCAATAATGGTCGCGAAACTTTTCAACAAAACAGCAATGCAGTACCAGAGGATTGC

TCAAGAGAAGCTTGCAATGATGGCCGAGAAACTTTTCAACAAAACAGCAATGCAGTACCA

GAGGATTGCTCAAGAGAAGCTTGCAATGATGGTCGCGAAACTTTTCAAGAAAACGATGTC

TTCTTGGAGGAGGCAATGCTAGACATGCCAGGGTTGCTCGTGGACATGGCAGCAGGTCTT

CTACTTCCTCCTCCACATTATGTAAGCAATGATAGTATGGACCGGGATCATACGGAGAAT

GATTCTTATATGTCATTATGGAGTTACTAG

>EVM0018598

ATGGCCACAACTTCATCTAGCTCCAAAAGGCACCCAGTGTATCGTGGAATCCGGAGCCGC

AGTGGAAAATGGGTGTCCGAAATCCGGGAGCCCAGAAAAACCACTCGTATTTGGCTCGGC

ACGTTCCCAAATCCGGAGATGGCAGCAGCAGCCTATGATGTTGCAGTCCTAGCCCTAAAA

GGTGCCGATGCAGTTCTTAACTTCCCGAGTTCTGTTGGGACTTATCCTGTTCCTGCATCA

ACATCACCCACTGATATTCGTAATGCAGCTAATGCTGCTGCTGCATTAAAAAAAGCTGAA

TTGAGCAACAATGCAGCATTAGTTGAACAGTCTAGGAATGACTATGCCTATATTGGTACC

TTTTTGGCATCAAGTGGAGAGGAATTTGTCGATGAGGAGGCACTGTTTGATATGCCAAAT

TTGCTGGTGGACATGGCAGGAGGAATGCTTCTTTCTCCACCAAGAATAACCTCATCATCG

CCATCTGATGACTCACAGGGAACTTCTGATGGAGAAAGTCTGTGGAGCTATTCTTAA

>EVM0054100

ATCCGTTACAGAGGCGTCAGGAAGAGACCATGGGGACGTTACGCGGCTGAGATCAGAGAC

CCTGGCAAGAAGACGAGGGTGTGGCTTGGCACTTTCGATACCGCTGAAGAGGCCGCTCGT

GCATATGATGCGGCAGCTCGCGAGTTTCGCGGAGCCAAAGCCAAAACTAATTTCCCGCCA

ATCGGGGAACTCATTCCCGCCTCAACGCGCAGTCCCAGCCAAAGCAGCACTGTTGAGTCC

TCCTCCCCCCCGCACCCACGCGCCGCCTCTCCTCCACAACTCGATCTCACTCTCAACACC

GCACACCATAAGATCACCCGCCATTTGTTTCCCGACGGGGCTGGTTTCCCTGGAGGCGCG

TGGCTCCCACACCCTGCTGCCCAGCGCCCAGTTTTCTTTGTCGACGCGTTTGCGCAGGCG

AGGAATAACAGCAAAAATAGGATAGTGAATAATATCAGCATGTGCATGTTTGACCGGACA

GCGATGGTGAATGGAGGTGGGGCCCAGAGCGATTCGGATTCATCATCAGTCCTTGATTAT

GACCATCATCATCATGATTGTAAGGGGCTATCACTTGATCTTGATCTGAACTTCCCCCCG

CCACCGGAAGTCGCATGA

>EVM0052580

ATGGAATTTGAGAATTATTCATCGTCATCATCCTCACAATCTTCAATTCGTCAAACTTTA

TCATGCAAGCCATGCAACAACCCTGATACTTGTAATCTTTTGCGAGGGCAAGAGAGGCAA

AATGCAGTTTTGATCAAGAAGAACAGAGCGGGGAGGACGAAGTTCAAGGAGACGCGGCAT

CCAGTATACAGGGGGATACGGAGAAGAAATGGGAACAAATGGGTATGTGAAATGCGTGAA

CCAAACAAGAAATCAAGAATTTGGGTGGGGACCTTCAAGAGCCCAGAAATGGCAGCTAGG

GCACATGATGTTGCTGCTTTAGCACTTAAAGGAGAGTTCGCTACTTTGAACTTTCTTGAT

TCACCTTTGATACTTCCTCGAGCAAAGTCGTCTTCTGCTAGAGATATACAAAGGGCTGCC

CTTGCTGCTACTGAGGTCTTTGGACAAAGTGCCCCTTCATGTTCTTCTTCCTCGTCACCT

GATCACAAGAAACTTTCATTCGTGACAACGAAAGTTTTGAGTTTATCTTCAAGTGATCTT

CCCCATGAGAAGGGCAAAGAAATTGCTAATGAAAAGCCTTTTTCAAACATGGCCATGCCA

TCATGCTCTGATAATTGCTCGGAGAAGGTCCCTAATTATTCCAGTGAAAGGTTCTTTGAT

GAGGAGGCATTGTTTAACATGCCAGGATTACTTGATAGCATGGCAGAGGGTTTGATCCTT

ACCCCGCCAGCTATGGCAAGTGGAGTCCTTAATTGGGATGACATGGCTTGTTCTACGGAC

TTGACATTATGGGAGGAAGACTGCTTAGATTCTCGGTGA

>EVM0007543

ATGGAAAACTTCCCTCCATTTCTTTACAGAAACCCTAAAAGAAGCTCTAGGCAATCAAGT

AGATACCTTGGAGTAAGGAGAAGACCATGGGGGAGATATGCTGCAGAGATTCGCAATCCA

TACACTAAAGAGAGGCATTGGCTAGGCACGTTTGACACTGCTGAGGAAGCTGCTGTAGCA

TATGATCTATCATCCATCTCTTTTAGTGGCATTGAAAGAGCTCGTACCAACTTTTACTAC

CCTCTCTTCCGGCATCCCTCGCCTCCACAAGAGGCGCCTCCGCCTCCACAACCACCGCCT

GAGATGGAGAAGGGTGATCAACTTGGTATGGAGGACATAGGCACCGCACAAGATGACGAG

TCTTTTGTTATTGCCTCTATCTTGCAAAGTTTTTGCCAGCCCACCGGTTATTCTTTCCAT

CCACAAATTTAA

>EVM0040155

ATGACTGTCTCGCCGGTTTCAAACTCCAACTCCGAGACAGGCTTCTCGAGCATTTACTCC

TCCTCCCCGCAAGCCCCTTATTCCCCATCCTCGTCAGGACTATCACCAAACCCGGTATCT

AAACCCGACCCGGAAGAAAACCCGAGAAAACCAAAGAGGCCAAGAGAGAGCGGAAGCGGA

AGCGGCAGCGGCAGCAAGCACCCGGTATTCAGGGGGGTCCGAATGCGGACATGGGGAAAA

TGGGTGTCCGAGATCCGGGAGCCCAGAAAGAAGAATCGGATCTGGCTAGGCACTTTTTCC

ACCCCGGAAATGGCAGCACGTGCCCATGACGTGGCAGCCTTAAGCATTAAAGGCAAGTCA

GCCATCCTCAACTTCCCCAAACTTGCCGGGTCTCTACCACGACCCGCTTCTAACTCTCCA

CGTGATGTCCAAGATGCCGCCGCAAAAGCCGCTTCCATGGACTTCAACATTGTCCCTTCG

GATCCAACCAAAGTCTGCGACGATGAAAATAACAGTAACAGCAACGACTCTACGGCGAGT

AATAATAATGGGTCATCGTCCTCGCTAGTGACACAGTCGTCCTCCTCCCCGTCCTCATCA

TCAACAGCGGAGGTGACATCATCACCAAGTGACGTGGCGACTCCTGAGGAACTGAGTGAG

ATAGTTGAGTTGCCGAGTTTGGAAACAAGTTTCGAGGAATTTCCCGAGTTTGTTTTGTCT

GGTGACTTGTGGCCGTACAATATTAATCAATCCTGGTATTGTGAAGACTACGGTGCGGAG

GGTACTGGGTATTTTAGCGATCAGTATTTGCCAATAATTCCAGAAAGTAATGTGATTACG

ACTACTGGTGCTTTTGAGACTTCATTGTGGGAACATTGA

>EVM0054412

ATGGATTTTACTCACTCAACAAAAACCAACTCTACACCCTCTCCTTCAAAGACCAAAAGA

AAGCAGCAGCAGCAGCAGCAGAACCACCAGAATCAACAAGAACAACATGAAGTACGGTTT

TTGGGGGTAAGGAGAAGGCCATGGGGCAGATATGCTGCAGAAATAAGAGACCCTTCAACA

AAAGAGAGGCACTGGCTTGGTACTTTTGACACCGCTGAGGAGGCTGCCTTGGCTTATGAC

CGTGCAGCCAGGTCCATGCGAGGTTCCAAAGCCCGCACCAACTTTGTCTACTCAGACATG

CCTCCTGCCTCCTCCGTCACTTCCATTATCTCACCAGACGAATCCCAACATGAAATTTCA

GCCCTCTTTGCTCCTCCTCCACAAAACCATGCTCATCAAAACGACACCAACTGCCAGCAG

CTCTACTTCTCTCAGGATCAGTACCCCTTCAATGCTTATGGTAACAGTAACACTAACTTG

TTAACAGGTGGAGAAGGTTGGGTTCAAGGATTTGAAGCTGGTGCTGTGGATGGCCCCGGA

GGGTCCTATGAGCCCAATACTGCTGGCAGCTTTGATGTCGCTACTGAGCCCAGCTACTTC

TCTAGTAAGGACAATATCGAGCTCCCACCCTTGCCTCCAGATGTCAACTCCAGTTGCTAT

GGGCCTGATATGGATCATGGATTCTGGAACGACGCAGGATTTTTTGGGTTTCAAGAGGAA

CAGAAAAATAACGTTAATGGGCTTGGAAATAGTGGATCAAGCTTGGGTTTTGATTCAAGT

GGCTTTGGGCAGAATGGCTCGTTATTTGAGATTATGCCATCGGTATCGGATTCGGTCAAA

GACGGGCTTGATCTGGGATCATCATCTACCCTTTACTTTTAA

>EVM0026277

ATGTCTGCTATGGTTTCAGCTCTCACTCAAGTCATGGGTACTTCCAACAATAACCCAACT

TCGGTGCAATCCACCCAGTGTGATCTGGAGCAATCCGTCGTCAAAGACGAGCCTGACCAG

TCTCGACCAGTGCAAGATCAAGAAAATACAAGGAGAAGGCATTATAGAGGAGTGAGACAG

AGACCTTGGGGTAAATGGGCAGCCGAGATACGTGATCCTAAAAAGGCAGCCCGAGTCTGG

CTTGGTACTTTTGACACTGCTGAGGATGCAGCCGTTGCATATGATAAAGCAGCACTTAAG

TTCAAAGGCACCAAGGCTAAGCTTAATTTTCCCGAAAGAGTTCAAGGAAGAACCGAGTTC

GGCTACTTCATGGGTTCTGGGGCTTCGAGCAATGTCGTGACTGAACAAAGTCCCAGGCCA

GTTGCTCCCCCTCCTCCCCCTCCCCCTTCATCATTTACACCAGACACTTACCCAGACTTG

GTTCAGTATGCACAAATCCTTTCTAGCAATGATGCCAATTTCCCGTACTACACTTCAAAC

CTCTTTAATCAACAACCTTTTGCTCCCCATTTTTCATCAAGCTTTTTATCGCAGCAACAG

CTGCAGCAACAACAAGATCTGATGAGATTCTCGTCAGGGTTCGATAGCTCCTTTAGTTCT

GATCAACAGGAGCATGGGAAGGACTCCAGTAATCCTAGCGAGTAA

>EVM0027008

ATGGCAAAACCATCGTCGTCATCAGGGCAACAGTCTGATAGAGATCACGAGCCCAAGTAC

AAAGGTGTTAGAAAGAGAAAGTGGGGGAAGTGGGTGTCCGAGATTAGACTTCCCAATAGT

CGGGAGAGAATATGGTTAGGATCTTATGACACTCCCGAAAAGGCGGCGCGTGCATTCGAC

GCGGCCCTGTACTGTCTGCGCGGTAGTGGCGCCAAGCTTAATTTCCCCGACACTCCGCCG

GACATTGCTGGCGGGCGGTCACTTAGCTCGCAAGAAATACGAGAAGTTGCTGCCAGGTTT

GCTAATGATGAGCCAACATCGTCGGCTGCTATGGGAGATGAATCATCAGCACATGTGGAG

AATTACACATCATCGTCGTCTGATGGTGGGGCTGGAGATATTATAGATTGGTCGTTTTTG

GATTTGCTGGATTCTAGCGAGGGTGCTTCAGATTTTGGACTCTATCATGGGCTGGACCGC

ATAGGTGGTGATTATTACCCCCGCCGCCGCCGCCGCCGCCTGAATATAGCATCGATGGGG

ATGATCACAATGGAGATGAAGCTTACTCTCATCAGTCATTTCTTTGGAACTTCTAAAGGG

ATTGGTTCAAATCTTGGTAAATTCTATCCTAGATACTTAGTAAATCTCAGGAACTCCGGC

GAATTTTAG

>EVM0034096

ATGAGTGATCATCATCAGCAAAATTCAGAGACGGAAAGTAGCTCAACAAACTCATTGTCA

CCAGCCTCACCCTCTTCACCTTTATCCAGCTCTACCCAATCGAAGAAACCTCTACAAGAC

CAAGATTCCTCGCCGTCTAAGAAAGTAAAAAGGACAAGAGACACGAACAAGCATCCAGTT

TATCGTGGAGTTCGCATGAGGAACTGGGGCAAATGGGTGTCAGAAATTCGTGAGCCGCGC

AAAAAATCTCGCATTTGGTTAGGTACTTTTCCTACACCAGAAATGGCAGCGCGTGCGCAT

GATGTAGCTGCTTTAAGCATCAAGGGAAACTCAGCAATTCTTAATTTCCCCGAACTCAGC

AATTCCTTACCCCGGCCAGCCTCACTTGCTCCTCGGGATATTCAAGCTGCCGCTGCTAAA

GCTGCTCAGATGGACAAGTTTGACCAAAAATCTGAAACAACCACAACATCTCCATCGTCC

CCTTCGACTTCTTCTTTGACATCTCTTGTTTCTTTGATGGACTTGTCGTCTCAGGAGGAA

GAGCTGTGTGAGATCGTTGAGTTGCCCAGTCTTGAAACGAGTTACGACGAGCTGAAAAAT

GATTTTGTTTACGTTGACTCTATTGATGGGTGGATGTATCCTCCGCCATGGATGCAAAGC

ATGGAAAATCACTGTTATGGCGGCGGCGGCGGCGGGTGTGCCTGTGATGATTTTACATTC

CCGAACGAGAGTACTGTTTTATGGAATTACTAG

>EVM0002257

ATGGTTCTCAGTATTTGGAAAACCAGCGTTGAAATGGTGAGAGAAAGAAGAGAGAGAGGA

GATGATAACGCGCGTTACAAGGGAGTGAGGATGAGAAAATGGGGGAAATGGGTAGCGGAA

ATAAGGCAACCTAACAGCAGAAATAGAATTTGGTTAGGTTCGTACAATTCTGCAGATGAA

GCGGCAAGAGCATACGATGCTGCTGTTTTATGTTTACGAGGGCCTTCGGCCACGTTTAAT

TTTCCATCGAACATGCCGGAAATTCCTGCTAGGACGGAGGTATTGTCTCCCACGCAGATA

AGGGAGGTTGCTTCAAGGCATGCAAGAAGGAGGGGTGCTGTGGAGCTTGCAGAGAGGATT

GTGAGACCTGGGTTGTGTGAAGTTCCGTCTGGGATGACTGAAGAGGTGTACTTGGGCGGT

GGAGAGAACATGGCGGAATTCAAGGATGGAATTTTTAGTGGGGCATACTATCAAACACCT

GGTGTGTGGGCAGATTAA

>EVM0043935

ATGGAATTTCATCAGCTCCAAAAGCAGCCTAGAGGAGTCTCGCCCAGCAAACCAAGCAAA

TTCAAGGACAGATGCTCGAGCAACAACTCCAAGAACAAGTTTGTTGGAGTTAGACAAAGG

CCTTCTGGGAAATGGATTGCAGAGATCAAAGACACCACTCAGAAGATCAGAATGTGGCTC

GGGACCTTTGAGACAGCTGAAGAAGCTGCTCGAGCTTATGATGAAGCTGCCTGTCTTCTT

CGTGGTTCGAACACCCGAACCAACTTCAACACTCTTGTTTCTTCCGATTCTCCTATTTCT

GCGAAAATTAGAAACCTCCTCAATCACAAAAGGAGTTTGAAACAAAACCCTTCCACAACT

TCCTCCGCAAAATCTACTACTAAAGCCAGCACTGTAGGTAGCACCACGAGCAGTTTCAAT

AGTAGCATGGGTAGTTTCCTACTTAATAATACCAATGTTGATAGCTTTCCATCATCTATT

GCTAGCCATGCTGGTTATTTCAATGGCATCATACAAGAGAACCAAATGTTTGATGATGCA

TACAGACCAGACTTGAGTGGCTGCTGCACTGGAGGGCTCGAGCCGGTTACGTCCCAGTTC

CATCCCTCGTGCTCGTCCCCATCTGGGTTGGATCCTCCTATTCCATATATTCAACAAGGA

ATGGAGCTGCCAAAGAATGTTGGTCTGCTGTCTGATGCATCATCCAGCGTAGAATTAGCC

GAATTCGAACGCATGAACGCCGAAAGGCAGGTACCAGCATCATTTTATGTCATGAATGGA

GCGAAAGAGTACTTTGAGAATGCTAACGAGTCAAGTGAGGCTCTCTGGGATCTCCCTGCA

CTCTGTCAATTGTTCTGTCCTAGTTAA

>EVM0016170

ATGGAAATTCATTTCCAGCAACAAAAGAAGTACAGAAAAGAAAGCATGCCAGTTAGCAGA

ACGAGCAAATTAAAGGGGACAAGAAGGCCTAAAAACAGCAACAAATTTGTTGGTGTTAGG

CAAAGACCTTCTGGTAGATGGGTAGCTGAGATCAAATACACTACACAAAAGATAAGGATG

TGGCTCGGAACATTTGAGACTGCTGAAGAAGCTGCTCGAGCTTACGATGAAGCTGCTTGC

CTGCTTCGTGGATCAAATACTCGAACCAACTTCATCACTCATGTATCCTTGGATTCTCCT

CTTGCTTCCCGGATTCGAAACCTCCTCAACCAAAAAAGGGGAGACAAACAGCAGCAGAGT

GAGGAAGAAACTTCTGATGTCTCTACCCCACCAACTACCACCACCATTACCACAAGCACT

GGTAGTTGGATTAGTAGTTGTGGCAGGAGTACTGATAATTACTCCAATTCCAGATCGGTT

CATGAAACTGGAAACTTGTTTGATGATGCATATAAACCAGATTTAAGCAGTACTTCAATT

GAGGAATTCAAGTCGGGTTCTTCTCAGTCTGATATTTCATGGGGTTTTGGACCTGTGTTC

GATCGGTTTGCATTTTCCCAGGAAGTGTTGGATTTTCCAAACAGTGTGGTATCGCCCGAG

ACAAGTGAGTTGGAATTCTCAGAATTTGATACGATGAAGGTGGAGAGACAGATATCAGCA

TCTCTATATGCGATGAATGGGGTGCAAGAGTACATGGAAGCTATCTATGATCCTATTGAA

GCTTTTTGGGATCTTCCACCACTATAA

>EVM0027493

ATGGCAGACCCTCAGACGCTGAAAAACTCACCTACTGTCACCAGACTCACCACTAGCAAG

GTGCAAGCAACCAAAGGTCCTGCGGCTAGAAGATTTGTTGGAGTCAGGCAAAGACCATCA

GGAAAATGGGTAGCTGAGATAAAGGATTCATCTCAGCGTGTGAGGTTATGGTTAGGAACA

TACGATACACCTGAAGAGGCAGCTAGAGCCTATGATGAAGCTGCCCGTGCCCTACGTGGA

GAAAATGCAAGAACCAACTTTGCATCGGTTATTCCAAACTTGAACCAATCTGGATCAGCC

CCTAGTTCTGATGGTGGGCTCGACTTGTCAGGATCGGATGGGAGGCACGTTCTTAGCTTC

TCTTCATTAAAGGCTAAATTGAGCAAGAATCTGCAAAGTATCATGGCTAGGACTACAGAG

AATAAGTCAACCAAAAATAGAGTGAGTGACCACTTTGCTTTTGCTAATATATTCCACTTT

AGGAGCCACCAATACCAAAACCCTGTGGATATGAAGAGCATCGAGAAAGTTGTGCAGCCG

AGCATCATTGTGCCCCATGTATCAGATCATAAACCTTCTTGTTCTTGGGAACCTTCTTGT

GTCTCAGATTGTAGCAATGAATGGATTGGATTCAGGCAGCATGGGTTTGATTCAGATGGA

TCTGATATTGGAGAAGTCAGCACGGTTAATTCTGGTCAAATGACAGGTTGGATTGATAAC

CCAGATATCAAAACTTGTTGTGGAGATCCTTGTCCAAGGAGTAAGAGGTTCAAGGTTTCG

TCTTCTGTTGTGGTTCCTCCTACCTTTAGTGGGCCTCCATCCTTCTGTGCTTCTTCTCCC

TCCTTTTGTGGCTCTCCATTTCATGTTGAGAACTAA

>EVM0051566

ATGGATGCCTCCTCGTGGATCAACTTGGTTCAGAGACGAGAGCAAGAACACAACGCCATA

GTATCAGCTCTCAAGCATGTAATCTCAGGGGGCACTGATCATGGCAGCACTACCGGCCCA

AATGAATACCAAGACCAGCAGAATATCCTAGCTGCTGCTGCTGCTGCTGCTGCTGCTGAT

GGTAATGCGGGTGAGAGGAGCAACGTCATTTACGTTTCAGATTCTGAGACGTGTCTGTTT

TGCAAATTGAGTACCTCTCAATGCCTAGGTTGCGCCTATTTCTCCGAGGGTACTAATGAT

CAAGGAGGAGGAGGAGGAGGAGGAAATGAGGACACTACAAAGCAGAAAGGTAAAGGGAAG

AGGAGGAAGAACAAGTATCGGGGAGTCCGCCAACGACCGTGGGGGAAATGGGCGGCGGAG

ATAAGGAACCCAACGCAAAGGAATCGTGTGTGGCTGGGCACGTTTGAGAATGCAGAAGAT

GCTGCGAGAGCTTATGACATGAAAAATATAGAATTTCGTGGAATTAGAGCCATAACTAAT

TTCCCATCATCTGACTACCAAGTTCAAGAGACCGAGCAAGATAACCCAAACCAGACTGGC

GAAGCAAGCAATGCCGCAGGGGAAGCTAGTGGTGGCCGGTAG

>EVM0057361

ATGGATGCCTTCTCGTCGAACGACTTGGTTCAGAGACGAGAACACGAACACAACGCCATA

GTATCAGCTCTCAAGCATGTAATCTCAGGTGGCACTGATCATGGCAGCAGTACCGGCCCA

ATTGAGTACCAGGACCAGCAGAATATCCTAGCTGCTGCTGCTGATGGTAATGCGGGTGAG

AGGAGCAACGTCATTTACGTTTCAGATTCTGAGACGTGTCTGGTTTGCAAATTGAGTACC

ACTCAATGCCTTGGTTGCGTCTATTTCTCCGAGGGTACTAGTGATCCAGGAGGAGGAGGA

GGAGGAGGAGGAGGAGGAGGGGAGGAGGACACCACAAAGCAGAAAGGTAAAGGGAAGAGG

AGGAAGAACAAGTATCGGGGAGTCAGGCAACGACCGTGGGGGAAATGGGCGGCGGAGATA

AGCGACCCAGCGCAAGGGACTCGTGTGTGGCTGGGCACGTTTGAGAATGCAGAAGATGCT

GCGAGAGCTTATGATATGAAAAATATAGAATTTCGTGGAATTAGAGCCATAACCAATTTC

CCACTATCTGACTACCAAGTTCAAGAGACGGAGCAACATAACCCAAACCAGACTGGCGAA

GCAAGCAATGCCGCAGGGGAAGCTAGTGGTGGCCGGTAG

>EVM0018988

ATGAACCACCAAAAACTGCTCCCCGTTCCGTTCTTCCCTCAACCCGAAACATTTCAAATG

TTTCGGTTCCTGACTGAAAATTCAACGTTTTGTGAAACTTTTTTAGAACCTCAAATTTCA

TTTTCTTCACCTTTCACCTTTAACCACTGCAACCCCACAAACTTCTCTCCTGTTTCACCC

GATATCCCGGCTGCTGTTTCACTCTCCGATGGATTCAAGAAATCACCAGAAACACCACTG

GTCCTTGATGGAATCGGTGCTGTTGTTGGTCAACAAGTCCTTTTTGGCAAGAACGATACC

AAGAATTCAGAAGTATCGAAGCTTTCTTCAATTTCTCAACCTACCGGGTCAAAAACAAGT

AAACAAGAGAGTGGCGCCATGAGAAACAGTGTTAGTGACGGGGTTTCAAAGCAAAAGGCC

TACAGAGGGGTGAGAAAGAGGCCATGGGGGAGGTGGTCAGCTGAGATAAGAGACCGGATA

GGAAGGTGCCGCCACTGGCTTGGAACATTCGACACTGCAGAGGAGGCAGCGCGTGCTTAT

GACTCAGCAGCTAGAAGACTTAGAGGTGCAAAAGCAAGAACTAACTTTGAAATCCCTCCT

GTTTTGCCCGCAATATCTCCTTCCTCTTCTCCACCAAGTTCATGCTCGGGCTCAATCTCT

AATAATAACGCAGAACCGAGGAAGAGGACTAAGGTCTGTAACCACAGCAGCAAAATGATT

GGCAGGAAGTGTGCGGTTGTCACTTCTGCGGCACAATTGTTTAGTGATTTTGAAGGGAAA

GGAACTAGTTCCGGAGGCAACGTGGAGCTCGAGTTGAAATTGGGAATGGGGTCTGATGGC

TATCAAGGTGGCAACAAGATCGCCGCCAGCGCTCCTCCATATATGGTGGTTTAG

>EVM0006672

ATGGCCAATCGTGAGCCTCGAGCTGACCAATTACCATCCCTTCCCTCTCCGATCCTGATC

CCGTCTCCGGCCAGCTCTTCGGGCCAGCCCTTTGATTCAAGGCACGAGCATTCGACGAGG

CTTATTCGATCACCCATTGGGTTCTCGAGTCAGCCCGTGAGTGGGAGGCAAGGTGACCAA

TCACCGAGGCGGCCAATTCGATCACCGGGTGGCTCATCGGCTCAACCCACAAGCGGAAGG

CACCCGAGTTTTAGAGGTATTAGGTTACGGAGTGGGAAATGGGTATCCGAGATCCGGGAA

CCACGTAAAACTACACGTGTATGGCTCGGTACGTATCCAACTCCTGAAATGGCAGCCACC

GCCTATGATGTGGCGGCTCTAGCACTTAAGGGCCCCGACACGCCACTGAATTTCCCGGAA

TCAATCCTTTCCTACCCAATACCAGCCTCCGCGTCCCCTAGTGATATAAGAGCTGCCGCT

GCTGGTGCAGCATCAGGCAGGTCATCTCAGCGTGGAAGAGGGCCACGTCCTGAAGTGGGC

CAGCCAGAAACAGAAGGCACATCATCTAGCATCACTGCTGATGCTCAATCAGGACAGGAA

ATTTTCGACGAGGAAGAACTCTTAAACATGCCCAATTTGCTAGTTGACATGGCCGGAGGA

ATGCTAGTGAGCCCGCCAAGAATAAACACGCCATCTTCCGATGATCATTCACCAGGGAGC

TCCGACGCTGAAAGCCTATGGTCCTACCCCTAA

>EVM0009316

ATGGATATTTTCTTTAGTTATTCTGATCAAAACCCAATTGGGTCCATCAATTTGTTGCCA

GTTTTTGACGAGCAAGAATGTTCTTATTCTTCTGCTCTTTCAGATAGTAGCATCACTAGT

AGTGTTACTAAAGGAGTTCAACAAGCCGCAATTTTTTCTGATGAGGAAGTAATGTTAGCT

TCCAGGAATCCGAAGAAGAGGGCAGGAAGAAAGAAGTTTAGGGAAACAAGGCATCCTGTG

TATAGAGGGGTGAGGAGGAGGAATTCGGGCAAGTGGGTGTGTGAAGTTAGAGAGCCTAAT

AAGAAATCAAGGATTTGGTTAGGGACTTTTCCTACCGCTGATATGGCTGCTAGGGCACAT

GATGTTGCTGCTTTGGCGTTGAGGGGGAGGTCTGCTTGCTTGAATTTTGCTGATTCTGCG

TGGAGGCTGCCGGTTCCGGCTTCCCGCGATGCCAAGGATATTCAGAAGGCTGCAGCGGAA

GCAGCAGAGGCTTTCCGGCCGGAGGGGTGTCTGGGAGTCGACTTAACGAGGACGGGAGAA

GAGGTTGAGAAGGTTGCGGAGACGGCGGCAGGGGAGGTGTTTTATATGGATGACGAGGCA

GATTTTGGTATGCCAGGTTTGTTGGCTAATATTGCTGAAGGGATGTTACTGCCACCACCT

AATTGTTGTGGTTGCTCCGGTGGGGATAGTGGGGATACAATGGAAAATAATGACACCGAC

ATGTCATTATGGAGTTTTTCTGTTTAA

>EVM0009672

ATGGGGAAATCATCGAAGCAAAGCCTGAAGAACTCTGAAAACACCGGCATTAATCCGACA

ACCAAGGTGAAGCGGACAAGAAAAAGTGTTCCTCGAGACTCCCCTCCTCGACGAAGCTCC

ATCTACAGAGGCGTTACAAGGCATCGATGGACCGGACGTTATGAGGCTCATTTGTGGGAT

AAGAACTGCTGGAATGAATCACAGAACAAGAAAGGAAGACAAGTGTATTTAGGGGCCTAT

GATGACGAAGAAGCGGCGGGACATGCGTATGATTTGGCAGCACTAAAGTACTGGGGACAG

GATACCCTCCTCAATTTCCCTGTGTCAACTTACGAAGAAGAACTCAAAGAGATGGAGGGC

CAGTCAAAAGAAGAATATATTGGATCACTGAGGAGAAAAAGTAGTGGGTTTTCACGCGGC

GTGTCCAAGTATAGAGGTGTAGCCAGACACCATCATAATGGAAGATGGGAGGCTCGAATT

GGCAGAGTTTTCGGCAACAAGTACCTCTACCTTGGAACATATGCTACTCAAGAAGAAGCA

GCGACGGCGTATGACATGGCAGCCATAGAGTACCGTGGACTTAATGCCGTGACAAACTTT

GACCTAAGCCGTTACGTCAAATGGCTGCGTCCCGATAACCAGGATAATATTACTAATCCT

CAACAAAACCCTAATGGCGATGCTAATCCTATACAAAACCTTGATCAAAAGCTCGGGTTG

GATTTCATATCGCATCAACAAAGTTCGAATGCTGATGACACAGAAGCGGCTCCAGCTCGG

CCTTGTGGTGCTGGTGGCCCGGCGTCATCGGCATTAGGGCTTCTGTTGCAGTCCTCCAAG

TTCAAGGAAATGCTGGAGAGAACATCGGCTTCTGATTGCCCTTCGACACCACCGGAGTCC

GACCGGGATCCGCCTCGAAGGAGCTTCCCGGATGACATCCAAACTTTCTTCGACTGCCAA

GACTCCGGTAGCTACACTGATGGCGATGACATTATTTTTGGAGACCTTCATTCATTTGCT

TCACCGATTTTTCATTGCGAATTGGATGGGTAG

>EVM0011072

ATGACTAGACCACAACAGCGATATCGTGGCGTCCGTCAAAGGCATTGGGGTTCTTGGGTT

TCAGAAATCCGCCACCCTCTCCTGAAGACTAGAATATGGTTAGGTACATTCGAAACGGCC

GAGGACGCGGCGCGGGCATACGACGAAGCAGCAAGGTTAATGTGCGGGCCCAAAGCACGT

ACAAATTTCCCTTACAATCCCAACGAACCGCAATCATCATCATCAAAGCTTCTCTCAGCT

ACCTTGGTAGCCAAGCTACATAAATGTCAAATGACTTCCCTACAAGCAGCCAAGAAAAAT

GTGACAAAACAATCGCATGATGTACAATGTACCACTTTTGTCTCTAGCCATGGCATTGCC

GGAAATACTGTCGAAAGCGGCTCGAAATGGCAGGCGGGGGACTGGGTTGGTGGGGAAAGT

CAAGTGGGGGACGGTGATCGTCAAGAACATTTTAAGTCACTTGAAGATCACCATATTGAG

CAGATGATAGAGGAGTTGCTAGATAGAGAGTCCATGGAGCTCTGCTATGTTGGTTCAACA

TGA

>EVM0017689

ATGTCAAAATCATTCATGAGTGGTTATGGAGAGAAAAAACAGTTCAAGAAGCCTGCGCAG

GCTAGTTCTCGGAGAGGGTGCATGAGAGGGAAAAGGGGGCCAGAGAATGCTCTCTGCTCT

TACAAGGGTGTCCGACAGAGAACTTGGGGGAAATGGATGGCTGAGATTCGAGAGCCCAAC

CGTGGTGCTCGTCTCTGGCTTGGCACCTTTGACACCTCCCATGAAGCTGCCACGGCTTAC

GATGCTGCTGCTAGTAAACTTTATGGATCTGAGGCAAAGCTCAATTTACCAGATCTCCAA

GGTAATAATTGCCAGTTTCCTGCCTCTCCATCCAATTCCCAAGTGACCCAAACAACCCAG

CCTTCTCAAATCATCCATAACTATAGTTCTACTACATCTACATGCTCGTCCAATAACCCA

AGTATCAAGTCTAATGAAATGGCACCAGTGCTATACAACCATGATCCAATCATGTATTTC

TGTAACGAAGGCGTTGATTCGAATGGAATGGAGGCAGTGAATTTTGGGCGAAGTGAAGTG

GAAATTAAAGAATATTGGTCGAATTTCAACGTGAACATTCCTTTCGATGATTCGATTTGG

GATGAGGCAGCAATGTCGATTAATTTTCTAGTGCTGGAAGATCCTGGGATTTTTGCAGGC

AATCTCATGGAAGGAACAGGTAGGGACACATTGCAGACTCCGTGGTGCATGTAA

>EVM0023967

ATGAAGAGGTCCTCGTCTTGCTCCTCCTCCTCCTCATCTTCTTGTGTTGCCTCTCAAAGC

ATTGAGAAACCAAAGGCCAAACGCGTTAGAAAGAATCAAAAGAGCAATCAAGGAAAATCC

CAGAATGCTGCTGCTAATCATAACTCTGGCAAAAGAAGTTCCATTTATAGAGGAGTCACC

AGACATAGATGGACAGGAAGGTTTGAAGCGCATCTTTGGGATAAGAGTTCATGGAACAAC

ATTCAAAACAAGAAGGGAAAGCAAGTTTATTTGGGTGCCTATGATAATGAGGAGGCAGCT

GCACGCACCTATGATCTTGCTGCCCTAAAGTACTGGGGAACAGAGACAACCTTGAATTTC

CCGATAGAAACATACACAAAAGAGAGAGAAGAGATGCAAAGGGCGAGTAAAGAAGAGTAC

TTGGCATCCTTGAGACGTCAAAGCAGTGGATTCTCGAGAGGAGTCTCCAAATACCGTGGG

GTGGCTAGGCATCATCATAATGGCCGATGGGAAGCTAGAATTGGACGAGTTTATGGGAAC

AAGTATCTCTACCTTGGAACTTATAATACACAAGAAGAGGCAGCGGCGGCGTATGATATG

GCGGCGATAGAGTATAGAGGAGCAAATGCAGTGACCAATTTTGATGTTAGCAATTACATG

GAGCGATTGAGGAAGAAAGGCATCCCCATAGACCGAATCCTCCAAGAACAACAGCTTCGT

AACAACTTGATTGATTCCAGAGTAGAAGTAGAGGCGGAGGTTGAACAACCACCACCACCG

CAACAACAAGAGGAAAAAGAGCAAAAAATAGTCCCGTCGTCGCAAATCCAACGCTCACAA

ATAAATCCAAGCCTGGATGACAGGTCTCCTATGGTAATTATGGACACCATCGAAGAGCAC

GAGCTAGCATGGAGCTTTTGCATGGATTTAGGATTGAACCTCACAATGCCTGATCTTCCT

CTCGAAAATTCTTGCGAGTTGCCAGACTTGTTCGATCATACCGGATTTGAAGACAACATC

GACTTGATATTTGACGCTTGTTGCTATGGAAATGAGGCCAGTCAGGCTGGCTACATATTA

GAGGATAATAGCACAGGAGGAGTTGAAAAGGTTGGTGTTACAAGGAGTGTTGACGAGGAA

AGTGAAAGTGGGAAGGACAAGTTGTCGTCTGATTCGGCCTCAAATTCTCCAACTAGTTCA

ACAGCCACCTCTGTTTCTTGTAACTATTATGTTTGA

>EVM0046201

ATGGAAAGCCGAGTAGACAAGGAGATGGCGACCAGAAAGAGAGGAGGGATGGGAAGTGAG

AGGCAATACAAGGGAATAAGGATGAGGAAGTGGGGGAAATGGGTGGCTGAGATACGAGAG

CCTAACAAAAGGTCAAGGATTTGGCTTGGTTCGTACTCATCACCGGTAGCGGCAGCTCGC

GCTTATGACACTGCTGTTTTTTATTTGAGAGGGCCGTCGGCGAGGTTGAATTTTCCTGAA

TTTTTGGCTGGAGAGAATTTTGGCGGCGGTGGATCATTTGGGGACATGTCAGCTGCTTCT

ATAAGGAAAAGAGCAACCGAGGTTGGAGCTCGTGTCGACGCTATTGAGACAGCGTTAAAC

CGCCGCCGCCGCCATCATGATGACCGTCAAAGAAATAGTAGTAACAGTACCAGTAGCAAT

GATAATAATGAAACGGTCGTTGATAGTAGAGAGTTGAAGTCACGGACGGTTGACTTGAAT

AAGATGCCTGACCCGGAGGATCTAGACGGAGATGAGTGGGATAGGAGTTAG

>EVM0008767

ATGTGGAACTTAAATGACTCGCCTGATCAAACAAGGGACGATGAATCCGAGGGATGGTCA

AGTCAAAAAACATCAATAGGTGGCGAAGTTGACAAGGGAAAAAGGGTCGGATCCGTGTCG

AATTCCAGCTCTTCTGCTGTAGTAATTGAAGATGGATCAGAAGAAGAGGATGGTTGTGAG

AGAGGAAATATATTAATCAAGAAACATAGTATAAGCTTCAGCAGCAAGATTTTGGGGTTC

TCGGTTCATTATGATCAAAACCCCATGGACACGAGTGATCCACCCGTGACCCGCCAGTTA

TTTCCTTTAGAGGATCAAGAAATGGGGTCCGCATCGAGAGTTGGAGGTGCATTTCCTCGG

GCTCACTGGGTTGGTGTCAAGTTTTGCCAATCGGATTCATCACTTGTATCTCAAAAATCC

ATGGAAGTCTCACGGCCACTGAAGAAAAGTAGGCGAGGACCGCGGTCCAGGAGCTCTCAA

TACCGTGGTGTTACCTTCTACCGGAGAACTGGCCGATGGGAATCTCATATATGGGATTGT

GGGAAGCAAGTGTATCTAGGTGGATTTGACACTGCACATGCAGCTGCTCGTGCATATGAT

AGGGCAGCTATCAAGTTTCGGGGAGTGGAAGCAGATATTAATTTTAGAATAGAAGATTAC

GAGGAAGACTTAAAACAGATGAGCAACCTCACCAAGGAAGAATTTGTTCATGTACTTCGC

CGGCAAAGCACAGGATTTCCTAGAGGAAGCTCCAAGTATAGAGGCGTAACCTTGCACAAG

TGCGGAAGATGGGAAGCTCGAATGGGCCAGTTTTTAGGCAAAAAGTATGTTTATCTAGGC

TTGTTTGATACTGAGATCGAAGCTGCAAGGGCCTATGACAGAGCTGCAATCAAGTACAAT

GGCAAAGAGGCAGTGACCAACTTTGATCCCAGCATTTATGAAAACGAGCTTGAATCATCA

GGGAATGCTGCGGCTCACAACCTTGATTTGAGCTTGGGCAATCCAGCTTCGAAGCAAAGT

AGTAAAGAATTTGGTCAGGATAGGCACAATGCTGCCATGGAGCAACTTTCAGCAGCAATG

TCATTTGAACCTAATTGGCAAAATCGGGGGTTCAGGCCTAAGCTTAACCTCTATGGAGGT

GACAACGATGGACATGGAAGAGATGGGCATCGGGAGACTGAAACGACCCAGCTTCTTAGC

AAAATCCACTTTCAATCTCTAGCATCACTCAAGTCTAGAGAGATGCCGAGATATGAGCAG

TTCAGGAGACCGTATGGAGATGGTCAGATGCTTCATGTTCTTCCCCCACAGTTCAACTCA

CCAAGTTATCAGATCCAGTATCCAAGCAGTAGCAATGGAGGCCGAATCGGAAGTGATCTT

TCCCTCTCTCTCACAGAGCTTCATCGGCGTCGTTATTATCAGCAATGGCAAGCAGGACCT

CCCCAGTGTACAAATGCTGCAGCATCATCAGGATTCCAACTGCAGATTAGGACTCCACAA

AACTGGCTGCAGGAAAATTGGTTCGACTGTCACATGAGACCCACTTAA

>EVM0044116

ATGGAATTTGAGAATTATTCATCGTCATCATCCTCGCAATCTTCAATTCGTCAAACTTTA

TCATGCAAGCCATGCAACAACCCTGATATTTATAATCTTACGCTAGGGCAAGAGAGGCAA

AATGCAGTTTTGATCAAGAAGAACAGGGCGGGGAGGACGAAGTTCAAGGAGACGCGGCAT

CCAGTATACAGGGGGGTGCGGCGAAGAAATGGGAACAAATGGGTTTGTGAAGTGCGTGAA

CCAAACAAGAAATCAAGAATTTGGGTGGGGACCTTCAAGAGCCCAGAAATGGCAGCTAGG

GCATATGACGTTGCTGCTTTAGCACTTAAAGGAGAGTTTGCTACTTTGAACTTTCTTGAT

TCACCTTCGATACTTCCTCGAGCAAAGTCGTCTTCTGCTAGAGATATACAAAGGGCTGCC

CGTGCTGCTACTGAGGTCTCTGGACAAAGTGCTTCTTCGTGTTCTTCTTCCTCGTCGCCT

GATCATAAGAAACTTTCATGCGAGACAAGGTCAACGAAAGTTTTGAGTTCATCTTTAAGT

CATCTTCCACATGAGAAGGGCACAGAAATTGCTAATGAAGAGCTTTTTTCAAACATGGCC

ATGTCATGCTCTGATAATTGCTCGGAGAAGGTCCCTAATTATTCCAATGAAAGGTTCTTT

GATGAGGAGGCATTGTTTAACATGCCAGGATTACTTGATAGCATGGCTGAAGGTTTGATC

CTTACCCCGCTAGCTATGGCAAGTGGAGCACTCAATTGGGATGACATGGCTTGTTCTACG

GACTTGACATTATGGGAGGACGACTGCTTAGATTCTCGGTGA

>EVM0042515

ATGGCACCAGCTACTAACTGGCTTTCTTTCTCTCTCTCTCCAATGGAAATGTTGAGCTCC

TCTACTGCCTCTCCTCACTACTTGTTCGATAACTTCTATGCTGATGGCTGGGCAAACCCC

AAAGAATCACAAGTCACGACCACAACAATGGCAGAAAGCTCTAATATTCTGACAAGCTTT

ATAGGCGCAGATACTCAACACCAGCAAGTCCCAAAACTGGAGGATTTTTTGGAGATTCTT

CATCATCTATTAATGTTCGATACTCGGATAGCCAGACAGAGACCCAAGACTCCGGCTTAC

TTCAACGACCAACAAGATCTTAAAGCTATTGCTGGGTTCCAAGCCTTTTCGACTAACTCA

GGCTCAGAGGTTGATGACTCGGCTTCAATAGCTCCCTCAAGGCTGGGTGGTATTGAGTCG

ACTGGGAACGGGTTGGGGTTCTCTAATAAAGCGGCCTTGTCACTTGGGGTGAGTAATGAT

ACAATCAATAATCAGGGTGGTTCTACAGAAAAGCTTGCTATTGTTTCTGCTGATAACGAT

TGCTCTAATAAAAAGATTACTGATACCTTTGGCCAGCGAACTTCTATTTACAGAGGAGTT

ACCAGACATCGATGGACAGGCAGATATGAAGCGCATCTGTGGGATAACAGCTGTAGGAGA

GAAGGTCAGGCCAGAAAAGGGCGTCAAGGTGGGTATGACAAGGAAGAAAAGGCAGCTAGA

GCTTATGATTTGGCAGCTCTAAAATACTGGGGTCCAGCGGCTACTACCAACTTCCCTGTT

TCGAACTATTCTACAGAATTGGAGGGCATGAAGTATGTGTCCAAGCAAGAATTCATTGCC

TCACTGAGGAGGAAAAGTAGCGGTTTTTCACGGGGAGCCTCTATTTACAGGGGTGTCACA

AGGCATCATCAACAGGGTCGTTGGCAAGCGAGGATTGGCCGAGTAGCTGGAAACAAAGAT

CTCTACCTTGGAACATTTGCCACAGAAGAGGAAGCAGCTGAGGCTTATGATATAGCTGCA

ATAAAGTTTAGGGGCCTGAATGCAGTGACCAATTTTGAGATGAACCGATATGATGTTGAA

AATATCATGAAGAGTGCTCTTCCAGTTGGTGGAGCAGCTAAGCGCTTAAAGACATCGCTA

AACGCCGAGCAGAAACCAAATGTGAACGACAACCAGCAGCCGCCCCATTTGCGGTGCAGC

AGCTATAGCACCGCCAACAACATCAGCTTTGCTGCTACCCAGCCAGGCTCTACTGCAGTC

CCTTGTGGGGTTCCTTTTGATGCTTCCACCGCGCTTTATCATCACAATCTTGTCCACCAC

CTCCAAACCACTAACAACTTCGCAGCAACTGACTCCTCCGGCTCCTCCTCATCCATGGCA

ACTGCAATGACTACTTCGCCGCAAACAACAGAGTATTTTACATGGCCCCATCAAACTTAT

TGA

>EVM0015962

ATGCAAGACTCTCAACAAAGAAAAACCTCACGTCCTAGCAGTGGCAGAACTGCTTCTGTT

GCTGTGACTGCTGGGTCAATTTCAGGTCCCCAACGTGCTTTTCGTGGTGTTCGGCGTAGA

AGTAGTGGAAAATGGGTGTCTGAGATTAGAGAGCCTAAGAAGCCTAACAGAATATGGCTG

GGCACATTTCCTAGCCCTGAAATGGCTGCCGTTGCTTATGATGTGGCCGCGCTTGCGCTT

AAGGGCCAAAATGCAGAGCTTAACTTCCCAAACTCGGCTGCTTCTCTGCCTGTTCCTGCT

TCCACTTCGCCACGTGATATTCAGGCGGCTGCAGCCTCTGCAGCTGCTGCTATTGGCGCT

GCGCAGGATGCTTTAGGGATTCCAAATACGGGGGATGATAATAAAATGGAACAGGAATTT

AGGCCAACGGTCGATGATCATTTTGTTGATGAGGATTTGATATTTGATATGCCTAATGTT

CTTGTGAACATGGCTGAAGGGATGCTTCTTAGCCCTCCTCGCTTGGACATTGCTGGTGAT

CATGAGACCACAACCGCCTATGACAGCACTGAAGACCAGGACCTTTGGAAATTCCCTTGA

>EVM0042416

ATGGAAGGAAGAACCAGGGACGGCCACTTGGGGATTAGTCCGCCGAGATATCGAGGTGTA

CGACAGCGAAAATGGGGGAAATGGGTGTCCGAAATCCGGGAGCCTGGTAAGAAAACAAGA

ATTTGGCTCGGGAGTTACGAGATGCCTGAAATGGCCGCGGCGGCGTACGATGTGGCAGCA

TTACACCTTAGAGGACGTGGGGCTCAGCTAAATTTTCCTGAAATGGATGTGCAAATGGCA

GCACAAGAGGCGGCTTTGCTGTTTCGGAGACCGATAAAATGTTCGGAGGGAGCAAGTGGT

AGCTCTAGTGGTGGTGGTGGTGGTACTATAAATGAGGCGCCACTGGACTCACCAAAAATG

TGGATGGAGTTAGCTGGCGCTCGTCTGTTAGATGAGCCTATGGCTATGGGTGATGATATT

GATGTTGAGCTCAGGGATGAGTGGGGTGAAATCGAGCACGAACAAGACTCCATTTGGGAT

TACTAA

>EVM0016569

ATGGAAGCGAGAAACACTGGTAGCCATGGTGGGATGAGTTCTTGCTATAGAGGGGTACGA

AAGAGAAAATGGGGCAAATGGGTGTCAGAGATACGTGAGCCAGGGAAAAAAAGTAGAATC

TGGTTAGGGAGTTTTGAGACACCAGAAATGGCAGCAACAGCTTATGATGTTGCGGCTTTA

CATTTTAGGGGATATGATGCAAAACTTAATTTCCCTGACTTGGTTCATAAACTACCAAAG

CCAGCAAGCTCTAATGCTGAAGATATTCGCATAGCAGCCCATGAGGCAGCCATGAGCCTC

AGGCCCTCTTCAGCTGAGTCTTCCCATGGTGGCAGCTCCAGCTCGAATGCTGGTCCCGTC

ACGGTTAGGCTCTCACCCAGCCAAATTCAAGCCATTAATGAGTCGCCCTTGGATTCACCA

AAGATGTGGATGCATATGTCAGAGGTAGCAATGCTAGAAGAGTCCATGATATACTCCAAC

GACGCTGAGGAGGAGGATGAGTGGGATAACAAGCAGACAGATTCTCTGTGGGATCCTTAG

>EVM0043720

ATGGAAAACTTCCCTCCATTTATTTACAGAAACCCTAAAAGAAGCTCTAGGCAATCAAGT

AGATACCTTGGAGTAAGGAGAAGGCCATGGGGGAGATACGCTGCAGAGATTCGCAATCCA

TACACTAAAGAGAGGCATTGGCTAGGCACGTTTGACACTGCTGAGGAAGCTGCTGTAGCA

TATGATCTATCATCCATCTCTTTTAGTGGCATTGAAAAAGCTCGCACCAACTTTTACTAC

CCTCTCTTCCGGCATCCCTCGCCTCCACAAGAGGCGCCTCCACCTCCACTACCACCGCCC

GAGCTGGAGAAGGGTGATCAACTTAGTATGGAGGACATAGGCACCGCACAAGATGACGAG

TCTATTGTTATTGCTTCTATCTTGCAAAGTTTTTGCCAACCCACCAGTTATTCTTTCCAT

CCACAAATTTAA

>EVM0034620

ATGAAACCCGTCATGAAAATGGATCGTCCAGTCAAGTTCACTGAGCATAGAAACCAAACC

AAACTTACAACCCCGTTTTTGTCAAATCCGGTCCAGTACTCAAAACCACCAAAGATTGTA

CGTATATCAGTAACTGACACTTACGCAACTGACTCTTCAAGTGACGAAGAAAACGAGGTT

TCAAGAATCTCTTCTCGTAAACAGCATGTCACAAGAGTCAAGAAGTTTGTCAATGAAATT

TCGATAGAGCCAACCGCGCCACCGTCAACAACCGCTGGCTGTGTCGCAGGAGACACCGGT

TCGAGGACCAGACCGTCGAGGAGTGCTAGGAAGAAGGCTGGCATTAATGTGTATCAGGGG

AGACGTTTGCCGGCGACGGCGGTGGCGGGGAAGAAGTTTAGAGGGGTGAGGCAAAGGCCC

TGGGGGAAATGGGCGGCTGAGATTAGAGATCCTTTGAGACGTGTGCGGCTGTGGCTAGGT

ACGTACGATACTGCTGAAGAAGCTGCGATGGTGTATGACAATGCGGCTATTCAGTTGCGT

GGAGCTGACGCGCTCACTAACTTCGTCACTCCTCCGGCGAGATGTTCACCGGTGGCAGCC

ACCTCCGGGTACATGTCCGGTGATGAGTCAAATAATAACCGTAATATTAACATTGACAAT

GTTGACAATGTTGGTGTTAGCTCTCCGATTTCAGTGCTCCGGTTTAGTGAAGAAGCCGAG

TCTCAAAGTGTCGGTTCAAGCAGAGAAATCCAAGAGACCGGAAATGAAGTCCGGGAAGTA

AAAGAGGACTCCTGTGTATCGGAAAACGTTTCCGACTTTTCCGAGCATAACTCCTCGATT

GACAGTCTGTATCCTCCAACAACCGACATTTATGAATTTCCGAGTTCAGTGAGGGGTATT

TTTGAGGAAACAAGTTTTGCTGATGGTGGCTGGAACGTTCAAGACCATTTTCAAGACATT

GGTGATTTGTTCGGGTCGGATTCACTTATAGCCATTTGA

>EVM0038704

ATGGAAAGAGTGCAAGAAAACAACTTTTCTTTTGCGTTCAACCCAGCTGATATTGGCTCC

TCACTGTCACAACTTATAATGACAGGAAGAACCAGCAATTTGGATTCTATCTTCTCTCAT

TGCCAACCAGCGCGTACTCTTAACAGTCCTGTTTTGGAGCCTTTAGGTTCTTCGAATAAA

CACCCCCTATCTCACAACTTGCTAACAATCCCTCTTCAAAATCCTGTGCAAACCAGCAAT

TATTTGGCTCCAACCAAGAAGAAACTATACAGGGGAGTCCGGCAGAGGCATTGGGGCAAA

TGGGTTGCTGAAATTAGACTTCCTCAAAACAGAATGAGAGTCTGGTTAGGAACTTATGAC

ACAGCAGAAGCTGCAGCTTATGCGTATGATCGTGCTGCTTATAAACTTAGAAGCGAATAT

GCTAGGTTGAATTTCCCGAATCTAAAAGATCCTACCAGGTTGGGATTCACAGATTGTGGT

AGACTGGATGCTTTGAAGAATACTGTAGACGCCAAGATTCAAGCAATTTGTCAGAAGGTT

AAAAGAGAGAGGGCAAAAAAGAAAGCAGCAAAGAAGAGTGATGCTAATGGTAATTGCACA

AGAAGTAAAAAGTCAGTGAAAGTAGATTCAAATGCATCGAAGCCATCTCCATCATCATCA

TCATCATCAGTTTCACCTTTGTTTTCTGGTGATAAATGGGGTGGTAGTGAATTGGTGTCA

CCGGCTGTTTCTGAAGATGGTTTTTGGAAGTGTGAGAGTTCTTCTCCTTCCGTGTCAACA

GATTGTGCAGTAATGGTGCCACAAGCTATGGAATTTGAAGACTGTTCCCTTGCAAGAATG

CCATCTTATGATCCTGAGTTAATTTGGGAGGTTCTTGCTAGTTAA

>EVM0006649

ATGGACGGGGTTACCTCATTGTGCGAGTTTAGTACTTTGTCTCATCATCCTCATCGTCGA

CATAGAAATCCAAGCTTTAATTCTCTCATCTCCTGCCTAACCGAGACATGGGGTGACTTG

CCGTTAAAAATGGATGATTCTGAAGATATGGTCATTTACAATTTCCTTAGAGATGCTGTT

CGTTTTGGATGGTCTCCGTTAGACTTAACCTTACCCTCTACTACCACTGCGACAGCAACA

AATGTCGTGGAAGCCGAGCCGAAATATGAGCTGGAGCAGGAGGCCGAGCCCGTAGAAGTG

GTTCAGGAAGCTGGATTGAACGGAGCTGCTTTTTCTAAGAGGGTGGTGGCTAAAGGGAGA

CATTATAGAGGAGTGCGGCAAAGACCTTGGGGGAAGTTCGCGGCTGAGATTAGGGACCCG

GCTAAGAATGGTGCAAGAGTGTGGCTTGGGACTTACGAGACCGCGGAGGAAGCGGCTTTG

GCTTATGATAGAGCGGCTTATAGAATGCGTGGCTCAAAGGCTTTGCTTAATTTTCCACAT

AAGATTGGGTCTAATGAGCCGGACCCGGTTAGGATCACCACTAAGCGTCGAGAGCCGGAG

ACGTGTTTGCTGGCTGTGGATAGTGGCCCGGCCAAGACGAGGAGGACCGTGGTGGCTAAT

GGAGATGAGATGGGGAGGGGGAGGGGATCTGATGTGTTTCAGATTGAACACCATATGATG

CCAGCTGGTGAGCAATTATTGGTCAGTTAG

>EVM0041820

ATGGAATCTTTTGATGAAGCTTCAACTTTGGAACTCATTAGGGATACCTCCAGCTCCAGT

TCTGCCGGCTCCATCAAGACTGAATCAACAGAACTCGGCCCCTTTGATCAATCAGTGCAG

CCACAACCCATGCTGCAGAGTCAATCAGGCATCGAATCGAAGCCTCAAACCTCACCAAAG

TCTCCGAGCACTCTAAGCCAGAGAAAACCATCCATGATCAAGAACATCGCAATTCCCCCG

CCAGCAACCTTGAACATGGCCCCGCAAGTAGCCCAACCGGTGGTGAACAGGGCAGATGCT

TCAAGTGAACAGGAAAGGCATTACAGGGGCGTTAGGCGTAGGCCATGGGGCAAATATGCA

GCTGAGATCCGTGACCCTAACAAAAAAGGAGCTCGTGTATGGCTTGGAACATTTGATACA

GCCATTGAAGCTGCAAAAGCTTATGATAGTGCTGCTTTCAGGTTACGTGGAAGTAAAGCG

ATCTTGAATTTCCCTCTTGAAGCTGGGAAGTCTAATTCTCAGCAACCTGAACAATTCATG

GGAACTTCAGGCAAGAAAAGAAAGATTGAGGAAATAGAGAGCAGTGTGGAAAGTACTAGT

TCTATGATCACAACTAAGGTGGTGAAGAGGGAAAGTTCTTCGCCGGAAACTGAGGTCAAA

GCAGCGGCTACGGAACCACTAACGCCGTCCAGTTGGAAGGGGTTTTGGGATGGTGAAGTA

ACGGGGATATTTAATGTGCCACCGCTATCACCGCTATCTCCCCACCCTTCATTTGGGTAT

TCAAGGCTGATGGTTGTTTGA

>EVM0055475

ATGGCAACCCCGGAAGAATCCTCAACATTAGAACTCATAAGACAACATCTTCTTGGAGAC

TTCACCTCCACAGACGAGTTTATCAGTAATCTTGAATCCAGAATAGCCAGTGTCTCTGTC

AAACTAGAGAGCTCCCTATCCGGGTCAGAATCCAACTCCCCAATATCCGACCAAAGTTAC

CATAGCACCCAAGAAATCTATAGCTTTGAAATAAAACCTGAAATCATAGACTTGACACCT

CCTGAGCCCATGTTATCAAGTTCAAGTAATCAGTACCCACCACCTGAACCTGTAAAGATG

ACAGATAAAGGAGAGGCGGTAAGGCATTACAGAGGGGTCCGAAGAAGGCCATGGGGCAAG

TTTGCAGCAGAGATCCGTGACCCTACCCGGAAGGGAAGTCGGGTTTGGTTGGGTACTTTT

GACAGTGATGTTGATGCTGCAAAAGCTTATGACTGTGCTGCGTTCAAAATGAGGGGAAGG

AAAGCAATCCTGAATTTTCCTTTGGAGGCTGGACTATCCAGCCCGCCGCCAGCCACAAGC

AGGAAGAGAAGAAGATCAAAGCGAGAAGAGGTGCAGCCGGATTCTGTTGACGTGTCAACG

GAAAACTGGAATATAAAGTGGAGTGGGGAAGAAGAGGGTGTTTCAGATGAGGAGCAGCTG

TCACCGTTATCCCAAGAGGGGGCTTAG

>EVM0002852

ATGGAGGTGAGTGATCGTGCTGCTAGCCATGGCGGTATAGCTTCTAGCTATAGAGGAATA

CGAAAGAGAAAATGGGGCAAATGGGTGTCGGAGATTCGTGAACCTGGGAAGAAAACTAGA

ATTTGGTTAGGCATTTTACATTTTAGGGGACGTGGAGCAAAACTTAACTTTCCTGAATTG

GCTGGTAAGCTACCAAAGCCAGCAAGCTCCAATGCTGACCACATTCGCATGGCAGCTCAT

CAGGCATCTATTAGCCTCAGTCCCTATACTGCTGACTCGTCCCACGGCGGCGGCTCTAGC

TCCAATGTCGGGCCTATCACGGTCAGGCTCTCACCAAGCCAAATTCAGGCCATCAATGAG

TCACCATTGGATTCACCAAAGATGTGGATGCAAATGGCAGAGATAGCAATGCTAGAGGAG

TCCTCCATGATGTTCTCTAATGACAATATTGAAGAGGATGGGTGGGATAACAACCAAACA

GATTCTCTTTGGTAA

>EVM0001533

ATGGAGGATTCGTTGAGAAAGAGTTCTTTGCTAGGGTTTAGCAGCAAGAACAGGAAGAGA

TGGAAGAGAAGCAGGGATGGTTGTGATTCAATTGAAGATACACTTGCAAAATGGAAGAAA

CACAATAAGCTTCAAATAAGCAAGATTCCAGGGAAGGGTTCAAAGAAGGGTTGTATGAAA

GGAAAAGGTGGCCCGGAGAATATGAACTTCAGATATAGGGGAGTTAGACAGAGAACCTGG

GGCAGGTGGGTTGCTGAAATCAGAGAACCTGTCAAAAAATGCAGTTTAATGAACAAAAAA

GGAAGTAGACTCTGGCTCGGTACATTTTCTACTGCTTTTGAAGCTGCTTGTGCTTATGAT

TATGCTGCGAATCTTATGTATGGTCCTAATGCCATACTCAATTTCCCTGACTACCCGGTT

GAATCAGGGGGTCACTTGAATAATATGTCGTCTTCGATTACTGCAACTGAGACATCATCA

ACTGAATCTAGAACGGCATTGGATAACCACGAGGATAATAAGGTTGACAAATCAAAGATG

AATCATTGTGGGTATATAGAAGGAAATAATCTATCAGGCTTTTCTAGGATTTGTTCTGTC

GATGAATCAGGAGAAGAAAATAAATTTAGAGCGGCGGAGAGCAGTGCTAGGGAGTTGAAA

GATGTGGAATGGAATTTAACAGATGATTGGAAATCGTCTCATCATATTGAAGCTGGAGCA

CCTGTATCGAGGGAAGAAATGGATGGAGAACTTGCAGAAATTTTTAGACCATGGGGCTGT

TACGGTATCAATAACAGTCTTAACAGATATGGACTCTTGCAGAATGAGACTGCAAATGTA

GATTATAAAAAATTGAGGAATGAAGTTGTGGAATCCAGCATGAGCACCAGACTTAATGAG

TTCGTAGATTCGGACTATGATATGAGAACTGATCATAAACCTATCTATGATGTTGAGAAG

CCATTAGTGAGGGAAGCAGCAGCAGGGGAAGAGTTTACAGGCTTGAAATTCAGTAACTAT

AATCACAATGAAACTACGCATGATTACATGAACCCGGGTTTATGCAATCAAGAAATTGAT

ATCAAGCCTTTTATCCAAGATATTTCATATAATTCTGTATTAAAAGGAGGGATGAGTTAT

GGTTATGATCCCGGGAAGGTTGGTTCTCCAAGCCACGTACAGGGTGGAAGGCCATCTACA

CTGTCCTGCCATTGGCAGACCCCATCAACCAATCTGCCAGGGAGCTCGAGTTACTTTCAG

GAGGCAGATTTAGGCCCTGGTTGGAATGTTGATCTATCACAGCAGTATTTCAACGGGAGC

TTAGTTGGAGAGCCAGGCATGCTTGATCCGTGGTGCCCTGATTTACAATTCTAG

>EVM0056869

ATGGTGCAATCAAAAAAGTTCAGAGGGGTGAGGCAGCGACGGTGGGGTTCTTGGGTTTCT

GAAATCAGACATCCTTTATTGAAGAGGAGGGTATGGCTAGGAACTTTTGAAACAGCAGAA

GAGGCAGCGAGGGCCTATGATCAAGCGGCAATCCTGATGAGTGGCCGTAATGCGAAAACT

AACTTTCCCATGCCTCAAACTTCGAATGAAGATGACCCAAAATCAGGTGATCAGCCTCCA

CTCACAGCACCACCAAATGGTTTGTCCCAGATCCTCCATGCCAAGCTAAGAAAGTGCAGC

AAGGCACCATCACCATCAATGACTTATTTGAGGCTTGACACTGAAAATTCCATTGGGGTT

TGGCAAAAGCGTGCGGGTCAGCGGTCCAACTCGAACTGGGTCATGACAGTGCAGCTTGGG

ACGAGAGATAAGAGCCAAGTTTCTGAGAGTACATTGCCATTGCCTGATCAATCTTCAGGA

GGGATATCAGGACCAGAATGCAGAGCAGAAATGGATGAAGAAGAGAGAGCTGCACTGCAA

ATGATTGAAGAGCTTCTTAACAGGGATTGTCCTAGCCCTCCTTCTGGGGTTCTAGATCAT

GGGGATGGTAGCTTTTTTCTTTAG

>EVM0057140

ATGGAGAATTGTAGAGAATCTCCATTGAAGCCGTGGAAGAAAGGACCAACAAGGAGCAAA

GGTGGTCCTCAAAACGCCATGTGTGACTACCGAGGGGTCCGTCAAAGAACATGGGGAAAA

TGGGTGGCAGAAATAAGGGAACCCAAGAGGAGATCCAGACTATGGTTGGGTTCTTTTGCT

ACGGCTGAAGAAGCTGCCATGGCCTACGATGAGGCTGCAAGGAGACTGTATGGACCAAAT

GCGTATCTTAATCTACCTCACCTTCGGTCAAACTTTAATCCTCCCAACAAATCACATAAG

TTCAAATGGATTCCTTGCAATAACTTCATCTCAATGTTTCCATCTTGTGGGTTGCTTAAT

ATTCATGCACAGCCTAGCGCTGATGTCATCCATCAGCGGCTCCAAGAACTCAAGAATAAG

AGAGCCCTTAATCAACCCTCTGTTGCTTCTAGCTCTTCTTCCTCTGAATCCAGAACTGAA

GTTATAATTGTAAGTGACGAAAACCATGTTGCAAATGTTTCCGTAGCTGAGAAAGAAAAG

GAAATATCATCAGAGACGATGCTGCTAACAAATCATGACGAGAAACCACAGCTTGATTTA

AACGAGTTCCTTCAGCAGCTGGGCATCCTGAAAGAAGAGAAACAGCCAGATAATAATGCT

GTCAATGAAGATTTCATGGAGCCAGAATCTTCACGAAAAAGTTTCGATTGGGACTCAATG

AATGAAATGCATGGAATTGCTGATCATCAAGGAGAGGAATTGAATAGTTTCCCAGTCTAT

GACATCCAAGAAGAGCTGGCTTTCCCATCTTCCATTTGGAACTTCTAG

>EVM0015665

ATGGCGTCTTCTTCTTCTTCTGATCCGGTTCTAAAACCAGAAATCGGCGGCGGAATATGT

GGTAGCGGAAGCGGCGGCAGAGGTGGAGGAGAGAGTTCTGAGGCGGCGGTGATAGCAAAC

GATCTGTTATTACTATACAGAGGACTAAAGAAACCAAAAAAGGAGAGAGGATGCACCGCT

AAAGAACGTATTAGCAAGATGCCTCCTTGTACTGCTGGTAAACGTAGCTCCATTTACCGT

GGCGTCACCAGACATAGGTGGACTGGTCGATATGAAGCTCACCTTTGGGATAAGAGTACA

TGGAACCAGAATCAGAATAAGAAGGGAAAGCAAGTATACTTGGGGGCATATGATGACGAG

GAAGCTGCAGCTAGGGCATATGATCTAGCTGCTTTGAAATATTGGGGCCCTGGAACACTC

ATTAATTTTCCAGTTACTGATTATACAAGAGATCTTGAAGAAATGCAGAATGTCTCCAGA

GAAGAGTACCTGGCATCTCTTCGGAGAAAGAGCAGTGGATTTTCAAGAGGAATCTCAAAG

TACCGTGCACTGTCTAGTCGATGGGACTCATCTTATAGTCGTGTGCCAGGATCTGAATAC

TTCAGTAACGTAAACTATGGTCCAGGTGATGATCAAGCAGCTGAAAGTGAATACAGTTTT

TGTATTGAGAGAAAGATAGATCTGACAGGCTATATTAAATGGTGGGGTTCCAATAAAACT

CGTCTGGCAGAGTCTATGACAAAGTCATCCGAAGATACAAAACATGGCTGTGCTGATGAC

ATTGGCAGTGAACTCAAAACATCAGAACGAGAAGTGCAATGTACTGAGCCATACCAGATG

CCCCGTTTAGGTTTATCCGTTGAAGGAAAAAGGCATAAAGGTTCTAAGATATCCGCGTTG

AGCATTTTGTCACAGTCAGCTGCATACAAGAACTTTCAAGAGAAGGCATCAAAAAAACAA

GAAACTGGCCCTGAAAATGATGGGAACGAAAATAGAAATAATATCAACAAGATGGACCAT

GGCAAAGCAGTCGAGAAGTCCACAAGTCACGATAGCAACAGTGAGAGACTTGGAGCCGCA

TTAGGAATGACTGGGGGATTGTCTCTTCAAAGAAATGCGTATCCATTGACTCCTTTCTTG

TCTGCTCCTCTTCTGACCAACTACAACACAATCGATCCCTTAGTAGACCCCATCCTTTGG

ACATCTCTTGTTCCTTCCCTTCCTACTGGACTTTCTCGAAATCCTGAGGTTACAAAGACT

GAGACCAGTTCAACTTATAGTTTCTTTCGGCCAGAGGAGTGA

>EVM0012873

ATGGAAGAGCCCGGCAAAGGGAAGGAAAATGATGAGAAAGGGAGGGGAGAGGTTCGATAC

AGGGGTGTTCGAAGGAGACCTTGGGGAAAATTTGCGGCGGAGATACGGGACCCGTCAAGG

CAAGGAGGAAGGTTATGGCTAGGGACATTTGATACTGCAGAGGAGGCAGCTCGGGCTTAT

GATAAAGCTGCATTTAACTTGAGACGTCAACTCGCAAATCTCAACTTTCCTAGTGATTTT

ATCTCTCATGTGATGGGGTATTCTCCTCGTCCTCAATCATTCCCACCATCTAGCTGTATT

GTTGGTGCAACTGAGAGTTATGAGAGGGGAAGCTCATCAAGTACCGGACAAGGAAAGCAA

GTTATCGAGTTTGAGTACTTGGATGACAAGATTTTGGAGGAGCTTCTTGAAACAGAAGAG

GAGAAGAAGAAGCGGCAGCAAGATTGA

>EVM0033385

ATGAGTGTTGACATGGAAGATTCGTCGAGACAGAGTTCTTTGCTGGGGTGTAGCAGCAAG

AACAAGAAGAAGTGGCGGGGAAGGCGGAATGGTTGTGAATCGATAGAAGATACAATCGCA

AGGTGGAAGAAACACAACAAACTTCAAATAAACAAGGTTCCAGGCAAGGGGTCGAAGAAA

GGTTGCATGAAAGGAAAAGGTGGCCCAGAGAATAATAGCTGTAGATATAGAGGTGTTAGG

CAGAGAACCTGGGGCAAGTGGGTTGCTGAAATCAGAGAGCCTGTCAAAAAAGGCAGCATA

ACGAAGAAACGAGGAAATAGACTATGGCTTGGTACATTCTCTACAGCTATTGAGGCAGCT

CGTGCTTATGATTATGCTGCGAGAGCCATGTATGGTCCTAATGCCATACTCAATTTCCCT

GACAACTCTCACGAATCAGGGGATCAGCAGGATAGCTTATCGTCTTCGGCTACAGCAACT

GAAACGGCATCAACTGGGTCTAAAACAACACTTGATAACTATGAGGATTTCAAGGTTGGG

AAATTGAAGGTGGGTTATTGTGGGTCAAGAGTGGTGAATAAAGAATCAGGCTGTTCTGGG

ATTTCTGCTGTCAATAAATCGGGGGAGGAAGCTGAGAAGTTTCAGGTGGCGGAGTGCAGT

GGAAGGGGGGTGAAAGATGGGGAATGGAATTTAGCAGATGATTGGATGTCTTCTCATCAT

GTTGAGGCTGAAGCACCGGTGTTGAGGAGAGAAATGGATGGAGAACTTGCAGAAATTGTG

GAATCTTGCTGTCACGGTATCAACGACAGGAATGACTTCTTGCAGAACGAGACCGAAAGT

GTAGAATATAGATTGAAGAACGAGACTGCGGAATCCGGATTGAGCGACAGACTCGATGAA

TGCTTGTATTCCGATATGGAAACTGATCGCAAACCTTTCTACGACGCTCAGATGCCATTA

ACGACGAAAGCAACAGGAGTAGAGTTTTCAGGGATGGGATTTAGTAACTTTAATAATCAG

TTTGAGGTAGGGCATGACCAATTAAACGAGGGTCTATGCGATCCAGAGATGGATACCAAG

CCTTTTAGCCACGGTGTTTCTGATATCCCTGCGTTAAAAGGAGAGGGGAATTACGAGTAT

GTTCACAGTGACGTTCATGCTGCAAGCCAGTTACAGAGTGGAAGGCCACCTGAACTCGCC

AGCCAGTTGCAGCCCGCAGGAACCAATCCATCAGGGAGCTTGAGCTACATTCAGGAAGCA

GATTTAGGTGCTGGTTGTAATTTTGATCTATCAAATCAGGACATCAACTGGGGATTAGTT

GGAGAGCAAGAATTGCTTGATCCCTGGTTCCCTGAATTACAGTTCCAGTAA

>EVM0018037

ATGGGGAGAACAAGAACAACAACAAAACAAGCTGCTGACCCGAATGGATCTGCAACCCAG

AATATGTTAGTAATTGCAAAAGAGCCCAGATACAGAGGAGTACGAAAGAGACCATGGGGG

AAATTCGCTGCGGAGATTAGAGATCCCTGGAAAAAGACCAGAGTTTGGCTGGGCACCTTC

GACTCTGCAGAGGATGCAGCTCGTGCCTACGATACGGCTGCTCGCTCCCTCCGGGGAGCA

AAGGCCAAGACAAACTTTCCTACCTCTGCAACGAACCAGTTATTCAATCATCCAAGCCAA

GCCCCGGCTGATCCCTTCTTGGATCACCACAGTATAAATCCTCAAAGACCCACATCTAGC

AGTTTGAGCAGTACAGTGGAGTCTTTCAGTGGTCCTAGGCCTCTGCAGCCAACAACAATG

AAATCGGGAAGTGGGCCGAGGAGATCTCATCCACGGATCCCACCGGTTGTTCCAGAAGAT

TGTCACAGCGATTGTGATTCGTCTTCTTCGGTGGTGGATGACCGAGATATCGCATCCGCT

GCTTCTTCTTTGTGCCGCAAGCCTTTGCCTTTCGATCTCAATCTCCCACCGTTGGATCAG

GTTGACTTTGGCTCTGGCGATGATCTCCACTGCACTGCTTTATGCCTTTGA

>EVM0002769

ATGCAAAGATCTTCAAAGAGGTCCAGAATCAGTGAAGCTCCCCCAGCAACTGTCTTTTCA

CCGCCGCCAGTTCCACCGCTAAGATTGACCCACGAGCAGGAGTTGGCTGTAATGGTTGCT

GCTCTCAAAAACGTAATTTCTGGCACAGCTTCAATGGATTTCTCAAGGGAGATAAATAGT

ATCAATATGCCATTCAACGCTTCACATTCACAATTTGGAAGTACAAGCAGTAACGGGAAC

GATTTTTACAGCTCTATTTTGCCTCCATCTTCGGATCTTGACACGTGTGGTGTTTGCAAG

ATCAAAGGGTGCTTAGGATGCAACTTTTTCCCGCCAAATCAAGAAGATAAAAAGGATGAC

AAGAAAGGGAAACGAAAGAGAGTAAAGAAGAATTATAGAGGTGTAAGGCAACGGCCATGG

GGAAAATGGGCTGCAGAGATAAGAGATCCACGGAAGGCTGCAAGGGTTTGGCTAGGGACG

TTTAACACTGCAGAGGAGGCGGCAAGGGCTTATGACAAGGCAGCCATTGATTTTAGAGGG

CCAAGAGCTAAGCTGAACTTCCCATTTCCTGATAGTGGCATTGCTAGTTTCGAAGAGAGG

ACAGAGAAGCAAGAAAAGCAGCAGGAAATCAGTGACACGAGAAGTGAGTTTGAAGCGGAA

AAGAACGAGTTCTGGGATAAGACTGCAGACGAAGAGTTAAAAGAATGGATGATGATAATG

ATGGATTTCGGTAATGGTGACTCTTCCGATTCTGCCGGTACTGCAAGTGCTGCTGCTACT

ATTGGTTTTTAA

>EVM0043678

ATGCAGAGACCCTCGAAGAGATCCAAAACAGGTGAAGCTCCGGCAGCGACCCTCGTTTCA

CCGCCGGGAATTCTACCTGTAAGACTGACCCAAGATCAAGAGTTGGCAGTAATGGTTGCT

GCTCTCAGGAACGTAGTTTCTGGCGCCAGTTCAACTGATTTCTCAATGGAGTTAAATAGT

TTTAATGAGCCGATCACCACTTCACATTCACAATTTGGAAGTACAAGCCGTAACGAGAAT

ACTTTTTGCAACTCAATATGGCCTCCACCGTCGGATCTCGACACGTGTTCTGTATGCAAG

ATCAAAGGCTGTTTAGGATGCAACTTTTTCCCGCCGAATCAAGAAGATAAAAAAGACGAC

AAGAAAGGGAAAAAAAAGAGAGTAAGGAAAAATTACAGAGGTGTAAGGCAACGGCCTTGG

GGAAAATGGGCGGCAGAAATAAGAGACCCGCTGCGGGCTGCAAGGGTTTGGTTAGGGACG

TTTACCACAGCGGAGGAGGCGGCAAGGGCTTATGATAAGGCAGCTATTGGTTTTAGAGGG

CCAAGAGCGAAGCTTAATTTTCCATTCCCTGACGGTGGTATTTCTAGTTTTGAAGAGAGT

AAAGAAAAGCATGGAAAACATGTGAAGCAGCAAGAAATAAGCGAGAACAGAAGTGGATTT

GAAGCGGAAATGGGGAAAGAAAGTGAGTTCTGGAATAAGATTGGAGATGAAGAGATACAT

GAATGGATGATGATGATGTTGGATTTTGGAAATGGGGATTCTTCGGGCTCTATCGGTACT

GAAAGTGCTTCTACTATCGGGATTTGA

>EVM0030823

ATGGAAATTCATTTCCAGCAACAAAAGACGCATCGAAAAGAAAGCATGCCAGTTAGCAGA

ACAACCAAATTAAAGGGGACAAGAAGGCCTAGAAACAGCAATAAATTTGTTGGTGTTAGG

CAAAGACCTTCTGGGAGATGGGTTGCTGAGATCAAATACACGACACAGAAGATAAGGTTG

TGGCTCGGAACTTTTGAGACTGCTGAAGAAGCTGCTCGAGCTTACGATGAAGCTGCTTGC

CTGCTTCGTGGATCAAATACTCGAACCAATTTCATCACTCGTGTATCCTTGGATTCTCCT

CTAGCTTCCCGGATTCGAAACCTCCTCAACCAGAAAAAGAGAGAAAAACAGCAGCAGAGT

GAGGAAGAAGAAACTTCTGATGTCTCGGCCCCACCAACTAGCACCATCATTACCACAAGC

ACTGGTAGTTTCATTAGTAGTTGTGGCAGGAGTAGTGATAATTACTCCAATTCCAGCTCG

GTTCATGGAGCTGATAACTTGTTTGATGATGCATATAAACCAGATTTAAGCAGCACTTCC

ATTGATCAGGAATTCAAGTGGGGTTCTTCTCAGACTGATCTTTCATGGGGTTTTGGACCT

GTGTTCGATCGGTTTACATTTTCCCAGGAAGTGCTGGATTTTCCAAACAGTGTGGTATCG

CCCGAGACAAGTGAGTTGGAATTCTCAGAATTTGATAAGATGAAGGTGGAGAGACAGATA

TCAGCATCTCTATATGCGATGAATGGGGTGCAAGAGTACATGGAAGCTATCTATGATCCA

ATTGAAGCTTTTTGGGATCTCCCACCACTATATTAA

>EVM0003762

ATGGGGAGAACAAGAACAACAACAAAACAAGCTGTTGACCCGAATGGATCTGCAACCCAA

AATATGTTAGTAATTGCAAAAGAGCCCAGATACAGAGGAGTACGAAAGAGACCATGGGGA

AAATTCGCTGCGGAGATTAGAGATCCCTGGAAAAAGACTAGAGTTTGGCTGGGCACCTTC

GACTCTGCAGAGGATGCAGCTCGTGCCTACGATACGGCTGCTCGCTCCCTCCGCGGAGCA

AAGGCCAAGACAAACTTTCCTGTCTCTGCAACGAACAAGTTATTCAATCGTCAAAGCCAA

ACCCAAACCCCAGCTGATCCCTTCTTGGATCACCACAGTATAAATCCTCAAAGACCCACA

TCTAGCAGTTTGAGCAGTACAGTGGAGTCTTTCAGTGGTCCTAGGCCTCTGCAGCCAACA

TCAACAAAATCGGGAAGTGGGCCGAGGAGATCTCATCCACGGATCCCACCGGTTGTTCCA

GAAGATTGTCATAGCGATTGTGATTCGTCTTCTTCTGTGGTGGATGACCGAGATATCGCA

TCCGCTGCTTCTTCTTTGTGCCGCAAGCCTTTGCCTTTCGATCTCAATCTCCCACCGTTG

GATCAGATTGACTTTGGCTCTGGCGATGATCTCCACTGCACTGCTTTATGCCTTTGA

>EVM0008628

ATGGAGCCACTACCATACACTGAAGATGACCCGGCTACAAATCCAGAGGCAGCCACCGCG

CTAGCCCACAAAATTTCAGACACTATCCGGGGAAGCGGCACCCGACATCCCGTCTATCGC

GGTGTCCGAAAACGGAGGTGGGGGAAATGGGTGTCTGAAATTAGAGAGCCTCGCAAGAAA

TCACGTATTTGGCTAGGCTCATTTCCCGTGCCAGAAATGGCAGCCAAGGCATACGACGTG

GCAGCGTATTGTCTTAGAGGTCGCACGGCGCAACTTAATTTTCCTGACGAAGTCGATGAG

CTGCCTAGACCATCGACGTGTACGGCCAGGGATATTCAAGCTGCAGCAGCTAGGGCTGCA

CATTTGGTGCTGATTCCAATGGAAAAGAGCAACGAAAAGAATAGTGATGGTGGTAGCGAC

GGTGAAGTTGCTGCTGATGATTTCTGGGGCGAGATTGAGTTGCCGGAATTGCTGCTGAGT

AATAGCAGGTACAGTTGGGATTGTTGTGGATGGAGTACTACTCTTGGTCTTGCAAGTGAT

AATTCAACGTGGCAGCCGGATGGAGAGGGTCTACAACCATCCTCGGCACGTCTTTAA

>EVM0054355

ATGAATCGCTTTTCTACCAAGTACACCGAGCATAAAACAGTGACCACCAAGCTGGTGAAA

TGGGAGGACTCCAAAGCCACAAGAATAGTACGGATTTCAGTTACCGATGACAATGCTACG

GACTCGTCTGGTGATGAAAATGAGGAGTCGAAAAGGCATCATCCAAGAGTTAGAAAACAC

ATAAACGAGATAAGAATATCTAATTGCGGGAGCGACAGAGCAGCAGAGGATGCAAAAAAA

TCTTCAAAAGTGGTAAGCCGGCAACAGGTTGTGAAAAATATTTCAAGTGACCAGTGTTAT

TATCCAGGGGTGAAAAGGTATCGCGGTGTGAGACAAAGGCCATGGGGAAGATGGGCAGCG

GAGATCAGAGACCCTTATAGGAAAACCCGGCTATGGTTAGGGACTTTTGATACAGCAGAG

GAGGCAGCCATGGTTTATGATCAGGCAGCTATTCGTATCAAAGGTCCTGATGCACAAACC

AATTTCACAAAACCTCCGGTGAGCAAACAGCATACCCGAGATGTTGATATCAATGTTAAC

ACTTCCGGGTATGATTCTGGTAAAGAATCTCATAACTGTCTATGTTCTCCAACTTCTGTG

CTACGATTTCAATCTACCGGGGAACCGGGGGCAGAGTCTCAGGTTTCTGTGCAGAGTGAT

TGTTACTGGAGGATACAAACGCAAGAAGAGGTGGTTAGAGAAGAAATAATAAAGGTTGGT

GAAGATGATGATGAGTGTTTGGTTACGGATCCATCATCGTGTTTGGATGCGTTTTGGGAT

TTTGAAAATCCTGCTCCGATATTTTTTGAGGAGTGTAGAGTACTGGATACTGTTTCGAGA

GAAGATTATGCTGATATTCCGGTACATTTTGATGGTGATTTTGGTTCTTGTTTATGGGAT

GTCGACAAGTATTTTGAGGCTTAA

>EVM0046342

ATGGAATTTCATCAGTTCCAAAAGCAGCCTAGAGGAGTCCCACCTAGCAAACCAAGCAAA

TTCAAGGAGAGATGCTCAAGCAGCAACTCCAAAAACAAGTTTGTTGGAGTTAGACAAAGG

CCTTCAGGGAAATGGATCGCAGAGATCAAAGACACTACTCAGAAGATCAGAATGTGGCTG

GGGACCTTTGAGACAGCTGAAGAAGCTGCTCGAGCTTATGACGAAGCTGCCTGTCTTCTT

CGTGGTTCGAATACCCGAACCAACTTTAACACTCATGTTTCTTCCAGCTCTCCTATTTCT

GCGAAAATTAGAAACCTGATCCATCACAAAAGGAGTTTGAAACAAAACCCTCCCGCAACT

TCCTCCACAAAATCTACTATTAAAGCCAGCACTATAGTTAGCACCACGAGCAGTTTCAGT

AGTAGCATGGATAAACCATCCCTAATGTTTGATGATGTATATAGACCAGACTTGAGTGGC

TGCATTGGAGGGCTTGAACCGGTTACGTCTCAGTTCCATCCCTCATGCTCGTTCCCATCT

GGGTTGGATCCCCCTATTCCATATATTCAAGAAGGAACGGAGCTGCCAAAGAATGTTGGT

TTGTTGTCTGATGCATCGTCCTGCGTAGAATTAGCCGAATTCGAACGCATGAAGGCGGAA

AGGCAGATACCAGCATCATTTTATGTCATGAATGGAGTGAATGAGTACTTTGAGAATGCT

AATGATTCAAGTGAGGCTCCCTGGGATCTCCCTGCACTCTGTCAATTTTCTGTCCTAGTT

AATTCTCAAAAAAAAAATTAA

>EVM0017324

ATGTGTGGCGGTGCTATTTTAGCTGAAATCATCCCTCGCAACCGCGCCCGCCGCGAGGCA

GCTTCAGAGTTCTGGCCGAACGCAACCTTCAATAAACTCAGTCCTTTTGATTCCTTTCTG

AGCCCACTTAGCAACCAGGAGGAGTCATTCACTCTCAAACGACCCCGACCCCAGCCCCAG

CCCCAGCCCATTTCTGAAACAGCGGAAGTGGAGAAACCCAATGCCAAGAGGCAGAGGAAG

AATCTATACAGAGGTATAAGGCAGCGACCTTGGGGTAAATGGGCAGCTGAGATTCGTGAC

CCAAGAAAAGGAGTCCGTGTTTGGCTCGGTACCTTCAACACTGCTGAGGAAGCAGCTAGA

GCCTACGACAGAGAGGCTCGCATAATTCGCGGCAAGAAAGCTAAAGTCAATTTTCCTAAC

GAAGACGAACATAACTATACCTCTCAAAATTCAAAAACCAAGCCTCCTATGTACCCAGCC

CCCACATGTCATTTCTCAAAGGGTTATGATTTTGGGTACGGTTCTGGTATGAATCAGATT

GAATCCTACTCTTCAAATGGTTTTAATGGCCAGCCAATTGTCACTTCTGGGGATGATTCT

GGGTCCACATCAGAGGAAGGAACAAGACTGGTGGACTGTAATCACAACTTGGAGAGTGAT

GTTTATATGGGTCAAGTGAAGCTGGAAATGGAAGAGGAGAAGCTAGAGAAGGTGAAGAAC

AAGGAGGCGGTGCTGGTGGATTTGGAAACTGGAATGGAAGAAAACGAGGTGCAGAAACTG

ACTGAGGAGTTAATCGCGTATGAGAATTACATGAAGTTTTATCAGATTCCCTATCTGGAT

GGGCAATCCATGGCACCAAATGGTTCCACCCAGGAAAACCTTGTGGGTAATCTTTGGAAT

TTCGATGATGACAGCGTCGCTCCTCCTGTCACCTCGGCACCTCTGTAA

>EVM0052698

ATGGAGGATTCGTTGAGACAGAGTTCTTTGCTAGGGTTTAGCAGCAAGAACATGAAGACA

TGGAAGAGAAGCAGGAATGGTTGTGATTCAATTGAAGATACACTTGCAAAATGGAAGAAA

CACAACAAGCTTCAAATAAGCAAGGTTCCAGGGAAGGGTTCGAAGAAGGGTTGTATGAAA

GGAAAAGGTGGCCCGGAGAATATGAACTTCAGATATAGGGGTGTTAGACAGAGAACCTGG

GGCAAGTGGGTTGCTGAAATCAGAGAACCTGTCAAAAAATGCGGTTTAATGAACAAAAAA

GGAAGCAGACTCTGGCTTGGTACATTTTCTACTGCTTTTGAAGCTGCTTCTGCTTATGAT

TATGCTGCGAGGCTTATTTATGGTCCTAATGCCATACTCAATTTCCCTGACTGCCCGGTT

GAATCTGGGGGTCACTTGAATAATATGTCGTCTTCGATTACTGCAACTGAGTCATCATCA

ACTGAATCTAGGAAGGCATTGGATAGTTACGAGGATAATAAGGTTGACAAATCAAAGATG

AATCATTGTGGGTATAGAGAAGGAAATAGCCCATCAGGCTTTTCTAGGATTTGCGCTGTC

GATGAATCAGAAGAAGAAGTTGATAGATTTAGGGTGGCGGAGAGCAGTGATAGGGAGTTG

AAAGCTGTGGAATGGAATTTAAGAGATGATTGGAAATCGTCTCATCATATTGAAGCTGAA

TCGCCTGTATTGAGGGAAGAAATGGATGGAGAACTTGCAGAAATTTTTAGATCCTGGGGC

TGTAACGGTATCAACAACAGATATGGACTCTTGCAGAATGAGACTGAAAATGAAGAACAT

AAAAAATTGAAGAATGAAGTTGTGGAATTCAGCATGAGCACCAGACTTGATGAGTTAGTA

GATTCGGACAATGATATGAGAACTGATCATAAACCTATCTATGATGTTGAGAAGCCATTA

ATGAGGGAAGCAGCAGCAGGGGAAGAGTTTTCAGGCTTGAAATTCAGCAACTATAATCAC

AATGAAACTGCGCATGATTACATGAATCCGGGTTTATGGAATCAAGAAATTGATATCAAG

CCTTTTATCCAAGATATTTCTGTATTAAAAGGAGGGATGAATTATGGTTATGATCCCGGG

AAGGCTGGTTCTCCAAGCCACGTACAGGGTGGAAGGCCATCTAGACTGTCCTGCCATTGG

CAGACCCCATCAACCAATCTGCCAGGGAGCTCGAGTTACTTTCAGGAGGCAGATTTAGGC

CCGGGTTGGAATGTTGATCTATCACAGCAGGACTTCAACGGGGGCTTAGTCGGAGAGCCA

GGAATGCTTGATCTGTGGTGTCCTGATTTACAATTCTAG

>EVM0008451

ATGAATTCTAACAACTGGCTTTCGTTTCCTCTTTCTCCCACTCACCCTTCCTTGCCTGCT

CATCTACATGCATCTCACCCTCATCAATTCTCTCTAGGGTTAGTCAATGATAGTATGGAA

AACCCATTTCAAACTCAAGAGTGGAGTCTTCTTAACACTCACCAAGGCAACAATGAAGTG

CCAAAGGTTGCAGACTTTCTTGGTGTTAGCAAATCTGAGAGTCAATCAGATCTTGTAGCC

TTCAATGAAATTCAAGCTAATGATTCTGACTATCTCTTTTCAAGCAATAGTCTAGTACCA

GTCCAAAATGCTGTTGTAGGCGCAAATAATACCTTCGAGTTTCAAGAAAATGCCAGCAAT

TTGCAGTCATTAACACTCTCTATGGGTAGTGCTAGTTGTAAAGGTTCGACATGTGAACCC

AGCGGTGATAGTAGCACTAATACTGTCGAAGCTGCTGCACCAAGAAGAACTTTGGATACA

TTTGGGCAAAGAACATCCATATATCGTGGTGTAACAAGGCATCGATGGACAGGAAGGTAT

GAAGCTCATTTATGGGATAATAGTTGCAGAAGAGAAGGTCAATCTAGGAAAGGAAGACAG

GTCTATTTAGGTGGCTATGACAAAGAAGAAAAGGCAGCTAGGGCTTATGATCTTGCTGCA

CTTAAGTACTGGGGAACATCCACCACTACCAATTTTCCAATCAGCAACTATGAGAAAGAA

ATAGAGGAAATGAAGCACATGACTAGGCAAGAATTTGTGGCCTCCATTAGAAGGAAGAGT

AGTGGCTTTTCTAGAGGTGCATCCATGTATCGTGGAGTTACAAGGCATCACCAGCATGGT

AGATGGCAAGCAAGGATAGGCAGAGTTGCAGGAAACAAAGATCTCTACTTGGGAACATTT

AGCACTGAGGAGGAGGCTGCAGAAGCTTATGACATAGCAGCAATAAAGTTTAGAGGGCTT

AATGCAGTGACTAATTTTGACATGAATCGATATGATGTGAAGAGCATTCTTGAAAGCAAT

ACTTTGCCAATTGGAGGAGGGGCAGCCAAACGGCTAAAGGAGGCTCAAGCAATTGAATCA

TCACGAAAAAGAGAAGAAATGATTGCTCTTGGATCAAGTTTTCCATATGGATCAAGTTCA

AGCTCTAGCAGGCTACAAGCTTACTCTCTAATGCAGACACCATTTGAGCAACCTCAACCT

TTACTTACTTTACAGAATCAAGACATTTCTCAGTATACTCAAGATTCCTCACTCCAACAA

AACTTTCTTCAAACTCAGCTTCATTTGCACCAGCAATCTACAGGATCTAATTTCCTTCAG

AACCAATCAAGCCAGAACCCTCAGTATTACAACAGTTATATTCAAAACAATCCAGCTTTA

CTTTATGGAATCTATGGTACTGGAGGTTATCTGGGAAATGGGCTGGGAATTGCTTCCAAT

TCAACAGGGTCTAATGCAGTAGGATCAGCTGAGGAACTTGCACTTGTCAAAGTTGATTAT

GATATGCCTCCTAGTGGCTATGGTAGCTGGTCTGGGGACTCAGTTCAGGGATCCAATCCA

GGTGTTTTCACAATGTGGAATGAGTGA

>EVM0021344

ATGATGACAGAAGAAAGCAGTGGCAGCTCAAGGAATTCAATATGTAAAACGTCGTCGTCG

TCCTTCGCCACCGCCACCACCCACAAGCTCGAGAAACAAAACCCAGTTAACCAGAAGTCG

AGAAAGGCAAGAGACTGCAGCAAGCACGCGGTTTACAGGGGTGTTAGAAAGCGAGCCTGG

GGCAAATGGGTGTCCGAGATCCGCCAGCCTCGTAAGAAATCACGTATCTGGCTCGGAACA

TTCCCTACGCCGGAAATGGCAGCTCGAGCTCACGACGTGGCTGCCCTGAGTATTAAAGGT

AGCTCTGCCATTTTGAATTTTCCAGAACTTGCTGCCTCATTTCCGCGCCCAGTGTCATTA

ATGCCACGTGATATTCAGACAGCTGCGGCTAAAGCTGCTGCCATGGTCGGGTTTAATTCA

TCGTCACCGCCCTCATCATTGTCGTCGTCGTCGGTCTCGGTGGCTGGAGATGTGGCAGAG

TCCGAGGAAGAATACTTGAGTGAAATCGTGGAGTTACCGAATATAGAAGGAAGCTTTGAC

TCTCCAGACCAGTCACAGCCCGAGTTCATGCTGTTCGACTCGGTTGATAGGTGGGTGTAT

CCGCCACTAGACTTGAGTGGAGAGTTTTCCGATCAGTTACTGTGCCTGGAGAGCTTGATT

TCTAGCAACTTTGGAGGATCTCTGTTGAATTAA

>EVM0049776

ATGGCAGCTACAATGGATTACCACAGTGGTAGATCGCTTCAATCAGACCTCCTTGGGGGT

GAATTAATGGAAGCACTTGAAACTTTTATGAAAAGTGCTTCCTCTTCAACTACCCCATCT

CCTTCTCAAACCCCTAATTATCCTTCTCCCCCCTCTACATCATCCAATTACTCTTCTTTC

TCTCCACAATCACTTCTGCAGCATCAGCAGTCCTTTTTCAACCCAGATGGTTGCTGCTCT

ACATCGACGACCTATCCATTTTCAACTGGGTTCTCGTTCGACGACCCAATGGGTCTCCAG

CAACCATATAGCTCAATTGAGCTTAACCACTATGCATCAACCCAGACCCACATGAATAAC

CTCTCATATCTTCAAGCTTACCAACAACCCCATCCCCACAAATTTTTATCCCCAAAGCCG

ATCCCCATGAAACAAATTGGTACACCACCAAAACCCACAAAACTTTATAGAGGAGTGAGG

CAAAGGCACTGGGGCAAATGGGTTGCGGAGATCCGTTTGCCTAAAAGTCGAACCCGACTC

TGGCTTGGCACATTCGACACGGCAGAGGAGGCAGCTTTGGCTTATGACAGAGCAGCTTAT

AAACTCAGAGGCGACTTTGCCAGACTTAACTTCCCAAATTTACTCCACCAAGGGTCCTAC

ATCGGCGATTACAAGCCTCTCCATTCCTCAGTGGATGCGAAACTTGAAGCTATCTGTGAA

AGCTTGGAGAACACTTCGCAGCAGAAACAAGGAGGGAAAACAAAGAGGCAAAGTAACTCG

ACGAAGAAGAAAGCCAACTCGGCAGTGGTGGCTCGGGAGGAGGAGCATGAGGTTGTTAAG

GCTGAGACAGAGTCTCCGGCATTGACAGAGAGTGTCGGATCGGGTGGATCTTCTCCTTTG

TCGGATCTAACGTTTCAGGATTTTGAGGAAGCACCGTTGGATTTTGAATCGGGAAATTTT

ATGTTGCAGACGTATCCTTCTTATGAGATTGATTGGGCCTCAATTGTATCTTAG

>EVM0037879

ATGGTGAAGAACGAGCTCAAGATCCAGTCAGAGACTTCCACACCAATGCAAGCATCATCA

GCATCTTCATCAGCATGCAAGAAGAGGAAATACAAGGGAGTTAGAATGAGAAGTTGGGGC

TCATGGGTTTCTGAGATAAGGGCACCAAATCAAAAGACAAGAATATGGTTAGGCTCTTAT

TCAACCCCTGAAGCTGCTGCTAGAGCCTATGATGCTGCGCTTTTATGTCTTAAGGGCTCT

TCAGCCAATCTCAACTTTCCTGTTGCTTCCTCACATTACATTCCTGACGCTGTGATGTCC

CCCAAGTCCATTCAGAGAGTGGCTGCAGCTGCTGCTAATACTTTCGTAGACAATCCCACC

ACCCCTCAAATATCGTCACCTCCTCTTCCTTCCTCCTCCTCCTCCTCCTCATCAATCCTA

TCATCCCCGTCAATGGTTTTCTCACCATCCTATCAGCTTGATGATTATATGTCACAGATG

GGATCGTTCAAGACCGACAATGAACCGATTTCAATGCTAGATTCTTGGTACAGTTTTGAT

GGATTACAATCTCCAAAGTATCTTGATCAAATGTTCAATGGGGCCTCATTCAATCCACCG

ATGATCGATGATTTCTACGAAAGCGATATTCGTCTATGGAGCTTTGCTGAATAG

>EVM0003769

ATGGATCTCCACAGTAGCAGACCGCTTCAATCAGACCCCGTCGTAGACGAGTTAATGGAA

GCACTCGAACCTTTTATGAAAAGTGCTTCCTTTTCAACTATCCCATCCCCTTCTCAAACC

CCTAACTATCCTTCTTCTCCTTCTCTCCCTTCTCTCCCTTCTACTTCTACTTCTTACGAT

CACATCTCTTCTTTCTCCACGCAATCACCTCTACACCAACAGCAACAGCCCTCTTTGTAC

CCAGACGTTTGCTGCTCCACATCGACTGCCACCCTATTTTCAACTGGGTTCTCGATTAAC

GACCCAATGGGTCTCCAGCAACCATCTAGTTCAATCGGGCTCAACCACTTCACCCCAACC

CATATCCAGCAGATCCAAAACCAATTCCACCAAAATAACAACCACTCTAACCTTCAAACT

TATCAACAACCGCAAACCCTAAATTTTTTATCCCCGAAGCCAGTCCCCATGAAGCAAATG

GGTATACCGTCAAAACCCACGAAGCTTTACAGAGGAGTGAGGCAAAGGCACTGGGGCAAG

TGGGTCGCTGAGATCCGTTTGCCCAAGAACAGAACCCGTCTATGGCTCGGCACCTTTGAC

ACAGGAGAAGAGGCGGCTTTGGCTTATGACAACGCAGCTTATAAACTAAGAGGCGAATTT

GCAAGGCTTAACTTCCCTCATCTACGCCACCAAGGGTCCCAAATTGGCGATTACAAGCCT

CTCCATTCCTCAGTTGATGCGAAACTCCAAGCTATTTGTAAGAGCCTGGAGAATTCTTCG

CAGCAGAAACAAGGAGGAAAAGTAGAGAAGCGAGGTAACTCGGCCAAGAAGGTAACCAAC

TCGGTGGTGGAGACTCAGGAGGAGGAGCCGGTGGTCGTTAAGTCTGAGACGGAGTCTCCG

GTGTTGACGGAGAGTGATGGGTCAGCTGGATCGTCTCCTTTGTCGGATCTGACGTTTCCG

GATTTTGAGGAAGCACCGTTGGATTTTGATTCGGGGAATTTTATGTTGCAGACGTATCCA

TCTTACGAGATTGATTGGGCTTCTATTCTATCTTAA

>EVM0030239

ATGATGTACGGGCAGAGCTTACCTGATCAGCCCGAGTATGCCTTTCTTGAATCCGTTCGC

TTGCATTTGCAAGACGAAACTGTTTCTACTGCTCCTACCACTACTTGCAGCAGCTTTAAC

CCAACAATTGCAGCGACGACGGCTGCTCCAGCTGCTGGCTGTGCCCCACCCCTTGTTTAT

TGCAGGAGCACAAGCTTCAGCAGCTTGTATCCTTGCTTAACGGAAAACTGGGGAGACTTG

CCACTCAAGGTCGACGATTCCGAGGATATGTTCCTTTATGCTGTCCTCCGCGACGCCGTC

ACCGATGGGTGGGTCCCATCACTCAAAACCAATCAGCTGGCGGAACCCAGTTTCCCACTA

GTGAAACTGGAACCCACAGAGAATTTGACTGTCTCGCCGCCATCCACGGCGGTTACAGTG

GCTGAGCCTGCGGTGTCTCCGTCCAAGGGGAAGCATTACCGGGGTGTGAGGCAGCGGCCT

TGGGGGAAGTTCGCAGCTGAGATTAGGGACCCAGCGAAGAACGGGGCCCGGGTTTGGCTC

GGGACGTTCGCTACGGCGGAGGATGCGGCGTTGGCTTACGACCGTGCTGCCTATAGAATG

CGCGGGTCGAGGGCGTTGCTGAATTTTCCGTTGAGAGTGAATTCCGGTGAGCCCGATCCG

GTTAGAGTGACTTCGAAGAGGTCTTCTCCGGAGCCATCATCGTCTGTGGACAGCGGGTCA

CCAAAGAGGAGGAAGAAGGTTGGTGGGACAGCGGGTGCTGCCACAGTGGTGGCTAAAGCT

GGGTTGGAAGTCGGGAATGGGCTTGGATGTCAAGTGGGTACACATGGCGAGCAACTGTTA

ATTACCTAA

>EVM0040670

ATGGAAGCTGATCAAACTCAGCAAGTATCTTCAGCCTTAGAGCTCATCAAACATCATTTG

CTTGGCGATCTTCTTTCACCTGTTGCTACCTCCTCCTCTTCTATTTATGAATTTTATACA

AATTCTGAAACTTCTACTTCCCTACAGCGTGAGACTACTGGCTGCTCCAGTTCTGACTTT

CCCACCGCACTCCCAGATTTTTTCGGTTCTCTCTTCGACTTCACTTCCGTTTTGAATCCC

GCTCAAGAGAATAACACCAGTATCTTTGAGTTTGAACCTAAAGCTGAGATCATTGATCTC

TGTACTCCAACGCCACTCAATTCTACCCCCCACATCAATTTCCTGTCAAGCAGCAGCAGC

GGAAGCAGCAGCGGCAGTAACTTCTTCGAATTTGAGCCCAAGCCTCAAAGTCATCAGAAT

TTCGAATTTGAATCGAAACCACAAATGGTTTCTCAACGAAGCAACAGTTTCTTCAATGAA

GAACCAAAGCCTCAAATTCAAGCAGCTCGGAAACGCTCTTTGAAAATATCGCTGCCTAAC

ATAAAACCCGAATGGATCCAGTTCTCTAACCCAAGCCCGAAAATCGTGCAGGATAATTCT

GGTGTTGCTGCGCAAGAGAAAAGGCATTACAGAGGAGTCCGACAAAGGCCATGGGGCAAG

TACGCAGCGGAGATCCGGGACCCGAACCGGGGAGGTTCACGTGTGTGGCTAGGAACCTTC

GGTACAGCTCTTGAAGCCGCTAGAGCCTATGATCGAGCTGCCTTCAAGCTGCGTGGGTCG

AAAGCGATTCTCAATTTCCCATTAGAAGCCGGGAAGTGCGTGGAGGAAAGTGAGAGGAAA

AGAAGTAGAGAGGTTGGCGTGGAAGAGAGAGAGGATGTGAAGAGGTTAATGACGTTGGTT

AAGAGGGAGGAGCCTGAAACTGAGAGGGATATCCCGTTGACGCCGTCAAGCTGGACGACT

GTCTGGGATGGTGGCGATACGAAGGATGTCTTTAATGTACCCTCGTTGTCTCCGTTATCT

CCTCACCCGCCATTGGGATTTCCACAGTTAATGGTTATTTGA

>EVM0030192

ATGGAAGCTGATCAAACTCAGCGAGTATCTTCAGCCTTAGAGTTCATCAAACATCATTTG

CTTGACGATCTTCTTTCACCTGTTGCTACCTCCTCGTCTTCTATTTATCAATTTTATACA

AATTCTGAAACTTCTACTTCCCTACAGCGTGAGACCACTGGCTGCTCCAGTTCTGACTTT

CCCACCGCACTCCCTGATTTTTTCGATTCTCTCTTCGACTTCACTTCCGTTTTGAATCCC

GCTCAAGAGAATAACACCAGTATCTTTGAGTTTGAACCTAAAGCTGAGATCATTGATCTC

TGTACGCCAACGCCACTCAATTCAACCCCCCACATCAATTTCCTGTCAAGCAGCAGCAGC

GGAAGCAGCAGCGGCAGTAACTTCTTCGAATTTGAGCCCAAGCCTCAAAGTCATCAGAAT

TTCGAATTTGAATCGAAACCACAAATGGTTTCTCAACGAAGCAACAGTTTCTTCAATGAA

GAACCAAAGCCTCAAATTCAAGCAGCTCGGAAACGCTCTTTGAAAATATCGCTGCCTAAC

ATAAAACCCGAATGGATCCAGTTCTCTAACCCAAGCCCGAAAATCGTGCAGGATAATCCT

GGTGTTGCTGCCCAAGAGACAAGGCATTACAGAGGAGTCCGACAAAGGCCATGGGGCAAG

TACGCAGCGGAGATCCGGGACCCGAACCGGAGAGGTTCACGTGTGTGGCTAGGAACCTTC

ACTACAGCTCTTGAAGCCGCTAGAGCCTATGATCGAGCTGCCTTCAAGCTGCGTGGGTCG

AAAGCGATTCTCAATTTCCCATTAGAAGCCGGGAAGTGCGTGGAGGAAGGTGAGAGGAAA

AGAAGTAGAGAGGTTGGCGTGGAAGAGAGAGAGGATGTGAAGAGGGTAATGACGGTGGTT

AAGAGGGAGGAGCCTGAAACTGAGAGGGATATCCCGTTGACGCCGTCAAGCTGGACGACT

GTTTGGGATGGTGGCGATACGAAGGATGTCTTCAGTGTACCGTCGTTGTCTCCGTTATCT

CCTCACCCGCCATTGGGATTTCCACAGTTAATGGTTATTTGA

>EVM0051058

ATGAAGAATTCCACTAAACAAGAGTCCCGGGACAGCTCCAAGCGAAATCAGGACCCGGAA

AGCAACTGTGTAAGCGACAAACAGGCTAAGAGTGGTGGAAATGCCGGAAGGCACCCGTCA

TACCGTGGAGTGAGAATGCGTCAATGGGGCAAATGGGTGTCCGAAATCCGGCAACCAAAG

AAGAAGTCAAGAATTTGGCTAGGAACTTTCTCAACAGCAGAAATGGCGGCTCGAGCACAC

GATGTAGCTGCCCTTACAATCAAAGGTCATTCAGCTCTGCTTAATTTTCCCGAACTCGCT

CATGAATTTCCACGCCCGGCTAGTTCTTCTCCCAAAGATATTCAAGCTGCCGCTGCCCTA

GCAGCTACCTTGAGCTGCAAGACAAGTCAAAAGGGCCGCGAAACTGAAGCTGGAGCCGAG

CGGATGCTTCCTCGCTCCCTGGATAGTACTTCGGCATCTACTGAGACGCAGGGGTATTCA

TTAAGCTCACCATTAAGAGATGATGACACATTTATCGACTTGCCAGATATTTTGCAAGAC

ACAAGCCATCAATTTGATGAGCTTTGCTACTTGTCACCATGGAAGCCGATTGAAACCGAG

ACTTCTGATGATGGATTTTGGCATCATGAGGAGCCTTCTTTACCGGAATACCACTCAAAG

TAG

>EVM0018852

ATGGATCCATCTTCATCGAAAAGCAAAAGGAAGCAACCACAACAAGTTCAGCAGGAACCA

GGTACTGGATTAAGGTTTCTTGGTGTTAGGAGAAGGCCATGGGGAAGATATGCAGCAGAG

ATAAGAGATCCTTCAACAAAAGAAAGGCATTGGCTAGGCACCTTTGACACTGCTGAGGAA

GCTGCCTTGGCCTACGACAGAGCCGCTCGCTCCATGCGCGGTCCTCGTGCTCGCACAAAC

TTTGTTTACTCCGACATGCCTGCTGGTTCATCCCTTACCTCCATTATCTCCCCTGAAGAC

CAACAATTTCTACAGCAGCAGCAGCAACTCCACCAAAATAGCGACCACAATGACAACCTG

TCTTCATTATTCTTCAATGCCCGGCCTCCCTCTCATGATCTTCAGCTGGACTCGACTCCC

ATTTTCAACCAGGATTTTAGCTCTCGATGCCATTTGCGAGACGGATTTTCTTCATTGACT

TCTGATGGAGATTTTTGGTGCTGCTCCAGCACCAGAACTTATAATCAACAGCTACAGCAT

GTACTTCCTCATGAGTTTCCTTCTGATTTATCTCTCGACTCGGGTTATAGTATGGGTCAG

TGTGGTTTGGCCGATTTATCATCTTCAAGGTTGATGGGCTTCGAGAACCAGACAGCAATG

ACTTCTGGATTTGACTCGGTGGGGGGTAGTGGTGGCTCTTCTTTCTGGTTTTATTCTGGC

GAATACGTTCACAGCCCACTTTTTAGCAGGATGCCACCAGTTTCAGATATGGTACCAGAT

GGTTTTGATTTGAGCTCCTCAGCTTATTTCTTCTAA

>EVM0023232

ATGTGTGGCGGTGCTATCATCTCAGATTTCATCGCTCCGACAGCCACCGCTCGAGCTTCT

AGGAGGTTGACCTCGGGTTTTGAGTGGTTTGAACGGAAGCCTTTCAACAGCAAGAAGCTC

TCCAAGCCAGTTGTTGCTGATCCTGAAGATGATTTTGAGGCTGATTTTCAAGAGTTCAAG

GATGAGTCTGATGTCGGAGAGGATTACGATGTCTTTGTTGATACCAAGCCTCTTTCTTTC

TCTGCTTCTGAACCTGCAAAAAAACGCGGGCTCTCTCCTGGTCCTGCTGCTGTTATATCT

GCTGGATTCAGTGGACTTGCTGAAAAATCAGCAAAGAGGAAGAGAAAGAACCAGTTTAGA

GGAATTAGGCAGCGTCCATGGGGAAAATGGGCTGCTGAGATTCGTGATCCCAGGAAAGGG

GTACGTGTCTGGTTGGGAACATTCAATACTGCAGAAGAAGCTGCAAGAGCATATGATGCC

GAGGCACGAAGAATTCGTGGCAAGAAAGCAAAGGTGAACTTCCCCAATGAAGCTCCATGT

GCTTCAGGAAAGCGTCCAATTAAGGAAAACTCACAGAAACGACTTACAAAGGCAAATTTA

AGCCAGAATTTCAGTTACTCGAGCAACCCAGAAACGGATTATAATACTATGGGCTTTGTG

GAAGAGAAACCACAATTGAACCAGTTTGGAATAATGAGTTCTTTCTCGGCTAGTGGAGAT

TCTGGGGTGATGCCCTTAACTTCTGACAATGCTGCTGTGTATTTCAATTCTGACAAGGGG

AGCAACTCATTTGATTCTGACATGGGGTGGGGAGAACAAGGCCCAAATACTCCTGAAATC

TTGTCTGTTCTTGCAGCAACTTCAGAAGTTGATGAATCTGTCTTTGTGGATGCTAATCCT

AAGAAGTTGAAACCATACTCTGAGAATGCAGTGCCTGTTGAAGAGAAGAATGGAAAATCT

CTGTCTGATGAGTTGCTGGCTTTTGACCTTCAGATGCCAGATCTTGTGGGTAGCTGGGAG

ACTTCTCTTGATAGCTTCCTTAATGGAGAGACAACTCAGGATGGCACAAACGCAATGGAC

TTGTGGAGCTTTGATGAATTCCCCTCTATGGTTGGGGGAGTTTATTGA

>EVM0028655

ATGTCATTATGGATTTTAATACTGTTTTTTTTTTTCAATTTGATTCTTAAGTTTTTATTT

TCCATGATTGACTTTTATGTTTTTTTTATTATACAGCCTCTCACATATTGGAGTTCTGAA

AATACTATGGATGGAGGTAGAACAAGTGAGGATGATCCAAAGCTTGAGAATTTCTTGGGT

TTTTACTCAAACTCACCTTCTAATGAGGCCAAAGTTTATTGTCAGCAAGAAGATTACCAG

TGTCATCAAAACCATGCTGATAGAGCAAATGATAATCTAGCACCGAGTTTCAACACCAAC

GGAGATGTAAAAAATGGGGAGAACAGTCTCACAAACCTTTCTTCTTTAATCCAAAGTTAC

CATTTCAATGACAATCCACGTACCTTAATCCCTAATCACTGTCTTCAGCACTATAATTTA

AACCCTAGCCATAGCCATAACCATGGAAGTGGCATGAACCAAGTGCCTTTCGAGAGTGCT

AGTTCAGTTTCTGGGTTCAAGTCTTGGCTTAGGCAGACGGCACCATTTTCTTCTAGTGGG

AAGTCTCCAAATGAAGCAAACAATAGCAACTTTCAATCATTATTACTCACAATGAGTCCT

AGTTCTCAGAATGGATTGGCTGCTATATCTCCATTGCAAGTGGTTGAGCCAGTTGTAAAA

CCACTAGCAAAAGAACCAGTTTCTCATAAATCTATCGACACATTTGGACAAATAACTTCA

CAATATAAAGGTGTAACAAGGCATAGATGGACTGGGAGATATGAGGCCCGTTTATGGGAC

AATAGCTTCAGGAAAGAAGGACAAACAAGGAAAGGAAGGCAAGGTGAGAGGCTATTTTCT

GTGGGTATGATAAGGAAGAAAAAGCAGCAATTAAGGGCTTATTATTTAGCTGCTTTGAAA

TATAGGGGTCCATCAACGCATATTAATTTCCCTTTGAACACTTACGAGAAGGAGCTTGAA

GAGATGAGGCAGATGACCAGGCAAGAATTTGTAGCCAATTTGAGAAGGAAAAGTAGTGGG

TTTTCAAGAGGAGCTTCTGTGTACAGAGAAGTGACAAGGCATCATCAACATGGAAGATGG

CAAGCTAGAATAGGAAGGGTTACAGGAAACAAGGATATAGTACCTTGGAACATTTATTAC

GATATAGGTACACAAGAAGAAGCTGCCGAGGCCTATGACGTTGCTGCAATTAAATTTAGG

GGAACCAGTGCTGTAACAAATTTTGGTACAAGGAGGTATGATGTGAAGAGAATTTGCTCA

AGCTCAACACTCATTGCGAGTGACCTAGCTAAACGTTCATCAAAAGATTGGGCTCCGGTG

GCCCTCGAGGATTACAATTCTTGTGCTTCATCAACATCTTGTCAGCCCCTCCTTCGCATT

TCAAGCAGTGAAGCCTCCGATGAATTGACTGGCATGATGTGGAGTGATCAAAATACAGGG

GAACATCAACAGCTACAAAGTGCTAACAACGATGTAACTTTGGTGTCTTCTAGTAGCAGG

AATTCCTCCAATGCTGCAAGTCCAAAACGTTCGGCTGGCTTATTTAGTGATTTTGTCGTC

CTTGGCAGCCAAAGCTATTCTCAGGGCTACTTTCCATTGCAGGGTGGTAAGTATGAAGAT

GGTAATAATGAAATAGATAATGGAAGCACTGACCGGAACATTGGTAGCCTTCAAGTCCCA

ATGTTTGAGTTGTGCAGTGGTTGA

>EVM0024365

ATGGCCACGACTTCTTCTAGCTCCAAAAGGCACCCAATGTATCACGGAATCCGGTGCCGC

AGTGGTAAATGGGTGTCTGAAATCCGAGAGCCACGCAAAGCTACTCGTATATGGCTTGGC

ACATTCCCAAAGCCGGAGATGGCAGCAGCAGCCTATGATGTTGCAGCCCTAGCTCTAAAA

GGTGATGGTGCAGTTCTTAACTTCCCAAGTTCTGTCGGGACTTATCCAGTGCCTGTTTCG

GCATCATCCAATGATATTCGTAATGCGGCTGCGGCAGCTGCGGCACTAAAAAAGGCTGAA

ATGAGCCGCAAGGAAGCTGGAGAAGAATTTGTTGATGAGGAGGCACTGTTTGATATGCCA

AATTTGCTGGTGGACATGGCAGGAGGAATGCTGCTTTCGCCACCAAGAATAACCTCGGCG

CCACCGTCTGATGATTCATATGGAAATTCTGATGGAGAAAGTCTGTGGAGCTATTTTTAA

>EVM0040623

ATGGCAGGATCAAGTTCCTTCTCAGCTGACAGGATAGAATCATCTTTGTTATCCGACAGT

AGCGATTCTCAACAAGATTCGCCCAGTTGGAACGAGGAGGTTTTACTAGCGACAAGTCGT

CCGAAAAAGCGTGCAGGGCGGAGAATATTCAAGGAAACTCGTCATCCAATTTATAGAGGT

GTCAGGAAGAGGAATGGCGATAAATGGGTCTGCGAATTGCGTGAACCAAATAAGAAGTCA

CGTATTTGGCTAGGCACGTATCCTACACCAGAAATGGCAGCCCGGGCACACGACGTAGCT

GCTCTGGCTTTCAGAGGAAAATCAGCTTGCCTTAATTTTGCGGACTCGGCATGGAGGTTG

CCTGCGCCAATATCAAACGAGGCTAAGGACATCAGGAGGGCAGCAAGCGAGGCAGCAGAG

CTGTGTAGGACTTCAGACTTTGGTGAAGTTTGTAGTTGTACTAACGATGGCATTAGAAAT

TTGCCGTCTGAAAATGCGGGGTATATAGACGAAGAAGCAGAGTTTAACATGCCAGGGTTG

CTTGCTAACATGGCCGAAGGCCTTCTCCTCTCTCCTCCGCATTATGCTGGGGATTCTATG

AATTGGAATGACTGCGAAATTGATGTTGATTGGTCGTTGTGGAGTATTTAG

>EVM0026062

ATGGAGTTCTCCGATCAACAACATCCTTTGAGGTCAAACTCAGTGCCCTTTGACAGGCAA

GAAACATCTTTCTCCGATAGCAGCTTGTCTCGAGGATTCGTCCATTCTGATGAAGAAGTC

TTACTAGCAAGAAGCTGTCCGAAAAAACGCGCTGGACGCAGAATATTCAGGGAGACTCGG

CACCCGGTTTTTAGAGGTGTTCGGAAGAGGAATGGTAACAAATGGTCACGGATATGGTTA

GGAACTTATCCTACACCAGAAATGGCCGCTCGAGCTCATGATGTTGCTGCATTGGCACTT

AGAGGCAAATCTGCCTGCCTTAACTTCGCTGATTCTGCTTGGAGGTTGCCGGTGCCAGTT

TCGAAGGATGCTAAGGATATTACAAGGGCGGCAAATGAGGCAGCAGAATTATTTAGGCCT

CGAGAGTTTGGTGGTCATCCGGCAAAACAGCAAAATAACAATGCAGTGCTAGAGGACTAC

CCACCTGAAATTTGCAGCGATGATTGCAAGACTTTCCAAGAAAATGACGTCTTCTTTGAG

GAAGCGGTTTTTGACATGCCAGGGTTGCTCGTGGACATGGCAGAAGGTCTTCTACTCTCT

CCTCCACGTTATGCAAGGAATGATAGCACCGACTGGGATCATATGGAAAATGGTTCTGAT

TTGTCATTATGGAGTTATCAGGCAAACTTCTTCTAG

>EVM0017041

ATGGCCAGACCACAACAGCGATATCGAGGGGTCCGACAAAGGCATTGGGGATCATGGGTG

TCTGAAATTCGTCACCCTTTATTGAAAACTAGAATCTGGCTGGGTACGTTTGAAACAGCA

GAGGATGCAGCAAGAGCTTACGATGAAGCTGCGAGGCTTATGTGTGGGCCTAGGGCTAGA

ACCAACTTCCCTTATAACCCAAATGCATCACAATCAGCTTCATCGAAACTTCTCTCTGCA

ACTTTGACAGCAAAGTTGCATAGGTGCTATATGGCCTCATTGCAATTGACAAAAAAAACA

TCACTTCATGGACAAAAACAACAGCAACAAAAGGCACCCACTTCACTTGCCACCACCCCC

ACCAACGGCATTGTCATAAAAAGCGAAGAACTTGATGCTTTATTGCCAGAGAAAAGTCCA

TTACAAGTTCAAGAGGCAGAGGCAAACTGGGTGTACAAGAAAGTTCAAGCTGATAACAAC

CAGCAATTCATAAAGCCTCTTGAAGATCATCACATTGAGCAAATGATTGAAGAGTTGCTT

GATTGTGGGTCCATTGAGTTTTTTTCTGGGGTAACAACTCAGTAA

>EVM0008598

ATGGTACAATCCAGGAAATTCAGAGGCGTCAGGCAACGCCAGTGGGGCTCCTGGGTGTCT

GAGATTCGCCACCCTTTACTGAAGAGGAGGGTATGGCTGGGGACATTTGAGACAGCTGAG

GCAGCGGCGAGAGCATACGATCAAGCAGCTATATTGGTGAATGGACAAAATGCCAAGACC

AATTTTCCTGCGAGCCATCTTGATCAGGACACTAAGCTTGACAAAGATGGCAGCTCTGCC

TTGCCTGCCAAGGAACTGGCTGAGCTTCTCTACTCGAAGCTAAGGAAATGTTGTGGAAAA

AACCCTTCTCCCTCGCTCACTTGTCTGAGGCTCGACAATGATAACTCTCATATTGGCGTG

TGGCAAAAGAAAGCTGGCTCCCGTTCAAGCTCAAACTGGGTCATGAGGGTCGAGCTTGGA

AATAGCAACACGAAGAGCACCCAGGTTATGGAGGAACTGCGACCTTCGTTGTCATCAAAG

TCATCGTCAACGGTTGAGATTGAACCTGAAAATGGTACGGATGAAGAGGAGAAAATAACC

ATGCAAATGATAGATGAGCTGCTTAATTGGAATTGA

>EVM0054855

ATGGAAGAAGCACTAAGAAGGCTCAATGGAATGCCGAACCATGTACCAGAATCCAACCCG

CCCGAAACCATAATCGCCGACCTCCAGAAGAAGTCAGCACCTACCACCACAAGCAAAAGG

TCACTGAAAGAAAGTGGTGCCAATAACGGAAACATGAGGTACCGTGGAGTCCGCAGGAGG

CCATGGGGTCGTTATGCAGCCGAGATAAGGGATCCTCAGTCTAAGGAACGGCGGTGGCTC

GGCACTTTTGACACTGCTGAGGAGGCTGCTTGTGCCTATGATTATGCTGCTCGAGTAATG

CGTGGTATCAAGGCACGTACTAATTTTGTGTATCCTGAGACTTATTCTGCAACAACTGAC

TCTCAAAATTTGCTCCCTCCAATTACTTTTTCAAAATCATCCCATCATCAGCCCTCTCGC

CAGTGTAATACAGTACCATCTGCAAATTGGTCTTCTCTTGGACACTCTCTCGTGGGTGAC

TTCTCAGCTGGTTGGTCTGCGCCACAGAGGAGTGCTAGTGGCAGTACCGCTAACACCTCT

TCTCTGAACATGCTTTTTCTTCGTGATTTCGTAAACTCCTCTTCTGGTTCGTCGTCTCAA

AACCATACTCAACCTTTATATGACCACTTTTCCGGCATCAATGGGTCCTCCTATTCTGCT

GCTAGCACTTACTCAGGTGGTTCTTTGGCGAATCCTTCAAGTAATTCCAACAGTACTGGC

TTATATAATTTCTCTGGAAATTTGGCAAACTCAACTGTCACAAGTCTACCCCGCATGGAG

TTTAATCAGAATTATACTCCTGTTGTCCCGAATAGGCCCAACTCCAAAACTGAGGATTTG

GGTTTCTTTCAGCAAGAGCCATCTGGTTCTGGTTTGTTGCAAGAAATTATTCAAGGGTTT

TTGCCTAAGCACTCCACTGAAACGATTGGCTTTTCAAAAAGCGCTGGAGAGTCAATGGTT

GCACCATCAGCTGAAATAATATCTGGTGGTCAGTCGCTAGATGGATCAAGGAGCAGGAAG

ATGCACTTAGTTGAAAACGGGCACCATGGTGCTTATCTTGACCATGAAGGAATAAGGCCA

TCAGCACAGGTTGAGGGCTGTTACCGTACTGGGGTTAGTTGTCATGACCAAGTACCGTAC

AATAACGAGATGTCACCAAATCTTCCGGTGGGTCAGGACTCCTTTCTGGAAGACATTTTT

CAGTACCCCGATCTCATGAGTGCCTTCGCAGCTAGGTTCAACAGTGCTTGA

>EVM0028978

ATGTGTGGCGGTGCTATTTTGGCCGACCTAATCCCTCGTAACCGTGGCCACCGTGTCGCG

GCTTCTGAGTTCTGGCCCAACTCATCCTTCAAGAAACCCGGCCCCTTCGACTCATATCCG

AGTCCTATCCGCAACCAGGAGTCATTCACTCTCAAACGATCCCAAGCCACTTCAGGGGAT

CATGAGCAAGTGGAGAAGCCGAATGCCAAGAGGCAGAGGAAGAACCTATACAGGGGTATA

AGGCAGCGACCTTGGGGTAAATGGGCAGCTGAGATTCGTGACCCAAGAAAAGGAGTCCGC

GTTTGGCTGGGTACATTTAACACTGCCGAGGAAGCAGCTCGAGCCTACGACAGAGAGGCT

CGCAAAATTCGCGGCAAGAAAGCCAAAGTCAATTTTCCTAACGAAGATGAGCATCGCTAC

ACGTCCCAAAACACGAAAACCAAGCCTTTCCTGCACCAAGCTCCCACTTGTCATTTCTCA

AAAGGTTATGATTCTGGGTACAGTTATGATGGATACCAGATCAAAACCTATCATTCAAAT

GGTTTAATCAACGAACCAATCGTTACTTCTGGGGAGGATGATTCTGGTTCAGGTTCAGAG

GAGGTGACAGGACTGCTGGGCTGTAATCAGAATGCGGAGAGCAATAATTATACGGGTCAA

GTGAAGGTGGAAGAGAAGAAACTGGAGGAGGAGGAGAAAGGGATGAATAAGGAGGTGGTG

GTGGTGGATTTGGAAACTAGGGAGGAAGAAAGCGAGGTGCAGAAGCTGACTGAGGAGTTA

ATGGCGTATGAGAACTTCATGAAGTTTTATCAGATTCCGTATCTTGATGGGCAATCAACG

GCACCAAATGGGACTACCCAGGAAAGCCTTGTGGATAGTCTTTGGAACTTTTTTGATGAT

TGTGTTGATGCTCCTGTCACTTCTGCTGCACCTCTGTAA

>EVM0007646

ATGGAGTATCATCAATTCCAAAAGCAGCCAAAAGGAGTCTCACACAGCAAATGCAAGGAG

AGATGCTTAAGCAATACCTCCAAGAACAAGTTTGTTGGCGTTAGACAAAGGCCTTCAGGA

AAATGGGTGGCAGAGATCAAAGACACTGCACAGTCACAGAAGATCAGAATGTGGCTTGGG

ACCTTTGAGACAGCTGAAGAAGCTGCGCGAGCTTATGATGAAGCTGCCTGCCTTCTTCGT

GGTTCGAATACCCGGACCAACTTTAGCACCCATGTTTCCTCCAACTCTCCTATTTCTATG

AAAATTAGAAACCTTCTCAGACACAAAAGGAGTTTGAAACAGAACTCTTCCGCAACTTCC

ACCACAAAATCTACCATTAAAACCAGCACTATAGCTAGCACCAAGAGTAGTTTCAATAGT

AGCATTAGTTGTTTTCTTCCTAATGATAGCAACATAGATAGTTTCCCATCCTCTATTGAT

AACCATAGTAGTTTTATTAATGGCATCAAACAAGAAAACAAAGTGTTCGATGATGCATAT

AGGCCAGACTTGAGTGGCTGCTGCATTGGAGGGCTTGAACCGGTTACATCTCAGTTCCAT

CCCTCGTGCTCACTCCCATCTGGGTTTGATCCTCAACTTCAATTTGTTCAAGAAGAGATG

CGGCTGCCGAAGAATGATGCTTTGTTGTCTGATACAGCATCTGTTCCAGAGTTAGCCGAA

TTCGAACGCATGACGGTGGAAAGGCAGATATCAGCATCATTATATGCCATGAATGGAGGG

AGTGAATACTTCGAGAATGCTAATGATTCAAGTGATGCTCTCTGGGATCTCCCTACACTC

TGCCAATTGTTCTGTCGTAGTTGA

>EVM0035153

ATGAATTCTAACAATTGGCTTTCATTTCCTCTTTCTCCTACTCATCCGACCTTGCCTGCC

CATCTACATGCATCTCACCCTCATCAATTCTCTCTAGGGTTAGTCAATGATAATATGGAG

AATCCATTTCAAACTCAAGAGTGGAGTCTTCTTAACACTCAAGGGAAAAATGAGGTGCCA

AAGGTTGCAGACTTTCTTGGTGTGAGCAAATCTGAGAATCAATCAGATCTCGTAGCCTTC

AATGAAATTCAAGCTAATAATTCTGAGTATCTCTTTTCAAGCAATAGTCTGTGTCCAGTC

CAAAATGCTGTGGTAGCAGCCAGTACTAACTATGAATTTCAAGAAAAATCTAGCAATTTG

CAGTCATTAACATTGTCTATGGGCAGTGCTAGTGGTAAGGGTTCTAAATGTGAAACCAGT

GGTGATAATAGTTCAAATTCTGTCGAAGCTGCTGCTCCAAGAAGGACGTTGGATACATTT

GGCCAAAGAACATCCATCTATCGTGGTGTAACAAGGCATCGATGGACAGGAAGGTATGAA

GCTCATTTATGGGATAATAGTTGCAGAAGAGAAGGTCAATCCAGGAAAGGAAGACAAGTC

TATTTAGGTGGCTATGACAAAGAAGACAAGGCTGCTCGGGCTTATGATCTTGCTGCACTT

AAGTACTGGGGAGCATCCACCACTACCAATTTTCCTATCAGCAACTACGAGAAAGAAGTA

GAGGACATGAAGCACATGACCAGACAAGAATTTGTGGCCTCCATTAGAAGGAAGAGTAGT

GGCTTCTCTAGGGGTGCATCCATGTATCGTGGAGTCACGAGGCATCACCAACATGGAAGA

TGGCAAGCAAGAATTGGCAGGGTTGCTGGAAACAAAGATCTCTACTTGGGAACTTTTAGT

ACTGAGGAGGAGGCTGCAGAAGCTTATGACATAGCAGCAATAAAGTTTAGAGGGCTTAAT

GCAGTGACTAACTTTGACATGAATCGATATGATGTGAAAAGCATTCTTGAGAGCAATAGT

TTGCCAATTGGAGGAGGGGCAGCCAAACGGCTAAAGGAGGCTCAAGCAATCGAATCGTCA

CAAAAACGAGAAGACATTGTTGCTCTTGGATCAAGTTATCCATATGGATCAAGTTCAAGC

TCTAGTAGACTACAAACTTACTCTCTGATGCAGAAGCCATTTGAGCAACCTCAACCTTTA

CTTACCCTACAAAATCAAGACATTTCCGATCAGTACACTCAAGATTCTTCATTTCATCAA

AATTACCTTCAAACACAGCTTCATTTGAACCAACTATCTACAGGGTCTAATTTCCTGCAC

ACTAACCAATCAAGCCAAAACCCTCAGTATCACAACAGCTATATCCAAAACAATCCAGCT

TTGCTTCATGGATTGTGGAACATGGGTCCATCTGTAATGGAGAATAATGGAAGTTCTAGT

GGGAGTTATAGCACTGGAGGTTATCTGGGAAATGGATTGGGAATGGCTACCAATTCGACA

GGGTCCATTGCAGTAGCTGAGGAGCTTCCACTTGTGAAGGTTGATTATGATATGCCTTCT

GGTGGCTATGCAAGTTGGTCTGGGGAATCAGTTCAGGGATCCAATCCAGGTGTTTTTACA

ATGTGGAATGAGTGA

>EVM0056670

ATGAAGAGGTCCTCGTCGTGCTCCTCCTCCTCTTCATCGTCTTCTTGTGTTGCCTCTCAA

AGCATTGAGAAACCGAAGGCCAAACGCGTTAGAAAGAATCAAAAGAGCAATCAAGGAAAA

TCCCAGAATAATGCTGCTGCTAATAATAATCATAACTCTGGCAAAAGAAGTTCCATTTAT

AGAGGAGTCACCAGACATAGATGGACAGGAAGGTTTGAAGCGCATCTTTGGGATAAGAGT

TCATGGAACAACATTCAAAACAAGAAGGGAAAGCAAGGTGCCTATGATAATGAGGAGGCA

GCTGCGCGCACCTATGATCTTGCTGCCCTAAAGTACTGGGGAACAGAGACAACCTTGAAT

TTTCCGATAGAAAAATACACAAAAGAGAGGGAAGAGATGGAAAGGGCGAGTAAAGAAGAG

TACTTGGCATCTCTGAGACGGCAAAGCAGTGGATTCTCGAGAGGAGTCTCCAAATACCGT

GGGGTGGCTAGGCATCATCATAATGGCCGATGGGAAGCTAGAATTGGACGAGTTTATGGG

AACAAGTATCTCTACCTTGGAACTTACAATACACAAGAAGAGGCAGCGGCGGCGTATGAT

ATGGCGGCGATAGAGTATAGAGGAGCAAATGCGGTGACCAATTTTGATGTCAGCAATTAC

ATGGAACGATTGAGGAAGAAAGGCATCCCCATAGACCGAATCCTCCAAGAACAACAACTT

CGTAACAACTTGATTGATTCCAGAGTAGAAGTAGAGGCGGAGGTTGAACAGCCGCCGCCG

CAACAACAAGAGGAAAAAGAGCAAAAAGTAGTCCCGTCGTCGCAACTTCAATGCTCACAA

ATAAATTCAAGCTTGGACGGCACGTCTCCTATGGTAATTATGGACACCATCGAAGAGCAC

GAGCTAGCATGGAGCTTTTGCATGGATTTAGGACTGAACCTCACGATGCCTGATCTCCCT

CTCGAAAATTCTTGCGAGTTGCCAGACCTGTTCGATCACACAGGCTTTGAAGACAACATC

GACTTGATATTCGACGCGTGTTGCTATGGAAATGAGGCCGATCAAGCTGGCTACATATTA

GAGGATAATAGCACAGGAGGAGTTGAAAAGGTTGGTGTTACAAGGAGTGTTGACGAGGAA

AGTGAAAGTCGGAAGGACAGGTTGTTGTCTGATTCGGTCTCAAACTCTCCAACTAGTTCA

ACCACCACCTCTGTTTCTTGTAACTATTATGTTTGA

>EVM0049616

ATGCTTCCAAATTCCGCAGTGCAGCCTGTTTACTTGAGTAGCAAGCAGCCACTCTCTGTC

TCTCTCCGAATACAAAAATATAAATGGCATGCAGTTTATGGTACACCACCGCCCTCATCC

ACCATTCACATACATGACATTCAAATCATCACACAGGCGAGGGCTCCTCTATACTTCGAC

AGATCAACAGCCAAATATCGAGGCCCTCTCAACCTTAATGAAGTCTTAAAAGCCATTCAC

TTTTTAATGGACAAGTCATCCCACCCACCCCAAGAACCCGCCGCCGCCGCCGCCACAATA

TCATCCAATGACAAAAGCATTGATAAAAACACCACCGCAACCACCCCCCACTACTACCAC

AACAATGGCAGTAGCAGGAAGTGCAAAGGCAAAGGAGGACCAGATAACGGTAAATTTAGA

TACAGAGGAGTTAGGCAAAGAAGCTGGGGCAAATGGGTAGCAGAGATCCGTGAGCCAAGA

AAACGAACCCGTAGATGGCTTGGCACTTTTTCCACAGCAGAGGACGCAGCACGGGCCTAC

GATCGAGCAGCCATCATCCTTTATGGTTCAAGGGCTCAACTCAATCTCCAACACTCAGGT

TCCTCTTCTTCTGCTCAGTCCGGATCAACTTCTCTCAACTCTGCCTCTTCCTCGAGCCAG

ACACTTCGCCCTTTGCTCCCTCGATCCCCTGGGTCTGGTTTCACTTTCTCTCTCTCAAAT

TCAATGGTTTCGGCAGCTTCGTCAGGACTTACGCCGCCTCGGGTTAGTTATCATTCGAAT

AATGTTGCCGGGTCGGCTATACCATGTCCAAGTACTAATGACGTGCTAATTCAAAGTCAC

CAGCAAGTTATGTTACAACGCTATCTTAATCAATATGGGGCTAATACAAGCAACCCCAAT

AATATATTTGATAGTTCTAGTGTAGCCACATCAACTACACCCTCCTATCAAAATCATAGT

TACCGTCAGCCGCAGCATCATGCGTGCGATGATGCTAGTTCGTTGTTGAGTTCTGTCTGT

TCGAGTTTCTTTTTGTCTGGAGGGAGTTCTCAAGTACCTATTGCTGCACCAGAGGGTTAT

CTTCAGGATCCGGTAAGGAATGTTGGGGCTGGATCTCCATCTGTGTGGCATGACGATGAG

TACCCACCGCCTAGTATTTGGGACGATGAGGATCCTTTCTTGTTTGATTTTTAA

>EVM0019343

ATGGAGGAAGCGCTTAGAAGGCTCGACGGAATACCGTCGTTATGCAGCCATACACTTGAA

ACCAGCCTACACGAAACCACCATCGCGGATCTCCAGAAGAAATCTGCACTTACCAACACG

GCTAGCACCACAACAAACAAGAGGTCACTGAAAGAAAGTGGTGGCACTGGTGGGAACATG

AGGTACCGTGGCGTCCGCAGGAGGCCATGGGGTCGTTATGCAGCAGAGATAAGGGATCCT

CAGTCTAAGGAACGGCGTTGGCTTGGCACTTTTGACACTGCTGAGGAGGCTGCTTGTGCC

TATGATTATGCAGCTCTATCAATGCGTGGTCTCAAGGCTCGGACAAATTTCGTGTACCCC

GATGCTCATTCTGCAACAGCTGAGCCTGCTCGTAATTTGCTCCCTCCATTTACTAACTCA

AAATCATCCTACCTGCCCTCTCGCAACTTTAATATATCCTCTTCAAACTCTTCTCTTGGT

CACCTTCATGTTGGTGACTTCTCTGCTGGTGGATCTGCACCGCAGAGGAGTACTAGTGAC

AGTATCGCTAGTACTTCTCTGAACAATATGCCTTTTCTCCGTGATTTCATTCACTCCTCT

TCTGGTTCGTCATCTCATAATCATCCCCAACCTTTTTCTGATCACTATCCATGCATCGAA

GGGTCCTCCAATTCTGATCCTATCAATTTCTCGGTTGGTTCTTTGGTGAATCCTTCTAGT

AACACTATTTCTGGCTCGTCTAATATCTCTTTGGCAGACTCAACTGTCACTACTCGATCG

CACATGGAGTTTCATCAGAATTACAGTACTCTTGGAGACCCGAAAAGTGCTGACGCTAAA

GCTGAAGAATTGGAATTCTTTCCACGGGAGCCATCCGATTCTGGTTTGTTGCAAGAGATT

ATTCAAGGGTTTTTCCCTAAACACTCCTCTGACAAGATTGCGTCTTCAGAAAGCTCTGGA

GAGTCAATAGCTGCACGGTCTGAAATAAAATCTCTTAATCAGTCACTAGATGGGTCGAGA

AGGGGCATGAAGAACTACTTTGTTAAAAATGAGCGCCATGATCCTTATCTTGGTCACCAA

GGAATAAGGTCGGGGCAGCTGATTGAGGATTGTTACAGTGGGGTTACTTCTCGTGAACTT

CCGCACTGTAATGAGATGGCATTGATCAATCACCAGATGGGTCCGGACTCCATGCTAGAT

GACATTTTTCAGTACCAAGATCGTATGAGTGCCTTTGCTGCTAGGTTCCAGAATGTTTGA

>EVM0050425

ATGCCGCTCAAGTCTGATGGGTCACTTTGCATGATGGATTCTCACACCAGGACACGGCCT

CAAGCAACAATGGTTACAGCTTCAACACCAAAACTTGAGGACTTCTTTGTTGGTGCAAAT

ATAGGGACCCATCACTATGAAGGCAATGATAGAGAAGCTATGGCTCTTAACCTAGACTGT

AGTCCTCCTATGTATTATCACCACTACACAGACCATGAGCCGAACAACCAAATTTGTCCA

AATCATCTCGAACAAAACCCCAGACAGCAGCAGCATCACCACATTCAAGTTCAGCAGTAC

CCATATTACTCTAACTTTAGGAACAATGAGATGTTAATAGGAGGAGAGGATGCCAAACAA

ATGACTCAGGCTGAAGATTGCAATCTTCTGCTTCCAAATATGGGAGACGATGGGATAACT

GGCATGAAGAATTGGGTTTCGACAAACTATCAAAGCAACCATGCCATGGAACAGAAGGTG

CTTAGATGTATGGTTGAAAATGGAGGTGAATCTGGGCCTAATATTAGCGCAATGACATAC

GGGGATTTGCAGTGTTTGAGCTTGTCAATGAGTCCTGGCTCCCAGTCGAGCTGTGTTTCT

GGTACTTCTCATCAAATCTCACCTTCGGTGATTGACTGTGCAGCCATGGAAACCAAGAAA

AGAGGAACTGAAAAGGCGGATCAGAAGCAAATTGTTCATAGGAAGTCCATTGATACATTT

GGGCAAAGAACTTCTCAATATAGAGGTGTTACAAGGCATAGATGGACAGGAAGATATGAA

GCCCATCTATGGGACAACAGTTGCAAGAAAGAAGGTCAAAGCAGGAAAGGAAGGCAAGTT

TATCTGGGGGGTTATGACATGGAAGAAAAAGCTGCGAGAGCTTATGATCTAGCTGCACTC

AAGTATTGGGGACCTTCTACTCATATCAATTCCCCTTTGGAGAATTATCAAAAAGAAATT

GAAGAAATGAAGAGCATGACCAGACAGGAATATGTTGCTCATTTGAGAAGAAAAAGTAGT

GGGTTCTCTAGGGGAGCTTCAATGTACCGAGGAGTAACAAGACATCATCAACATGGACGA

TGGCAAGCTCGGATTGGAAGGGTTGCCGGAAACAAGGACCTTTATCTAGGAACATTCAGC

ACCCAAGAGGAAGCAGCTGAAGCCTATGATATAGCCGCTATAAAATTTCGAGGAGTTAGT

GCTGTGACCAACTTTGACATAACAAGGTATGATGTGGAACGAATCATGGCTAGTAATACG

CTTCTTGCTGGGGAATTAGCCAGGCGAAACAAAGACATTGGACCTTGTAATGATGCTCCT

ACCAGTCAAAATCCTTCTACTCATCACAGCAATGGGGAATCCATGCCCTCACAGAAGAGT

GAAAACGAGGCGGATTGGAAAATGTTCTATCAGTCCTCCCAGCAACAGGATCACAAAGGG

TCAAATGTTGTGGATAATTACAGAACTCAGGCATTATCACTGTCCCCTGAAAATGTAATT

GCAAATGATAGCATGAGCTCAATTCACCAGCAAAAGGTAGAAGACTCTGCCAAGATTGGA

ACTCACATGTCAAATGCATCGTCGCTGGTGACTAGTTCAAGTAGCTCTAGAGAAGGAAGC

CCTGATAGAGCAAACCTACCAATGCTCTTTGGAATGCCTCCCTCGGCAGCATCCAAGTTG

TTCACTAGTCCAAATTGTGATGTGAATTCTTGGATCCCAACAACAGCAGCAGCAGCCGCC

CAATTCAGGCCTACTGCAGTCTCGTTGCCTCAAATGCCAATGTTTGCTGCTTGGACAGAT

GCCTAG

>EVM0012824

ATGGCACCAGCTACTAACTGGCTTTCTTTCTCTCTCTCTCCAATGGAAATGTTGAGGTCA

TCTGAATCCCAGTTGATGTCTTACGAGAGCTCCTCTACTGCCTCTCCTCACTACTTGTTC

GATAACTTCTATGCTGATGGCTGGGCAAACCCCAAAGAATCACAAGTCACGACCACAACA

ATGGCGGAGAGCTCTAATATTCTGACAAGCTTTATAGGCGCAGAAACTCAACACCAGCAA

GTCCCAAAACTGGAGGATTTTTTCGGAGACTCTTCATCATCTATTAATGTTCGATACTCA

GATAGCCAAAGAGAGACCCAAGACTCGTCCTTGACCCACATCTATGACCATCAAGGCTCT

GCAGCGGCTTACTTCAACGAGCAACAAGATCTTAAAGCTATTGCTGGGTTCCAAGCCTTT

TCGACTAACTCAAGCTCAGAGGTTGATGACTCGGCTTCAATAGCTCCCACGCGGCTGGGT

GGTATTGAGTCGACTGGGAACGAGTTGGGGTTCTCTAATAAAGCGGCCTTATCACTTGGG

GTGAATAATGATGCAAGCAATAATCAGGGTGGTTCTACAGAGAAGCTTGCTATTGTTTCT

GCCAATAACGATTGCTCTAATAAAAAGATTGCCGATACCTTTGGCCAGCGTACTTCTATT

TACAGAGGAGTTACGAGGCATCGATGGACAGGCAGATATGAAGCGCATCTATGGGATAAC

AGCTGCAGGAGAGAGGGTCAGGCCAGAAAAGGGCGTCAAGTGTACTTAGGTGGGTATGAT

AAGGAAGAAAAGGCAGCTAGAGCCTACGATTTGGCAGCTCTAAAATACTGGGGTCCAGCT

GCTACCACCAACTTCCCTGTCTCGAACTATTCTACAGAATTGGAGGGCATGAAGTATGTG

TCCAAGCAAGAATTCATTGCCTCACTGAGGAGGCGAAGTAGCGGTTTTTCACGGGGAGCC

TCTATTTACAGGGGTGTCACAAGGCATCATCAACAGGGTCGTTGGCAAGCGAGGATTGGC

CGAGTAGCTGGAAACAAAGATCTATACCTTGGAACTTTTGCCACAGAAGAGGAAGCAGCT

GAGGCTTATGATATAGCTGCAATAAAGTTTAGGGGTCTGAATGCAGTGACCAATTTTGAG

ATGAACCGATATGATGTTGAAACTATCATGAAGAGTGCTCTTCCAGTTGGTGGAGCAGCA

AAGCGCTTAAAAACATCACTAAACGCCGAGCAGAAACCAAATGTGAACGACAACCAGCAG

CCTCCCCATTCGAGGTGCAGCAGCTACAGCACCACCACCAACATCAGCTTTGCTGCTATC

CAGCCAGGTTCTACTGCGGTCCCTTGTAGTGTTCCATTTGATGCTTACACCGCACTTTAT

CATCACAATCTTGTCCACCACCTCCAAACCACTAACAGCTTCGCAGCAACGGACTCCTCC

GGCTCCTCCTCATCCATGGCAACTGCAATGACTACTTCGCCACAAACAACGGAGTATTTC

GCATGGCCTCATCAAACTTACTGA

>EVM0040310

ATGGGAAAAACATCAAGGCAAACCCTAGAGGATTCTGCAAACACCAGCACAAATCCGACG

AACAAGGTGAAGCGGACGCGAAAAAGTGTGCCGCGAGACTCTCCACCTCGACGTAGCTCC

ATCTACAGAGGCGTTACGAGGCATCGATGGACTGGGCGTTATGAGGCTCATTTGTGGGAT

AAAAACAGCTGGAATGAATCGCAGAACAAGAAAGGAAGACAAGTGTATCTAGGGGCCTAT

GATGATGAAGAAGCGGCTGCACATGCCTATGACTTGGCAGCATTAAAGTACTGGGGACAG

GACACTATCCTCAATTTCCCGTGGTCAACTTACGAAGAAGAGCTCAAGGAGATGGAGGGT

CAGTCAAAGGAAGAATATATTGGATCGTTGAGGAGAAAAAGTAGTGGATTTTCACGCGGG

GTGTCCAAATACAGAGGCGTGGCCAGACACCATCATAATGGAAGATGGGAAGCTCGGATC

GGCAGAGTTTTCGGCAACAAATACCTCTACCTTGGAACATATGCCACTCAAGAAGAAGCA

GCAACAGCATATGATATGGCAGCTATAGAGTACCGTGGAATTAATGCTGTTACAAACTTT

GACCTGAGCCGCTACATCAAATTGCTACGTCCCAATATTAATCAAAATAATCCTCAACAA

AACCCTAATGGCGATGCTAATCGGATTCAAGACCTTGACCGGAAGACTGGAATGGATTTC

ATGTCCCGCGAACCAAGGTCGAGTGTTAATGAGACGGAAGTGACCGACCCGACCCGGCCT

GTAGGCGGCGCGGCATCATCGGCGTTAGGGCTCTTGCTGCAATCCTCCAAGTTCAAGGAA

ATATTGGAGAGAACATCGGCTGCCAATCCCCCCTTGACGCCACCAGAATCGGATCGTGAT

CCGCCACGCCGGAGCTTCCCGGATGACATCCAAACTTTCTTCGATTGCCAAGACTCCAGT

AGCTACACCGATGGTGATGATATTATTTTCGGAGAGCTAAATTCATTTGCTTCATCGATT

TTCCACTACGAATTGGATGGACAGGAAATGGTATCTGGCACAAATAAGTCCATCCTTACA

ATATAA

>EVM0054250

ATGACTAGACCGCAGCAGCGATACCGCGGCGTCCGTCAAAGGCACTGGGGTTCTTGGGTT

TCCGAAATTCGCCATCCTCTCCTGAAGACCAGAATATGGCTGGGCACATTCGAAACGGCG

GAGGACGCAGCAAGAGCATACGATGAGGCAGCAAGGCTAATGTGCGGGCCGAAACCACGT

ACAAATTTCCCTTACAATCCCAATGAGCCACAATCATCTTCATCGAAGCTTCTCTCAGCT

ACCTTGGCAGCCAAGCTACATAAATGCCACATGGCATCTCTACAAGCAACCAAGAAAAAC

GAAACAGGACAGTCATATGAATCACAGTGCAAGAAGACATTTACCTCTAGCCATGGCATT

GCCGGAAAAACTGTCGGAACCTGCTCAAAATGGCAGGAGGGAAACTGGGTTGGTGAGGAC

AGTCAAGTAGGGAACGGTGATCGTGAGCAGCATTTCAAGTCACTGGAAGCTCATCATATA

GAGCAGATGATAGAGGAGTTGCTAGATTGTGGGTCCATGGAGCTCTGCTCTGTAGGTTCA

ACATGA

>EVM0013578

ATGTCTCTCACACCAGTCATGGGTGCTACCTACAGTAACGTCCCGAGTTCGGTGCAATCA

ACCTCATTTGCTCTCGACCAAACCGTCGTCAAAGACGAACCTGATCACTCTCAACCAGTG

CAAGATCAAGAAAATACGAGGAGAAGGCATTACAGAGGAGTGAGACAAAGACCTTGGGGT

AAATGGGCAGCCGAGATACGCGACCCTAAAAAGGCAGCTCGAGTCTGGCTCGGTACTTTT

GACACTGCTGAGGATGCAGCTGCTGCATATGACAAAGCAGCACTCAAGTTCAAAGGCACC

AAGGCTAAGCTTAATTTTCCTGAAAGAGTTCAAGGTAGAACCGAGTTCGGCTACTTCATG

GGTTCTGGGGATTCAAGCAATGTCTCGACTGAACAAAATCCGAGCCCAGCTGCTCCCCCT

CCTCCCCCTCCATCATTTGCACCAGACACTTACCCAGACTTGGTTCAATATGCACAGATC

CTTTCTAGCAATGATGCCAATTTCCCGTACTACACGTCAAACCTCTTTAATCAGCAAACT

TATCCCCCCCATTTTTCACAAAGCTTTTTAGCACAGCAACGACTAGATCAACAACAACAA

CAAGATCTAATGAGATTCTCGTCAGGGTTCGATGAATTCTCTAGCTCATATCAACAGGAA

CATGGGAAGGACTCCAGTAATCCTAGTGAGTAG

>EVM0039609

ATGGATGGAAGCTGCATAGATGAAAGCACGATTAGTATCACTGATAACTCAATATCCATT

ACACCAACTAGCCTCTCTCCTTTCCCACCACCTGCTACCACTACAAAATCACCATCTGAG

TCACTTTGCCGTGTTGGGAGCGGCAATAGTGTTATTCTTGATTTAGAATCAGGTGTCGAA

GCTGAATCAAGAAAACTCCCTTCTTCCAAATACAAAGGTGTTGTCCCCCAACCGAATGGT

CGCTGGGGCGCACAGATTTACGAGAAGCACCAGCGTGTGTGGCTCGGGACATTCAATGAA

GAAAATGAAGCGGCAAGAGCCTATGATATTGCTGCTCAGAGATTCCGTGGAAGGGATTCT

GTGACCAACTTCAAGCAAGTTGATGAGACCGAAGATGATGAAATAGAGGCTGCTTTCCTG

AACGCTCATTCAAAAGCTGAAATCGTTGACATGTTGAGGAAACACACGTACAGCGATGAG

CTAGAGCAAAGCAAGAGGAATCACAAGAGTAACAAGGGGGCAAATGGGAAGCAATACAAT

AACTATGGGGATAATAGTTATGATCATGGCTGTGTTCGGGTGTTGAGAGCGCGTGAACAG

CTTTTTGAGAAAGCTGTGACCCCAAGTGATGTTGGGAAATTGAATCGGCTCGTGATACCA

AAACAACATGCGGAAAAGCATTTTCCTTTGCAAAGCACATCAAACAATAGCACTAAAGGT

GTATTGCTTAACTTGGAAGATGTGAGCGGCAAAGTTTGGAGGTTTCGTTATTCTTATTGG

AATAGTAGCCAAAGTTATGTTTTGACAAAAGGGTGGAGCCGATTTGTCAAGGAAAAGAAC

TTGAAAGCTGGTGACACTGTTTGCTTTCAAAGATCAACTGGACCTGACAAACAGCTTTAC

ATTGATTGGAAACCAAGAGGCGGGTCCAGCCTGGTCCAGCCGGTTCAGATGGTTAGGTTA

TTCGGGGTTAACATTTTTAATGTACCTGGGATGGAAAATGGGTGTAATGGGAAGAGATCG

ATGAGGGATTTGGAGCTTTTGTCAATAGACCATCAGTATAGTAAGAAGCAAAGGATAGTC

CGAGCTTTGTAA

>EVM0001548

ATGAGTGGCAGCAGCAGCAGCAGCAGCAGAGCAGATCAAGAAAGTGGAGCAAGTGCAGCT

CAGAAGAAATTCAAGGGAGTGAGGAGGCGAAAATGGGGCAAGTGGGTGTCTGAAATTAGG

GTTCCGGGCAAGCAAGAACGGCTTTGGCTAGGCTCTTACTCCACACCAGAGGCAGCTGCG

GTGGCTCATGACATAGCCTCGTATTGCTTACGAGGACCCTCTTCTCTAGAGAGTCTGAAT

CTCCCTCTCCTGTTGCCTGCAAGTGTAAGGGAAGACATGTCACCTAAGTCCGTACAGAGA

GCTGCTTCGGATGCTGGCATGGCAATTGATGCTCAAATCATACGAAACAGGGTGCCGGAA

AATGAGGTCAAGTTTGGGACTGAAAGTGGTACAGTTAATCATGGACTAGAGACAGGATTA

TGGGAACCTGCAGGTGGTGCTCATGGTGATAATTGGCATGGAAACGATATTAGAATGAGA

GAAGGGGACATCTCCATTGAAGATTATCTCTAG

>EVM0043548

ATGGAGGTTCCATCAGGTACTGATCACCATCAACAAACACAGTCTGCCTCTGCTTCTGTT

CCTGCTGCGATCCCAACAAACATTTTCCACTCCCAGTTACCACACCTTAACAGCGGTATC

TACTATGGTGTTGACGGTGAAAATGGTGGTTTTTACTCTCCTTTGCATTTTATGCCGCTC

AAGTCTGATGGGTCACTTTGTATGATGGATTCTCACACCAGGACACGGCCTCAAGCAACA

ATGGTGACAACTTCAACACCAAAACTTGAGGACTTCTTTGTTGGGGCAAATATGGGGACC

CATCACTATGAAAGCAATGATAGAGAAGCTATGGCTCTTAACCTAGACAGTAGTCCTCCT

ATGTATTATCACCACGACACAGACCATGCGCCGAGCAACCAATTTTGTCCAAACCATCTC

GACCAAAACCCCAGACAGCAGCAGCATCACAACATTCAAGTTCAACAGTACCCGTATTAC

TCTAACTTTAGGAACGATGAGATGTTAATAGGAGAGGATGCCAAGCAAATGACTCAGGTT

TCAGATGGCAATCTTTTGCTTCCAAATATGGGAGACGATGGGATAACTGGCATGAAGAAT

TGGGTTTCGACAAACTATCAAAGCAACCATGCCATGGAACAGAAGATGCTTAGATGTATG

GTTGAAAATGGAGGTGAATCTGGGCCTAATATCAGCGCAATGACATACGGGGATTTGCAG

TGTTTGAGCTTGTCAATGAGTTCTGGCTCCCAGTCAAGCTGTGTCTCTGGTTCTTCTCAT

CAAATCTTACCTTCGGTGGCTGACTGTGCAGCCATGGAAACCAAGAAAAGAGGAACTGAG

AAGGTGGATCAGAAGCAAATTGTTCATAGGAAGTCCATTGATACATTTGGGCAAAGAACC

TCTCAATATAGAGGTGTTACAAGACATCATCAACATGGAAGATGGCAAGCTCGGATCGGA

AGGGTTGCCGGAAACAAGGACCTTTATCTAGGAACATTCAGAGCAGCTGAAGCCTATGAT

ATAGCTGCTATAAAATTTCGAGGAGTTAGTGCTGTGACCAACTTCGACATAACAAGGTAC

GATGTGGAAACGAATCATGGCTAG

>EVM0002934

ATGGTTAGCTTACGGAGGCGTAAACTCTTGGGACTTTGTGCTGGGAGAAGTTCTTTCCTG

ACTCCACTTCCTCGATTTTTTGACAACGGAACTGCTCCTGTAGGTTCTGCTCAGAATGGT

AGGTCTGTCAGTGTGCATCCTCTGCCATCAGATGATGCCAACCTGCCAGAGGAGAAAACC

ACTGCAAAAGTAGGAGCTGGCTCTTCAAATGTTTCGGCTTCTAGCTCGTCAAAAGAGCAG

CGATCTCAACCGTATCCAGAGCAACCAGTTAAACGCAGAAAGAGACATAGGAGGAAACAT

GTTCAGAACCAGGAACCATGCGTGATGAGAGGCGTCTATTTTAAAAATATGAAATGGCAG

GCAGCAATTAAAGTTGACAAGAAGCAGATTCACCTAGGGACTGTTGGTTCTCAAGATGAG

GCTGCTCATTTGTATGACAGGGCTGCTTTCATGTGTGGGAGGGAGCCCAATTTTGAGCTC

TCAGAGGAGGAGAAGCAAGAACTTAGGAAATTCAAGTGGGATGATTTTTTGGCAATCACT

CGCAGTGCAATTAACAATAAAAAACACAAGAGAAGGATTGGGGTAGGTTTGCAGAAGAGA

TCAGAGACCACATTGCAGGATGGTGACTGGGACGGCAAGGAACGACTCAATGGCTTTTCA

GCTTCAGAAGATGTGGAACCAGACTCATCAGCCTCTTGA

>EVM0054778

ATGCAATATTATCAACAAAGCAGCACCTCAAGTGCTGACAGCAGCAGTAGCAGTGGACGT

AGCAGATCTGCTTCTATTGCTGCTACTGCTGGGGCTCTTGCACCTAAGGTTTCAGGCCAC

CATCATGCTTTCCGGGGAGTTCGGCGTAGGAGTAGTGGAAAATGGGTGTCTGAGATTAGA

GAGCCTAGGAAGCCTAACAGAATCTGGTTAGGCACATTTCCTACCCCTGAAATGGCTGCT

GTTGCTTATGATGTGGCGGCTCTTGCACTTAAAGGCCAAGATGCAGAGCTTAATTTCCCG

GACGCAGCTGCTTCTCTGCCAGTTCCTGCTTCCACATCGCCGCGTGATATTCAGGCTGCC

GCAGCCTCTGCTGCTGCTGCTATAGGTGCTGCAAAGGATGCTTTAGGGATTCCAAGTATG

GGGGATACTAATCAAATGGAACAGGACATTAGGCCAATGGTGAATGATCAGTTTGTTGAT

GAGGATTTGATGTTTGATATGCCGAATGTTCTTGTGAACATGGCAGAAGGGATGCTTCTT

AGCCCTCCTCGCTTGGACATTGCAGGTGATGATGCTACAGCCTATGACTACAGCACTGGA

GACCAGAACCTTTGGAAATTCCCTTGA

>EVM0013126

ATGTCAGCAGCTATAGATATCTACAGCGCACAGGTACCAGCCTTTTCAGATCCTTGTAGA

GAAGAACTCATGAAAGCACTTGAACCTTTTATGAAAAGTGCTTCGCCATCACCAACTTCT

ACTTCTTATTCTTCACCATCTCCTTCAACTTCTTCTCCTTCTTTCTCTTCTTACCCTCCT

TGCTTTTACAGTAATAACTCTCCTATCTCTTCATATCCCAACTTGGACCTTAGCTTTTGC

TCGCCAACGAGCACCCAGATGTTTTCTAATGGGCTCTTGGATTATAGCCAAATGGGTTAT

GAGCAAACAGGTCCAATTAGGCTGAACCACCTTACCCCTTCACAAATCCTCCAAATCCAA

GCCAAAATCCACCTCCAACAACAGCAGCAGCAGCAGAAAATGGAAAATGTTGCTGCCACA

CCACAGTTTGTCCATAACCAAAGGGCTTGCAACCTCTTAGCTCCAAAAGCTGTCCCTATG

AAACAATCTGCTGCTTCGTCTCCTCATAAGCCAACAAAGCTTTATAGAGGAGTCAGGCAG

AGACATTGGGGAAAATGGGTTGCTGAGATTAGACTTCCAAAGAACAGAACTAGACTCTGG

CTTGGCACTTTTGACACAGCTGAAGAGGCAGCTTTGGCTTACGACAAGGCTGCTTATAAG

TTGAGAGGAGAATTTGCTAGGCTTAACTTTCCACATCTTCGCCACCAGGGAGCTCACGTG

TCCGGTGAATTTGGCGACTACAAGCCTCTCCATTCCTCTGTTGATGCCAAGTTACAAGCA

ATTTGTCAAAGCCTAGGTTTGCAAAAACAGGGGAAATCAAGGGAGCCCTGTTCAGCTGCA

AATTCAAAAAAGACTGCACCGGCTCCTTTGCAAGCAAAAATTGAAGATGACTGTTCTTTG

GAAGGTGCATTGAAAACGGAGTATGAGAATTTTGGAGTCGAGGACTATAAGGTGGAGATG

CCATCACCATCACCAGCTTCATCTGACGAATCATTGGCTGGTTCTTCTTCACCAGAATCG

GAGATTTCTTTCTTGGATTTCTCTGATTCCTTGCAGCGGGACGAGTTTGAGAATTTTGGT

TTGGAGAAGTACCCTTCAGTTGAGATTGACTGGTCATCCATCTAA

>EVM0026052

ATGGATCCCTTCTCTACCAAATACACAGAGCATAAAACCGTGACCAACAAGCTGGTGAGA

TGGGAGGACTCAAAAGCCACAAGAATTGTAAGGATTTCAGTAACTGATGGCAATGCAACG

GACTCGTCTGGAGATGAAAATGAGGAACCCAAGATCCAACATCCAAGAATTAAAAAGCAT

ATCAACGAGATAAGAATGAAAAATTGCAGCGAGAGAGCAGCAAGGGATGCGGCAAAATCA

TCAAGACAACAGGCCGTGAAAAAAAACTCAAGGGATCAGTATTATTATCCAGAAGGGAAA

AAATGGGCAGCAGAGATCAGAGACCCTTATAGGAGAACCCGGGTATGGCTGGGGACTTAT

GATACAGCAGAGGAGGCAGCCATGGTTTATGACGAGGCTGCTATTCGTATCAAAGGACCT

GATGCGCAAACCAATTTCACAAAAACTACAGTGAGCAAACAACATACCCCGGATGTTGAT

ATCAACGCTAACATTTCTGGGTATGATTCTGGTAAAGAGTCTCATAATAGTCTATGTTCT

CCAACTTCTGTCCTTAGATTTCAATCTACCGAGGAACCGGGTCCAGAGTCACAGGCCACT

GCTGATGCTGCTGTTCAGAGTGATTCTTTCTGGAGACAAAAAAAAGAGGTGGTTCAAGAA

GAAAAATTGAAGGGCGATGACGATGAGTGTTTGTTAATGGATCCTCTGTGTTTGAAGGAG

TATTGGGATTTTGAAAGTCATGCGCCGATTTTTTTCGAGGAGTGTAGTGTGCCGGATACT

GTTTTGAGAGAAGATTATGCTGAGATGCCGGTGCATCTGGACTGTGATTTTGGCTCCTGT

TTATGGGATGTTGACAAGTATTTCGAGGCATAA

>EVM0034397

ATGGAGCAACCACCATACACAGAAGATGACCCGATCACAAACTCTCCAGAGGCAGCCACC

GCCCTAACCCACCAAAGTCCTGAAACTCCACGGAGCAGCGGCACTCGAAACCCTGTCTAT

CGCGGTGTCCGAAAACGGAGGTGGGGGAAATGGGTGTCTGAAATTAGAGAGCCACGCAAG

AAATCACGTATCTGGCTAGGCTCGTTTCCTGTCCCAGAAATGGCAGCCAAGGCGTATGAT

GTGGCAGCGTACTGTCTGAAAGGTCGTAAAGCGCAGCTTAATTTTCCTGACGAAGTGGAT

GATTTGCCCAGACCGTCCTCGTGTACGGCCAGGGACATTCAAGCAGCGGCAGCAAAGGCA

GCTCATTCAGTGCTGATTCCAAGGCAAAAGAGCATCGAGGCAAATAGTGACAATGGTGCT

GACGGAGAAGTTGCCGGTGATGATTTCTGGGGTGAGATAGAACTGCCGGAATTGCTGATG

AGTAATAGTGGGTGCTGTTGGAATTCTTGTGGATGGAGTACTGTTGCAAGTGATAGCTCA

ACGTGGCAACTGGATGGAGAGGGTCTCCCACAGTTCATGGCATGTCTTCATTAG

>EVM0013573

ATGGATATGTTCTTTGGTTATTCTGATCAAAACCCAAGCGGGTCAATGAATTTGATGCCA

GTTTTTGACGAGCAAGAATGTTCTTATTCTTCTGTTCTTTCAGATAGTAGCATCACTAGT

AGTGTTACTAAAGGAGTTCAACCAGCCGCATTTTTTTCTGATGAGGAAGTAATGTTAGCT

TCCAGGAATCCGAAGAAGAGGGCAGGAAGAAAGAAGTTCAGGGAAACAAGGCATCCTGTG

TATAGAGGGGTGAGGAGGAGGAATTCGGGTAAGTGGGTTTGTGAAGTTAGAGAGCCTAAT

AAGAAATCAAGGATTTGGTTAGGGACTTTTCCTACTGCTGATATGGCTGCTAGGGCACAT

GATGTTGCTGCTTTGGCGTTGAGGGGAAGGTCTGCTTGCTTGAATTTTGCTGATTCTGCG

TGGAGGTTGCCGGTTCCGGCTTCCAGCGATGCCAAGGATATCCAGAAGGCTGCAGCGGAA

GCAGCTGAGGCTTTCCGGCCGGAGGGGTGTCTGGGAGTCGATTTAACGAGGACGGGAGAT

GAGGTTGAGAAGGTTGCAGAGACGGCGGCAGGGGAGGGGTTTTATATGGATGATGAGGCA

GGTTTTGGTATGCCAGGTTTGTTGGCTAATATTGCTGAAGGGATGTTATTGCCACCACCT

AATTATTGTGGTTTTTCCGGTGGGGATAGTTGGGATACAATGGAAAATAATGACACTGAC

ATGTCATTATGGAGCTTTTCTGTTTAA

>EVM0004316

ATGGCCAATCCTGAGCCTCAAGCCGACCAATTACCACCCCTTCCCTCTCCGATCCTAATT

CCTTCTCCGACCAGCAGCTCTTCGGGTCAGCCCTTTGATTCAAGGCATGAACATTCGACG

AGGCTTATTCGATCGCCCATTGGATTTTCGAGTCAGCCCATGAATGGGAGGCATGGTGAC

CAATCACCGAGGCGGCCAATTCGATCACCGGGTGGTTCATCGGCTCAGCCCACAAGCGGA

AGACACCCGAGTTTTAAAGGTATTAGGTTACGGAGTGGGAAATGGGTATCCGAGATCCGG

GAACCACGTAAAACTACACGTGTATGGCTCGGTACGTATCCAACTCCTGAAATGGCAGCC

ACTGCCTATGATGTGGCGGCTCTAGCACTTAAAGGCCCCGACACGCCACTGAATTTCCCG

GAATCAATCCTTTCCTACCCAATACCAGCCTCGGCATCCCCGAGTGATATAAGGGCCGCT

GCTGCTAGTGCAGCATCAGGCAGGTCATCTCAGCGTGAAAGAGGGCCACGTCCTGAAGTG

GGCCGGCCTGAAACAGAAGGCACATCATCTGGTATCACTGCTGGTGCTCAATCAGGACAG

GAAATTTTCGACGAGGAAGAACTCTTAAACATGCCCAATTTGCTAGTTGACATGGCAGGA

GGAATGCTAGTAAGCCCACCAAGAATAAACACACCATCTTCTGATGATCATTCACCAGGG

AGTTCCGATGCTGAAAGCCTATGGTCCTACCCCTAA

>EVM0036425

ATGAGAAAAATCCGTATCATCTGTAATGATCCATATGCCACCGAAACTGATTCGAGCGAC

GATGAATCTGATAGGAGATCTTTCAAAAAACGTTTCGTGCAAGAAATCAATCTTCCTCTT

GCTGTTTCTCCCCAACCAAAAGCCCTTGAGCCCGAGAGTTCCTGTCAAGACAGTAATAAT

AGTGCAAAACCCCCCAGCAAGAGGAGAAGGGTTTTGCCTAAAACCCCAACATCAACAACT

GAAGAAAAGAAACTAGCACCAAAGAAACCCGTAGGTGTTAGGCAGAGGAAATGGGGTAAA

TGGGCAGCCGAGATTAGGAACCCAGTGACCAAAGTGAGGACATGGTTGGGTACTTTCAAT

ACTCTCGAAGAAGCTGCTCAAGCCTACGAGGCGAAGAAGCAGGAGTACGAGGCTGTGTCT

TTGGCTACAGCAGTTTCTGAGAAAAGCCAAATCATGTCATCGGCTGCGGTTGTCTTGCAA

TCTCACTCTCACAATAGCAGCAAAAATCAAGGCAGCAGTGCTTCTGATGAAACGGATGAC

AGCGTTGTTTCACATACTTCCCCAGCCTCTGTTCTTGAACTTGAGCCTGACACTTCTGTG

GTGTCAAATGTCACTGGTGATTGTGGTCTTTTGATTAAAGATGAAGATATCTGTGCCTCT

GTCGCGGATCTTGATATACCTGATCTGGCTTTTATGACCGATACTCTGGCGACTTGTCCG

TTTGATCATGATCTTAACCTTGGACAGGAGTTTGGGAATCTTATTGACGAGTTTGGTCGA

TTCTATGATGATTACTGTGGCATTATTGATCTTGATATCTGCGGTCTAAATGGAGATGAA

CCGAGCGATCTTCCTGATTATGATTTCGAGTTTGGGAACGAGGAGTTTTCTTATTTGGAT

GATCATCAACAGAAACCCTTCAACATCGCATGCCTATAA

>EVM0033748

ATGGATGGAAGCTGCATAGATGAGAGCACAACTAACAGTACTGATAACTCCATATCCATC

ACACCAACCAAACTCACTCCTTCTCCTTCTCCAACTCGAGCTCCCGCAACAAAATCACCA

CCTGATCAGTCACTTTGCCGTGTTGGGAGCGGCAATAGCGTGATTCTTGATTCAGAATCT

GGTGTCGAAGCTGAATCAAGAAAACTCCCTTCTTCTAAATACAAAGGAGTTGTCCCCCAA

CCAAACGGTCGCTGGGGTGCACAGATTTACGAGAAGCACCAGCGTGTGTGGCTTGGGACA

TTCAATGAAGAAGATGAAGCAGCAAGAGCCTATGATACTGCTGCGCAGAGGTTTCGTGGA

AGGGATGCCGTGACGAACTTCAAACTAGTTAATGAAACTGAAGATGACGAGATCGAGGCT

GCTTTTCTGACCTCTCATTCAAAAGCTGAAATCGTTGACATGTTAAGGAAACATACGTAC

AACGATGAGCTGGAGCAAAGCAAAAGGAACCACAAGAGTAACAATGGGGTAAATGGGAAG

CAATACAAGAATACAGCAAACTATGGGAGTAACAGTTATGACCATGGTAGTGACCAGGTG

CTGAAGCCGCGTGAACAACTTTTTGAGAAAGCTGTCACTCCAAGTGACGTTGGGAAATTG

AATAGGCTTGTCATACCAAAACAACATGCAGAAAAGCACTTTCCTTTGCAGATTACATCA

AGCTGTAGCAGCAAAGGTGTGTTGCTTAACCTGGAAGATATGAGTGGTAAAGTGTGGAGG

TTTCGTTATTCTTATTGGAATAGTAGCCAGAGTTATGTTTTGACCAAAGGGTGGAGCCGG

TTTGTAAAAGAAAAGAACTTGAAAGCTGGTGACATTGTTTGCTTTCAAAGATCGACAGGA

CCAGAGAAGCAGCTTTACATTGATTGGAAAGCAAGAAGCGGGTCCAACACGGCCCAACCG

ATTCAGATGGTGAGGTTGTTTGGGGTCAACATTCTCAACGTACCTGGGATGGAAAATGGA

TGTAATGGGAAGAGATCAGTGAGAGAAATGGATCTTTTGTCTTTAAATCATCAATATAGC

AAGAAACAAAGGATCATCGGAGCTTTGTAA

>EVM0006637

ATGGATTCCACTTCTCATCAGAACTGGCTCGGTTTCTCTCTTCCCAACCATCATCACTTC

AATAATAATATCAACATCCCTTCCTCTTCTGATTCCTCTCACCTCTCTCTCTTCCAAGCC

TTTATCACCGCCCCCACTGCCTCAGCGCAAGAAGTAAATGCAGCAGTAGCTGCTGGTCGA

GCTACGGACATCTCTTTATTTACAACTTCTGGACCAAAACTTGAGGACTTTCTTGGTAGC

TGCACCACTACATCCCCATCTCAAACACCACAACAGCCGCTGTGTGGCCAGTTCTCTACT

GAGACACCCGTGACTCTTACCGCCACTACTTTATCTGACAGTACTAGCTCTGAGATATAT

GATTCTGAGCTCAAAACTATAGCCGCTAGCTTCCTTAGTGGTTATGCCTCTGCTGATAAT

AAAAAAATCGACAGTAACCAAAAACATCAACAACTTCTTGTTCAGGCTGAACATGCACCA

AAGAAAACTGTTGAAACTTTTGGCCAGCGTACTTCAATCTATCGAGGAGTCACCAGGCAT

AGATGGACTGGAAGATATGAAGCTCATTTGTGGGATAATAGTTGCAGAAGGGAAGGCCAA

AGTAGAAAAGGAAGACAAGTTTATTTGGGTGGCTATGACAAGGAGGAGAAAGCAGCTAGA

GCTTATGATCTTGCAGCTCTCAAGTACTGGGGTCCGACCACCACTACAAACTTTCCGGCT

TCTAACTATGAGAAGGAAATAGAAGGGATGAAGCACATGACAAGGCAAGAGTTTGTTGCG

TCACTTCGAAGAAAAAGTAGTGGTTTCTCTAGAGGAGCATCGATTTACAGAGGAGTGACG

AGGCACCATCAGCACGGTCGGTGGCAAGCAAGGATAGGAAGAGTTGCTGGGAACAAGGAT

CTCTATCTTGGTACCTTCAGCACACAGGAGGAAGCTGCCGAAGCCTATGACATTGCAGCA

ATCAAATTTAGAGGCCTAAATGCTGTTACCAACTTTGACATGTGTCGCTATGACGTGAAA

AACATAGCTAACAGCAATCTTCCCATTGGAGGAATATCCGGCAAGTCAAAAAATTCCTCA

GAATCAGCTTCCGATAGCAAAAGCATTGATGGAAGCCGATCGGACGAGCGAGATCTCTCC

TCCGCATCCTCAGTAACCTTTGCTTCTCAGCCTGCAACTTCTACTCTTAGCTTTGCCATA

CCCATCAAACAAGACCCGTCAGATTATTGGACCAATATCCTTGGATACCAAAACACGACC

ACTGCTATGAACAATGCCAGGAACAGTAGCAGCATTGTTGATCCAAGCGCTTTACTTCAA

TCTTCCACAAGCGGTCCTGCTTTTCAAAGCCCGACAGGTTTTAGTATGGACTTCAATGCA

AATTCATCTGTCAACGAAAGCAATAACAGTGGGCTATTATTATTCAATGGTGGTTACACA

CAGCAGCAGAGTGGTATTTCTACATCGTCATCAGGTTCAAATATCCCATTTGCTACACCC

ATCGCCTTCCATAGTAATGGTAGTACTTACGAGGGTAACCCAGGTTATAGTAGCTGGATT

GCTCAGTCTTTGCATTCTTTTCAATCTGCAAAGCCCAATCTCTCACTGTATCAGACTCCC

ATTTTCGGAATGGAATGA

>EVM0019656

ATGAAAGGGTCTTCATCCTGCTCCTCCTCCTCCTCCTCCTCCTCGTCTTATTGCCTTGCC

TCTGAAAGCATTCAGAAACCAAAAGTCAAACGCATTGGAAAAAACCAAAAGCGCAATCAA

GGGAAATCCAGAATGATGCTGCCACATCGGTGGACGGGAAGGTTTGAAGCTCATCTTTGG

GACAAGAGCTCACGGAATAACATTCAAAACAAGAAGGGAAAGCAAGTTTATTTGGGTGCT

CATGACAATGAGGAGGCAGCTGCACACACCTATGATCTTGCTGCCCTGAAGTACTGGGGA

GCAGAGACAACCTTGAATTTTCCGGCAGAAACCTACACAAGAGAGATTGAAGAGATGCAA

AGGATGAGCAAGGAAGAGTACTTGGCATCACTTAGACGGCGAAGCAGTGGATTCTCCAGA

GGAGTCTCCAAATACCGCGGGGTGGCCAGGCATCATCACAATGGGCGATGGGAAGCAAGA

ATTGGACGAGTTTATGGGAATAAATATCTCTATCTTGGAATTTATAATACGCAAGAAGAG

GCAGCGGCAGCATATGATATGGCAGCAATAGAATACAGAGGAGTAAATGCTGTGACCAAT

TTTGATGCTAGCAATTATATAGAACGGCTGAGGAAGAAAGGCGGCATCCCTATAGACCAT

ATCCTCCAAGAACAACAACTTCGTAACAGCTCGATTGATTCCGTCATAGAAGCAGAAGCA

GAAGCTGAACAACCATCAACCGAACAACAAGAGGAAAAGGAACAAAATGTAGCTCCGTCG

TTACAAGTTCAATGTACACAGCTCAATTCAAGCTTGGATGGCACATCTTCTATGGTTACT

TTGGAGACTATCGAAGAGAACGAGCTAGCATGGAGCTTCTGCATGGATCCAGGACTGAGC

CTCGCAATGCTTGATCTTCCCCTCAAAAATACTTGGGAGTTACCGGACCTGTTCAATCAT

ACAGGGTTTGAAGACAACGTTGACTTAATATTCGATTCATTAATCTTCATGGATCAGTGT

TTTGAACTGGTTGCTTTTGATTAA

>EVM0040690

ATGGAAGTGAGTGATAGTACAGCTAACCATGGTGGTATATCTTCTAGCTATAAAGGAATA

CGAAAGAGAAAATGGGGCAAATGGGTGTCGGAGATACGTGAACCTGGGAAGAAAACTAGA

ATATGGTTAGGCAGTTTTGAGACACCAGAAATGGCAGCAACTGCTTATGACGTTGCGGTT

TTACATTTTAGGGGACGTGAAGCAAAGCTTAATTTTCCTGAATTGGCTGGTAAGCTACCA

AAGCCAGCAAGCTCCAATGCTGACCACATTCGCATGGCAGCTCATCAGGCAGCTATTAGC

CTTAGGCCCTATACTGCTGACTCGTCCCACGGTGGTGGCTCTAGCTCCAATGTCGGGCCT

ATCACGGTTAGGCTCTCGCCAAGCCAAATTCAGGCCATCAATGAGTCACCATTGGATTCA

CCGAAGATGTGGATGCAAATGGCAGAGATAGCAACTCTAGAGGAGTCCTCCATGATGTTC

TCTAATGACAATATTGAAGAGGATGAGTGGGATAACAACCAAATAGATTCTCTTTGGGAT

CCTTAG

>EVM0055308

ATGAAAGAAGGAATCAAACAAAGCAATCAAACACTTCCTGTTATATATATCAGTCACTCA

ACGTTTCTCTTCTGCTCCACACAACTTGCTATAGAGACTTCCCTCATGGAAAAACATACC

AACCTTGAGATCGATGCTCAAGACAGCTCCCTTACTTCATCTTCCACCACCACCACCACC

ACCGCTTCCTCCTCCTCCTCCTCCTTTTCTACCACCATTACTTATACTGATCGAAGCTCA

AAAAAAGAAGAGAATTCTATTGCTGGTCCAAAAGGATCGAGAAAATCTCAAGAATTGCAG

GGTGGCAATGAGGGTAATAAAAGGCGAAAGACTACCGATAATGAGAATAATGGGAAGCAT

CCAACATATAGAGGAGTGAGAATGAGAAGCTGGGGCAAATGGGTGTGCGAGATCAGAGAG

CCAAGGAAGAAGTCAAGAATATGGCTTGGAACATACCCTACAGCCGAAATGGCTGCTCGA

GCTCATGATGTAGCCGCCTTAGCCATTAAAGGCGGCTCAGCTTACCTCAATTTCCCTGAA

TTGGTTGATGAACTGCCTCGTCCACTCAGCAAGTCTCCTAAGGACATTCAAGCTGCAGCT

GCCAAGGCAGCCGCCGCTTCGTTCCCCGAAACAAGACACTGTGAGGCAGAAGCTGACCTG

AACCGAGCGGAGCTGAACGTTTCGAATTTATCAGATAATTTGGCCATGGACAACACACAA

GAATCAAGCAGTTCTCCTTCAACTGATGTTGATGACAAGTTGTTTGACCTTCCTGATCTC

TTCATCGACGGCGTTAATCACAGTGATGGATTTTGCTTTTATTCTCCGCCATGGCAATTA

TGCTCAACTGATACCGGCTACCGGCTTGAAGAGCCATTTTTATGGGAGTACTAG

>EVM0052610

ATGTCGCAAGGAGGGAACAACAGCTTAGGGTCTGAAGATTTAGATTTCTTGGAGTCCATT

CAGCAGTATCTTGTTAGCACTACCGATCGTGATTTTGATACTCTGATGAGTACTTGCCTA

CAAATGGAAAATAGTGAGGGTACTGCTTCAAGCTTCTGTAGTGATCATGATATCCTCTTT

AATGATTCAATGACCTCAACAACGTTAGCGGTCTTTGAATCGGATCCTCAAGGGCTGGGC

ATTCCTGCGGTGGAAGTGGCGTGTAAGAGTAACGCGCTGCCGAAAGGGAGTCAATATAGG

GGGGTGAGGAGGAGGCCGTGGGGGAAGTATGCGGCGGAGATTAGGGACCCGAAGAAGAAT

GGAGCAAGGATGTGGCTTGGAACTTACGAGACACCAGAGGATGCTGCTCTTGCTTATGAT

CAAGCTGCTTTTAAGATTCGTGGAAACAAAGCTAAGCTTAATTTTCCTCATCTAATTGGT

TCTACAGAATATGAACCAGTTAGAGTGACTTCCAAGAGAAGATCTCCTGAGCCTGGATTG

GCGCATGATGGCTCGCCCAAGAGAAGAAAATGA

>EVM0053966

ATGTTTGGAGAGAAGAGCTTGGAATCAGGCCTTGAATTCAATATCAATACTGTTTCCAGT

ACCAGTACTGTTAACGCAGCAACATATTACAAGAGCCTCAGCTTTAGTAATATTTATTCA

ACAGAAAGTTGGGGTGATTTGCCATTCAGACAAGATGATTCTGATGATATGATCGTTTAC

AATGCTCTCCGGGATGCCTTTCCACAAACAACTGTTCCATTGGAAGTGGCGCCGCGCGAG

CCTGGAGCGCCGCCGCTGAAGGAAAAGGGATTGCAGTACAAGGGTGTGAGAAGGAGGCCA

TGGGGAAAATATGCGGCAGAGATTAGGGACCCCAAGAAGATTGGGGCGAGGATGTGGCTT

GGGACTTATGAGAAGCCTGAGGATGCTGCACTAGCTTACGATCGAGCTGCTTTCAAAATG

CGTGGCTCGAAGGCTAAGCTTAATTTCCCTCATCTGATCGGATCCAACGATATCTGGGAA

CCAGCTACAGTAACTTCTAAGAAGCGGCTTTCAGCAGAGCCTTCCATTTCAGATTACGAC

TTGCAAAGTTGGACGAAGCAAATCAAGATCAGTTCGGACGAGTCATTGGTAGCCAGTTAT

TTTAGCTGA

>EVM0006944

ATGGATTCTTCATATTTTCCATTCCAAAACTCTCTATTCTCCCCGGAATCATCTTCTTCT

TTCAATTCCCGTGATTCCTTCTCATGTAACATACAAAACTTCTACAGCCAGCCCCTCTCT

CTTAATGAAAATCATGATTCCCAGGAAATGTTTCTCTTGGAAGTCCCGAATGAGGTCCCC

GTCAACTCCTTACACACCTCATCATCCGCCGATACTACCTGTGACGAAGCAAGCTCCAGA

GCTAATTATCAAGATGAGCCACCCCAACAAATTGCTTACCGAGGGGTTAGGAAGCGGCCT

TGGGGCAAGTTCGCTGCCGAGATAAGGGATTCAACTAGAAAACATGTGAGAGTTTGGTTA

GGAACTTTTGATACTGCCGAGGCCGCAGCCTTAGCTTATGATCAAGCTGCATTTACAATT

AGAGGTTCGATGGCCGTTCTAAATTTTCCTGTCCAAAAAGTTTACGAGTCACTTCAGCAA

ATGAGCTATGGATTCCAAGAAGGGAAATCACCGATTGTCGCCATGAAAGAAAGACACTCA

ATGAATCGGAAAGCCGAGAGTAGGAGAAAGAAAGAGAAAGGAAGGAGAACTGAAATGGAG

AATGTGGTGGTCCTTGAGGATTTAGGGGCTGATTACTTGGAAGATCTTCTAACCATTTCA

GAGAGTGCTAGTCCTTGGTAA

>EVM0044050

ATGTCTATAATTCATGAACCTCCTGGATTGAAAACAAACCTCCATGCGTCCGAACCAGCA

TCAAAACGGAGCTCCAAGAAGAGGACCCGCCAACCTGAAGCCCCATTTCATGGGGTAAGA

AAACGGAGTTGGGGCCGTTATGTATCTGAAATCCGGTTACCCGGCAAGAAGACCCGAGTA

TGGCTAGGATCATTTGGGTCACCCGAGATGGCTGCCAGGGCCCATGACTCGGCCGCTTTC

TTTTTAAGAGGCAACTCGGCGAGTCTCAACTTCCCTGACTCAGTCGGGTCACTTCCCCGC

CCCGATTCTTGCTCTAGCAAAGACATCCAATTGGCAGCGGCGAAAGCTGCAATTGGGTTC

GAGAAAGCGAGGGCAGGTAACGGGGTAGGGATCGAGGATAGCGAGTCAGGTGGAGAACCC

GAAAGTTCAATTTGGCTGGGTTTGGGTGAGATAGAAACGGTGTCGTATGAAGAAGTGAAA

CGAACTCCTTTGTTTTGTCCTTTGAGATTCGATTCAGTGAGTGGAAATGACGAGATGTTG

TATATGAACGATCAGGGAGCTTTTATTTGCTAA

>EVM0030182

ATGGAGGATCAGTTTCCAAAAATAGAAACCTTCATGCACAAGAAACTGGCAGCTTGTAGT

AGTTTTCATGGAATGGCAACAGGCTCGAAATATTTTGGTGATCCAACCAACTGGGGTGCT

CTATGTAGGACAGCTTGTGGCGTCGAGGATGGAAAAAGTGGCATGAAATCGCCGTCGCAA

TCGTCTTCTGAAAGATTATTTTCGAGTTCCGAATCTAGTTCTTCGGCAGATGATCAGGTT

TCAGGTACCCATTTGATCGGTAACATCCCTGGAATTGGTAAACAGGGTGTTGTCAAACAT

AATCATGAAAGTTGTTTTATGTCAGGATTGAATTCTATCAACGCTCATGGAGCTAATTCA

ATATCTGATCAAACTATCAAAGAAAGCTCCATTCCTGTGAATTTCTTGGAATCTTTTCCA

ACATTAAATAACCAAGCTCAAGTACCTGAGCCTCCTTCTCCATCTTCGTTATCAAACACA

TCAAATCCCCCAAATTTAACCTTGTTTTTGCAGGAACCAGCAGCAGCTTCATTGCTAGAT

CCGTCCAAACAAGTTCTCGATCCACCTGGCAAGGATCAAAGATGTGAACCTTCCATTTCA

TTGTTTCCAAACATTTCATTTTCCATGCCTCAACTTGACCAAATTGATTTCCAGCCGAGT

AATGAATGGTTCAAAATCAATCAGACTTTGGCAAATTATTCTACCAAAGGATTTAACGAC

TATTGGCTTAGCACTACGAAGACACAGCCGATGAAATACACGGGCAGAAGACTGCAGAAC

CAGCACCTGAACCCTTCTATTTCATCGGCATCAACCTCGCCGGGAAAGCTGTTCAGAGGA

GTGCGGCAAAGGCACTGGGGAAAATGGGTTGCAGAGATTAGGTTACCAAGAAACCGGACG

AGGGTCTGGTTAGGGACCTTTGACACAGCAGAAGAAGCTGCCATTGCTTATGACACAGCA

GCTTACATGTTACGAGGAGACTACGCGCATTTGAATTTCCCAGATCTTAAGCATCAACTG

AAGTCTAATTCATTGAACAGAACCACAGCTGCCCTTCTTCAATCAAAGCTACAAGCAATA

TCACAAGGCGACAACATTTCCTGTGATCAAAATAAGCATAGCAGCGATCATCCACCATCA

CCAAACAAGATTAATGTGCATGATACAGATGCTAAAAAACTCAAAGGTCCGAGCCAAAAT

ACAACAAGAAAAGAGCGGCAATTTGAGTTAGGAGGCAAAACTGGAGATGAAGTGATCGAG

AGCAAGAAAGGTCAAGATGTTGTAGCATCGGATATTGAAGCTGTTCAGCTTAGTAGAATG

CCTTCCTTGGACATGGACATGATCTGGGATGCTCTTTTAGTCTCTGACTCATGA

>EVM0000006

ATGAACCACCAAAAACTACTCCCCGTTCCGTTCTTCCCTCAACCCGAAACATGTCCAATG

TTTCGGCTCCTGACTGAAAATTCAACGTTTTGTGGTTCTTTTTTAGAACCCCAAATTTCA

TTTTCTTCACCTTTTACCTTTAACAACTACAACCCCACAAATTGCTCTCCTGTTTCACTA

GATATCCCGGCTGCTGTTTCGCTCTCTGATGGATTCAAGAAATCACCAGAAACAACACCG

GTCCTTGATGGTATCGGTGCTGTTGTCGGTCAACAAGTTCTTTTTGGCAAGAACGATACC

AAGAATTCAGAAGCATCGAAGCTTTCTTCAATTTCTCAACGTACCGGGTCAAAAACAAGT

AAACAAGAGAGTGGCGCTATGAGAAACAGTGTTAGTGACGGGGTTTCAACACAAAAGGCC

TACAGAGGAGTGAGAAAGAGACCATGGGGGAGGTGGTCAGCTGAGATAAGAGACCGGATA

GGAAGGTGTCGCCACTGGCTTGGAACTTTCGACACCGCAGAGGAGGCAGCGCGTGCTTAT

GACTCAGCTGCTAGAAGACTTAGAGGAGCAAAAGCAAGAACTAACTTTGAAATCCCTCCT

GTTTTGCCTACCATATCTCCTTCCTCTTCTCCACCAAGCGCATGCTCGGGCTCAATCTGT

AACAATAACGCAGAAGCGAGGAAGAGGACTAAGGTCTGTAACCACAGCAGCAAAATGATC

GGTAGGAAGTGTGCAGTTGTCACTTCTGTGGCACATTTGTTTAGTGATTTTGAAGGGAAA

GGAACTAGTTCCGGTGGCAACATGGAGCTCGAGTTGAAGTTGGGAATGGGTCCTGATGGT

TATCGAGGTGGCAACAAGATCGCCTCCGGCGCTCCTCCATATATGGTGGTTTAG

>EVM0025220

ATGGAAACACATCAAGGAGAGAAGAGCACCAAGAAGCAAGAGAAAGGAAGAGGGGAGAAT

GAGTATAGGGGGATTCGAAGGCGACCATGGGGTAAGTTTGCTGCCGAGATACGTGACCCG

ACAAAGAACGGGGCACGTCGATGGCTTGGAACATATGACACGGCGGAGGAGGCGGCTCGG

GCATATGACCGAGCTGCTTTTGCCTTCCGAGGTCATTTAGCCATTCTCAATTTCCCGAAT

GAATACCAGCATCAGGATCCGAACTCTGCCATGTTATGTGGTTCTTCATCTTCATTCTCC

ACTGCCGATCCAGTGAATTATGGCCATGAAGTTTCCTCCACTGGTGGACAAGAAGTTATA

GAGTTCGAGTATCTGGATAACAAATTGTTGGAGGAGCTGCTGGGAACGAATGATCCCAGA

AGGTAG

>EVM0035905

ATGGAACCTTCTTTGTTTTACCATGATCAAAACCTGAATTCTGATTTCTCCCCTGAATCT

TCTTTTGGTTCTTTAGATTCATTTTCATGGGATGACCTTCTTTTCCAGAGCAATCCACTT

CCTTTTAACACCAATGACTCCGAGGAGATGCACCTTTCTAATGTTTTAGCCGATGGAGCG

CCCAAGGAATCCTCGGAATCTAATTCCTCGAGCGGAATTAAGGAAGAGGAAGTGACTTCA

AATGCGAAAGAAGAGGAAGCGGCTAGGAAAGAGAAATCTTACAGAGGGGTTAGAAGGCGG

CCATGGGGGAAGTATGCTGCAGAGATTAGGGATTCTACAAGGAATGGGATTAGGGTTTGG

CTAGGGACCTTTGATAGTGCAGAGGCAGCTGCTTTGGCTTATGATCAAGCAGCATTTTCC

ATGCGGGGTTCGATGGCCGTTCTCAATTTTCCGGTGGAGATGGTGAGAGAGTCGCTTCAA

GACATGAATTACGGATGCGAAGATGGGTGTTCGCCTGTGGTGGCACTGAAGAGGAAACAC

TCTATGAAAAGAAAATCTACAAGTAGGAAAAGTAAAGTAAATCAAGTCCCTTATTCTAGG

CCACAAAATGTGGTGGTTTTGGAGGATTTAGGAGCTGACTACCTGGAAGAGCTTCTGAGT

TCATGTGACAGTTCTAAGTATTGGTGA

>EVM0045646

ATGGATCATTCTTTTTTCAGCCACCCAGATATAGAATTCTCGCCATCATCTCCCTCCAAC

TCTCTAGATCTACTGCAGGATGGCCTTCTTTTTAACCCTCATGATGATTTCTATCTATTC

AGCTCAAATGACTCTGAGGAGACACTTCCATCTGATGTTCCTTTCACCGAAGCCAAAGAA

TCTGCTGATAGCATTGCCTCCGATGCCCCACAGAATAACGAGGCCAAAGACTTATCGGAA

CTTGTTTCCTCGAATGAGATCGAGGAAAAAGAAGCCACTCCGAATGCTAAAGAAGAAAAT

CCCGGAAGGGAAAAGACCTATAGAGGGGTCCGGAAGCGGCCGTGGGGAAAATATGCGGCG

GAGATAAGAGATTCCACTAGAAATGGAGTGCGAGTTTGGCTGGGCACTTTCGATACTGGT

GAAGCAGCTGCTTTAGCTTATGACCAAGCTGCATTATCTTTACATGGCTCGAAGGCTGTC

CTCAATTTTCCGGTTGAGAAAGTCAGACAATCATTAGGAGAAATGAAACGTGGTTTAGAG

GATCGGTGGTGCTCCCCTGCCGAGGCACTGAAGAAGACTCATTCGAAAAGAAAGGCGGTG

AGTAGGAAAGGGAAAGGGAAAGGGTTAGCAACCAAGGAAGTGGTGGTGTTCGAGGATCTC

GGAGCTGAATATTTGGAAGAACTTTTAAGCTCTTGTGAGAGAGGTACTCCTCAATGA

>EVM0055887

ATGGGTACTCTTATTCAAGGTTCTAACGACACTTCGATGTCCATGGATTCTACTAAGAAG

AGGAAAAGGGCAATTAATAAATCTGTAGCTATGACGCTTCAAAAGTGGAAGGACTACAAT

GAGTACCTTGATGCTCAAGGTTATGGAGGTAGTAAACCAGCTCGTAAAGCTCCTGCCAAA

GGCTCAAAGAAGGGGTGCATGAAAGGTAAAGGAGGGCCAGAGAATTCATTCTGCAATTAC

AGAGGTGTGAGACAGAGGACATGGGGGAAGTGGGTTGCTGAGATTCGGGAGCCAAACAGA

GGGCCTAGGTTATGGCTTGGTACTTTTCCTACTGCCTATGAAGCTGCTCTTGCCTATGAC

GGTGCTGCACGAGCTATGTATGGTCCTTGTGCTCGTTTAAACATTCCAGATGCTGTGAAT

TCAACAAGCTCATCGAAGGATAACTGCTCTGTTGTTACACCGTCATACTATTCTTCAGCC

GCAAGCCCTGCTGACTCCGTCGCTACATCAACCCACTCTGAGGTTTGTGCGTACGAGGAT

CCTAGCCAGAATGGATTAAGCCAAGCCGAGGACTGGAGGACAAATATATCAAGCCAAGCT

GAGGTTTGCGAGCAGAATGTATCAAGCCAACATATCGAGGATTGTTCTCGGGGAGTTGAA

AAAAGTAGCAAGCTTAGTCAAGATGAGCTGAAGACTCAATCTGAAAACCCTTTGTTGACC

AGTGACTGGGACAACTACTCGTGGGATGAGATCTTTAGCGTAGAAGAGCTGCTAGCAGGG

GATATCGATTCTGGGATGACAGGAGCAGAGGGGTACTTCAGTTTAGGATTTTGA

>EVM0022735

ATGGCCTCCACGAATAAGTGGCTAGGTTTCTCGCTATCCCCTCAAGAACTTCCATCATCA

CAGTCTGATCATCAAGATCACTCTCAAAACAAAGACTCTCGCCTTCGTTTCCACTCAGAT

GAAATCTCTGGTGCAGATCATGTCTCTGGGGAGAGCTTTGATCTTACTTCTGACTCCACC

GCTCCTTCTCTCAACCTCCCTGCCTCTTTTGGTACATTTGAAGCCTATAGAAACAATCAA

TCTCAAGATTGGAATAATATGAAGCGTTTTGGCATGAATGCAGACCCCAACTACAGTACC

ACCTCAGACGTCCCGATATTCTTGGGTTCTTCATGCAATAGCCAGAACATTGATCAAAAT

CAAGAACCTAAGCTCGAGAACTTCCTCGGTGGCCATTCTTTTGGCAATCATGAACACAAA

CTCAATGTCTGCAGCACCATGTATGATACCAGTGGAGACTATATGTTCCAAAACTGTTCC

TTGCAACTCCCATCTGAGGATGCATCAAATGAAAGAACAAGCAGTAATGGAGGAGGTGAT

ACTAGCATCAACAATAATAACACCAATAGTTCCATTGGTTTATCCATGATTAAGACCTGG

TTAAGGAACCAGCCAGCACCGACACAACAAGACACAAGCAATAAGAGCAATGGCGGTGCA

CAAAGCTTGTCGCTTTCAATGAGCACCGGATCACAATCTGCAGGTTCTGCTTTGCCACTT

CTAGCAGTAAATGGAGGAGGAAATAGAACTGGAGGAGAGCAGAGTTTCTCTGATAATAAC

AAACATCAAAAGACCACACCGAGTATTGGTACCCAAACCGGTGCCATCGAAACGGTACCG

AGAAAATCTATTGATACTTTCGGCCAAAGAACTTCTATATACCGTGGTGTAACAAGACAT

AGATGGACTGGTAGATATGAAGCTCATCTATGGGACAATAGCTGTAGAAGAGAAGGGCAG

ACTCGCAAGGGAAGACAAGTTTATTTGGGTGGTTATGACAAAGAAGATAAAGCAGCTAGA

GCTTATGATTTAGCAGCATTGAAATACTGGGGCACTACCACCACGACAAATTTTCCGATT

AGCAACTACGAGAAAGAGGTAGAAGAAATGAAGCACATGACTAGGCAGGAGTATGTTGCG

TCTCTTCGAAGGAAAAGTAGTGGGTTTTCTCGGGGTGCTTCCATTTATCGAGGTGTAACT

AGACATCATCAACATGGAAGATGGCAAGCAAGGATCGGAAGAGTTGCAGGGAACAAAGAC

CTTTACTTGGGAACTTTCGGCACCCAAGAAGAAGCAGCAGAGGCCTATGACATTGCCGCC

ATAAAATTCCGTGGACTGAATGCAGTAACCAACTTCGACATGAGCAGATACGATGTTAAC

AGCATAATTGAGAGCAGCACATTGCCTGTCGGGGGTGCGGCTAAGCGGCTGAAAGAGGTG

GAGCATGCTGAAATTACGACGGATGTGCAGAGAATAGATGATCGTGACAGCTCGAGCTCA

CAACTCATCGATGGGACTGGCAACTATAGTGCTGCGGCACACCATGGCTGGCCTACTATT

GCGTTTCAACAAGCTCAGGCTTTTAGCATGCAGTATCCCTATGGCCAGAGGGTTTGGTGC

AAGCAAGAACAGGACTCTGACAATCATGGCTTTCAAGAGATTCATCAATTACAACTGGGA

AACTCCCAAAATTTCTTCCAGCCCTCGGCTCTACATAACGTCATGAGCATGGACTCGTCT

TCAATGGAACACAGCTCCTGTTCTGATTCTGTCATGTATAGCGGCGGAGGTCACGATGGT

ACCAGCACTGGAACCAACGGAAGCTATCAGGGAATTGGTTATGGAGGCAATGCTGGGTAT

GCCGTCCCAATGGCTACGGTTATAGCTAATAATGGAAACACCCAAGATCAAGGGAGTGGT

TATGGAGATGGAGAGGGGAAGGCTCTTGGCTATGAAAATATGTTTTCCTCGTCGGATCCT

TATCATGCTAGAAATTTGTACTATCTTTCCCAGCAATCATCTTCCGGTGTGATCAAGGCT

AGTGCATATGATCAGGGCTCAGCATGTAACAGCTGGGTGCCAACAGCTGTTCCTACCATT

GCAGCAAGGTCTAACAATATGGCTGCTTGCCATGGAGCTCCACCACCTTTCACCGTATGG

AATGAGAGTACATAA

>EVM0040588

ATGGGAAAAACATCAAAGCAAACCCTAAAGGACTCTGCAAACTCCAGCACAAATCCCACG

AGCAAGGTGAAGCGGACGCGAAAAAGCGTGCCTCGAGACTGTCCACCTCGACGTAGCTCC

GTCTACAGAGGCGTTACGAGGCATCGATGGACTGGACGTTATGAGGCTCATTTGTGGGAT

AAAAACAGCTGGAATGAATCGCAGAACAAGAAAGGAAGACAAGTGTATCTAGGAGCCTAT

GACGATGAACAAGCGGCTGCACATGCCTATGACTTGGCAGCATTAAAGTACTGGGGACAG

GATACCATCCTCAATTTCCCATGGTCAACTTACGAAGAAGAGCTCAAGGAGATGGAGGGC

CAGTCAAAAGAAGAATATATTGGATCATTGAGGAGAAAAAGTAGTGGATTCTCACGCGGG

GTGTCCAAATACAGAGGCGTGGCCAGACACCATCACAATGGAAGATGGGAAGCTCGGATC

GGCAGAGTTTTCGGCAACAAATACCTCTACCTTGGAACATATGCCACTCAAGAAGAAGCA

GCAACAGCGTATGACATGGCAGCCATAGAGTACCGTGGAGTTAATGCGGTTACAAACTTT

GACCTAAGCCGCTACATCAAATTGCTACGTCCCGATATTACCCAAAATAATCTTAAACAA

AACCCTAATGGCGATGCTAATCGGATTCAAGACCTTGACCAGAAGACTGAAATGGATTTC

ATGCCCCACCAGCCAAGCTCGAGTGTTAATGAGACGGAAGTGACCGACCCGACTCGGCCT

GCAGGCGGCTCGGCATCATCGGCGTTCGGGCTTTTACTGCAATCCTCTAAGTTCAAGGAA

ATATTGGAGAGAACATCGGCTGCCGATCCCCTCTTGACGCCACCAGAATCGGATCGTGAT

CCGCCACGCCGGTGCTTCCCGGATGACATCCAAACTTTCTTCGACTGCCAAGACTCCAGT

AGCTACACTGACGATGATGATATTATTTTCGGAGAGCTAAATTCATTTGCTTCATCGATT

TTTATAATACTTTATTTTTATTTTTATTTTTGA

>EVM0049667

ATGATGGACCAGAGTATCCTATGTCCCATAAAATACACTGAGCACAAAAAAGTTATGAAA

AAATTCACCAAGCCAACCTTCAAGACTAAAAAGCTAGCCTCCGATGACCGGTGCCGCTTG

CTAGAACCATCAAACCCACAACCAAGGCTCGTCAGAGTAACAGTTACCGACCATGATGCC

ACAGACTCTTCTAGTGATGAGGAGGGATTGTTGTTTGGCCGGCAAAGGGTGAAAAGATAT

GTGAACGAGATTAACATCCAAGCCAGCTGCAAAGAAACTAATGCCATTGCTCCTACTGTT

GCCAGTAACCGGAAAAGAAGTGTCGGCGATATTCCTCAACGGCCAACGAAAAAAATTGCT

CCTCAATCAACTAACAACGGCAGAAAGTTCCGTGGCGTTCGGCAAAGGCCTTGGGGTAAA

TGGGCGGCAGAGATAAGAGATCCTACCAAAAAACAGAGGTTATGGTTAGGGACTTACGAC

ACCGCTGAGGAGGCTGCTAGAGTGTATGATAATGCCGCCATCAAGCTTCGAGGGCCGGAT

GCTTTGACAAACTTCATCACACCACCTAGCAGGGAGGAGCAAGAACAAGAGAAAAGCACA

TCGGAAGAACCAGAGGGGGCAGAAGAAAAGAAACCAGAAAGCAATGTTGACACAGTTTCC

GGCTCCGGTTATGAATCTGCTGACGAGTCTCACAGTCTGTCATCTCCAAAATCTGTGCTC

ACGTTCAGATTACCTTCGGCTGAATCGCACAAACCACCTGTCAACCCCTTCCAAGAAGTT

AAAACGGAGCCACGTTCTCAAGACAATCAAGAAGCGTTCGGGGAGAGCAACTCGACTGAC

TATTTACCTCTAGACGCGCCGTTTCTTGATGATTTTTTCAATTTTGAAACCTCAGGGCCA

ACACTCTTTGATGATCAAGCGACGGGGACAGCATCGGTTTTTGAGGAGACCTTTGTGTCT

AATCAAGATTGGGGTGACATACTCTTGACCCGCTTCAAGATTTCAGTTCATTATCATCGG

CTTGCTTGGATGACGATGATTTCTTTCATTTCTTGA

>EVM0022064

ATGAGTGGCAGCAGCAGCAGCAGCAGCAGCAGAGCAGATCAAGAAAGTGGAGCCAGCGCA

GCTCAGAAGAAATTCAAGGGCGTGAGGAGGCGAAAATGGGGCAAGTGGGTGTCTGAAATT

AGGGTTCCGGGCAAGCAAGAACGGCTTTGGTTAGGCTCTTACTCCACACCAGAGGCAGCT

GCGGTGGCTCATGACATAGCCTCGTATTGCTTACGCGGACCTTCTTCTATAGAGAGTCTG

AATCTCCCTCTCTTGTTGCCTGCAAGTGTAAGGGATGACATGTCACCCAAGTCCGTACAG

AAAGCTGCTTCGGATGCTGGCATGGCTATTGATGCTCAGATCATACGAAACAGGGTGCCA

GAAAATGAGGTCAGGTTCGGGACAGGAAGTGGTACGGTGAATCATGGATTATGGGAACCT

GCAGGTGGTGATCATGGCGATAATTGGCATGGAAACAATATTAGAATGAGAGAAGGGGAC

ATCTCCATTGAAGATTATCTCTAG

>EVM0005180

ATGGAAGGGAGAAACACCGGTAGCCATGGTGGCATGAGTTCTTGCTACAGAGGGGTACGA

AAGAGAAAATGGGGCAAATGGGTGTCAGAGATACGTGAGCCAGGGAAAAAAAGTAGAATC

TGGTTAGGGAGTTTTGAGACGCCAGAAATGGCAGCCACAGCTTATGATGTTGCGGCTTTA

CATTTTAGGGGATATGATGCAAAACTTAATTTCCCTGACTTGGTTCATAAACTATCAAAG

CCAGCAAGCTCTAATGCTGAAGATATTCGCATAGCAGCCCATGAGGCTGCCATGAGCCTC

AGGCCCTCTACAACTGAATCCTCCCATGGCGGCAGCTCGAGCTCAAATGCTGGTCCCGTC

ACGGTTAGGCTCTCACCCAGCCAAATTCAAGCCATTAACGAGTCGCCCTTGGATTCACCA

AAGATGTGGATGCATATGTCAGAAGTGGCAATGCTAGATGAGTCCATGATATACTCCATT

GACGCTGATGAGGAGGATGAGTGGGATAACAAGCAGACAGATTCTCTATGGGATCCTTAG

>EVM0050604

ATGGAAGGAAGAACCAGGGACGGCCACCTGGGGATTAGTCCGCCGAGATATCGAGGTGTG

CGGCAGCGAAAATGGGGGAAATGGGTGTCCGAAATCCGGGAGCCTGGTAAGAAAACAAGA

ATTTGGCTTGGGAGTTATGAGATGCCTGAAATGGCCGCGGCGGCGTACGATGTGGCAGCA

TTACACCTTAGAGGACGTGGGGCCCAGCTAAATTTTCCTGAAATGGTGGATAGTTTGCCC

CGGCCGGTGAGCTCTAGCACTGAGGATGTGCAAATGGCAGCACAGGAGGCGGCTTTGCTG

TTTCGGAGACCGATAAAATGTTCGGAGGGAGTAAGTGGTAGCTCTAGTGGTGGTGGTTTA

GGTCCCGTTAGGGTTGGGTTGTCCCCGAGCCAGATTCAAGCTATAAATGAGGCACCACTG

GACTCACCAAAAATGTGGATGGAGTTAGCCGGCGCTCGTCTGTGGAATGAGCCTCCGGCT

ATGGGTGGTGATATTGATGTTGAGCTCAGTGATGAGTGGGGTGAAATGGAGCATGAACAT

GACTCCATTTGGGATTATTAA

>EVM0029243

ATGGTAAAACCTGGAACGTCATCTTCGTCTATGTCATCCGAGCAACAGGCTGATAGAACC

CACGAGCCCAAGTACACGGGTGTTCGAAAGAGAAAATGGGGTAGGTGGGTGTCTGAAATC

AGGCTACCAAATAGCCGGGAGAGGATTTGGTTAGGATCCTATGACACGCCTGAGAAGGCG

GCGCGTGCGTTTGACGCTGCCCTGTACTGCCTACGTGGTAGTGGCGCCAAGTTCAATTTC

CCGGACAATCCACCGGACATTGTCGGCGGGCGGTCACTCAGCCCGCAGGAGGTGCAGGAA

GTTGCTGCCAGGTTTGCTAATGAAGAGCCAGCGCTGTCCAGTGCTGCTATGGGAGGTGAG

TCGTCGTCACATGTGGAGAACTACACGTCGTCATCTTCTGAATGCGGGGCTGGGCAAATG

GATAGTGATACAATAGATTGGTCGTTTTTGAACATGTTGGATTCTAATGAGGGTGCATCC

GATTTTGGACTCTATCACGGGCTGGACCATATGGGCGGTGATTATTATACCCCGCCGCCG

CCGCCATCTGATTATAACGAGGATGATAACAATGGAGACGAAACTTACTCTCACCAGTCA

TTTCTGTGGAACTTTTGA

>EVM0042683

ATGGATTTTACTCAGTCAACAAAAACCAACTCCACACCCTCTCCTTCAAAGACCAAAAGA

AAGCAGCAGCAGCAGAAACACCAGAATCAACAAGAACAAAATGAAGTACGGTTTTTGGGG

GTAAGAAGAAGGCCATGGGGCAGATATGCTGCAGAAATAAGAGACCCTTCAACAAAAGAG

AGGCATTGGCTTGGTACTTTTGACACCGCTGAGGAGGCTGCCTTGGCTTATGACCGTGCA

GCCAGGTCCATGCGAGGCTCCAAAGCCCGCACCAACTTTGTCTACTCAGACATGCCTCCC

GCCTCCTCCGTCACCTCCATTATCTCACCAGACGAATCCCAACATGAAATTCCAGCCCTC

TTTGCTCCACCTCCACAAAACCATGTTCATCAAAACGACACCAACTGCCAGCAGCTCTAC

TTCTCTCAGGATCAGTACCCCTTCCATGCTTATGGTAACAGTACCAGCAACCTGTTAGCA

GGTGGAGAGGGTTGGGTTCAGGGCTTTGAAAGTGGTGCTGCTGATGGCCCCGGAGGGTCG

TATGAGCCCAATAGTGCTGGCAGCTTTGATGTCACTACTGAGCCCAGCTACTTCTCCAGT

AAGGACAATATCGAGCTCCCACCCTTGCCTCCAGATGTCAACTCCAGTTGCTATGGGCCT

GATATGGATCATGGGTTCTGGAATGACGCAGGATTTTTTGGGTTTCAAGAGGAACAGAAA

AATAACGATAATGGGCTTGCAATTAGTGGATCAAGCTTGGGTTTTGATTCAAGTGACCTC

AGCCAGCATGGCTCGTTATTTGAGATTGTGCCATCGGTATCGGATTCGGTCGCAGACGGG

CTCGATCTGGGATCGTCATCTACCCTTTACTTTTAA

>EVM0017998

ATGTCTGCCATGGTTTCAGCTCTCACTCAGGTCATGGGTAACCCAACCTCGGTGCAATCC

ACCCAATCTGCTCCGGAGCAATCCGTCGTCAAAGACGAACCTGACCTGTCTCGACCAGTG

CAAGATCAAGAAAATACAAGGAGAAGGCATTATAGAGGAGTGAGACAGAGACCCTGGGGT

AAATGGGCAGCCGAGATACGTGATCCTAAAAAGGCAGCCCGAGTCTGGCTTGGTACTTTT

GACACTGCTGAGGATGCAGCCGTTGCATATGATAAAGCAGCACTTAAGTTCAAAGGCACC

AAGGCTAAGCTTAATTTCCCCGAAAGAGTTCAAGGAAGAACCGAGTTCAGCTACTTCATG

GGTTCTGGGGCTTCCAGCAATGTCTTGACTGAACAAAGTCCCCGGCCAGTCGCTCCTCCT

CCTCCTCCACCTCCCCCTTCATCATTTGCACCAGATACTTACCCAGATTTGCTTCAGTAT

GCACAAATCCTTTCTAGCAATGATGCCAATTTCCCGTATTACACTTCAAACCTCTTTAAT

CAACAACCTTTTGCTCCCGATTTTTCATCAAGCTTTTTATCACCGCAACAGCTACAGCAA

CAACAAGATCTGATGAGATTCTCGTCAGGGTTCGATACCTCCTTTAGCTCTGATCAACAG

GAGCATGGGAAGGACTCGAGTAATCCTAGCGAGTAA

>EVM0000535

ATGGCACCGAGAGAGAAGACGGCGTCCGTTAAAGTTAACGGCGGCCCATGTGTTGGTGCT

GGTGGAGGAGGAGGCAAGGAGAAAGAGGTGCATTTTAGAGGTGTGAGAAAGAGGCCATGG

GGAAGATTCGCCGCCGAGATAAGAGACCCTAGTAAGAAGAGCCGGGTTTGGCTTGGCACT

TTCGACACCGCAGAGGAAGCCGCACGTGCCTACGATGCTGCCGCTCGTGAGTTCCGTGGC

TCTAAGGCGAAAACCAACTTTGCTTACCCATCATACGAGAATGCTAGGGAAAACACGATC

TCTGTGGATAGAAATAACAAGAACAACAGTTGTGGTGTTGTCGGTAATAGTAATAACAAC

CAGAGTCCAAGTCAAAGCAGTACCGTGGAGTTTTCAAGCACCGATACACCGTTGGATCTT

AATCTCGGTCCTGCTGTATCCACCGTGAGATTGCCGTTTCAGTCGATGGTGATGAATCGT

CAGCAGGTGATTTATTTTGATCCCGTGAAGAGTCAGTGCCAAAAGATGGTTTTTGATGGC

GGTCATCATCATCCTAACCAGCCGATGAGTTTTTCTTGTGATTCTGATTCATCCTCTGTC

GTCTTTGATTTGAATAATCAAGATATCAAGACGCCGAGAAGTTTTGTTGATCTTGATCTT

AACTTTCCTCCCCAACCGGAGATTGCATGA

>EVM0044386

ATGTGTGGAGGTGCCATCATTTCCGATATCGTTGCCGTTAAACGTGGCCGGATATCGACC

ACTGACGATCTCTGGCTTGAACTTGACACCCTATCTGAATTTTTTGGATTTGATCATGGT

TCTATAAACAACATTAATAATGGTAGCAAAAAGGAAAACCTATCAAATCTCAAGCTCCCT

CCAAAGCCAAGCCAGCTCAACCAAGTGATAACAGAGAAAGTTGAGAAGCCAAGCCAAGCA

GCAGAGCAAGAAGCTGGTAACAAGAAGGCTCAGAGAACCAGAAAGAACGTGTACAGAGGA

ATAAGGCAAAGGCCATGGGGGAAATGGGCAGCAGAAATAAGAGACCCACACAAAGGTGTT

CGAGTTTGGCTGGGCACTTACAACACAGCCGAGGAAGCCGCTAAAGCTTATGATGAAGCC

GCGAAGCGCATCCGTGGTGATAAAGCCAAACTCAACTTCCCCGCCCAGCCACCACCCTAT

TCAGAGGCGGCGGCGCCACCCACCAAGAAACGTTGCATCTCGGGTCCTGAAACGGCTTCT

GTGGCTAGTTTTGACACCGCCCCACCTCGAGAGTCATATTATGGGTATCAAAACGAAGAC

TATGATCTGACCGAGAAAATCTTGAACTTGGAGTCGTTTCTAGGCCTGGAGCCGAAGCAG

ATGGCGGACCAGCTGAGTTGTCATTGTGACCCTGTGGACCTTTGGATGCTCGATGATCTT

GTGACACATCATCAGTATCTACGAGAGCTTAATTATTAG

>EVM0055399

ATGGAAGAAGCACTAAGAAGGCTCAACGGAATGCCGAACCATGTACCGGAGTCCAACCCG

CCCGAGACCATAATCGTCGACCTCCAGAAGAAGTCAGCACCCACCACCACAAGCAAAAGG

TCACTGAAAGAAAGTGGTGGAAATAGCGGAAACATGAGGTACCGTGGAGTCCGCAGGAGG

CCATGGGGTCGTTATGCGGCCGAGATAAGGGATCCTCAGTCTAAGGAACGGCGGTGGCTC

GGCACATTTGACACCGCTGAGGAGGCTGCCTGTGCCTATGATTATGCTGCTCGAGTTATG

CGTGGTATCAAGGCACGTACTAATTTTGTGTACCCTGAGACATATTCTGCAACAACTGAC

TCTCAGAATTTGCTCCCTCCATTTACTTTCTCAAAATCATCCCACCATCAGCCCTCTCGC

CAGTGCAATACAGCACCATCTGCAAATTGGTCTTCTCCTGGACACTCTCTCGTGGGTGAC

TTCTCAGCTGGTTGGTCTGCGCCACAGAGGAGTTCTAGTGGCAGTGCCGCTAATACTTCT

TCTCTGAACATGCTTTTTCTTCGTGACTTCGTTGACTCTTCTGGTTCGTCGTCTCAAAAC

CATGCTCAACCTTTGTATCATGAGCACTTTCCCTGCATTAATGGGTCCTCCTATTCTGCT

CTTAGCACTTACTCAGGTGGCTCTTTGGCGAATCCTTCTAGTAATCCCAGCAGTACTGGT

ATATATAATTTATCTGGAAATTTGGCAAACTCAACTGTCACTAGTCTACCCCACATGGAG

TTTAATCAGAATTATACTCCTGTGGCCCTGAATAGGCCCACCTCAAAAGCTGAAGATTTG

GGTTTCTTTCCGCAAGAACCATCCGGTTCCGGTTTGTTGCAAGAGATTATTCAAGGGTTT

TTGCCCAAGCACTCCACTGAAACGACTGACTTTTCAAAAAGCTCTGGAGAGTCGATGGTT

ACACCATCAGCTGAAATAATATTTGGTGGCCAGTCGCTAGATGGATCAAGGAGCACGAAG

CACTTAGTTGAAAACGACCACCATGGTGCTGCTTATCTTGATCACGAAGGAATAAAGCCA

TCAGCACAGGCCGGGGGCTATTACCGTACCGGGGTTAGCTGCCATGAACAAGTAACGTAC

AATAACGAGATGTCACTGAATCTTCCGGTGGGTCAGGACTCATTTCTGGAAGACATTTTT

CAGTACCCAGATCTTATGAGTGCCTTCGCAGCTAGGTTCAATAATGTTTGA

>EVM0049532

ATGGACGAGGACAAAACAAAGAGCAAGGATGTAGGAGGAGAGGTCAAGTACAGAGGTGTC

CGGAGGAGGCCATGGGGAAAATTCGCTGCCGAGATACGTGATTCCACCCGGCACGGGGCG

AGGGTGTGGCTGGGGACTTTTAACACGGCAGAGGAGGCAGCAAGAGCCTACGACAGAGCA

GCTTATGCAATGAGAGGGCACTTGGCCGTTCTCAATTTTCCTAATGAGTACCCAAACATG

GCCGGTAATGCTAGCGTTGGTTCCACAAGTTCTACTCCTTTTTCCTCTTCAGGTTATCCA

GGTTCTTCCTCCTCTACATCATCAATGCAGCAAGAAGTTTTTGAGTTCGAGTGCTTGGAT

GATAAGTTGCTTGAGGAGCTGCTTGAGCAAGAAGAGAAAAGGAATAAGAAAAGGTAA

>EVM0002921

ATGGAAGAGACTAACAAAGGGGGGGGAAAGGAGTTGAAAGGGAGAGAAGAGGTTCGATAC

CGGGGTGTTCGTAGGAGACCCTGGGGAAAATTTGCTGCGGAAATACGAGACCCATCAAGG

CAGGGAGCAAGGTTATGGTTAGGGACATTCGATACGGCAGAGGATGCAGCCCGTGCTTAT

GATAGAGCTGCATTTAGCATGAGAGGTCATTTTGCAATCCTCAACTTTCCTAATGATTAT

CTCTCTAATGCGACGGGGACTACTCCTCGTCGTCCACAAGTTTCATCATCTAGTAGTGAT

GTTGGTGCAAGCGAGAGTTTTGAGAGAGGAAGCTCATCAAGTGCCGGACAAGGAAAGCAA

GTTATCGAGTTTGAGTACTTGGATGATAAGATTTTGGAGGAGCTTCTTGAAACAGAAGAG

GAGAAGAAGAAACGGCAGCAAGATTGA

>EVM0025231

ATGGATGCCTCCATCTTTCACCCTGCAAATTCTGATTTCTCACCGGAATCTTCTTTTGGA

TCACCGGACTCTTTTTACAGCCAACCTTTTAACCAAAGCTCTCTCCCTTCAATGAAAATG

ACTCCGATGAAATCCACTTTCTATAATGGGATTGTCAAGGAAGAGGAGGTTAGTTCAGTT

GCCAGAAAAAAACCCGACAAGGAAAAGTCCTACAGGGGCGTCAGGAGGCGGCCATGGGGC

AAATTCGCGGCGGAGATACGGGATTCCACCAGGAATGGGAGGAGGGTATGGCTAGGCACG

TTTGACAGTGCAGAAGCAGCTGCTCTGGCTTATGACCAGGCAGCTTTTTCGATGAGAGGA

CCTGGGACTATACTAAATTTCCCAGTTGAAAGAGTGAGCGAGTCACTGAAGGACATGAAG

TGCAGTCATCATGAGGATGGCTGCTCGCCGGTGGTGGCTCTGAAGAGAAAGTACTCCATG

AAAAGAAAACTGGGAAGCAGGGGAAAGAAAGAGAGGGACGTGAGGATAGAGAATGTACTG

GTTCTGGAAGATCCAGGTGCTGATTATTTCGATCAAACATGCTGA

>EVM0038570

ATGGAGGATCAGTTTCCTAGATTGGAAACCCTTATGCACAAGGAATTGGCAGCTTATAGT

AGCTTTCATGGAATGGCAACAGGATCAAGATATTTTGTTGATCCCACAATTTGGGGTGCT

CTAGCTGGGGCTACTTGTCATGTAGATGATGGAAATAATGGCACGAAATCGCCATCACAA

TCGTCTCCCGAAAGATTGTTTTCGAGTTCGGAATCGAGTTCTTCGGCAAACGATGTGGTT

TCAGTTACCAATTTGGTTGGTAACATCCCTGGATTTGGTGAACAGGATGTTTCCAAACAA

AATCATGAAAGTTGTTTTATGTCAGGATTGTATTCTATCAATTCTCCTGGAGGTTCATCT

TCTGACCATATTGTCAAAGAAAGCTCCATTAATCCTGTGAATTTCTTGGAATTGTTTCCA

GCACTACATAACCAAGCCCTAGTATCTGAACCTCCCTCTCCATATTCCTTATCAAACACA

TCTAAATTCCCAAATTTGACCTTGTTTTTGCAGGAACCAGCAGCTCTCATCCAAACAAGT

TGTCAAACACCTTGGCCAGGATCAAAACCCAAACAAGCTGTCCATCAACTTGGCAAGGAT

CACAGATGTGAGCCTATGTCATTTTTTCCAAACACTTCATTCTCCATGCCTCAACTAGGC

CAAATTCATTGCCAACCAAGCAATGAATGGCTCAAAATAAATCAAGCTTTGGCAAACTAT

TCTACCAAAGGATTTAATGACCATTGGCTTAGAACTACGCCCACACAGCCGATGAAATAC

ACCAGTCGAAGAATATTGCAGAACCACCATCAAAAGCCTTCATTTTTATCAGCTTCCACA

TCGCCGGGAAAGGTTTTTAGAGGAGTGAGGCAAAGGCACTGGGGCAAATGGGTCGCAGAG

ATTAGATTACCGCGAAACCGGACGAGGGTTTGGCTTGGGACTTTCAACACAGCAGAAGAA

GCTGCAATTGCTTATGACACAGCAGCTTACATGTTACGAGGAGATCATGCGCATTTGAAT

TTCCCAGATCATAAGCTTCAACTCAAGGCTAATTCATTGAACGGAACCACAGCTGCCCTT

CTTGAAGCAAAGTTACGAGCAATATCACAAGGCAACACCATTTCTGGTCAAAAGAAGCAC

AACAATGATCCACCGCCACCATCACCAGATAAGATTCTACATGACATCAATTCTACAAAA

CCGAAAGGTTTGGTCCAAAACACAACGAGAAAAGAGTTGAAATTTGATTTGGAAGGCAAA

ACTGGTTGCCACGAACTGATTCATGAGAACCAGAAAGGTCAAGAGGTCGTAGCATCTGAT

GTAGAAGCTGTTTTGTTAAGTAGAATGCCTTCCTTGGACATGGACATGATCTGGGATGCT

CTTTTAGTCCCTGATTCATGA

>EVM0042411

ATGAGTGATCAGCAGCAGCTAAAGCTGGAGACAGAAAGTAGCTCAACAAACGCATTCTCA

CCACCCTCACCATCTTCACCATCTTCTAACTCGACCCCGTCAAAGAAACATCAACAAGAC

CAAGATTTTTCTCCTTGTAAGAAAATAACAAGAATGAGAGACTCTAGCAAGCATCCAATC

TATCGAGGAGTTCGGATGAGAAACTGGGGCAAATGGGTGTCTGAAATTCGAGAGCCTCGA

AAAAAGTCGCGCATTTGGCTGGGTACTTTCCCTACCCCAGAAAAGGCGGCGCGTGCGCAC

GATGCCGCTGCTTTAAGCATCAAAGGAAACTCAGCTATTCTTAATTTCCCTGAACTCGCT

AACTTCTTACCCCGTCCAGCTTCACTTGCTCCTCGTGACGTTCAAGCAGCCGCTGCTAAA

GCTGCCCAAATGGACACTTTTGACCGAAAATCTGAAACAACCACCACCACAACAGCGTCT

TCATCGTCTTCCTGTACATCTTCTTTGACATCCCTTGTTTCTTTAATGGACTTGTCTCAA

GATGAAGAGTTGAGCGAGATTGTTGAGCTGCCAAGTCTCGAGACTAGTTATGACGAGTTA

AACAATGATTTTGTTTACTCTGACTCTGTTTATGGGTGGATGTATCCTCCACCGTGGATG

CAAAGCGTGGAGAGTCTCTGTTTCAGCGGCGGCGGCGGGTATGCTTGTGAAGATCTTACA

CTGCCGAATGAGAGTACTGTTTTATGGAATTATTAG

>EVM0001696

ATGAATGAAAAAAACACTAGCTCAAGCAATTCGATATGTAATTCACCAGCACCACCGCCA

TCATCTTTGGCCACTACCATTACCAATAAAAAGGCCAGAGACTGCAGCGAGCACCCAGTT

TATACGGGTGTTAGAAGGCGAGCCTGGGGCAAATGGGTGTCCGAAATCCGCCAACCTCGT

AAGAAATCACGTATTTGGCTCGGAACATTCCCTACGCCAGAAATGGCAGCGCGTGCCCAC

GATGTGGCAGCTCTGAGTATCAAAGGTGACTCTGCCATATTAAACTTTCCAGAGCTTGCT

GACTCATTACCACGCCCAGTGTCGGTTATGCCACGTGACATTCAGTCAGCCGCGGCGAAA

GCTGCTGCCATGGTCGAGTTTAACTCATCATCACCATCCTCATCACTGTCGTCGTCATCG

GTAACGGTGTCTGAAGATGTGGAAAAATCCGAGGAAGAGTACTTGAGTGAAATTGTGGAG

TTACCGAATATAGAAGGGAGCTTTGATTCTGCTGATCAGTCACAAGCTGAGTTTATGTTG

TTTGACTCGGTCGATGGCCATCGATGGGTGTATCCACCTTTAGACTTATGTGGAGACTTT

TCTGATTACTTGTTGGGCCTGGAGAACTTGATTTGA

>EVM0031168

ATGCTTTGCCACTCGATAAAAAAGAAACCCGTCATGAAAATGGATCGTCCAGTAAAGTTC

ACTGAGCATAGAAACCAAACCAAACTTACCACCCCATTTTTGTCAAACCCGGTCCAATAC

TCAATACCACCAAAGATTGTACGTATATCAGTAACTGACACTTGCGCAACTGACTCTTCA

AGTGACGAAGAAAACGAGGTTTCAAGAATCTCTTCTCGTAAACAGCATGTCACAAGAGTC

AAAAAGTTTGTCAATGAAATTACGATAGAGCCAACCGTACCACCATCAACAACCGCTGGC

TGTGTCGCAGGCGACATCGGTTTGAGGACCAGACCGTCGAGGAGTGCTAGGAAGAAGGTT

GGCATTAATGTGTCTCAGGGGAGACGTTTGCCGGCGACGGCGGTGGCGGGGAAGAAGTTT

AGAGGGGTGAGGCAAAGGCCTTGGGGGAAATGGGCGGCTGAGATTAGAGATCCTTTGAGA

CGTGTGCGGCTGTGGCTAGGTACGTACGATACTGCTGAAGAAGCTGCGATGGTTTATGAC

AATGCGGCTATTCAGTTGCGTGGAGCTGACGCGCTCACTAACTTCGTCACCCCTCCTGCG

AGATGCTCACCGGTGGCAGCCACCTCCGGCTACATGTCCGGTGGTGAGTCAAATAGTAAC

CGTAATATGAACATTGACAATGTTGGTGTTAGCTCTCCAATTTCAGTGCTCCGGTTTAGT

GAAGAAGCCGAGTCTCAAAGTGTCGGTTCAAGCAGAGAAATCCAAGAGACCGGAAATGAA

GTCCGGGAAGTAAAAGAGGACTCCTATGTATCGGAAAACGTTTCCGACTTTTCCGAGCAT

AACTCCTCTATCGACAGTCTGTATCCTCCACCAACCGACATTTATGAATTTAGGAGTTCA

GTGAGGGGTATTTTTGAGGAAACAAGTTTTGCTGATGGTGGGTTTTTAAAAGATGATGAT

TTTAGGTACATGGATCTGGATTTCGGGTTCGGGTTCGGGTTATCTAGCTGGAAAGTTCAA

GACCATTTTCAAGACATTGGTGATTTGTTCGGGTCGGATTCACTTATAGTCATTTGA

>EVM0034491

ATGTGTGGAGGTGCCATTATTTCCGATTTTGTTGCTGTTAAGCATGGCCGGAGATTGACA

GTTGAGGATCTCTGGGAGGATCTCCGGCCTGAACTTGACCTCCTCTCTGAATATCTTAGA

TTTGATCTTCACAATAATGGTAGAAAAAAAGACCCATCAAAACTCTTGCTCCCTCAAAAG

CCAAGCCACACCAACCAAGTCCAGCAAGTGATAACAGAGAAAGTTGAGAAGCCAAGTCAT

GCCACAGATAAAGAAAATGGTAACAAGAAGGCCCAGAGAACCAGAAAGAGTGTGTACAGA

GGAATAAGGCAAAGGTCATGGGGCAAATGGGCATCTGAAATAAGAGACCCACACAAAGGT

GCTAGAGTTTGGCTTGGCACTTACAATACAGCCGAGGAAGCCGCCAAAGCCTATGATGAA

GCCGCCAAACGCATCCGAGGTGATAAGGCCAAGCTCAACTTCCCTTCTCAGACACCGCCA

ACTCCGGAGGCGCCACCACCTGCCAAGAAGCGTTGCGTCTTTGCTCCTGAAACAGCTTCT

GTTGCTAGTTCTTACACAAAACCACCACAAGAGCCATATTTTGGGTATCAAAATGAAGAT

TATGAGCTGAAAGAGCAAGTCTTGACGTTGGAGTCGTTTCTTGGACTGGAACCTGACCAG

ATGGCGGCTCAGCTGAGGGGAAATGGCGGTGATAATCGTGAACCTGGGGACCTTTGGATG

CTTGATGATCTTGTGACCCATCATCAATATCATCGTCAGCTTAATTATTTGTAA

>EVM0046023

ATGGCCAGCTCCAAAACCTTTGATAAACCCTTGGAAGGGTATGATGCCCTTAACATTCAT

GCAGGCTTTGCTCTCCTGCAGAGAAACACGACCCCGCCTCAAACTGGTGAGAGAAGAGGC

AGAAGAAAGCAAGCGGAACCAGGAAGGTTTCTTGGAGTTAGGAGGAGACCTTGGGGGAGA

TATGCTGCTGAAATTAGAGACCCAACAACAAAAGAAAGGCACTGGCTTGGCACCTTTGAC

ACTGCTCAAGAAGCTGCTCTAGCTTATGATAGGGCTGCTCTTTCCATGAAAGGCACCCAA

GCAAGAACCAACTTCACTTACTCTGATAACACAACCTTTCACTCCCTTCTCACTCCATTT

GACATTCAAGCCTTGTTGCCACAATCACAGCTTCTCACTGACACTCAGATTATACAGCCA

ACCAATCAGAACAGTCCCGTTAAGCCTGAGATTTGTCAAAATAAGACCCCAAGTAGCAAT

ACATATGCTGAATCTTCTGGTGGATCAGCTCATCAAGCTAACGATTTCTTTTTCTCGAAT

GAGGATTCTAACTCAGGTTATCTGGGCTGCATTGTTCCTGATAACTGCTTGAGGCCACCG

CCCTCAAGCCCCACTAGTAAAGCCTCCAGAGCCAGCACATCCAATGCTTCAAATGATCAA

AACTTTTGCTTCATGAACACAACCTACCTTGAAGATCATTCTCAATGTAACAAGTACGCA

CCTCCTCTCGATATCACAAATTTGCCGACCAAGGCATACAGTCCTGTAAACTGTCCTGGC

TTTGATGAACTCAACAATGGATTTTGGGGGGAACAACCGTCGTGGGATGGCAATTCCGGT

GACCTCTCAGCACTGATGAACAACCCATTTATGATGGAAAATGTATGCATGGATACTTCG

CATAGCATCGACAATCCTACTTGTGGCTTAATGCCTCAAGCTACTTCTTCAGTATCCAGC

CCTTCGTTATCATATCCAGCTTATGGTGATGCAGTTAACTTTGGATATTCACTCTTTTGA

>EVM0023162

ATGGCACCCAAGAAGCTTAATGCTAGCAACAGTGGCTCTTTCAAGAAAGCAAGTGGGGAC

CAAGACACGAAAGAGATACATTACAGGGGAGTGAGGAAGAGGCCGTGGGGGAGGTATGCT

GCCGAGATAAGGGATCCCGGGAAGAGAAGCCGGGTTTGGCTGGGTACTTTTGATACGGCG

GAGGAGGCTGCTAGAGCCTATGATAAAGCGGCGCGTGAGTATCGGGGTGCTAAGGCGAAG

ACCAACTTTCCTTTTGCTGAGGGGGTGGTTAATTATGACGATGATAAGCAGAGTTCTAGC

CAGAGCAGCACCGTGGAATCTTCGAGCTCTCCGGTTGTTTCTGCTGTGGTGACCCGCCAG

GCTGGTGGAGTGGGCGGGGTTGTGGGGATGGGAGGGTTTCCTTTTGTGTACCAGCAGCAG

AATGTTAACGTTGTTGCTCCTGTCTGGTTTTTTGATAGCATGAGGCCTGAGTTTGCGACT

CAGCGAATCCCCGTCCGGTTTGACCAGGTGGGTCTTGAAAGTGCTGGTTGTGCCCAGAGT

GACTCGGGTTCATCTCTGGTTGATCCTCAGCCAAGGAGAACAGTTTTCGATCTTGATCTT

AACTTGCCTCCACCGTTGGATGCTTGA

>EVM0008654

ATGGCAGCAGCGATAGATATCTACAGCACAACAATACCAGTTTTTTCGGATCCTTATAGA

GGAGAACTCATGAAAGCACTCGAACCTTTTATGAAAAGTGCTTCATCTCCTTCGACTTCT

TCTCATTCTTTCTCTTCCTACCCTTGTAGCTATGACAACAGTAGCTTCCCAGTCACTTCA

CATCCCAACTTAGACCTTAACTCTTACACTCCAACGAGCACCCAGATGTTTTCCAAGGGG

TTCTCGGGTTATGACCAAATGAGTTTTGAGCAAAAAGGTCCAATTGGGCTTAATCACCTT

ACCCCCTCACAAATCCTTCAAATCCAAGCCAAAATCCACCTCCAACAACGGCAGCAGCAG

CAACAAATGGCAAATCATTCTCCCTCATTGCAGTTAGTCCATAACCAAAGGACTAGCAAC

TTCTTGGCTCCCAAACCTGTCCCTATGAAACAACAGTCTGCTCCTCCTCAAAAGCCAACG

AAGCTTTATAGAGGAGTGAGGCAGAGACATTGGGGAAAATGGGTTGCTGAGATTAGACTT

CCAAAGAACAGAACAAGGCTCTGGCTTGGCACCTTTGACACAGCCGAGGAGGCAGCTTTG

GCTTATGATAAGGCAGCTTATAAGCTTAGAGGAGAATTTGCCAGGCTCAACTTTCCACAT

CTTCGCCACCAAGGAGCTCATGTGTCTGGTGAGTTTGGTGACAACAAGCCTCTCCACTCC

TCTGTTGATGCGAAGTTGCAAGCAATTTGTCAGAGCTTGGGCTTGCAAAAACAGGGGGAA

ACAGAGGAACCTTGTTCTGTTGCTGATTCAAAGAAGACCGTTTCAGCTCCTTTGCAGGCA

AAAATCGAAGATGACATTTCTTTGAAGGGTGAATTGAAAAGGGAGTTTGAGAATTTTGGA

GGTGAGGAATTTAAAGCGGAGATCCCATCACCCTCACCAGCTTCATCTGATGAGTCATTG

GCCGGTTCTTCTTCACCAGAATCTGAGATTTCTTTCTTTTTCTCTGATTCCTTACGGTGT

GACGAGTTTGAGAATTTTGGTTTGGAGAAGTATCCTTCAGTTGAGATTGACTGGTCATCT

ATCTAA

>EVM0023217

ATGTGCCTATTGAAGGTGGCGAACCAAAGAGGAGACCCGGGGGAGTACACCAGGTACCCT

GCAACAGGTAGCTATGACAGCCAGGGAGAGAGATCATACACAGCGCCACAAATATTTTCT

GAATCGAATCAACCGATAGAGCAGCAGGTGGAGGCGACACAATCACCGCATATGTTCATG

GGGTACAGCAGTTCATCGGGGATGTCTGCGATGGTATCAGAACTTACACATGTGGTGTCA

GGGCAGAGAGGAAGTGCTAGTGATTGGGGATCATATGGGGCTTTAGGTCTAGGAGGTGCA

ACAATAACATCGAATTTTGGTCAGGGACCACCTGGTTCTAACACGAGCACTCCTGCTTCT

CCACCTCTATCTGCCTATTCTTCTACTTCTGGTTCTGGTTCTGGTTTGTGGATTGGCCAA

AAGAGAGGGAGAGAAGAAGAGGCTGGTGCAGCAGCGCAACTGATGGAGTCTTTGCCAAGG

GTTTATAGAGGGTTTAATGATTTAAGAAGTTCACAAGGAGATTCATCATCATCCACTGCA

ACTGCAACCGAAGAAGTGTCTGCTTCAACTACAGTAATCCCCACCACAACCACACCCTCC

ACCACAGCAACACCATCAAGTGAAACTGCGTCACTGGAAGAAACTGGAGAGCAAAGAAGA

AGATACAGAGGAGTTAGGCAGAGACCATGGGGTAAATGGGCAGCCGAGATACGTGATCCG

CACAAAGCAGCAAGAGTCTGGCTAGGCACATTTGATACTGCAGAGGCTGCAGCTAGAGCC

TATGATGAGGCTGCTTTAAGATTCAGAGGAAATAGAGCTAAACTTAACTTCCCTGAAAAT

GTTAGATTGCTGCCAGCTCAAATGCAAAATGTTACCGCTTCTCAAGGTCCCATCTCTCAC

TCACAATTATCTTCTCATAGCCAGTTACAGCCAATCTCATCTCCGAGGCAACAGGCACAG

CGGCCGCAGGCGCCAGCACCTGCATTGTTTCAGTCTCAAGCTGATATCATGAGGGACTAC

TGGGAGTACTCCCAGCTGTTGCAGGGTTCTGGGGACTTTCATGGACAACAACGACAACAG

CCACCACCTTCAAGTTTGATACAGCATATGTTTTATAATCCCCATGTGGCTTCTCTACAA

TCACCAGCTTTGTCTTCATTGTCTTCATCTTCTTCAGGTTCTTCACTTGCAGCGATATCT

TCCGGTTCATTACCATCAACATTTTCTCCCTCTGCCGCTTCTTCCTTCCCTCTGCTTTTT

GCCGGTCAACAGTTGGGTTATTTTCGGCCACCACAAAACCAGAATCAATCTAGTGGTTCT

GATTTTCCGGTGCCCCCATGGACAGATTCTAGTCACAATCCTTCCTCTAATGGTTAA

>EVM0022031

ATGGATTCTTCATATTCTCACTACCAGAACTCACAATTCTCCCCGGAATCTTTTTCCTCC

TTTAACTCCATCGATTCTTTCTCGTGTAACACACAGAGCTTCTACAGCCAGCCCCTTCCT

TTCAACGAGAACGATTCCCAAGAAATGCTTCTCTTAGGAGTCCTCAACGAGAACCCCATC

AACTCTTTCGACACCGTATCATCCACCAATACTAGCTACGATGAGGTGAGTTCAAGAGCT

GATTACCAGGGGGAACAGCCCCGAGAAATTGCTTACAGAGGGGTCAGAAGGCGGCCATGG

GGAAAGTATGCTGCCGAGATACGGGATTCGACGAGAAATGGCGTGAGAGTTTGGCTGGGA

ACATTCGACACCGCCGAGGCGGCGGCCTTAGCTTATGATCAAGCTGCATTTGCAATGAGA

GGTTCGATGGCAGTACTGAATTTTCCTGCCCAGAAAGTTTACGAGTCACTCAAAGAAATG

GGCTATGGCTTCCAGGAAGGGCAATCACCGATTCTAGCGATGAAAAAGAAACATATAATG

AACAGGAAAGCCGAGAGTAGGAAGAAGAAGGAGAGAGAAAGCAGAACGGAAATGGAAAAT

GTGGTGGTGCTGGAAGATTTAGGGGCCGATTACTTGGAAGAACTGCTAACCATTTCCGAG

AGTGCTAGTCCTTGGTGA

>EVM0013555

ATGGTGAGAGAAAGAAGGGATAGAGGAGATGATAACGGGCGTTACAAGGGAGTGAGGAGG

AGAAACAGAAATAGAATATGGTTAGGTTCGTACAATACTGCAGATGAAGCGGCAAGAGCA

TACGATGCTGCTGTTTTATGCCTACGAGGGCCTTCGGCGACGTTTAATTTTCCAACGAAT

GTGCCGGAAATTCCTGCTACGACGGAGGTATTGTCTCCCACGCAGATAAGGGAGGTTGCT

TCTAGGCATGCAAGAAGGAGGGGTGCTATGGAGCTTGCAGAGAGGATTGTGGGACCTGGG

TTGTGTGAAGTGCCATCTGGGATGAGTGGAGAGGCTTACTTGGGTGGTGGAGAGAATATG

GCGGAATGCATGGATGGAATTTTTCGCGGGGAATACTATCAAACACCTGGTGTGTGGACA

GATTAA

>EVM0045980

ATGGTACAATCAAAGAAATTCAGAGGCGTCAGGCAACGCCAGTGGGGGTCCTGGGTGTCT

GAAATTCGCCACCCGTTACTGAAGAGGAGGGTGTGGCTGGGGACATTTGAGACGGCTGAG

GCAGCCGCAAGGGCTTACGATCAAGCGGCTATATTGATGAACGGACAGAATGCCAAGACC

AATTTTCCGGCGAGTCACCTCAATCAAGACATGAATCTCGGCGAAGATAACAACTCTCCC

TTGCCTGCCAAGGAACTGGCTGAGCTACTGAACTCGAAGCTGAGAAAGTGTTGCGGGAAA

GATCCTTCTCCTTCACTCACTTGCCTGAGGCTTGATAATGACAACTCTCGTATCGGTGTG

TGGCAAAAGAAAGCTGGTTCTCGTTCAAGTTCAGACTGGGTCATGAAGGTGGAGCTTGGA

AATTACAATAAGAAGACAGTGTCGCCGTCGAAGGCTGAGATTGAGCCGGAAAAAGGTACG

GAAGAGGAGGATAAAATTGCTATGCAGATGATAGAAGAGCTACTTAATTGGAATTGA

>EVM0019845

ATGGCCAGACCACAACAGCGATATCGAGGGGTTCGACAAAGGCATTGGGGATCATGGGTG

TCTGAAATTCGCCACCCTCTATTGAAGACTAGAATTTGGCTAGGCACGTTTGAAACTGCA

GAGGATGCGGCAAGAGCCTATGATGAAGCTGCAAGGCTTATGTGTGGACCAAGGGCAAGG

ACCAACTTCCCTTATAACCCAAATGCATCGCAGTCAGCTTCGTCAAAACTTCTTTCAGCA

ACTTTGACAGCAAAGTTGCATAGGTGCTATATGGCTTCATTGCAAGTAACTAAACAAACA

TCAATTCAAGAACAAAAACAGCAGAAACAAAAGGCACCAACTTCGCTTGTCAACATCCCT

ACCAATGGCATTGTCATAAAAAGTGAGGAACTTGATGCTTCGTTGCCAGAGAAAAGTCCA

TTGCAAGTTCAAGAGGCAGAGGCAAACTGGGTGTACAAGAAAGTTCAAGTTGACAACAAC

CAGCAGTTCATCAAGCCTCTTGAAGATCACCACATTGAGCAAATGATTGAAGAGTTGCTA

GATTACGGGTCTATTGAGTTATGTTCTGGGGTAGCAACTCAGTAA

>EVM0039499

ATGTGTGGCGGTGCTATTTTATCTGAAATCATCCCTCGTAACCGCGCCCGCCGCGAAGCA

GCTTCAGAGTTCTGGCCAAACGCATCCTTCAATAAACTCAGTCCTTTTGATTCCTATCTG

TGCCCACTTAGCAATCAGGAGGAGTCATTCACTCTCAAACGACCCCGACCCCAACCCCAT

CACCAGCCCACTTCTGCGGGAGTGGAGAAACCCAATGCCAAGAGGCAGAGGAAGAATCTA

TACAGAGGTATAAGGCAGCGACCCTGGGGTAAATGGGCAGCTGAGATTCGTGACCCAAGA

AAAGGAGTCCGTGTTTGGCTCGGTACCTTCAATACTGCTGAGGAAGCAGCTAGAGCCTAC

GACAGAGAGGCTCGCATAATTCGCGGCAAGAAAGCCAAAGTCAATTTTCCTAACGAAGAT

GAACATAACTATACCTCCCAAAACTCAAATACCAAGCCTCCTATGTACCCAACCCCCACT

TGTCATTTCTCAAAGGGATATGATTTTGGGTACGGTTCTGGTATGAATCAGATTGAATCC

TACTCTTCATATGGTTTTAATGGCCATCCAATGGTCACTTCTGGGGAGGATGATTCTGGG

TCCGCATCAGAGGAAGGAACAAGACTGGTGGACTGTAATCACAACGTGGAGAGTAATATT

TTTATGGGTCAAGTGAAGCTGGAAGTGGAAGACCAGAAGCTAGAGAAGGTGAAGAGCAAG

GAGGTGGTGTTGGTGGATTTGGAAACTGGAATGGAAGAAAACGAGGTGCAGAAACTGACT

GAAGAGTTAATTGCGTATGAGAATTACATGAAGTTTTATCAGATTCCCTATCTGGATGGG

CAATCCATGGCACCAAATGGTTCCACCCAGGAAAACCTTGTGGGTAATCTTTGGAATTTT

GATGATGACAGCGTCGCTCCTCCTGTCACCTCGGCACCTCTGTAA

>EVM0004610

ATGTGTGAGTTAAAAGTGGCGAACAGAGGGAAGAGAACAGTTAAAAAAGAAGTTGGTAGT

GTTGAAGAATGGCCACTACCAGCAATGCTTTCTGGGCTGGATAGGGAAAGAGAGATGTCA

GCTATGGTTACTGCTTTGACACATGTAGTGGCTGGAAATGTGCCTGCAGAGTTTTCGGAT

TGTTTAAGCGAAGCAGTGTCAAATAATCAAGATACTTGTGCTCATGGTGATTTGTCAGCA

AAGAGAGAAAGGGAAGAAGAAGAGGGTGGTGGCAAAGAAGAGTATAAAAGGCTCAGTAGA

GCATTTGGCGATGACTTCTCCATGCATGGGGGCGGCTCATCTTCTGCTGGAAGAGCTACG

GGAAAATCCATCACCACCACACGCACCATGACACCTGCAGCCACACGGATGGCATTAACT

CCCGTATATGAATATAATGAAACCTGCAGAGAAGAGCCGAGGAGAAAATATAGGGGAGTG

AGGCAAAGGCCATGGGGGAAGTGGGCTGCTGAGATAAGGGATCCATTTAAGGCAGCTAGA

GTTTGGTTAGGCACTTTCGATACAGCTGAGGCTGCAGCAAGAGCCTATGATGAAGCCGCT

CTCAGATTTAGAGGCAACAAGGCCAAGCTAAACTTCCCAGAAAACGTCAAGCTTCGATCG

TCTCCCCCCAATCCAATCGCAAACCAGTTGACCATCTCCGATTCTCCACGTAGCACCGGC

CTTCTGTCCATTCCTATATCCACCGAGGCCATAATTCATTCCCAAGCTCTTCACCCTACG

CAGAGCCGTGAGATTTCAAGAGAGCAGGTGAACCAACCTCAATTGTTCTTAGATTTTGGT

GGTTATCAAAGGCAACCCATAAGTCTATACGACCAGATGTTTTTGCCACCTCCAGTTGTT

TCGTCTTATCCTTCGATATCTTCTTCAGCAGAACATTCCGATCACATGTTTTTACCAGCT

CAGCAACCAGGCGAGATTAGGCAAGCCACTGATAGCCAAAGTGGTGGGGCAGAATTTCAG

TTGCCAGAGTGGTCAGATTATAGCTATTATACTTCCTCTTCTGGATAG

>EVM0025179

ATGGAGGAGAGAAACGGTGGTAGCCATGGCGGTGTGAGTTCGAGCTATAGAGGGGTGAGA

AAGAGGAAATGGGGGAAATGGGTGTCAGAGATTCGTGAGCCGGGGAAAAAAAATAGAATT

TGGTTAGGGAGTTTTGAGACCCCAGAAATGGCAGCAGCAGCTTATGACGTTGCTGCTCTA

TATTTCAGGGGAAATGATACAAAGCTTAATTTTCCTGACTTGGTTCATAATCTACCGAAG

CCAACAAGCTCTAGCTCTGAAGATATACGCATGGCAGCCCATGAGGCAGCCATGAGCCTT

AGGCCATTCACGGCCGAGCCCTCCCATCGTGGCAGCTCCACCTCCAGCGCCGGTCCCATC

ACTGTTAGGCTCTCTGCAAGCCAAATTCAGGCCATCAATGAGTCACCCTTGGATTCACCG

AAGACATGGATGCAAATGTCAGAGATAGCAATGCTGGAGTCGCCCATGGTGTTCTCCCAT

GGCATCGAGGAGGATCAGTGGGATAACAAGCAAACATATTCTCTTTGGGATCCTTAG

>EVM0006866

ATGGAAGGAAGAATCAGGGAAGACCATCTTGGGATTAGTCCGCCGAGATACAGAGGTATA

CGACAACGAAAATGGGGGAAGTGGGTGTCCGAAATCCGGGAGCCCGGTAAGAAGACAAGA

ATCTGGCTAGGGAGTTATGAGATGCCTGAAATGGCGGCGGCCGCATACGACGCGGCAGCA

TTGCACCTTAGAGGACGCGGGGCGCAACTAAATTTTCCTGAAATGGTGGATTGTTTGCCC

CAGCCGGCGAGCTCTAGCCCAGAGGATGTGCAAATGGCGGCACAAGAAGCGGCTTCGCTG

TTTCGGAGACCAATAAGATGTTCGGAGGCTGTAACCGGTGACTCTAGTGGTGTTGTTGGT

GGTGGTGGTAGTCTAGGTCCTGTTAGGGTTGGGTTGTCACCAAGCCAAATTCAAGCTATA

AACGAGGCACCATTGGACTCACCCAAAATGTGGATGGAGTTAGCCGGGGCTCTTCTGCTG

GAGGAGCCGATGATTATGGGTGATGATATTGATGTTGAGAATAGTGATGAGTGGGGAGAA

ATGCAGCATGATTCCATTTGGGATTATTAA

>EVM0041252

ATGGATCGCTTTTCTACCAAATACACAGAGCATAAAACCGTGACCAACAAGCTGGTGAGA

TGGGAGGACTCAAAAGCCACAAGAATTGTAAGGATTTCAGTAACTGATGGGAATGCAACG

GACTCTTCTGGAGATGAAAATGAGGAACCCAAGATCCAACATCCAAGAATTATAAAGCAT

ATCAACGAGATAAGAATGACAAATTGCAGCGACAGAGCAGCAAGGGATGCGGCAAAATCA

TCAAGACAACAGGTTGTGAAAAAAAACTCAAGGGATCAGTATTATTATCCAGAAGGGAAA

AAGTATCGTGGTGTGAGGCAAAGGCCTTGGGGGAGATGGGCAGCAGAGATCAGAGACCCT

TATAAGAGAACCCGGGTATGGCTAGGGACTTATGATACAGCAGAGGAGGCAGCCATGGTT

TATGACGAGGCTGCTATTCGTATTAAAGGACCTGATGCGCAAACCAATTTCGCAAAAACT

CCAGTGAGCAAACAACATACCCCGGATGTTGATATTAATGCTAACATTTCTGGGTATGAT

TCTGGTAAAGAGTCTCGCAATAGTCTATGTTCTCCAACTTCTGTACTTAGATTTCAATCT

ACCGAGGAACCAGGTCCAGAGTCACACGCTACTGTTGATGCTGCTGTTCAGAGTGATTGT

TTCTGGAGACAAAAAAAAGAGATGGTTCAAGAAGAAATACTGAAGGGTGATGATGATGAG

TGCATGGTAATGGATCCTCTGTGTTTGAAGGAGTATTGGGATTTTGAAAGTCCTGCGCCG

ATTTTTTTTGAGGAGTGTAGTGTACCGGATACTGTTTTGAGAGAAGATTATGCTGATATG

CCGGTGCATTTAGATTGTGATTTTGGCTCCTGTCTATGGGATGTTGACAAGTATTTTGAG

GCATAA

>EVM0042752

ATGAAAGGTAAAGGAGGGCCAGAGAATTCAGTCTGCAATTACAGAGGTGTGAGACAGAGG

ACATGGGGGAAGTGGGTTGCTGAGATTCGGGAGCCAAACAGAGGGCCTAGGTTATGGCTT

GGTACTTTTCCTACTTCCTATGAAGCTGCTCTTGCCTATGATAGAGCTGCACGAGCTATG

TATGGTCCTTGTGCTCGTTTAAACATTCCAGATGTTGTGAATTCAACAAGCTCGTCGAAG

GATAACTTATCTGCTGTTACACCGTCATACTATTCTTCAGCCGCAAGCCCTGCTGACTCC

GTCGCTACATCAACCCACTCTGAGGTTTTTGTGTACGAGGATCCTAACCAGAATGTATTA

AGCCAAGCCGTGGACTGGAGGGCAAATATATCAAGCCAAGCTGAGGTTTGCGAGCAGAAT

GTATCTAGCCAACATATTGAGGATTGTTTTCGGGGAGTTGAAAAAAGTAGCAAGCTGAGT

CAAGATGAGCTGAAGATTCAATCAGAAAATCCTTTGTTGATCAATGACTGGCACAACTAC

TCGTGGGATGAGATCTTTAGCGTAGAAGAGCTGCTAGGGGATATCGATTCTGGGATGACA

GGAGCAGAGGGCTACTTCAGTTTAGGATTTTGA

>EVM0020075

ATGTGTGGAGGTGCCATCATTTCCGATATCGTTGCCGTTAAACGTGGCCGGATATCGACC

ACCGAGGATCTCTGGCTTGAACTTGACAGCATATCTGAATTTTTCGGATTTGATTATGGT

TCTATAAACAATATTAATAATGGTAGCAAAAAGGAAAACAAATCAAATCTCAATCTCCCT

CAAAAGCCAAGCCAGCTCAACCAAGTGATAACAGAGAAAGTTGAGAAGCCAAGCAAGGCA

ATAGAGCAAGGAGCTGGTCACAAGAAGGCTCAGAGAACCAGAAAGAACGTGTACAGAGGA

ATAAGGCAAAGGCCATGGGGAAAATGGGCAGCCGAAATAAGAGACCCACACAAAGGTGTT

AGAGTTTGGCTGGGCACTTACAACACAGCTGAGGAAGCCGCTAAAGCTTATGATGAAGCC

GCGAAGCGCATCCGTGGTGATAAAGCCAAACTCAACTTCCCCGCCCAGCCACCACCCTAT

TCGGAGGCAGCGGCGCCACCCACCAAGAAACGTTGCATCTCGAGTCCTGAAACGTCTTCT

GTGGCTAGTTTTGACCCCCCTCCACCTCGAGAGCCATATTATGGGTATCAAAACGAAGAC

TATGATCTGACCGAGAAAATCTTGAAGTTGGAGTCGTTTCTAGGCCTGGAGCCGAAGCAG

ATGGCGGTGCAGCAGAGTTGTAACGGAGGTGGAGGTGGAGGTGGTGATTGTCACTGTGAC

CCTGTGGACCTTTGGATGCTCGATGATCTTGTGACACATCACCAGTATCAACGTGAGCTT

AATTATTAG

>EVM0044977

ATGAATAACAAAAATTCACTCCCCGTTCCGTTCTTCCCGAAACCCGAAACATATCATTTA

TCGGAAATGTTTAGTCTCCTCAATGAAAATTCATCATTTCGTGGTTCATTTTTACAGCCC

CAAAATTCATCTTCTTCACCTTTTACCTCTAATCACTACGACCCCACAAATTACTCTCCT

GACTCGACCGATATTTCGGCTATTGTTTCACTCCCCGGTATAATCAAGAAACCACCCGAG

GCACCACTGGTTCTTGATGGAATTGGTGCTGTTGTTGGCCAAGAAGTCCTTTTTGGCACG

ACCAATACCAAGGATTCGGAAACATCGGAATTTGATTCGATTTCTCAACGAATCGGGTCA

AAAGAAAGAAGACAAGAAAGTGGTGCTAAGAGAAACTGTGTTAATAATGAGGCTCCAGTG

CAGAGGAACTACAGAGGGGTGAGAAAGAGGCCATGGGGGAGGTGGTCAGCTGAGATACGA

GACCGTATAGGGCGGTGCCGTCACTGGCTTGGGACATTCGACACCGCGGAGGAGGCCGCG

CGCGCTTATGACGCTGCTGCTAGAAGACTTAGAGGTGCAAAAGCAAGAACTAACTTTGAA

ATTCCTCCTGTCTTGCCTCCTATACCTTCTTCTCCTTCTCCCCCAAGTGCATGTTCAGGT

TCAAGTTTAAACAATGCAGAAGCGAGGAGGAGGACTAGCAATTCTAAAAACAACAAAATG

ATTGGTAATGGTGGTAGAAAGTGTGCAGTCGTTACTTCTGTGGCGCATTTGTTTAGTAAT

TTTGAAGGGAAAGGAACTAGCGGTGGCATTGTGGAGCTTGATTTAAAGTTGGCGACAGGG

TTTGGTGGTCATCACGATGGCAACAAGAGTGCTGCTGGTGCTCCTCCTCCATCTATGGTG

GTTTAG

>EVM0051919

ATGGAGGATCAGTTTCCTAGATTGGAAACCTTCATGCACAAGGAATTGGCAGCTTATAGT

AGTTTTCATGGAATGGCAACAGGATCAAGATATTTTGGCGATCCCACCATTTGGGGTGAT

CTATCTGGGGCTACTTGTAATGACACGAAATCGCCATCACAATCGTCTCCTGAAAGATTG

TTTTCGAGTTCGGAATTGAGTTCTTCGGCAAACGATGTGGTTTCAGGTACCAATTTGGCT

GGTAACATCCCTGGATTTGGTGAACAGGATGTTTCCAAACAAAATCATGAAAGTTGTTTC

ATGTCAGGACTGTATTCCATGAAATCTCCTGGAGGGTCAATATCTGACCATATTGCCAGA

GAAAGCTCCATTAATCCTGTGAATTTCCTGGAATTGTTTCCAGCACTGCATAACCAAGCT

CAAGTATCTGAACCTCCCTCTCCATATTCCTCATCAAACACATCGAAATTCCCAAATTTA

ACCTTGTTTTTGCAGGAACCAGAAGCTTTCATCCAAACAAGCTGTCAAACAACTTGGCAA

GGATCGAATCCCAAACAAGCTGTCCATCAACTTGGCAAGGATCAAAGATGCGAGCCTATG

CCATTGTCTCCAAACACTTCATTCTCCGTGCCTCAACTAGGCCAAGTTCATTGCCAACCA

AGCAATGAATGGCTCAAAATAAATCAAGCTTTGGCAAACTATTCTACCAAAGGATTTAAT

GACTATTGGCTTAGAACTACGCCCACACAGCCGATGAAGTACACCAGTCGAGGAATATTG

CAGAACCACCATCAAAAGCCTTCATTTTTATCAGCCTCAACGTCGCCGGGAAAGGTTTTT

AGAGGAGTGAGGCAAAGGCACTGGGGCAAATGGGTCGCAGAGATTAGATTACCGCGGAAC

CGGACGAGGGTTTGGCTAGGGACTTTCAACACAGCAGAAGAAGCTGCTTTTGCTTATGAC

ACCGCAGCTTTCAAGCTACGAGGAGATCACGCGCATTTGAATTTCCCAGATCTTAAGCTT

CAACTCAAGGCTAATTCATTGAACGGAGCCACAACTGCCCTTCTTGAAGCAAAGCTACGA

GCAATATCACAAGGCAACACCATTTCTGGTCAAAAGAAGCACAGCAATGATCCACCACCA

CCACCACCACCACCAGATAAGATTCTACATGACATGAATGCTACAAAACCGAAAAACACA

ACGAGAAAAGAGTTGAAATTTGATTTGGAAGGCAAAACTGGTTGCCACGAACTGATTCAT

GAGAGCCAGAAAGGTCAAGAGGTCGTCGCATCTGATGTTGAAGCTGTTTTGTTAAGTAGA

ATGCCTTCCTTGGACATGGATATGATCTGGGATGCTCTTTTAGTCTCTGATTCATGA

>EVM0045726

ATGGATCGTCCAGTCAAGTTTAGTGAGCATAGAAACCAGACCAGACTTTCAATCTCATTT

TTATCAAACCCGGTCCAACACTCAAAACCACCAAAAATTGTACGGATATCAGTTACAGAC

ACTTACGCCACTGACTCTTCAAGTGACGAAGAAAACGAGGTGGCAACAGTCTCTTCCCAT

CAACGACATGTCACAAGGGTTAAAAAGTTTGTCAATGAAATCTCTATTGAGCCATCGCCG

CCACCACGACTCTCATCATCGAGAAGCGCTGACGCTGTACTCAGCGACACGGTTTTGAGG

AACAGACCGTCGAAGATTACTAGGAAGAAGGCTGGCGATGTTGGGTCTCGGACGAGACGT

TTGCCGGCAAAGGCAACGACGAATGCGGTGGTGGGGAAGAAATTCAGAGGGGTGAGACAA

AGGCCGTGGGGGAAATGGGCGGCTGAGATTAGAGATCCTTCGAGACGTGTACGGCTATGG

TTGGGCACTTATGATACTGCCGAAGAAGCGGCTATGGTATATGACAACGCGGCTATTCAG

CTGCGTGGACCCGACGCTCTCACCAACTTCATCACTCCTCCGGCTAGGTGTTCACCGGTG

GCAGCCACCTCCGGGTACATCTCCGGTGACGAGTCAAATAGTAACTATAATATTAACAAT

GACCGTGTTGTTAATGTAAGCTCTCCAATTTCAGTGCTCCGGTTTGTTGAAGAGGCCGAG

ACTCAGAGTGTCAGCTCTAGTAGAGAAGTCCGAGGTGCCGGAAATGAAGCTCGAGAAGTA

AAAGAGGGCTCCTGTAGATCGGAGAACTTGTCCGATTTTTCGGAGTATAATTCATCGATC

GATAGTCTATTTCCTCCCACAACTGACATTTGTGAACTTCGGAGTTCAATGAGGGATATT

TTCGAGGAAACAAGTTTTGCTGGCGGAGGGTTCTTAAAAGATGATGATTTCAGTGACATG

GATTTGGATTTCGGGTTTGCTGATGGTGGGCTCGGTTTATCAAGTTGGAACGTGAAAGAC

CATTTCCAAGATATTGGTGATTTGTTCGGCTCGGATTCTCTTTTAGCTTTCTGA

>EVM0015466

ATGGAAAGAATGCAAGGAGACAACTTTTCTTATGCATTTTGCAACACAACCGATATTGGT

TCCTCACTGTCACAACTTATATTGGCAGGAGGGACCAGCACTATGGATTCAATTTTTTCG

CATTGCCAAGCGGCAAGTACTCTTACTAGTCCTGCTTTTGAGCCTTTAGGCTCGTCAGTC

TATCTCCGCCAGAGAGATCTACTGCAAAAATTCATTGAAGAAAACAGAACAAACACTTCC

TTTTCTCACAATTTGCTAACAAACCCTCTTCAAAACCATGTGCACACCAGCAATTATTTG

ACTCCAACCAAGAAGAAACTATACAGAGGAGTCAGGCAGAGGCACTGGGGCAAATGGGTT

GCTGAAATTAGACTCCCTCAAAACAGAATGAGAGTCTGGCTAGGTACCTATGACACAGCA

GAAGCTGCAGCTTATGCTTATGATCGTGCAGCTTATAAACTTAGAGGTGAATATGCCAGA

TTGAATTTTCCAAATATAAAAGATCCTACTGAGCTTGGACTTACAGATTGTGCTAGATTG

AATGCTTTGAAGAGTACCGTAGATGCCAAGATTCAAGCAATTTGTCAGAAGGTAAAAAAA

GAGAGGGCTAGAAAGAATGCAGCAAAGAAGAGTAATTTTGATGGTAAAAGCACGGAAAGT

CAAAAACCAGTGAAGGTAGATTCAAACTCATCAACACCATCATCATCATCCTCGTTTTCA

CCTTTGCTTTCGGGCGAGAATTGGGGTTGTGATGAATTGGTGTCGCCGGCTGTTTCTGAA

GAGGGGCTTTGGAAGTGTGAGAGTTCTTCTCCTTCTGTGTCTGCAGATTGTCCTGTAATG

GTGCCACAAGATTCGGAATCCGAAGGCTGTTCCCTTGCAAGAATGCCATCTTATGATCCT

GAGTTAATTTGGGAGGTTCTTGCTAATTGA

>EVM0014688

ATGGATTTTTTCAGTCATTATTCTGATCCAAGTCCGCTTGGGGCGAGAAATTTGTGGTCA

GTTTTCAATGAGAATAATGGTATTGGTCAAGAACAGTGTTCTTATTCTCCTGTTCTTTCG

GATAGTAGTATTACCAGTACTGTTACTACAGGAGTTCAACCAGTTCCGAGTCTTTCTGAT

GAGGAAGTAATGTTAGCTTCGAGGAATCCGAAGAAGAGGGCAGGAAGAAAGAAGTTCAGG

GAAACAAGGCATCCGGTGTTTAGAGGGGTGAGGAGGAGGAATTCAGGTAAGTGGGTCTGT

GAAGTTAGAGAGCCTAATAAGAAATCAAGAATTTGGTTAGGGACTTTCCCTATCGCTGAA

ATGGCTGCTCGAGCACATGATGTTGCTGCTTTGGCATTGAGGGGTAGGTCTGCTTGCTTG

AATTTTGCGGATTCTGCTTGGAGGTTGCCGGTTCCGGCTTCTAGTGAAGCTAAGGATATT

CAGAAGGCTGCAGCGGAGGCAGCTGAGGTTTTTCGGCCGGAGGGCTGTGTGGGAGTTGAG

TTGATGAGGAGAGAAGATGAGGGGAAGAAGTTGGCGGAGACTCCTGCAGAGGCGGAGGAG

GAGGTGTTTTATATGGATGATGAGGCTATTTTTGGAATGCCAGGGTTGTTGGCTAATATG

GCTGAAGGGATGTTATTGCCACCACCTCATTGTGGTGGTGGAGGGGATGGTAGGGGTGAC

ATGGACGATATTGACCCTGATATGTCACTATGGAGTTTTTCTATATGA

>EVM0044087

ATGGCAGATCCCAATTCAAATGCTCTCCAAGCAGACCAGTTACCACCACCTCAACCTTCA

AACCCATCTATACAAATCCCTGACTCTCCATCCTCTATTCTGCTCCAAGATGATCAATCA

CCTCATTCCACCTCCTCGAACCCGTCTCCAAGATTGTTTCCTTCTCCAGGTGGATCTCTG

GGACAGCCATTAACGGTGAGACATGATGAATATTATTCACCAAGGTCCGGGTTTCCGAGC

CAGCCTGTGAGTGGAAGGCAGGATGACCAGTCACCAAGGACCACCCGATCCTCAGGTGGG

TCATCGACTCAGCCCACAAGCGGAAGACATCCGAGTTATAAAGGAATTAGGTTACGGAGT

GGGAAATGGGTATCAGAGATCCGGGAACCACGTAAAAGTACGCGAGTATGGCTTGGTACG

TATCCAACTCCTGAAATGGCAGCCGCCGCTTATGATGTGGCGGCTCTAGCCCTTAAAGGC

CCTGATACACCACTAAATTTCCCGGAATCGATTCATTCCTACCCAATACTAGCCTCAGCA

TCCTCTAGTGATATAAGGGCCGCAGCTTCTAGTGCAGCATCAAGCAGGTCGCCGAAACCT

GAAGCTGCACCAAATCCTGAATTGGATCGCCTGAAACGGAAGGCACATCATCCAGAATTC

ATCGACGAGGAAGAACTTTTAAACCTTCCCAATTTGCTAGTTGACATGGCAGGAGGAATG

CTAGTAAGCCCTCCAAGAATAAACACGCCATCTTCTGATGATTCACCAGGGAATTCCGAT

GCTGAAAGCCTATGGTCCTACCAGTAA

>EVM0018678

ATGGTAAAACCATCGTCGTCATCAGGGCAACAGTCTGATAGAGATCATGAGCCCAAGTAC

AAAGGTGTTAGAAAGAGAAAGTGGGGGAAGTGGGTGTCCGAGATTAGACTTCCCAATAGT

CGGGAGAGAATATGGTTAGGATCTTATGACACGCCCGAAAAGGCGGCGCGTGCATTCGAC

GCTGCCCTGTATTGTTTACGTGGTAGTGGTGCCAAGCTTAATTTCCCCGACACTCCACCG

GACATTGCTGGCGGGCGGTCACTTAGCTCGCAAGAAATACGAGAAGTTGCTGCCAGGTTT

GCTAATGATGAGCCAACATCGTCGACTGCTATGGGAGATGAATCATCAGCACATGTGGAG

AATTACACATCATCATCGTCTGATGGTGGGGCTGGAGATATTATAGATTGGTCGTTTTTG

AATTCGTTGGATTCTAGCGAGGGTGCTTCAGATTTTGAACTCTATCATGGGCTGGACCGT

ATCGGTGGTGATTATTACCCCCCACCACCGCCGCCTCTGCCTGAATATAGCATCGATGGG

GATGATCACATTGGAGATGAAGCTTACTCTCACCATTCATTTCTTTGGAACTTTTGA

>EVM0019323

ATGGTACAATCCAAGAAATTCAGAGGCGTCAGGCAACGCCAGTGGGGCTCCTGGGTGTCT

GAGATTCGCCACCCTTTACTGAAGAGGAGGGTATGGCTGGGGACATTTGAGACAGCTGAG

GCAGCGGCGAGAGCATACGATCAAGCAGCTATATTGGTGAATGGACAAAATGCCAAGACC

AATTTTCCTGCGAGCCATCTTGATCAGGACACTAAGCTTGGCAAAGATGGCAGCTCTGCC

TTGCCTGCCAAGGCACTGGCTGAGCTTCTCTACTCGAAGCTAAGGAAATGTTGTGGAAAA

AACCCTTCTCCCTCGCTCACTTGTCTGAGGCTCGACAATGATAACTCTCATATTGGCGTG

TGGCAAAAGAAAGCTGGCTCCCGTTCAAGCTCAAACTGGGTCATGAGGGTCGAGCTTGGA

AATAGCAACACGAAGAGCGCCCAGGTTATGGAGGAACTGCGACCTTCGTTGTCATCAAAG

TCATCGTCAACGGTTGAGATTGAACCTGAAAATGGTACGGATGAAGAGGAGAAAATAACC

ATGCAAATGATAGATGAGCTGCTCAATTGGAATTGA

>EVM0029410

ATGGAAGGAGGGTGCTATACATCATCAGGCACTTCAGGCTCAACTTCAGCGACAAGGATT

GAGAAGCGTAAGCATAGCAGGCAGCAAAACCAGGAGAAGCCATATAGAGGGATAAGGATG

AGGAAGTGGGGCAAGTGGGTTGCTGAAATTAGAGAACCAAACAAGAGATCTAGGATTTGG

CTCGGTTCCTATTCAACACCAATTGCTGCAGCTCGTGCGTACGACACGGCCGTTTTCTAT

CTCCGGGGACCTTCTGCTAGGCTTAATTTCCCTGATTTGATACACCAAGAAGATGAGTTG

TGTGATGTGTCTGCTGCTTTTATACGCCAGAAAGCCACTGAAGTCGGGGCTAAAGTGGAC

GCCTTACAAACAGCACTCCATGCATCACCTGGAGACAATCCACCAGCAAATCGATTGGTA

CTTTCAGAGAAACATGATTTGAACGAGTATCCGGAGAATTCTGATGAAGAGAGTTCAAAA

GAAGATCAACGTTAA

>EVM0018397

ATGAAATCTACGGGTGATAACAACAATGGAAGTCATAATAACTGGTTAGGCTTCTCTCTC

TCACCCCACATGAAAATGGAGGTTGCCTCTGACCCCCAACATCATCATCAGTACCATCAT

CAAAACAATGCTTCTTCTGCTGTTCCAACAAGTTTCTACCTATCCACTGCTCACTTCATA

GCTCTGGAATCGGTTATGGAGTTGGAGAAAATGATGGGATTTCACTCTCCTCTGTCTGTT

ATGCCCCTTAAGTCTGATGGGTCTCTTTGTATCATGGAAGCTCTCACTAGATCACAACCC

GAAGGAATGGTGTCGAGTCCGTCACCGAAACTGGAGGACTTCCTAGGTGGTGCAACTATG

GAAAGTCATCAGTACAGTAACCATGAAAGGGAAGCTATGGCCTTAAGCCTTGACAGCATA

TACTACCATCAAAATTCAGAGCCAGAAACTAACAGACAACATTCACTCGACCTTCACGAA

CCATATAGGCAGCAAGACCAGCAGTTTTCAGTTCAGCCCCATCCATACTATTCTGGAATA

GCATGCCAAGATTGTAGCTCACCTGTTCCTCAAATGGGAGACGATGAACTGCCTTGCTTT

AAAAACTGGGTCAATAGACACTATTCCTCCCAAAGTGCACTGGAGCAGCATATTAATAGT

AGCATGGTTAATGATGGCGGGTCATCTTGTTCTGTTAGTGCCATGGGGTGTGGGGATTTA

CAGTCTCTCAGCTTGTCTATGAGTCCTGGTTCGCAGTCAAGCTGCATTACAGCTCCGAGG

CAGATCTCTCCTACTGGCACTGAATGCGCTGCTATAGAAACGAAGAAGCGAGGTCCTGCA

AAAGGGGGTCAAAAACAACCTGTTCATAGGAAGTCTATCGACACATTTGGCCAAAGAACA

TCACAGTATAGAGGTGTCACAAGGCACAGATGGACTGGTAGATATGAAGCCCATCTCTGG

GACAATAGTTGTAAGAAGGAGGGCCAGACCAGGAAAGGAAGGCAAGGGGGTTATGATATG

GAAGAGAAAGCTGCAAGGGCTTATGATCTTGCTGCCCTTAAGTACTGGGGGCCATCAACT

CATATAAATTTTCCGCTAGAAAATTACCATGAAGAACTTGAAGAAATGAAAAATATGAGC

CGCCAGGAACATGTTGCTCATTTGCGAAGAAAAAGTAGTGGGTTTTCCAGGGGGGCCTCA

ATCTACAGAGGAGTAACGAGACATCACCAGCATGGAAGGTGGCAAGCAAGGATAGGCAGG

GTTGCAGGAAACAAGGATCTTTATCTTGGGACTTTCAGTACCCAAGAGGAAGCTGCAGAG

GCTTATGATATCGCCGCGATCAAATTTCGCGGGGTTAATGCCGTGACGAACTTTAACATA

ACAAGATATGATGTTGATAGGATCATGGCCAGCAACACTCTTCTAGCAGGAGAGCTAGCC

AGACGAAACCGAGATACGGAATCTAGCATTGGGGCCATTGATTATAACACATCAACACAC

AACAATGGCGTTCTATATCAGTCATCGCAGGAACAGCCAAATGCAGAATCTCTTGATCAG

AAGTCGATGAGTTCTGGAAATTATCGAAGCTCTTCTTTCTCAATGCAAGATCTTATTGGT

ACTGATCATTCAGTGAACTCTAGCCAGCTAGTGGTGGACGAATCAGCCAAGCTAGGGACT

CTTCTCTCTAACCCTTCATCGTTGGTGACCAGTTTGAGCAGTTCTAGAGAACCTAGCCCT

GATAAGACTGCCACCCCTATGCTCCTCGCTAAACCTCCACTAGCATCAAAGTTCGTTAGC

CCTACAACTAGCGTTAATCCTTGGTTCCAAGCTGCCCAATTGCGGCCAGCGGCAATCCCC

ATGTCTCACTTGCCAGTTTTTGCTACATGGAATGATACCTAA

>EVM0028452

ATGACTGTCTCGCCGGTTTCAATCTCCAACTCCGAAACAGGCTCCTCAAGCATTTATTCC

TCCTCCCTGCAAACCCCTTATTCCCCATCTTCGTCAGGACTATCACCAAACCCGGTATCT

AAACCCGACCCGGAAGAAAACCAGAGAAAACCAAAGAGGCCAAGAGAAAGCGGCAGCAAG

CACCCGGTATTCAGGGGGGTCCGAATGAGGACATGGGGGAAATGGGTGTCGGAGATCCGG

GAGCCCAGAAAGAAGAATCGGATCTGGCTAGGCACTTTTTCCACCCCGGAAATGGCAGCA

CGTGCCCATGACGTGGCAGCCTTGAGCATTAAAGGTAAGTCAGCCATCCTCAACTTTCCC

GAACTTGCCGGGTCTCTACCACGACCCGCCTCTAGCTCTCCACGTGATGTCCAGGATGCC

GCCGCAAAAGCCGCTTCGATGGACTTCAACATTGTGCCTTCGGCTCCCGCCAAAGTCTGC

GACGATGAAAATAAAAGTAACAGTAACGACTCGACGGCGAGCAATAATAATGGGTCATCG

TCCTCGCTAGTGACACAGTCGTCCTCTTCCCCGTCCTCATCATCGACAGTGGAGGTGACT

TCATCACCAAGTGACGTGGCGACTCCTGAAGAACTGAGTGAGATAGTTGAGTTGCCGAGT

TTGGAAACAAGTTTCGAGGAATTTCCCGAGTTTGTTTTGTCTGGTGACTCGTGGCCGTAC

AATATTAATCAATCCTGGTATTGTGAAGACTACGGTGCGGAGGGCACCGGGTATTTTAGC

GATCCGTTTTTGTCAATAATTCCAGAAAGTAATGTGATTTCGACTACTGGTGCTTTCGAG

ACTTTATTGTGGGAACATTGA

>EVM0025980

ATGGATTCTACTTCTCATCAGAACTGGCTCGGTTTCTCTCTTTCCAACCATCATCACATG

AATAATATCAGCATCCCTTCCTCTTCTGATTCCTCTCACCTCTGTCTCTATGAAGCCTTT

AGCACCACACCCACCACCACTACCTCAGCACAAGAAGATAATGCAGTAGCTGCCGGTAGA

CCAGCTGACATCTCTTTATTTACCTCTTCTGGACCAAAATTAGAGGACTTCCTTGGTGGC

TGCACCACTACACAGCCACCACAACAGCCACTGGGTGGTCTGTTCTCGACTGAGACACCC

GGGACTACTACGCCTGCTACTGTATCTGACAATAGTAGCTCTGAGATATATGATTCCGAG

CTCAAAACTATAGCTGCCAGCTTATTTCGTGGTTATTCCTCTACTGATCACCAGCAAACC

GGTAGCTCTCAAAAACATCAGCAGCTTCTTTTGCAGCCTGAACATGCACCAAAGAAAACT

GCTGATACATTTGGCCAACGTACTTCAATTTATCGAGGAGTCACCAGGCATAGATGGACT

GGTAGATATGAAGCCCATTTGTGGGATAATAGCTGCCGAAGGGAAGGCCAAAGTAGAAAA

GGGAGACAAGTCTATTTGGGTGGCTATGACAAGGAGGAGAAAGCAGCTAGAGCCTATGAT

CTTGCAGCTCTCAAGTACTGGGGTCCGACCACCACTACAAACTTTCCGGTTTCCAACTAT

GAGAAGGAGGTGGAAGGGATGAAGCACATGACAAGGCAAGAGTTTGTTGCGTCACTTCGA

AGAAAAAGCAGCGGTTTCTCCAGAGGAGCCTCAATTTACAGGGGAGTGACAAGGCACCAT

CAACACGGCCGGTGGCAGGCAAGGATCGGAAGAGTTGCCGGCAACAAAGATCTCTATCTC

GGCACCTTCAGCACCCAGGAAGAAGCTGCCGAAGCTTATGACATTGCAGCAATCAAGTTT

AGAGGCCTAAATGCTGTAACCAACTTTGACATGAGTCGCTATGACGTCAAAAGCATAGCT

AACAGCAATCTTCCGATTGGAGGAATATCTGGCAAGTCAAAGAACTCATCAGAATCAGTT

TCTGATAGCAAAAGCATTGATTTAAGTCGATCAGACGATCGAGATCTCTCCTCAGCGTCC

TCTGTAACCTTTGCTTCCCAGCCTACGACCTCTACTCTTAGCTTCTCCATGCCCATCAAA

CAAGACCCATCAGATTACTGGACCAACATCCTTGGATACCAAAACAACACCATGAATAAT

ACCAAGAACAGTAGCAGCATTGTTGCACCGAGCACTTTACTTCAATCTTCCACAGGTTTT

CCTGCTTTTCAAGGTCCAACAGCTTTCAGTATGGACTTTAACACGCATTCGTCAGTCAAT

GAAAGCAACCACAGTGGGATCTTATTCAATGGTGGTTATATAGAGCAGCAGAATGGTGGC

GATGGAATCAGTACATCATCGTCAACCTCGAGTGTTCCATTTGCTACATCCATTGCCTTG

CACAGTAATGGAAGCAGTTACGAGGGTAACTCGAGTTATGGTACCTGGATTTCTCAATCT

TTGTTCCAATCTGCGAAGCCAAACCTCTCAGCGTATCAGACACCTATTTTTGGAATGGAA

TGA

>EVM0057600

ATGAAGTCCATCAACAATGATGATAGCAACAATAACCATAATGCTGACTGGTTAGGCTTC

TCTCTGTCCCCCCAAATGAATATGGAGGTTCCATCAGGTACTGATCACCATCAACAAACA

CAGTCTGCCTCTGCTTCTGTTCCTGCTGCGATCCCAACAAACCTTTTCCACTCCCAGTTA

CCACACCTTAACAGCGGTATCTACTATGGTGTTGACGGTGAAAATGGTGGTTTTTACTCT

CCTTTGCATTTTATGCCGCTCAAGTCTGATGGGTCACTTTGTATGATGGATTCTCACACC

AGGACACGGCCTCAAGCAACAATGGTGACAACTTCAACACCAAAACTTGAGGACTTCTTT

GTTGGGGCAAATATGGGGACCCATCACTATGAAAGCAATGATAGAGAAGCTATGGCTCTT

AACCTAGACAGTAGTCCTCCTATGTATTATCACCACGACACAGACCATGCGCCGAGCAAC

CAATTTTGTCCAAACCATCTCGACCAAAACCCCAGACAGCAGCAGCATCACAACATTCAA

GTTCAACAGTACCCGTATTACTCTAACTTTAGGAACGATGAGATGTTAATAGGAGAGGAT

GCCAAGCAAATGACTCAGGTTTCAGATGGCAATCTTCTGCTTCCAAATATGGGAGACGAT

GGGATAACTGGCATGAAGAATTGGGTTTCGACAAACTATCAAAGCAACCATGCCATGGAA

CAGAAGATGCTTAGATGTATGGTTGAAAATGGAGGTGAATCTGGGCCTAATATCAGCGCA

ATGACATACGGGGATTTGCAGTGTTTGAGCTTGTCAATGAGTTCTGGCTCCCAGTCAAGC

TGTGTCTCTGGTTCTTCTCATCAAATCTCACCTTCGGTGGCTGACTGTGCAGCCATGGAA

ACCAAGAAAAGAGGAACTGAGAAGGTGGATCAGAAGCAAATTGTTCATAGGAAGTCCATT

GATACATTTGGGCAAAGAACCTCTCAATATAGAGGTGTTACAAGGCATAGATGGACAGGA

AGATATGAAGCCCATCTATGGGACAATAGCTGCAAGAAAGAAGGTCAAAGCAGGAAAGGA

AGGCAAGTTTATCTGGGTAAGCCTTTTTCATGTACAATGATCAGACATTTTTCAAATATA

GACATATGGGGTTATGACATGGAAGAAAAAGCTGCCAGAGCTTATGATCTAGCTGCACTC

AAGTATTGGGGACCTTCTACTCATATCAATTCCCCTTTGGAAAATTATCAAAAAGAAATT

GAAGAAATGAAGAACATGACCAGACAGGAATATGTTGCTCATTTGAGAAGAAAAAGTAGC

GGGTTCTCTAGGGGAGCTTCAATGTACCGGGGAGTAACAAGCAGACATCATCAACATGGA

AGATGGCAAGCTCGGATCGGAAGGGTTGCCGGAAACAAGGACCTTTATCTAGGAACATTC

AGCACCCAAGAGGAAGCAGCTGAAGCCTATGATATAGCTGCTATAAAATTTCGAGGAGTT

AGTGCTGTGACCAACTTCGACATAACAAGGTACGATGTGGAACGAATCATGGCTAGTAAT

ACGCTTCTTGCTGGGGAATTAGCTAGGCGAAACAAAGACATTGGACCTTGTGATGATGAT

GCTACCAATCAAAATCCTTCTACTCATAACAGCAATGGGGAATCCATGCCCCCCTCACAG

AAGAGTGAAAACGAGTCGGATTGGAAGATGTTCTATCAGTCCTCCCTGCAACAGGATCAC

AAAGCCTCGGATGGTGTGGATAATTACAAAACTCAGGCATTCTCGCTGTCCCATGAAAAT

GCAATTGCAGATGAGAGCATGATCTCAATTCACCAGCAAAAGGTTGAAGACTCTGCCAAG

ATGGGAACTCACATGTCAAATGCGTCGTCGCTGGTGACTAGTTTAAGTAGCTCTAGAGAA

GGAAGCCCTGATAGAGCAAGCCTTCCAATGCTCTTTGGAATGCCTCCCTTAGTAGCATCC

AAGTTCACCAGTCCAAATTGTGATGTGAATTCTTGGATCCCAGCAGCAGCAGCATCCCAA

TTGAGGCCTGCTGCAGTGTCGTTGCCTCACATGCCAATGTTTGCTGCTTGGACAGACGCC

TAG

>EVM0031973

ATGGAGCAACCACCATACACAGGAGATGACCCGATCACAAACTCTCCAGAGGCAGCCACC

GCCCTAACCCACCAAAATCCTGAAACTCCACGGAGCAGCGGCACTCGAAACCCTGCCTAT

CGCGGTGTCCGAAAACGGAGGTGGGGGAAATGGGTGTCTGAAATTAGAGAGCCACGCAAG

AAATCACGTATCTGGCTAGGCTCGTTTCCTGTCCCAGAAATGGCAGCCAAGGCGTATGAT

GTGGCAGCATATTGCCTTAAAGGCCGTAAAGCGCAGCTTAATTTCCCTGACGAAGTGGAT

GATTTGCCCAGACCGTCCACGTGCACGGCCAGGGACATTCAAGCAGCGGCAGCAAAGGCA

GCTCATTCAGTGCTGGTTCCAAGGAAAAAGAGCATCGAGACAAATAGTGGCAATAGTGTT

GACGGAGAAGTTGCCGGCGATGATTTCTGGGGCGAGATAGAATTGCCGGAATTGTTGATG

AGTAACAGTGGGTGCTGTTGGAATTCTTGTGGATGGAGTACTGTTGCAAGTGATAGCTCA

ACGTGGCAACAGGATGGAGAGGGTCTACCCCAGTTCATGGCGTGTCTTCATTAG

>EVM0025365

ATGGAGTATCATCAATTCCAAAAGCAGCCAAAAGGAGTCTCACGCAGCAAGTTCAAGGAG

AGATGCTCAAGCAAGACCTCCAAGAACAAGTTTGTTGGCGTTAGACAAAGGCCTTCAGGA

AAATGGGTGGCAGAGATCAAAGACACTACACAGTCACAGAAGATCAGAATGTGGCTTGGG

ACCTTTGAGACAGCTGAAGAAGCTGCGCGAGCTTATGATGAAGCTGCCTGTCTTCTTCGT

GGTTCGAATACCCGAACCAACTTTGGCACTCATAACTCTTCCGCAACCTCCACCACAAAA

TCTACCATTAAAACCAGCACTATAGTTAGCACCAAGAGTAGTTTCAATATTAGTTGTTTT

CTACCTAATGATAGCAACATAGATAGCTTCCCATCATCTATTGATAACCATGGTAGTTTT

ATTAATGGCACCAAACAAGAAAACAAAGCGTTTGATGATGCATATGGGCCAGACTTGAGT

GGCTGCTGCGTTGGAGGGCTTGAACCGGTTACATCTCAGTTCCATCCCTCGTGCTCACTC

CCATCTGGGTTTGATCCTCAACTTCAATTTGTTCAAGAAGGAATGTGGTTTCCGAAGAAT

GATGGTTTGTTGTCTGATACATCACCTGGTCCAGAGTTGGTCGAATTCGAACGCATGACG

GTGGAAAGGCAGATATCAGCATCATTATATGCCATGAATGGAGGGAATGAGTACTTCGAG

AATGCTAATGATTCAAGTGATGCTCTCTGGGATCTCACTACACTCTGCCAATTGTTCTGT

CCTAGTTGA

>EVM0000460

ATGGAAAGAATGCAAGAAAACAACTTTTCTTTTGCATTTAGCAACACAACTGATATTGGT

CCCTCACTGTCACAACTTATATTGGCAGGAGGGACCAGCACTATGGATTCAATTTTTTCG

CACTGCCAAGCGGCAAGTACTCTAACTACTCCTGCTTTTGAGCCTTTAGGCTCGTCAGTC

TATCTCCGCCAGAGAGATCTACTGCAAAAATTCATTGAAGAAAACAGAACAAACACTTCC

TTTTCTCACAATTTGCTAACAAACCCTCTTCAAAACCCTGTGCACACCAGCAATTATTTG

ACTCCGACCAAGAAGAAACTATACAGAGGAGTCAGGCAGAGGCACTGGGGCAAATGGGTT

GCTGAAATTAGACTCCCTCAAAACAGAATGAGAGTCTGGTTAGGTACCTATGACACAGCA

GAAGCTGCAGCTTATGCTTATGATCGTGCAGCTTATAAACTTAGAGGTGAATATGCCAGA

TTGAATTTTCCAAATATAAAAGATCCTACTGAGCTAGGACTTACAGATTGTACCAGATTG

AATGCTTTGAAGAGGACCGTAGATGCCAAGATTCAAGCAATTTGTCAGAAGGTAAAAAAG

GAGAGGGCTAGAAAGAATGCAGCAAAGAAGAGTAATATTGATGGTAAAAGCACGGAAACT

CAAAAACCAGTGAAGGTAGATTCATACTCATCAACACCATCATCATCATCCTCATTTTCA

CCTTTGCTTTCGAGCGATAATTGGGGTTGTGATGAATTGGTGTCGCCGGCTGTTTCTGAA

GAGGGGCTTTGGAGGTGTGAGAGTTCTTCTCCTTCTGTGTCTGCAGATTGTCCTCTAATG

GTTCCACAAGATTCGGAATCCGAAGGCTGTTCCCTTGCAAGAATGCCATCTTATGATCCT

GAGTTAATTTGGGAGGTTCTTGCTAATTGA

>EVM0024472

ATGGATCATCCAGTCAAGTTTAGTGAGCATAGAAACCAGACCAGACTTTCAATCTCATTT

TTATCAAACCCGGTCCAGTACTCAAAGCCACCAAAAATTGTACGGATATCAGTAACAGAC

ACTTACGCAACTGACTCTTCAAGTGACGAAGAAAACGAGGTGGCAACAGTCTCTTCCCAT

CAACGAAATGTCACAAGGGTTAAAAAGTTTGTCAATGAAATCTCTATTGAGCCATCACCA

CCACCACAACCATCGTCACCAAGAAGTGCTGACGGTGTCCTCAGCGACACTGTTTTGACG

AACAGACCGTCGAAGATTACTAGGAAGAAGGCTGGCGATGGTGGGTCTCGGACAAGACGT

TTGCCGGTAAAGGCAACGGCCATTGCGGCGGCGGGGAAAAAATTCAGAGGGGTGAGACAA

AGGCCGTGGGGGAAATGGGCGGCTGAGATTAGAGATCCTTTGAGACGTGTACGACTATGG

TTGGGCACTTATGAAACCGCTGAAGAAGCTGCTATGGTTTATGACAACGCGGCTATTCAG

TTGCGTGGACCCGACGCGCTCACCAACTTTATCACTCCCCCGGCTAGGTGTTCGCCGGTG

GCAGCCACCTCCGGGTACATCTCCGGGTACGAGTCAAATAGTAACTATAATATTAACAAT

GACCGTGATGTTAATGTGAGCTCTCCAATTTCAGTGCTCCGGTTTGGTGAAGAGGCCGAG

ACTCAGAGTGTCAGCTCAAGTAGAGAAGTCCGAGATGCCGGAAATGAAGCTCGAGAAGTA

AAAGAGGGCTCCTGTATATCGGAGAACCTGTCCGATTTTTCGGAGTATAATTCTTCGATC

GATAGTCTGTTTCCTCCCACAGCTGACATTTGTGAACTTCGGAGTTCAATGAGGGATATT

TTCGAGGAAACAAGTTTTGCTGATGGAGGGTTCTTAAAAGATGATGATTTCAGTGACATG

GATTTGGATTTCGGGTTTGCCGATGGAGGGTTCGGTTTATCAAGTTGGAACGTGAAAGAC

CATTTCCAAGATATTGGTGATTTGTTCGGGTCGGATTCTCTTTTAGCTTTCTGA

>EVM0020564

ATGTTGGATCTTAACTTGATTGCGGCTTCTGGTGACTCGTCGAGGACAAGGAAAAGTAAT

AAACTGAAGCAGAAGAAGATTCTAGAGCTTTCCAACTCTCCAGTGGAAAGCTCTGGCAGC

TTCGATTCTTCTTCTATAGTCAATGCTGATGGCCATGGTGATGAGGACTCTTGCTCCAAC

GGCGACGTTTTTGCTTTCAATTTCAGTGTCTTGTCAAATGAAATCAGCTTCAAGAAGACC

GTTGATTGTGATAATAACGAGGATGATGATGACAGTGGAGATGGAACGATCCAGCTTTTT

CCAGTGGCGTTTGGGATCAAGAATGTAGGAGGAGAGTCTCGGTCTTCTTCGACTATGCAG

ACGCAGTGGTCGGGTCTCGGTGAGCGCTGGGATCAAGGTTCTCATCCGGAGCTCGGGATC

GTGGCTCAACAAAAGCCAGTGAAGAAGAGTCGGCGTGGGCCCAGGTCGAGAAGCTCCCAG

TATAGAGGGGTCACGTTTTATAGGAGAACTGGAAGATGGGAATCTCATATTTGGGATTGT

GGAAAGCAAGTTTACCTGGGGGGATTTGATACTGCGCATGCTGCAGCTAGGGTGTATGAT

CGAGCTGCAATTAAGTTCCGCGGAGCTGACGCTGATATCAACTTTAATGTTGCTGATTAT

GATGGGGATATTAAGCAGATGCGTAATTTCACAAAGGAAGAATTTGTGCATATCCTACGT

CGTCGGAGCACTGGATTCTCAAGGGGCACCTCAAAATATAGAGGAGTGACACTGCACAAA

TGTGGGCGATGGGAGGCTCGTATGGGGCAGTTCCTGGGGAAGAAGTACATTTATCTGGGG

TTATTTGATAGCGAGACAGAAGCTGCAAGGGCTTATGACAAAGCTGCCATTAAATGTAAT

GGAAGGGAAGCAGTTACCAACTTTGAGCCAAGCACGTATGAAGGAGAGATTCTTTCCGAG

CCTCACAATGGAGACGGAGACCAAAATCTTGACCTGAACTTAGGAATTGCTCCTCCTGAT

ACATCTGATGGCCCAAAGGTAGACAGCAACATGGGTGGCTTTTATTTCCAGAGTGGCTGG

GATGACATTTCTTTTGATAGGGCACGAAAGATTCTGAACTCTACTTCTGCAACAATGAAA

AATCAACAACCTAATGGCCCAGGAATGACATCTGTACACCCTCCTACATGGGATGGTGTA

AACCATCGAATCTTTACCATCTCTGAGGAAAGAGCAATAGAGAAGAGAATGGTAGATTCC

TTCCCAAATTGGACCAGGCAAATCCAAGGCCCTTATGGTGGCGGAGCAAATCCACTGCCT

CTCTTCTCTACTGCAGCATCATCAGGATTCTATTCTTCTGCAATTATGGCACCTCCAGCA

GCTGCCGGTCAACTTTACTTTCCAAGTTCCACAATCGTCAACCGTTTCCCTCCGATTTCC

AACCCGGCAACATCCCGCAATTCTACTCTAGTAGCTGAATGGTATAGAACAGCCATAATC

CAGAACCTTTCTTGTACAGAGGAAAGAAATGGGTAG

>EVM0003861

ATGGTGAAGAACAGTGCCGGGAAGCCGATTGCAGAGAGAAGAGACTCAAAATTCAAGGGT

GTCCGAAAAAGAAAATGGGGCAGGTGGGTGTCTGAGATCAGGCTACCGAATAGCCGGGAA

AGAATTTGGTTGGGATCGTACGATTCTGCAGAAAAGGCTGCTCGAGCGTTCGATGTGGCC

CTTTTTTGCTTGCGTGGTCGAGTTGCGAAATTTAATTTCCCGGATAACCCTCCAAACATA

GCGGGTGCCAGGTCACTTTCTCCGGCTGAAATTCAAGAAGTTGCAGCGAAGTTTGCCAAT

TCGGAGCCTCAAAGAAGCCATTCGGATCAGTTTGAAACAAATCAATCTGTATCCGAGTCT

CGAGCAGAATCTCCCTGTCCTTCAGTTGTTTCCGAATCTCGAGCAGAATCTCCATGCACT

TCAGTTGTATCCGATGAGACTGTCCAGTTGGAAAGTAGTGAATTGAGGTGGGATGGGTCG

TTTTTGGATATGTTAATGACTACAAATTCGAGTAATTACTCCGCCGAATATGGAATATTT

CCGGGTTTCGATGATCTTCATAATGATTTTTTCCCATCATCATTAACTCCTAATCTCGAT

TATGGAGAGGAGACCAATTTTGATGGTGTTTTAGAACAAGATTCATTTCTTTGGAATTTC

TAA

>EVM0009928

ATGGCCAGACCACAACAGCGATATCGAGGGGTCCGACAAAGGCACTGGGGATCATGGGTG

TCTGAAATTCGTCACCCTTTATTGAAGACTAGAATTTGGCTAGGCACGTTTGAAACAGCA

GAGGATGCAGCAAGAGCTTACGATGAAGCTGCAAGGCTTATGTGTGGGCCTAGGGCTAGA

ACCAACTTCCCTTATAACCCAAATGCATCACAATCAGCTTCGTCGAAACTTCTCTCTGCA

ACTTTGACAGCAAAGTTGCACAGGTGCTATATGGCCTCATTGCAATTGACCAAAAAAACA

TCACTTCATGAACAAAAACAACAGCAACAAAAGGCACCCACTTCACTTGCCAACACCCCC

ACCAACGGCATTGTCATAAAAAGCGAAGAACTTGATCCTTCATTGCCAGAGAAAAGTCCA

TTACAAGTTCAAGAGGCGGAGGCAAACTGGGTGTACAAGAAAGTTCAAGTTGATAACAAC

CAGCAATTCATAAAGCCTCTTGAAGATCATCACATTGAGCAAATGATTGAAGAGTTGCTT

GATTATGGGTCTATTGAGTTTTTTTCTGGGGTAACAACTCAGTAA

>EVM0053305

ATGGCTCCTAGAGAAAGACCTGACAACCACCACCTCAACAGCCCTAGATCGGAGATCCGT

TTTAGAGGCGTGAGAAAGAGGCCATGGGGACGGTACGCCGCTGAGATCAGAGACCCAGGC

AAGAAAACGAGGGTTTGGCTTGGCACTTTTGATACTGCTGAAGAGGCCGCCCGTGCATAC

GATGCGGCTGCTCGTGAATTCCGAGGAGCCAAAGCCAAAACTAATTTCCCTGCCATTGGT

GAGATTAACCCGAACCCCACGCGCAGTCCTAGCCAAAGCAGCACTATCGAGTCCTCCCCT

CCGACCCCACGCGCAGCTTCCCCGCCGCCACTCGATCTCACTCTTAACATTTCCCACCGT

AACCCCGACCGCCAACAGTTTCCCAACGGAGTCAGGTTTCCAGGAGGCGCGTGGCTCCCA

TTCCCTGCTGTTGCGCGTCCCGTCTTCTTTTTTGACGCGTTTGCTCATGCGAAGAATAAT

ACTCCTAGCAGTAGTAGTATAGTGAATAATATTAACATGTGCAGGTTTGATCGAACGGTG

ATGGTGAATGGAGGTGGGGGGCCTAGTGATTCGGATTCGTCATCAGTCGTTGATTTTGAT

CGTCATCACGATAGCAAGGGATTGTCACTTGATCTTAACCTGGCCCCACCACCGGAAGTC

GCATGA

>EVM0014537

ATGGAAAGAATGCAAGAAAACAACTTTTCTTTTGCGTTCAACACAGCTGATATTGGTTCC

GCACTGTCACAACTTATAATGACAGGAGGGACCAGTACTTTGGATTCTATCTTCTCTCAT

TGCCAACCAGCGCGTACTCTTAACAGTCCTGTTTTGGAGCCCTTAGGTACTTCAGTCTAT

CTACGCCAGAGAGATCTATTACAAAAATTCAGTGAAAAAAACAGAATAAACACACCCTTG

TCTCACAATTTGCTAACAAACCCTCTTCAAAATCCTGTGCACACCAGCAATTATTTGGCT

CCAACCAAGAAGAAGCTATACAGGGGAGTCAGGCAGAGGCATTGGGGCAAATGGGTTGCT

GAAATTAGACTTCCTCAAAACAGAATGAGAGTCTGGTTAGGTACTTATGACACAGCAGAA

GCTGCAGCTTATGCTTATGATCGTGCTGCTTATAAACTTAGAAGCAAATATGCTAGGTTG

AATTTTCCAAATCTAAAGGATCCTGCTAAGTTGGGATTCACAGATTGTGGTAGACTGGAT

GCTTTGAAGAATACTGTAGACGCCAAGATTCAAGCAATTTGTCAGAAGGTTAAAAAAGAG

AGGGCAAAAAAGAATGCAGCAAAGAAGAGTAATGTTAATGGTAAAAGCACGAGAAGTAAA

AAGCCATTGAAAGTAGATTCAAAGGCATCAATGCCATCTCCATCATCATCATTTTCACCT

TTGTTTTCTGGTGATAATTGGGGTGGTAGTGAGTTGGTGTCACCGGCTGTTTCTGTAGAT

GGGTTTTGGAAGTGTGAGAGTTCTTCTCCTTCTGTGTCAACAGATTGTGCAGTAATGGTG

CCACAAGCTATGGAATTTGAAGACTGTTCCCTTGCAAGAATGCCATCTTATGATCCTGAG

TTAATTTGGGAGGTTCTTGCTAATTAA

>EVM0054964

ATGGCAACCCCGGAAGAATCCTCAACATTAGAACTCATAAGACAACATCTTCTTGGAGAC

TTCACCTCCACAGACGAGTTTATCAGTAATCTTGAGTCCAGAATAGCCAGTGTCTCTGTC

AAACTAGAGAGCTCCCTATCTGGGTCAGAATCCAACTCCCCAATATCCGACCAAAGTTAC

CATAGCACCCAAGAAATCTATAGCTTTGAAATAAAACCTGAAATCATAGAGTTGACACCT

CCTGAGCCTATGTTATCAAGTTCAAGTAATCAGTACCCACCACCTGAACCTGTACAGATG

ACAGATAAAGGAGAGGCGGTAAGGCATTACAGAGGGGTCCGAAGAAGGCCATGGGGCAAG

TTTGCAGCAGAGATCCGTGACCCTACCCGGAAGGGAAGTCGGGTCTGGTTGGGTACTTTT

GACAGTGATGTTGATGCTGCAAAAGCTTATGATTGTGCTGCATTCAAAATGAGGGGCAGG

AAAGCAATCCTGAATTTTCCTTTGGAGGCTGGACTATCCAGCCCGCCGCCAACCACAGGA

AGGAAGAGAAGAAGATCAAATCGAGAAGAGGTGCAGCCGGAATCTGTTGATGTGTCAACG

GAAAACTGGAATATAAAGTGGAGTGGGGAAGTAGAGGGTGTTTCAGATGAGGAGCAGCTG

TCACCGTTATCCCAAGAGGGGGCTTAG

>EVM0036702

ATGGAATCTTTTGAAGAAGCTTCAACTTTGGAACTCATTAGGGAGCACCTCTTGACTGAC

TTTGCTTCCATAGAAAGCATAATCACCAATCTTGATGATCTTTGTAGCACCTCCAGCTCC

ACAGAACTCGATCCCTTTGATCAATCAGTGCAGCCACAGCCCATGCTGCAGAGTCAATCA

AGCATCGAATCCAACCAGAGAAAACCATCCATGATCAAGAACATCGCAATTCCTCCGCCA

GCGACCTTGAACATGACCCCACAAGTAGCCCAGCCGGTGGTGAACAGGGCAGATGTTTCG

ATTGAACAGGAAAGGCATTACAGGGGCGTTAGGCGTAGGCCATGGGGCAAATATGCAGCT

GAGATCCGTGACCCGAACAAAAAAGGAGCTCGTGTATGGCTTGGAACATTTGATTCAGCC

ATTGAAGCTGCAAAAGCTTATGATAGTGCTGCTTTCAGGTTACGTGGAAGTAAAGCGATC

TTGAATTTCCCTCTTGAAGCTGGAAAGTCTAATTCTCAGCAACCTGAACAATTCTTGGGA

ACTTCAGGCAAGAAAAGAAAGATTGAGGAAATAGAGAGCAGTATGGAAAGTACTAGTTCT

ATGATAACAACAAAGGTTGTGAAGAGGGAAAGTTCTTCGCCGGAAACTGAGGTCAAAGCA

GCGGCTACGGAACCACTAACGCCGTCCAGCTGGAAGGGGTTCTGGGATGGTGAAGTTACG

GGGATATTTAATGTGCCACCGTTGTCACCGTTATCCCCCCACCCTTCATTTGGGTATTCA

AGGCTGATGGTTGTTTGA

>EVM0054070

ATGGATGGGGTTACCTCATTGTGCGAGTTCAGTGCTTTGTCTCTTCATCATCATCGTCGA

CATAGAAATCCAAGCTTTACTTCTCTCATCTCCTGCCTAACCGAGACATGGGGTGACTTG

CCATTAAAAATGGATGATTCTGAAGATATGGTCATTTACAATTTCCTTAGAGATGCTGTT

CGTTTTGGATGGTCTCCGTTAGACCTAACCTCACCCTCTACTACCACTGCAACGGCAACA

AATGTCGTTAAAGCTGAGCCGGAATATGAGCTGGGGCAGGAAGCCGAGCACATTGAAGTG

GTCCAGGAAGCTGGATTGAAGGGGGCTGTTTTTTCTAAGAAGGTGGTGGCTAAAGGGAGA

CATTATAGAGGAGTGAGGCAAAGACCTTGGGGGAAGTTTGCGGCTGAGATTAGGGACCCG

GCTAAGAATGGTGCAAGACTGTGGCTTGGGACTTACGAGACGGCGGAGGAAGCGGCTTTA

GCTTATGATAGAGCGGCTTACAGAATGCGTGGCTCAAAGGCTCTGCTTAATTTCCCACAT

AAGATTGGGTCTGATGAGCCGGACCCGGTTAGGATCACGACAAAGCGTCGAGAGCCGGAG

ACGTGTTTGCTGGCTGTGGATAGTGGCCCGGCTAAGACGAGGAGAACCGTGGTGGCTAAT

GGAGATGAGATGGAAAGGGGGAGGGGATCTAACGTGTTTCAGGCTGGGCACCATGCAATG

CCAGCTGGTGAGCAAGTATTGGTCAGTTAG

>EVM0054822

ATGTACGGACAGAACATGCTTGATCAGTCCGATTATGCCTTTCTTGAGTCCGTTCGCTTG

CATTTGCTTGGCGAAACTATCTCTTCTGCAGCTGCCAGCAGCTGCTTTAATCCAGATACT

ACTAACAGTGCGATCTCAACAGCAACCACCCCTGCAACCTTTACTCCAGCCCCTGTTTAC

TGCAGGAGCACAAGCTTCAGCAGCTTGTACCCTTGCTTAACAGAAAACTGGGGAGACTTG

CCACTCAAAGTCGATGATTCTGAGGATATGTTCCTTTACGGTGTCCTCCGCGACGCCGTC

ACCGTTGGTTGGGTCCCATCACTCATGACCGATCAATTTATTGAGCACAGTCTTCCAGCG

GTGAAGCTAGAACCCTCTGAGAATTTGACGATATCCCCTTCGCGCGCGCAGGCTCCGATA

GCTGAGCCGGCGGTGGTTCCGTCCAAGGGGAAGCATTACCGGGGTGTGAGGCAGCGGCCG

TGGGGGAAATTCGCTGCGGAGATTAGGGACCCGGCGAAGAATGGGGCCCGGGTTTGGCTG

GGGACATTCGAGACGGCAGAGGATGCAGCGTTGGCTTACGACCGCGCTGCCTATAGAATG

CGTGGGTCGAGGGCGTTGTTGAATTTTCCGTTGAGGGTGAATTCCGGTGAACCGGATCCG

GTTAGAGTGACTTCGAAGAGGTCGTCGCCGGAGCCATCATCGTCTGTGGAGAGTGCGTCC

CCAAAGAGGAGGAAGAAGGTTGGTGGCGCGGCGGGTAATGCTACAGTGATTGCTCAAGCT

GGGATGCAAATGACGAATGGGGTTGGGTATCAAGTGGGCACACATGGCGAACAATTATTG

GTTGCTAGATAA

>EVM0043703

ATGAGTCTTCTATCCCCTAATGAATTAAAACCCATATTTATCTCCAACCCCACCATGTCT

GTTCAGCCAAAAGTCAAATTTACTAAGCATGTACTCACCACTAATAAACTCATCATGAAC

CAGCAGCTGCATCCTTCTTCAAGAACCCGCCAAAGACTTGTTAGAATCATCCATACGGAC

CCTGATGCCACTGATTCCTCCAGCGATGAAGAAGAGGGAGCACAAGAGAGGAAGTATGTC

AAGACAGTGAAGCGACAAGTGAGTGAAATAAGCTTTTTAGAGCAAGAGAAAACAGCACCG

GCAGCTCCTTCCTCCACAACTTCAAATGATGATCAAGAACCAAGAAGAAAGAGACCTTCT

TCAAGATTACCGGTGTCTGACGTCTCTCGTCAGAAAAAGTTCAGGGGGGTCCGTCAGAGA

CCCTGGGGAAAGTGGGCTGCCGAGATCCGTGACCCGACCCAACGGAAACGTGTCTGGCTC

GGCACTTTTGACACTGCAGAGGAAGCTGCTACAGTGTATGATCGCGCTGCGGTTAGACTA

AAAGGACCCGACGCCGTCACCAACTTTCGTACTAACACTGCTACAGAGGAGAGAGCTAAC

GTTCACTCCGGTTCAAGTGATGTGAAGTGTGAACCATCTGATTCGCCGTCTAGCAGGGGT

ACCGCAACCGTTTTGGCATCGCCCACATTAGTCCCCCGTCAAGTAGAGGTGACACCGTTT

GACCTTGTTTTAACGGAGAAGGCTAAGGTTGTTGACGGTCAGCGCGAGTCATGTGAGTCG

TCGTTAAGTGGTGTTAACGACATGTCATCACCAACGTCTGTTCTCCGTTACTTAGAGTTC

ACACTGTTTGATAGTTTGGGTTACTGTGACGTTGACGCTTTTGGGTTCGAAATTGACGTG

CCGTTAAGTTTGCCAAATTCTATGTTGTCGGGGAAATTCTTTGGCAAGGAAGAATTCGTT

GACCTCGATGATTTCTTGGTGGATGCCATCTGTTAA

>EVM0055401

ATGGATGCAGCAAAGAGTAATATTGGAAAATCCAAGAAGGGTATTGTTGATGAAACTCGA

AAGATGACGATGGAAGAAGATCGGCAGCTTGATAGAGGCAAAAACGAAGCTGATGTTAGC

TTCGAGAAGAGGCAATGGAAGCCGGCTTTTGGTGATGCTTCCCTGTCGAACAGGCCTTTG

AAGAAAACCTGTAGCCCTGTACGTCAAGAACAGATGCACTCCTCTGCATCTTTAGCTCAT

CAACTGCCATCTTGTTTCTCTGTTTCTTCTAGCTCTGCCTCGACTCTGTCGCTGTATCCG

CCTTCTTCGTCGCTTTCACCTATCTCATCTTCAACTTCTTCAAGTTCCAGACTTGTGTTT

CCTTTTGCATTTGAAGGATCTAATCAACCTATTCAGTTCCCTCAACAATTTAGAACATCC

CCTTCTTTGCCTGTCTTCCATCCACTGTCTCAAGCTGCACAGAATCAACAGCAAATGATT

TCTTTTGGTCAAAACCAGCAGCAACATGGCATTGCATATCCTCCGTTTTTAGCTGGAGGA

TTAACAATGGCTGACCAGCAGCAGCAGCTGTTTCAGTACTGGAGTGATGCATTGAACTTA

AGTCCGAGAGGAAGGATGATGATGATGAACAAGCTGGGGCCGGATGGGAGGCCATTGTTT

AGGCCTCCGATTCAGCCTATAAACACAAGAAAGCTTTATAGGGGAGTGAGACAAAGGCAT

TGGGGGAAATGGGTTGCCGAGATTCGTCTCCCTCGAAACAGGACTCGCCTTTGGCTAGGG

ACATTTGATACAGCAGAAGATGCTGCCTTAGCTTATGATCGCGAGGCCTTCAAGTTAAGA

GGAGAGAATGCTAGGCTGAATTTCCCTGAACTTTTCCTCGGCAAAGATAAAGCAACTTCC

ACAGCTCCAAATTCAACAGTTTCTTCTCCGCCGACTCCCAATCAAAGTACAAAGCCAAAA

CCAGCCGAAGAAGGCCTTAACTTGCAGGCAGAAACCATGTCACCACCAATATTACCACCA

CAGCCACCGCCAGAGCAACCTCCAGGAGACCATCCTGATGATGATTGCGGGATGGGTTCG

AGTGTGGCTACGGCGAGTGATGAGATTCAGGCAGTGGCAGAGGGGTCTAGTGCAGGGGAA

GGCATTTTGGGGTCTCAAGAAATGATGTGGGGAGACATGGCAGAAGCTTGGTATGATGCT

ATTCAAGCAGGTTGGGGTCCAGGGAGTCCTGTGTGGGACGATTTGGACTCCACTAACAAT

CTTTTATTACAATCACATCTTCCTTTTGTTAATCCAAATCAACAGCAGTTTAATGATTCT

TGTGTTCTCCAAGACAACATGGGCTCATCTTCATCTTCTTCATCCTCCTCTTTCTTCCCC

GTGAAATCATTCTTCTTGAAGGATCAAGATTGA

>EVM0006682

ATGGCTCCCAGAGAAAAACCTAACCACAGCAGCCCAAATATCCTCGGATCAGAGATCCGT

TACAGAGGCGTCAGGAAGAGACCATGGGGACGTTACGCGGCTGAGATCAGAGACCCTGGC

AAGAAAACGAGGGTGTGGCTTGGCACTTTCGATACCGCTGAAGAGGCCGCGCGTGCATAT

GATGCGGCAGCTCGCGAGTTTCGCGGAGCCAAAGCCAAAACTAATTTCCCTACAATCGGT

GAGCTCATTCCCGCCTCAACTCGCAGTCCCAGCCAAAGCAGCACTGTAGAGTCCTCCTCC

CCTCCGCACCCACGCGCCGCTTCTCCCCCTCCACAACTCGATCTCACTCTTAACACTGCG

CACCATAAGACCACCCGCCATCAGTTCCCCGACGGGGTTAGTTTCCCTGGAGGCGCGTGG

TTCCCACACCCTGCTGCCCAGCGCCCAGTTTTCTTTATAGACGCGTTTGCACAGGCGAGG

AATAACAGCAAAAATAGTATAGTGAATAATATGAGCATGTGCATGTTTGACCGGGCAGCG

ATGGTGAATGGAGGTGGGGTCCAGAGCGATTCGGATCCATCATCAGTCCTTGATTATGAT

CATCATCATCATCATGATAGTAAGGGGCTATCACTTGATCTTGATCTAAACCTTCCCCCG

CCACCGGAAGTCGCGTGA

>EVM0047172

ATGGCGGCTGGAAAAAAAACCCGAAGAAAAAACCCCCACAAAGAAAAACACTTGGCTCGA

TATTGGCAACTGCTGTTTCATTCAAGTTTCATGTGCGTATTGAAGGTGGCGAACCCAAGA

GGAGACTCAGGGGAGTACAGAAGGTACCCTGCAAGAGATAGCGATGATAACCAGGAAGAG

AGATCATACACAGCACAGCAAATATTTTCCGAATTGAATCCACCAAGACAGCGGCGTGCA

GAGGTGGCTCAGCCACCTCAGATGTTCACGGGGTATGGCAACTCATCAGAGATGTCCGCG

ATAGTATCAGCACTTACACATGTGGTGTCAGGCCACAGAGAAAGCACTAGCGATTGGGGA

TCATATGAGCCTGCAGGTGAGACAATAACGTCAGCTTTTGTTCCGGCGGCGAGTGGTTCT

AACGCTACTCCCGCTTCTCCACCCTTGTCTGCTTATTCTTCAGCTTCTGGCTCTGGTTCC

TGGATTGGCCAGAAGAGAGGGAGAGAAAAAGAACCCGGTGCAGCAACTCCGTTGAAGGAG

TCTTTGCCAGGGGTTTACAGAGGGCTTAATGGTGTTAGAAGTTCACTGGGCGATTCGTTG

TCATCTGGTGCAGCAACTGAAACTGAAGAAGTGTCTGCTTCAACTTCAGTATTCCCGGCC

ACAGCCACACCCTCCGCCATAGCACCGCCATCAACTGAAACAGCATCTATGGGCGAAACT

GGAGAACGGAAAATAAGATACAGAGGAGTCAGGCAAAGGCCATGGGGCAAATGGGCAGCA

GAGATACGTGATCCACACAAAGCAGCGAGAGTCTGGCTAGGCACATTTGAAACTGCAGAA

GCTGCAGCTAGAGCCTATGACGAGGCTGCTTTAAGATTCAGAGGAAACAGAGCTAAACTT

AACTTCCCTGAAAATGCCAGGCTACCACCAGCTCAAACGCAGAATGTTACTGCTTCTCAA

GCTCCCGTTTTCCCTTCACAATTACCTTCCGGCCACCTGTTTCAGTCAATCTCATCTCCA

AGGCAGCAAGCACAGCGACCGCAGGTACCAGCACCTGCATTGTTTCAGTCTCAGCCTGAT

ATTATAAGAGACTACTGGGATTACTCCCAGCTGTTGCAGAATTCTGGGGACTTCCTTGAA

CAACAACAACCACCTTCAAATTTGTTACAGCAGATGTTTTATAATCCCCAGCTGGCTTCT

CTTCAATCATCAACCTTCTCTTTATCTCCTTCATCTACTTCAGGTTCTTCATTTGCAGAA

ATATCTTCCGGTTCGTTATCATCAACACTACCTCCCTCTGCTTCTTCGTTTCCTCTGCTT

TTTGCTGGTCAACAGTTGGGTTATTTTCGGTCACCACAAAACCAGAATCCAGCTGCTAGT

TCCGATTTTCCGGTGCCCCCGTGGACAGATTCTAGTCCCCGCCATCCTTCCTCCACTGGT

TAA

>EVM0053799

ATGAGGAACTGGGGCAAATGGGTGTCAGAAATTCGTGAGCCACGCAAAAAATCTCGCATT

TGGCTAGGGACTTTTCCAACACCAGAAATGGCAGCGCGTGCGCATGATGTCGCTGCTTTA

AGCATCAAAGGAAAGTCAGCTATTCTTAATTTCCCCGAACTCATCAATTCCTTACCTCGA

CCAGCTTCACTTGCTCCTCGTGATATTCAAGCTGCCGCTGCTAAAGCTGCTCAAATGGAC

AAGTTTGACAAAAAATCTGAAACAACCACAGCATCTCCATCGTCCTCTTCGACTTCTTCT

TTGACATCTCTTGTTTCTTTGATGGACTCGTCGTCTCAAGAGGAAGAGCTGTGTGAGATT

GTTGAGTTGCCAAGTCTTGAAACGAGCTATGATGAGTTGAAAAATGATTTTGTTTACGTT

GACTCTATTGATGGGTGGATGTATCCTCCGCCATGGATGCAAAGCATGGAAAATCACTGT

TATGGCGGCGGTGGCGGGTTTGCTTGCGATGATTTTACATTTCCGAATGAGAGTACTGTT

TTATGGAATTATTAG

>EVM0056893

ATGGTGCAGTCAAAAAAGTTCAGGGGGGTGAGGCAGCGACATTGGGGTTCCTGGGTTTCT

GAGATTAGACATCCTTTACTGAAGAGGAGGGTATGGCTAGGCACATTTGAAACAGCAGAA

GAAGCAGCAAGAGCCTATGATCAAGCAGCAATCCTGATGAGTGGTCGTAATGCGAAAACT

AACTTTCCAATACCTCAAACTAATTCAAACGAGGAGGACCCAAAATCAAGTGATCAGCCT

TTACTCCCAGCACCACCAAATGGTTTGTCTGAGATCCTTCATGCTAAGCTAAGAAAGTGT

AGCAAGGCACCATCACCATCGATGACTTGCTTGAGGCTTGACACTGAGAATTCACTTATC

GGGGTTTGGCAGAAGCGTGCAGGTGAACGTTCTGACTCAAATTGGGTCATGAGAGTGCAG

CTTGGACAGAGAGAAAGCCAGGTTTCTGAGAGTACACTGCCGTTGCCTGAGTCTTCAGGA

GGGGTATCAAAACCAGAATTGAGAGCAGAAGTGGGTGAAGAAGAGAGAATTGCTCTGCAA

ATGATTGAAGAGCTTCTCAACAGGAATTGTCCTAGCCCTTCTTTTGGGGTTCAAGATCAC

GGTGATGGTAGCCTTTTTCCTTAG

>EVM0054439

ATGGAAACACATCAAGGAGAGACGAGCACCAAGAAGCAAGAGAAAGGAAGAGGGGAGAAT

GCGTATAGGGGGATTCGCAGGCGACCTTGGGGTAAGTTTGCTGCCGAGATACGTGACCCA

ACAAAGAACGGGGCCCGTCGTTGGCTTGGAACATATGACACGGCGGAGGAGGCGGCTAGG

GCATATGACCGAGCTGCTTTTGCCTTCCGAGGTCATTTAGCCATTCTCAATTTCCCGAAT

GAATACCAACATCAGGATCCAAACTCTGCCATGTCATGTGGTTCTTCATCTTCATTCTCC

ACTGCCGATCCTGTGAATGATGGCCATGAAGTTTCCTCTACTGGTGGGCAAGAAGTTATA

GAGTTTGAGTATCTGGATAACAAATTGTTGGAGGAGCTGCTGGGAACGAATGATCCCAGA

AGGCAGCATTAG

>EVM0001248

ATGGAATCTTCTTTGCTTTACCATGATCAAAACCCGAATTCTGATTTCTCCCCTGAATCC

TCTTTTGGTTCTTTAGATTCATTTCCATGGGATGACCTTCTTTTCGAGAGCAATTCACTT

CCTTTTAGCACCAATGACTCCGAGGAGATGCTCCGTTCTAATGTCTTGGCCGATGGAGCG

CCCAAGGAATCCACGGAATCTAATTCCTCGAGTGGAATCAAGGAAGAGGAAGTGACTTCA

AATGCGAAAGAAGAGGAAGCGGCTACGAAAGAGAAATCTTACAGAGGGGTTAGAAGGCGG

CCATGGGGGAAGTATGCTGCAGAGATAAGGGATTCTACGAGGAATGGGGTTAGGGTTTGG

CTAGGGACCTTCGATAGTGCAGAGGCAGCTGCTTTGGCTTATGATCAAGCAGCATTTTCC

ATGCGGGGTTCCATGGCTGTTCTCAATTTTCCGGCGGAGATGGTGAGAGAGTCACTTCAA

GACATGGATTGTGGATTCGAAGATGGGTGCTCGCCTGTGATCGCACTGAAGAGGAAACAC

TCAATGAAAAGAAAATCAACGAGTAAGAAAAGTAAAGTACATCAAGTACCTTATTCTAGG

CCACAAAATGTGGTGATTTTGGAGGATTTAGGAGCTGACTACCTGGAAGAGCTTCTGAAT

TCATGTGACAGTTCTAACTACTGGTGA

>EVM0008755

ATGGATTATTCTTTTTTCAACCACCCAGATATAGAATTTTGGCCATCATCTCCCTCCAAC

TCTCCAGATCTATTGCAGGATGACCTTCTTTTTAACCCTCATGATGATTTCTTTCTATTC

AGCTCAAATGATTCTGAAGAGACACTTCCATATGATGCGCCTTTCACCGAAGCCAAAGAA

TCTGCTGATAGCATTGCCTCCGATGCTCCACCGAATAACGAAGCCAAGGACTTATCGGAA

CTTGTTTCCTCGAATGAGATCGAGGAAAAAGAAGCCACTCCGAATGCTAAAGAAGAAAAT

CCCGGAAGGGAAAAGACCTACAGAGGGGTCCGAAAGCGGCCGTGGGGAAAATATGCGGCG

GAGATAAGAGATTCCACTAGAAACGGAGTGCGAGTTTGGCTCGGCACTTTCGATACTGGT

GAAGCAGCTGCTTTAGCTTATGACCAAGCTGCATTATCTTTACATGGCTCGAAGGCTATC

CTCAATTTTCCGATCGAGAAAGTCAGAAAATCATTAGGAGAAATGAAACGTGGTTTAGAA

GATCGCTGGTGCTCCCCTGCCGAGGCACTGAAGAAGACTCATTCGAGAAGAAAGGCAGTG

AGTCGGAAAGGGAAAGGGAAAGGGAAAGGGTTAGCAACCAAAGAAGTGGTGGTGTTCGAG

GATCTAGGAGCTGAATATCTGGAAGAACTATTAAGCTCTTGTGAGAGAGGTACTCCTCCA

TGA

>EVM0011531

ATGAAGTCCATGAACAATGATGATGATAGCAATAGCCATAATGCAAACTGGTTAGGCTTC

TCTTTGTCCCCTCAATTGAAAATGGAGGTTCCATCTGGTGCTAATCACCATCACCAAACG

CAGTCTCCTTCTGCGGCTATTCCTACTGCAATCCCATCAGGTTTTTTCCACTCCCAGATA

CCACAACTCAACTATGGCATTTACTATGGCGTTGATGATCAGAGTGAAAATGGAGGGTTC

TACTCTCCTTTACCTGTTATGCCACTCAAGTCTGATGGCTCACTTTGTATGATGGACGCT

CTCACAAGGACACAGCCTCAAGGTGAAGCAGCAATGGTAACTACTTCAACACCAAAACTA

GAGGACTTTTTTGGTGGTGCAACTATGGGGACCCATCACTATGAAAGCAACGATAGAGCA

GCTATGGCTCTTAGCTTAGACAGTAGTCCTAGTATGTATTTTCACCAAGACCCAGACCAT

GAGCCCGACAGCCAAAATAGTCTAAATCATCTTCAACAAAACCCCAGACAGCAACAGCAT

CACCACGTTCAACTTCAGCACTACCCGTATTATTCTTACTTTAGGAACAATGAGATGTTA

GTTGGAGAGGAAGCAAAACAAATGGCTCAGGCGTCAGATTGCGGTCTTAGGCTTCCTAAC

ATGGGAAATGAAGGGATAACTGGCATGAAGAATTGGGTTTCGAGAAACTATCAAGGTAGC

CGTGCCATGGAGCAGAAGATGCTTAGATCCATGGTAGAAAATGGAGGCGAATCCGGGCCA

AATACCAGTGCAATGACATATGGGGATTTGCAGTGTTTGAGCTTGTCAATGAGTCCTGGC

TCCCGGTCAAGCTGTGTTACTGGTTCTCTGCAAGTCTCACCTTCTGTTACTGACTGTGCA

GTCATGGAAATCAAGAAAAGAGGACCTGAAAAGGTGGATCAGAAGCAAATTGTTCATAGG

AAGTCTATTGATACATTTGGGCAAAGAACTTCTCAATTTAGAGGTGTTACAAGGCATAGA

TGGACTGGGAGATATGAAGCTCATTTATGGGACAACAGCTGCAAGAAAGAAGGTCAAAGC

AGGAAAGGAAGGCAAGTTTATCTAGGGGGTTATGATATGGAAGAAAAAGCTGCAAGAGCT

TATGATCTAGCTGCACTCAAGTATTGGGGACCCTCAACTCATATCAATTTTCCTCTGGAA

AATTATCAAAAAGAAATTGAAGAAATGAGGGACATGAGTAGGCAGGAGTATGTTGCTCAT

TTGAGGAGGAAGAGCAGTGGATTTTCTAGGGGAGCTTCAATGTACAGAGGAGTTACAAGA

CACCATCAACATGGAAGATGGCAAGCTCGGATTGGTAGGGTTGCCGGAAATAAGGACCTT

TATCTAGGGACATTCAGCACCCAGGAGGAGGCCGCTGAAGCCTATGATGTGGCTGCTATA

AAATTTCGTGGAGTTAATGCTGTAACCAACTTTGTGATAACAAGATATGATGTGGAACGA

ATCATGGCTAGCAATACACTTCTAGCTGGAGAATTGGCCAGGCGAAACAAAGACATAGGA

CCTTGTGATGATACTTCTACCAATCACAGTATTCCTTCTACTCGTAACAGCAATGGGGAT

TCCTTGGCCTCTCAGAAGAACAGTGAAAACGAATCAGATTGGAAAATGGCTCTCTATCAG

TCATCACAGCAACTGGATCACAAGGCCTCGAATGTCATGGATAATTACAAAACTCAGGCT

TTCTTATTGTCTCCCGAAAATGTAATTGGATTTGATTGCATGAGCTCAGCTCACCAGCAC

GAGTTAGAGGACCCGTCCAAGATGGGAGCTCACGTGTCAAATGCTTCTTCATTGGTGACT

AGTTTAAGTAGCTCTAGAGAAGAAAGCCCTGACAGAGCAAGCCTACCAATGCTCTTCGGA

ATGCCTCCCTCTGCATCATCCAAGTTGTTCGCTAGTCCAACTAGCGACGTGAACTCTTGG

ATCCCAGCAGCATCTGCCCATTTGAGGCCTGCAGTATCACTGCCTCACGTCCCAGTTTTT

GCTGCATGGACCGATGCGTAG

>EVM0049893

ATGGAAGGAGGGTGTTGTACATCGTCAACAAATACTTCAAGTTCAACTGCAACAGCAACC

ACACAGAAACGGAAGCATAGCAGGCAACAAAACCAAGAGAAGCCATATAGAGGGATAAGG

ATGAGGAAGTGGGGTAAGTGGGTTGCTGAAATTCGAGAGCCAAACAAGAGGTCCAGGATT

TGGCTTGGGTCCTACTCAACACCAATAGCTGCAGCTCGTGCATACGACACGGCTGTTTTC

TATCTGCGAGGTCCTTCTGCTCGGCTTAATTTCCCTGATTTGATATACCAAGAAGATGAG

TTGCGAGATGTGTCTGCTGCATCTATACGCCAGAAAGCCACTGAAGTTGGGGCTAAAGTT

GATGCCCTACAAACCGCTCGCCATGCATCACCAGAAGATAACTCACGAGTGTTACTTTCA

GAGAAACCTGATTTGAACAAGTACCCAGAAAATTCAGATGAAGAGTGA

>EVM0035393

ATGGCTAGGAAGAGAAAGGTCAGTGGAAGTGCTGAGGACAAGAGCTCTGGTGAAGGAACC

ATGGCTTGGGATGAGATGGTCATGGAAGCTGATGCAGCAACAGCACTAGGAGGAGCTAGG

AGAGCTAGAAAGAGATTTGTTGGTATTCGACAAAGGCCATCGGGTAGATGGGTGGCTGAG

ATTAAGGACATCATACAGAAAATTAGGGTGTGGTTAGGCACCTTCAATACTGCTGAGGAA

GCAGCGAGAGCCTACGATGAAGCTGCTTGCTTGCTTCGTGGTGCTAATACAAGAACAAAC

TTTTGGCCTTCTTCTCCAATTTCTTCAGCACCTCCAGCTCTTCCCTCGAAGATTACTAAC

CTTCTACTCCAAAGGATTACAGCAAGAAACAAATCTTGTGATCCTTCTGCAACCGCTCAG

CCCCACAACCAGCAGCAAAAACTAGTGGAGGAATATAGAGAACCAGCATCCTATTTCTCA

GACACCCAATTCACTGGTTTCCTCGACCATACAGACGATTACTCCATGCGCAACGATAAC

ACCAATAACACTATTGCTAGCTCATTTGATTGCACGACAACAAGTATTGTGCCATGCTTA

GCAGACAAAGAAGATAGCTCAGGAAAAGAATGGGACTTTGACTATAGCTGGAGTGATGTG

GCAGAGTCATCCAGTGGTGATGGTAACAACTTGGGAGGTGAAGGAGAGGGAGAGGAAGCA

GGGAACGACATAGGAGCGTTGATGATGTTGGCTTTTGAGATTGCTGAAGAGATAGAGGAG

CCAGTGGAGCCTGAGTGCTATGGAGATGAGTCTGCTATGCTTAAAGCAGCCATGAAGAGA

ATGAAATATGAGCGGAAGTTCTCAGCTTCTCTCTATGCTTTTAATGGGATTCCTGAATGC

TTAAATCTGAAGCTTGGATCAGGAAATGCCAAGGGAAATGGACGTTCTGATCAGTTAGCC

AAACTTCGAAATGCATGTAACAGTAAAAAGGATGAGAGTAGAGCAGAAGAAGAAAGGTTG

GAAGTGATGCAGAAACAGGATGATTACTCGCAAAGCTCAATGGAAATGAAATCTTCTTCC

GCTTCTAAGATTAATGATGGCGAATTGTCACTTTGGAACACTCTTGATCTACCGCCAATC

TGCTTCATAAACTAA

>EVM0012647

ATGCTGACACACAGCCCCAACACGATGGCCATACAAACAAAAGTCAAATTTACTGAACAT

GTATTCGTCACTAAAAAGCTCATCCAGAACCAGCAGCAGCATCCTTCTTCAAGGATCCGC

GGTCAGAGAATAGTTAGAATCATCCACACCGACCCTGATGCCACTGACTCCTCCAGCGAC

GAAGAAGAACAAGATCAAGAGAGGAAGTTTGTCAAGAGAGTGAAGACACGAGTGAGTGAA

ATAAGATTGTTAGAACAAGAAACAACAGCACCAGCAGCTCCTTCTCCAAGAACTTCAAAT

GATCATCATCGACACTCGACAAGAAAGAGGCCTTCTTCAAGACTACCGGTGTCCGACGTC

ACTCGCCGGAAAAACTTCAGAGGTGTCCGTCAGAGACCCTGGGGAAAGTGGGCTGCCGAA

ATCCGTGACCCGACCCGACAGAAACGGGTCTGGCTAGGCACTTTTAACACTGCAGAGGAA

GCTGCTACTGTCTATGACAGGGCTGCAGTGAAACTAAAAGGTCCCGACGCCGTCACCAAC

TTTCCTATTAAATCCGTTATGACGGTGAAAGCTAACGACGATGGTCCAGACGTCCAGTGT

GAGTCATGTGACTCACCGTCTAGCAGTGGTGTTAACGTTATGCCATCACTCGCGTCAGTC

CTCCGTTACGAGGAGCTGGCGCCGTTTGATCCTGTTTTGACCGAGAAATCCAAGGTTATC

GACGATCACCGCGAGTCATGCGAGTCACCGCTGAGCGGTGCTAACGTGATGACTTCACCA

ACGTCTGTTCTTCGTTACGAAGAGTTGACGCTGTTTGATAGTTTGGCTTACGGTGACGTT

GACGCTTTTGGGTTTAAAATTGACGTGCCGTTAGGTTTGCCGGACTTTATGTTGCCGGTG

AAATTCGTCAACGATGAAGAATTCGTTGACCTCGATGATTTCTTGGTGGAGGCCATTTGT

TAA

>EVM0029842

ATGGCTGCAGCGATAGATATCTACAACACAGCAGTACCAGTTTTTTCAGATCCTTATAGA

GGAGAACTCATGAAAGCACTCGAACCTTTTATGAAAAGTGCTTCACCATCACCATCTTCG

ACTTCTTATTTTTCACCATCTCCTTCGACTTCTTCTCATTCTTTCTCCTCTTACCCTTCT

TACAATAGCTTCCCTGTCACTTCACATCCCAATTTGGACCTTAACTTTTACACTCCAACG

AGCACCCAGATGTTTTCTAAGGGGTTCTCGGGTTATAACCAAATGGATTTTGAGCAAAAA

GGTCCAATTGGGCTTAATCACCTTACCCCTTCACAAATCCTTCAAATCCAAGCCAAAATC

CACCTCCAACAGCAGCAGCAGGAGCAACAAATGGCAAATCATTCTCCCTCGTCGCAGTTC

CATAACCAAAGGACTGGCAACTTCTTGACTCCAAAACCTGTCCCTATGAAACAACAATCT

GCTTCTCCTCAAAAGCCAACGAAGCTTTATAGAGGAGTGAGGCAGAGACATTGGGGAAAA

TGGGTTGCTGAGATTAGACTTCCAAAGAACAGAACAAGGCTCTGGCTTGGCACCTTCGAC

ACAGCTGAGGAGGCAGCTTTGGCTTATGATAAGGCAGCTTACAAGCTTAGAGGAGAATTT

GCCAGGCTCAACTTTCCACATCTTCGCCACCAAGGAGCTCATGTGTCTGGTGAGTTTGGT

GACTACAAGCCTCTCCATTCCTCTGTTGACGCGAAGTTGCAAGCAATTTGTCAGAGCTTG

GGCTTGCAAAAACAGGGGGAAACAGGGGAACCTTGTTCTGTTGCTGATTCAAAGAAGACC

GTTTCAGCTCCTTTGCAGGCAAAAATTGAAGATGATTCTTCTTTGAAAGGTGAATTGAAA

AGGGAGTTTGAGAATTCTGGAGTTGAGGAATTTAAAGCGGAGATCCCATCACCCTCACCA

GCTTCATCTGATGAGTCATTGGCCGGTTCTTCTTCACCAGAATCTGAGATTTCTTTCTTT

TTCTCTGATTCCTTACAGCGGGACGAGTTTGAGAATTTTGGTTTGGAGAAGTATCCTTCA

GTTGAGATTGACTGGTCATCTATCTAA

>EVM0018160

ATGATGAAAGAAGAGAGCGGTGGCAGGTCAAGGAATTCAGTATGTACATCGTCGTCGACG

TCTTTCTCCGCCGCCGCCACCCACAAGCTCGAGAAACAAAACCCAGTTAGCCAAAAATCG

AGAAAGGCAAGAGACTGCAGCAAGCACGCGGTTTATAGGGGTGTTAGAAAGCGAGCCTGG

GGGAAATGGGTGTCCGAAATCCGCCAGCCTCGTAAGAAATCACGTATCTGGCTCGGAACA

TTCCCTACGCCAGAAATGGCAGCTCGTGCTCACGACGTGGCTGCCCTGAGTATTAAAGGT

AACTCTGCCATTTTGAATTTTCCAGAACTTGCTGCCTCATTACCGCGCCCGGTGTCATTA

ATGCCACGTGATATTCAGACAGCTGCGGCTAAAGCTGCTGCTATGGTCGACTTTAATTCA

TCGTCACCACCCTCATCACTGTCGTCGTCGTCGGTCTCGGTTTCTGGAGATGTGGCAGAA

TCCGAGGAGGAATACTTGAGTGAAATCGTGGAGCTACCGAATATAGAAGGAAGCTTCGAC

TCTCCTGACCAGTCACAGCCTGAGTTCATGCTGTTTGACTCGGTTGATAGGTGGGTGTAT

CCACCACTAGACTTGAGTGGAGAGTTTTCTGATCAGTTATTGTGCCTGGAGAGCTTGATG

TCTAGCAACTTTGGAGGATCTTTGTTGAATTAA

>EVM0051611

ATGGCGGTAGACGCGTGGCCCCACCGTAACGACACTGTCACAACAACCCTCTCCCTTGCA

GGACCTCCAGCTTCCCCCACACCGCCAGTTAGGCCGTGGGGTCGATTCGCTGCTGGGATT

CGGGATCCATGGAAGAAGACGCGGCGTTGGCTTGGAACTTTTGACACGACCGAGGAAGCG

GCACTTGCTTATGACGAGGCAGCTCGATCTCTCCGCGGACAAAAGGCAAGGACAAACTTC

TTCCACCGTGACCATCTTGCTCCCCTGCCTTATTCTCAAACAACGGTGGCTGGTGGCGGT

GGTTTAAGGATATGGTACTGCTCGCCGGTCTGTCTTTGTGATGAAGTGTCAAGAATGGCT

GCGCCAACGGCGGTGGATCCGGTGAGGTCAGAGTATCAGCGGTACAAGATCGAGAATGTG

AATGAAAAGATGGGTTTGGTTTTAAATGAAGAGCAGCAAAGGCCGTTGTTGTTTGATTCG

AACTTGCCGGCTCCAATCTTTTGA

>EVM0052364

ATGGACCATCCTAATGTAGGAAAGGTCAGCCAGAAAGGTGGAGAAATTAGATACAGAGGC

GTTAGGCGGCGGCCGTGGGGGAAATTTGCAGCGGAGATACGTGACTCGACAAGGCATGGA

GCAAGGTTATGGCTAGGGACTTTTAACACAGCAGAAGAGGCTGCGAGGGCTTATGATAGA

GCTGCGTATTCAATGAGGGGACCTTTAGCAGTCCTTAACTTCCCTGGTGAATATCCTAAA

ACTAAGGTTGTTTCCGATGTAACTTCCTCTTCCATTTCTTCTTCACCATCACCATCATCA

TCATCTGCTTCATCTTCTTCAATGTCACAGGATGCAGAAACAAGTGGGATTGGACGAGGA

AAAGAAGTCTTTGAGATTGAGTACTTGGATGATAAGTTATTGGAGGACCTTCTTGATTTT

GAGGAAGGAACTAGCAAGAAATCAGAGATACCGCATGGTTTATACAAAAAAAAATCTCTC

TTTTCTAACTTTCAAAATAAAAATTAG

>EVM0017235

ATGGAAACTCACCAAGGGGAGAAGAACTCGAAGAAGCAAGGGAAAGGAAGAGAGAATGCG

TATAGGGGAATTCGCCGGCGACCATGGGGTAAGTTTGCTGCTGAGATACGTGATCCGACG

AGGAATGGAGCACGTCGTTGGCTCGGAACATTTGACACAGAAGAGGAGGCGGCCCGGGCC

TATGACCGAGCTGCTTTTGCCTTCCGGGGTCACTTAGCCATCCTCAACTTTCCGAACGAG

TACCACCATCAGGATCCAAACTTTACCAAGTCATTTGCTTCCTCGTCTTCGTTCTCCACT

GAGAATCCTTCGAGTTATGGCCATGAAGCTTCCTCCACCGGTGGACAAGAAGTTATAGAG

TTCGAGTATTTGGATAACAAATTGTTGGAGGAGCTGCTGGGAACACATGATCACGGCAGG

CAGCTTTAG

>EVM0008128

ATGGCACATTCTTTGTTTTATCATAATCAATACTTGAGTCCAGATCTCTCCCCTGAATCT

TCTTTCGGTTCTTTAGATTCATTTCCATGGGATGATCTGCTTTTTCAAAGCAATTCCTTT

CCTTTTAGCGTTAGTGGCTCGGAGGAAATGGTGCTTTTTGATGTTTTAGCCGACGGTGCC

AAGGAGTCCTCGGAATCTAACTCCTCGAGTGGGATTAAGGAGGAGGAAGTGACTTCAAAT

GTCAAAGAAGAGGAGCCGAAGAAAGAGAAATCCTACAGAGGGGTTAGGAGGCGGCCATGG

GGTAAATATGCTGCCGAGATAAGGGATTCTACAAGAAATGGCGTCCGAGTCTGGCTCGGA

ACCTTTGATAGCGCCGAGGCTGCTGCTTTGGCTTATGATCAAGCAGCATTCTCCATGAGG

GGTTCGATGGCTGTTCTCAATTTTTCTGCGGAGATGGTGAGAGAATCACTGGAGAAGATG

AAGTACAGATGCGAAGATGGGTGCTCGCCTGTGGTGGCACTCAAGAGGAGACACTCCGTG

AGAAGAAAATCAACGGGTAGGAAAAGTAAAGTGAATCAAGCGGCCAGTACTAGACAGCAA

AATGTGGTGGTTTTGGAGGATTTGGGAGCTGACTACCTTGAAGAGCTTCTGAATTCATGC

GAGAGCTCTAGTTCTTGGTGA

>EVM0018983

ATGGATCATTCTCTCTTCAACAACCCAAATCAAGAATTCTCAACATATTCTCCCTTCGGC

TTTCTGGATCCATCGTGGGATGAGCTTCCTCTTGACCTTCATGACTTCTCTCCAATCAAC

TCAGATGACTTTGAAGAGAGTCATCATCAAGAGACTCTTCCATCGGACGTTCCATTCACC

GAAGCCAAAGCATCTACTCACAATATTACCTCGAAAGTTCCACCAAACATAGCCAGAAAT

AATTCACCGCAAGTTAGTTCCTCGAAAGAGATCAAGGAACAGGTAGGCGCCCCTCCGAGA

GCTGAAGAAGAAAATCCCACAAGGAAAAAGACCTATAGAGGGGTCCGAAATCGGCCGTGG

GGAACATATGCAGCAGAAATAAGAGATTCCACCAGGAACGGCGTGCGAGTTTGGCTGGGC

ACATTTGATACTGGTGAAGCAGCTGCTTTAGCTTACGACCAAGCAGCATTATCATTACAG

GGCTGTAAGGCAGTCCTCAATTTTCCGGTCGAGCAAGTGAGAGAATCACTCGGGGAGTTG

AAGTGTGGTTTTACAGACCGGTGCTCGCCAGCTGAGGCACTGAAGAAGACTCACTCCAAA

AGAAAACCAATGAGTAGTAAAAGGAAAAGCAAAGGGTTAGCAACCAAAGAAGTGTTGGTG

TTCGAGGATCTCGGAGCTGAATATTTGGAAAAACTCTTGAGCTCTTGCGAGAGAGGTATT

CCTTAA

>EVM0027529

ATGTTGGATCTTAACTTGACTGCGGTCCCTGGTGATTCTTCAACGACAGTGAAGAGGAAT

AATAATAGTAACAAGAAGATTCTGGAGCTTTCCAACTCTCCAATGGAAAGTATCGGGAGC

TTCAATTCTTCTTCTATAGTCAATGCGGATGGCTGTGGTGATGAGGACTCTTGCTCCAAC

GGCGACGTTTTCGCTCTCAATTTCAGTATCTTGTCAAATGAAAGCAGCTCCAAGAAGACC

GTTTATTCTAATAATAATAATGACCGTGGAGATCGAACGATCCAGCTTTTTCCAGTGGAG

TGCGGGACCGAGAATGCAGCAGGAGGCTCTAACTCTTCTTCGGCGGTGCAGATGCAGCGG

ATGGATCTCGGTGGGTCCATGAACTATGGTGTTCCTCCGGAGCAGGGGATTGGGGCTCGA

CAACAAACGCCCGTGAAAAAGAGTCGGCGTGGGCCCAGGTCGAGGAGCTCGCAGTATAGA

GGAGTCACGTTTTATCGGAGAACTGGAAGATGGGAATCTCATATTTGGGATTGTGGAAAG

CAAGTCTACTTGGGTAATTTTGAGATAATATTATTCGATTATTTAAAATTAATTTTGATA

CATTGGTTCTATGGGGTACAATTAATACTTGCTAATTTGATATTAATTTATTTTGTGAAT

CTGGGTGTTTGGTTTCTTGTAGGGGGATTTGATACTGCACATGCTGCAGCTAGGGCATAT

GATCGAGCTGCGATCAAGTTCCGTGGAGTTGATGCAGACATCAACTTTAACGTTAGTGAT

TATGATGAGGATATTAAGCAGATGAGTAATTTCTCAAAAGAAGAATTTGTGCATATTCTA

CGTCGTCAGAGCACTGGATTCTCTAGGGGCAGCTCAAAATATAGAGGAGTGACACTCCAC

AAGTGTGGCCGATGGGAGGCTCGTATGGGGCAGTTCCTGGGAAAGAAGGCTTATGACAAG

GCTGCTATTAAATGCAATGGAAGGGAAGCAGTTACCAACTTTGAGCCAAGCATATATGAA

GTGGAGATACTTCCAGAGCCCAATAGTGGAGATGGCAACCAAAATCTTGACCTGAACTTG

GGAATTGCTCCTCCTGATACTTCTGATAGCCTAAAGTAA

>EVM0039676

ATGGCGGTAGACGCGTGGCGCCACCGTAACGACACTGTTACAACAACCCTCTCCCTTGCA

GGACCTCCAGCTTCCCCCACACCAGTGGTGGAGGATAAACCTCAACGCCACTTCAGAGGC

GTGAGGAAGAGGCCGTGGGGTCGATTCGCTGCTGAGATTCGGGATCCATGGAAGAAGACG

CGGCGTTGGCTTGGAACTTTTGACACGGCCGAGGAAGCGGCACTTGCTTATGACAAGGCA

GCTCGATCTCTCCGTGGACAAAAGGCAAGGACAAACTTCTTCCACCTCTGTCTTTGTGAT

GACGTGTCAAGAATGGCTGCGCCAGCGGCGGTGGATCCGGTTAGGTCAGAGCATCAGGGG

TACAAGATCGAGAATGTGAATGATCAAAACATGGGTTTGGTTTTAAATGAAGAGCAGCAA

AGGCCGTTGTTGTTTGACTTGAACTTGCCGGCTCCACTCTTTTGA

>EVM0027771

ATGGACCATCCTGATGTAGGAAAGGTCAGCCAGAAAGGTGGAGAAATTAGATACAGAGGC

GTTAGGAGGCGGCCGTGGGGGAAATTTGCAGCGGAGATACGTGACTCTGCAAGGCATGGA

GCAAGGCTATGGCTAGGGACTTTTAACACAGCAGAAGAGGCTGCGAGGGCTTATGACAGA

GCTGCATATTCAATGAGGGGTCCTTTAGCAGTCCTTAACTTCCCTGGTGAATATCCTAAA

ACTAAGGTTGTTTCCGATATAACGTCTTCTTACATTTCGTCTTCACCATTGTCGTCGTCA

TCTTCATCTGTTTCATCTTCTTCAATGTCACAGGATGCAGAAACAAGTGGGATTGGACGA

GGAAAAGAAGTCTTTGAGATTGAGTACTTGGATGATAAGTTACTGGAGGACCTTCTTGAT

TTTGAGGAAGGAACTAGCAAGAAATCAGACAATGGTTACTGTCAAGTTATCTGA

>EVM0014312

ATGGAAACTCACCAAGGGGAGAAGAACTCGAAGAAGCAAGGGAAAGGAAGAGAGAATGCG

TATAGGGGAATTCGCAGGCGACCATGGGGTAAGTTTGCTGCTGAGATACGTGATCCGACG

AGGAACGGAGCACGTCGTTGGCTTGGAACATTTGAAACGGAAGAGGAGGCGGCCCGGGCC

TATGACCGAGCTGCTTTTGCCTTCCGGGGTCACTTAGCCATCCTCAACTTTCCGAACGAA

TACCAGCATCAAGATCCAAACTTTACCAAGTCATTTGCTTCCTCATCTTCGTTCTCCACT

GAGAATCCTTCGAGTTATGGCCATGGAGTTTCCTCCACTGGTGGACAAGAAGTTATAGAG

TTCGAGTATTTGGATAACAAATTGTTGGAGGAGCTGCTGGGAACACATGATCACAGCAGG

CAGATTTAG

>EVM0040250

ATGGCACCTTCTTTGTTTTATCATAATCAATACTTGAATCCAGATCTCTCCCCTGAATCT

TCTTTCGGTTCTTTAGATTCATTTCCATGGGATGATCTGCTTTTTCAAAGCAATTCCTTT

CCTTTTAGCACTAGCGGCTCGGAGGAAATGGTGCTTTTTGATGTTTTAGCCGATGGTGCT

AAGGAGTCCTCGGAATCTAACTCCTCGAGTGGGATCAAGGAAGAGGAAGTGACTTCAAAT

GTCAAAGAAGAGGAGCCGAAGAAAGAGAAATCCTACAGAGGGGTTAGGAGGCGGCCATGG

GGTAAATATGCTGCCGAGATAAGGGATTCTACAAGAAATGGCGTCCGAGTCTGGCTAGGA

ACCTTTGATAGCGCGGAGGCGGCTGCTTTGGCTTATGATCAAGCAGCATTCTCCATGAGG

GGTTCGATGGCTATTCTCAATTTTTCTGCGGAGATGGTGAGAGAATCACTCGAGAAGATG

AAGTACAGATGCGAAGATGGGTGCTCGCCTGTGGTGGCACTCAAGAGGAGACACTCCGTG

AGAAGAAAATCAACGGGTAGGAAAAGTAAAGTGAATCAAGTGGCCAGTACTAGACAGCAA

AATGTGGTGGTTTTGGAGGATTTGGGAGCTGACTACCTTGAAGAGCTTCTGAATTCATGC

GAGAGCTCTAGTTCTTGGTGA

>EVM0023703

ATGAAGGATGGATCATTCTCTCTTCAACAACCCAATCAAGAATTCTTAACATATTCTCCC

TTCGCCTTTCTGGATCCATCGTGGGATGAGCTTCCTTTTGACCTTCATGATCTCTCTCCA

ATCAACTCAGATGACTCTGAAGAGAGTCCTCATCAAGAGACTCTTCCATCGGACGTTCCG

TTCACCGAAGCCAAAGCATCTACTCACAATATTACCTCGGAAGTTCCACCAAACATAGCC

AAAAATAATTCACCGCAACTTAGTTCCTCGGAAGAGATCAAGGAACAGGGAGCCACTCCG

GGAGCTAAAGAAGAAAATCCCACAAGGAAAAAGACCTATAGAGGGGTCCGAAATCGGCCG

TGGGGAACATATGCAGCAGAAATAAGAGATTCCACCAGGAACGGGGCGCGAGTTTGGCTG

GGCACATTTGATACTGGTGAAGCAGCTGCTTTGGCTTATGACCAAGCAGCATTGTCATTA

CAGGGCTCTAAGGCAGTCCTCAATTTTCCGGTGGAGCAAGTGAGAAAATCACTCGGAGAG

TTGAAGTGTGGTTTTACAGACCGGTGCTCACCAGCTGAGGCACTGAAGAAGACTCATTCC

AGAAGAAAGCCAAAGAGTAGTAAAAGGAAAAGGAAAGGGTTAGCAACCAAAGAAGTGTTG

GTGTTGGAGGATCTCGGAGCTGAATATTTGGAAAAACTCTTGAGCTCTTGTGAGAGAGGT

ATTCCTTAG

>EVM0000172

ATGGGAGGAATGTCAAAATCAATCGTGAGTGGTTTTGGAGAGAAAAAACAGTCCAAGAAG

CCTGCGCAGGGTAGTTCTAGAAAAGGGTGCATGAGAGGCAAAGGAGGACCAGAAAATGCT

CTCTGCACTTACAAGGGTGTACGCCAGAGAACTTGGGGCAAATGGGTGGCCGATATTCGG

GAGTCCAACCATGGTGCTCGTCTCTGGCTTGGCACCTTCGACACTTCCCATGAAGCTGCC

ATGGCTTACGATGCTTATGGCCCTGAGGCAAAGCTCAACTTACCCGAGTTACAAGCTAAT

AATAGCCTGTTTCCAGCCTCTCCTGCGAATTCTCAAGTGATCCAAATGACACACCAACCT

TCTCATCTCATTCATCATTCTAATCCCACGTCTACATGTTCATCAAATATCCCAAGCATG

GAGTCAAATGAGGCGAAACCAATATTTTACAACCATGATCCAATCATGTCTTTCTCTAAT

GAAAGCGTTTATTCGAATGGAATGGAGGCAGAAAATGACGCAAATTCACAATCTGGGTGA

>EVM0044966

ATGTGTGGCGGTGCTATAATCTCTAACTTTATACCTCCGACCACCACCGCTAGATCTTCT

CGGCGGTTTACGGAGGGATTCGAGTGGCTTGATACGAAGAAACCCGTCAATAACAAGAAG

TGCTCGAAGCCTGTTGTTAATCTTGAAGATGATTTCGAGGCTGCCTTTCAAGAGTATGAG

TCTGATGTCGATGAGAGCTATGATGTCAAGCCTTTTGCTTTCTCTGCTACTGCTTCTGCC

CCTGCTAAAAAACGTTCTGCAGCTGTTAAATCTTCTGAATTCAGTGGGCAAGCTGAAAAA

TCAGCAAAGAGGAATAGAAAGAACCAGTATAGAGGAATCCGGCAGCGCCCATGGGGAAAA

TGGGCTGCTGAGATTCGTGACCCCAGGAAAGGGGTACGTGTCTGGCTGGGAACGTTCAAT

ACTGCAGAGGAAGCTGCAAGAGCATATGATGTTGAGGCACGTAGAATTCGTGGCAAGAAA

GCTAAGGTGAACTTTCCTGATGAAGCTACACTTGCTTCATCAAAGCAGTCAATTAAGGAA

AACTCACGGAAATCACTTCCAAAGACAAATTCAAGCCGGACTTTCAGTTACTTGAGCAAC

CCAGAACTGAATTATAATAATATGGGCGTAGTGGAAGAGAAACCACTAGTTAATCAGTTT

GGATCAATGAATTCTTTTCCAGCCAGTGGAGATTCTGGGATGAAAACCTTAGCTCAATCT

GACAGTGCTCTTATGTGTTTCAATTCTGACCAGGGGAGCAACTCATTTGATTGTGACTTG

GAGTGGGGAGAACAAACCCCAATGACTCCTGAAAGCTTGACTGTTGAATCTCTCCTTGCC

AATCCTGAGGAGTTGAAATCATACTCTGGGAATGCAGTGCCTGCTGAAGAGAAGAGTGGA

AAATCTCTGTCTGAAGAGTTGCTGGCGTTTGACGATCAGTTGAAATACCTTCAGATGCCA

GATCTTGAGGGTAGTAGCTGGGAGGCTTCTCTTGATAACTTCCTTAATGGAGAGACAACT

CAGGATGGCACAAACACAATGGACTTATGGAGCTTCGATGACTTCCCCTCTATGGTTGGG

GGGGTTTATTGA

>EVM0026368

ATGAATCCATCTTCATCAAAAAGTAAAAAGAAGCAACATCAGCAAGTGCAGCAGGAACCA

GGCACTGGATTAAGGTTTCTAGGTGTCAGGAGAAGGCCATGGGGAAGATATGCAGCAGAG

ATAAGAGACCCTTCAACAAAAGAAAGGCATTGGCTAGGCACCTTCGACACTGCCGAGGAA

GCTGCCTTGGCCTATGACAGAGCTGCTCGCTCCATGCGCGGTTCTCGTGCTCGCACAAAC

TTTGTTTACTCAGACATGCCTGCTGGTTCTTCTGTCACATCCATTATATCCCCTGATGAA

CAACATTCTCTGCAGCAGCAACAGCAGCAGCGTAGTGGTAATGATAACAACTTGTCTTCA

ATATCCTTTAATGTCCCTTCATTCCATGATCATCAGCAGGACTCCACTCCCATTTTCAAC

CAGGATTTTAACTCCCAATGCCATTTGGTTGAGGGGTTTTCTTCAACGACATCTGGTGGA

GATTTTTGGAGCTGCTCCAGGAATAATAACTATAATCAACAGCCACAACATGTTACACAA

AACAATGTGCTTTCTCATGATTTTCCTTCAGATACTTTTCACGGCTCGGGTTATAATACG

GGTCATTGTGATTGGATAGATTCATCAACTTCGGGGTTGATGGATTTTGAGGACCAGACA

ACAATGAGTAATGGGCTTGAGTCTGTGGGGTGTAGCAGTGGTTCGTATTTCGGGTTTGAT

TCTGGAGAGTATGTCCACAGTCCACTTTTTAGCAGGATGCCTCCAGTCTCAGATACAGCA

CCAGATGGTTTTGATTTGGGCTCCTCTTCTTATTTCTTCTAG

>EVM0036581

ATGAAAGGGTCTTCATCTTGCTCCTCCTCCTCCTCCTCGTCTTATTGCCTTGCCTCTGAA

AGCATTCAGAAACCAAAAGTCAAACGCATTGGAAAAAACCAAAAGCGCAATCAAGAGAAA

TCCCAGAATGATGCTGCCGCCAATAGTACTAGCCCTGGCAAAAGAAGTTCCATCTACAGA

GGAGTCACCAGACATAGATGGACAGGAAGGTTTGAAGCTCATCTTTGGGACAAGAGTTCA

CGGAATAACATTCAAAACAAGAAGGGAAAGCAAGTTTATTTGGGTGCTCATGACAATGAA

GAGGCAGCTGCACACACCTATGATCTTGCTGCCCTGAAGTACTGGGGAGCAGAGACAACC

TTGAATTTTCCGGCAGAAACCTACACAAGAGAGATTGAAGAGATGCAAAGGATGAGCAAG

GAAGTGTACTTGGCATCACTTAGACGGCGAAGTAGTGGATTCTCCAGAGGAGTCTCCAAA

TACCGCGGGGTGGCCAGGCATCATCACAATGGGCGATGGGAAGCAAGAATTGGACGAGTT

TATGGGAATAAATATCTCTATCTTGGAATTTATAATACGCAAGAAGAGGCAGCGGCAGCA

TATGATATGGCAGCAATAGAATATAGAGGAGTAAATGCTGTGACCAATTTTGATGCTAGC

AATTATATAGAACGGCTGAGGAAGAAAGGCGGCATCCCTATAGACCAAATCCTCCAAGAA

CAACAACTTCGTAACAGCTCGATTGATTCCGTCATAGAAGCAGAAGCTGAACAACCATCA

ACCGAACAACAAGAGGAACAGGAACAAAATGTAGCTCCGTCGTTACAAGTTCAATGTACA

CAGCTCAATTCAAGCTTGGATGGCACATCTTCTATGGTTACTTTGGAGACTATCGAAGAG

AACGAGCTAGCATGGAGCTTCTGCATGGATCCAGGACTGAGCCTCGCAATGCTTGATCTT

CCCCTCAAAAATACTTGGGAGTTACCGGACCTGTTCAATCATACAGGGTTTGAAGACAAC

GTTGACTTAATATCGATCATGTTGCTATGGAAACTAGTAGCACAGGAAGGGTTGAGGAGG

TTGGTGTTGCAAGGAGTATAG

>EVM0022896

ATGGCACTTCTACACCGATTTCTGAGTCATACCTGTCTTTCTTCTATCTCCTCACAACCA

GGAATCTTCTCCTTCACAGTTTTGTTCTCTCTCTATGTTCATGGGGAGCTTTTGGAGGAA

TTGATTGGAAGGAAAAAGAAAATCCCAGAAAATATGATAGACCAAAGTATCCTATGTCCC

ATAAAGTACACTGAGCACAAAAAAGTTACAAAAAAATTCACTAAACAACCCGTAAAGCCT

AAAAAGGTTCACTCCGATGACCCGTGCCGCTCACCAGAACCCTCAAACCCACCACCAAGG

CTTGTTAGAGTGACGGTCACCGACCGTGATGCCACAGACTCCTCTAGTGACGAAGAGGGA

GAGTTGTTTGGCCGGCAAAGAGTGAAAAGATATGTGAACGAGATCAACATCCAACCTGCA

GCCTTCTGCAAAGAAACTAATGTCATTGCTGCTACTATTGCCAAAGGCCGGAAAAGAAGT

GGCGGTGATATCCCTCTAAAGCCGGCGAAAAAAATTACTACCCAGTCAACCAACAATGGC

AGAAAGTTCCGCGGCGTTCGGCAGAGGCCTTGGGGTAAATGGGCAGCAGAGATTAGAGAT

CCTGCTAGACGCGTGCGGTTATGGTTAGGGACTTACGACACCGCTGAGGAGGCTGCGAGA

GTTTATGATAATGCTGCTATCAAGCTCCGAGGGCCAGATGCCTTAACAAACTTCATCACG

CCGCCTAGCAGGGAGGAGGATCAAGAAGAGAAAAGTAGTGCAGAGGAGGAATCGGAAGAA

AAGAAACCAGAGATTAACGTGGACACAGTTTCTGGCTCCGGCTATGAATCTGCTGACGAG

TCTCACAGTCTGTCATCTCCAAAATCTGTGCTCACTTTCAGATTACCTTCGGCTGAATCA

CACAAACCACCGGGCCAACCTTCCCAGGAAGCTAAAATGGCGGAGCCAAAGCATCAAGAA

AACCAAGAAGCCTTCGGCGAGAGCAATCTGTGTGATTATTTACCACTCGACTCAACCTTC

CTTGATCAGTTTTTCAGTTTTGGAACAGCCGGGCCAACCCTCTTAGACAATCAATTTATA

CCGGCGGCGGCGGCGACATCAATTTTTCAGGAGAGTATTATGTGTGATCAAGATTTTGGT

GACACATTTCTTGAACCACTTCAAGATTTGACTTCGTTATCATCGTTTTTCCAAGACGAC

GATGATTGTTTCGATTTCTTAAACTCATAA

>EVM0013798

ATGGAAGAAGCACTAAGAAGGCTCAACGGAATGCCGAACCATGTACTGGAGTCCAACCCG

CCCGAGACCACAGTCGTCGACCTCCAGAAGAAGTCAGCACCCACCACCACAAGCAAAAGG

TCACTGAAAGAAAGTGGTGGAAATAGCGGAAACATGAGGTACCGTGGAGTCCGCAGGAGG

CCATGGGGTCGTTATGCGGCCGAGATAAGGGATCCTCAGTCTAAGGAACGGCGGTGGCTC

GGCACATTTGACACCGCTGAGGAGGCTGCCTGTGCCTATGATTATGCTGCTCGAGTTATG

CGTGGTATCAAGGCACGTACTAATTTTGTGTACCCTGAGACATATTCTGCAACAACTGAC

TCTCAGAATTTGCTCCCTCCATTTACTTTCTCAAAATCATCCCACCATCAGCCCTCTCGC

CAGTGCAATACAGCACCATCTGCAAATTGGTCTTCTCCTGGACACTCTCTCCACTTTCCC

TGCATTAATGGGTCCTCCTATTCTGCTCTTAGCACTTACTCAGGTGGCTCTTTGGCGAAT

CCTTCTAGTAATCCCAGCAGTACTGGTATATATAATTTATCTGGAAATTTGGCAAACTCA

ACTGTCACTAGTCTACCCCACATGGAGTTTAATCAGAATTATACTCCTGTGGCCCTGAAT

AGGCCCACCTCAAAAGCTGAAGATTTGGGTTTCTTTCCGCAAGAACCATCCGGTTCCGGT

TTGTTGCAAGAGATTATTCAAGGGTTTTTGCCCAAGCACTCCACTGAAACGACTGACTTT

TCAAAAAGCTCTGGAGAGTCGATGGTTACACCATCAGCTGAAATAATATTTGGTGGCCAG

TCGCTAGATGGATCAAGGAGCACGAAGCACTTAGTTGAAAACGACCACCATGGTGCTGCT

TATCTTGATCACGAAGGAATAAAGCCATCAGCACAGGCCGGGGCTATTACCATGTCACTG

AATCTTCCGGTGGGTCAGGACTCATTTCTGGAAGACATTTTTCAGTACCCAGATCTTATG

AGTGCCTTCGCAGCTAGGTTCAATAATGTTTGA

>EVM0003379

ATGCTGTTTTTCTGCACTAATGAAAGTTTTGAGCGAGTTTCTAGCTGTTTTAATTCTTGT

GCTAAAATGAAACGTTTCGTGTTTCTTTTGGAGTGCAGGAAGACTAGAATATGGTTAGGT

ACATTCGAAACGGCCGAGGACGCGGCGCGGGCATACGACGAGGCAGCAAGGTTAATGTGC

GGGCCAAAAGCACGCACAAATTTCCCTTACAATCCCAACGAACCGCAATCATCATCATCA

AAGCTTCTCTCAGCTACCTTGGTAGCCAAGCTACATAAATGTCAAATGACTTCTCTACAA

GCAACCAAGAAAAATGTGACAAAACAGTCACATGATGTACGATGTACCACTTTTGTCACT

AGCCATGGCATTGCCGAAAACACTGTCGAAAACGGCTCGAAATGGCAGGCGGGGGACTGG

GTTGGTGGGGAAAGTCAAGAGGGGAACGGTGATCATCAAGAACATTTTAAGTCACTTGAA

GATCACCATATTGAGCAAATGATAGAGGAGTTGCTAGATAGAGGGTCCATGGAGTTCTGC

TATGTTGGTTCATCATGA

>EVM0045763

ATGGATGGCTCCTTCTTTCAATATTCAAATTCTGATTTTTCATCCGAATCTTGTTTTGAA

TCGCCAGACTTTTTCTATGGCCGATCTTTTAGCCAAAGTTCTCTCCCCTTCAATGAAAAT

GACTCCGACGAAATGCTTCTTTTCGGGCTAATCTCGGAGGCGACACAAGGAACGTCAAAA

ACAACTTCCTATAATGGAGTTATCAAGGAAGAAGAGGTTAGTTCCATATCCAAAGAAGAT

CCGAACAAGGAAAAGTCCTACAGGGGTGTCAGGAGGCGGCCGTGGGGCAAATTCGCGGCA

GAGATAAGGGATTCCACAAGGCACGGCGTAAGGGTGTGGTTAGGCACATTTGACAGTGCA

GAGGCAGCTGCTTTGGCTTACGATCAAGCTGCTTTTTCAATGAGAGGAACTGGGGCAACA

CTCAATTTCCCAGTTGAAAAGGTGAGAGAGTCACTGAAGGATATGAAGTGTACGGATCAC

GAGGATGGGTGCTCGCCTGTGGTGGCTCTAAAGAGGAAGCACTCCATGAGAAGGAAACTG

GGAAGCAGAAGCAAGAAAGAGAGGAATGTGGTGGTTCTGGAAGATTTAGGTGCTGATTAT

CTGGAACAACTATTGAATTCATCTGAAGATGCTGCTAGTCCTTGCTGA

>EVM0036069

ATGGAAGAGCCCGGCAAAGGGAAGGAAAATGATGAGAAAGGGAGAGGAGAGGTTCGATAC

CGGGGGGTTCGGAGGAGACCTTGGGGGAAATTTGCAGCGGAGATACGAGACCCGTCAAGG

CAAGGAGGAAGGTTATGGCTCGGGACATTTGATACGGCAGAGGAGGCAGCTCGGGCTTAT

GATAAAGCTGCATTTAACATGAGACGTCAACTCGCAAATCTCAACTTTCCCGGTGATTTT

ATATCTCAAGTGATGGGGTATTCTCCTCGTCCTCAATCATTCCCGCCATCTAGCAGTAAT

GTTGGTGCAACTGAGAGTTTTGAGAGGGGAAGTTCATCAGGTACCGGACAAGGAAAGCAA

GTTTTCGAGTTTGAGTACTTGGATGACAAGATTTTGGAGGAGCTTCTCGAAACAGAAGAG

GAGAAGAAGAAGCGGCGGCAAGATTGA

>EVM0012743

ATGGACGAGGACGGGACAAAGAGCAGGGATGAAGGAGGGGAGGTCAAGTATAGAGGTGTC

CGGAAGAGGCCATGGGGTAAATTCGCTTCGGAGATACGTGATTCAACCAGGCACGGAGCG

AGGGTGTGGCTGGGGACTTTTAACACGGCGGAGGAGGCAGCAAGAGCATATGACAGAGCA

GCTTATGCAATGAGAGGGCACTTGGCCATTCTTAATTTTCCGAACGAGTATCCCAATATG

GCAAGTGCTGGTTCCAGCAGCTCTACTCGTTATTCAGGTTCTTCTTCTTCATCATCAATG

CAACCAGAAGTTTTTGAGTTCGAATGCTTGGATGATAAGTTGCTCGAGGAGATGCTTGAG

CAAGAAGAGATGAGGAGTAGGAAAAGGAAATTGCTAGCCAGTTACCCTCCATGGTTTTCC

TTATTCAAATCTTCCTTGGATGGCCATGGTCAGCGACAAGCAGAGCACGGATTTAAACCG

TGGGCTAAACTTAAGGCATAA

>EVM0001106

ATGCGTGAGCTAAAAATGGAGAAAACTGATAAGAAAAGAAGAGGAAAAGAGGTTGGTAGT

GGTGGAGAGCGGCTGCATAGCCAAACTCTGCCAACGATGCTTACCGAGTCGAGTAGGGAA

AGAGAGATGTCAATTATGGTGACTGCTTTGACACATGTAGTGGCTGGAAATGTAACTGCA

GATGATTCGGATTCCTTGACAGCAGTTTCAAACAATCAAGGTGATTCTGTTCATGGTGGT

TTGTCGGTGAAGAGAGAAAGGGAAGAAGATGGCGGTGGCAGTGAAGTGGATAAAGGGTTC

CGTAGAGCATTTGGTGATGACTTCTTACATGGGGGCTCTCCTTCTGCTGGAAGAGGAATT

TCCATCGTCACTACAAGCATCATAGCACCTGCGTCTAAACAGATGACATTTACTCCAGTA

TATGAATACAATGAAACCCACAGAGACGAGCCACAGAGAAAATATAGGGGAGTGAGGCAA

AGGCCATGGGGCAAGTGGGCTGCTGAAATAAGAGATCCAGTCAAGGCAGCTAGAGTTTGG

TTAGGCACTTTTGATACAGCTGAGGCTGCAGCAAGAGCCTACGATGAAGCTGCTCTGAGA

TTTAGAGGCAACAAAGCCAAGCTCAACTTCCCAGAAAACGTCAAGCTTCAACCATCTCCT

CCGCACAGCCAGACAGCAAACCAGTTTACTGTCTCTGATTCTCCAAGTACAGGCTTTCTG

TCCCTTCCTCCATCTTCTAACCCCATGCTTCATTCCCAGGCTCTTAACCATACGCAAAAC

CGTGAGATTTCAAGAGAGCAAGTTAACCAACCGCAATTGATCTTGGGTGTTGGTGGTTAT

CAAAGGCAACCCGTGAGTCTATACGACCAGATGTTTATGTCATCTTCATTTGTTTCTTCT

AATTCCTCATCATCCTCCACACAACCTTCCGATCCTATGTTTTCCCCAACCCAGGAACCT

GGTGAGTTTAGGCCTTCCGCTAGTGGCGGAAGAGATTTTCAGTTGCCGGCCTGGTCAGGT

TATAACCATTATACATCATCTTCCAGATAA

>EVM0002653

ATGCCACCAAAAAGACAATCTTTGCTTTACACTACATCCGGCAAAAAACCCAACGATACT

AAACGAATCTTGTCGGAGTTAAAAATGGAGTCCACTATGAGAAAAATCCGTGTCATCTGT

TATGATCCGTATGCTACCGAATCCGATTCGAGTGATGATGAATCTGACAAGAAATCGTTT

AGAAAACGTTTTGTTCGAGAAATCAATCTTCCTTTGGTTGTAACCCCCCAGCCAAAAACC

TTTGAGCCTGAGAGTTCATGTCAAGAAAGTAATAACAGTGCTAAATCCCAGGGTCAGAAG

AGGAGGGTTTTGACCGAACCCGGTAAGAAGAAGAGGGTTTTGCCCGAAACCCCAACATCG

ACCTTCGAAGGTAAGAAACTAGCACCAAAGAAACCTGTTGGTGTTAGGCAGAGGAAATGG

GGTAAATGGGCAGCTGAGATTCGGAATCCAGTGACCAAAGCTAGGACGTGGCTGGGTACT

TTCGATACTCTTGAAGAAGCTGCCCAAGCCTATGAGGCAAAGAAGCAGGAATACGAGGCT

TTGGCTATGTTAGCCTCTCAAAAAAGTCAAAACATTTCATCGTCTCTGGCCATCTCACAA

TCTCATTCTCACATCAGCAGCAAAAAACAAGTCAGTTCTGCATCTTCCGAGGATACAGAT

GATAGCGTTGTTTCGCATACTTCCCCTGCTTCTGTTCTTGAGCGGGACACATCTGTGCTG

ACTGTTACTGGTGAATGTGCTCTTGTGATCAAAGATGAAGACGTTGGTTCTTCTGTTGCG

GATCTTGAGATACCTGATTTAGATTTCATGAACGACCCACTGGCATCTTGCTCAATGGAT

CAAGATCTTAACCTGAGTCTCGACCTTGGTAATCTTATTGATGAGTTTGGTCGATTTTAT

GATGATTACTGCGGTATTGATGATCTTGCTATTTGCGGGCTAAATGGTGAAGAACCAGGC

GAGCTTCCTGATTACGATTTTGAGTTTGGCAACGAGGAATTCAACTATTTGGATGATCAT

CAACAGAAGCCCCCCAACATTGCATGCCTATAA

>EVM0046641

ATGAACGAGATTAGCTCACTGTGCGAGTTCAGTGCTCTGTCTCATCATCATCAACATAGA

ACTCCGAGCTTTAATTCTCTTATTTCTTGCTTAAACGAGACATGGGGTGACTTGCCATTA

AAAGTTGATGATTCTGAAGATATGGTCATTTACAATTTCCTCAGAGATGCTGTTAGTATT

GGATGGTCTCCATTAGACTTGAGCACTTCAACCGCTACTTCTTCTTCTTCTTCTTCTGCT

TCTACTACTACTACGACGACGACGACGATAACAGCTAATGTTGTAAAAGCAGAGCCGAAA

GAAGAGCTGGATCTGGAAGCCGATCCCATTAAATTGGTTCAAGAATCTAGCTTAAATATG

GAAGCTGTTGTGACTAAGAAGGTGGTGACCAAGGGAAGACATTATAGAGGAGTGAGGCAA

AGGCCGTGGGGGAAGTTTGCGGCTGAGACAAGGGACCCAGCTAAGAATGGCGCGAGAGTG

TGGCTTGGGACATACGAGACGGCAAAGGAAGCGGCTCTGGCTTATGATAGAGCGGCTTAT

AGAATGCGTGGCTCAAAGGCTTTACTTAATTTTCCACATAAGATAGGGTCTAATGAGCCG

GATCCGGTAAGGATCACTGCCAAGCGTCGACAGCCAGATACAGGTCTGACGGCTGTGGAT

ACTGGCTCGGCTAAGATGAGAAAAATGTTGGTGGTTGAGGGAGCTGAGTTGGAAAGGGGG

GTGGGATATAATGTGTTTCAGGTTGGTCCTCACAAGGGGTTAATACCAGTTGGCGAGCAA

TTATTGGCTAGTTAG

>EVM0006972

ATGGCATCTTTTGATGAAGCTTCAACTTTGGAACTCATTAGGCAACACCTCCTGACTGAC

TTTGCTTCCATGGACAGTTTCATCTCCGATCTTGATGATCTTTGCAGCACTTCCACCACC

AGCTCTACTGATGACTACTGCATCAAGACTGAATCGACAGAATTTGGTCCATTCGATCAA

CCACAATTCAAGCTGCTAACTCAATCTAGCATTGAATCCAAGCCTCAATCCTCTCCAAAG

TCTTCAACTTCTCTAAGCCAGAGAAAACCATCCATGATCAAGGACATAGCCATACCTCCA

CCAGCAACCTTGAACGTGACCCCACAAGTAGCGCAGCCAGCGGTGAACAAGGCAGACATG

TCCAGTGAACAAGAAAGGCATTACAGGGGTGTGAGGCGTAGGCCTTGGGGAAAATATGCA

GCGGAGATCCGTGACCCTAACAGAAAAGGAGCTCGTGTTTGGCTTGGAACATTTGATACT

GCCATTGAAGCTGCAAAAGCTTATGATTGTGCTGCTTTCAGGTTACGAGGAAGTAAAGCG

ATCTTGAATTTCCCTCTTGAAGCTGGTAAGTTGAATTCTCAACAGCCTGAACAGTTATTG

GAAACCTCTTGCAAGAAAAGAAAGGTTGAAGGAATAGCGAGTTGCGTGGAAAGTACCAGT

CCTATCACTAATAAGGTGGCGAAGAGGGAAACTTCTTCACCGGAAAGCGAGGTCAAAGCT

GTGGCTTCCGAACCATTAACGCCGTCAAGTTGGAAGGGGTTTTGGGATGGTGAAGTCACG

GGGATATTTAGTGTGCCACCATTATCGCCGTTATCTCCACATCCTTCATTTGGATACTCA

AGGCTCATGGTCGTATGA

>EVM0019924

ATGGCAACCCCAGAAGAATCCTCCACATTAGAACTCATAAGACAGCATCTTCTTGGAGAC

TTCACCTCCACAGATGAGTTCATCAATAATCTTGAGTCCAGTGTAGCCAGTATATATGTC

AAGCTGGAGAACTCCTTATCTTGGTCTGAATCCAACTCCCCAATATCAGACCAAAGTTAC

TGTACCCAAGAAACCTGTAGTTTTGAAATAAAATCCGAAATCATAGATTTGACACCTCCT

GAAACCATGTTTTCAGATTCAAGTAATCAGTCCCCAGCACCTGAACTCGTAAAGATGACA

GATAAAGATGAGACAAGAAGGCATTACAGAGGGGTCCGAAGAAGGCCATGGGGAAAGTTT

GCGGCAGAGATCCGTGACCCAACCAGGAAGGGTAGTCGGGTTTGGTTAGGTACTTTTGAC

AGTGATATCGATGCTGCAAAAGCTTATGATTGTGCTGCATTCAAAATGAGGGGCAGGAAG

GCTATCCTGAATTTTCCTTTGGAGGCTGGACTATCCAACCCGCCTCCGGCCACAGGCAGG

AAGAAGAGAAGGGTGAAGAGAGAAGAGGTGCTGCCAGAATCTATTGATGTCTCGCCGGAA

AACTGGAATGCAGGGTGGAGTGGAGAGGAAGCGGAGGGCGTTTCTGATGAGGAGCAACTG

TCACCGTTATCTCGAAAACCAGTTCTGCTTACGTGTCAGTAG

>EVM0032101

ATGGATTTTACTCGCTCGACAATAACCAACTCCACACCCTCTCCTTCAAAGACCAAAAGA

AAGCAGCAGCAGCAGCAGCAACCACAGACCCAACAAGAAGTAAGGTTCTTAGGGGTAAGG

AGAAGGCCATGGGGTAGATATGCTGCGGAAATAAGAGACCCTTCAACGAAAGAGAGGCAC

TGGCTTGGTACTTTTGACACAGCTGAGGAAGCTGCCGTGGCTTATGACCGTGCAGCCAGG

TCCATGCGGGGCTCTAAAGCTCGCACCAACTTTGTTTACTCAGACATGCCTCCTGCATCC

TCTGTCACTTCCATTATCTCACCAGAAGAGTCACAACATGACAATTCTACCGTCTTTTCT

CCTATTCCTCGACACAATACTCACCAAAATGACACCAACTGCCAACAGCTCTGCTTCTCT

CGGGATCAGTATCCCTTCAATGCTCAAGCTTTCGGTAACAGTAGTAGCAACTGGTTAGCG

GGTGGAGCAGGTTGGGTTCAAGGATTTGAAGCCGGGGCTGGAGACGGGCCTTGTGGGTCA

TTCGAGCCCAATCATGCTGACAGTTTGGATGTTCCTAGCGGGCCTAACTATATCTCTAAC

ACGGACAGCACTGAGCTCCCACCTTTGCCTCCTGATGTCAACTCTAGCTGTTATGGGTGT

GATATGGGTCATCAATTTTGGAATGACACGGGGTTTTTTGGGTTTCACGAGGAACAGTTA

AATGATGCGAATGGGCTTGAAGTTAGTGGATCATTCATGGGCTTTGAGCCCAATGACTTT

GGGCAGCATGGCTCGTTATTCGGGATCGTGCAGTCGGTCACAGACGGGCTTGATTTGGGA

TCACCATCTACTTTTTATTTTTAA

>EVM0032029

ATGTCTTGTATGGTTTCAGCTCTCACTCAAACCATGGTGCTACCGACAATAACGTCCCGA

AAGGCATTACAGAGGAGTGAGACAAAGACCTTGGGTAAATGGGCAGCCGAGATACGTGAC

CCTAAAAAGGCAGCTCGAGTCTGGCTCGGTACTTTTGACACTGCTGAGGATGCAGCTGTT

GCATATGACAAAGCAGCACTCAAGTTCAAAGGCACCAAGGCTAAGCTTAATTTTCCTGAA

AGAGTTCAAGGGAGAACCGAGTTCGGCTACTTCATGGGTTCTGGGGATTCAAGCAATGTC

TCGACTGAACAAAATCCGAAGCCAGTTCCTCCTCCTCCCCTCCCCCTTCATCATTTGCAC

CGGACACTTACCCGGACTTGCTTCAATATGCACAGATCCTTTCTAGCAATGATGCCAATT

TCCCGTACTACACTAGTATTATCTTCGATTTCAGTGCTAAGAGGTCAACATGGCATCCCT

TAG

>EVM0051729

ATGGATTCAGCTAAGACCAACACTGGAAAATCCAAGAAGGGTGTTGTTGATGAGACTGAG

AGGCAGAGAACGGACCAAGATTGGCAGCTTGATAGAGGCAGAAAAGAAGCTGATATTAGC

TTTGAAAGGCGGCAATGGAAGCCTGTTTTTGATGAAGCTTCCTTGTCGGATAGGCCTTCC

AAGAAGATCCGTAGCCCTGAACGTCAAGAACAAAAGCAATCCTCTGCAAATTTAGCTCAT

CAATTACCACCTTTCTCTGTTTCTCCTAGCTCTGCCTCTACTCAGTCTTTATATCCGCCA

TCTTCATCTTCAAGTTCAGGACATCAGTTTCCTTTTGCCTTAGAAGGGTCTAATGAACCT

ACTCAGTTCCACCAACAATTTGGAACAAACCCTTCATCAACTATTTTTCGTCCACCATCT

CAGCAGCAAATGATTTCTTTTGCTCAAAACCAGCAATATGGCATTGCGTATCCTCCGTTT

CTTACAGGAGAATCGGCATTGGCTAACCACCACCAGCAGCAGCAGCAGTTTCAGTACTGG

AATGACGCATTGAACTTAAGTCCGAGAGATAGGATGATGATGATGATGAACAGGTTGGGG

CCAGATGGGAGGCCATTGTTTAGGCCACCGATTCAGCCTATAAATACAACAAAGCTTTAT

AGGGGAGTGAGGCAAAGGCATTGGGGAAAATGGGTTGCCGAGATTCGTCTCCCTCGAAAT

AGGACTCGCCTTTGGCTGGGCACATTTGACACAGCTGAAGATGCTGCCTTAGCTTATGAT

CGCGAGGCCTTTAAGTTGAGAGGAGAGAATGCTAGGCTGAATTTCCCTGACCTTTTCCTC

AACAAAGATAAAGCAACTTCCACCGCTCCAAGTTCATCAGTTTCTTCTCTTCCGACCCCC

AATCAAAGTTCAATGCCAAAACAAGCCCGGGAAAGCCTTAACTTGCAGGTGGGAACCATG

CCGCCGCCGCCGCCACCACTAGAACAGCCCCAAGGAGACCATACTGATGATGATTCCGGG

ATGGGTTCAAGTGGGGCTACTGTGAGTGATGAGGTTCATGCAGTGGCAGAGGGGACCAGT

GCAGGGGAAGGCATTTCGGGGTCTCAGGAGATGGCGTGGGGAGACATGGCAGAAGCTTGG

TATAATGCTATTCAAGCAGGTTGGGGTCCTGGGAGTCCTGTGTGGGATGATTTGGATAAC

ACTAACAATATTTTATTACAATCACACCTTCCTTTTGTTACTCCAAATCAACAGCAGTTT

GATGATTCTTCTGATCTCCAGAGACAACAAGACAGCATGGGCTCAGCTTCTTCTTCTTCC

CCCCCCACGAAGCCATTCTTTTGGAAGGATCAAGATTGA

>EVM0044712

ATGAATCGCTTTTCTACCAAGTACACTGAGCATAAAACTGTGACCAACAAGCTGGTGAAA

TGGGAGGACTCGAAAGCCACAAGAATAGTACGGATTTCAGTTACCGATGACAATGCTACG

GACTCGTCTGGTGATGAAAATGAGGAGTCGAAAAGGCATCATCCAAGAGTTAGAAAACAT

ATAAACGAGATAAGAATAACAAATTCCGGGAGCGACAGGGCAGCAGAGGATGCAAAAAAA

TCTTCAAAAGTGGTAAGCCGGCAACGGGTTGTGAAAAATATTTCGAGAGACCAGAGCTAT

TATCCCGGGGTGAAAAGGTATCGCGGTGTGAGACAAAGGCCATGGGGGAGATGGGCAGCA

GAGATCAGAGACCCTTATAGGAGAGCCCGGCTATGGCTAGGGACTTATGATACAGCAGAG

GAGGCAGCCATGGCTTATGATCAGGCAGCTATTCGTATTAAAGGTCCTGATGCACAAACC

AATTTCACAAAACCTCCGGTGAGCAAACAGCATACCCGAGATGTTGATATTAACGTTAAC

ATTTCCGAGTATGATTCTGGTAAAGAATCTCATAATAGTCTATGCTCTCCAACTTCTGTG

CTAAGATTTCAATCTACCGAGGAACCGGGGGCAGAGTCTCAGGTGCAGAGTGATTGTTAC

TGGAGGATACCAACGCAAGAAGAGGTGGTTAGAGAAGAATCAATAAAGGTTGGTGATGAT

GATGATGAGTGTTTGGTTACGGATCCATCGTGTTTGGGAGAGTTTTGGGATTTTGAAAAT

CCTGCTCCGATTTTTTTTGAGTGTAGCGTTCCGGGTACTGTTTCGAGAGAAGATTATGCC

GATATTTCGGTACATTTTGATGGTGATTTCGGTTCCTGTTTATGGGATGTCGACAAGTAT

TTTGAGGCTTAA

>EVM0052641

ATGGAGCAACTACCATACACTGAAGATGACCCGACTACAAATCCAGAGGCAGCCACCGCA

CTAGCCCGCAAAATTTCAGACAAAACCCGGGGAAGCGGCACCCGACATCCCGTCTATCGC

GGTGTCCGAAAACGGAGGTGGGGAAAATGGGTGTCTGAAATTAGAGAGCCTCGCAAGAAA

TCACGTATTTGGCTAGGCTCATTTCCTGTGCCAGAAATGGCAGCCAAGGCATACGATGTG

GCAGCGTATTGTCTTAGAGGTCGCACGGCGCTACTTAATTTTCCGGACGAAGTCGATGAA

TTGCCTAGACCATCCACGTGTACGGCCAGGGATATTCAAGCAGCAGCAGCTAAGGCTGCA

CATTTGGTACTGATTCCAATGGAAAAGCGCAACGAAAAAAACAACGATGGTGGTAGCGAC

GACGGTGAAGTTGCCGGGGATGATTTCTGGGGCGAGATTGAGTTGCCGGAATTGTTGTTG

AGTAATAGCGGGTACAGTTGGGATTGTTGTGGATGGAATACTACTTTTGCAAGTGATAAT

TCAACGTGGCAGCCGGATGGAGAGGGTTTAAAACTTAAAATAAAAATTCGTGGTACAACC

CGTTCACCACCAGAACGATGA

>EVM0013502

ATGGATGTGATGGTTTCAGCTCTATCTCAGGTAATCGGAAGCGGCCACAGCAACCCAGCT

CAGGTGCATGAAAATCCAGTGACCTCGACGCAATCCAGCACAGAAAACGATCATCAGACC

CAGCCAGTTGTTCAAGACCAAGGGAATGCAAGGAGACGTCATTACAGAGGAGTCCGGCAA

AGGCCGTGGGGCAAGTGGGCCGCCGAGATCCGAGATCCCAAGAAGGCAGCTCGAGTGTGG

TTAGGCACTTTCGAAACGGCCGAGGCTGCCGCCTTTGCTTACGATGAAGCAGCTCTTAGG

TTCAAAGGAAGCAAGGCCAAGCTCAACTTTCCTGAACGGGTTCAAGCCGGCGCGACCGAA

TTAGGATTCATTAGCTGCGGCCAAGGCTCGCACACGGTGGCTGAACCTGTCCCTGATCAC

ATTATGGCTCCTCTAGCTCGTTCCCGACGACCACATGAAGCGTATAATCCAAATAATTTT

CAATATCCACAATTTCTTGGAACTACAGCAGGCTATAGCTTCAGCCATGTCATGCCAGAA

GCTCCATTTGGTGGAGAAACGTTTCTTTCACCGACTTCCAGCTCCACTGCATCTTCTAAC

TCGTGGCCGATATCTTCTCAGCAGCAGCAGCAACAACAACAAGAGGAACTATTGCGATTA

TCAAATCAGTTTGGAAGCTCTTACAGCTCTCGTTATGATCCTTCTACATTTAAAGACGAG

GGGCGCTGA

>EVM0032021

ATGAGTGATCATCAGCAGCAAAATCTGGAGACAGAAAGTAGCTCAACAAACGCATTCTCC

CCGCCCTCACCATCTTCACCATCTTCTAACTCTACCCCGTCAAAGAAACATCAACAGGAA

CAGGATTTTTCTCCCTGTAAGAAAATAACAAGAATAAGAGACTCTAGCAAGCATCCAGTC

TATCGAGGAGTTCGGATGAGAAACTGGGGCAAATGGGTGTCTGAAATTCGAGAGCCTAGA

AAAAAGTCGCGCATTTGGCTGGGTACTTTTCCTACCCCAGAAAAGGCGGCGCGTGCGCAC

GATGCCGCTGCTCTAAGCATCAAAGGAAACTCAGCTATTCTTAATTTCCCTGAACTCGCT

AACTCTTTACCCCGTCCAGCTTCACTTGCTCCTCGTGACGTTCAAGCAGACGCTGCTAAA

GCTGCCCAAATGGACATGTTTGACCCAAAATCTGAAGCGACCACCACCACAACAGCGTCT

TCATCGTCTTCTTGTACATCTTCTTTGACATCCCTTGTTTCTATGATGGACTTGTCTCAA

GATGAAGAGTTGAGCGAGATTGTCGAGTTGCCAAGTCTCGAGACGAGTTATGACGAGCTA

AACAATGATTTTGTTTACTCTGACTCTGTTTATGGGTGGATGTATCCTCCACCGTGGATG

CAAAGCGTGGAGAGTCTCTGTTTCAGCGGTGGCGGCGGCGGGTATGCTTGTGAAGATCTC

ACACTGCCGAATGAGAGTACTGTTTTATGGAATTATTAG

>EVM0005644

ATGGAAAAACAAATCAACCTTGAGATTGATGCTCAAGACAATCCCCCCGCCTCAGCTTCC

ACTACCGCCTCCGCCACCAAGTGGACCACCACCTCCTCCTCTCCCACCTCCTCTTCCTCC

GCGCCCATTTCATACATTAACCGAAGCTCAAAGAAAGAAGAGAACTCTATTGCTATAAAA

GTATCAAGAAAAGCTCAAGAATTACAAAGTGGCAGCGAGGGTAGTAAAAGGCGAAAGACC

AACGAAAATGAGAATAACGGGAAGCATCCAACATATAGAGGAGTAAGGATGAGAAGTTGG

GGCAAATGGGTGTCTGAAATCAGAGAGCCAAGGAAGAAGTCAAGAATATGGCTTGGAACA

TACCCTACAGCTGAAATGGCAGCTCGAGCACATGATGTAGCCGCGTTAGCCATCAAAGGA

AGCTCAGCTTATCTCAATTTTCCTGAATCTGCTCACGAACTTCCTCCTCCACTCAGCAAG

TCTCCCAAGGACATTCAAGCTGCAGCCGCCAAGGCAGCGGCTGCTTCGTTCACTGAAACA

AGATACTGTGAAGAAGGTGGGGGCGAAGCCGAGCTTAACGTTTCGAGTTTATCAGATAGT

TCGGCCATGGACAATACACAAGAATCAAGCGGTTCTCCTTCTACTGATTTCGATGACACA

TTGTTTGACCTTCCTGATCTCTTCATTGATGGCGTTCATCACAGTGATGGGTCTTGCTAT

TATTCTTCATCATGGCAATTATGTGCAGCCGATAATGGCTTCCGCCTTGAAGAGCCATTT

CTATGGGAGTACTAG

>EVM0045686

ATGGCGCCCAAGAAGGATTGTGATAATGGCAATTGTGCTTTGAAGAAAGCAAGTGGTGAT

CATGATAAGAAGGAAATCCACTACAGGGGAGTGAGGAAGAGGCCATGGGGGAGGTATGCT

GCTGAGATAAGGGATCCCGGGAAGAAAACCCGGGTTTGGTTGGGTACGTTTGATACGGCT

GTGGAGGCTGCAAGGGCCTATGATAAGGCGGCGCGTGAGTATCGTGGTGCTAAGGCGAAG

ACCAACTTTGCTTTGGCGGAGAAGGTGGTTAATTATAACGATGAGAAGCAGAGCTCTAGC

CAGAGCAGCTCGGTTGAGTCGTCTAGCTCCCCGGTGGTTTCTGCGGTGGCGCGTGATGTA

ACTCACCAAGTTGGAGGGGTCATGGGGATGGGAAGGTTTCCTTTTGTGTTGCAGCATCAG

CCGACTGTTAACGCTGTCGGTCCTGTCTGGTTTCTTGATAGTGTTAAGCCTGAGCTTGTG

GCTCAGCGATTCCCTGTCCGGTTTGATCCGGTGGGTCTAGAGGGCGGGGCCCAGAGTGAC

TCGGATTCATCATCTGTGATTGATTATAAGCCAAGGAGATCAATTCGTGATCTTGATCTT

AACCTGCCTCCACCAGCTGATGCATGA

>EVM0004890

ATGGAAGATTCGTCGAGACAGAGTTCTTTGCTAGGGTGTAGCAGCAAGAACAAGAAGTGG

CGGGGAAGGCGTAATGGTTGTGAATCAATAGAAGATACAATCGCTAGGTGGAAGAAACAC

AACAAACTTCAAATAAACAAGGTTCCAGGCAAGGGGTCGAAGAAAGGTTGCATGAAAGGA

AAAGGTGGCCCAGAGAATAATAGCTGTAGATATAGAGGTGTTAGGCAGAGAACCTGGGGC

AAGTGGGTTGCTGAAATTAGAGAGCCTGTCAAAAAAGGCAGCATAACGAAGAAACGAGGA

AATAGACTCTGGCTCGGTACATTCTCTACTGCTATTGAAGCCGCTCGTGCTTATGATTAT

GCTGCGAGAGCCATGTATGGTCCTAATGCCATACTCAATTTCCCTTACTTCTCTCACGAA

TCTGGGGATCAGTTGGATAGCTTATCGTCTTCGGCTACAGCAACTGAAACACAGTCAACT

GGGTCTAAAACAACACTGGATAAATATGAGGGGAAATTGAAGATGGGTTATTGTGGGTCA

AGAGTGGTGAATAATAAACAATCAGGTTCTTCTGGGATTTCTGCTGTCGATAAATCGGGG

GAGGAAGCTGAGAAGTTTCAGGTGGCAGACAAATGCTTGGATGTCTCTCGTCATGTTGAG

GCTGAAGCACCCGTGTTGAGGAGAGAAATGGATGGAGAACTTGCAGAAATTGTGGAATCT

TGCTGTCACAGTATCAACGACAGGAATGACTTCTTGCAGAATGAGACGGAATCCGGATTG

AGCAACGGACTCAATGAATGCTTGTATTCTGATATGACAACTGATCGCAAACCTTTCTAC

GATGCTCAGATGCCATTAACGACGAAAGCAACAGGAGTAGAGTTTTCAGGGCTGGGATTT

AGTAACTTTAATGATCACTTTGAGGTCGGGCATGACCAATTAAACGAGGGTTTATGCGAT

CCAGAGATTGATATCAAGCCTTTTAGCCACGGTGTTTCTGATATCCCTGCATTAAAAGGA

GAGGGGAATTACGAGTATGTTCACAGTGACGTTCATGCTGCAAGCCAGTTACAGAGTGGA

AGGCCACCTGAACTCGCCAGCCAGTTTCAGCCCCCAGGAACCAATCCATCAGGGAGCTTG

AGCTACATTCAGGAGCCAGATTTAGGTGCGGGTTGTAATTTTGATCTATCAAATCAGGAC

ATCAACTGGGGATTAGTTGGAGAGCAAGAATTGCTCGATCCCTGGTTCCCTGAATTACAA

TTCTAG

>EVM0039575

ATGTTGGATCTCAATCTTGGTATCGATTACAGTGATTCATCCTGCGATAACAACAAGAAT

AATATGATGGTTATTGTTGATGTCGAGAACCACTATCACCAAGAAGAAGCGGCAGAAGCT

TCGAGGACTCATCAAATGGAAGATTCGGCTACTTCAAACTCCTCCGTCATCAACAATACA

GAAGATGAAAACTCCTCAAACAACAGCAACTCCGCTTTCATTTTTGATTTCCTGAAGAAA

GATGAAAACTTTACCAGTACAACCACCACTGATGCTTCAAAGCAGACAAGCTCAAACTGT

GATTTCACTACCCAACAACTTTCTCCTCAAATAAGTGGGCTGAAATTGAATTTGCAGCCG

GGCTTGGCTGTAAAAACAGATACAAGACCACAGTGGTTGAAGCTTTCACAGATTGGGTCT

AGTGGGGAGTCTGAACTGAAAATTGTGCAGCAAAAACAACAGCAAGCGAGGAAGAGTAGG

AGAGGACCAAGGTCTAGAAGCTCACAGTATAGAGGAGTGACGTTTTACAGGAGAACTGGG

AGATGGGAGTCACATATCTGGGATTGTGGGAAGCAAGTATATTTAGGTGGGTTTGACACT

GCTCATACTGCTGCAAGGGCATACGATCGAGCAGCAATCAAGTTTCGGGGAGTTGATGCA

GATATCAACTTTAATTTAAGCGATTATGAAGAAGATATGAAACAGATGAAAAACCTTAAT

AAAGAAGAATTTGTTCACATTCTTCGTCGCCAAAGCACCGGTTTCTCACGAGGTAGCTCA

AAATACAGAGGTGTAACTCTCCACAAATGTGGCAGATGGGAAGCTCGAATGGGGCAATTC

CTTGGAAAGAAGTATATTTATCTTGGATTATTCGACAGCGAAGTAGAAGCTGCAAGGGCT

TATGACAAGGCAGCCCTCAAATGTAATGGAAGGGAAGCAGTGACCAATTTTGAGCCTAGT

GTTTATAAAGGAGAGAAGGGTTTCGATATGAATCATGGAGGAAGTGGCCACAATCTTGAT

TTGAGCCTTGGAATTTCTCAGCCAACGAATGATTCTAAAGGGAATGGCAATTTGGGAGAC

GGCCACTGTAGTTATGGTGGCTGTGAAATACCCATCAGAGAGAGACAAGTGGCTGGGGGC

ACTGCTGCTGCACATATGGGTTTAGAGACGCTGCGTGGTCTACCAGTGGTATCGAAGAAC

CTTCTGACATGGTCTGGCATGCACCCTGGTTTAGCATCAAGCTACGAGGCTACGGAGAAG

AGGTTTGAAGCTGTTTCTTCACCGAGATTCTCAAGCTGGCCATGGCAAATAAATGGCAGC

AGCAACAATGTTGTTGCTACAATGCCACAGCTCTGTGTTGCAGCATCATCAGGATTCTCT

TCTTCGACGAAAACTGTTCCTCCAGCTTCTCTTCCACTCAACCAACAGAACCACTTTGCC

AACAACCTGCGACTAACTGCATTCACCACATCTACCGCCAACCCCTTTTACTCTTATATA

AGCTAA

>EVM0038265

ATGAAGTCCATGAACAATGATGATGATAGCAATAGCCATAATGCAAACTGGTTAGGCTTC

TCTTTGTCCCCTCAAATGAAAATGGAGGTTCCATCAGGTGCTGATCACCATCACCAAACA

CAGTCTCCTTCTGCAGCTATTCCTACTGCAATCCCATCAGGTTTTTTCCACTCCCAGATA

CCACAACTCAACTATGGCATTTACTATGGCGTTGATGATCAGAGTGGAAATGGAGGTTTC

TACTCTCCTTTGCCTGTTATGCCACTCAAGTCTGATGGCTCACTTTGTATGATGGACGAT

CTCACAAGAACACAGCCTCAAGGTGAAGCAGCAATGGTAACTACTTCAACACCAAAACTA

GAGGACTTCTTTGGTGGTGCAACTATGGGGACCCATCACTTTGAAAGCAACGATAGAGCA

GATATGGCTCTTAGCTTAGACAGTAGTCCTAGTATGTATTTTCACCAAGACCCAGACCAT

GAGCCCGACAGCCAAAATAGTCTAAATCATCTTCAACAAAACCCCAGACAACAACAGCAT

CACCACATTCAACTTCAGCACTACCCGTATTATTCTTACTTTAGGAACAATGAGATGTTA

GTTGGAGAGGAAGCTAAACAAATGGCCCAGGCGTCAGATTGCGGTCTTAGGCTTCCAAAC

ATGGGAAATGAAGGGATAACTGGCATGAAGAATTGGGTTTCGAGAAACTATCAAGGTAAC

CGTGCCATGGAGCAGAAGATGCTTAGATCCATGGTAGAAAATGGAGGCGAATCTGGGCCA

AATATCAGTGCAATGACATATGGGGATTTGCAGTGTTTGAGCTTGTCAATGAGTCCTGGC

TCCCAGTCAAGCTGTGTTACCGGTTCTCTGCAAGTCTCGCCTTCTGTGGCTGACTGTGCA

GTCATGGAAACCAAGAAAAGAGGGCCTGAAAAGGTGGATCAGAAGCAAATTGTTCATAGG

AAGTCTATTGATACATTTGGGCAAAGAACTTCTCAATTTAGAGGTGTTACAAGGCATAGA

TGGACTGGGAGATATGAAGCTCATTTATGGGACAACAGTTGCAAGAAAGAAGGTCAAAGC

AGGAAAGGAAGGCAAGTTTATCTAGGGGGTTATGATATGGAAGAAAAAGCTGCAAGAGCT

TATGATCTAGCTGCACTCAAGTATTGGGGACCCTCAACTCATATCAATTTTCCTCTGGAA

AATTATCAAAAAGAAATTGAAGAAATGAGGGACATGAGTAGGCAGGAGTACGTTGCTCAT

TTGAGGAGGAAGAGCAGTGGATTCTCTAGGGGAGCTTCAATGTACAGAGGAGTTACAAGA

CACCATCAACATGGAAGATGGCAAGCTCGGATTGGTAGGGTTGCCGGAAATAAGGACCTT

TATCTGGGAACATTCAGCACCCAGGAGGAGGCAGCTGAAGCCTACGATGTGGCTGCTATA

AAATTTCGTGGAGTTAATGCTGTGACCAACTTTGTCATAACAAGATATGATGTGGAACGA

ATCATGGCTAGCAGTACACTTCTAGCTGGAGAACTGGCCAGGCGAAACAAAGACATAGGA

CCTTGCGATGATACTTCTACCAATCACAGTATTCCTTCTACTCATAACAGCAACGGGGAA

TCCTTGCCCTCGCAGAAGAACAGTGAAAACGAATCAGATTGGAAAATGGCTCTCTATCAG

TCATCACAGCAACTGGATCAAAAGGCCTCGAATGTTATGGATAATTACAAAACTCAGGCT

TTCTTATTGTCTCCCGAAAATGTAATTGGATTTGATTGCATGAGCTCAGCTCACCAGCAC

GAGTTGGAGGACCCGTCCAAGATGGGAGCTCACATGTCAAACGCTTCTTCATTGGTGACC

AGTTTAAGTAGCTCTAGAGAAGAAAGCCCTGACAGAGCAGGCCTGCCGATGCTCTTTGGA

ATGCCTCCCTCCGCAGCATCCAAGTTGTTCGCTAGTCCGACTAGCGATGTGAACTCTTGG

ATCCCAGCAGCATCTGCCCAACTGAGGCCTGCAGTATCATTGCCTCACGTCCCAGTTTTC

GCTGCATGGACAGACGCGTAG

>EVM0036168

ATGTTGGATCTCAATCTTGGTATCACCTCTAGCGATTCATGTTGTGACGATAACAACAAG

AACAATATGATGGTAATTGTTGATTCTGGGTACCACCAAGAAAAAGAAGCAGCTTCAAGG

AATCAACAAATGGAAGATTCAGCTACCTCAAACTCCTCCATTACCAACACTACCGAGGAT

GAGAATTCCTCAAACATCAGTAACTCTGCTTTCATTTTTGATATCTTGAAGAAAGATGGA

AACTTTACCAACACAGGCACCATTATGGCTACAAAAGAAACAAATTCGAACTGTGATTTC

ACAACTCAACAAATTTTTCCTGGATGCACCGGGCTGGGACTGAATTTCCAGCCAGGATTG

GCTGTAACATCGGGTACAAGACCTCAGTGGCTGAAGCTTTCACAGATGGGGTCAAGTCCA

GAGGCAGAGCTGAATGTGCAGCAAAAACAGCAGCAAACGAGGAAGAGTAGGAGAGGACCA

AGGTCTAAAAGCTCACAGTATAGAGGAGTGACATTTTACAGGAGAACGGGGAGATGGGAG

TCACATATATGGGATTGTGGGAAGCAAGTATATTTGGCAGGTGGATTTGACACTGCTCAT

GCTGCAGCACGGGCATATGATCGAGCTGCAATTAAGTTTCGGGGAGTTGACGCAGACATC

AACTTTAATTTGAGTGATTACGAAGAGGATATGAAGCAGATGAAAAACCTTAGTAAAGAA

GAATTTGTCCACATTCTTCGCCGTCAAAGCAACGGTTTCTCACGAGGTAGCTCAAAATAC

AGAGGCGTGACTCTTCACAAATGTGGCAGATGGGAAGCTCGAATGGGGCAATCCGTTGGA

AATAAGGCTTATGACAAGGCAGCCCTCAAATTTAACGGAACAGAAGCAGTGACCAATTTT

GAGCCTAGCGTTTATGAAGGAGATATGATTTCCGATCCTAACAATGGAGGAAGCGGCCAC

AATCTTGATTTGAGTCTTGGAATTTCTCAGGCATCAAATGATCCCAAGGGGAATGACAAC

GTGGGAGACCTCCATTGTCGTTATGGTGGCTGTGAAATGCCCAGCAAAGAGAGACAAGTG

GTGGAGGGCGCTGCTGCTGCACATATGGGTTTACAAACCCTTCACGGTTCACCATTGGCG

GCAAAGAATCTTCCGGCATGGTCTGGCATATACCCTGGTTTATTACCAAGCTACGAGGAA

AGGGCTACGGAGAAGAGGGTGGCAGCTGTTTCTTCACCGATATTCTCAAGTTGGCCGTGG

CAAATAAATGGCGGCAACAGCGTTGCTGCTACAATGTCACAGCTTTCAGTTGCAGCATCA

TCAGGATTCTCTGCTTCGACGATAACTGCCTCTTCAGCCACCCTTCCATTCAACCACCAG

AACCATTTCGCCGGCAACCTGCGTCTCTCTGCACCCACCCCATCCGCCACCAACCCCTCT

ATTTACTCTGATAAGAGCTGA

>EVM0032156

ATGGACAATTCATCTCTCTCTCATCCTCCCCAAGAACCCACCACCACCACCATCACAATA

CCATCCAATGACAAAAGCACCGATAACATCACCACCGCAACCACCCCCACTACCGCCACA

ACAAGTGACACAAACAGTAACAACAGCAGCAGTGGCTGTAGCAGGAAGTGCAAAGGCAAA

GGAGGACCAGACAACGGTAAATTTAGATACAGAGGAGTCAGGCAAAGAAGCTGGGGAAAA

TGGGTAGCGGAGATCCGTGAGCCAAGAAAACGAACCCGTAAGTGGCTTGGAACTTTCGCT

ACCGCAGAGGACGCGGCACGAGCCTATGATCGAGCAGCCATCATCCTTTATGGCTCTAGG

GCTCAACTCAACTTGCAACCCTCAGCCTCCTCATCTTCTGCACAGTCCGGATCAACTTCT

CGCAACTCTACCTCTTCCTCGAGCCAGACTCTTCGCCCTTTGCTCCCTCGTCCCCCTGGG

TTTGGCTGTGGCTTTGGTTTCACTTTCTCTCTCTCAAATCCAATGGCTTCTCCGGCTGTC

ACGGCAGCTTCACCGGGATTTACTCCATACGAGGTTAATTGTCTTTCGAATAATGTCGTT

GGATCAGCCTTACAGTGTGCTAGTACTAATGAAATGCCAGGGCAAAATCAACAGCAAGCT

ATGTTACAACGCTATCTCATTCAACATGGGGCAAATGCAACCAACCCCGATAATATATTT

GTTAGTTCTAGTGTAGCAACGACAACCTCGTATCAAAATCATTGTAATCGTCTGCCGCAG

CATCATGCATACGATGATGTTAATGCGTTGGTGGGTTCCGTCGGGTCGGGTTTTTCTCTG

TCTGGTAGCAATACTCCCGTTGTTGCACCAGTGGGTCATCCTCTGCAGGATCCGGCAATG

TATGCTGGACCTGGATCTCCATCTGTGTGGAATGATGACGAGTACCCGCCTCCTAGTATT

TGGGACGATGAGGACCCTTTCTTGTTTGATTTTTGA

>EVM0035890

ATGGGTACTCTAGATCAAGATTCTAAAGCTACTTCTTTGTCCACGGATTCTACTAAGAAG

AGGAAAAGGGCGAGCAATAAATCTGTAGCGGAGACCCTTAAAAAGTGGAAGGAGTATAAT

GAGTATCTTGATTCTCAAGGTGATGGAGGTAATAAACCAGCTCGTAAAGTTCCGGCCAAG

GGCTCAAAGAAGGGGTGCATGAAAGGTAAAGGGGGACCAGAGAATTCAGTCTGCAATTTC

AGAGGTGTTAGACAGAGGACATGGGGGAAGTGGGTTGCTGAGATTCGGGAACCAAACAGA

GGCCCTAGGCTATGGCTTGGTACTTTTCCTACTGCCTACGAAGCTGCTCTTGCCTATGAT

GAAGCTGCACGAGCCATGTATGGTCCGTGTGCTCGTTTAAACGTTCCTGATGTGCTGAAT

TCAACAAGCTCATTGAAGGACAACTTCTCCCCGGCAACACCATCTTGCTGTTCTCCAGCA

GCAAGTTCTGCAGACTCTGCTACTACATCAACCCACTCCGAGGTGTGCGTGTATGAGGAT

CCTAAGCAGAATGTATCCAGCCAAGCTGAGGTTCTTGCGCATCATATATCGAGCCAACAT

CATATTGAGGACGGTTCGCAGGGAGTTGATATATCAAGCCAGCAACCTATTGAGGATGGT

TCGCAGGGAGTTGATATATCAAGCCAACAACATATTGAGGATGGTTCGCAGGGAGTTGAA

AATAGCACACCGGGGGACGAGCTGAGGAGTCAATTAGAAAATCCTTTGTGGACTCCTTCA

GAAATGCCCTTGTGGACCAATGACTGGCACAACTACTCGCTCGATGAAATGTTTAGCTTT

GATGAGCTGCTAGGCGATATTGATGCGGGAAGTATGGGGGCAGAAGGGTACTTCAACTTG

GGATTTTGA

>EVM0007616

ATGGCCTCCATGAATAACTGGTTAGGTTTCTCTTTATCCCACCAAGAACTTCCTTCATCA

CAATCTGACCACTCTCAAAACACAGACTCTCGCCTTGGTTTCCACAGTGATGAAATCTCT

GGTACCGATGTCTCTGGCGAATGTTTTGATCTCACTTCCGATTCCACCGCTCCTTCTCTA

AACATTAATCCTGCTACTTTTGGTATACTTGAAGCCTTTAGAAACAACCAATCTCAAGAT

TGGAATATGAAGAGTTTAGGCATGAATCCAGACACTAACTACAAAACCGCCTCAGGCCTC

CCTATATTCATGGGTACTTCATGCAATAGCCAAAACATTGATCAAAATCAAGAACCTAAA

CTCGAGAACTTCCTTGGTGGCCATTCTTTTGTCAATCATGAACACAAACTGAATGGCTGC

AACGCCATGTATAATACCACTGGAGAGTATGTATTCCAAAACTGCTCTTTGCAACTTCCA

TCTGAGGCGACATCAAATGAAACAATCAGGAGCAATGGAGGAGGTGAAAATAAAAATAGT

TCCATTGGGTTATCCATGATCAAGACCTGGTTAAGGAACCAGCCAGCACCGACCCAACAA

GACACCAATAATAAAAACAGTGGTGGTGCACAAAGTTTGTCTCTTTCAATGAGTACCGGG

TCGCAACCAGCTGCTTCTGCTTTGCCACTTCTAGCGGTAAATGGAGGAGGTAATAGTACT

GGGGGAGATCACAGTTCTTCTTCTGATAAAAACAAGGAACAAAAGACCAAACCGAGTCTT

GATAGCCAAACCGTTGCCATCGAATCGGTGCCAAGGAAATCTATCGATACTTTTGGCCAA

AGAACATCTATATACCGTGGTGTAACAAGACATAGATGGACCGGTAGATACGAAGCTCAT

CTATGGGACAATAGCTGTAGAAGAGAAGGACAAACTCGGAAGGGAAGGCAAGTTTATTTG

GGAGGTTATGACAAAGAAGATAAGGCAGCTAGAGCCTATGATTTAGCAGCATTGAAATAT

TGGGGCACGACTACTACGACAAATATTCCAATTAGCAACTACGAAAAAGAGATAGAAGAA

ATGAAGCACATGACTAGGCAGGAGTACGTTGCGTCTCTTCGAAGAAAAAGTAGTGGGTTC

TCTCGGGGCGCTTCTATATATCGAGGTGTAACTAGACATCATCAGCACGGAAGATGGCAG

GCAAGGATTGGAAGAGTTGCGGGGAACAAGGACCTTTACTTGGGAACTTTCAGCACCCAA

GAGGAAGCAGCAGAGGCCTATGACATTGCGGCCATAAAATTCCGTGGGCTGAATGCAGTG

ACCAACTTTGACATGAGCAGATATGATGTTAGCAGCATACTCGAGAGCAGCACATTGCCG

ATTGGAGGCGCGGCTAAGCGGCTGAAAGAGGCGGAGCATGCTGAAATAACAATGGATGTT

GCACAAAGAACAGATGATCATGACAACTTGAGCTCACAACTCACCGATGGAGCTGGCAAC

TACGGTGCAGTACACCATGGCTGGCCTACTGCTGCATTTCAGCAGGCACAGCCTTTTAGC

ATGAACTATCCATATGGCCAGAGGCTTTGGTGCAAGCAAGAACAGGATTCTGACAATCGC

AGCTTTCAAGAAATTCATCAACTACAGCTGGGAAATACCCACAATTTCTTTCAGCCTTCT

GTTTTACATAACCTAATGAGCATGGACTCTTCTTCAATGGAACATAGCTCTGGTTCTAAT

TCTGTCATGTATAGCAGTGGAGTTAACGATGGCACAAGTACGGGAACCAATGGAGGCTAT

CAGGGAATTGGTTATGGAAGCAATGCTGGGTATGCCGTCCCCATGACTACGGTCATATCT

AATAATGACAACAACCAAAATCAAGGAAATGGTTATGGAGATGGAGAGGTGAAGGCTCTG

GGGTATGAAAATATGTTATCCTCGTCTGATCCATATCATGCCAGAAACTTGTATTATCTT

TCACAGCAATCTCCGGCCGGTGGAATCAAGGCTAGTGCATACGATCAGGGTTCAGCATGC

TACAATTGGGTGCCAACGGCTGTTCCTACCGTCGCAGCAAGGTCTAACAACATGGCTGTT

TGTCATGGAGGGCAACCTTTCACTGTCTGGAATGACGGTACATAA

>EVM0017733

ATGCGTTTTCTATCCCCTAATGAATTACAACCCATACTTATCTCCAACCCCACCATGTCT

GTTCAACCAAAAGTCAGATTTACTAAACATGTACTCGCCAAGAACCAGCAGCTGCATCCT

TCTTCAAGAACCCGCCAAAGACTTGTTAGAATCATCCACACGGACCCTGATGCCACTGAT

TCCTCCAGCGATGAAGAAGAGGAAGAGCAAGAGAGGAAGTTTGTCAAGATAGTGAAGAGA

CAAGTGAGTGAAATAAGCTTTTTAGAGCAAGACACAACAGCACCAGCAGCTCCTTCCTCC

GCAACTTCAAATGATGATCAGAAACCAAGAAGAAAAAGACCTTCTTCAAGATTACCGGTA

CCTGACGTCTCTCGTCAGAAAAAGTTCAGGGGGGTCCGTCAGAGACCCTGGGGAAAGTGG

GCTGCCGAGATCCGTGACCCGAACCGACAGAAACGTGTCTGGCTCGGCACTTTTGACACT

GCAGAGGAAGCTGCTACAGTATATGATCGCGCTGCGGTGAGACTAAAAGGACCCGACGCC

GTTACCAACTTTCGTACTAGCACTGCTACAGCGGAGAGAGCTAACGTTAACTCTGGTTCA

AGTGATGTGGAGTGTGAGTCATTTGATTCGCCGTCTAGCAGGGGTACCGGTACCGATTTG

GCATCGCCCACATTAGCCCCCCGTCAAATAGAGTTGACGCCGTTTGACCTTGTTTTAACG

GAGAAGGCTAAGGTTGCTGACGGTCAGCGCGAGTCATGTGAGTCGTCGTTAAGTTGGCTT

AACGACATGTTATCACCAACGTCTGTTCTCCATTACGACGAGTTCACGCAGTTTGATAGT

TTGGGCTACTGTGACGTTGACGCTTTTGGGTTCGATATTGACGTGCCGTTAAGTTTGCCA

AATTCTAAGGAAGAATTCGTTGACCTCGATGATTTCTTGGTGGATGCCATGTGTTAA

>EVM0000005

ATGGAAAAACAAATCAACCTTGAGACTGATGCTCAAGACAATCCCCCCAACTCAGCTTCC

ACTACCACCTCCGCCACCAAGAGGACCACCTCCGCCTCCTCCACGCCCATTTCACACACT

AACCGAAGCTCCAAGAAAGAAGAGAATTCTATAGCTATAAAAGTATCAAGAAAAGCTCAA

GAATTACAAAGTGGCAACGAGGGTAGTAAAAGGCGAAAGACCGCCGAAAATGAGAATAAC

GGGAAGCATCCAACATATAGAGGAGTAAGGATGAGAAGTTGGGGCAAATGGGTGTCTGAA

ATCAGAGAGCCAAGGAAGAAATCAAGAATATGGCTTGGGACATACCCTGCAGCTGAAATG

GCAGCTCGAGCACATGATGTAGCCGCGTTAGCCATCAAAGGCAGCTCAGCTTATCTCAAT

TTTCCTGAATTCGCTCACGAACTTCCTCCTCCACTCAGCAAGTCCCCCAGGGACATTCAA

GCTGCAGCCGCCAAGGCAGCGGCTGCTTCGTTCACTGAAACAAGATACTGTGAAGGTGGG

GGCGAAGCCGAGCTTAACGTTTCGAGTTTATCAGATAGTTCGGCCATGGACAATACACAA

AAATCAAGCGGTTCTCCTTCTACTGATTTTGATGATACATTGTTTGACCTTCCTGATCTC

TTCAACGATGGCGTTCATCACAGTGATGGGTTTTGCTATTATTCTTCATCATGGCAATTA

TGTGCAGCCGATACTGGCTTCCGGCTTGAAGAGCCATTTTTATGGGAGTGCTAG

>EVM0055854

ATGGCCGGCTCCAAAACCTTCGATAAGCCCCTGGACGGGTACGATGCCCTTGACATTCAT

GCAGGCTTTGCGATCCTTCAGAGAAACACGACCCCGCCTCAAGCTGGCGAGAGGCGAGGA

AGAAGAAAGCAAGCCGAGCCGGGGAGGTTTCTTGGAGTCAGGAGGAGACCTTGGGGGAGA

TATGCTGCTGAAATCAGAGACCCAACAACAAAAGAAAGGCACTGGCTCGGCACCTTCGAT

ACGGCTCATGAAGCGGCTCTAGCTTATGATAGGGCTGCCCTTTCCATGAAAGGCACCCAA

GCAAGAACCAACTTCACTTACTCTGATAACACAACCTTTCACTCCCTCGTTACTCCATTT

GACATTCAAGCCTTCTTGCCACAATCACAGTTTCTCACTAACACTCAGATTAAACACTCA

ACCAATCAGAACAGTCCCGTTAAGCTTGAAATCTGTCAAAATAAGACCCCAATTAGTAGC

AGTACGTATGCTGAATCTTCTGGTGGATCAGCTCATGAAGCTAGCGATTTCTTTTTCTCG

AATGAGGACTCTAACTCAGGTTATCTGGGCTGCATTGTTCCTGATAACTGCTTGAGGCCA

CCTCCCTCAGGCCCCACTGGTAAAGCCTCCAAAACCAGCACATCCAATGCTTCAAATGAT

CGAAACTCTTGCTTCATGACCACAACTAATTACCTTGAAGATCATCCGCAATGTAGCAAG

TACGCGCCTCCTCTCGATATCACAAATGTGCCGACCAAGGCATATAGTCCTGTAAACTTT

CCTGGCTTTGACGAGCTCAACAATGGATTTTGGGGGGAGCAACCGTCGTGGGATGGCAAT

TCCGGTGACCTCTCAGCACTGATGAACAACCCATCAATGATGGAAAATGTATGCATGGAT

ACCTCGCATGGCATCGACAATCCTCCTGCTTGTGGCCTAATGCCTCAAGCTACTTCTTCA

GTATCATATCCAGCTTATGGCGATGCAGCTAGCTTTGGATATTCACTCTTTTGA

>EVM0006536

ATGTTGGATCTCAATCTTGGTATCACCCACAGTGATTCATCCTGCGATGATAACAAAAAG

AATAATATGATGCTTTTTGTTGATGTCTTGAACCACTATCACCAGGAAGAAGCGGCAGCT

TCGAGGACTCATCGGATGGAAGATTCGGCCACCTCAAACTCCTCCGTTATCAACAATACA

GAAGATGAAAACTCCTCAAACAACAGTAACTCTGCTTTCATTTTTGATATCTTGAAGAAA

GATGAAAACTTTACCAGCACAACCACCACTGATGCTTCAAAGCAGACAAACTCAAACTGT

GATTTCACCACCCAGCAACTTTCTCCTCAAAGAAGTGGGCTGAGATTAAATTTGCAGCCG

GGCTTGGGTGTAACAACAGATACAAGACCACAGTGGCTGAAGCTTTCACAGATGGGGCCT

GGTGGGGAGTCTGAACTGAAAATTGTGCAGCAAAAACAACAGCAAGCGAGGAAGAGTAGG

AGAGGACCAAGGTCTAGAAGCTCACAGTATAGAGGAGTGACGTTTTACAGGAGAACTGGG

AGATGGGAGTCACATATCTGGGATTGTGGGAAGCAAGTATATTTAGGTGGGTTTGACACA

GCTCATACTGCTGCAAGGGCATACGATCGAGCAGCAATTAAGTTTCGTGGAGTTGATGCA

GATATCAACTTTACTTTAAGCGATTATGAAGAAGATATGAAGCAGATGAAAAACCTTAAT

AAAGAAGAATTTGTTCACATCCTTCGTCGCCAAAGCACCGGTTTCTCTCGAGGTAGCTCA

AAATACAGAGGTGTAACTCTCCACAAATGTGGCAGATGGGAAGCTCGAATGGGGCAATTC

CTTGGAAAGAAGTATATTTATCTTGGATTATTCGACAGCGAAGTAGAAGCTGCAAGGGCT

TATGACAAGGCAGCCCTCAAATGCAATGGAAGGGAAGCAGTGACCAATTTTGAGCCTAGT

GTTTATAAAGGAGATAACGTTTTCGATACGAATCATGGAGGAAGCGGCCACAATCTTGAA

TTGAGCCTTGGAATTTCTCAGCCAACGAATGATTCGAAAGGGAATGACAATTTGGGAGAC

GGCCATTGTAGTTATGGTGGTTGTGAAATGCCCATCAAAGATAGACAAGTGGCTGGGGGC

ACTGCTGCTGCACATATGGGCTTAGAGACGCTTCGTGGTCTACCGGTTGTATCGAAGAAC

CTTCTGACATGGTCTGGCATGCACCCTGGTTTAGCATCAAGCTACGAGGCTACTACGGAG

AAGAGGTTTGAAGCTGTTTCTTCACCGAGATTCTCAAGCTGGCCATGGCAAATCAATGGC

AGCATCAATGTTGTTGCTACAATGCCACAGCTCTGTGTTGCAGCATCATCAGGATTCTCT

TCGTCGACAAAAACTGTTCCTCCAACTTCTCTTCCATTCAACCAGCAGAACCACTTTGCC

AACAACCTGCGACTAACGGCATTCACCACCTCTGCCACCAACCCATTTTACTCTTACATA

AGCTAA

>EVM0039635

ATGGCCAGACCACAACAGCGATATCGAGGGGTTCGACAGAGGCATTGGGGATCGTGGGTG

TCTGAAATTCGCCACCCTCTATTGAAGACTAGAATTTGGCTAGGCACGTTTGAAACTGCA

GAGGATGCGGCAAGAGCCTATGATGAAGCTGCAAGGCTTATGTGCGGACCAAGGGCAAGG

ACCAACTTCCCTTATAACCCAAATGCATCGCAGTCAGCTTCGTCAAAACTTCTTTCAGCA

ACTTTGACAGCAAAGTTGCATAGGTGCTATATGGCCTCATTGCAAGTGACTAAACAAACA

TCAATTCAAGAACAAAAACAGCAGAAACAAAAGGCACCAACCTCGCTTGCCAACATCCCT

ACCAATGGCATTGTCATAAAAAGTGAGGAACTTGATGCTTTGTTTTCAGAGAAAAGTCCA

TTGCAAGTTCAAGAGGCAGAGGGAAACTGGGTGTACAAGAAAGTTCAAGTTGACAACAAC

CAGCAGTTCATCAAGCCTCTTGAAGATCACCACATTGAGCAAATGATTGAAGAGTTGCTT

GATTACGGGTCTATTGAGTTATGTTCTGGGGTAGCAACTCAGTAA

>EVM0044041

ATGGAGATGACAAGGAATACTGGCGATCAGATAAGCCCAGGAAGGCTTCGCTTGTGCATG

ATAGAAGAAGAAAGGAGCGCTGGGGAGACTGGTAAGTGTATTAAAAGACGACGAAGAGAC

CCTTCTGCGTTTGCATTGAGCTGCAATATTATTGATCAACAAAGTGACCAGCAGCAGCAG

CCACAGTCACTTGGTGATCGAACGGAAGCTGTTGCCACTACAGTCAAGAGAAGTTCAAGA

TTCCGGGGAGTTAGCAGGCACCGTTGGACGGGACGATTTGAAGCTCACTTATGGGATAAA

GCAACTTGGAATCCAACTCAGAGAAGGAAGGGTAAACAAGTTTATCTCGGAGCTTATGAT

GAAGAAGAATCCGCTGCAAGAGCATATGATAATCATTTAGCTGCTCTCAAATACTGGGGG

ACCTCAACTTTCACAAATTTCCCGGCGTCTGACTACGAGAAAGAAATTGAAATAAAGGAA

ACGGTAACTAAAGAAGAACATCTAGCCTCCTTGAGAAGGAGAAGCAATGGGTTTTCAAGA

GGTGTGTCCAAATACAGAGGAGTTGCTAGGCACCATCACAATGGGAGATGGGAAGAAAGG

ATAGGGAGAGTTTTTGGAAACAAGTATCTTTATCTTGGCACGTACAGCACTCAAGAGGAA

ACTGCTCGTGCGTATGATATAGCAGCCATTGAATACAGAGGGATCAATGCTATAACCAAT

TTCGATTTAAGTACATACATCAGATGGCTGAAGCCAGAAGCCAGTCTTCCTGCACCTCAA

GAATCGAAACCAACCTCAGCCCCCCTGCCAATGACAGCCTTCTCCAATCACTTTCCAAGC

GAGAAACCAACCCAGCTATCCGTTCTCCAGATGGACCCTTCTTTAATAGATAATTTAAAC

ACCCCTAAACACGAGGGCATTTTTCACAGGAAGACGCTTCCCGTTGATCTTGTGGAGAAG

AACTTGAGTACCACATTCGAAGAAATTGATTCGGAAAACCCGAATAATAGCAACAACAAC

GCTGGGGAAGCATTCTATGAAGGATTCAGCCGCACGGCAGGGAGAGGCAAATACTTGCCA

CCATACAACAGAATGAAGCATTCTCTTTGGAATGGAGCCTTGAGCATGCCTTCTCGTTTC

CATTAA

>EVM0042467

ATGGAGGAAGCGCTTAGAAGGCTCGACGGAATACCGAGCCATACACTCGAAACCAGCCTA

CACGAACCCACCATCGCGGATCTCCAGAAGAGATCTGCACTTACCAGCACGGCTAGCACT

GCAACAAACAAAAAGGCACTGAAAGAAAGTGGTGGAACTGGTGGGAACATGAGGTACCGT

GGCGTCCGCAGGAGGCCATGGGGTCGTTATGCAGCCGAGATAAGGGATCCTCAGTCTAAG

GAACGGCGTTGGCTTGGCACTTTTGACACTGCTGAGGAGGCTGCTTGTGCCTATGATTAT

GCAGCTCTAGCAATGCGTGGTCTCAAGGCTCGGACAAATTTCCATAATATATCGTCTTCA

AACTCTTCTCTTGGTCACCTTCATGTTGGTGACTTCTCTGCTGGTGGGTCTGCACCGCAG

AGGAGCACTAGTGACAGTATTGCCAGTACTTCTCTGCACATGCTTTTTCTCCGTGATTTC

ATTCACTCCTCTTCTGGTTCGACATCTCATAATCATCCCCAACCTTTTTCTGATCACTAT

CCATGCATCGAAGGGTCCTCCAATTCTGATCCTGTCAATTTCCCAGGTGGTTCTTCGGTG

AATCCTTCTAGTGACACTATTTCTAGCTCGTTTAATGTCTCTTTGGCAGACTCAACCGTC

ACTACTCTACCGCACATGGAGTTTCATCAGAATTATACTACTCTTGGAGACCCGAAAAGG

GTTGACGCTAAAGCTGAAGAGTTGGAATTCTTTCCACAGGAGCCATCCGATTCTGGTTTG

TTGCAAGAGATTATTCAAGGGTTTTTCCCTAAACACTCCCCTGACAAGATTGCGTCTTCA

GAAAGCTCTGGAGAGTCAATAGCTGCACCTTCTGAAATAATACCTCTTAATCAGTCACTA

GATAGGTCGAGAAGTGGCATGAAAAAGTACTTTGGTAAAAATGAGCACCATGATCCTTAT

CTTGGTCGCCAAGGAAGAAGGTCGGGGCAGCTGATTGAGGGTTCTTACAGTGGGGTTAGC

TCTCGTGAACTTCCGCACTGTAATGAGATGGCATTGATCAATCATCAGATGGGTCCAGAC

TCCATTTTAGATGACATTTTTCAGTACCCAGATCGCATGAGTGCCTTTGCAGCTAGGTTC

CAGAATGTTTGA

>EVM0032803

ATGAAATCTATGAGTAATGATAGCAATGGAAGTAGTAATAACTGGTTAGGCTTCTCTCTC

TCGCCCCACATGAAAATGGAGGTTGATTCTGACCTCCAACACCATAATCAGTATCATCAT

CAAACCCAAACCCAAACACATGCTGCTGCTGCTGTTCCAACAAGTTTCTATCTATCTCCT

TCTCTCTTTAACAGTTCTGAAATCTGTTATGGAGTTGAAGAAAATGGTGGCTTTCAGTAC

TCTCCTTTGTCTGTTATGCCCCTCAAGTCTGACGGGTCTCTTTGTATCATGGAAGCTCTC

ACTAGATCACAGCCTGAAGGAATGGTGTCGAGTCCGTCACCAAAACTTGAAGACTTCCTA

GGTGGTGCAACTATGGGAAATCCTCAGTACGGTAGCCATGAAAGGGAAGCCATGGCCTTA

AGTCTAGACAGCGTATATTACCAGCAAAGCTCAGAGCCAGTAACTAACAGACAACATTCT

CTCTATAAGCAACAAGACCAACAGTTTTCATTTCAAACCCATCCATACTACTCTGGGATG

GCATGCCCAGGTTTGTATCAGGCCTCACTGGAGGGAGAAACCAAGGGTACTCAGCTTGCA

GACTGTAACTCACCTATTCCTCAAATGGCAGACAATGAGCTGCCTTGCTTGAAAAACTGG

GTCGCTAGACATTATTCCTCGCAAAATGCACTGGAGCAGCAGATTAATAGTGGCACGGCT

GATGATGGTGGGGCTTCTTGCTCTGCTAGTGCGATGGGTTGTGGGGATTTACATTCTCTC

AGCTTGTCTATGAGCCCTGGTTCGCAGTCAAGCTGCATTACAGCTCCAAGGCAGATCTCA

CCTGCTGGCACTGAATGTGTGGCCATAGAAACAAAGAAGAGAGGCCCTGTAAAAGTGGAT

CAAAAACAGCCTGTTCATAGGAAGTCTATTGACACATTTGGCCAAAGAACGTCACAGTAT

AGAGGCGTTACAAGACACAGGTGGACTGGTAGATATGAAGCCCATCTCTGGGACAATAGC

TGTAAAAAGGAAGGCCAGACCAGGAAAGGAAGGCAAGTATATCTGGGGGGTTATGATATG

GAAGAAAAAGCTGCAAGGGCTTATGATCTTGCTGCCCTTAAGTACTGGGGATCTTCAACT

CATATAAATTTTCCGTTAGAAAATTACCAGGGAGAACTTGAAGAAATGAAAAACATGAGC

CGACAGGAATATGTTGCACATTTACGGAGGCAAGGCAATGGTTTTAACATTTTTTCTCCT

TCGCTTTACAGAAAAAGTAGTGGGTTTTCTAGGGGGGGCTCAATCTACAGAGGAGTAACA

AGGCATCACCAGCATGGAAGATGGCAAGCAAGGATAGGCAGGGTTGCAGGAAACAAGGAC

CTTTATCTCGGGACATTCAGCACCCAAGAGGAAGCTGCTGAAGCTTATGATATTGCTGCC

ATCAAATTTCGCGGGGTTAATGCTGTGACAAACTTTAACATAACAAGATACGATGTTGAT

AGGATCATGGCCAGCAACACACTTCTAGCCGGAGAACTAGCCAGGCGGAACCGAGAAACA

GTATCTAGCACTGAGGCCAATCACTATCAGAACAATGGTGTTTTATACCAGTCTCCGCAG

GAACAGCCAAATGTCTGTGGAGAATCTCTTGATCAGATGTCAATGAGTGTTGGGAATTAT

CGAAGCACCTCTTTCTCAGTGGCAGTGCAAGATCTTATGGGCATGGATCATTCAGTGAAC

TCAAGCCAGCCAGTGGCAGATGAGTCAACCAAGCTAGGAACTCATCTCTCGAACCCCTCT

TCTTTGGTGACCAGTTTGAGCAGCTCTAGAGAAGCTAGCCCTGACAAGACTGGAACCCCC

ATGCTGTTTGCTAAAACTCCACTGGCATCAAAGTTCATTGGCCCTACAACCAACGTTACT

CCTTGGATCCAAGCATCAGCCCAATTGAGGCCACCAGCAATCTCCATGGCTCACTTGCCA

GTATTTGCTGCATGGAATGATACCTAA

>EVM0016157

ATGGTAAAACCTGGAACGTCATCTTCGTCAGTTTCATCTGAGCAGCAGGCTGATAGAACC

CAGGAGCCCAAGTACAAGGGTGTTCGAAAGAGAAAATGGGGAAGGTGGGTGTCTGAAATC

AGGCTACCAAATAGCCGGGAGAGGATTTGGTTAGGATCCTATGACACGCCTGAAAAGGCG

GCGCGTGCATTTGACGCTGCCTTGTATTGCTTACGCGGTAGTGGTGCCAAGTTCAATTTC

CCAGACAATCCACCGGACATTGTCGGTGGGAGGTCACTTAGTCCGCAAGAGGTACAGGAA

GTTGCTGCCAGGTTTGCTAATGAAGAGCCGGCATCGTCCAGTGCTGCTATGGGAGGTGAA

TCATCGTCACATGTGGAGAACTACACATCATCGTCTTCTGACGGCGGGGCTGGACAAATG

GATAGTGGTACAATAGATTGGACATCGTTTTTGAATATGTTGGATTCTAATGAGGGTGCA

TCAGATTTTGGACTCTACCATGGGCTGGACCATATGGGCGGTGATTATTATACCCCTCCT

GCCCCACCGCCGCCGCCACCTGATTATTATAACGAGGATGATAACAATGGAGACGAAAAC

TACTCTCACCAGTCATTTCTGTGGAACTTTTGA

>EVM0051790

ATGGTGAAGTACAGTGCCGGGGAGCCAATTGCAGAGAGAAGAGACTCGAAATTCAAGGGT

GTTAGAAAAAGAAAATGGGGCAGGTGGGTGTCTGAAATCAGGCTACCGAATAGCCGGGAA

AGAATCTGGTTAGGATCGTATGATTCTGCAGAAAAGGCTGCTCGAGCGTTCGATGTGGCC

CTTTTTTGCTTACGCGGTCGAGTTGCGAAATTTAATTTCCCGGATAACCCTCCAAACATA

GCGGGTGCCAGGTCACTTTCTCCGGCTGAAATTCAAAAAGTTGCAGCAAAGTTTGCCAAT

TCGGAGCCTCAAAGAAGCCAATCGGATCAGTTGGAAACAAATCAATCTGTATCTGAATCT

CGAGCAGAATCTCCCTGCCCTTCAGTTGTATCCGAATCTCGAGCAGAATCTCCATGCACT

TCTGTTGTATCCGATGGGACTGTCCAGATGGAAAGTAGTGAATTGAGCTGGGATGGGCCG

TTTTTGGATATGTTAATGAATACAAATTCCAGTAAATCCCCATGCACTTCTGCTGTATCC

GATGGGACTGTCCAGTTGGAAAGTAGTGAATTGAGGTGGGATGGGCCGTTTTTGGATATG

TTAATGAATACAAATTCCAGTAATTACTCCGCCGAATATGGAATATTTCCGGGTTTCGAT

GATCTTCATAATGATTTTTTCCCATCATCATCAACTCCTAATCTCGATTATGGAGAGGAG

ACCAATTTTGATGGTGTTTTAGAACAGCATTCATTTCTTTGGAATTTCTAA

>EVM0043629

ATGTCAGCAGCTATAGATATCTACAGCGCACCAGTACCAGCTTTTTCAGATCCTTGTAGA

GAAGAACTCATGAAAGCACTTGAACCTTTTATGAAAAGTGCTTCGCCATCACCAACTTCT

ACCTCCTATTCTTCACCATCTCCTTCAACTTCTTCTCCTTTCTCTTCTTACCCTCCTTGC

TTTTACAATAATAACTCTCCCGTCTCTTCATATCCCAACTTGGACCTTAGCTTTTGCTCG

CCAACGAGCACCCATATGTTTTCTAATGGGTTCTTGGATTATAGCCAAATATATGAGCAA

ACAGGTCCAATTGGGCTGAACCACCTTACCCCTCCACAAATCCTCCAAATCCAAGCCAAA

ATCCACCTCCAACAACAGCAGCAGCAGCAGAAAATGGAAAGTGTTGCTGCCACACCACGG

TTTGTCCATAACCAAAGGGCTAGTAGCTTCTTAGCTCCAAAAGCTGTCCCTATGAAACAA

TCTGCTGCGTCTCCTCATAAGCCAACAAAGCTTTATAGAGGAGTCAGGCAGAGACATTGG

GGAAAATGGGTTGCTGAGATTAGACTTCCAAAGAACAGAACTAGACTCTGGCTTGGCACT

TTTGACACGGCTGAAGAGGCAGCTTTGGCTTATGACAAGGCTGCTTATAAGCTGAGAGGA

GAATTCGCTAGGCTTAACTTTCCACATCTTCGCCACCAGGGAGCTCACGTGTCTGGTGAA

TTTGGCGACTACAAGCCTCTCCATTCCTCTGTTGATGCCAAATTACAAGCAATTTGTCAA

AGCCTGGGTTTGCAAAAACAGGGGAAAACAAGGGAGCCCTGTTCTGTTGCTAATTCAAAA

AAGACTACACCGGCTCCTTTGCAAGCAAAGATCGAAGACGACAGTTCTTTGAAAGGTGCA

TTGAAAACTGAGTATGAGGATTTTGGAGTTGAGGACTATAAGGTGGAGATGCCATCACCA

TCACCAGCCTCATCTGACGAATCATTGGCTGGTTCTTCTTCACCAGAATCAGAGATTTCT

TTCTTGGATTTCTCTGATTCCTCACAGCGGGACGAGTTTGAAAATTTTGGTTTGGAGAAG

TACCCCTCAGTTGAGATTGACTGGTCATCCATCTAA

>EVM0044805

ATGGCAGCTACAATGGATTTCCACAGCTGTAGACCGCTTCAATCAGACCTCCTTGGTGGT

GAGTTAATGGAAGCACTTGAACCTTTTATGAAAAGTGATTCTTCTTCAACGACCTCATCT

CCTTCTCAAACCCCAAACTACCCTTCTCCCCCTTATACGTCATCCAATTACTCTTCTTTC

TCTCCACAAGCACTTCGGCAACGTCAACAGTCCTTTTTCAACCCAGACGACTGCTGCTCT

ACATCGACAACCTATCCATTTTCAACCGGGTTCTCGTTCGACGACCCAATGGGTCTCCAG

CAACCATCGGACTCAGTTGAGCTTGACCACTTTACATCAACCGAGACCCACCTGAATAAC

CTCTCGTATCTTCAAGCTTACCAACAACCCCATCCCCACAAATTTTTATCCCCAAAGCCG

ATCCCCATGAAACAAATAGGTGCACCACCAAAACCCACAAAACTTTACAGAGGAGTGAGA

CAAAGGCACTGGGGCAAGTGGGTCGCGGAGATTCGTTTGCCCAAAAACCGAACCCGACTC

TGGCTTGGCACATTCGACACAGCAGAGGAGGCAGCTTTGGCTTATGATAGAGCAGCTTAT

AAACTCAGAGGCGACTTTGCCAGACTTAACTTCCCAAATTTACTCCACCAAGGGTCCTAC

ATCGGCGATTACCAGCCTCTCCATTCCTCAGTGGATGCGAAACTTAAAGCTATCTGTGAA

AGCTTGGAGAACTCTTCGCAGCAGAAACAAGGAGGGAAAACAAAGAGGCAGAGTAACTCG

ACGAAGAAGAAAGCCAACTCGGCAGTGGTGACTAGGGAGGAGCAAGAGGTTAAGGCTGAG

ACAGAGTCTCCGCCATTGACTGAGAGTGCTGGGTCGGGTGGATCTTCGCCTTTGTCGGAT

CTGACGTTTCAGGATTGCGAGGAAGCACCGTTGGATTTTGAGTCGGGGAATTTTATGTTG

CAGAAGTATCCTTCTTATGAGATTGATTGGGCTTCAATACTATCGTAG

>EVM0033660

ATGGTGAAGAACGAGCTCAAGATCCAGTCAGAGACTTCCACGCCATCATCGGCATCTTCT

TCAGCATGCAAGAAGAAGAAATACAAGGGAGTTAGAATGAGAAGTTGGGGCTCATGGGTT

TCTGAGATAAGGGCACCAAATCAAAAGACAAGAATATGGTTAGGCTCTTATTCAACCGCT

GAAGCTGCTGCTAGAGCCTATGATGCTGCGCTTTTATGTCTCAAGGGCTCTTCAGCCAAT

CTCAACTTTCCTATTTCTTCCTCACATTACATTCCTGACGCTGTGATGTCCCCCAAGTCC

ATTCAGAGAGCCGCCGCCGCCGCTGCTAATACTTTCGCAGACAATCCCACCACCCCTCAA

GTATCGTCACCTCCTCTTCCTTCCTCCTCGTCGTCGTCATCGTCAATCCTATCATCTCCG

TCAATGGTTTCCTCGCCGTCCCATCAGCTTGATGATTATATGTCACTGATGGAATCGTTT

GGGACCGACAATGAACCGATTCCAATGCTAGATTCTTGGTACAGTTTTGATGGATTACAA

TATCCAAAGTATCTTGATCAAATGTTCGATGGAGCGTCATTCAATCCACCGATGATCGAT

GATTTCTACGAAACCGATATTCGTCTATGGAGCTTTGCTGAATAG

>EVM0057138

ATGGTGCAGTCAAAAAAGTTCAGGGGGGTGAGGCAGCGACATTGGGGTTCCTGGGTTTCT

GAGATTAGACATCCTTTACTGAAGAGGAGGGTATGGCTAGGCACATTTGAAACAGCAGAA

GAAGCAGCGAGAGCCTATGATCAGGCAGCGATCTTGATGAGCGGTCGAAATGCGAAAACT

AACTTTCCAATACCTCAAACTAATTCAAACGAGGAGGACCCAAAATCAAGTGATCAGCCC

TTACTCCCAGCACCACCAAATGGTTTGTCTGAGATCCTTCACGCTAAGCTAAGAAAGTGC

AGCAAGGCGCCATCACCATCGATGACTTGCTTGAGGCTTGACACTGAGAATTCACTTATC

GGGGTTTGGCAGAAGCGTGCAGGTGAGCGTTCCGACTCGAATTGGGTCATGAGAGTGCAG

CTTGGACAGAGAGAAAGCCAGGCTTCTGAGAGTACACCGCCTTTGCCTGTGTCGTCAGGA

GGGGTATCAAAGCCAGAATTGAGATCAGAAGTGGGTGAAGAAGAGAGAATTGCACTGCAA

ATGATTGAAGAGCTTCTCAACAGGAATTGTCCTAGCCCTTCTTTTGGGGCTCAAGATCAC

GGAGATGGTAGCCTCTTTCTTTAA

>EVM0045295

ATGGAAAATTGCAGAAGGTCTCCATTGAAGCCCTGGAAGAAAGGTCCAACAAGAGGCAAA

GGCGGTCCTCAAAATGCCATGTGTGAGTACCGTGGGGTTCGTCAAAGAACATGGGGCAAA

TGGGTGGCAGAAATCAGAGAGCCCAAGAAGAGAACCAGACTATGGTTGGGTTCTTTTGCC

ACTGCTGAAGAAGCTGCTATGGCCTATGATGAGGCTGCAAGAAGATTGTATGGACCGGAT

GCTTATCTTAATCTACCTCACCTTCAGTCTAATTATAATCCTCTAAACAAATCACAGAAG

CTCAAATGGATTCCTTCCAAGAACTTCATTTCCATGTTTCCTTCTTGTGGGCTGCTTAAT

ATACATGCACAGCCTAGCGTTCATGTCATCCATCAGCGGCTCCATGAACTCAAGAGTAAT

AGGGCCCTTCATCAATCCCCTGTCGCTTCAAGTTCTTCCTCCTCTGAATCCAGAAATGAA

GTGATGATTGTAAGTGAAGAAAACCATGTAGCAAACCTCTCCGTAGCAGAGAAAGATGTG

GAAATATCATCAGAGAAGATGCTGATAAGAAATCATGACGAGAAACCACAGATTGATCTG

AATGAGTTCCTTCAGCAGCTGGGCATACTGAAAGAAGAGAAGCAACCGGATAGCAGTGAT

GTAGAAGAATGTCTCACGCTGCCAGAATCTTCACAAAAAGATGAAAACGAATTGGCAGCT

TTGGCAGACAAGAGTTTCAATTGGGATTCACTGATTGAAATGCATGGAATTACAGATCAT

CAAGCAGCAGAATTGAATAGTTTCCCAGTTTATGACGCCCAAGACGAGGCGGCCTTCCCA

ACTTCCATTTGGAACTTCTAG

>EVM0000045

ATGTGCGTATTGAAGGTGGCGAACTCAAGAGGAGACTCAGGGGAGTACAGCAAGTACCCT

GCAACAGATTGCGATGATAACCAAGAAGAGAGATCATACACAGCACAGCAAATATTTTCC

GAATTGAATCAACCAAGACAGCGGCATGTAGAGGTACCTCAGCCGCCGCATATGTTAATG

GGGTATGGCAATTCAACAGAGATGTCTGCGATGGTATCAGCACTTACACATGTGGTGTCA

GGCCAAAGAGAGAGCACCAGTGATTGGGGATCATATGAACCTGCAGGTGTAGGAGGTGAA

ACAATAACGTCAGCTTTTGTTCAGGCAGAACCTGGTTCTAGTGCTACTCCCGTTTCTCCA

CCCTTGTCTGCTTATTCTTCAGCTTCTGGCTCTGGTTCCTGGATTGGCCAGAAGAGAGGG

AGAGAAAAAGAAACCGGTGCAGCAACTCCATTGAAGGAGTCTTTGCCAGGGGTTTATAGA

GGACTTAATGATTTTAGAAGTTCACAGGGCGATTCGTTGTCATCTGGTGCAACTGCAACT

GAAGAAGCGGCTGCTTCAACTTTAGTATTCCCCGCTACAGCCACACCCTCCTCCACAGCA

GCACCGTCAAGTGAAACAGCATCTCTGGGTGAAACTGGAGAGCGAAAAATAAGATACAGA

GGAGTCAGGCAAAGGCCATGGGGCAAATGGGCAGCAGAGATACGTGATCCACACAAAGCA

GCGAGAGTCTGGCTAGGCACATTTGAAACTGCAGAAGCTGCAGCTAGAGCCTATGATGAG

GCTGCTTTAAGATTCAGAGGAAACAGAGCTAAACTTAACTTCCCTGAAAATGCCAGGCTA

CCACCTGCTCAAATGCAGAATGTTACTGCTTCTCAAGCTCCCGTTTTCCGTTCACAATTA

CCTTCTGGCCACCAGTTTCAGTCAATCTCATCTCCAAGGCAGCTAGCACAGCGACCGCAG

GTGCCTGCACCTGCATTGTTTCAGTCTCAGCCTGATATTATAAGAGACTACTGGGAGTAC

TCCCAGTTGTTGCAGAGTTCTGGGGACTTTCTTGAACAACAACCACCACCTTCAAATTTT

TCACAGCAGATGTTTTATAGTCCCCAGCTGGCTTCTTTTCAATCACCAACTTTGTCCTCA

TCACCTTCGTCTACTTCAGGCTCTTCATTTGCAGCGGTATCTTCTGATTCGCTATCATCA

ACACTACCTCCCTCTGCCTCTTCGTTTCCTCTGCTTTTTGCTGGTCAACAGTTGGGTTAT

TTTCGGTCACCACAAAATCAGAATCCAGCTGCTAGTTCCGATTTTCCGGTGCCCCCATGG

ACAGATTCTAGTCCCCATCCTTCCTCCACCGGTTAA

>EVM0005349

ATGGTGTTAACCTCCCAGAGCAACGAGCTGCCGTTTAATGAGAATGACTCGCAAGATATG

GTCATCTATCAGATGATCAACGAGGCCGGAGCCCCAAATAGCAGCAGCACATATAATATT

CTTCCGAGAAGCCAGACCAACACTCCGATCATGCTCCAGCCAGCTCGGACAGTCATAGCA

AAGAAGCATTACAGGGGCGTGAGGCGTCGGCCATGGGGCAAATATGCTGCCGAAATTCGA

GACTCCACAAGACACGGGGCTCGGATATGGCTAGGCACGTTCGAAACGGCTGAGGCGGCT

GCCCTGGCTTATGATAGGGCTGCTTTTAACATGCGTGGCTCGAAGGCCCTCCTTAATTTT

CCAGCTGAAGTGCTGGCTGCTATATCTACTCAAAACCTTCAACCGATTTTGAGTTCGGCG

AGGTCACGTAAGAAAGCAATGGATTCACGTGGTAGCTCTAGCACGGTAACTCTTGCAGCA

TCACAATCCGAATCAGAGAGCAGCACGAGTGGGGAGTCACGCCATCGGGGGTCGGATATC

CTAGAGAGTTAA

>EVM0031305

ATGTTTGGAGAGAAGAGCTTGGAATCAGGCCTTGAATGCAGTATTAATACTGTTTCCAGT

ACCAGTAATGCTAACGCACCGACATATTACAAGAGCCTCAGCTTTAGTAATATTTATTCA

ACAGAAAGCTGGGGTGATTTGCCATTAAGAGAAGATGATTCTGAGGATATGATCGTTTAC

AATGCTCTCCGGGATGCTGTTAACTCGGGATGGCTTCCATCAGTATCGTCGCAATCTAAT

ATCACGACGACGGCAGAAGAGTTTCCACAAACAACTGTTCCATTGGAAGTGGCACCGCGC

GAGCCTGGAGCGCCACCGCTGAAGGAAAAGGGATTACAGTACAAGGGTGTGAGAAGGAGG

CCATGGGGAAAATATGCGGCGGAGATTAGCGACCCCAATAAGATTGGGGCAAGGATGTGG

CTTGGGACTTATGAGAAGCCCGAGGATGCTGCACTAGCTTACGATCGAGCTGCGTTCAAA

ATGCGTGGCTCGAAGGCTAAGCTTAATTTCCCTCATCTGATCGGATCCGACGATACAGTA

ACTTCTAAGAAGCGGCTTTCAGCAGAGCCTTCCATTTCAGAATATGACTTGCCACGTTGG

ACGAAGCAAATCAAGATCAGTTCGGACGAGTCGTTGGTAGCCAGTTATTATAGTTGA

>EVM0017641

ATGTACAGTCAAACCGCCTCACGTTCCGGTTTTGCTCTGTTAGAATCAATTCGTCATCAC

CTTCTCGAAGATGAACCGGTTGCAGAGATTAGCAATGGCATCAATGCTCGTCCGGTTTAT

TGCAAGAGCTTGAGCTTTAACAGGCTTTTCTCAATGGATAATTGGAGTGATATCCTATTA

CACATTGATAACTCCAGCCAGACCAAGGCCTATGACACTCGAGTACCAGAGTCCATTAAC

ACGCAATGGTCTCTGTGGAATCAACTTGATTCCATGAACACTGCATCTAACTATGAGGTT

GAAGTGAGCGTGGAGGATGCATCCAATTACTTGCCACCACCAAACGGGAGCAGTTATAAG

GGAGTGAGGAGAAGGCCAGGGGGAAAATATGCGGCCGAGATTAGGTATCCCATGAAAAAT

GGTGCACGACAATGGCTGGGGACTTATGAGACACCGGAGGATGCGGCTGTGGCTTATGAT

CTGGCCGCTTTTAAAATGCGCGGTGCAAAAGCTAAGCTTAACTTTCCACATCTGATTGGC

TTGAGCAATTACGAACCCGTTAGAGTGACTAACAAGCGTCCCTCGCCTGAGCCATCTTCC

TTGTCATCGCCGGATTCATTGTTGTCGGAGGTGGATGATGAATCTTCGAAATCCAAGCGG

GGAGCATGTAGATTTGACTCTTGA

>EVM0057308

ATGTCGCAAGGAGGGAGCAACAGCTTAGGGCCTGAAGATTTAGACTTCTTGGAGTCCATT

CAACAGTATCTTACTAGCCCACAAATGGAAAATAGTGAGGGTACTGCTTCAAGCTTCTGT

AGTGATCATGATATCCTCTATAATGTTTCAATGACCTCAACAACGTTAGCGGTCTTTGAT

TCGGATCCTCAAGGGCAGGGCATTCCTGCGGTGGAAGTGGCGAGTAAGACTAACGCGCTG

CCAAAAGGGAGTCAATATAGGGGGGTGAGGAGGAGGCATTGGGGGAAGTATGCGGCGGAG

ATTAGGGATCCGAAGAAGAATGGAGCAAGGATGTGGCTTGGAACTTACGAGACACCTGAG

GATGCTGCTCTTGCTTATGATCAAGCTGCTTTTAAGATTCGTGGAAGCAAAGCTAAGCTT

AATTTTCCTCATCTAATTGGTTCTACAGAATATGAACCTGTTAGATGTTTGGGATTTCAG

AAGCGTGAGGAGTTAGATTCTTACGGTTCTGGACCTCGATCGGGACCTTTGTAA

>EVM0007023

ATGGAAAAACATATCAACCTTGAGATCGATGCTCAAGACAGCTCCCTCACTTCGTCTTCC

ACCACCACCGCTTCCTCCTCCTCCTTTTCTACCACCATTTCATACACTGATCGAAGCTCA

AAAAAAGAAGAGAATTCTATTGCTGATCCAAAAGGATCGAGAAAATCTCAAGAATTGAAA

GGTGGCAATGAGGGTAGTAAAAGGCGAAAGACTACCGATAATGAGAATAATGGGAAGCAT

CCAACATACAGAGGAGTGAGAATGAGAAGCTGGGGCAAATGGGTGTGTGAGATCAGAGAG

CCAAGGAAGAAGTCAAGAATATGGCTTGGAACATACCCTACAGCCGAAATGGCTGCTCGA

GCTCATGATGTAGCCGCCTTAGCCATTAAAGGCGGCTCAGCTTACCTCAATTTCCCTGAA

TTGGTTGATGAACTGCCTCGTCCACTCAGCAAGTCTCCCAAGGACATTCAAGCTGCCGCT

GCCAAGGCAGCCGCAGCTTCGTTCCCCGAAACAAGACACTGTGAGGCAGAAGCTGATCTG

AACCGAGCCGAGCTTAACGTCTCGAATTTATCAGATAATTTGGCCATGGACAACACACAA

GAATCAAGCAGCTCTCCTTCAACTGATGTTGATGACAAGTTGTTTGACCTTCCTGATCTT

ATCTTCATCGACGGCGTTAATCACAGTGATGGATTTTGCTTTTACTCTCCACCATGGCAA

TTATGTTCAGCTGATACTGGCTACCGACTTGAAGAGCCATTTTTATGGGAGTACTAG

>EVM0000144

ATGAATTCTAACAACTGGCTCTCGTTTCCTCTTTCTCCCACTCATCCTTCATTGCCTGCT

CATCTACATGCATCTCACCCTCATCAATTCTCTCTAGGGTTAGTCAATGATAGTATGGAA

AACCCATTTCAAACTCAAGAGTGGAGTCTTCTTAACACTCATCAAGGCAACAATGAAGTG

CCAAAGGTTGCAGACTTTCTTGGTGTTAGCAAATCTGAGAGTCCGTCAGATCTTGTAGCC

TTCAATGAAATTCAAGCTAATGATTCTGACTATCTCTTTTCAAGCAATAGTCTAGTACCA

GTCCAAAATGCAGTTGTAGGCGCAAATAACACCTTTGAGTTTCAAGAAAATGCCAGCAAT

TTGCAGTCATTAACACTCTCTATGGGTAGTGCTAGTTGTAAAGGTTCGACATGTGAACCC

AGCGGTGATAGTAGCACTAATACTGTTGAAGCTGCTGCACCAAGAAGAACTTTGGATACA

TTTGGGCAAAGAACATCCATATATCGTGGTGTAACAAGGCATCGATGGACAGGAAGGTAT

GAAGCTCATTTATGGGATAATAGTTGCAGAAGAGAAGGTCAATCTAGGAAAGGAAGACAG

GTCTATTTAGGTGGCTATGACAAAGAAGAAAAGGCAGCTAGGGCTTATGATCTTGCTGCT

CTTAAGTACTGGGGAACATCCACCACTACCAATTTTCCAATCAGCAACTATGAGAAAGAA

ATAGAGGAAATGAAGCACATGACCAGGCAAGAATTTGTAGCTTCCATTAGAAGGAAGAGT

AGTGGCTTTTCTAGGGGTGCATCCATGTATCGTGGAGTTACAAGGCATCACCAGCATGGT

AGATGGCAAGCAAGGATAGGCAGAGTTGCAGGAAACAAAGATCTCTACTTGGGAACATTT

AGCACTGAGGAGGAGGCTGCAGAAGCTTATGACATAGCAGCAATAAAGTTTAGAGGGCTT

AATGCAGTGACTAATTTTGACATGAATCGATATGATGTGAAGAGCATTCTTGAAAGCAAT

ACTTTGCCGATTGGAGGAGGGGCAGCCAAACGGCTAAAGGAGGCTCAAGCAATTGAATCA

TCACGAAAAAGAGAAGAAATGATTGCTCTTGGATCAAGTTTTCCATATGGATCAAGCTCA

AGCTCTAGCAGGCTACAAGCTTACTCTCTAATGCAGACACCATTTGAGCAACCTCAACCC

TTACTTACTTTACAGAATCAAGACATTCCTCAGTACACTCAAGATTCCTCACTTCACCAA

AACTTTCTTCAAACTCAGCTTCATTTGCACCAGCAATCTACAGGATCTAATTTCCTTCAG

AACCAATCAAGCCAGAACCCTCAGTATTACAACAGTTATATCCAAAACAATCCAGCTTTA

CTCTATGGATTGTGGAACATGGGCTCTTCATCATCTGTAATGGAGAATAATGGCAATTCT

AGTGGGAGCTATAGTACTGGAGGCTATGTGGGAAATGGGCTGGGAATTGCTTCCAATTCA

GCAGGGTCCAATGCAGTAGGATCAGCTGAGGAACTTGCACTTGTCAAAGTTGATTATGAT

ATGCCTTCTAGTGGCTATGGTAGCTGGTCTGGGGACTCGGTTCAGGGATCCAATCCAGGT

GTTTTCACAATGTGGAATGAGTGA

>EVM0056353

ATGGAGGGAAGAATCAGGGAAGACCATCTTGGGGTTAGTCCGCCGAGATACAGAGGTATA

CGACGGCGGAAATGGGGGAAGTGGGTGTCCGAAATCCGGGAGCCTGGTAAGAAGACAAGA

ATCTGGCTAGGGAGTTATGAGATGCCTGAAATGGCGGCGGCCGCTTACGATGTGGCAGCA

TTGCACCTTAGAGGACGCGGGGCGCTGCTAAATTTTCCTGAAATGGTGGATTGTTTGCCC

CAGCCGGCGAGCTCTAGCGCGGAGGATGTGCAAATGGCGGCACAAGAAGCGGCTTCGCTG

TTTCGGAGACCAGTAGGATGTTCGGAGGCTGTAAGCGGTGATTCTGGTGGCGGCGGAAGT

GGTGGTGGTGGTGGTATAGGTCCGGTTAGGGTTGGGTTGTCACCAAGCCAAATTCAAGCT

ATAAACGAGGCACCATTGGACTCACCAAAAATGTGGATGGAGTTAGACGGGGCTCTTCTG

TTGGAGCAGCCTGTGATTATGGGCGATGATATTTGTGTTGAGTATAGTGAGGAATGGGGA

GAAATGCAGCATGATTCCATTTGGGATTATTAA

>EVM0032428

ATGGAGGAGAGAAACGGTGGTAGCCATGGCGGTGTGAGCTCGAGCTATAGAGGGGTGAGA

AAGAGAAAATGGGGGAAATGGGTGTCAGAGATTCGTGAGCCGGGGAAAAAAAATAGAATT

TGGTTAGGGAGTTTTGAGACCCCGGAAATGGCAGCAGCAGCTTATGATGTTGCTGCTCTA

CATTTTAGGGGAAACGACACAAAACTTAATTTTCCTGACCTGGTTCATAATCTACCGAAG

CCAGCGAGCTCTAGCTCCGAAGATATACGCATGGCAGCCCATGAGGCAGCTATGAGCCTT

AGGCCACTCACGGCAGAGCCCTCCCGTCGTGGCAGCTCCGCCTCCGACGCAGGTCCCATC

ACTGTTAGGCTCTCTGCAAGCCAAATTCAGGCCATCAATGAGTCACCGTTGGATTCACCG

AAGACATGGATGCAAATGTCAGAGATAGCAATGCTGGAGTCGTCCATGGTGTTCTCCAAT

GGCATCGACGAGGATCAGTGGGATAACAAGCAAACATATTCTCTCTGGGATCCTTAG

>EVM0001082

ATGACCGGTAGCAGCAGCGGCAGCAGAATGGATCGAGGAAGAGGAGCAAGTGCAGGTCAG

AAGAAATTCAAGGGAGCAAGGAGGCGGAAATGGGGGAAGTGGGTTTCCGAGATAAGGATT

CCGGGGAAGCAACAACGGCTCTGGTTAGGTTCTTATTCTACACCCGAGGCGGCTGCGGTG

GCTCATGACATAGCTTCTTATTGTTTACGTGGACCTTCTTCGTTAGAGAGTCTGAATTTC

CCTCTAATGCTGCCTGCAAGTGTAAGGGAAGAGATGTCACCCAAGTCCATACAGAAAGCT

GCGTCAGATGCCGGACTGGCAATCGATGCTCAGATGATACTGAGCCGGTCGCCTGAAAAT

GAGGTCAGGATTGGTGCAGAAAATGTTGCCATTAATCAAGGGCTAGAGACAGAGTTATGG

GAGCCCGCAGGTGGTGATAGCAGTAATCATCGTGAGAATTGGCGTGGAAACAATATCGGT

GCGAGAGAAGGGGAGAGTTTAAACATCTCCATCGAAGATTATATGCTGTAG

>EVM0006666

ATGTCTATAAATCATGAACCTCCTGGATTGAAAACAAACCCACACGCGTCCGATCCAGCA

TCAAAACGGAGCTCCAAGAAGAGGACCGGCCAGCCTGACGCCCCATTTCATGGCGTAAGA

AAACGGAGTTGGGGCCGTTATGTATCTGAAATCCGGTTACCCGGAAAGAAGACCCGAGTA

TGGCTAGGATCATTTGGGTCACCCGAGATGGCTGCCCGGGCGCATGACTCGGCCGCTTTC

TTTTTAAGAGGCAAGTCAGCGAGTCTCAACTACCCTGACTCGGTCGGGTCTTTTCCCCGC

CCCGAGTCTTGCTCTAGCAAAGACATCCAATTGGCTGCGGCGAAAGCTGCAACTGGGTTT

GAGAAAGCGAGGGTAGGTAACCGGATCGGGATTGAGGATAGCGAGTCAGGTGGAGAACCC

GATAGTTCAATTTGGCTGGGTTTGGGTGAGATAGAAACGGTGTCGTACGAAGAAGTGAAA

CGAACTCCTTTGTTTTGTCCTTTGAGATTCGATTCAGTGAGCGGAAATGACGAGATGTTG

TATATGAACGATCAGGGAGCTTTTATTTGCTAA

>EVM0023183

ATGGAGGATCAGTTTCCTAGATTGGAAACCCTTATGCACAAGGAATTGGCAGCTTATAGT

AGCTTTCATGGAATGGCAACAGGATCAAGATATTTTGTTGATCCCACAATTTGGGGTGCT

CTAGCTGGGGCTACTTGTCATGTAGATGATGGAAATAATGGCACGAAATCGCCATCACAA

TCGTCTCCCGAAAGATTGTTTTCGAGTTCGGAATCGAGTTCTTCGGCAAACAATTTGGTT

TCAGGTACCAATTTGGTTGGTAACATCCCTGGATTTGGTGAACAGGATGTTTCCAAACAA

AATCATGAAAGTTGTTTTATGTCAGGATTGTATTCTATCAATTCTCTTGGAGGTTCAATA

TCTGACCATATTGTCAGAGAAAGCTCCATTAATCCTGTGAATCTCCTGGAATTGTTTCCA

GCACTGCATAACCAAGCTCAAGTATCCGAACCTCCCTCTCCATATTCCTCATCAAACACA

TCGAAATTCCCAAATTTGACCTTGATTTTGCAGGAACCAGCAGCTTTCATCCAAACAAGT

TGTCAAACAACTTGGACAGGATCAAAACCCAAACAAGCTGTCCATCAACTTGGCAAGGAT

CAGAGATGTGAGCCTATGTCATTTTTTCCAAACACTTCATTCTCCATGCCTCAACTAGGC

CAAACTCATTGCCAACCAAGCAATGAATGGCTCAAAATAAATCAAGCTTTGGCAAACTAT

TCTACCAAAGGATTTAATGACTATTGGCTTAGAACTACGCCCACACAGCCGATGAAATAC

ACCAGTCGAAGAATATTGCAGAACCACCATCAAAAGCCTTCATTTTTATCAGCTTCAACA

TCACCGGGAAAGGTTTATAGAGGAGTGAGGCAAAGGCACTGGGGCAAATGGGTCGCAGAG

ATTAGATTACCGCGAAACCGGACGAGGGTTTGGCTAGGGACTTTCAACACAGCAGAAGAA

GCTGCAATTGCTTATGACACAGCAGCTTACATGTTACGAGGAGATCATGCGCATTTGAAT

TTCCCAGATCATAAGCTTCAACTCAAGGCTAATTCATTGAACGGAACCACAGCTGCCCTT

CTTGAAGCAAAGTTACGAGCAATATCACAAGGCAACACCATTTCTGGTCAAAAGAAGCAC

AACAATGATCCACCGCCACCATCACCAGATAAGATTCTACATGACATGAATTCTACAAAA

CCGAAAGGTTTGATCCAAAACACAACGAAAAAAGAGTTGAAATGTGATTTGGAAGGCAAA

ACTGGTTGCCACGAACTGATTCATGAGAACCAGAAAGGTCAAGAGGTCGTAGCATCTGAT

GTAGAAGCTGTTTTGTTAAGTAGAATCCCTTCCTTGGACATGGACATGATCTGGGATGCT

CTTTTAGTCTCTGATTCATGA

>EVM0009817

ATGAATAACAAAAATTTACTCCCCGTTCCGTTCTTCCCGAAACCCGAAACATATCATTTA

TCGGAAATGTTTAGTCTCCTAAATGAAAATTCATCATTTCGTGGTTCATTTTTACAGCCC

CAAAATTCATTCTCTTCACCTCTTACCTCTAGTCACTACGACCCCACAAGTTACTCTCCT

TTTTCGACTGATATTTCGGCAATTGTTTCACTCCCCGGTATTATCGAGAAACCACCCGAG

ACACCAATGGTTCTTGATGGAATTGGTGCTGTTGTTGGCCAAGAAGTCCTTTTTGGCACA

AACAAAACCAAGGATTCAGAAACATCGGGTTTTGATTCGATTTCTCCACGAATTGGGTCT

AAAGAAAGAAGACAAGAAAATGGGGCTAAGAGAAACTGTGTTCATAATGGGGTTCCAGTG

CAAAGGACCTATAGAGGGGTGAGAAAGAGGCCATGGGGGAGGTGGTCAGCTGAGATACGA

GACCGTATAGGGCGGTGCCGCCACTGGCTTGGGACATTCGACACCGCGGAGGAGGCAGCG

CGTGCATATGACGCTGCTGCTAGAAGACTTAGAGGTGCAAAAGCAAGAACTAACTTTGAA

ATTCCTCCTGTCTTGCCTACTATACCTTCTTCTCTTTCTCCCCCAAGTGCATGTTCAGGT

TCAAGTCTAAACAATGCAGAGGTGAAGAGAAGGACTAGCAATTCTAAAAGCAACAAAATG

ATTGGTAATGGTGGTAGAAAGTGCGCAGTCGTTAGTTCTGTGGCACATTTGTTTAGTAAT

TTTGAAGGGAAAGGAACCAGTGGTGGCAATGTGGAGCTTGATTTAAAGTTGGCGACAGGA

TTTGGTGGTTATCATGATGGCAACAAGAGCGCTGCTGGTGCTCCTCCTCCATCTATGGTG

GTTTAG

>EVM0024412

ATGCATGAACCTTTCTCCCTATATATGCCCCAGCATACCATCCTCTCGAAAAACTTGAAA

ATTCAAAATCATACACACTGCTTCATCCTTATACCAAAATCTATACGAGTAAGAATGCAC

GGGCAGAACACGCTTGATCAGTCCGATTATGCCTTTCTTGAGTCCGTTCGCTTGCATTTG

CTTGGCGAAACTATTTCTTCTGCAGCTACCACCAGCTGCTTTAATCCGGATACTACTCAC

AGTGCAGTCTCAACAACAACCACCCCTGCTACCTTCACTCCAGCCCCTGTTTACTGCAGG

AGCACAAGCTTCAGCAGCTTGTACCCTTGCTTAACAGAAAACTGGGGAGACTTGCCACTC

AAAGTCGATGATTCCGAGGATATGTTCCTTTACGGTGTCCTCCGCGACGCCGTCACCGTT

GGTTGGGTCCCGTCACTCATGACCGATCAATTCATTGAGCACGGTACTCCAGCGGTGAAG

CTAGAACCCTCTGAGAATTTGACGATATCCCCTCCGCGCACGGAGGCTCCGATAGCTGAG

CCGGCGGTTGTTCCGTCCAAGGGGAAGCATTACCGGGGTGTGAGGCAGCGGCCGTGGGGG

AAATTCGCGGCGGAGATTAGGGACCCGGCGAAGAATGGGGCCCGGGTTTGGCTGGGGACA

TTCGAGACGGCGGAGGATGCAGCGTTGGCTTACGACCGCGCTGCCTATAGAATGCGTGGG

TCGAGGGCGTTGTTGAATTTTCCGTTGAGGGTGAATTCCGGTGAACCTGATCCGGTTAGA

GTGACTTCGAAGAGGTCGTCGCCGGAGCCATCATCGTCTGTGGAGAGTGCGTCCCCGAAG

AGGAGGAAGAAGGCTGGTGGCGCGGCGGGTAATGCTACGGTGATTGCTCAATCTGGGATG

CAAATGACGAATGGGGTTGGGTATCAAGTGGGCACACATGGCGAACAATTATTGGTTGCT

AGATAA

>EVM0015672

ATGGAAACATCACTAAATATACAATGTGATAATCAAACTACCACCTTTTCCTCCTCCTCC

TCTTCCTCTTCTTCAATCACCTCTTCCATCCCTTCTCTATTAATGAAGAATCCCAGAAAA

CAAGACCAGTCTGATCAAGACCCGAAAAACAATAATGAAAGTAACAAAAAGGCAAAGAGT

GGTGGAATTGCTGGAAGGCACCCATCCTACCGTGGAGTTAGAATGCGTCAATGGGGCAAA

TGGGTGTCCGAAATCCGGCAACCAAAGAAGAAGTCGAGAATCTGGCTGGGTACTTTCCCA

ACAGCAGAAATGGCGGCTCGAGCTCACGATGTAGCTGCCCTTACAATTAAAGGTCACTCA

GCTCACCTAAATTTCCCTGATCTCGCTCATGAGTTTCCACATCCAGCTAGTTCATCTCCC

AAAGATATTCAAGCTGCCGCTGCCCTAGCAGCTTCCTTGAGCTGCAAGACAAGTCAAAAG

GGCCGTGAGACCGAAGCTGAAGAGGAGCTGATGCTTCCTCGCTCCCCGGATAGTGCTTTG

GCATCTAATGAGACACAGGAGTATTCATTAAGCTCGCCATTAAGAGATGATGATGGCGAC

GCATTTATCGATTTGCCGGATATTTTGCAAGACACGGGCCATCAATTTGATGAATTTTGT

TACAAGCCATCACGGCAGCTGGTTGGAGCCGAGACTTCTGATATCGGGTTCTGGCTTCAC

GAGGAGCCTTCTTTATGGGAATACCACTCAAGTAGAAACATCTATTGA

>EVM0013811

ATGTCAACAGAAGTATCATCAGCCCTGGAGTTCATCAAGAACCACTTGTTTGGCGATCTT

CTTTCACCTCTCGCTACCTCCTCCTCCTCCTCCTCCCTCCTCCTCAGTTTCTTTTTATCA

ACTACCAGCTGCCGCTCACAGACTTCGACTTCTGCCTCTTCCACTGCATTTCCTGGTTTT

TTCGATTCTCCTGACCCTAGTTTCTTCGAATTCACTTCCGTTTTCAGTCCTGCTCAACAT

AACAAGACCAATATCTTCGAGTTTGAAGCTAAACCTGAAATTATTGATCTCTCAACGCCA

AAGCCCCTTGATTCAACCTCCCACCTCAATTTGCAACCAAGCAGCAGCAGTAACTCGTTC

GAACTTGTGAAACCACAAATTATTTCTCAACCAAGCAACAATTTCTTCCACGAAGAATCA

AGGCCCGGAGTTCAACCAGCTCGGAAACCGTCATTGAAAATATCGCTGCCCAGCAAAAAA

TCCGAATGGATTCAGTTTTCTAACCAGGACCCGCAACCCTTGGATGATAAGTCTGGTGTT

GCCGTAGAAGCAAAAAAGCATTGCAGAGGAGTCCGACAAAGGCCATGGGGCAAGTACGCA

GCTGAGATACGGGACCCGAACCGGAGAGGTGCACGTGTGTGGCTAGGAACCTTCGATACA

GCTCTCGAAGCTGCCAGAGCCTATGATCGAGCTGCCTTCAAGCTGCGCGGGTCGAAAGCG

ATTCTCAATTTTCCACTAGAAGCTGGGAAGTGTGATGTACAGGCCAACGAAGAAGGGGAA

AGGAAAAGACTTAGAGAGTGTGACGCAGAAGAGGTTGGGGATGTTAAGAGGGTTATGCGA

GTGGCAAAGAGAGAGGAACCCGAGAGGGATGTTCCGCTGACGCCGTCATGCTGGACAGCT

GTATGGGATCTTTGTGGCGATTCAAATGGTGTCTTTAACGTGCCTCCGTTGTCTCCGTTA

TCTCCTCACCCGCCGCTGGGATTTTCACAGCTAATGGTTATCTGA

>EVM0004062

ATGGCCTCCACGAATAAGTGGTTAGGTTTCTCTCTATCCCCTCAAGAACTTCCATCATCA

CAGTCTGATCATCAAGATCACTCTCAAAACAAAGACTCTCGCCTTCGTTTCCACTCAGAT

GGAATCTCTGGTGCAGATCATGTCTCTGGGGAGAGCTTTGATCTTACTTCTGACTCCACC

GCTCCTTCTCTCAACCTGCCTGCCTCTTTTGGTATTCTTGAAGCCTATAGAAACAATAAA

TCTCAAGATTGGAATAATATGAAGCGTTTAGGCATGAATGCAGACACAAACTACAGTACC

ACCTCAGACGTCCCGATATTCTTGGGTTCTTCATGCAATAGCCAGAACATTGATCAAAAT

CAAGAACCTAAGCTCGAGAACTTCCTTGGTGGCCATTCTTTCGGAAATCATGAACACAAA

CTTAATGTCTGCAGCACCATGTATGATACCAGTGGAGACTATATGTTTCAAAACTGTTCC

TTGCAACTTCCATCTAAGGATGCATCAAATGAAAGAACAAGCATTAATGGAGGAGGTGAT

ACTATCAACAATAATAATACCAATAGTTCCATTGGTTTATCCATGATTAAGACTTGGTTA

AGGAACCAGCCAGCACCGACACAACAAGACACAAGTAATAAGAGCAATGGCGGTGCACAA

AGCTTGTCCCTTTCAATGAGCACCGGGTCACAATCAGGTTCCGCTTTGCCACTTCTAGCA

GTAAATGGAGGAGGAAATAGAACTGGAGGAGAGCAGAGTTTTTCTGATAATAACAAACAA

CAAATGACCACACCGACTCTTGGTGACCAAACCGGTGCCATCGAAACGGTACCAAGGAAA

TCTATTGATACTTTCGGCCAAAGAACTTCTATATACCGTGGTGTAACAAGACATAGATGG

ACTGGTAGATATGAAGCTCATCTATGGGACAATAGCTGTAGAAGAGAAGGGCAGACTCGC

AAGGGAAGACAAGTTTATTTGGGTGGTTATGACAAAGAAGACAAGGCAGCTAGAGCTTAT

GATTTAGCAGCTTTGAAATACTGGGGCACTACCACCACGACAAATTTTCCGATTAGCAAC

TACGAGAAAGAGATAGAAGAAATGAAGCACATGACTAGGCAGGAGTATGTTGCGTCTCTT

CGAAGGAAAAGTAGTGGGTTTTCTCGGGGTGCTTCCATTTACCGAGGTGTAACTAGACAT

CATCAACATGGAAGATGGCAAGCAAGGATCGGAAGAGTTGCAGGGAACAAAGACCTTTAC

TTGGGAACTTTCAGCACCCAAGAAGAAGCAGCAGAGGCCTATGACATTGCAGCCATAAAA

TTCCGTGGACTGAATGCAGTAACCAACTTCGACATGAACAGATATGATGTTAACAGCATA

ATTGAGAGCAGCACATTGCCTGTTGGGGGTGCGGCTAAGCAGTTGAAAGAGGTGGAGCAT

GCTGAAATAACGACGGATGTGCAAAGAATAGATGATCGTGACAGCTCCAGCTCACAACTC

ATCGATGGGACTGGCAACTATAGTGCTGCAGCACACCATGGCTGGCCTACTATTGCGTTT

CAACAAGCTCAGGATTTTAGCATGCAGTATCCCTATGGCCAGAGGCTTTGGTGCAAGCAA

GAACAGGACTCTGACAATCATAGCTTTCAAGAGATTCATCAATTACAACTGGGAAACTCC

CAAAATTTCTTCCAGCCCTCGGCTCTGCATAACGTCATGAGCATGGACTCGTCTTCAATG

GAACACAGCTCTTGTTCTGATTCTGTCATGTATAGCGGCGGAGGTCACGATGGTACCAGT

ACTGGAACCAACGGAAGCTATCAGGGAATTGGTTATGGAAGCAATACTGGGTATGCTGTC

CCAATGGCTACAGTTATAGCTAATGATGGAAACACCCAAGATCAAGGGAATGGTTATGGA

GATGGAGAGGGGAAGGCTCTTGGCTATGAAAATATGTTTTCCTCGTCGGATCCGTATCAT

GCTAGAAATTTGTACTATCTTTCCCAGCAATCATCTTCCGGTGTGATCAAGGCTAGTGCA

TATGATCAGGGTTCAGCATGTAACAACTGGGTGCCAACAGCAGTTCCTACCATTGCAGCA

AGGTCTAACAATATGGCTGTTTGCCATGGAGGTCCACCACCTTTCACAGTATGGAATGAG

AGTACATAA

>EVM0051961

ATGGATAGGAAGAGAAAGGTCAGTGAAAGTGCTGAGGACAAGAGCTCTGGTGAAGGAACC

ATGGCTTGGGATGAGATGGTCAAGGAAGCTGCTGCAGCAGTAGCACTAGGAGGAGCTAGG

AGAGCTAGAAAGAGATTTGTTGGTGTTCGACAAAGGCCATCGGGTAGATGGGTGGCTGAG

ATTAAGGACACCATACAAAAAATAAGGGTGTGGTTAGGAACCTTTGATACTGCTGAGGAA

GCAGCGAGAGCCTATGATGAAGCTGCTTGCTTGCTTCGTGGTGCTAATACAAGAACAAAC

TTTTGGCCTTGTTCTCCCACTTCTTCAACAACTCCAGCTCTTCCCTCGAAGATTACTAAC

CTTCTACTCCAAAGGCTTAAGGCAAGAAACAACTCTTGTCATCCTTCTACAACCACTCAG

CCCCACAACCAGCAGCAAAAACTAGTGGAGGAATATAGAGAGCCAGCATCCGATTTCTCA

GACACCCAACTCACTGATTTCCTCAACCATACAGACGATTACTCCATGCGCAACGATAAC

ACCAGTAACACTATTGCTAGTGCATTTGATTGCACGACAACAAGTCTTGTGCCATGCTTA

ACAGAGAAAGAAGATAGCTTAGGAAAAGAATGGGACTTCGACTATAGCTGGAGTGATGTG

GCACAGTCATCCAGTGGTGATGGTAACAACTTGGGAGGTGAAGGAGAAGAAGATGGAGAG

GGAGAGGAAGAAGAGAACGACATAGGTGCTTTGGATTTTCATTTCGCTCATGATGTTGGA

TCTCCTTTTTACTACTCTCCTTTTGAGATTGCTGAGGAGATAGAGGAGCCAGTGGAGCCT

GAGTGCTATGGAGATGAGCCTTCTATGCTTAAAGCAGCCATGAAGAGAATGAAATTTGAG

AGGAAGTTCTCAGCTTCTCTCTATGCTTTTAATGGGATTCCTGAATGCTTAAAACTGAAG

CATGGATCAGGAAATGCCAAGGGAAAAGGACGTTCTGATCAATTAACCAAACTTCGAAAT

GCATGTAACCGTAAAAAGGATGAGAATAGAGCAGAAGAAGAAAGCTTGGAAGTGATGCAG

AAACAGGATGATTACTCGCAAAGCTCAATAGAAATGAAATCTTCTTCAGCTTCTATGATT

AATGATGGCGAATTGTCACTTTGGAACACTCTTGATCTACCGCCAATCTGCTTCATAAAC

TGA

>EVM0055116

ATGGTGAAAAGCGAGCTCAAAATCCAGTCAGAGGTTTCAAAACCGATGGCAATATTATCG

TCAACGCCTTCACCATCAGCATGCAGGAAGAAGAAATACAAGGGAGTTAGAATGAGAAGT

TGGGGCTCATGGGTGTCAGAGATTAGGGCACCAAATCAAAAAACAAGAATATGGTTAGGC

TCTTATTCAACTCCTGAAGCTGCTGCTAGAGCCTATGATGCTGCACTCTTTTGCCTCAAG

GGCTCCGCGGCCAATCTCAACTTTCCTATTACTTCCTTGCATTACATTCCTGACACTGTT

ATGTCCCCAAAGTGCATTCAAAGAGTGGCTGCAGCTGCCGCTAATGGTTTTGTGGACAAT

CCCGCCACCCCACAAGCATCATCGCCTCCTCTTCCTTGCTCCTCCTCCTCCTCCTCGTCA

ATCTCATCACCCCCATCAGTGGTCTCCTCGTCAATCTCATCACCCCCATCAGTGGTCTCC

TCGTCAATCTCATCACCCCCATCAGTGGTTTCCTCGCCGTCCGATCAGCTTGATGATTAT

ATGCCACTGTTGGAATCTTTTGAGACCAGCAATGAACCAATTTCAATGCTAGATTCTTGG

TACAGTTTTGATGGCTTACAATCTCCGAAGTACCTTGATCAAATGTTCAATGGGGTCTCA

TTCAATCCACCAATGATCGATGATCTCTACGAAGGGGATCTTCGTCTGTGGAGCTTTGCT

GAATAG

>EVM0037206

ATGGTGCAATCAAAAAAGTTCAGAGGGGTGAGGCAGCGACGGTGGGGTTCTTGGGTTTCT

GAAATTAGACATCCTTTATTGAGGGTATGGCTAGGAACTTTTGAAACAGCAGAAGAGGCA

GCAAGGGCCTACGATCAAGCGGCAGTCTTGATGAGTGGACGTGATGCGAAAACTAACTTT

CCCATGCCTCAAACTTCGAATGAAGATGACCCAAAATCAGGTGATCAGCCTCCACTCACA

GCACCACCAAATGGTTTGTCCCAGATCCTCCATGCCAAGATAAGAAAGTGCAGCAAGGCA

CCATCACCATCAATGACTTATTTGAGGCTCGACACTCAGAATTCCATTGGGGTTTGGCAA

AAGCGAGCTGGTCAGCGGTCCAACTCAAACTGGGTCATGACAGTGCAGCTCGGGACGAGA

GATAAGAGCCAAGTTTCTGAGAGTACATTGCCATTGCCTGATCAATCTTCAGGAGGGGTA

TCAGGACCAGAATGCAGAGCAGAAAGGGATGAAGAAGAGAGAGTTGCACTGCAAATGATT

GAAGAGCTTCTTTACAGGAGTTGTCCCAGCCCTACTTTTGGAGCTCAAGATCATGGAGAT

GGTAGCTTTTTGCTTTAG

>EVM0047653

ATGGGAGGAATGTCAAAATCAATCGTGAGTGGTTATGGAGAGAAAAAACAGTTCAGGAAG

CCTGCGCAGGCTAGTTCTCGGAAAGGTTGCATGAGGGGGAAAGGGGGGCCAGAGAATGCT

CTCTGCTCTTACAAGGGTGTCCGCCAGAGAACTTGGGGAAAATGGGTGGCTGAGATTCGA

GAGCCCAACCGTGGTGCTCGTCTCTGGCTTGGCACCTTTGACACCTCCCATGAAGCTGCC

ACGGCTTACGATGCTGCTGCTCGGAAACTTTATGGAACCGAGGCAAAGCTCAATTTACCA

GATCTCCAAGTTAATAATTGCCAGTTTCCTGCCTCTCCAGCCAATTCCCAGGTGACCCAA

ATGACAACCCAGCCTTCTCAAATCATCCATAACTATAGTTCTACAACATCTACATGCTCG

TCCAATACCCCAAGTATCGAGTCTAATGAAATGACGCCAGTGCTATACAACCACGATCCA

ATCATGTCTTCCTCTAACAAAGGCGTTGATTCGAATGGAATGGAGGCAGTAAATTTTGGG

CGAAGTGAAGTGGGCATCAACGAATTTTGGTCGAATTTCAACGTGAACATTCCTTTCGAT

GACTCGATTTGGGATGAGGCAGCAATGTCTATTAATTTTCCAGTGCTGGAAGATCCTGGG

ATTTTTGCAGGCAATCTCATGGAAGGAACAGGTAGGGACACATTGCAGACTCCGTGGTTC

ATGTAA

>EVM0052780

ATGTTGGATCTTAACTTGACTGCGGTTTCTGGTGATTCGTCAACGACAGTGAAGAGGAAT

AATAATAGTAACAAGATTCTAGAGCTTTCCAACTCTCCAATGGAAAGTTCCGGGAGCTTC

AATTCTTCCTCTATAGTCAATGCCGATGGCTGTGGTGATGAGGACTCTTGCTCCAACGGC

GAAGTTTTCGCTCTCAATTTCAGTATCTTGTCAAATGAAAGCAGCTCCAAGAAGACCGTT

TTTTCTAATAATAATGATGATCATCATAGTGACCTTGGAGATCGAACGATCCAGCTTTTT

CCAGTGGAGTGCGGGACCAAGAATGCAGGAGGAGGGTCTACCTCTTCTCCGGCGGTGCAG

ATGCAGCGGATGGATCTCGGTGGGTCCATGAACTATGGTGTTCCTCCGGAGCAGGGGATC

GGGGCTCAACAACAAAAGCCAGTGAAAAAGAGTCGGCGTGGGCCCAGGTCGAGGAGCTCG

CAGTATAGAGGAGTCACGTTTTATCGGAGAACTGGAAGATGGGAATCTCATATTTGGGAT

TGTGGAAAGCAAGTCTACTTGGGGGGATTTGATACTGCACATGCTGCAGCTAGGGCATAT

GATCGAGCTGCGATCAAGTTCCGTGGAGTTGATGCGGACATCAACTATAACGTTAGTGAT

TATGATGAGGATATTAAGCAGATGAGTAATTTCACAAAAGAAGAATTTGTGCATATTCTA

CGTCGTCAGAGCACTGGATTCTCTAGGGGCAGCTCAAAATACAGAGGAGTGACACTCCAC

AAGTGTGGCCGATGGGAGGCTCGCATGGGGCAGTTCCTGGGAAAGAAGGCTTATGACAAG

GCTGCTATTAAATGCAATGGAAGGGAAGCAGTTACCAACTTTGAGCCAAGCACATATGAA

GTGGAGATGCTTTCAGAGCCCAATAGTGGAGTTGCTTCTCGTACAGATGGCAACCAAAAT

CTTGACCTGAACTTGGGAATCGCCCCTCCAGATACTTCTGATAGCCTAAAGGTAAACAGC

AACGTGGGTGGCTTTTATTTCCACAGTGGCTGGGATGGCGTGTCAACTGATAGGGCACCA

AAGTTTCTGAATTCTGGTTCTGCAACAATGAAAAATCAACTACCGAATGGCCCTGGAGTT

ACATCTGTTTACCCTCCTCCTATGTGGGATGGTCTGAATCATCGAGTCTTTCCCATCTAT

GAGGAAAGAGCAATCGAGAAGAGAATGGAAGTAGATTCCTTTCCAAATTGGACCAGGCAA

ACCCAAGGCCCTTATGGTGGTGGAGCAAATGCACTGCCTGTCTTCTCTACTGCAGCATCA

TCAGGATTCGCTTCTTCAGCAATTATGGCACCTTCAGCAGCTGCTGGTCAACTTCACTTT

TCAAGCTCAACAACCCTCCACCATCGTAACTCTCCTTCAATGACCAACCCCAGCAACTTC

TCCCAGTTCTTCTACTGTAGGAGCTGA

>EVM0010374

ATGGCAGATCCCAATTCAAATGTTCCTCCAGCAGACCAGTTACCACCGCCTCAACCTTCA

AACCCATCTATACAAATCCCTGACCCTCCATCCTCTATTCTGCTCCAGGATGATCAATCA

CCTCATTCCACCTCCTCGAACCCGTCTCCTAGATTGATTCCTTCTCCGGGTGGATCTCTG

GGCCAGCCATTAACGGTGAGACATGATGAATATTATTCACCAAGGTTCGGGTTTCCGAGC

CAGCTTGTGAGTGGAAGGCAGGATGACCAATCACCAAGGTCCACCCGTTCCTCAGGCGGG

TCATCGACTCAGCCCACAAGCGGAAGACACCCGAGTTATAAAGGAATTAGGTTACGGAGT

GGGAAATGGGTATCTGAGATCCGGGAACCACGTAAAACTACGCGAGTATGGCTTGGCACG

TATCCAACTCCTGAAATGGCAGCCGCCGCTTATGATGTGGCGGCTCTAGCCCTTAAAGGA

CCTGATACACCACTAAATTTCCCGGAATCGATTCATTCCTACCCAATACTAGCCTCAGCA

TCCTCTAGTGATATAAGGGCCGCAGCTTCTAGTGCAGCATCAAGCAGGTCGCCGAGACCA

AATCCTGAATTGGATCGGCCCGAAACGGAAGGCACATCATCCAGTACTGCTGCTGGCATT

CAATCAGGACAGGAATTCATCGACGAGGAAGAACTTTTAAACCTTCCCAATTTGCTAGTT

GACATGGCAGGAGGAATGCTAGTAAGCCCTCCAAGAATAAACACCCCATCTTCTGATGAT

TCACCGGGGAATTCCGATGCTGAAAGCCTATGGTCCTACCAGTAA

>EVM0032370

ATGGATTGTTTCAATCACTATTCTGATCCAAGTCCACTTGGGGCGAGAAATTCTTGGTCA

GTTTTTAATGAGAATAATGGTATTGGCCAAGAACAGTGTTCATATTCTCCTGTTCTTTCG

GATAGTAGCACTGCCAGTACTGTTACTACAGGAGTTCAGCCAGCTCCGAGTCTTTCTGAC

GAGGAAGTAATGTTAGCTTCGAGGAATCCGAAGAAGAGGGCAGGAAGAAAGAAGTTCAGG

GAAACAAGGCATCCGGTGTTTAGAGGGGTGAGGAGGAGGAATTCAGGTAAGTGGGTCTGT

GAAGTTAGGGAGCCTAATAAGAAATCAAGAATTTGGTTAGGGACTTTCCCTATCGCTGAA

ATGGCTGCTAGGGCACATGATGTTGCTGCTTTGGCATTGAGGGGAAGGTCTGCTTGCTTG

AATTTTGCGGATTCTGCTTGGAGGTTGCCGGTTCCGGCTTCTAGTGAAGCTAAGGATATT

CAGAAGGCTGCAGCAGAGGCAGCGGAGGCTTTCCGGCCAGAGGGCTGTGTGGGAGGTGAG

TTGATGAGGAGGGGAGATGAGGGGAAGAAGGTGGCGGAGACTGCTGCAGAGGCGGAGGAG

GGGGTGTTTTATATGGATGATGAGGCTGTTTTTGGGATGCCAGGCTTGCTGGCTAATATG

GCTGAAGGGATGTTATTGCCACCACCTCATTGTGGTGGTGGAGGGGATGGTTGGGGTAAC

ACGGAAGATATTGATCCTTATATGTCACTATGGAGTTTTTCTATATAA

>EVM0016530

ATGGCTCCTAGAGAAAGACCTGACAACCACCACCTCAACAGCCCTAGATCGGAGATCCGT

TTTAGAGGCGTGAGAAAGAGGCCATGGGGACGGTACGCCGCTGAGATCAGAGACCCAGGC

AAGAAAACGAGGGCCGCCCGTGCATACGATGCGGCTGCTCGTGAATTCCGAGGAGCCAAA

GCCAAAACTAATTTCCCTGCCATTGGTGAGATTAACCCGAACCCCACGCGCAGTCCTAGC

CAAAGCAGCACTATCGAGTCCTCCCCTCCGACCCCACGCGCAGCTTCCCCGCCGCCACTC

GATCTCACTCTTAACATTTCCCACCGTAACCCCGACCGCCAACAGTTTCCCAACGGAGTC

AGGTTTCCAGGAGGCGCGTGGCTCCCATTCCCTGCTGTTGCGCGTCCCGTCTTTTTTTTG

ACGCGTTTGCTCATGCGAAGAATAATACTCCTAGCAGTAGTAGTATATGATTCGGATTCG

TCATCAGTCGTTGATTTTGATCGTCATCACGATAGCAAGGGATTGTCACTTGATCTTAAC

CTGGCCCCACCACCGGAAGTCGCATGA

>EVM0003611

ATGGATGGAAGCTGCATAGATGAGAGCACAACTAATAGTACTGATAATTCCATATCCATC

ACACCAACCTCCCTCACTCCTCCTTCTCCACCTCCAGCTCCCACAACAAAATCACCACCT

GAGTCACTTTGCCGTGTTGGGAGCGGCAATAGTGTGATTCTTGATTCAGAATCTGGTGTC

GAAGCTGAATCAAAAAAACTCCCTTCCTCTAAATACAAAGGAGTTGTCCCCCAACCAAAC

GGTCGCTGGGGTGCACAGATTTACGAGAAGCACCAGCGCGTGTGGCTTGGGACATTCAAT

GAAGAAGATGAAGCAGCAAGAGCCTATGATACTGCTGCGCAAAGGTTTCGTGGAAGGGAT

GCCGTGACGAACTTCAAACTAGTTAATGAAACTGAAGATGATGAGATCGAGGCTACTTTT

CTGACCACTCATTCAAAAGCTGAAATCGTGGACATGTTAAGGAAACATACGTACAACGAT

GAACTAGAGCAAAGCAAAAGGAACCGCAAGAGTAACAATGGCATAAATGGGAAGCAATAC

AAGAATACAGCAAGCTATGGGAGTAACAGGTATGACAATGATTGTGGTCAGGTGTTGAAA

GCGCGTGAGCAGCTTTTTGAGAAAGCTGTCACTCCAAGTGACGTTGGGAAATTAAATCGG

CTTGTTATACCAAAACAACATGCAGAAAAGCATTTTCCTTTGCAGAGTACATCAAGGTGT

AGCAGTAAAGGTGTGTTGCTTAACCTGGAAGATATGAGTGGTAAAGTGTGGAGGTTTCGT

TATTCTTATTGGAATAGTAGCCAGAGTTATGTTTTGACCAAAGGGTGGAGCCGGTTTGTA

AAAGAAAAGAACTTGAAAGCTGGTGACATTGTTTGCTTTCAAAGATCAACAGGACCAGAG

AAGCAGCTTTACATTGATTGGAAACCAAGAACCGGGTCCAACCAGGCCCAACCGATTCAG

ATGGTGAGGTTGTTTGGGGTCAACATTCTCAACGTTCCTGGGATGGAAAATGGATGTAAT

GGGAAGAGATCAGTGAGAGAAATGGAGCTTTTGTCTTTAAATCATCAATATAGCAAGAAA

CAAAGGATCATCGGAGCTTTGTAA

>EVM0042153

ATGGAGTCCAAAATGAGAAAAATCCGTATCATCTGTAATGATCCATATGCCACCGAAACC

GATTCGAGCGACGATGAATCTGATAGAAGATCTTTTAAAAAACGTTTCGTGCAAGAAGTC

AATCTTCCTCTTGCTGTTTCTCTCCAACCAAAAGCCCTTGAGCCTGAGAGTTCCTGTCAA

GACAGTAATAATAGTGCAAAACCCCCCAGCAAGAGGAGAAGGGTTTTGCCTAAAACCCCA

ACATCAACAACTGAAGAAAAGAAACTAGCACCAAAGAAGCCTGTTGGTGTTAGGCAAAGG

AAATGGGGTAAATGGGCAGCCGAGATTAGGAACCCAGTGACCAAAGTGAGGACATGGTTG

GGTACTTTCAATACTCTTGAAGAAGCTGCTCAGGCCTACGAGGCGAAGAAGCAGGAATAC

GAGGCTCTGGCTCTGGCTACAGCAGTCTACGAGAAAAGCCAAATCATGTCATCGGCCGCG

GGTGTCTTGCAATCTCACTCTCACAATAGCAGCAAAAATCAAGGCAGCTATGCTTCTGTT

GAAACTGATGATAGCGTTGTTTCACATACTTCCCCAGCTTCTGTTCTTGAACTTGAGCTT

GACACTCCTGCGGTGTCAAATGTCACTGGTGATTGTGGTCTTTTGATTAAAGAGGAGGAT

ATTTGTGCATCTGTTGCGGATCTTGATATACCTGATCTGGCTTTTATGACCGATACTCTG

GCGACTTGTCCGTTTGATCATGATCTTAACCTTGGACAGGAGTTTGGGAACCTTATTGAC

GAGTTCGGTCGATTCTATGATGATTACTGTGGCATTGTTGATCTTGATTTCTGCGGTCTA

AATGGTGATAAACCGAGCGATCTTCCTGATTATGATTTCGAGTTTGGCAACGAGGAGTTT

TCTTATTTGGATGATCATCAACAGAAACCCTTCAACATTGCATGCCTATAA

>EVM0056730

ATGAATTCTAACAACTGGCTTTCATTTCCTCTTTCTCCTACACATCCTACCTTGCCTGCC

CATCTACATGCATCTCACCCTCATCAATTCTCTCTAGGGTTAGTCAACGATAATATGGAG

AACCCATTTCAAACTCAAGAGTGGAGTCTTCTTAACACTCAAGGCAACAATGAGGTGCCA

AAGGTTGCAGACTTTCTTGGCGTGAGCAAATCTGAGAATCAATCAGATCTCATAGCCTTC

AATGAAATTCAAGCTAATAATTCTGAGTATCTCTTTTCAAGCAATAGTCTGTTTCCAGTC

CAAAATGCTGTGGTAGCCGCCAGTACTAACTATGAATTTCAAGAAAATTCTAGCAATTTG

CAGTCATTAACATTGTCTATGGGCAGTGCTAGTGGTAAGGGTTCTAAATGTGAAACCAGT

GGTGATAATAGTACAATTTCTGTCGAAGCTGCTGCTCCGAGAAGGACTTTGGATACATTT

GGCCAAAGAACATCCATCTATCGTGGTGTAACAAGGCATCGATGGACAGGAAGGTATGAA

GCTCATTTATGGGATAACAGTTGCAGAAGAGAAGGTCAATCCAGGAAAGGAAGGCAAGTC

TATTTAGGTGGATATGACAAAGAAGACAAGGCTGCTAGGGCTTATGACCTTGCTGCACTT

AAGTACTGGGGAACATCCACAACTACCAATTTTCCTATCAGCAACTACGAGAAAGAAGTA

GAGGACATGAAGAACATGACCAGACAAGAATTTGTGGCCTCCATTAGAAGAGTAGTGGCT

TCTCTAGGGGCATCCATGTATCGTGGAGTCACGAGGCATCACCAACATGGAAGATGGCAA

GCAAGAATTGGTAGGGTTGCTGGAAACAAAGATCTCTACTTGGGAACTTTTAGCACTGAG

GAGGAGGCTGCAGAAGCTTATGACATAGCAGCAATAAAGTTCAGAGGGCTTAATGCAGTG

ACTAACTTTGACATGAATCGATATGATGTGAAAAGCATTCTTGAGAGCAATAGCTTGCCA

ATTGGAGGAGGGGCAGCCAAACGGCTAAAGGAGGCTCAGGCAATCGAATCGTCACAAAAA

CGAGAAGAAATTGTTGCTCTTGGATCAAGTTATCCATATGGATCAAGTTCAAGCTCTAGT

AGACTACAAGCTTACTCTCTGATGCAGAAACCATTTGAGCAACCTCAACCTTTACTTACC

CTACAAAATCAAGACATTCCCGATCAGTACACTCAAATTCTTCATTTCAGGTCTAATTTC

CTGCATACTAACCAATCAAACGAAAACCCTCAGTATTACAACAGTTATATCCAAAACAAT

CCAGCTTTGCTTCATGGATTGTGGAACATGGGTTCATCTGTAATGGAGAATAATGGCAGT

TCTAGTGGGAGTTATAGCACTGGAGGTTATCTGGGAAATGGGCTGGGAATGGCTACCAAT

TCGACAGGGTCTAACGCAGTAGCTGAGGAACTTCCACTTGTTAAGGTTGATTATGATATG

CCTTCTGGTGGCTATGCAAGTTGGTCTGGGGAATCAGTTCAGGGATCCAATCCAGGTGTT

TTTACAATGTGGAATGAGTGA

>EVM0001221

ATGACAAGGAATACTGGCGATCAGATAAGCCCAGGAAGGCTTCGCTTGTGCATGATAGAA

GAAGAAAGGAGCGCTGGGGAGACTGGTAAGTGTATTAAAAGACGACGAAGAGACCCTTCT

GCGTTTGCATTGAGCTGCAATATTATTGATCAACAAAGTGACCAGCAGCAGCAGCCACAG

TCACTTGGTGATCGAACGGAAGCTGTTGCCACTACAGTCAAGAGAAGTTCAAGATTCCGG

GGAGTTAGCAGGTTTAATTTGATACAGCTAGCTAGGATCGCACGTTGGACGGGACGATTT

GAAGCTCACTTATGGGATAAAGCAACTTGGAATCCAACTCAGAGAAGGAAGGGTAAACAA

GGAGCTTATGATGAAGAAGAATCCGCTGCAAGAGCATATGATAATCATTTAGCTGCTCTC

AAATACTGGGGGACCTCAACTTTCACAAATTTCCCGGCGTCTGACTACGAGAAAGAAATT

GAAATAAAGGAAACGGTAACTAAAGAAGAACATCTAGCCTCCTTGAGAAGGAGAAGCAAT

GGGTTTTCAAGAGGTGTGTCCAAATACAGAGGAGTTGCTAGGCACCATCACAATGGGAGA

TGGGAAGAAAGGATAGGGAGAGTTTTTGGAAACAAGTATCTTTATCTTGGCACGTACAGC

ACTCAAGAGGAAACTGCTCGTGCGTATGATATAGCAGCCATTGAATACAGAGGGATCAAT

GCTATAACCAATTTCGATTTAAGTACATACATCAGATGGCTGAAGCCAGAAGCCAGTCTT

CCTGCACCTCAAGAATCGAAACCAACCTCAGCCCCCTGCCAATGA

>EVM0031724

ATGGATGTGATGGTTTCAGCTCTAGCTCAAGTAATTGGAAGCAGCCACAACAACTCAGCT

GATCAAGTGCATGAAAATCCAGTGACCTCGACACAATCAAGCACAGAAAATGATCAGACC

CAGCCAGTTGTTCAAGAGCAAGGGAATGCAAGGAGACGACATTACAGAGGAGTCCGGCAA

AGGCCGTGGGGCAAGTGGGCCGCCGAGATCCGGGATCCGAAGAAGGCATCTCGAGTGTGG

TTAGGCACTTTCGAAACGGCCGAGGCTGCTGCCCTTGCTTACGATGAAGCGGCTCTTAGG

TTCAAAGGAAGCAAGGCCAAGCTCAACTTTCCAGAACGGGTTCAAGCCGGCACGATCGAA

TTAGGCTTCTTTAGTTGCGGCCAAGGCTCGCACACTGTGGCTGAACCTGTCCCTGATCAC

ATTATGGCTCCTCTAGCTCGTTCTCGACGACCACAGGAAGCATATAATCCAAATAATTTT

CAATATCCACAATTTCTTGGAACTACAGCAGGCTATGGCTTCAGCCATGTCATGCCAGAA

GCTCCATTTGGTGGAGAAACGTTTCTTTCACCGACTTCCAGCTCCACCGCATCTTCTAAC

TCGTGGCCGATATCTTCTCAGCAGCAGCAACAACAACAACAGCAGCAAGAGGAACTATTG

CGATTATCAAATCAGTTTGGAAGTTCTTACAACTCTCGATATGATCCTTCTACATTCAAA

GATGAGGGGCTCTGA

>EVM0021255

ATGCAAGATTCTCAACAAGGAAAAACCTCAAGTCCTAACAGTGGCAGTGGCAGTAGCAGT

AGCAGAACTGCTTCTGTGACTGCTGGGGCTAGTGCACCTCATATTTCAGGTCCCCCACGT

GCTTTTCGTGGTGTTCGGCGTAGAAGTAGTGGAAAATGGGTGTCTGAGATTAGGGAGCCT

AAGAAGCCTAACAGAATCTGGCTGGGCACATTTCCTAACCCTGAAATGGCTGCTGTTGCT

TATGATGTGGCCGCGCTTGCGCTTAAGGGCCAAGATGCAGAGCTTAACTTCCCAAACTCG

GCTGCTTCTCTGCCTGTTCCTGCTTCCACTTCGCCACGTGATATTCAGGCGGCTGCAGCC

TCTGCAGCTGCTGCTATTGGAGCTGCAAAGGATGCTTTAGGGATTCCGAATACGGAGGAT

ACTAATAAAATGGAACAGGAAATTAGGTCAACGGTCGATGATCATTTTGTTGATGAGGAT

TTGATATTTGATATGCCTAATGTTCTTGTGAATATGGCTGAAGGGATGCTTCTTAGCCCT

CCTCGCTTGGACATTGCTGGTGATGAGACCACAACCACCTATGACAACACTGATGACCAG

AACCTTTGGAAATTCCCTTGA

>EVM0040570

ATGGCACCAGCTACTAACTGGCTTTCTTTCTCTCTCTCTCCAATGGAAATGTTGAGGTCA

TCTGAATCCCAGTTGATGTCTTACGAGAGCTCCTCTACTGCCTCTCCTCACTACTTGTTC

GATAACTACTATGCTGATGGCTGGGCAAACCCCAAAGAATCACAAGTCACGACCACAACA

ATGGCAGAAAGCTCTAATATTCTGACAAGCTTTATAGGCGCAGATACTCAACACCAGCAA

GTTCCAAAACTGGAGGATTTTTTTGGAGATTCTTCATCATCTATTAATGTTCGATACTCG

GATAGCCAGACAGAGACCCAAGACTCGTCCTTGACTCACATCTATCATGACCATCAGGGC

TCTGCAGCGGCTTACTTCAACGACCAACAAGATCTTAAAGCTATTGCTGGGTTCCAAGCC

TTTTCGACTAACTCAGGCTCAGAGGTTGATGACTCGGCTTCAATAGCTCCCTCAAGGCTG

GGTGGTATTGAGTCGACTGGGAACGGGTTGGGGTTCTCTAATAAAGCGGCCTTGTCACTT

GGGGTGAGTAATGATACAATCAATAATCAGGGTGGTTCTACAGAAAAGCTTGCTATTGTT

TCTGCTGATAACGATTGCTCTAATAAAAAGATTACTGATACCTTTGGCCAGCGAACTTCT

ATTTACAGAGGAGTTACCAGACATCGATGGACAGGCAGATATGAAGCGCATCTGTGGGAT

AACAGCTGTAGGAGAGAAGGTCAGGCCAGAAAAGGGCGTCAAGGTGGGTATGATAAGGAA

GAAAAGGCAGCTAGAGCTTATGATTTGGCAGCTCTAAAATACTGGGGTCCAGCGGCTACC

ACCAACTTCCCTGTTTCGAACTATTCTACAGAATTGGAGGGCATGAAGTATGTGTCCAAG

CAAGAATTCATTGCCTCACTGAGGAGGAAAAGTAGCGGTTTTTCACGGGGAGCCTCTATT

TACAGGGGTGTCACAAGGCATCATCAACAGGGTCGTTGGCAAGCGAGGATTGGTCGAGTA

GCTGGAAACAAAGATCTCTACCTTGGAACATTTGCCACAGAAGAGGAAGCAGCTGAGGCT

TATGATATAGCTGCAATAAAGTTTAGAGGCCTGAATGCAGTGACCAATTTTGAGATGAAC

CGATATGATGTTGAAACTATCATGAAGAGTGCTCTTCCAGTTGGTGGAGCAGCTAAGCGC

TTAAAGACATCGCTAAACGCCGAGCAGAAACCAAATGTGAACGACAACCATCTGCCGCCC

CATTTGCGGTGCAGCAGCTATAGCACCGCCAACAACATCAGCTTTGCTGCTATCCAGCCA

GGCTCTACTGCAGTCCCTTGCGGGGTTCCATTTGATGCTTCCACCGCGCTTTATCATCAC

AATCTTGTCCACCACCTCCAAACCACTAACAACTTCGCAGCAACTGACTCCTCCGGCTCC

TCCTCATCCATGGCAACTGCAATGACTACTTCGCCGCAAACAACAGAGTATTTTACATGG

CCCCATCAAACTTATTGA

>EVM0000771

ATGCAATATTATCAGCAAGGCAGCACCTCAAGTGCTGGCAGTAGCAGTGGAAGTAGCAGA

AATGCTTCTGTTGCGGCTACTGCTGGGGCTATTGCACCTAAGGTTTCAGGCCACCACCAT

GGTTTCCGGGGAGTTCGGCGTAGGAGCAGCGGAAAATGGGTGTCTGAGATTAGAGAGCCT

AGGAAGCCTAACAGAATCTGGTTAGGCACATTTCCTACCCCTGAAATGGCTGCTGTTGCT

TACGACGTGGCGGCGCTTGCACTTAAAGGCCAAGACGCAGAGCTTAATTTCCCGGACGCA

GCTGCTTCTCTGCCAGTTCCTGCTTCCACGTCGCCGCGTGATATTCAGGTTGCCGCAGCC

TCTGCGGCTGCTGCTATAGGTGCTGCAAAGGATGCTTTAGGGATTCCAAGCATGGGGGAT

ACTAATCAAATGGAACAGGAAATTAGGCCAATGGTCAATGATCAGTTTGTTGACGAGGAT

TTGATGTTTGATATGCCTAATGTTCTTGTGAACATGGCAGAAGGGATGCTTCTTAGCCCT

CCTCGCTTGGACATTGCAGGTGAAGATGCCACAGCCTATGACTACAGCACTGGAGACCAG

AACCTTTGGAAATTCCCTTGA

>EVM0020349

ATGGAAAGTGGAGTAGACAAGGATATGGCAACAAGAAAGAGAGGAGGGATAGAAAGTGAG

AGGCAATACAAGGGGATAAGGATGAGGAAGTGGGGAAAATGGGTGGCTGAGATAAGAGAG

CCTAACAAAAGGTCAAGGATTTGGCTTGGTTCCTACTCAACACCAGTAGCGGCGGCTCGT

GCTTATGACACCGCCGTTTTTTATTTGAGAGGGCCGTCGGCAAGGCTCAATTTTCCTGAA

TTTTTGGCTGGAGAGAATTTTGGCGGTGGTGGATCATGTGGGGACATGTCAGCTGCTTCT

ATAAGAAAAAGAGCAACTGAGGTTGGAGCTCATGTTGACGCTATTGAGACAGCGTTAAAC

CACCACCACCACGTCCATCACGATGACCGTCAAAGAAACAGTAATAATAGTAGCAGTAGT

AATGATAATAATGAAACTGCTGTTGACAGTAGAGAGTTGAAGCCACGGCCGGTTGACTTG

AATAAGGTGCCTGACCCAGAGGATTCCGATGGAGATGAGTGGAAGAGGAGTTAA

>EVM0010409

ATGTGGAACTTAAATGACTCCCCTGATCAAACAAGAGACGATGAATCCGAGGGATGCTCA

AGTCAAAAAACATCTATAGATGGCGAAGATGACAAGGGCAAAAGGGTCGGATCCGTGTCG

AATTCCAGCTCTTCTGCTGTGGTGATTGAAGATGGATCAGAGGAAGAGGATACTGTTGGA

GATAAAGGAAATAAAACAATCAAGAAACGTAGTATGAGCTCTAGCAGCAGCAAGATTTTC

GGGTTCTCGGTGCCTTATGATCAATACTCCGTGGACATGAGTGATCCACCAGTGACCCGG

CAGTTCTTTCCCTTAGAAGATCAAGAAATGGGGTCCACATCGGGCGGTGGAGGTAGTTTT

GGAGGTGGTGATGGAGTTGGAGGTGGATTTCCTCGGGCTCACTGGGTTGGTGTCAAGTTT

TGCCAATCGGAATCATCATTTGCTTCTCAAAAATCCATGGAAGTCTCACAACCATTGAAG

AAAAGTAGACGAGGACCACGGTCCAGGAGCTCTCAATACCGTGGTGTTACCTTCTACCGG

AGGACTGGCCGATGGGAGTCTCATATATGGGATTGTGGGAAGCAAGTGTATCTAGGTGGA

TTTGACACTGCACATGCAGCTGCTCGCGCATATGATAGGGCAGCTATCAAGTTTCGGGGA

GTGGAGGCGGATATTAATTTTAGAATTGAAGATTATGAGGAGGACTTGAAACAGATGAGC

AACCTTACAAAGGAAGAATTTGTTCATGTACTTCGCCGGCAAAGCACGGGATTTCCTAGA

GGTAGTTCCAAGTATAGAGGTGTAACCTTGCACAAGTGTGGAAGATGGGAAGCTCGAATG

GGCCAATTCTTAGGCAAAAAGTATGTTTATTTGGGCTTGTTTGATACTGAGATTGAAGCT

GCAAGGGCCTATGACAAAGCTGCCATCAAGTGCAATGGCAAGGAGGCAGTAACCAACTTT

GATCCCAGCATTTATGCAAATGAGCTTAACTCAATTGAATCATCAGGTAATGCTGCGGAT

CATAGTCTTGATTTGAGCTTGGGCAATCCAGCTTCGAAGCAAAACAGTGTAGAATTTGGT

GAGGATAGGCACGATGTTGCAATGGATCCGGCAACTATGCCATTTGAACCTAATTGGCAA

AATCAGGGGCTTAGACCCAAGCTTAACCTTCATAGAAGTGACAATGATGGACATGGAAGA

GATGGGTATGGAGAGACTGAGACGACTCAGCTTCTTAGCAAAATCCACCTTCAATCTCCA

GCATCTCTTAAGTCCAGTGAAATGCCAAGATATGAGCACCAGTTCAGATCACACGGAGAT

AGTCAGATGCATCCTTTTTTCTCTCCACAGTTTAACTCGCCAAACTATCAAATTCAGTAT

CCAAGCAGCAGCAATGGAGGCCGAATTGGAGGTGATCTCTCTCTCTCACCAGCTGAGCTT

CATTATCGTCATCATTATCAACAATGGCAGGCAGGTCCTCCCCAGTTTGCAAATGCTGCA

GCATCATCAGGATTCCAACAGCAAATTAGGACTCCACAAAACTGGCTGCAGAAAAATGGG

CTCAACTCTCTCATGAGACCTTCTTAA

>EVM0023642

ATGGAAGGAGGGTGCTATACATCATCAAGCACTTCAGGCTCAACTTCAGCGACAAGGATA

GAGAAGCGTAAGCATAGCAGGCAACAAAACCAGGAGAAGCCATATAGAGGGATAAGGATG

AGGAAGTGGGGCAAGTGGGTAGCTGAAATTAGAGAGCCAAACAAGAGATCTAGGATTTGG

CTCGGTTCCTATTCAACACCAATTGCTGCAGCTCGTGCATACGACACGGCCGTTTTCTAT

CTCCGAGGACCTTCTGCTAGGCTCAATTTCCCTGATTTGATACACCAAGAAGATGAGTTG

TGTGATGTGTCTGCTGCTTCTATACGCCAGAAAGCCACTGAAGTCGGGGCTAAAGTGGAC

GCCTTACAAACAGCACTCCATGGATCACCTGGAGACAATCCACCAGCAAATCGATTGTTA

CTTTCAGAGAAACCTGATTTGAACGAGTATCCGGAGAATTCTGATGAAGAGAGTTCAAAA

GAAGATCAACGTTAA

>EVM0043533

ATGAAATCTACGGGTGATAACAACAATGGAAGTAATAATAACTGGTTAGGCTTCTCTCTC

TCACCCCACATGAAAATGGAGGTTGCTTCCGACCCCCAACATCATCATCAGTACCATCAT

CAAAACAATGCTTCTTCTGCTGTTCCAACAAGTTTCTACCTATCCACTGCTCACTTCAAT

GGCTCTGGAATCGGTTATGGAGTTGGAGAAAATGATGGATTTCACTCTCCTCTGTCCGTT

ATGCCCCTTAAGTCTGATGGGTCTCTTTGTATCATGGAAGCCCTCACTAGATCACAACCC

GAAGGAATGGTGTCGAGTCCGTCACCGAAACTGGAGGACTTCCTAGGTGGTGCAACAATG

GAAAGTCATCAGTACAGTAGCCATGAAAGGGAAGCTATGGCCTTAAGCCTTGACAGCATA

TACTACCATCAAAATTCAGAGCCAGAAACTAATAGACAACATTCACTCGACCTTCACGAA

CCATTTAGGCAGCAAGACCAGCAGTTTTCAGCCCCACTGGAGGGGGGAACCAAGGGTACT

CAGCTTGCAGATTGTAGCTCACCCGTTCCTCAAATGGGAGACGATGAACTGCCATGCTTA

AAAAACTGGGTCGCTAGACACTATTCCTCCCAAAGTGCACTGGAGCAGCATATTAATAGT

AGCATGGTTAATGATGGCGGGTCTTCTTGTTCTGTTAGTGCTATGGGGTGCGGGGATTTA

CAGTCTCTCAGCTTGTCTATGAGTCCTGGTTCGCAGTCAAGCTGCATTACAGCTCCGAGG

CAGATCTCTCCTACTGGCACTGAAGGCGCTGCTATAGAAACAAAGAAGCGAGGTTCTGCA

AAAGGGGGTCAAAAACAACCTGTTCATAGGAAGTCTATTGACACATTTGGCCAAAGAACA

TCACAGTATAGAGGTGTTACAAGGCACAGATGGACTGGTAGATATGAAGCCCATCTCTGG

GACAATAGTTGTAAGAAGGAGGGCCAGACCAGGAAAGGAAGGCAAGGGGGTTATGATATG

GAAGAGAAAGCTGCAAGGGCTTATGATCTTGCTGCCCTTAAGTACTGGGGCCCCTCAACT

CATATAAATTTTCCGTTAGAAAATTACCAGGAAGAACTTGAAGAAATGAAAAATATGAGC

CGCCAGGAATATGTTGCTCATTTGCGAAGAAAAAGTAGTGGGTTTTCTAGGGGGGCCTCA

ATCTACAGAGGAGTAACGAGACATCACCAGCATGGAAGATGGCAAGCAAGGATAGGTAGG

GTTGCAGGAAACAAGGACCTTTATCTTGGGACTTTCAGTACCCAAGAGGAAGCTGCTGAA

GCTTATGATATCGCTGCGATCAAATTTCGTGGGGTTAATGCCGTGACTAACTTTAACATA

ACAAGATATGATGTTGATAGGATCATGGCCAGCAACACTCTTCTAGCAGGAGAGCTAGCC

AGGCGAAACCGAGATACGGAATCTAGCATTGTTTCCATTGATTACAACACATCAACACAC

AACAATGGTGTTCTATATCATTCATCACAGGAACAGCCAAATGCCTGTGGAGAATCTCTT

GATCAGAAGTCGATGAGTACTGGAAATTATCGAAGCTCTTCTTTCTCAGTGGCATTGCAA

GATCTTATTGGTACTGATCATTCAGTGAACTCTAGCCAGCTAGTGGTAGACGAATCAGCC

AAGATAGGGACTCTTCTCTCTAACCCTTCATCGTTGGTGACCAGTTTGAGCAGTTCTAGA

GAACCTAGCCCTGATAAGACTGCCACCCCTATGCTCCTCGCTAAACCTCCACTAGCATCA

AAGTTCGTTAGCCCTACAACTAGCGTTAATCCTTGGTTCCAAGCTGCCCAATTGCGGCGA

GCGGCAATCCCCATGTCTCACTTGCCAGTTTTTGCTACATGGAATGAATCATAA

>EVM0003138

ATGCTCTTTCAAAAATCAATGGGTTACCCAATTACCCCTCGTCCCCTCCTAGCCGTTATA

CCTTTTCACCCTGCAACAGCCCCCAAACAGCGTCGTGCTTCCTTTGCAACTCTCAAGTTG

TTTGGTTTTTATTTGGATCAAATCATGGCGTCTTCTTCCTCTGATCCGGTCCTGAAACCA

GAAGTCGGACGCGTTGGATGCGGCGGCGGAAGCGGTGGAGGTGGTGGTGGAGAGAGTTCA

GAGGCGGCAGTGATAGCGAACGATCAGTTATTACTGTGCAGAGGAATAAAGAAGCCAAAG

AAAGAGAGAGGATGCACTGCAAAAGAACGTATTAGCAAGATGCCTCCTTGCACTGCTGGT

AAACGTAGCTCCATCTACCGTGGTGTTACCAGGCATAGATGGACTGGTCGATATGAAGCT

CATCTTTGGGATAAGAGTACATGGAACCATAATCAGAATAAGAAGGGAAAGCAAGTATAC

TTGGGGGCATATGATGATGAGGAAGCTGCAGCTAGGGCATACGATCTTGCTGCTTTAAAA

TATTGGGGCCCTGGAACCCTTATTAATTTTCCAGTTACTGATTATAAGAGAGATCTTGAA

GAAATGCAAAATGTCTCAAGAGAAGAGTATCTGGCATCTCTTCGGAGAAAGAGTAGTGGT

TTTTCAAGAGGGCTCTCGAAGTACCGAGCACTATCTAGTCGATGGGACTCATCTTGCAGT

CGTATGCCTGGATCTGAATACTGCAGTAGTGTAAACTATGGTGCAGGTGATGATCGAGCA

GCTGAAAGTGGATATGGTGGCAGCTTTTGTAGTGAAAGAAAGATAGATCTGACAGGCTAT

ATTAAATGGTGGAATTCCCATAGCACTCGTCAAGTGGAATCCATCACGAAGTCATCAGAA

GATACTAAACATGGATGTCCTGACGACATTGGCAGTGAGCTTAAAACATCAGAACGAGAA

GTAAAGTGTACCCAGCCATACCAGATGCCTCATTTAGGCTTATCTGTTGAAGGAAAAAGG

CAAAAAAGTTCTACAATATCTGCCCTGAGCATTTTATCACAATCAGCTGCTTACAAGAGC

TTACAAGAGAAGGCGTCAAAAAAGCAAGAAACTAGCACTGAAAATGATGAGAATGAAAAC

AAAAATACTGTTAACAAAATGGACCGTGGCAAAGCAGTAGAGAAGTCCACGTGTCACGAT

AGCAGTAGTGAGAGGCTGGGAGGAGCTACATCAGGAATGACTGGGGGATTGTCTCTTCAA

AGAAATGTGTACCCATTGACTCCTTTCTTGTCTGCTCCTCTTCTGACCAACTACAACACA

ATGGATCCCTTAGTCGACCCCATTCTTTGGACATCTCTTGTTCCTGCTCTTCCCGCTGGA

CTTTCTCGAAATCCCGAGGCAGGTTTATTCTTACTTAAAGCTTGTCAATATTTCTTTCTT

GTCAAAAAGACTGAGACCAGTTCAACCCATAGCTTCTTTCGGCCAGAGGAGTGA

>EVM0041453

ATGGATTCTTCATATTTTCACTTCCAAAACTCTCTATTCTCCCCGGAATCATCTTCTTCT

TTCAATTCCCGTGATTCTTTCTCATGTAACATACAAAACTTCTACAGCCAGCCCCCCCCT

CTCAATGAAAATCATGATTCCCAAGAAATGTTTCTCTCAGAAGTCCCAGATGAGGTCCCC

GTCAACTCCTTACATACCTCATCATCGACCGACACTACCTGTGACGAAGCAAGCTCCAGA

GCTAGTTATCAAGATGAGCCACCCCAACAAATTGCTTACAGAGGGGTTAGGAAGCGGCCT

TGGGGCAAGTTCGCTGCCGAGATAAGGGATTCAACAAGAAAACATGTGAGAGTTTGGTTA

GGAACTTTTGATACTGCCGAGGCCGCAGCCTTAGCTTATGATCAAGCTGCATTTACAATT

AGAGGTTCGATGGCCGTCCTAAATTTTCCTGTCCAAAAAGTTTACGAGTCACTTCAGCAA

ATGAGCTATGGATTCCAAGAAGGGAAATCACCGATCGTCGCCATGAAAGAAAGACACTCA

ATGAATCGGAAAGCCGAGAGTAGGAGAAAGAAAGAGAAAGGAAGGAGAACTGAAATGGAA

AATGTGGTGGTCCTTGAGGATTTAGGGGCTGATTACTTGGAAGATCTTCTAACCATTTCA

GAGAGTGCTAGTCCTTGGTAA

>EVM0043216

ATGGATGGAAGCTGCATAGATGAATGCACGAATAGTATGGCTGATAACTCAATATCCATT

ACACCAACTTGCCTCTCTCCTTTCCCACCACCTGCTACCACTACAAAATCACCATCTGAG

TCACTTTGCCGTGTTGGGAGCGGCAATAGTGTTATTCTTGATTCAGAATCAGGTGTCGAA

GCTGAATCAAGAAAACTCCCTTCTTCCAAATACAAAGGTGTTGTCCCCCAACCAAATGGT

CGCTGGGGTGCACAGATTTACGAGAAGCACCAGCGTGTTTGGCTCGGGACATTCAATGAA

GAAAATGAAGCAGCAAGAGCCTATGATATTGCTGCCCAGAGATTCCGTGGAAGGGATTCT

GTGACCAACTTCAAGCAAGTTAATGAGACCGAAGATGATGAAATAGAGGCTGCTTTCCTG

AACGCTCATTCAAAAGCTGAAATCGTTGACATGTTGAGGAAACACACGTACAGCGATGAG

CTAGAGCAAAGCAAGAGGAACCACAAGAGTAACAAAGGGGTAAATGGGAAGCAATACAAT

AACTATGGGGAAAATAATTATGATCATGGCTGTGTTCGGGTGTTGAAAGCGCGTGAACAG

CTTTTTGAGAAAGCTGTGACTCCAAGTGATGTTGGGAAATTGAATCGGCTTGTGATACCA

AAACAACATGCGGAAAAGCATTTTCCTTTGCGAAGTACGTCAAACAATAGTACTAAAGGT

GTATTGCTTAACTTGGAAGATGTGAGCGGCAAAGTGTGGAGGTTTCGTTATTCTTATTGG

AATAGTAGCCAAAGTTATGTTTTGACAAAAGGGTGGAGCCGATTTGTTAAAGAAAAGAAC

TTGAAAGCTGGTGACACTGTTTGCTTTCAGAGATCAACTGGACCTGACAAACAGCTTTAC

ATTGATTGGAAACCAAGAGGCGGGTCCAGCCTGGTCCAGCCGGTTCAGATGGTGAGGTTG

TTTGGGGTTAACATTTTTAATGTACCTGGGATGGAAAATGGGTGTAATGGGAAGAGATCG

ATGAGGGATATGGAGATTTTGTCAATAGATCATCAGTATAGTAAGAAGCAAAGGATTGTC

CGAGCTTTGTAA

>EVM0033580

ATGGATTCTTGTTCTCATCAGAACTGGCTGGGTTTCTCTCTTTCCAATCATCATCACATG

AATAATAATATCAACATCCCTTCATCTTCTGATTCCTCTCACCTCTGTCTCTTTGAAGCC

TTCAACCCCGCCCCCGCCCCCGCCCCCGCCCCCAGTACCTCAGCGCAAGAAGTAAATGCA

GTAGTAGCTGCTGGTAGAGCTACGGACATCTCTTTATCTACAACTTCTGGACCAAAACTT

GAGGACTTTCTCGGTAGCTGCACCGCTACATCACCATCACAAACACCACAACAGCCGCCG

TGTGGCCAGTTCTCTACTGAGACACCCATGACTACTACCGCCACTACTTTATCTGACAGT

ACTAGCTCTGAGATATATGATTCTGAGCTCAAAACTATAGCCGCTAGCTTCCTTCGTGGT

TTTGCCTCTGCTGATCATCAAAAAATCGACAGTAACCAAACACATCAACAACTTCTTGTT

CAGGCTGAACATTCACCAAAGAAAACTGTTGAAACTTTCGGCCAACGTACTTCAATCTAT

CGAGGAGTCACCAGGCATAGATGGACTGGCAGATATGAAGCCCATTTGTGGGATAATAGT

TGCAGAAGGGAAGGTCAAAGTAGAAAAGGAAGACAAGTTTATTTGGGTGGCTATGACAAG

GAGGAGAAAGCAGCTAGAGCTTATGATCTCGCAGCTCTCAAGTACTGGGGTCCGACCACC

ACTACAAACTTTCCGGCTTCTAACTATGAGAAGGAAATAGAAGGGATGGAGCACATGACA

AGGCAAGAGTTTGTTGCGTCACTTCGAAGAAAAAGTAGTGGTTTCTCTAGAGGAGCATCG

GTTTACAGAGGAGTGACTAGGCACCATCAACACGGTAGGTGGCAAGCAAGGATAGGAAGA

GTTGCTGGCAACAAAGATCTCTATCTTGGCACCTTCAGCACACAAGAGGAAGCTGCCGAA

GCCTATGACATTGCAGCAATCAAATTTAGAGGCCTAAATGCTGTTACCAACTTTGACATG

AGTCGCTATGACGTGAAAAACATAGCTAAAAGCAATCTTCCCATTGGAGGAATATCCGGC

AAGTCAAAAAATTCCTCAGAATCAGCTTCTGATAGTAAAAGCATTGATGGAAGCCGATTG

GACGATCGAGATATCTCCTCAGCATCCTCAGTAACCTTTGCTTCTCAGCCTGCAACTTCT

ACTCTTAGCTTTGCCATACCCATCAAACAAGACCCGTCAGATTATTGGACCAATATCCTT

GGATACAAAAACACTACCACTGCCATGAACAATGCCAAGAACAGCAGCAGCATTATTGAT

CCAAGCACTTTACTTCAATCTTCCACAAGCGGTCCTGCTTTTCAAAGCCCGACAGGTTTT

AGTATGGACTTCAATGCAAATTCATCTGTCAATGAAAGCAACAGCAGTGGGCTATTATTA

TTCAATGGTGGTTATACACAGCAGCAGAGTGGTATAAGTACATCATCCTCAAGTTCGAAT

ATCCCATTTGCTACACCCATCGCCTTCCATAGTAATGGTAGTAGCTACGAGGGTAACCCA

GGTTATAGTAGCTGGACTGCTCAATCTTTGCATTCTTTCCAATCTGCAAAGCCCAATCTC

TCAGTGTATCGGACTCCCATTTTCGGAATGGAATGA

>EVM0011412

ATGCTCTTTCAAAAACCATTAAGTTACCCAATTACCCCTCATCCCGTCCTAGCCGTTATA

CCTTTTCACCCTGCAACAGCTCCCAAAACAGCGTCGTCATTTCCAAACCGATTAAGGATA

TTGTCGTCATTTAACCATATCGAAAAGGTGCTTCCTTCGCAACTCTCAATTTGTTTGGTT

TTTTATTTGGATCAAATCATGGCGTCTTCTTCCTCTGATCAGGTCCTGAAACCAGAAGTC

GTAGGCGTTGGATGCGGCGGCGGAGGTGCTGGTAGAGAGACTTCAGAGGCGGCAGTGATA

GCGAACGATCAGTTATTACTGTTCAGAGGACTAAAGAAGCCAAAGAAAGAGAGAGGATGC

ACTGCAAAAGAACGAATTAGCAAGATGCCTCCTTGCACTGCTGGTAAACGTAGCTCCATC

TACCGTGGTGTCACCAGGCATAGATGGACTGGTCGATATGAAGCTCATCTATGGGATAAG

AGTACATGGAACCATAATCAGAATAAGAAGGGAAAGCAAGTATACTTGGGGGCATATGAT

GACGAGGAAGCTGCAGCTAGGGCCTACGATCTTGCTGCTTTAAAATATTGGGGCCCTGGA

ACACTTATTAATTTTCCAGTTACTGATTATAAAAGAGATCTTGAAGAAATGCAAAATGTC

TCAAGAGAAGAGTATCTGGCATCCCTTCGGAGAAAGAGTAGTGGTTTTTCAAGAGGGCTC

TCGAAGTACCGTGCACTATCTAGTCAATGGGACTCATCTTGCAGTCGCAAGCCTGGATCT

GAATACTGCAGTAGTGTAAACTACGGTGATGATCGAGCAGCTGAAAGTGAATATGGTGGC

AGCTTTTGTAGTGAAAGAAAGATAGATCTGACAGGCTATATTAAATGGTGGAATTCCCAT

AGCACTCGTCAAGTGGATTCCATCACGAAGTCATCAGAAGATACTAAACATGGCTGTCCT

GACGACTTTGGCAGTGAACTTAAAACATCAGAACGAGAAGTAAAGTGTACCCAGCCATAC

CAGATGCCTCATTTAGGCTTATCTGCTGAAGGAAAAAGGCATAAAGGTACAATATCTGCC

TTGAGCATTTTGTCACAATCAGCTGCTTACAAGAGCTTACAAGAGAAGGCGTCAAAAAAG

CAAGAAACTAGCACCGAAAACGATGAGAATGAAAACAAAAATACTGTTAACAAAATGGAC

CGTGGCAAAGCAGTAGAGAAGTCCACAAGTCACGATGTCAGTAGTGAGAGGCTGGGAGGA

GCTACATTAGGAATGACTGGGGGATTGTCTCTTCAAAGAAATGTGTACCCATTGACTCCT

TTCTTGTCTGCTCCTCTTCTGACCAACTACAACACAATGGATCCCTTAGTCGACCCCATT

CTTTGGACATCTCTTGTTCCTGCTCTTCCCACTGGACTTTCTCGAAATCCCGAGGTCAAA

AAGACTGAGACCAGTTCAACCTATAGCTTCTTTCGGCCAGAGGAGTGA

>EVM0047762

ATGCTCTTTCAAAAACCATTAAGTTACCCAATTACCCCTCATCCCGTCCTAGCCGTTATA

CCTTTTCACCCTGCAACAGCTCCCAAAACAGCGTCGTCATTTCCAAACCGATTAAGGATA

TTGTCGTCATTTAACCATATCGAAAAGGTGCTTCCTTCGCAACTCTCAATTTGTTTGGTT

TTTTATTTGGATCAAATCATGGCGTCTTCTTCCTCTGATCAGGTCCTGAAACCAGAAGTC

GTAGGCGTTGGATGCGGCGGCGGAGGTGCTGGTAGAGAGACTTCAGAGGCGGCAGTGATA

GCGAACGATCAGTTATTACTGTTCAGAGGACTAAAGAAGCCAAAGAAGAGAGAGGATGCA

CTGCAAAAGAACGAATTAGCAAGATGCCTCCTTGCACTGCTGGTAAACGTAGCTCCATCT

ACCGTGGTGCATAGATGGACTGGTCGATATGAAGCTCATCTATGGGATAAGAGTACATGG

AACCATAATCAGAATAAGAAGGGAAAGCAAGTATACTTGGGGGCATATGATGACGAGGAA

GCTGCAGCTAGGGCCTACGATCTTGCTGCTTTAAAATATTGGGGCCCTGGAACACTTATT

AATTTTCCAGTTACTGATTATAAAAGAGATCTTGAAGAAATGCAAAATGTCTCAAGAGAA

GAGTATCTGGCATCCCTTCGGAGAAAGAGTAGTGGTTTTTCAAGAGGGCTCTCGAAGTAC

CGTGCACTATCTAGTCAATGGGACTCATCTTGCAGTCGCAAGCCTGGATCTGAATACTGC

AGTAGTGTAAACTACGGTGCAGGTGATGATCGAGCAGCTGAAAGTGAATATGGTGGCAGC

TTTTGTAGTGAAAGAAAGATAGATCTGACAGGCTATATTAAATGGTGGAATTCCCATAGC

ACTCGTCAAGTGGATTCCATCACGAAGTCATCAGAAGATACTAAACATGGCTGTCCTGAC

GACTTTGGCAGTGAACTTAAAACATCAGAACGAGAAGTAAAGTGTACCCAGCCATACCAG

ATGCCTCATTTAGGCTTATCTGCTGAAGGAAAAAGGCATAAAGGTACAATATCTGCCTTG

AGCATTTTGTCACAATCAGCTGCTTACAAGAGCTTACAAGAGAAGGCGTCAAAAAAGCAA

GAAACTAGCACCGAAAACGATGAGAATGAAAACAAAAATACTGTTAACAAAATGGACCGT

GGCAAAGCAGTAGAGAAGTCCACAAGTCACGATGTCAGTAGTGAGAGGCTGGGAGGAGCT

ACATTAGGAATGACTGGGGGATTGTCTCTTCAAAGAAATGTGTACCCATTGACTCCTTTC

TTGTCTGCTCCTCTTCTGACCAACTACAACACAATGGATCCCTTAGTCGACCCCATTCTT

TGGACATCTCTTGTTCCTGCTCTTCCCACTGGACTTTCTCGAAATCCCGAGGCAGGTTTA

TTCTTACTTAAAGCTTGTCAATATTTCTTTCTTGTCAAAAAGACTGAGACCAGTTCAACC

TATAGCTTCTTTCGGCCAGAGGAGTGA

>EVM0004638

ATGTCTACCAACAGGCAAGAGCCATCCCGATCTTCTGATAGCAGCAACTCTCGAGTGACT

GTCCAGTCTGATGAAGAGGTGTTGCTAGCAACAAGCTGTCCCAAAAAGCGCGCTGGCCGC

AGGATATTCAAGGAGACTCGGCACCCGGTTTTTAGAGGTACGAGGAAGCGGAACGGCAAC

AAATGGGTGTGTGAAATGCGGGAGCCAAACAAGAAGTCACGAATATGGTTGGGAACATAT

CCTACACCAGAAATGGCAGCACGGGCCCATGATGTCGCTGCTTTGGCACTAAGAGGCAAA

TCTGCCTGCCTTAACTTCGCTGATTCTGCTTGGAGGTTGCCTGTGCCGGTTTCAAACGAC

GCGAAGGATATTAGAAGGGCAGCAAATGAGGCAGCAGAATTATTCAGGCCTCAGGAGTTC

GGTGGCTGTCAGGCCAGCCACCAGAACCGCGGTGCAGTGCCAGAGGATTGCTCGAGTGAA

GTTTGTAATGATGGTCGCGGAACTTTTCAAGAAAACGACGTCTTCTTTGAGGAGGCAATG

CTAGACATGCCAGGGTTGCTCGTGGACATGGCAGCAGGTCTTCTACTTCCTCCTCCATAT

TATGTGAGAAATGATAGTTTTGGCCGGGATCATTCGGAGAATGATTCTTATGTGTCATTA

TGGAGTTACTAG

>EVM0057013

ATGGCCACAACTTCATCTAGCTCCAAAAGGCACCCAGTGTATCGTGGAATCCGGAGCCGC

AGTGGCAAATGGGTGTCCGAAATCCGGGAGCCCAGAAAAACCACTCGTATTTGGCTTGGC

ACGTTCCCAGAGCCGGAGATGGCGGCAGCCGCCTATGATGTGGCAGTCCTAGCCCTAAGA

GGTGCCGATGCAGCTCTTAACTTCCCGAGTTCTGTTGGGACTTATCCTGTTCCTGCATCA

ACATCACCCACTGATATTCGTAATGCAGCTAATGCTGCTGCTACATTAAAAAAAGCTGAA

TTGAGCAACAATGCAGCATTAGCCGAACAACCTAGGAATGACTATGCCAATATTGGTACC

TTTTTGGCATCAAGTGGAGAAGAATTTGTCGATGAGGAGGCACTGTTTGATATGCCAAAT

TTGCTGGTGGACATGGCAGGAGGAATGCTGCTTTCGCCACCAAGAATAACCTCATCGCCA

TCTGATGACTCACAGGGAACTTCTGATGGAGAAAGTCTGTGGAGCTATTCTTAA

>EVM0052812

ATGGGAGCACCAACAGGTGATGGTTTGCGAGGCAGGAGGAAGTCATCTTCCAGGGGACAT

CACAGGTTTGTTGGGGTTAGGCAGAGGCCGTCAGGGAGATGGGTGGCAGAGATCAAGGAC

TCGTTGCAAAAGGTTAGGCTCTGGCTTGGAACTTTCGATACTGCTGAGGATGCTGCAAGA

GCATATGATGATGAGGCTAGGGCGTTGCGCGGTGCTAATGCTCGTACAAACTTTGAATTG

CCTCAGCCTGCACCAAATTCTGGTGCTGGCCGTGCTAATCTAGTAGAAGTTGAGCCCTTC

TCATTTGAGGAAGTTTGCCGGACGGGAAGTGAAGCGGAAGGGATTCTTGGTGCACTCAAA

GCCAAGCTGCTTGATGGAAAGGGATTGCGGGTTCTTCCGTCTGCTACTTGTTCTTCAAGT

TTGCAGCCCAACTTGGTGGCGGAAACTTCTCACATAAACGATTCTAAGAGATATCTGGCA

TCGGCAGCCTCTATTCCTCCTGGAGCTGGAACTCTTAATGCTATTCAGGATCCTGGTACT

TCAGGATCTCGTAAGATTGACCTCGTCTTAGATCATGTTGCGGCAGGCCTGTGGAATCAA

CCATGCCATAGTACTACAGCAACAACAAGCTTGGAATGGCCCAGTGAACCAGCACCAAAT

GAAGTCGCCTGGGCCACGCAGATGAACCATATTTCTGATCAGGCCGCTGCTATGTTTACC

GGTAGCACTACAATTGCTACTTCTGCATGGCCACTTTCTGAGACAGCTCAACCAAGTGTT

GATTTGATGTATTCGAATCCGTGCACCTTTGAGGTGCCTAAGAACAAGATTAGGGGAATG

ATTACAGCAAACATGCCAGCATCACAAATTGATGGATCAGCGGAAGTTATTTGGTCTGCT

GAGCAACAATTTCTGCACTATGATAATAGTGGCTGGACCGGTGGTAATAGCACTTGGGAT

CCCTTTCTCTTGTATCCTCATTCCTCAGCGTTAGGGTGA

>EVM0054684

ATGTGTGGCGGTGCTATTTTGGCCGACCTCATCCCTCGTAACCGTGGCCATCGCGTCGCG

GCTTCTGAGTTCTGGCCTAACTCATCCTTCCAGAAACCCGGTCCCTTCGACTCATATCCG

AGTCCTCTCCGCAACCAGGAACCATTCACTCTCAAACGATCCCAAGCCACTTCAGGGGAT

CATGAACAAGTGGAGAAGCCGAATGCCAAGAGGCAGCGGAAGAACCTATACAGGGGTATA

AGGCAGCGACCTTGGGGAAAATGGGCAGCTGAGATTCGTGACCCAAGAAAAGGAGTCCGC

GTTTGGCTGGGTACATTTAACACTGCCGAGGAAGCAGCTCGAGCCTACGACAGAGAGGCT

CGCAAAATTCGCGGCAAGAAAGCCAAAGTCAATTTTCCTAACGAAGATGACCACCACTAC

ACGCCCCAAAACACGAAAACCAAGCCTTTCCTGTACCAAGCTCCCACTTGTCACTTCTCT

AAAGGTTATGATTCTGGGTACGGTTATGATGGATACCAGATCAAAACCTATCATTCAAAT

AGTTTAATCAACGAACCAATCGTTACTTCTGGGGAGGATGATTCTGGGTCAGTTTCAGAG

GAGGTAACAGGACTGCTGGGCTGTAATCAGAGTGTGGAGAGCAATAATTATACGGGTCAA

GTGAAGGTGGAAGAGGAGAAACTGGAGGAGGAAAAGAAAGGGATGAATAAGGAGGTGGTG

ATGGTAGATTTGGAAACTAGGGAGGAAGAAAGCGAGGTACAGAAGCTGACTGAGGAGTTA

ATGGCGTATGAGAACTTCATGAAGTTTTATCAGATTCCGTATCTGGATGGGCAATCAACG

GCACCAAACGGGACTGCCCAGGAAAGCCTCGTGGATAGTCTTTGGAACTTTTTTGATGAT

GGTGCTGATGTTCCTGTCACTTCTGCACCTCTGTAA

>EVM0030954

ATGGCGCAGAAAGATTCTAGCACTATTTCAGCGAAGGTTCTCATACAATCTGACAAAAAA

AGAAAAAATCCAAAGAGGGGAGATAAAACTAGGCTTGATATTTGTACCCAGCTTTTTCGT

TCGAGTTACATGTGCATATTGAAGGTGGCGAATCAAAGAGGAGACCCGGGGGAGTACACC

AGGTACCCTGCGACAGGTAGCTATGACAGCCAGGGAGAGAGATCATACACAGCACCGCAA

ATATTTTCTCAATCGAATCAACCGATAGAGCAGCAGGTGGAGGTCACACAACCACCGCAC

ATGTTCATGGAGTACAGCAGTTCGACGGGGATGTCTCCAATGGTATCCGAGCTTACACAT

GTGGTGTCAGGCCAGAGAGGAAGTGCTGGTGATTGGGGATCATACGGGGCTTTAGGTCTC

GGAGGTCACTCCGCTTCTCCACCTCTATCCGCCTATTCTTCAACTTCTGGTTCTGGTTCT

GGTTTGTGGATTGGCCAAAAGAGAGGGAGAGAAGAAGAGGCTGGTGCAGCAGCGCAACTG

ATGGAGTCTTTGCCAAGGGTTTATAGAGGGTTTAGTGATTTAAGAAGTTCACAGGGAGAT

TCATCATCATCCGCTGCAACTGAAGTGTCTGCTCCAACTACAGTAATCCCCACCACAACT

ACAACCTCCACCACAGCAACACCATCAAGTGAAACTGCATCGCTGGAAGAAACTGGAGAG

CAAAGAAGAAGATACAGAGGAGTTAGGCAGAGACCATGGGGGAAATGGGCAGCCGAGATA

CGTGATCCACACAAAGCAGCAAGAGTCTGGCTGGGCACATTCGATACTGCAGAGGCTGCA

GCTAGAGCCTATGACGAGGCTGCTTTAAGATTCAGAGGAAATAGAGCTAAACTTAACTTC

CCTGAAAATGTTAGATTATTGCCAGCTCAAATGCAAAACGTTGCCGCTTCTCAAGTTCCC

ATCTCTCATTCACAGTTATCTTCTCATAGCCAGTTACAGCCGATCTCATCTCCAAGGCAA

CAAGCACAGCGGCCGCCGCAGGCGGCAGCACCTGCAATGTTTCGGTCTCAGGCTGATATT

ATGAGGGACTACTGGGAGTACTCCCAGTTGTTGCAGGGTTCTGGGGACTTTCATGGACAA

CAACGACAGCAGCCGCCACCTTCAAGTTTGATACAACATATGTTTTATAATCCCCAGGTG

GCTTCTCTACAATCACCAGCTTTGTCTTCATTGTCTTCATCTTCTTCACTTGCAGCGATA

TCTTCCGGTTCATTACCATCAACATTTTCTCCCTCTGCCGCTTCTTCCTTCCCTCTGCTT

TTTGATGTTCAACAGTTGGGTTATTTTCGGCCACCACAAAACCAGAATCAATCTAGTGGT

TCTGATTTTCCGGTGCCCCCATGGACAGATTCTAGTCACAATCCTTCCTCTAATGGTTAA

>EVM0004204

ATGGCTTCTTCAAGGGAAGGACACTACAGGGGAGTACGAAAGAGACCATGGGGTCGCTAT

GCTGCAGAAATACGTGACCCATGGAAGAAAACACGAGTTTGGTTAGGCACATTTGATACA

CCTGAAGAAGCTGCTCTTGCTTATGATGGTGCAGCTCGTTCTCTTCGTGGAGCTAAAGCA

AAGACTAACTTTCCCTCACCACCTTCCACTTCTGGTCTCTCTCTGGACCTTAATCTTCCA

TCTGACCCTCACCACACCCACCACCATCTTCGTTGGAGTTCAAGTCCTCATGTCGGGTCT

CACAGGTTTGGCGGTTTTGGCGAGTTCTTGCAGACTGGAGTAGTTTTAAAAGAAATGAAC

TTCAATGCTACCGAGGCTGCAGCAGCTTCTGGGTCAGTAGTGAAAATAGAGGGTCCTGGC

GTGGGTGCTGTATCTGGACCTCCGGTGCCGGAAAATGTTGCCCCGGCTTCGTTTTTGGGG

ATGGTTCGACGTGGGTTGCCAATAGATTTGAACGAACCTCCACCTTTGTGGCTGTGA

>EVM0049898

ATGGCACCTAGAGAGAAGAAGGCGGCCATTAAAGCTAACGGCGGCGGAGCTGGTGGTGGC

GGTAAGGAAGTGCATTTTAGAGGAGTGAGAAAGAGGCCGTGGGGAAGGTACGCTGCCGAG

ATAAGAGACCCGAGTAAGAAGAGCCGGGTTTGGCTTGGCACCTTCGACACGGCGGAGGAA

GCTGCACGTGCCTACGATACTGCCGCTCGTGAGTTCCGTGGTTCGAAGGCAAAAACTAAC

TTTCCATATCCATCGTGCGAGAACGCTAAAAAAAACGTTGTCTCCATCGACAACAGCAAG

AAAGCTGCCAGCAAGAATAACAGTTGCGGTGGTGGCGGCGGCGGTAATCAGAGTCCCAGT

CAAAGTAGTACCGTGGAGTTCTCTAGCAGTGATGCACCGTTGGATCTTAATCTCGATCCT

GGTGCATCCACCGTTAGATTGCCGTTCCAGCCGATGGTGATGAATCATCAACAGGCGGTT

TATTTTGATGCATTGATGAAGAGCCAATATCGAAGGATGGTTTTCGATCATGGGTATCGT

CATCATCCTGACCAGCCGGTGGTTTTTTCTTGCGGCGGGGTCCTAAGTGATTCTGATTCA

TCCTCTCTGGTGTATTTGAATCATCAAGATGTCAAGACACCGAGTAGATTTGTTGATCTT

GATCTTAACCTTCCTCCCCCACCGGAGATTGCATGA

>EVM0012041

ATGGTGTTAACCTCCCAGAGCCATGATCTCCCTTTGAATGAGAATGACTCACAAGACATG

GTCATATATGACATGATCAATGATATCAGTGCCCCAAATAGCAGCACGTGTAACACCCTT

CCAAGAAGCCATGTCAGCACTTCAAGCATGCTCCGACCAGCCATAGCAAAGAAGCACTAC

AGAGGCGTGAGGCGTCGGCCATGGGGAAAATATGCTGCCGAAATCCGAGACTCCAGACGA

CGCGGGGCCCGAATATGGCTAGGCACATTCGAAACGGCAGAGGAGGCTGCCCTGGCTTAT

GATAGGGCTGCTTTTAATATGCGCGGCTCTAAGGCCCTCCTTAATTTTCCAGCTGAAGTA

GTTGCTGCACCTACATCAACTCAAAACCTTCAGCCAATTTTGAGTTCAACAAGGTCAAGT

GAAAATGCTTCGGATACAAGTCGTAGCTGTAGAACAATATCAATTCCAACATCGCAATCT

GAATCAGAGAGTTGCCAAAGTGGGGAGCCACGCCAAAAGGGGTCTGATATCGTAGAGAAT

TAA

>EVM0028403

ATGACTATACAAAGTGATCATCGTTCAGCATCCGAAGGAATCTTAGAGAATGTTTGGGCA

AGCTACATAGGAGAAGATAGAGGAGACAGAGGAATTAGTAATGGTGAACAAGAAGTGTCT

AAATCATGGAAGGAATTGCCTAGTCTTGATAGGAGAGACGAGTCGATGGAGGTTCTACGA

AGGCTACCGAGTCTAGGCAGGTGGATCTCGATGGGTGCTGAGACCTGGGAGGAACTTCTT

GATGGAATCATTCCGGAAATTAACAATACCTGCAATGATAGTGCAGAAAACAAAGGCCCC

AGCAACCTATGCTCCACAGCGAGTACTATGAAAGCTGAGAAAGTGACCGCCAGGCATTAC

CGGGGAGTGAGGAGGCGGCCATGGGGAAAGTACGCAGCAGAAATAAGGGACTCGTCAAGG

AAAGGAGCTCGAGCTTGGCTTGGGACTTTTGAGACAGCTGAAGAAGCAGCTTTGGCTTAC

GACAAGGCTGCTCTGAGAATCAGAGGCCCCAAGACATACCTCAACTTCCCACTTGAAACA

GTTGCTAAGGCTATGTGCATTGACTCCTCCAAAAATGACTATAATGTTTTTTCCACCACA

ATTTCTCAAGGGAACGACACTTCTTGCACATTTCTCGGAAGCAGTGACAAAGTTTCAGCT

ATCCATAGAAAAAGGGCTTCGAGAGATTGGGAAGTGAAAAGTGATTCGACGATGATGGAA

CAACCCGGACTCAAGAGAATGGCTCTCGAACATCCTGAACTCGACGTGGTAGAATTTCAG

GACCTGGGGAGTGACTACCTGGACAGTTTACTGTCCTCTTTATAG

>EVM0029476

ATGGCTATACGAAGTGATCATCGTGCAACATCTGAAGGAATCCTAGAGAATGTTTGGGCA

AGCTACATTGGAGAGCATAGAGGAGACAAAGTAACAAGGAATGATGAACAAGAAGTGCTT

AAATCATGGAAGGAATTGCCTAGTCTTGATAGTAGAGATGGGTCAATGGAGGTTCTAGAA

AGCCTACCAAGTTTAGGCAGATGGATATCGACGGGTACCGAGTCCTGGGAGGAAATTCTT

GATGGAATCAATCATGAAACTAACAGCCTAAGCTCCAAAGAGAGTACTGTGAAAACACAG

AAAGTGACTTCCAGACACTACCGGCCGGGGTGTAAGGAGGCGGCCTTGGGGCAGGTATGC

AGCAGAGATAAGGGAAAAGGAGCTCGAGTTTGGCTTGGAACTTTTATAACAGCTGAAGAA

GCACCTCTGGCTTATGATAAGGCTGCTTTGAGAATTAGAGGCAAGGCAATGGGCATCCAC

TGCTCCAAAAATGACGACAATGTTTCATCTAACACAACTTCACAAGGGTATGACACGTCT

TGCACATCTCTGGGAAGCGGTGACGGAGTTTCAGTGATCCCTAGAAGAAGGAGAATTGCT

TGCGTAGAAGAATTGTTTGCGGATGATTTCGACGTGGTAGAATTTCAGGTCATGGGGAGT

GACTACTTGGACAATTTGTTTTCCTCTTTATAA

>EVM0035055

ATGGTGTTAACCTCCCAGAGCAACGAGCTGCCGTTTAATGAGAATGACTCACAAGACATG

GTCATATATCAGATGATCAACGAGGCAGGAGCCCCAAATAGCAGCACATATAATATACTT

CCGAGAAGCCAGATCAACACTCCGAGCATGCTCCAACCAGCTCGGACAGTCATAGCAAAG

AAGCATTACCGGGGCGTGAGGCGTCGGCCATGGGGCAAATATGCTGCCGAAATTCGAGAC

TCCACAAGACACGGGGCTCGAATATGGCTCGGCACGTTCGAAACGGCTGAGGCGGCTGCC

CTGGCTTATGACAGGGCTGCTTTTAACATGCGTGGCTCGAAGGCCCTCCTTAATTTTCCA

GCTGAAGTTCTGGCTGCTATATCAACTCAAAACCTTCAGCCAATTTTGAGCTCGACGAGG

TCACGTAAAAAAGCAATGAATTCACGTGGCAGCTCAAGCACGGTAACTATCGCAGCATCA

CAATCTGAATCAGAGAGCAGCACAAGTGTGGAGTCACGCCATCGGGGGTCGGATATCCTA

GAGAGTTAA

>EVM0048064

ATGGCACCGAGAGAGAAGACAGCGTCCGTTAAAGTTAACGGCGGCGCATGTGGTGATGCT

GCTGGAGGAGGATGCAAGGAGGTGCATTTTAGAGGTGTGAGAAAGAGGCCATGGGGAAGA

TTCGCCGCCGAGATAAGAGACCCTAGTAAGAAGAGCCGGGTTTGGCTTGGCACTTTCGAC

ACGGCGGAGGAAGCCGCACGAGCCTACGATGCTGCCGCTCTTGAGTTCCGTGGTTCTAAG

GCGAAAACCAACTTTGCTTACCCATCATACGAGAATGTTAGGGAAAACACGATCTCTGTG

GATAAAAACCATAAAACATCTAACAAGAACAACAGTTGTGGTGCTGTCGGAAATTGCAAT

ACCAACCATAGTCCAAGTCAAAGCAGTACCGTGGAGTTTTCAAGCAGTGATACACCGTTG

GATCTTAATCTCGGTCCTGCTGCATCCACCGTGAGACTGCCGTTTCAGCCGATGCTGATG

AATCATCAGCAGGTGATTTATTTTGATCCCGTGAAGAGTCAGTGCCAAAAGATGGTTTTG

GATGACGGTCATCATCATCATCATCCTAACCAGCCGATGAGTTTTTCTTGTGGTGGGTTC

CAAAGTGATTCTGATTCATCCTCTGTCGTCCTTGATTTGAATAATCAAGATATCAAGACG

ACGAGAAGTGTTGTTGATCTTGATCTTAACTTTCCACCCCCACCGGAGATTGCATGA

>EVM0001371

ATGGCTTCTTCGAGGGAAGGACACTACAGGGGGGTTCGAAAGAGACCATGGGGTCGCTAT

GCTGCAGAGATACGTGACCCATGGAAGAAAACACGAGTTTGGTTAGGCACATTTGACACA

CCTGAAGAAGCTGCTCTTGCTTATGATGGTGCAGCTCGTTCTCTTCGTGGAGCTAAAGCA

AAGACTAACTTCCCGTCACCACCTTCCACTTCCGGTCTCTCTTTTGACCTTAATCTTCCA

TCGGATCCTCACCACCGCCATCTTCGTTGGGGTTCAAGCCCTCATGTGGGGTCTCACAGG

TTTGGTGCTCTTGGTGAGTTCTTGCAGACTGGAGTAGTTTTTCAAGAAATGAACGTCAAT

GCTACCGAGGCTGCAGCAGCTTCTGGGTCAGTAGCGAAGAATGAGGGCCCTGGTGTTGGT

GCTGTTGCTGGGACTCCAGAGCCAGAAAATGTTGCTCTGGTTTCGTTTTCGGGGATGGTG

CGACGTGGGCTGCCGATAGATTTGAACGAGCCTCCGCCTTTGTGGATGTGA

>EVM0034803

ATGACTAGACCGCAGCAGCGATACCGCGGCGTCCGTCAAAGACACTGGGGCTCTTGGGTT

TCCGAAATTCGCCATCCTCTCCTGAAGACTAGAATATGGCTGGGCACATTCGAAACGGCG

GAGGACGCAGCAAGAGCATACGATGAGGCAGCAAGGCTAATGTGTGGGCCGAAACCACGT

ACAAATTTCCCTTACAATCCCAATGAGCCACAATCATCTTCATCGAAGCTTCTCTCAGCT

ACCTTGGCAGCCAAGCTACATAAATGTCACATGGCATCTCTACAAGCAACCAAGAAAAAT

GAAACAGGACAGTCATATGAAGCACAATGCAAGAAGACATTTACCTCTAGCCATGGCATT

GCCGGAAAAACTGTCGAAACCTGCTCAAAATGGCAGGAGGGGAACTGGGTTGGTGAGGAT

AGTCAAGTAGGGAACGGTGTTCATGATCAGCAGCATTTCAAGTCACTTGAAGCTCATCAT

ATAGAGCAAATGATAGAGGAGTTGCTAGATTGTGGGTCCATGGAGTTCTGCTCTGTAGGT

TCACCATGA

>EVM0040725

ATGTCTTGTATGGTTTCAGCTCTCACTCAAACCATGGGTGCTACCGACAATAACGTCCCG

AGTTCGGTGCAATCAACCCCATTTGCTCTCGACCAAACCGTCGTCAAAGACGAACCTGAT

CAGTCTCAACCAGTGCAAGATCAAGAAAATACGAGGAGAAGGCATTACAGAGGAGTGAGA

CAAAGACCTTGGGGTAAATGGGCAGCCGAGATACGTGACCCTAAAAAGGCAGCTCGAGTC

TGGCTCGGTACTTTTGACACTGCTGAGGATGCAGCTGTTGCATATGACAAAGCAGCACTC

AAGTTCAAAGGCACCAAGGCTAAGCTTAATTTTCCTGAAAGAGTTCAAGGGAGAACCGAG

TTCGGCTACTTCATGGGTTCTGGGGATTCAAGCAATGTCTCGACTGAACAAAATCCGAAG

CCAGTTCCTCCTCCTCCCCCTCCCCCTTCATCATTTGCACCGGACACTTACCCGGACTTG

CTTCAATATGCACAGATCCTTTCTAGCAATGATGCCAATTTCCCGTACTACACGTCAAAC

CTCTTTAATCAACAAGCTTATCCCTCCCATTTTTCACAAAGCTTTTTATCACAGCAACTA

CTAGATCAACAACAACAAGATGTAATGAGATTCTCGTCAGGATTCGATGGCTTCTCTAGC

TCATATCAACAGGAACAAGGGAAGGACTCCAGTAATCCTGGCGAGTAG

>EVM0032830

ATGGCGGTAGACGCGTGGCGCCACCGTAACGACACTGTTACAACAACCCTCTCCCTTGCA

GGACCTCCAGCTTCCCCCACACCAGTGGTGGAGGATAAACCTCAACGCCACTTCAGAGGC

GTGAGGAAGAGGCCGTGGGGTCGATTCGCTGCTGAGATTCGGGATCCATGGAAGAAGACG

CGGCGTTGGCTTGGAACTTTTGACACGGCCGAGGAAGCGGCACTTGCTTATGACAAGGCA

GCTCGATCTCTCCGTGGACAAAAGGCAAGGACAAACTTCTTCCACCGTGACCATCTTGCT

CCCCTGCCTCATTCTCAAACAACGGTGGCTGGTGGCGGTGATTTAAAGATACGGTGCTCG

CCAGTCTGTCTTTGTGATGACGTGTCAAGAATGGCTGCGCCAGCGGCGGTGGATCCGGTT

AGGTCAGAGCATCAGGGGTACAAGATCGAGAATGTGAATGATCAAAACATGGGTTTGGTT

TTAAATGAAGAGCAGCAAAGGCCGTTGTTGTTTGACTTGAACTTGCCGGCTCCACTCTTT

TGA

>EVM0035539

ATGGCGTCTTCTTCTTCTTCTGATCCGGTTCTAAAACCAGAAATCGGCGGCGGTATATGT

GGTAGCGGAAGCGGAGGCAGAGGTGGAGGAGAGAGTTCAGAGGCGGCGGTGATAGCAAAC

GATCAGTTATTACTATACAGAGGACTAAAGAAACCAAAAAAGGAGAGAGGATGCACCGCT

AAAGAACGTATTAGCAAGATGCCTCCTTGTACTGCTGGTAAACGTAGCTCCATTTACCGT

GGCGTCACCAGGCATAGGTGGACTGGTCGATATGAAGCTCACCTTTGGGATAAGAGTACA

TGGAACCAGAATCAGAATAAGAAGGGAAAGCAAGTATACTTGGGGGCATATGATGACGAG

GAAGCTGCAGCTAGGGCATATGATCTAGCTGCTTTGAAATATTGGGGCCCTGGAACACTC

ATTAATTTTCCAGTTACTGATTATACAAGAGATCTTGAAGAAATGCAGAATGTCTCCAGA

GAAGAGTACCTGGCATCTCTTCGGAGAAAGAGCAGTGGATTTTCAAGAGGAATCTCAAAG

TACCGTGCACTGTCTAGTCGATGGGATTCGTCTTATAGTCGTGTGCCAGGATCTGAATAC

TTCAGTAACGTAAACTATGGTGCAGGTGATGATCAAGCAGCTGAAAGTGAATACAGTTTT

TGTATTGAGAGAAAGATAGATCTGACAGGCTATATTAAATGGTGGGGTTCCAATAAAACT

CGTCTGGCAGAGTCTATGTCAAAGTCATCCGAAGATACAAAACATGGCTGTGCTGATGAA

ATTGGCAGTGAACTCAAAACATCAGAACGAGAAGTGCAATGTACTGAGCCATACCAGATG

CCCCGTTTAGGTTTATCTGTTGAAGGAAAAAGGCATAAAGGTTCTAAGATATCTGCGTTG

AGCATTTTGTCACAGTCAGCTGCATACAAGAACTTGCAAGAGAAGGCATCGAAAAAACAA

GAAACTGGCCCTGAAAATGATGAGAACGAAAATAGAAATAATATCAACAAGATGGACCAT

GGCAAAGCAGTCGAGAAGTCCACAAGTCACGATAGCAACAGTGAGAGGCTTGGAGCCGCA

TTAGGAATGACTGGGGGATTGTCTCTTCAAAGAAATGCGTATCCATTGACTCCTTTCTTG

TCTGCTCCTCTTCTGACCAACTACAACACAATCGATCCCTTAGTAGACCCCATCCTTTGG

ACATCTCTTGTTCCTTCCCTTCCCACTGGACTTTCTCGAAATCCTGAGGTTACAAAGACT

GAGACCAGTTCATCTTATAGTTTCTTTCGGCCGGAGTGA

>EVM0055941

ATGTGTGGCGGTGCTATCATCTCAGATTTCATAGCTCCGACAACCACCGCTCGATCTTCT

AGGAGGCTGACCTCGGGCTTTGAGTGGTTTGAACGGAAACCTTTCGACAACAAGAAGCAG

TCGAAGCCAGTTGGTGCTGATCTTGAAGATGATTTTGAGGCTGATTTTCAAGGGTTCAAG

GATGAGTCTGATGTCGAAGAGGATTATGATGTCTTTGTTGATGCCAAGCCTTCTTCTTTC

TCTGCTTCTGAACCTACTAAAAAACGCGCGTTCTCTCCCGCAGCACCTAAAGTATCTGTG

CCTGTGTATGTTATTGAAGGTCCTACTGCTGTTATATCTGCTGGATTCAATAGACTTGCT

GAAAAATCAGCAAAGAGGAAGAGAAAGAACCAGTTTAGAGGAATTAGGCAGCGTCCATGG

GGAAAATGGGCTGCTGAGATTCGTGATCCCAGGAAAGGGGTACGAGTCTGGTTGGGAACA

TTTAATACTGCAGAAGAAGCTGCTAGAGCATATGATGCCGAGGCCCGTAGAATTCGTGGC

AAGAAAGCAAAGGTGAACTTCCCCAATGAAGCTCCATGTGCTTCAGGAAAGCGTACAATT

AAGGAAAACTCACAGAAACATCTTACAAAGGCAAATTTAAGCCAGAATTTCAGTTACACG

AGCAACCCAGAAACAGATTATAATAATATGGGCTTTGTGGAAGAGAAACCACAATTGAAC

CAGTTTGGAATAACGAGTTCTTTCTCGGCCAGTGGAGACTCTGGGGTGACGCCCTTAACT

CTTTCTGACAACGCTCCTGTGTATTTCAATTCTGACAATGGGAGCAACTCATTTGATTGT

GACATGGGGTGGGGAGAACAAGGCCCAAATACTCCTGAAATCTTGTCTGTTCTTGCAGCA

ACTTCAGAAGTTGATGAATCTGTCTTCGTGGATGCTAATCCTAAGAAGTTGAAACCGTAC

TCCGAGAATGCAGTGCCCGTTGAAGAGAAGAATGAAAAATCTCTGTCCGATGAGTTGCTG

GCTTTTGACACTCAGCTGAAATACCTACAGATGCCAGATCTTGTGGGTAGCTGGGAGGCT

TCTCTTGATAGCTTCCTTGATGGAGAGACAACTCAGGATGGCACAAACGCAATGGACTTG

TGGAGCTTTGATGATTTCCCCTCTATGGTTGGGGGAGTTTATTGA

>EVM0012457

ATGAATCCATCTTCATCGAAAAGCAAAAGGAAGCAACCACATCAAGCTCAGCAGGAACCA

GGTACTGGATTAAGGTTTCTTGGTGTTAGGAGAAGGCCATGGGGAAGATATGCAGCAGAG

ATACGAGACCCTTCAACAAAAGAAAGGCATTGGCTAGGCACCTTTGACACGGCTGAGGAA

GCTGCCTTGGCCTACGACGGAGCCGCTCGCTCCATGCGCGGTCCCCGTGCTCGCACAAAC

TTTGTTTACTCCGACATGCCTGCTGGTTCATCCGTTACCTCCATTATCTCCCCTGAAGAC

CAACAGTTTCTACAGCAGCAGCAGCAACTCAAACAAAATAGCGACCACGATGACAGCCTG

TCTTCATTATTCTTCAATGCCCGCCCCCCCACTCATGATCTTCAGCCGGACTCGACTCCC

ATTTTCAACCAGGATTTCAGCTCTCAGTGCCATTTGCGAGACGGATGTTCTTCAATGATT

TCTGATGGAGATTTTTGGTGCTGCTCCAGCACCAGAACTTATAATCAACAGCTACAACAC

GTTAATGATTCTAATGTACTTCCTGATCATGAGTTTCCTTCTGATTTATCTCTCGACTCG

GGTTTGGCCGATTCATCATCTTCAAGGTTGATGGGCTTCGAGGACCTGACAGCAATGACT

ACTGGGTTTGACTCGGTGGGGGGTAGTGGTGGTTCTTCTTTCTGGTTTGATTCTGGCGAA

TACGTTCACAGTCCACTTTTTAGCAGGATGCCGCCAGTTTCAGATATGGTACCAGATGGT

TTTGATTTGAGCTCCTCTGCTTATTTCTTCTAA

>EVM0018299

ATGTCGTTAGCCCCGCCGGAAAGCAACTGTGGAAGTGATAAGCAGGCTGAGAGTAGTGGA

AATTCCGGAAGGCACCCATCATACCGTGGAGTGAGAATGCGTCAATGGGGCAAATGGGTG

TCCGAAATCCGGCAACCAAAGAAGAAGTCAAGAATTTGGCTCGGATCTTTCTCAACAGAA

GAAATGGCGGCTCGAGCACACGATGTAGCTGCCCTTACAATCAAAGGCCGTTCAGCTCTG

CTTAATTTTCCCGAACTCGCTCATGAATTTCCACGCCCGGCTAGTTCGTCTCCCAAAGAT

ATTCAAGCTGCCGCTGCCCTAGCAGCTACCTTGACCTGCAAGACAAGTCAAAAGGGCCGC

GAAACTGAAGATGGAGCCGAGCTTATGCTTCCTCGCTCCCTGGATAGTACTTCGGCATCT

ACTGAGACGCCGGAGTATTCATTAAGCTCACCATTAAGAGATGATGACACATTTATCGAC

TTGCCAGATATTTTGCAAGACATAAGCCATCGATTTGATGAGTTTTGTTACTTGTCACCA

TGGAAGCCGATTGGAACCGAGACTTCTGATGATGGATTTTGGCGTCACGAGGAGCCTTCT

TTATGGGAAATACCACTCAAGTAG

>EVM0009409

ATGGATGAGGACGGGACAAGGAGCAAGGATGAAGGAGGAGAGGTCAAGTATAGAGGCGTA

CGGAAGAGGCCATGGGGTAAATTCGCGGCGGAGATACGTGATTCAACCAGGCACGGAGCG

AGGATGTGGCTGGGGACTTTTAACACGGCGGAGGAGGCAGCAAGAGCATATGACAGAGCA

GCTTATGCAATGAGAGGGCACTTGGCCATTCTTAATTTTCCGAACGAGTATCCCAATATG

GCAAGTGCTGGTTCCAGCAGCTCTACTCGCTGTTCAGGTTCTTCTTCTTCTTCATCATCA

ATGCAACGAGAAGTTTTTGAGTTCGAATGCTTGGATGATAAGTTGCTCGAGGACATGCTT

GAGCAAGAAGAGAAAAGGAGTCAGAAAAAGTAA

>EVM0038648

ATGGATGGCTCCTTCTTTCAATCTTCAAATTCTGATTTTTCATCCGAATCTTGTTTTGAA

TCGCCAGACTTTTTCTATGGCCGATCTTTTAACCAAAGTTCTCTCCCCTTCAATGAAAAT

GACTCCGACGAAATGCTTCTTTTTGGGCTAATCTCGGAGGCGACTCAAGAAACGTCAAAA

ACAACTTCCTATAATGGAGTTATCAAGGAAGAAGAGGTTAGTTCCATATCCAAAGAAGAT

CCCGACAAGGAAAAGTCCTACAGAGGTGTCAGGAGGCGGCCATGGGGCAAATTCGCGGCA

GAGATAAGGGATTCCACAAGGCACGGCGTAAGGGTGTGGTTAGGCACATTTGACAGTGCA

GAGGCAGCTGCTTTGGCTTACGATCAAGCTGCTTTTTCAATGAGAGGAACTGGGGCGACA

CTCAATTTCCCAGTTGAAAAAGTGAGGGAGTCACTGAAGGATATGAAGTGTACTGATGAT

CACGAGGATGGGTGCTCGCCTGTGGTGGCTCTAAAGAGGAAGCACTCCATGAGAAGGAAA

CTGGGAAACAGAAGCAAGAAAGAGAGGAATGTTAGGATAGAGAATGTGGTGGTTTTGGAA

GATTTAGGTGCTGATTATCTGGAGCAACTATTGAATTCATCTGAAGATGCTGCTAGTCCT

TGCTGA

>EVM0021312

ATGGAAATTCATTTCCAACAACAAAAGCAGCATAAACATGCAAGCATTCCAGTTAGCAAA

GCAAGCAAACTCAAGGGGAGAAGTAGGCCGAACAGCAACAGTAACAAGTTTGTTGGTGTT

AGGCAAAGACCTTCAGGTAGATGGGTGGCTGAGATCAAAGACACAATACAAAAGATAAGG

ATGTGGCTAGGAACCTTCGAGACAGCTGAAGAAGCTGCACGAGCTTACGATGAAGCTGCT

TGCCTGCTTCGTGGATCCAACACACGAACCAACTTCGTCACTCATGTGTCCTTGGATTCT

CCTCTTGCTTCCCGGATTAGAAATCTACTCAACAGCAAAAAGAGAGACAAACAACAACAG

AATGAAGAAGAGGAAGAGGCTGCTGAAGTCTCTACTCCACCAACTAGTACCATTACTACC

ACAAGTAGTTCTATCACCACTAGGAGCAGCAGCAGTTCTGGCATGAATGATAATTACTCT

CATTCCGGTTCAATTCATCAAACGGATCACTTGTTTGAGGATGCATATAAACCGGATTTG

AGCAACTGCTTTGAGGAGTTCAAGTTGGGTTCTTTCCAGTCTGATCTTTCATCTGTATTC

GATCGGTTTTCATATGCACAGGAAGTATTGGATTTTCCAGAAAGTGTGGTATTACCCGAG

GCAAGTGACTTGGGATTCTCAGAATTTGATAGGATGAAGGTGGAGAGACAGATATCAGCA

TCTCTATACGCAATTAATGGGGTGCAAGAGTACATGGATGCTGTCTATGATCCTATTGAA

GCTTTATGGGATATTCCACCACTATAA

>EVM0049397

ATGGCTTCTTCAAGGGAAGGACACTACAGGGGGGTTCGAAAGAGACCATGGGGTCGCTAT

GCTGCAGAGATACGTGACCCATGGAAGAAAACACGAGTTTGGCTAGGCACATTTGATACA

CCTGAGGAAGCTGCTCTTGCTTATGACGGTGCAGCTCGTTCTCTTCGTGGAGCTAAAGCA

AAGACTAACTTCCCATCACCACCTTCCACTTCTGGTCTCTCTCTGGACCTCAATCTTCCA

TCCGACCCTCACCACAATCTTCGTTGGAGTTCAAGTCCTCATGTCGGGTCTCACAGGTTT

GGTGGTTTTGGCGAGTTCTTGCAGACTGGTGTAGTTTTAAAAGAAATGAACTTCAATGCT

ACCGACGCTGCAGCAGCTTCTGGGTCAGTAGTGAAAATAGAGGGTCCTGGTGTTGGTTCT

GTAGCTGCACCTCCGGTGCCGGAAAATGTTGCCCCGGTTTCGTTTTTGGGGATGGTTCGA

CGTGGGTTGCCAATAGATTTGAACGAACCTCCACCTTTGTGGCTGTGA

>EVM0052258

ATGGCACCTAGAGAGAAGAAGGCGGCCATTAAAGCTGACGGCGGCGGAGTGGGTGGTGCT

GGTGGAGGTAAGGAAGTGCATTTTAGAGGAGTGAGAAAGAGGCCGTGGGGAAGGTACGCT

GCCGAGATAAGAGACCCGAGTAAGAAGAGCCGGGTTTGGCTTGGCACTTTCGACACGGCG

GAGGAAGCTGCACGTGCCTACGATACTGCAGCTCGTGAGTTCCGTGGTTCGAAGGCCAAA

ACTAACTTTCCATATCCATCGTGCGAGAACGCTAAGAAAAACGTTGTCTCTATCGACAAC

AGCAAGAAAGCTGCCAACAAGAATAACAGTTGCGGTGGCGGTAATCAGAGCCCCAGTCAA

AGTAGTACCGTGGAGTTCTCTAGCGGTGATGCACCGTTGGATCTTAATCTTGATCCTGGT

GCATCCACCGTTAGATTGCCGTTTCAGCCGATGGTGATGAATCATCAACAGGTGATTTAT

TTTGATGCATTGATGAAGAGTCAGTATCGAAGGTTGGTTTTCGATCATGGGTATCGTCAT

CATCCTGACCAGCCGGTGGTTTTTCCTTGTGGCGGGGTCCTAAGTGATTCTGATTCATCC

TCTGTCGTTGATTTGAATCATCAAGATGTCAAGACACTGAGAAGATCTGTCGGTCTTGAT

CTTAACCTTCCTCCCCCACCGGAGATTGCTTGA

>EVM0053591

ATGGAAGGAGGGTGCTGTACATCGTCAGCAAATACTTCAAGCTCAACTGCAGCGGCAGGC

ACACAGAAACGGAAGCATAGCAGGCAGCAAAACCAAGAGAAGCCATATAGAGGGATAAGG

ATGAGGAAGTGGGGCAAGTGGGTTGCTGAAATCAGGGAACCAAACAAGAGGTCGAGGATT

TGGCTCGGGTCCTACTCAACACCAATAGCTGCAGCTCGAGCATACGACACGGCTGTTTTC

TATCTGCGAGGTCCTTCTGCTAGGCTTAATTTCCCTGATTTGATATACCAAGAAGATGAG

TTGCGAGATGTGTCTGCTGCATCTATACGCAAGAAAGCCACTGAAGTTGGGGCTAAAGTT

GATGCCCTACAAACCGCTCGCCATGCATCACCAGAAGATAACTCACGAGTCTTAATTCCA

GAGAAACCTGATTTGAACAAGTACCCAGAAAAGTCGGATGAAGAGTGA

>EVM0057697

ATGTGGAACTTGAATGACTCGCCTGATCAAACAAGAGACGATGAATCCAAGGGATGGTCA

AGTCAAAAAACATCAATAGGTGGCGAAGTTGACAAGGGAAAAAAGGTCGGATCCGTGTCG

AATTCCAGCTCTTCTACTGTAGTAATTGAGGATGGATCAGAAGAAGAGGATGGTTGTGGA

GAGAGAGGAAATATATTAATCAAGAAACATAGTATAAGCTTCAGCAGCAAGATTTTCGGG

TTCTCGGTTCCTTATCATAAAGACTCCATGGACACGAGCGATCCACCGGTGACCCGCCAG

TTATTTCCTTTAGAGGATCAAGAAATGGGGTCCGCGTCGAGAGTTGAGGGTGCATTTCCT

CGGGCTCACTGGGTTGGTGTCAAGTTTTGCCAATCGGATTCATCACCTGTTTCTCAAAAA

TCCATGGAAGTCTCACGGCCATTGAAGAAAAGTAGGCGAGGACCGCGGTCCAGGAGCTCT

CAATACCGTGGTGTTACCTTCTACCGGAGGACTGGCCGATGGGAATCTCATATATGGGAT

TGTGGCAAGCAAGTGTATCTAGGTGGATTTGACACTGCACATGCAGCTGCTCGTGCGTAT

GATAGGGCAGCTATCAAGTTCCGGGGAGTGGAAGCAGATATTAATTTTAGAATAGAAGAA

TACGAGGAAGACTTAAAACAGATGAGCAACCTCACCAAGGAAGAATTTGTTCATGTACTT

CGCCGGCAAAGCACAGGATCTCCTAGAGGAAGCTCCAAGTACAGAGGCGTAACCCTGCAC

AAGTGCGGCAGATGGGAAGCTCGAATGGGCCAGTTTTTAGGCAAAAAGTATGTTTATCTA

GGCTTGTTTGATACTGAGATCGAAGCTGGAAGGGCCTATGACAGAGCTGCAATCAAGTAC

AATGGCAAAGAGGCAGTGACCAACTTTGATCCCAGTATTTATGAAAACGAGCTTGAATCA

TCAGGGAATGCTGCGGCCCACAACCTTGATTTGAGCTTGGGCAATCCAGCTTCGAAGCAA

AGTAGTATAGAATTTGGACAGGATAGGCACAATGCTGCCATGGAGCAACTTTCAGCAACG

ATGTCATTTGAACCTAGTTGGCAAAATCGGGGGTTCAGGCCTAAGCTTAACCCCTATGGA

GTTGACAGCGATGGACATGGAGGAGATGGGCATCTGGAGACTGAAACGACCCAGCTTCTT

AGCAAAATCCACTTTCAATCTCTAGCATCGCTCAAGTCTAGAGAGATGCCAAGTTATGAG

CAGTTCAGGAGACCGCATGGAGGAGATGGTCAGATGCTTCATGTTCTTCCCCCACAGTTC

AATCCACCAAGTTATCAGATCCAGTATCCAAGCAGTAGCAATGGAGGCCGAACCGGAAGT

GATATTTCCCTCTCTCTCACCGAGCTTCATCGTCGTCGTCAATATCAGCAATGGCAAGCA

GGACCTCCCCAGTTTGCAAATGCTGCAGCATCATCAGGATTCCAACTGCAGATTAGGACT

CCACAAAACTGGCTGCAGGAAAATGGGTTCGACTGTCTCACGAGACCCTCTTAA

>EVM0026318

ATGGAAAGCGGAGTAGACAAGGAGATGGCGACAAGAAAGAGAGGTGGGATGGGAGGTGAG

AGGCAATACAAGGGAATAAGGATGAGGAAGTGGGGGAAATGGGTGGCTGAGATACGAGAG

CCTAACAAAAGGTCAAGGATTTGGCTTGGTTCCTACTCATCACCGGTAGCGGCAGCTCGC

GCTTATGACACTGCTGTTTTTTATTTGAGAGGGCCGTCGGCGAGGTTGAATTTTCCTGAA

TTTTTGGCTGGAGAGAATTTTGGCGGCGGTGGATCATTTGGGGACATGTCAGCTGCTTCT

ATAAGGAAAAGAGCAACCGAGGTTGGAGCTCGTGTCGACGCTATTGAGACAGCGTTCCGC

CGCCGCCGCCATCATGATGACCGTCAAAGAAATAGTAGTAACAGTTCCAGTAGTAATGAT

AATAACGAAACGGTCGTTGATAGTAGAGAGTTAAAGTCAGGGACGGTTGACTTGAATAAG

ATGCCTGACCCGGAGGATCAAGATGGAGATGAGTGGGATAGGAGTTAG

>EVM0046939

ATGGCACCCAGGAAGCTTAATGTTAGCGACGGTGGCTCTTTCAGGAAAGCAAGTGGGGAC

CATGGCAAGGAGATACATTACAGGGGAGTGAGGAAGAGGCCGTGGGGGAGGTATGCTGCC

GAGATAAGGGATCCCGGGAAGAGAAGCCGGGTTTGGTTGGGTACTTTTGATACGGCGGAG

GAGGCTGCTAGAGCTTATGATAAAGCGGCGCGTGAGTATCGGGGTGCTAAGGCGAAGACC

AACTTTCCTTTCGCTGAGGGGGTGGTTAATTATGACGATGATAAGCAGAGTTCTAGCCAG

AGCAGCACCGTGGAGTCTTCGAGTTCTCCGGCTGCTTCTGCGGTGGTGACCCGCCAGGTT

GGTGGAGTGGGCGGGGTTGTGGGGATGGGAGGGTTTCCTTTTGTGTACCAGCAGCAGAAT

GTTAACGTTGTTGCTCCTGTCTGGTTTTTTGATAGTGCGAGGCCGGAGTTTACGACTCAG

CGATTCCCTGTCCGGTTTGACCAGGTGGGTCTTGAAAGTGCTGGCTGTGCCCAGAGTGAC

TCGGATTCATCAACGATGGTTGATTGTCAGCCAAGGAGAACAGTTCTTGATCTTGATCTG

AACTTGCCTCCACCGTTGGATGCTTGA

>EVM0030767

ATGATTTCAAGCTCCAAAAGCCCCATCATCAATGTGGAGACTTTGTCATTAAGCAACAAA

CTTGGAAGGCTTCATAGCAATATTGGTAATGTAGAAAAATTCAAAGGCATTGTGCCACAA

AAAAAGGGACATTGGGGTGCACAAATATATGCAAATCATCAAAGAATTTGGCTGGGAACA

TTCAAGACTGAGAAAGAAGCAGCCATGGCCTATGATAGTGCTGCCATAAAGCTCAGAAGC

ACGGATTTGCACCGAAATTTTCCATGGAACGACCACAATGCTCAAGAGCCTAGTTTCCAA

AACCAATATAGCACAGAAAGGATTCTGAACATGATTAGAGACGGATCTTATCAACAGCTT

TTTGTGGATTTTCTAATGAAACAGTCACGAAGAGAAGAAATTTGTGGAACTACTGATGAT

CTCAATGGAAGGAGGGTGCATGTAGATGATGAACAATTTTCATGCATGCAACTTTTTCAA

AAGGATCTGACACCAAGTGATGTAGGGAAGCTTAATAGACTTGTGATACCCAAAAAAATT

GCTGTCAAGTACTTCCCCTACATTTTTAAAGATGTCGAAGATCAGAGGGTACTAAATTCT

GGGGTAGATGACACAGAACTCGTATTTTATGATAGGTTCATGAAGTCTTGGAAATTTAGA

TATTGCTATTGGAGAAGTAGCCAGAGCTTTGTTTTCACAAAGGGCTGGAATAAGTTTGTC

AAGGATAAAAAATTGAAGGAAAAAGATATCATTGCATTTTATACATGCGCGTTCCCAGAG

AAGGTGCAAGAGCAACATGGTCAGGGCCAATATTTTTCCATGATTGACGTATTTTATTGC

AATGAACAAAGTGGCAAGGTTGCTGGGTTTAACCACATGGAGGTTATGCAGAGAGAATTG

GCAGTGATTTTAAAGCAAAATATGAGAAAAAAGCTACAGAGGGACGGGAAAGAATTGAAG

GAGGAGAGAGTATTGAAAAAAAAGGAACTGAAAAACAAGGTTAAAGGAAAAGGGTTTAGG

CTTTTTGGGGTGCAGATCAGTGATGTTTTTCATTTGGAAACTGAGGTTGCGTTGCAGCTC

CAGCCTTCTTCCCAAACGGGTGCCAAGTGA

>EVM0049295

ATGGCGAGAGAGAGAAGAAATTGTAAGGGGCGTTACAGGGGAGTGAGGATGAGAAAGTGG

GGAAAATGGGTAGCAGAAATAAGGCAACCAAATAGTAGAAATAGAATATGGTTAGGTTCA

TATAATACTGCAGAGGAAGCAGCAAGAGCATATGATGCTGCTGTTTTCTGTTTACGTGGG

CCTTCGGCGACGTTTAATTTTCCATCGAATGTACCGGAAATTCCTTCAACGACGGAGATG

TTGTCTCCCGCGCAGATTAGGGAGGTTGCTTTTAGGCATGCAAGAAGGGGGAGTACTTTG

GAAGCAGCAGAGAGGATTGCGGAATCTGGGTTGTTTGAAGGGCCATCTGGGAGGAGTGGA

GAGGTGTATCTGGGTGGTGGAGTCGAAGGGGCGGAAAATTTAGAGGGAATTCCGAGTGGG

GCATATTATCAAACATCTGATGTGTTTGTGCTGTGA

>EVM0035105

ATGGTGAAGTACAGTGCCGGGGAGCCAATTGCAGAGAGAAGAGACTCGAAATTCAAGGGT

GTTAGAAAAAGAAAATGGGGCAGGTGGGTGTCTGAAATCAGGCTACCGAATAGCCGGGAA

AGAATCTGGTTAGGATCGTATGATTCTGCAGAAAAGGCTGCTCGAGCGTTCGATGTGGCC

CTTTTTTGCTTACGCGGTCGAGTTGCGAAATTTAATTTCCCGGATAACCCTCCAAACATA

GCGGGTGCCAGGTCACTTTCTCCGGCTGAAATTCAAAAAGTTGCAGCAAAGTTTGCCAAT

TCGGAGCCTCAAAGAAGCCAATCGGATCAGTTGGAAACAAATCAATCTGTATCTGAATCT

CGAGCAGAATCTCCCTGCCCTTCAGTTGTATCCGAATCTCGAGCAGAATCTCCATGCACT

TCTGTTGTATCCGATGGGACTGTCCAGATGGAAAGTAGTGAATTGAGCTGGGATGGGCCG

TTTTTGGATATGTTAATGAATACAAATTCCAGTAAATCCCCATGCACTTCTGCTGTATCC

GATGGGACTGTCCAGTTGGAAAGTAGTGAATTGAGGTGGGATGGGCCGTTTTTGGATATG

TTAATGAATACAAATTCCAGTAATTACTCCGCCGAATATGGAATATTTCCGGGTTTCGAT

GATCTTCATAATGATTTTTTCCCATCATCATCAACTCCTAATCTCGATTATGGAGAGGAG

ACCAATTTTGATGGTGTTTTAGAACAGCATTCATTTCTTTGGAATTTCTAA

>EVM0000418

ATGGAAAGTTCTGGCAGCTTCGATTCTTCTTCTATAGTCAATGCTGATGGCTATGGTGAT

GAGGAATCCTGCTCCAACGGCGACGTTTTGGCTTTCAATTTCAGTGTCTTGTCAAATGAT

ATCAGCGTCAAGAAGACCGTTGATTGTGATAATAACGATGATGATGATGGCAGTGGAGAT

CGAACGATCCAGCTTTTTCCAGTGGCGTGTGGGATCAAGAATGCAGGAGGAGAGTCGCGG

TCTTCTTCGACGATGCAGATGCAGCGGTCGGGTCTCGGTGAGTGCTGGGATCAAGGTTCC

CAACCGGAGCTCGGGATCGTGGCTCAACAAAAGCCAGTGAAGAAGAGTCGGCGTGGGCCC

AGGTCGAGAAGCTCCCAGTATAGAGGAGTCACGTTTTATCGGAGAACTGGAAGATGGGAA

TCTCATATTTGGGATTGTGGAAAGCAAGTTTACCTGGGGGGATTTGATACTGCACATGCT

GCAGCTAGGGTGTATGATCGAGCTGCAATTAAGTTCCGTGGAGCTGACGCTGATATCAAC

TTTAATGTTACTGATTATGATGGGGATATTAAGCAGATGCCTAATTTCACAAAGGAAGAA

TTTGTGCATATCCTACGTCGTCGGAGCACTGGATTCTCGAGGGGCACCTCAAAATATAGA

GGAGTGACACTGCACAAATGTGGGCGATGGGAGGCTCGTATGGGGCAGTTCCTGGGGAAG

AAGTACATTTATCTGGGGTTATTTGATAGCGAGACAGAAGCTGCAAGGGCTTATGACAAA

GCTGCCATTAAATGCAATGGAAGGGAAGCAGTTACCAACTTTGAGCCAAGCACGTATGAA

GGAGAGATTCTTTCCGAGCCTCACAATGGAGACGGCGACCAAAATCTTGACCTGAACTTG

GGAATTGGTCCTCCTGATACATCTGATGGCCCAAAGGTAGACAGCAACATGGGTGGCTTT

TATTTCCAGAGTGGCTGGGATGACATTTCTTTTGATAGGGCACCAAAGGTTCTGAGCTCT

GCTTCTGCAACAATGAAAAATAAACAACCTCATGGCCCAGGAATGACATCTGTACACCCT

CCTACGTGGGATAGTGTAAACCATCGAATCTTTACCATCTCTGAGGAAAGAGCAATAGAG

AAGAGAATGGTAGATTCCTTTCCAAATTGGACCAGGCAAATCCAAGACCCTTATGGTGGC

GGAGCTAATCCACTGCCTCTCTTTTCTACTGCAGCATCATCAGGATTCTCTTCTTCTGCA

ATTATGGCACCTCCAGCAGCTGCCGGTCAACTCTACTTTCCAAGGTCCACAATCCTCAAC

CGTTTCCCTCCAATTTCCAACCCCAGCAACATCTCGCAATTCTACTGTAGTAGCTGA

>EVM0045883

ATGAGTATACAAAGTGATCATCGTTCAGCATCCGAAGGAATCTTAGAGAATGTTTGGGCA

AGCTACATGGGAGAAGATGGAGGAGACAAAGGAATTAGTAATGGCGAACAAGAAGTGTCT

AAATCATGGAGGGAATTGCCTAGTCTTGATAGGAGAGACGAGTCGATGGAGGTTCTACGA

AGGCTACCGAGTCTCGGCAGGTGGATCTCGATGGGTGCTGAGACCTGGGAGGAACTTCTC

GATGGAATCATTCCGGAATATAACAATACCTGCAATGATAGTGCAGAAAACAAAGCCCCC

AGCAGCCTATGCTCCACAGCGAGTACGATGAAAGCTGAGAAAGTGACCACCAGGCATTAC

CGGGGAGTGAGGCGGCGGCCATGGGGAAAGTATGCAGCAGAGATAAGGGACTCGTCGAAA

AAAGGAGCTCGAGCTTGGCTTGGGACTTTTGAGACAGCTGAAGAAGCAGCTTTGGCTTAC

GACAAGGCTGCTCTGAGAATCAGAGGCCCCAAGACATTCCTTAACTTCCCACTTGAAACA

GTTGCTAAGGCTATGTGCGTTGACTGCTCCAAAAATGACTCCATAGTTTTGTCCACCACA

ATTTCTCAAGGGAACGACACTTCTTGCACATTTCTTGGAAGCAGTGACGAAGTTTCAGAT

ATCCATAGAAAAAGGGCTTCGAGAGACTGGGAAGTGAAAAGTGAGTCGACGATGATGGAA

CAACCTGGACTCAAGAGAATGGCTCTCGAACAACCTGAACTCGACGTCGTAGAATTTCAG

GACCTGGGGAGTGACTACCTGGACAATTTACTGTCCTCTTATCTTTGGTAA

>EVM0037464

ATGGCGAAATTATCACAGCAGAACACAAAAAACATAGCAAGTAACAATAGCAACACGACT

AATGGTGTGATCGCTGGCTTCGTTATATGCTGGTGGGCAGGTGCCTATGATGATGAAGAA

GCGGCAGCGCATGCTTATGACTTAGCAGCACTGAAGTACTGGGGTCCTGACACAATTCTT

AATTTTCCGTTATCGACATACCAAAATGAACTGAAAGAAATGGAGGGTCGGTCGAGAGAG

GAGTGCATTGGATCATTGAGAAGGAAAAGCAGTGGCTTTTCTCGTGGAGTTTCAAAATAC

CGAGGAGTTGCTAGGCATCATCACAATGGGAGGTGGGAAGCTCGGATTGGTAGAGTATTT

GGCAACAAATACCTGTATCTCGGGACATATGAAGAAGCTGCCACCGCTTACGATATGGCG

GCTATTGAATACCGTGGCCTGAATGCTGTTACCAACTTCGATCTTAGCCGGCACATCAAA

TGGCTAAAACCCCATCAAAATTACAGCAATAACAATTACGATCCAGGTCTCCCTAGCCCT

ATCATAGGCACTGATAATTCAACCCATCCTAACCCTAACCATAACCATAACCATAACCAT

AACCACGAGCTTGGAGCAACCTCCCTTCAAAACCAACAAACGTACCAACCTACCCTAGGG

CTCTTACTTCAAACATCAAAGTTCAAGGAAATGATGGAAATGACAGCAGTGGCAGATTGC

CCACCAACCCCGTCGCCGGGCTTAGACCTGATGACACCGTGCAGCTTCCTTGAAGACGTA

CAGACATATTTTGACCGTCACGAGTCAAGTGGCTACGGGGATCAAGCGGCTGACATGATT

TTTGGAGACCTCAGCTCGTATGTGCCACCCATGTTTCAGTGCGATTTCGAGACATAA

>EVM0043933

ATGGATTCTACTTCTCATCAGAACTGGCTCGGTTTCTCTCTTTCCAACCATCATCACATG

AATAATATCAGCATCCCTTCCACTTCTGACTCCTCTCACCTCTGTCTCTATGAAGCCTTT

AACACTACACCCACCACCACTACCTCAGCACAAGAAGATAATGCAGTAGCTGCCGGTAGA

CCAGCTGACATCTCTTTATTTACCACTCCTGGACCAAAATTAGAGGACTTCCTTGGTGGC

TGCACCACTACACAGCCACCACAACAGCCATTGGGTGGTCTGTTCTCGACTGAGACACCC

GGGACTACTACGCCTGCTGCTGTATCTGACAATAGTAGTTCTGAGATATATGATTCCGAG

CTCAAAACTATAGCTGCCAGCTTATTTCGTGGTTATTCCTCTACTGATCACCAACAAACC

GGTAGTTCTCAAAAGCATCAACAACTTCTTTTGCAGCCTGAACATGCACCGAAGAAAACT

GTTGATACTTTTGGCCAACGTACTTCAATTTATCGAGGAGTCACCAGGCATAGATGGACT

GGTAGATATGAAGCTCATTTGTGGGATAATAGTTGCCGAAGGGAAGGCCAAAGTAGAAAG

GGAAGGCAAGTTTATTTGGGTGGCTATGACAAGGAAGAGAAAGCAGCCAGAGCCTATGAT

CTCGCAGCTCTCAAGTACTGGGGTCCGACCACCACTACAAACTTTCCGGTTTCTAACTAT

GAGAAGGAGCTAGAAGGGATGAAGCACATGACAAGGCAAGAGTTTGTTGCGTCACTTCGA

AGAAAAAGTAGCGGTTTCTCCAGAGGAGCCTCCATTTACAGGGGAGTGACAAGGCACCAT

CAACACGGTCGGTGGCAAGCAAGGATAGGAAGAGTTGCCGGAAACAAAGATCTCTATCTC

GGCACCTTTAGCACCCAAGAAGAAGCTGCCGAAGCTTATGACATTGCAGCAATCAAGTTT

AGAGGCCTAAATGCTGTAACCAACTTTGACATGAGTCGCTATGACGTCAAGAGCATAGCT

AACAGCAATCTTCCCATTGGAGGAATATCTGGCAAGTCAAAGAACTCATCAGAATCAGTT

TCTGATAGCAAAAGCATTGATGCAAGTCGATCAGACGATCGAGATCTCTCCTCAGCGTCC

TCTGTGACCTTTGCCTCTCAGCCCACTACGACTTCTACTCTTAGCTTTTCCATGCCCATC

AAACAAGACCCATCAGATTATTGGACCAATATTCTTGGATACCAAAACACCACCAACACC

ATGAACAAGAACAGTAGCAGCATTGTTGCACCAAGCACTTTACTTCAATCTTCCACAGGT

TTTCCTGCTTTTCAAGGTCCAACAGCTTTCAGTATGGACTTCAACACGCATTCGTCAGTC

AATGAAAGCAGCCACAGTGGGATCTTATTCAATGGTGGTTATATAGAGCAACAGAATGGT

GGTGATGGTATCAGTACATCATCATCCAGCTCGAGTATTCCATTTGCTACACCCATTGCC

TTGCATGGTAATGGAAGCAGTTACGAGGGCAACTCAAGTTATGGTAGCTGGATTTCTCAA

TCTTTGTTCCAATCTGCGAAGCCCAACCTCTCAGCGTATCAGACACCTATTTTTGGAATG

GAATGA

>EVM0018696

ATGGTGAAGTACAGTGCCGAGAAGCCAATTGCAGAGAGAAGAGACTCAAAATTCAAGGGT

GTTAGAAAAAGAAAATGGGGCAGGTGGGTGTCTGAAATCAGGCTACCGAATAGCCGGGAA

AGAATTTGGTTGGGATCGTATGATTCTGCAGAAAAGGCTGCTCGAGCGTTCGATGTGGCC

CTTTTTTGCTTACGTGGTCGAGTGGCGAAATTTAATTTCTCGGATAACCCTCCAAACATA

GCGGGTCCCAGGTCACTTTCTCCGGCTGAAATTCAAGAAGTTGCAGCAAAGTTTGCCAAT

TCGGAACCTCAAAGAAGCCGATCGGATCAGTTTGAAACAAATCAATCTGTATCTGAGTCT

CGAGCAGCATATCCCTGTCTTTCAGTTGTATCCGAATCTCGAGCAGAATTCTCCTTTTTA

ATATGGAAATTCTGTAAAAATCTTAAGAAGACTAGTTTTAAAAATAAATGGATGCAAATC

CTTTTCATATATACATGCTCTCTTACTGAAGGCCACATACTATGCTAA

>EVM0027097

ATGACGATGCAGACAGAAACCTACACAAGAGAGATTGAAGAGATGCTGAGGATGAGCAGG

GAAGAGTACTTGGCATCACTTCGACGGCGAAGTAGTGGATTCTCCAGGGGAGTCTCCAAA

TACCGTGGGGTGGCCAGGCATCATCAGAATGGGCGATGGGAAGCTAGAATCGGTCGAGTT

TATGGGAATAAATATCTCTATCTCGGAACATATAATACGCAAGAAGAGGCAGCGGCAGCA

TATGATATGGCAGCAATAGAATATAGAGGAGCAAATGCTGTGACCAATTTTGATGCTAGC

AATTATATAGAATGGCTGAGGAAGAAACGCATCCCTGTAGACCAAATCCTCCAAGAACAG

CAACTCCGTAACAGCTCGACTGATTCCGCCTTAGAAGCAGAAGCAGAGGCTGAAGAACCA

TCACCAGAACAACAAGAGGAGCAAGAACAAAAGGTAGCTCCGTCGTTACAGGTTCACTGC

ACACAGCTCAATTCAAGCTTGGATGGCACATCTCCTATGGCGACTATCGAAGAGAACGAG

CTAGAATGGAGCTTCTGCATGGATCCAGGACTGAACCTCACAATGCTCGATCTTCCTCTC

GAAAATACCTGGGAGTTACCGAACCTGTTCAATCATACAGGGTTTGAAGACAACATTGAC

CTGATATTCGATACATGTTGCCATGGAAACTTGTAG

>EVM0015330

ATGTTGACACACTGCCCCAGCACCATGTCCGTACAAACAAAAGTCAATTCTACTAAACAT

GTATTCGCCACTAAAAAGCTCATCCAGAACCAGCAGCAGCATCCTTCTACAAGGATCCGC

GGCCAGAGAATAGTTAGAATCATCCACACCGACCCTGATGCCACTGACTCCTCCAGCGAC

GAAGAAGAAGAACAAGATCAAGAGAGGAAGTTTGTCAAGAGAGCGAAGACACGAGTGAGT

GAAATAAGATTGTTAGAGCAAGAAACAACAGCACCAGCAGCTCCTCCAAGAACTTCAAAT

GATCATCATCGACACCCAACAAGAAAGAGGCCTTCTTCAAGACTACCGGTATCCGACGTC

ACTCGCAGGAAAAACTTCAGAGGTGTCCGTCAGAGACCCTGGGGAAAGTGGGCTGCCGAA

ATCTGTGACCCGACCCGACGGAAACGGGTCTGGCTAGGCACTTTTAGCACTGCAGAGGAA

GCTGCTACTGTCTACGACAGAGCTGCAGTGGAACTAAAAGGTCCCGACGCCGTCACCAAC

TTTCCTACTAAATCCGTTGTGACGGTGAAAGCTAACGACGATAGTCCAGACGTCCAGTGC

GGGTCATGTGACTCACCGTCTAGCAGTGGTGTTAACGTTATGCCATCACTCGCGTCAGTC

CTCCGTTACGAGGAGCTGGCGCCGTTTGATCCTGTTTTGACCGAGATGTCGAAGGTCATC

GACGATCACGGCGAGTCATGCGAGTCACCGTTTAGTGCTAACGTGATGACTTCACCAACG

TCTGTTCTTCGTTACGAAGAGTCGACGCTGTTTGATAGTTTGGCTTACGGTGACGTTGAC

GCTTTTGGGTTTAAAATTGACGTGCCGTCAGGTTTGCCGGACTTGATGTTGCCGGTGAAT

TTTGTCAACGATGAAGAGTTCGTTGACCTCGATGATTTCTTGGTGGAGGCCATTTGTTAA

>EVM0009348

ATGGAGAGTTGCAGAGAATCTCCATTGAAGCCGTGGAAGAAAGGACCAACAAGAAGCAAA

GGTGGTCCGCAAAACGCCATGTGTGACTACCGAGGGGTCCGTCAAAGAACATGGGGAAAA

TGGGTGGCAGAAATAAGGGAACCCAAGAGGAGATCCAGACTATGGTTGGGTTCTTTTGCT

ACGGCGGAAGAAGCTGCCATGGCCTACGATGAGGCTGCAAGGAGATTGTATGGACCAAAT

GCTTATCTTAATCTACCTCACCTTCAGTCAAACTCTAAGCCTCCCAACAAATCACACAAG

TTCAAATGGATTCCTTGCAATAACTTCATCTCAATGTTTCCTTCTTGCGCGTTGCTTAAT

ATTCATGCACAGCCTAGCGTTCATGTCATCCATCAGCGGCTCCAAGAACACAAGAACAAC

AGGGCCCTTAATCAACCCTTAACTGAAGTTATTATTGGAAGTGACGAAAACCATGTTGCA

AATGTTTCCGTAGCTGAGAAAGATGAAGAAATATCATCAGAGACGATGCTGCTAACAAAT

CATGACGAGAAACCACAGATTGATTTAAACGAGTTCCTTCAGCAGCTGGGCATCCTGAAA

GAAGAGAAACAGCCAGATGACAATGATGTCAATGAAGATTTCATGGAGCCAGAATCTTCA

CGAATATATCAAAACGAACTTACAACCTTGGTGGACAAGAGTTTTGACTGGGATTCAATG

AATGAAATGCATGGAATTGCTGATCATCAAGGAGAGGAATTGAATAGTTTCCCAGTTTAT

GACATCCAAGAAGATCTGGTTTTCCCAACTTCCATTTGGAACTTCTAG

>EVM0036226

ATGGTTAGCTTACGGAGGCGTAAACTCTTGGGACTTTGTGCTGGGAGAAGTTCTTTCCTG

ACTCCACTTCCTCGATTTTTTGACAATGGAACTGCTCCTGTAGGTTCTGCTCAGAATGGC

AGGTCTGTCAGTGTGCATCCTCTGCCATCAGATGATGCCAACCTGCCAGAGGAGAAAACC

ATTGCAAAAGTAGGAGCTGGCTCTTCAAATGTTTCGGCTTCTAGCTCGTCAAAAGAGCAG

CGATCTCAACCGTATCCAGAGCAACCAGTTAAACGCAGAAAGAGACACAGGAGGAAACAT

GTTCAGAACCAGGAACCATGCCTGATGAGGGGCGTCTATTTTAAAAATATGAAATGGCAG

GCAGCAATTAAAGTTGACAAGAAGCAGATTCACCTAGGGACTGTTGGTTCTCAAGATGAT

GCTGCTCATTTGTATGACAGGGCTGCTTTCATGTGTGGGAGGGAGCCCAATTTTGAGCTC

TCAGAGGAGGAGAAGCAAGAACTTAGGAAATTCAAGTGGGATGAGTTTTTGGCAATCACT

CGCAGTGCAATTAACAATAAAAAACACAAGAGAAGGATCGGGGTAGGTTTGCAGAAGAGA

TCAGAGACCACATTGCAGGACGGTGACTGGGACGGCAAGGAACGACTCAATGGCTTTTCA

GCTTCAGAAGATGTGGAACCAGACTCATCAGCCTCTTGA

>EVM0052605

ATGAGTATACAAAGTGATCATCGTTCAGCATCCGAAGGAATCCTAGAGAATGTTTGGGCA

AGCTACATGGGAGAAGATGGAGGAGACAAAGAAATTAGTAATGGCGAACAAGAAGTGTCT

AAATCATGGAGGGAATTGCCTAGTCTTGATAGGAGAGACGAGTCGATGGAGGTTCTACGA

AGGCTACCGAGTCTCGGCAGGTGGATCTCGATGGGTGCTGAGACATGGGAGGAACTTCTC

GATGGAATCATTCCGGAAATTAACAATACCTGCAATGATAGTGCAGAAAACAAAGCCCCC

AGCAGCCTATGCTCCACAGCGAGTACGATGAAAGCTGAGAAAGTGACCACCAGGCATTAC

CGGGGAGTGAGGCGGCGGCCATGGGGAAAGTATGCAGCAGAGATAAGGGACTCGTCGAGA

AAAGGAGCTCGAGCTTGGCTTGGGACTTTTGAGACAGCTGAAGAAGCAGCTATGGCTTAC

GACAAGGCTGCTCTGAGAATCAGAGGCCCCAAGACATTCCTCAACTTCCCACTTGAAACA

GTTGCTAAGGCTATGTGCGTTGACTGCTCCAAAAATGACTCCAATGTTTTCTCCACCACA

ATTTCTCAAGGGAACGACACTTCTTGCACATTCCTTGGAAGCAGTGACAAAGTTGCGGAT

ATCCATAGAAAAAGGGCTTCGAGAGACTGGGAAGTGAAAAGTGAGTCGACGATGATGGAA

CGACCTGGACTCAAGAGAATGGCTATCGAACAACCTGAACTCGACGTCGTAGAATTTCAG

GACCTGGGGAGTGACTACCTGGACAGTTTACTGTCCTCTTATCTTTGGTAA

>EVM0028845

ATGGTGCAACCAAACAAGTTCAGAGGTGTCAGGCAACGCCACTGGGGCTCCTGGGTTTCT

GAGATTCGTCATCCTTTACTAAAAAGAAGGGTGTGGCTCGGAACATTTGATACAGCTGAA

GAGGCAGCAAGAGCCTACGATGAAGCAGCTGTTTTGATGAGTGGCCGTAGCGCCAGAACA

AACTTCCCGGTGGTTGCAAATCAAACAAGAAATGCTCAGAACTCTCCTTCCTCCTCCTCC

TCAGCTCTCGGTGCAAAACTACGCAGATATTGCAGATCTCCATATCCTTCCATAACTTGT

CTAAGGCTTGACGCCGAGAATTGCCACATCGGAGTCTGGCAAAAGCGTGCCGGTGCGCAT

TCGGTTTCAGATTGGATTATGACTGTTGAGCTTGGAAAGAAGGATGGACGGCAAGCCCAG

GAACAAAAGACTCTCATCTCAGATACATCAAATATGGCAGGCCAAGAAGGTGGTAGTGAT

GCAGGTCTAGATGACGAGCAAAGGGTTGCGTTGCAGATGATAGAGGAGCTACTGAATAGG

TAG

>EVM0005032

ATGAATGAAGAAAACACTAGCTCAAGCAATTCGATATGTAATTCACCAGCACCACCGCCA

TCATCTTTGGTCACTACCATTATCAATAAGCTCAACAAACAAAACCCACTTCAGCAAAAC

TCAAGAAAGGCCAGAGACTGCAGCGAACACCCAGTTTATACGGGTGTTAGAAGGCGAGCC

TGGGGTAAATGGGTGTCCGAAATCCGCCAACCTCGTAAGAAATCACGTATTTGGCTCGGA

ACATTCCCTGCGCCAGAAATGGCAGCTCGTGCCCACGATGTGGCAGCGCTGAGTATCAAA

GGTGACTCTGCCATATTAAATTTTCCAGAACTTGCTGACTCATTACCACGCCCAGTGTCG

GTTATGCCACGTGACATTCAGTCAGCCGCGGCGAAAGCTGCTGCCATGGTCGAGTTTAAC

TCATCATCACCATCCTCATCACTGTCGTCATCATCGGTAACGGTGTCTGAAGATGTGGAA

AAATCCGAGGAAGAGTACTTGAGTGAAATTGTGGAGTTACCGAATATAGAAGGGAGCTTT

GATTCTCCTGATCAGTCACAAACTGAGTTTATGTTGTTTGACTCGGTCGATGGGCATCGA

TGGGTGTATCCACCTTTAGACTTATGTGGAGACTTTTCTGATCACTTACTGGGCCTGGAG

AACTTGATTTGA

>EVM0055695

ATGGCAACCCCAGAAGAATCCTCCACACTAGAGCTCATAAGACAACATCTTCTTGGAGAC

TTCACCTCCACAGATGAGTTCATCAATAATCTTGAGTCCAGTGTAGCCAGTATATATGTC

AAGATGGAGAACTCCTTATCTGGGTCTGAATCCAACTCCCCGATATCAGACCAAAGTTAC

TGTACCCAGGAAACCTGTAGGATTGAAATAAAATCCGAAATCATAGATTTGACACCTCCC

GAGCCCATGTTTTCAGATTCAAGTAATCAGTCCCCACCACCTGAACTCGTAAAGATGACA

GATAAAGATGAGACAGCAAGGCATTACAGAGGGGTCCGAAGAAGGCCGTGGGGAAAGTTT

GCGGCAGAGATCCGTGACCCAACCAGGAAGGGTAGTCGGGTTTGGTTAGGTACTTTTGAC

AGTGATATCGATGCTGCAAAAGCTTATGATTGTGCTGCATTCAAAATGAGGGGCAGGAAG

GCTATCCTGAATTTTCCTTTGGAGGCTGGACTATCCAGCCCGCCTCCGGCCACAGGAAGG

AAGAAGAGAAGAGTGAAGAGAGAAGAGGTGCTGACAGAATCTGTTGATGCCTCGCCGGAA

AACTGGAATGCAGGGTGGAGTGGAGAGGAAGCGGACGGCGTTTCTGATGAGGAGCAACTG

TCACCGTTATCTCGAAAACCAGTTTTGCTTACGTGTCAGTAG

>EVM0049755

ATGGCATCTTTTGATGAAGCTTCAACTTTGGAACTCATTAGGCAACACCTCTTGACTGAC

TTTACTTCCACGGACAGCTTCATCTCCGATCTCGATGATCTTTGCAGCACTTCCACCACC

AGCTCTACTGATGACTACTGCATCAAGACTGAATCGACAGAATTCGATCCCTTCGATCAA

CCGCAATTCAAGCTGCAAACTCAATCTAGCATTGAATCCAAGCCTCAATCCTCACCAAAG

TCTTCAACTTCTCTAAGCCAGAGAAAACCATCCATGATCAAGGACATAGCCATACCTCCA

CCAGCAACCTTGAACGTGACCCCACAAGTAGCACAGCCTGTGGTGAACAAGGCAGATATG

TCCAGTGAACAAGAAAGGCATTACCGGGGTGTTAGGCGTAGGCCTTGGGGAAAATACGCA

GCGGAGATCCGTGACCCTAACAGAAAAGGAGCTCGCGTTTGGCTTGGAACATTTGATACT

GCCATTGAAGCTGCGAAAGCATATGATAGTGCTGCTTTCAGTTTACGAGGAAGTAAGGCG

ATCTTGAATTTCCCTCTGGAAGCTGGCAAGTCGAATTCTCAACAGCCTGAACAATTATTG

GAAACCTCTTGCAAGAAAAGAAAGATCGAAGGAGTAGAGTGTTGCGTGGAAAGTACTAGT

CCGATCGCTAATAAGGTGGCGAAGAGGGAAACTTCTTCACCGGAAAGTGAGGTCAAAGCT

GTGGCTTCCGAACCATTAACGCCGTCAAGTTGGAAGGGGTTTTGGGATGGTGAAGTCACG

GGGATATTTAGTGTGCCACCGTTATCGCCGTTATCTCCACATCCTTCATTTGGATACTCA

AGGCTCATGGTCGTATGA

>EVM0006603

ATGAACGAGATTAGCTCACTGTGCGAGTTTAGTGCTCTGTATCATCATCATCAACATAGA

AGTCCGAGCTTTAATTCTCTTATTTCTTGCTTAAACGAGACATGGGGTGACTTGCCGTTA

AAAGTTGATGATTCTGAAGATATGGTCATTTACAATTTCCTCAGAGATGCTGTTAGTTCT

GGATGGTCTCCAGTAGACTTGAGCACTTCAACCGCTACTTCTTCTTCTTCTACTACTACT

ACTACTACTACGACGACGACGACGACGACGACGATATTAGCTAATGTTGTAAAAGCCGAG

CCGAAAGACGAGCCGGATCTGGAAACCGATCCCATTAAATTGGTTCAAGAATCTAGCTTA

AATATGGAAGCTGTTGTTACTAAGAAGGTGGTGACCAAGGGAAGACACTATAGAGGAGTG

AGGCAAAGGCCGTGGGGGAAGTTTGCGGCTGAGACAAGGGACCCAGCTAAGAATGGCGCG

AGAGTGTGGCTCGGGACTTACGAGACGGCAAAGGAAGCGGCTTTGGCTTATGATAGAGCG

GCTTATAGAATGCGTGGCTCAAAGGCTTTACTTAATTTTCCACATAAGATAGGGTCTAAT

GAGCCGGATCCGGTTAGGATCACTGCCAAGCGTCGACAGCCAGATACAGGTCTGACGGCT

GTGGATACTGGCTCGGCTAAGATGAGAAAAATGTTGGTGGTTGAGGGAGCTGAGTTGGAA

AGGGGGGTGGGATATAATATGTTTCAGGTTGGTCCTCACAAGGGGTTAATGCCAGTTGGC

GAGCAATTATTGGCTAGTTAG

>EVM0024690

ATGGTGAAGAGCGAGCACAAAATCCAGTCAGAGGCTTCAAAACCAATGGCAATATCGTCA

ACGCCTTCGCCATCAGCGTGCAAGAAGAAGAAATACAAGGGAGTTAGAATGAGAAGTTGG

GGCTCATGGGTGTCAGAGATTAGGGCACCAAATCAAAAAACAAGAATATGGTTAGGCTCT

TATTCAACTCCTGAAGCTGCTGCTAGAGGCTCCGCAGCCAATCTCAACTTTCCTATTACC

TCCTTGCATTACATTCCTGACACTGTTATGTCCCCAAAGTCCATTCAAAGAGTGGCTGCC

GCTGCCGCTAATAGTTTTGTGGAAAATCCCACCACCCCACAAGCACCATCGCTTCCTCTT

CCTTGCTCCTCCCCCTCCTCGTCAATCTCATCACCCCCATCAGTGGTTTCCTCGCCGTCC

GATCAGCTTGATGATTATATGCCACTGTTGGAATCTTTTGAGACCAACAACGAACCAATT

TCAATGCTAGATTCTTGGTACAATTTTGATAGCTTACAATCTCCAAAGTACCTTGATCAA

ATGTTCAATGGGGTCTCGTTCAATCCACCAATGATCGATGATCTCTACGAAGGGGATCTT

CGTCTATGGAGCTTTGCTGAATAG

>EVM0050816

ATGGTGAGGAAGAGAAAGGCAGGTGAGGGTGAAGAAGAAAGCAATGATGGAAAAATGGGT

TGGGACCAGATGATGGAGGAGGCTGAATCACTCCATGGAGTTCGGAGAGCTCGAAAACGA

TACCTTGGGGTGAGGCAGCGGCCATCAGGCCGATGGGTGGCAGAGATAAAAGACACAATT

CAGAAGATTAGAGTGTGGCTGGGGACCTATGATACTGCTGAGGAAGCCGCAAGAGCTTAT

GACGAGGCAGCTTGCTTGCTCCGTGGAGCTAATACTCGAACAAATTTCTGGCCTTGCTCC

TCCCCGTCTTCGCATTCAAAGCCAGCTCTTCCCCCAAAGATTGTCAACATTCTGTTACTT

AGGCTTAAAGCTAGAGACAATTCATTGACACAGACCACTACTTTCCCTGTTAACCAACAG

GAGCAAGAAGCAAGAGAACAAGGAAACCAGGTCGAACATTTCTTCGAAATGCCTGGAGAT

ATCAGCATTGTTGAAAATACTGATACTGCTAATAGCACTGCCGATACTATGACTATCAGC

GATCACACGAGTGGTTGCTTTGAATCACGTTATATTGCAGAAGATCATCGGAGCACAAGC

ACATTTGAAGTGGATGATAATTGGAGTAATGTTGATGCCAAAGAAGAGATTGATACGGGG

CTGATTGATTTTCAATTCGATGATGCACTAGGATCATCTTCCTACCATTCTCCATTTGAC

ATCGCACAGGAGATGATGGAGCTAATGGAGCAGGAACATCATGGGGATGAGCCACCAATG

ATTAGAGAGATCATGAAGCGGTGGAAGCACGAACGGAAGTTCTCAGCTTCTCTTTATGCT

TATAATGGGGTATCCGAATGTTTAAGGCTGACATTCAGATCAGGAATGCAAGCAGAGGCA

AATGGTGGGTACGAGTTGCCATCTAACTTTGGAATCAATAGTAATAACGAGGAGGAAAAG

AACAGAGATGACACGGGGGGAAATGCCATAGAGGAGGTGCAAGAAGAAGTCGAGATTCCA

CAAAGACTAACAGAAAAGGGGTCCTCGTCTCCTTCCTCTTCTTTGAGCAAGGAAGGTGGT

GAATTGTCTCTTTGGAGCTGGCTCGATCTTCCTCCTGTCTGCTTCAATAATATTTAG

>EVM0025018

ATGGATTCAGTTAAGACTAACACTGGAAAATCCAAGAAGGGTGTTGTTGATGAAACTCAA

AGGCAGACAAAGGACCAAGATTGGCAGCTTGATAGAGGCAGAAAAGAAGCTGATATTAGC

TTTGAAAGGCGGCAATGGAAGCCTGTTTTTGGTGAAGCTTCCTTGTCGGATAGGCCTTCC

AAGAAGATCCGTAGCCCTGAACGTCAAGAACAAACTCAATCCTCTGCAAATTTAGCTCAT

CAATTACCATCTTTCTCCGTTTCTTCTAGCTCTGCCTCTACTCAGTCTTTATTCCCGCCT

TCTTCATCTTCAAGTTCAATACATCAGTTTCCTTTTGCCTTAGAAGGGTCTAATCAACCT

GTTCAGTTCCACCAACAATTTGGAACAAACCCTTCATTAACTATTCTTCGTCCACCATCT

CAGCAGCAAATGATTTCTTTTGCTCAAAACCAGCAATATGGCATTGGATATCCTCCGTTT

TTTACAGGAGAATCTGCATTGGCTAACCAGCAGCAGCAGCAGCTGTTTCAGTACTGGAAT

GACGCATTGAACTTAAGTCCGAGAGATAGGATGATGATGATGAACAGGTTGGGGCCAGAT

GGGAGGCCATTGTTTAGGCCACCGATTCAGCCTATAAATACAACAAAGCTTTATAGGGGA

GTGAGGCAAAGGCATTGGGGGAAATGGGTTGCCGAGATTCGTCTCCCTCGAAATAGGACT

CGCCTTTGGCTAGGCACATTTGACACAGCTGAAGATGCTGCATTAGCTTATGATCGCGAG

GCCTTCAAGTTGAGAGGAGAGAATGCTAGGCTGAATTTCCCTGAGCTTTTCCTCAACAAA

GATAAAGCAACTTCCACCGCTCCAAGTTCATCGGTTTCTTCTCTTCCGACCCCCAATCAA

AGTTCAATGCCAAAACAAGCCCGGGAAGGCCTTAACTTGCAGGTGGGAACCATGCCGCCG

CCACCACCACCACCAGAACAGCCCCAAGGAGACCATCCTGATGATGATTCCGGGCTGGGT

TCAAGTGGGGCCACTGTGAGTGATGAGGTTCAGGCAGTGGCAGAGGGGACCAGTGCAGGG

GAAGGCATTTCGGGGTCTCAGGAGCTGGTGTGGGGAGATATGGCAGAAGCTTGGTATAAT

GCTATTCAAGCAGGTTGGGGTCCAGGGAGTCCTGCGTGGGATGATTTGGATACCACTAAT

AATTATTTATTACAATCACACCTTCCTTTCGTTTCTCCAGATCAACAGCAGTTTAATGAT

TCTTCTGATCACCAGAGACAACAAGACATGGGCTCAGCTTCTTCTTCCTCCTTCCCCACG

AAGCCATTCTTTTGGAAGGATCAAGATTGA

>EVM0001609

ATGCGTTTTCTATCCCCTAATGAATTACAACCCATACTTATCTCCAACCCCACCATGTCT

GTTCAACCAAAAGTCAGATTTACTAAACATGTACTCGCCAAGAACCAGCAGCTGCATCCT

TCTTCAAGAACCCGCCAAAGACTTGTTAGAATCATCCACACGGACCCTGATGCCACTGAT

TCCTCCAGCGATGAAGAAGAGGAAGAGCAAGAGAGGAAGTTTGTCAAGATAGTGAAGAGA

CAAGTGAGTGAAATAAGCTTTTTAGAGCAAGACACAACAGCACCAGCAGCTCCTTCCTCC

GCAACTTCAAATGATGATCAGAAACCAAGAAGAAAAAGACCTTCTTCAAGATTACCGGTA

CCTGACGTCTCTCGTCAGAAAAAGTTCAGGGGGGTCCGTCAGAGACCCTGGGGAAAGTGG

GCTGCCGAGATCCGTGACCCGAACCGACAGAAACGTGTCTGGCTCGGCACTTTTGACACT

GCAGAGGAAGCTGCTACAGTATATGATCGCGCTGCGGTGAGACTAAAAGGACCCGACGCC

GTTACCAACTTTCGTACTAGCACTGCTACAGCGGAGAGAGCTAACGTTAACTCTGGTTCA

AGTGATGTGGAGTGTGAGTCATTTGATTCGCCGTCTAGCAGGGGTACCGGTACCGATTTG

GCATCGCCCACATTAGCCCCCCGTCAAATAGAGTTGACGCCGTTTGACCTTGTTTTAACG

GAGAAGGCTAAGGTTGCTGACGGTCAGCGCGAGTCATGTGAGTCGTCGTTAAGTTGGCTT

AACGACATGTTTATCACCAACGTCTGTTCTCCATTACGACGAGTTCACGCAGTTTGA

>EVM0008900

ATGGCACCCAGGAAGGATAATGATAATGGCAATTGTTCTTTGAAGAAAGCAAGTGGTGAT

CATGATAAGAAGGAAATCCATTACAGGGGAGTGAGGAAGAGGCCATGGGGGAGGTATGCT

GCTGAGATAAGGGATCCCAGGAAGAAAACCCGGGTCTGGTTGGGTACGTTTGATACGGCA

GTGGAGGCCGCAAGGGCCTATGATAAGGCGGCGCGTGAGTATCGTGGTGCTAAGGCGAAG

ACCAACTTTGCGTTAGCGGAGAAGGTGGTTAACTATAACGATGAGAAGCAGAGCTCTAGC

CAGAGCAGCTCGGTTGAGTCGTCTTGCTCCCCGGTGGTTTCTGCGGTGGCGCGTGATGTA

ACTCACCAGGTTGCAGCAGCAGCGAATGTTAACGCTGTTGGACCTGTCTGGTTTCTTGAG

AGTGTTAAGCCTGAGCTTGTGGCTCAGCGATTCCCTGTCCGTTTCGACCCGGTGGGTCTT

GAGGGCGGGGCCCAGAGTGACTCGGATTCATCATCTGTGATTGATTATAAGCCAAGGAGA

TCCATTCGTGATCTTGATCTTAACCTGCCTCCACCAGCTGATGCATGA

>EVM0045225

ATGGATTCTTCATATTCTCACTACCACAACTCGCAATTCTCCCCGGAGTCTTCCTCCTCC

TTTAATTCCCTTGATTCTTTCTCGTGTACCTCACAAAACTTCTACAGCCAGCCCCTTCCT

TTCAACGAGAACGATTCCCAAGAAATGCTTCTCTTACGAGTCCTCAATGAGAACCCCATC

AACTCTTTCGACACCGTATCATCCACCAATACTAACTATGACGAGGTGAGTTCAAGAGCT

GATTACCAGGGGGAACAGCCCCGAGAGATTGCTTACAGAGGGGTTAGAAGGCGGCCATGG

GGAAAGTATGCTGCCGAGATAAGAGATTCGACAAGAAATGGCGTGAGAGTTTGGCTAGGA

ACATTCGACACCGCCGAGGCGGCGGCCTTAGCTTATGATCAAGCTGCATTTGCAATGAGA

GGTTCGATGGCAGTTCTGAATTTTCCTGCCCAGAAAGTTTACGAGTCACTCAAAGAAATG

GGCTATGGTTTCCAAGAAGGGCAATCACCGATTCTAGCAATGAAAAAGAAACATATAATG

AACAGGAAAGCCGAGAGTAGGAAGAAGAAGGAGAGAGAAAGCAGAACGGAAATGGAAAAT

GTGGTGGTGCTGGAAGATTTAGGGGCTGATTACTTGGAAGAACTGCTAACCATTTCAGAG

AGTGCTAGTCCTTGGTGA

>EVM0029866

ATGGCACCCAGGAAGGATAATGATAATGGCAATTGTTCTTTGAAGAAAGCAAGTGGTGAT

CATGAAAAGAAGGAAATCCATTACAGGGGAGTGAGGAAGAGGCCATGGGGGAGGTATGCT

GCTGAGATAAGGGATCCCAGGAAGAAAACCCGGGTTTGGTTGGGTACGTTTGATACGGCA

GTGGAGGCCGCAAGGGCCTATGATAAGGCGGCGCGTGAGTATCGTGGTGCTAAGGCGAAG

ACCAACTTTGCGTTAGCGGAGAAGGTGTTTAACTATAACGATGAGAAGCAGAGCTCTAGC

CAGAGCAGCTCGGTTGAGTCGTCTTGCTCCCCGGTGGTTTCTGCGGTGGCGCGTGATGTA

ACTCACCAGGTTGGTGGGGTTATGGGGATGGGAAGGTTTCCTTTTGTGTTGCAGCAGCAG

CAGCCGAATGTTAACGCTGTTGGACCTGTCTGGTTTCTTGAGAGTGTTAAGCCTGAGCTT

GTGGCTCAGCGATTCCCTGTCCGTTTCGACCCGGTGGGTCTTGAGGGCGGGGCCCAGAGT

GACTCGGATTCATCATCTGTGATTGATTATAAGCCAAGGAGATCAATTCGTGATCTTGAT

CTTAACCTGCCTCCACCAGCTGATGCATGA

>EVM0041629

ATGGCTCCTGCAGAAAGACCTGACAACCACCACCTCAATAGCCCTAGATCGGAGATCCGT

TTTAGAGGCGTGAGAAAGAGACCATGGGGACGGTACGCCGCTGAGATCAGAGACCCGGGC

AAGAAAACAAGGGTTTGGCTTGGCACTTTTGATACTGCTGAAGAGGCCGCCCGTGCTTAC

GATGCGGCGGCTCGTGAATTCCGTGGAGCCAAAGCCAAAACTAATTTCCCTACAATTGGT

GAGCTTAACCCGAACCCCACGCGCAGTCCTAGCCAAAGCAGCACTGTCGAGTCCTCTCCT

CCGACCCCACGCGCCGCTTCCCCGCCGCCACTCGATCTCACTCTTAACATTTCCCACCGT

AATCCCGACCGCCAACCGTTTCCCAATGGAGTTAGGTTTACAGGTGGCGCGTGGTTCCCA

TTCCCTGCTGTTGCGCGTCCCGTCTACTTTTTTGACGCGTTTGCGAAGAGTAATACCCCC

AGCAATAGTAGTGTAGTGAATAATATTAACATGTGCAGGTTTGATCGAACGGTGATGGTG

AATGGAGGTGGGGGGCCGAGTGATTCGGATTCGTCATCAGTCGTTGATTTTGATCGTCAT

AATGACAGCAAGGGATTGTCACTTGATCTTAACCTGGCCCCACCACCGGAAGTCGCATGA

>EVM0050911

ATGAATTCTAACAACTGGCTTTCGTTTCCTCTTTCTCCCACTCACCCTTCCTTGCCTGCT

CATCTACATGCATCTCACCCTCATCAATTCTCTCTAGGGTTAGTCAATGATAGTATGGAA

AACCCATTTCAAACTCAAGAGTGGAGTCTTCTTAACACTCACCAAGGCAACAATGAAGTG

CCAAAGGTTGCAGACTTTCTTGGTGTTAGCAAATCTGAGAGTCAATCAGATCTTGTAGCC

TTCAATGAAATTCAAGCTAATGATTCTGACTATCTCTTTTCAAGCAATAGTCTAGTACCA

GTCCAAAATGCTGTTGTAGGCGCAAATAATACCTTCGAGTTTCAAGAAAATGCCAGCAAT

TTGCAGTCATTAACACTCTCTATGGGTAGTGCTAGTTGTAAAGGTTCGACATGTGAACCC

AGCGGTGATAGTAGCACTAATACTGTCGAAGCTGCTGCACCAAGAAGAACTTTGGATACA

TTTGGGCAAAGAACATCCATATATCGTGGTGTAACAAGGCATCGATGGACAGGAAGGTAT

GAAGCTCATTTATGGGATAATAGTTGCAGAAGAGAAGGTCAATCTAGGAAAGGAAGACAG

GTCTATTTAGGTGGCTATGACAAAGAAGAAAAGGCAGCTAGGGCTTATGATCTTGCTGCA

CTTAAGTACTGGGGAACATCCACCACTACCAATTTTCCAATCAGCAACTATGAGAAAGAA

ATAGAGGAAATGAAGCACATGACTAGGCAAGAATTTGTGGCCTCCATTAGAAGGAAGAGT

AGTGGCTTTTCTAGAGGTGCATCCATGTATCGTGGAGTTACAAGGCATCACCAGCATGGT

AGATGGCAAGCAAGGATAGGCAGAGTTGCAGGAAACAAAGATCTCTACTTGGGAACATTT

AGCACTGAGGAGGAGGCTGCAGAAGCTTATGACATAGCAGCAATAAAGTTTAGAGGGCTT

AATGCAGTGACTAATTTTGACATGAATCGATATGATGTGAAGAGCATTCTTGAAAGCAAT

ACTTTGCCAATTGGAGGAGGGGCAGCCAAACGGCTAAAGGAGGCTCAAGCAATTGAATCA

TCACGAAAAAGAGAAGAAATGATTGCTCTTGGATCAAGTTTTCCATATGGATCAAGTTCA

AGCTCTAGCAGGCTACAAGCTTACTCTCTAATGCAGACACCATTTGAGCAACCTCAACCT

TTACTTACTTTACAGAATCAAGACATTTCTCAGTATACTCAAGATTCCTCACTCCAACAA

AACTTTCTTCAAACTCAGCTTCATTTGCACCAGCAATCTACAGGATCTAATTTCCTTCAG

AACCAATCAAGCCAGAACCCTCAGTATTACAACAGTTATATTCAAAACAATCCAGCTTTA

CTTTATGGAATGTGGAACATGGGCTCTTCATCATCTGTAATGGAGAATAATGGCAGTTCT

AGTGGGAGCTATGGTACTGGAGGTTATCTGGGAAATGGGCTGGGAATTGCTTCCAATTCA

ACAGGGTCTAATGCAGTAGGATCAGCTGAGGAACTTGCACTTGTCAAAGTTGATTATGAT

ATGCCTCCTAGTGGCTATGGTAGCTGGTCTGGGGACTCAGTTCAGGGATCCAATCCAGGT

GTTTTCACAATGTGGAATGAGTGA

>EVM0043812

ATGCAAGATCAAGATGCATCGTCAAGCATGATTTCAAGTTCCAAAAGCCCCATCATCAAT

GTGGAGACTTTGGGATTTAGCACAGGCAACAAACTTGGAAGTCTTCATAGCAATTTTGGT

AATGTGGAAAAATTCAAAGGCATTGTGCCACAAAAAAATGGACATTGGGGTGCACAGATA

TACGCAAATCATCAAAGAATTTGGCTAGGAACATTCAAGACTGAGAAAGAAGCAGCCATG

GCCTATGATAGCGCTGCCATAAAGCTCAGAAGCACGGATTTGCACCGAAATTTTCCATGG

AACGACCACAATGTTCAAGAACCTAGTTTCCAAAACCAATATAACACAGAGAGGATTCTG

AACATGATTAGAGATGGATCTTATCAACAGCTTTTTGTGGATTTTCTAATGAAACAATCA

CGAAGAGAAGAAATTTGTGGAAGTACTAATGATCTCAATGGAAGGAGGGTGCATGTAGAT

GATGAACAATTTTCATGCATGCAACTTTTTCAAAAGGATCTAACACCAAGTGACGTAGGG

AAGCTTAATAGACTTGTGATACCCAAAAAATTTGCTGTCAAGTACTTCCCCTACATTTTT

AAAGATGCTGAAGATGATAGGGTACTAAATTCTGGGGTAGATGACACAGAGCTCATATTT

TATGATAGGTTCATGAAGTCTTGGAAATTTAGATATTGCTATTGGAGAAGTAGCCAAAGC

TTTGTTTTCACAAAGGGTTGGAATAAGTTTGTCAAGGAGAAAAAATTGAAGGAAAAGGAT

ATCATTGCATTTTATACATGCGCGTTCCCAGAGGTGCAAGAGCAACATGGTCAGGGCCAA

TATTTTACCATGATTGACGTAGTTTACTGCGATAAACAAAGTGGTAAGGTTGGTGGGTTT

AACCACATGGAGGTTATGCAAAGAGAATTGGCAGTGATTTTAAAGCAAAATATGAGAAAG

AAGCAAAAGGACGGGAAAGAATTGAAGGAAGAGAGAGTATTGAAAAAGAAGGAACTGAAA

AACAAGATTAAAGGAAAAGGGTTTAAGCTTTTTGGGGTGCAGATCAGTGATGTTTTTCAT

TTGGAAACAGAGGTTGCGTCGCAGCTCCAGCCTTCTTCCCAAACAGGTGCCGAGTGA

>EVM0015843

ATGGATGCCTCCTCGTCGAAAAACTTGGTTCAGAGACGAGAGCACGAACACAACGCCATA

GTATCAGCTCTCAAGCATGTAATCTCAGGCGGCACTGATCATGGCAGCACTACCGGCCCA

TTTGAATACCAGGACCAGCAGAATATCCTAGCTGCTGCTGCTGATGGTAATGCCGGTGAG

AGGAGCAACGTCATCTACGTTTCAGATTCCGAGACGTGTCTGGTTTGCAAATTAAGTGTC

TCTCAATGCCTTGGTTGCGTCTATTTCTCCGGTGGTGCTAATGATCAAGGAGGAGGAGGA

GGAGGAAATGAGGACACCACGGAGAAGAAAGGTAAAGGGAAGAAGAGGAAGAGCAAGTAT

CGGGGAGTCAGGCTACGACCGTGGGGGAAATGGGCGGCGGAGATAAGGGACCCGGCGCGA

GCGAGCCGTGTGTGGCTGGGCACGTTTGAGAATGCAGAAGATGCTGCGAGAGCTTATGAT

ACGAAAAATATAGAATTTCGTGGAATTAGAGCCATAACCAATTTCCCACTATCTGACTAC

CAAGTTCAAGAGACGGAGCAAGATAACCCAAACCAGACTGGCGAAGCAAACAATGCCGTA

GGGGAGACTAGTGGTGGCCGGTAG

>EVM0052615

ATGTGGAACCTAAATGACTCCCCTGATCAAGCAAGAGACGATGAATCCGAGGGATGCTCA

AGTCAAAAAACATCTATAGATGGCGACGATGACAAGGGCAAAAGGGTCGGATCCGTGTCG

AATTCCAGCTCTTCTGCTGTTGTGATTGAAGATGGATCAGAGGAAGAGGATGCTGTTGGA

GAGAAAGGAAATAAAATAATCAAGAAACGTAGTATAAGCTTTAGCAGCAGCAAGATTTTC

GGGTTCTCGGTGCCTTATGATCAAGACTCCGTGGACATGAGTGATCCACCAGTGACCCGG

CAGTTCTTTCCTTTAGAAGATCAAGAAATGGGGTCCACATCGGGCGGTGGAGGTAGTTTT

GGAGGTGGTGATGGAGTTGGAGGTGGATTTCCTCGGGCTCACTGGGTCGGTGTCAAGTTT

TGCCAATCGGAATCATCATTTGCTTCTCAAAAATCCATGGAAGTCTCACAACCATTGAAG

AAAAGTAGACGAGGACCGCGGTCCAGGAGCTCTCAATACCGTGGTGTTACCTTCTACCGG

AGGACTGGCCGATGGGAGTCTCATATATGGGATTGTGGGAAGCAGGTGTATCTAGGTGGA

TTTGACACTGCACATGCAGCTGCTCGTGCATATGATAGGGCAGCTATCAAGTTTCGGGGA

GTGGAGGCGGATATTAATTTTAGAATTGAAGATTATGAGGAGGACTTGAAACAGATGAGC

AACCTCACAAAGGAAGAATTTGTTCATGTACTTCGCCGGCAAAGCACAGGATTTCCTAGG

GGTAGCTCCAAGTATAGAGGTGTAACCTTGCACAAGTGTGGAAGATGGGAAGCTCGAATG

GGCCAATTCTTAGGCAAAAAGTATGTTTATCTGGGCTTGTTTGATACTGAGATTGAAGCT

GCAAGGGCCTATGACAAAGCTGCCATCAAGTGCAATGGCAAGGAGGCAGTAACCAACTTT

GATCCCAGCATTTATGCAAATGAGCTTAACTCAATTGAATCATCAGGTAATGCTGCGGAT

CATAGTCTTGATTTGAGCTTGGGCAATCCAGCTTCGAAGCAAAACAGTGTAGAATTTGGT

GAGGATAGGCACAATGTTGCAATGGATCGGGGAACGATGCCATCTGAACCTAGCTGGCAA

AATCAGGGGCTTAGACCCAAGCAGCTTAACCTTCATAGAAGTGACAATGATGGACATGGA

AGAGATGGGTATGGAGAGACTGAGACGACTCAGCTTCTTAGCAAAATCCACATTCAATCT

CCAGCATCGCTCAAGTCCAGTGAAATGCCAAGATATGAGCAGCAGTTCAGATCACACGGA

GATAGTCAGATGAATCCTTTTCTCCCTCCACAGTTTAACTCACCAAACTATCAAATTCAG

TATCCAAGCAGCAGCAATGGAGGCCGAATTGGAAGTGATCTCTCTCTCACCCCAGCTGAG

CTTCATTATCGTCATCATTATCAACAATGGCAAGCAGGTCCTCCCCAGTTTGCAAATGCT

GCAGCATCATCAGGATTCCAACAGCAAATTAGGACTCCACAAAACTGGCTGCAGAAAAAC

GGGTTCAACTCTCTCATGAGACCTTCCTGA

>EVM0050804

ATGGAAAGTGTTGTAGACAAGGATATGGCAACAAGAAAGAGAGGAGGGATAGAAAGTGAG

AGGCAATACAAGGGGATAAGGATGAGGAAGTGGGGAAAATGGGTGGCTGAGATAAGAGAG

CCTAACAAAAGGTCAAGGATTTGGCTTGGTTCCTACTCAACACCAGTAGCGGCGGCTCGT

GCTTATGACACCGCCGTTTTTTATTTGAGAGGGCCGTCGGCGAGGCTCAACTTTCCTGAA

TTTTTGGCTGGAGAGGATTTTGGCGGTGGTGGATCATGCGGGGACATGTCAGCTGCTTCT

ATAAGAAAAAGAGCAACTGAGGTTGGAGCTCATGTTGACGCTATTGAGACAGCGTTAAAC

CACCACCACCTCCATCACGATGACCGTCAAAGAAACAGCAATAATAGTAGCAGTAGTAAT

GATAATAACGAAACGGCCGTTGACAGTAGAGAGTTGAAGCCACGGCCGGTTGACTTGAAT

AAGGTGCCTGACCCAGAGGATTCCGATGGAGATGAGTGGAAGAGGAGTTAA

>EVM0017086

ATGGATGCATCCATCTTTCACTCTTCAAATTCTGATTTCTCACCGGAATCTTCTTTTGGA

TCACCGGACTCTTTTTATAGCCAATCTCTTAACCAAAGCTCTCTCCCGTTCAATGAAAAT

GACTCGGATGAAATGCTTCTTTTTGAACTAATCTCGGAGGGCACTCAAGAAACTTCTAGA

GCAACTTCCTATAATGGGATTGTTAAGGAAGAAGAGGTTAGTTCAGTTGCCAAAAAAAAA

CCCGACAGGGAAAAGTCCTACAGGGGTGTTCGGAGGCGGCCATGGGGCAAATTCGCGGCT

GAGATACGGGATTCCACAAGGAATGGGATACGGGTATGGCTAGGCACGTTTGACAGTGCA

GAAGCAGCTGCTTTGGCTTATGATCAGGCTGCTTTTTCGATGAGAGGACCTGGGACTATA

CTAAATTTCCCAGTTGAAAGAGTGAGGGAGTCACTTAAGGACATGAAGTGTAGTCGTGAT

GAGGATGGTTGCTCAGCTGTGGTGGCTCTAAAGAGAAAGTACTCCATGAGAAGAAAACTT

GGAAGCAGGGGAAAGAAAGAGAGGGATATGAGGATAGAGAATGTACTGGTTCTGGAAGAT

CTAGGCGCTGATTATCTGGATCAACTGTTGAATCGATAA

>EVM0040715

ATGGAAGAGCCTGACAAAGGGAGGGAAAAGGAGTTGAAAGGGAGAGAAGAGGTTCGATAC

CGGGGTGTTCGGAGGAGACCTTGGGGAAAATTTGCGGCTGAAATACGAGACCCATCAAGG

CAGGGAGCAAGGTTGTGGTTAGGGACATTTGATACGGCAGAGGAGGCAGCTCGTGCTTAT

GATAGAGCTGCATTTAGCATGAGAGGTCATCTTGCAATCCTCAACTTTCCTAATGAATAT

CCCTCTCATGCGACGGGGACCTCTTCTCGTCGTCCACAAGTTTCATTATCCAGTAGTGAT

GCTGGTGCAAGCGAGAGTTTTGAAAGAGGAAGCTCATCACGTGCCGGACAAGGACAGCAA

GTTATCGAGTTTGAGTACTTGGATGATAAGATTTTGGAGGAGCTTCTTGAAACAGAAGAG

GAGAAGAAGAAACGGCAGCAAGATTGA

>EVM0029622

ATGGACGAGGACAAAACAAAGAGCAAGGATGTAGGAGGAGAGGTCAAGTACAGAGGTGTC

AGGAGGAGGCCATGGGGAAAATTCGCTGCGGAGATACGTGATTCAGCCAGGCATGGGGCG

AGGGTGTGGCTGGGGACTTTTAACACGGCAGAGGAGGCAGCAAGAGCCTATGACAGAGCA

GCTTATGCAATGAGAGGGCACCTAGCCGTTCTCAATTTTCCTAATGAGTACCCAAACATG

GCCGGTAATTCTAGCGTTGGTTCCAGAAGCTCTACTCCTTTTTCCTCTTCAGGTTATCCA

GGTTCTTCTTCCTCTTCATCATCAATGAAGCAAGAAGTTTTTGAGTTCGAATGCTTGGAT

GATAAGTTGCTTGAGGAGATGCTTGAGCAAGAAGAGAAAAGGAATAAAAAAACGTAA

>EVM0021270

ATGCACAAGAAACTGGCAGCTTGTAGTAGTTTTCATGGAATGGCATCAGGCTCGAAATAC

TTTGGTGATCCAACCACCTGGGGTCGCGTCGAGGATGGAAAAGGTGGCATGAAATCGCCG

TCGCGATCGTCGTCGGAAAGATTATTTTCGAGTTCCGAATCTTGTTCTTCGGCAGATGAT

CGGGTTTCAGGTACCAATTTGATCGGAAACATCCCTGGAATTTTTTTTATGTCAGGATTG

AATTCTATCAACGCTCATGGAGCTAATTCAATATCTGATCAAACTATCAAAGAAAGCTCC

ATTCCTGTGAATTTCTTGGAATCTTTTCCAACATTAAATAACCAAGCTCAACTACCTGAG

CCTCCTTCTCCATCTTCGTTATCAAACACATCAAACCCCCCAAATTTAACCTTGTTTTTG

CAGGAACCAGCAGCAGCTTCGTTGCTAGATCCGTCCAAACAAGTTCTCGATCCACCTTGC

AAGGATCAAAGATGTGAACCTGCCATGTCATTGTTTCCAAACATTTCATTTTCCATGCCT

CAACTCGACCAAATTCATTTCCAACCGAGTAATGAATGGTTCAAAATCAACCAGAAGTTG

GCAAATTATTCCACCAAAGGGTTTAACGACTATTGGCTTAGCACGACGAAGACACAGCCG

ATGAAATACACGGGCAGAACACTGCAGAACCACCACCAGAAGCCTTCTTTTTCATCGGCA

TCAACCTCGCCGGGAAAGCTGTTCCGAGGAGTGAGGCAACGGCACTGGGGAAAATGGGTT

GCAGAGATTAGGTTACCGAGAAACCGGACGAGGGTCTGGTTAGGGACCTTTGACACAGCA

GAAGAAGCCGCCATTGCTTACGACACTGCAGCGTACATGTTACGTGGAGACTACGCGCAT

TTGAATTTCCCAGATCTTAAGCATCAACTGAAGTCTAATTCATTGAACAGAACCACAGCT

GCCCTTCTTCAATCAAAGCTACAAGCAATTTCACAAGGCAACAACATTTCCGGCGATCAA

AATAAGCATATTAGCGATCATCCACCACCGCCATCACCAAACAAGATTAATGTGCATGAT

TCAGATGCTAAAAAACTCAAAGGTCCGAGCCAGAATACAGCAAGAAAAGAGCGGCAATTT

GAGGTAGGAGGCAAAACTGGAGGTGAAGTGATGGAGAACAGGAAAGGTCAAGAGGTTGTA

GCATCGGATATTGACGCTGTTCAGCTAAGTAGAATGCCTTCCTTGGACATGGACATGATC

TGGGATGCTCTTTTAGTCTCTGATTCATGA

>EVM0004107

ATGGTACAATCAAAGAAATTCAGAGGCGTCAGGCAACGCCAGTGGGGCTCTTGGGTGTCT

GAAATTCGCCACCCGTTACTGAAGAGGAGGGTGTGGCTGGGGACATTTGAGACGGCTGAG

GCAGCCGCAAGAGCGTACGATCAAGCAGCTATATTGATGAACGGACAGAATGCCAAGACC

AATTTTCCGGCAAGTCACCTTGATCAAGACATAAATCTCGGCAAAGATAACAACTCTCCC

TTGTCTGCCAAGGAACTGGCTGAATTACTGAACTCGAAGTTAAGAAAGTGTTGCGGCAAG

GATCCTTCTCCTTCACTCACTTGCCTGAGGCTTGATAATGACAACTCTCGTATCGGTGTG

TGGCAAAAAAAAGCTGGTTCTCGTTCAAGTTCAGACTGGGTCATGAAGGTGGAGCTTGGA

AATTACAATAAGAAGACAGAGCCACCGTCGAAGGCTGAGATTGAGCCGGAAAAAGGTACG

GAGGAAGAGGATAAAATTGCTATGCAGATGATAGAAGAGCTACTTAATTGGAATTGA

>EVM0008274

ATGCAAGCTGCTATCTGTTTAGGCACCGAAGGGATTTTGGACGAAAACTTCTCTGAAGCA

ATAGTGATAGGTAACAAAGAAGAGAATGCAATGGGCAGTGATTTGGGTTTATCGACACAA

CCGATCGATTTCTCGCAGATACCCAACCATGCCAGGGAAGTAGGAGCCATGGCACAAATG

ACTCGTGATCAAGATGTTGCCAACTTTAATAAGTCAAGAAATGACTTGACGAGAGGCAGC

AAATCTAGATTTCGTGGAGTTGCAAGGCATGAAGGCACTAGCAAATGGCAAGCTTGGACT

CCAACGGGTGAACTTATCTTGAACATTTTTGAGACGGAAGAGGAAGCAGCAATTGCGGTC

GACCTAGCATGCATAAAGCAGAAAGGATACCAAGCAAACACCAACTACGACATAAGATAC

TATAATGTTCAGGCAATGATCAATGGCAGAAAACCCGAGCTGCCACAAATGGAGGTGGAA

ACACCATGTCCAGTGACAGCAGCACCGGCGCAAGTTTATCGTCACAGGATAAACACAGAC

GGTGCTGGATACAGCACCACCATGCAGCCTACAATCTACCGAGCTCGAGGGTTTCGCCCA

GCAAAAGATCTACCAAACTTTGCTGCGATCTTGGGTCTTGTGAAGCAGGATAATGGATCA

GGGTTAAGGTTCAAACATGGAGATTTTAGCCCTTTTAACTTCTACCAGAGGCAACAGGTG

AAAAAACCAGCAGCTCGAAACAAAAACCCTAACTCCGTCAACTTTGAGAGTGAGCAGATG

CTGAATACTGCAGGACGACTAGGGTTTAAACATTCAAGCACAAGCCGTTTCAAGGTTTAC

AGGAGGAACGCCATGTGGAAAGGGAAAGCGAAGGTGAGCTTCTGA

>EVM0049140

ATGGCTCCCAGAGAAAAACCTAACCACAGCAGCCCAAATATCCTCGGATCAGAGATCCGT

TACAGAGGCGTCAGGAAGAGACCATGGGGACGTTACGCGGCTGAGATCAGAGACCCTGGC

AAGAAAACGAGGGTGTGGCTTGGCACTTTCGATACCGCTGAAGAGGCCGCGCGTGCATAT

GATGCGGCAGCTCGCGAGTTTCGCGGAGCCAAAGCCAAAACTAATTTCCCTACAATCGGT

GAGCTCATTCCCGCCTCAACTCGCAGTCCAGCCAAAGCAGCACTGTTGAGTCCTCCTCCC

CTCCGCACCACGCGCCGCTTCTCCCCCTCCACAACTCGATCTCACTCTTAA

>EVM0002622

ATGCCACCAAAAAGACAACCTTTGCTTAACACTAAGTCCGGCAAAAAACCCAACGATACT

AAACGGATCTTGTCGCACGAGTTAAAAATGGAGTCCACTATGAGAAAAATCCGTATCATC

TGTTATGATCCGTATGCTACCGAATCCGATTCGAGTGATGATGAATCTGACAAGAAATCG

TTTAGAAAACGTTTTGTGCGAGAAATCAATCTTCCTTTGGTTGTAACCCCCCAGCCAAAA

ACCTTTGAGCCTGAGAGTTCCTGTCAAGAAAGTAATAATAGTGCTAAAACCCCGGGTCAG

AAGAGGAGGGTTTTGGCCGAACCCGGTAAGAAGAAAAGGGTTTTGTCCGAAACCCCAACA

TCAACATTAGAAGGTAAGAACTTAGCACCAAAGAAACCTGTTGGTGTTAGGCAGAGGAAA

TGGGGTAAATGGGCAGCTGAGATTCGGAATCCAGTGACCAAAGCTAGGACGTGGCTGGGT

ACTTTCGATACTCTTGAAGAAGCTGCCCAAGCCTATGAGGCAAAGAAGCAGGAATACGAG

GTTTTGGCTATGTTAGCCTCTCAAAAAAGTCAAAACGTTTCATCGTCTCTGGCCATCTCA

CAATCTCATTCTCACAACAGCAGCAAAAAACAACTCAGTTCTGCATCTTCCGAGGATACA

GACGATAGCATTGTTTCGCATACTTCCCCTGCTTCTGTTCTTGAGCTTGACGCATCTGTG

CTAAATGTTACCGGTGAATGTGCTCTTATGATCAAGGATGAAGACGTTGGTTCTTCTGTT

GCGAATCTTGAGATACCGGATTTAGAGTTTTTGAACGACCCACTGGCATCTTGCTCAATA

GATCAAGATCTTAACTTGAGTCTCGACCTTGGTAATCTTATTGATGAGTTTGGTCGATTT

TATGATGATTACTGTGGTATTGATGACCTTGCTATTTGCGGGCTAAATGGTGAAGAACCA

GGTGAGCTTCCTGATTATGATTTTGAGTTTGGCAACGAGGAATTCAATTATTTGGATGAT

CATCAACAGAAGCCCCCCAACATTGCATGCCTATAA

>EVM0024408

ATGGAAATTCATTTCCAACAACAAAAGCAGCATAAACATGCAAGCATTCCAGTTAGCAAA

GCAAGCAAACTCAAGGGGAGAAGTATGCCGAACAACAGCAGTAACAGGTTTGTTGGTGTT

AGGCAAAGACCTTCTGGTAGATGGGTGGCTGAGATCAAAGACACGACTCAAAAGATAAGG

ATGTGGCTAGGAACCTTCGAGACAGCTGAAGAAGCTGCACGAGCTTACGATGAAGCTGCT

TGCCTGCTTCGTGGATCCAACACTCGAACCAACTTCACCACTCATGTGTCCTTGGATTCT

CCTCTTGCTTCGCGGATTCGAAATCTCCTCAAAAGCAAAAGGAGAGACAAACAACAACAG

AATGAAGAAGAAGCTGCTGAAGTCTCTACTCCACCAACTAGTACCATTACTACCACAAGC

AGTTCTATCACCACTAGCAGCAGCAGCAGTTCTGGCATGAGTGGTAATTACTCTCATTCC

AGTTCAATTCATCAAACGGATCATTTGTTTGAGGATGCATATAAACCGGATTTGAGCAAC

TGCGTTGAGGAATTCAAGTTGGGTTCTTCCCAGTCTGATCTTTCATGGGGTTTTGGGCCT

GTATTCGAGCGGTTTTCATGTGCGCAGGAAGTATTGGATTTTCCAGAAAGTGTGGTATTA

CCCGAGGCAAGTGACTTGGGGTTCTCAGCATTCGATAGGATGAAGGTGGAGAGACAGATA

TCAGCATCTCTATACGCAATTAATGGGGTGCAAGAGTGCATGGATGCTGTCTATGATCCT

ATTGAAGCTTTGTGGGATATTCCACCACCATAA

>EVM0009993

ATGAAATCCATGAGTAATAATGACAATGGAAGTAGTAATAATAACTTGTTAGGCTTCTCT

CTCTCGCCCCACATGAAAATGGAGGTTGCTTCTGACCTCCAACACCATCATCAGTATCAT

CATCAAACCCAAACCCAAACCCAAACACATGCTGCTGCTGCTGCTGCTGCTGCTGCTGCT

GCTCCAACAAGTTTCTATCTATCTAACTCTCTCTTCAACAGTTCTGAAATCTGTTACGGA

GTTGGAGAAAATGGTGGCTTTCACTCTCCTTTGTCTGTTATGCCCCTCAAGTCTGACGGG

TCTCTTTGTATCATGGAAGCTCTCACTAGATCACAACCTGAAGGAATGGTGTCGAGTCCG

TCACCAAAACTTGAAGACTTCCTAGGTGGTGCAACCATGGGAAGTCATCAGTACCGTAGC

CATGAAAGGGAAGCCATGGCCTTAAGTCTGGACCGCGTATATTACCAGCAAAGCTCAGAG

CCAGAAACTAACAGACAACATTCTCTCAACCTTCACGAACCATATAGGCAGCAAGAACAG

CAGTTTTCAGTTCAAACCCATCCATACTATTCTGGAATAGCATGCCCAGGATTGTACCAG

GCCTCACTGGAGGGAGAAACCAAGGGTACTCAGCTTGCAGACTGTAATTCACCTATTCCT

CAAATGGCCGACGATGAGCTGCCTTGCTTGAAAAACTGGGTTGCTAGACATTATTCCTCG

CAAAATGCACTTGAGCAGCAGATTAATAGTGGCATGGTTGATGATGGTGGGGCTTCTTGT

TCTGCTAGTGCGATGGGTTGTGGGGATTTACAGTCTCTCAGCTTGTCTATGAGCCCTGGT

TCGCAGTCAAGCTGCATTAAAGCTCCCAGGCAGATCTCACCTGCTGGCACTGAATGTGTG

ACCATAGAAACAAAGAAGAGAGGCCCTGTAAAAGTGGGTCAAAAACAGCCTGTTCATAGG

AAGTCTATTGACACATTTGGCCAAAGAACGTCACAGTATAGAGGTGTTACAAGACACAGG

TGGACTGGTAGATATGAAGCCCATCTCTGGGACAATAGTTGTCAAAAGGAAGGCCAGACC

AGGAAAGGAAGGCAAGGGGGTTATGATATGGAAGAAAAAGCTGCAAGGGCTTATGATCTT

GCAGCCCTTAAGTACTGGGGATCTTCAACTCATATAAATTTCCCGTTAGAAAATTACCAG

GGAGAACTTGAAGAAATGAAAAACATGAGCCGACAGGAATATGTTGCACATTTACGGAGA

AAAAGTAGTGGGTTCTCTAGGGGGGCCTCAATCTACAGAGGAGTAACAAGGCATCACCAG

CATGGAAGATGGCAAGCAAGGATAGGCAGGGTTGCAGGAAACAAGGACCTTTATCTCGGG

ACGTTCAGCACCCAAGAGGAAGCTGCTGAAGCTTATGATATTGCTGCGATCAAATTTCGC

GGGGTTAATGCTGTAACAAACTTTAACATAACAAGATACGATGTTGATAGGATCATGGCC

AGCAACACCCTACTAGCCGGAGAACTAGCCAGGCGAAACAGAGATACAGAATCTAGCATT

GAGGCCAATGATTATAACACATCAACACAGAACAATGGTGTTTTACACCAGTCTTCTCCG

CGGGAGCAGCCAAATCTCTGTGGAGAATGTCTTGATCAGAAGTCACTGAGTGTAGGGAAT

TATCGAAGCACCTCTTTCTCAGTGGCAGTGCAAGATCTTATGGGTATGGATCATTCAGTG

AACTCAAGCCAACCAGTGGTAGATGAATCATCCAAGCTAGGAACTCATCTCTCGAACCCC

TCGTCTTTGGTGACCAGTTTGAGCAGCTCTAGAGAAGCTAGCCCTGACAAGACTGGAACC

CCCATGCTCTTTGCTAAACCTCCACTGGCATCGAAGTTCATTGGCCCTACAGACAACGTT

ACTCCTTGGATCCAAGCAGCAGCCCAATTGAGGCCACCAGCAATCTCCATGGCTCACTTG

CCAGTCTTCGCTGCATGGAATGATACCTAA

>EVM0006689

ATGGCCTCCATGAATAACTGGTTAGGTTTCTCTTTATCCCATCAAGAACTTCCTTCATCA

CAATCTGACCACTCTCAAAACACAGACTCTCGCCTTGGTTTCCACAGTGATGTAATCTCT

GGTACCGATGTCTCTGGCGAGTGCTTTGATCTGACTTCCGATTCCACTGCTCCTTCTCTA

AACCTTCCCGCTACTTTTGGTATACTTGAAGCCTTTAGAAACAATCAAACTCAAGATTGG

AATATGAAGAGTTTAGGCATGAATCCAGACACTGACTACAAAACCGCCTCATGCCTCCCT

ATATTCATGGGTACTTCATGCAATAGCCAAAACATTGATCAGAATCAAGAACCTAAACTC

GAGAACTTCCTTGGTGGCCATTCTTTTGGCAACCATGAACACAAACTGAATGGCTGCAAC

ACCATGTATGATACCACTGGAGAGTATGTATTCCAAAACTGCTCTTTGCAACTTCCATCT

GAGGCGACATCAAATGAAAGAATCAGGAGCAATGGAGGAGGTGAAAATAAAAATAGTTCC

ATTGGGTTATCCATGATCAAGACTTGGTTAAGGAACCAGCCAGCACCGACCCAACAAGAC

ACCAATAATAAAAACAATGGTGGTGCACAAAGTTTGTCCCTTTCAATGAGTACTGGGTCG

CAATCAGCCGCTTCTTCTTTGCCACTTCTAGCAGTAACTGGAGGAGGTAATAATACTGGG

GGAGATCACAGTTCTTCTTCTGATAATAACAAGGAACAAAAGACCACACCAAGTCTTGAT

AGCCAAACCGTTGCCCTCGAAGCGGTGCCAAGGAAATCTATTGATACTTTTGGCCAAAGA

ACATCTATATACCGTGGTGTAACAAGACATAGATGGACTGGTAGATACGAAGCTCATCTA

TGGGACAATAGCTGTAGAAGAGAAGGACAAACTCGGAAGGGAAGGCAAGGAGGTTATGAC

AAAGAAGATAAGGCAGCTAGAGCCTATGACTTAGCAGCATTGAAATACTGGGGCACGACT

ACTACGACAAATTTTCCAATTAGCAACTACGAAAAAGAGATAGAAGAAATGAAGCACATG

ACAAGGCAGGAGTACGTTGCGTCTCTTCGAAGAAAAAGTAGTGGGTTTTCTCGGGGTGCT

TCTATATATCGAGGTGTAACGAGACATCATCAGCACGGAAGATGGCAGGCAAGGATTGGA

AGAGTTGCGGGGAACAAAGACCTTTACTTGGGAACTTTCAGCACCCAAGAGGAAGCGGCA

GAGGCCTATGACATTGCGGCCATAAAATTCCGTGGGCTGAATGCAGTGACCAACTTTGAC

ATGAGCAGATATGATGTCAGCAGCATACTCGAGAGCAGCACATTGCCAATTGGAGGCGCG

GCTAAGCGGTTGAAAGAGGCGGAGCATGCTGAAATAACAATGGATATTGCGCAAAGAACA

GATGATCATGACAACTTGAGCTCACAACTCACTGATGGAGCTGGCAACTATGGTGCAGTA

CACCATGGCTGGCCTACTGCTGCATTTCAGCAGGCACAGACTTTTAGCATGCACTATCCA

TATGGACAGAGGCTTTGGTGCAAGCAGGAACAGGACTCTGACAATCGCAGCTTTCAAGAG

CTTCATCAACTACAGTTGGGAAATACCCACAATTTATTTCAGCCTTCCGTTTTACATAAC

CTAATGAGCATGGATTCTTCTTCAATGGAACATAGCTCTGGTTCTAATTCTGTTATGTAT

AGCAGTGGAGTTAATGATGGTACAAGTACGGGAACCAATGGAGGCTATCAGGGAATTGGT

TATGGAAGCAATGTTGGGTATTCCGCCCCGATAACTACGGTTATATCTAATAATGAGAAC

AACCAAAATCAAGGAAATGGTTATGGAGATGGAGAGGTGAAGGCTCTGGGGTATGAAAAT

ATGTTATCCTCGTCTGATCCATATCATGCCAGAAACCTGTATTATCTTTCACAGCAATCT

CCCGCCGGTGGGATCAAGGCTAGTGCATACGATCAGGGCTCAGCATGTTACAACTGGGTG

CCAACAGCTGTTCCTACCGTCGCAGCAAGGTCTAACAACATGGCTGTTTGTCATGGAGCA

CAACCTTTCACTGTATGGAATGATGGTACATAA

>EVM0018103

ATGGGATCAGAATTGTTGGGCTCTGAGATTGAGAATATGGGTACTCTAGATCAAGATTCT

AAAGCTAATTCTATGTCCACGGATTCTACTAAGAAGAGGAAAAGGGTGAGCAATAAATCT

GTAGCAGAGACCCTTAAAAAGTGGAAGAAGTATAATGAGTATCTTGATTCTCAAGGTGAC

GGAGGTAATAAACCAGTACGTAAAGTTCCTGCCAAGGGCTCAAAGAAGGGGTGCATGAAA

GGTAAAGGAGGACCAGAGAATTCAGTCTGCAATTACAGAGGTGTGAGACAGAGGACATGG

GGGAAGTGGGTTGCTGAGATTCGGGAACCAAACAGAGGCCCTAGGCTATGGCTTGGTACT

TTTCCTACTGCCTACAAAGCTGCTCTTGCCTATGATGAAGCTGCACGAGCCATGTATGGT

CCGTGTGCTCGTTTAAACCATCCTGATGTGCTGAATTCAACAAGCTCATCGAAGGACAAC

TTCTCCCCAGCAACACCATCTTGCTGTTCTCCAGCAGCGAGTTCTGCAGACTCTGCTACT

ACTTCAACCCACTCCGAGGTGTGCGTGTATGAGGATCCTAATCAGAATGTATCCAGCCAA

GCTGAGGTTCTTGCGCATCATATATCAAGCCAACAACATATTGAGGATGGTTCGCAGGGA

GTTGATATATCAAGCCAACAACATATTGAGGATGGTCCGCAGGGAGTTGATATATCAAGC

CAGCAACATATTGTGGATGGTTCGCAGGGAGTTTATATATCAAGCCAACAACATATTGTG

GATGGTTCGCAGGGAGTTGAAAATAGCACACTGAGGGACGAGCTGAGGAGTCAATCAGAT

AATCCTTTGTGGACTCCTTCAGAAGAGCCCTTGTGGACCAATGACTGGAACAACTACTCG

TTGGATGAAATATTTAGCTTTGATGAGCTGCTAGGCGATATTGATGCGGGAATGATGGGG

GCAGAAGGGTACTTCAACTTGGGATTTTGA

>EVM0025370

ATGGACAATTCATCTCTCTCTCATCCTCCCCAAGAACCCACCACCACCACCACCACAATA

CCATCCAATGACAAAAGCACCGATAACAATACCACCGCAACCACCCCCACTACCGCCACA

ACAAGTGACACAAACAGTAACAGCAGCAGCAGTGGCTGTAGCAGGAAGTGCAAAGGCAAA

GGAGGACCAGACAACGGTAAATTTAGATACAGAGGAGTCAGGCAAAGAAGCTGGGGAAAA

TGGGTAGCGGAGATCCGTGAGCCAAGAAAACGAACCCGTAAGTGGCTTGGAACTTTCGCC

ACCGCAGAGGACGCGGCACGAGCTTATGATCGAGCAGCCATCATCCTTTATGGCTCTAGG

GCTCAACTCAACCTGCAACCCTCAGCTTCCTCATCTTCTGCACAGTCCGGATCAACTTCT

CGCAACTCTACCTCTTCCTCGAGCCAGACTCTTCGCCCTTTGCTCCCTCGTCCCCCTGGG

TTTGGCCGTGGCTTTAGTTTCACTTTCTCTCTCTCAAATCCAATGGCTTCTCCGGCTGTC

ACGGCAGCTTCGTCGGGATTTACTCCATACGAGGTTAATTGTCTTTCGAATAATGTCGTT

GGATCAGCCTTACAGTGTGCTAGTGCTAATGAAATGCCAGGGCAAAATCACGAGCAAGTA

ATGTTACAACGCTATCTCTTTCAACATGGGGCTAATACAACCAACCCCAATAATATATTT

GTTAGCTCTAGTGTAGCGACGGCAACAACAACCTCGTATCAAAATCATTGTAATATTCTG

GCGCAGCATCATGCATACGATGATGTTAATGCGTTGGTGGGTTCCGACGGGTCGAGTTTT

TCTCTGTCTGGTAGCAATACTCCTGTTGTTGCACCAGAGGGTCATCTTCTGCAGGATCCG

GCAATGTATGTTGGACCTGGATCTCCATCTGTGTGGAATGATGAGGAGTACCCGCCTCCT

AGTATTTGGGACGATGAGGACCCTTTCTTGTTTGATTTTTGA

>EVM0036052

ATGTTGGATCTCAATCTTGGTATCACCTCTAGCGATTCATGTTGTGACGATAACAACAAG

AGCAATATGATGGTAGTTGTTGATGCTGGGTACATCCAGGAAAAAGAAGCAGCTTCAAGG

AATCAACAAGTGGAAGATTCAGCTACCTCAAACTCCTCCATTACCAACACTACCGAAGAT

GAGAATTCCTCAAACAACAGTAACTCTGCTTTCATTTTTGATATCTTGAAGAAAGATGGA

AACTTTACCAACACAGGGACCATTAATGCTACAAAAGAAACAAACCCAAACTGTGATTTC

ACAACTCAACAACTTTTTCCTGGAAGCACCGGGCTGGGGCTGAATTTCCAGCCAGGATTG

GCTGTAACATCGGCTACAAGACCTCAGTGGTTGAAGCTTTCACAGATGGGGTCAAGTCCG

GAGGCAGAGCTGAATGTGCAGCAAAAACAGCAGCAAGCGAGGAAGAGTAGGAGAGGACCA

AGGTCTAGAAGCTCACAGTATAGAGGAGTGACGTTTTACAGGAGAACGGGGAGATGGGAG

TCACATATATGGGATTGTGGGAAGCAAGTATATTTAGGTGGATTTGACACTGCTCATGCT

GCAGCACGGGCATATGATCGAGCTGCAATTAAGTTTCGGGGAGTTGACGCTGACATCAAC

TTTAATTTAAGTGATTATGAAGAAGATATGAAGCAGATGAAACACCTTAGTAAAGAAGAA

TTTGTTCACATTCTTCGCCGTCAAAGCAACGGTTTCTCACGAGGTAGCCCAAAATACAGA

GGTGTGACTTCTCACAAATGTGGCAGATGGGAAGCTCGAATGGGGCAATTCGTTGGAAAT

AAGGCTTATGACAAGGCAGCCCTCAAATTTAACGGAAGAGAAGCAATGACCAATTTTGAG

CCTAGCATTTATGAAGGAGATATGATTTCCGATCCTAACAATGGAGGAAGCGGCCACAAT

CTTGATTTGAGCCTGGGAATTTCTCAGCCATCAAATGATCCCAAGGGGAATGACAACGTG

GGAGACCTCCATTCTCGTTATGGTGGTTTTGAAATACCCAGCAAAGAGAGACAAGTGGTG

GAGGGCACTGTTGCTGCACATATGGGTTTACAAACCCTTCATGGTTCACCAATGGCATCA

AAGAATTTTCCAGCATGGTCTGGCATATACCCTGGTTATTTATCAAGCTACGAGGAAAGG

GCTACGGAGAAGAGGGTGGAAGCTGTTTCTTCGCCGATATTCTCAAGCTGGCCGTGGCAA

ATAAATGGCAGCAACAGCGTTGTTGCTACAATGTCACAGCTCTCAGTTGCAGCATCATCA

GGATTCTCTGCTTCGACGATAACTGCCTCTTCAGCCACCCTTCCATTCAACCAGCAGAAC

CATCTCGCCGGGAACCTGCGTCTCTCTGCACCCACCACATCCGCCACCAACCCCTCTCTT

TACTCTTATAAGAGCTGA

>EVM0011459

ATGCAAACATCTTCAAAGAGGTCTAGAATCAGTGAAGCTCCCTCAGAAACTGTCTTTTCA

CCGCCGGCAGTTCCACCGCTAAGATTGACCTACGAGCAGGAGTTGGCTGTAATGGTTGCT

GCTCTAAAAAACGTTGTTTCTGGCACCGCTTCAATGGATTTCTCAAGGGAGATAAATAGT

ATCAACATGCCACTCAACGCTTCATATTCACAATTTGGAAGTACAAGCAGTGACGGGAAT

GATTTTTACAACTCTATTTTGCCACCATCTTCGGATCTTGACACGTGTGGTGTTTGCAAG

ATCAAAGGGTGCTTAGGATGCAACTTTTTCCCGCCAAATCAAGAAGACAAAAAGGATGAC

AAGAAAGGGAAGCGAAAGAGAGTAAAGAAGAATTATAGAGGTGTAAGGCAACGGCCATGG

GGAAAATGGGCTGCAGAGATAAGAGATCCACGGAAGGCGGCGAGGGTCTGGCTAGGGACG

TTTAACACTGCGGAGGAGGCGGCAAGGGCTTATGACAAGGCAGCCATTGATTTTAGAGGG

CCAAGAGCTAAGCTGAACTTCCCATTTCCTGATAGTGGCATTGCTAGTGTCGAAGAGAGT

AAAGAAGAGCAAGAAAAGCAGCAGGAAATCAGTGACACGAGAAGTGAGTTTGAAGCGGAA

AAGGGAGAAGACAACGAGTTCTGGGATAAGACTGGAGACGAAGAGTTAAAAGAATGGATG

ATGATGTTGATGGATTTTGGCAATGGCGACTCTTCCGATTGTGCTGGCACTGCAAGTGAT

GCTGCTCCTATTGGTTTTTAA

>EVM0028773

ATGGCTGCAGTTAAGAGTAGTATTGGAAAATCCAGGAAGGGTTTTGTTGATGAAACTCGA

AAGATGACGATGGAAGAAGATCGGCAGCTTGGTAGAGGGGGAAAAGAAGCTGATGTTAGC

TTCGAGAAGAGGCAATGGAAGCCGGTTATTGGTGAAGAATCCTTGTCGAACAGGCCTATG

AAGAAAACCTGTAGCCCTGTACGTCAAGAACAGATGCAATCCTCTGCATCTTCCGCTCAT

CAACCACCATCTTGTTTCTCTGTTTCTTCTACCTCTGCCTCGACTCTGTCTCTGTACCCG

CCTTCCTCGTCGCCTTCCCCTATCTCATCCTCAGGTTCTTCAAGTTCAAGAATTGTGTTT

CCTTTTGCCTTTGAAGGATCTAATCAAACTATTCAGTACCCTCAACAATTTAGAACACCC

CCTTCTTTGCCTATCTTCCATCCACTGTCTCAAGCTGCACAGAATCAACAGCAGATGATT

TCTTTTGGCCAAAACCAGCAGCCACATGGCATTGCATATCCTCCGTTTTTTGCTGGAGGA

TTAGCAATGGCTGACCACCAGCAGCAGCTGTTTCAGTACTGGAGTGATGCGTTGAACTTA

AGTCCGAGAGGAAGGATGATGATGATGAACAAGCTGGGGCCGGATGGGAGGCCAATGTTT

AGGCCTCCGATTCAGCCTATAAACTCAAGAAAGCTTTATAGGGGAGTGAGACAAAGGCAT

TGGGGGAAATGGGTTGCCGAGATTCGTCTCCCTCGAAACAGGACTCGCCTCTGGCTGGGC

ACATTTGATACAGCAGAAGATGCTGCCTTAGCTTATGATCGCGAGGCCTTCAAGTTAAGA

GGAGAGAATGCCAGGCTTAATTTCCCTGAACTTTTCCTCGGCAAAGATAAAGCAACTTCC

ACAGCTCCGAATTCAACAGTTTCTTCTCCGCCGACTCCCAATCAAAGTTCCAAGCCAAAA

GTAGCCCAAGAAGGCCTTAACTTGCTGGCAGAAACCATGTCACCACCAATATTACCACCA

CCGCCACCACCAGAGCAACCTCCAGGAGACCATCCTGATGATGATTGCGAGATGGGTTCG

AGTGGGGCTACGGTGAGTGGTGAGATTCAGGCAGTGGCAGAGGGGTCTAGTGCAGGGGAA

GGCATTCTGGGGTCTCAAGAATTGATGTGGGGAGACATGGCAGAAGCTTGGTATGATGCT

ATTCAATCCGGTTGGGGTCCAGGGAGTCCTGTGTGGGACGATTTGGACTCCACTAACAAT

CTTTTATTACAATCACATCTTCCTTTTGTTAATCCAAATCAACAGCAGTTTAATGATTCT

TGTGTTCTCCAAGACAACGTGGGCTCATCTTCTTCTTCCTCCTTCTTCCCCATGAAATCA

TTCTTCTTGAAGGATCAAGATTGA
